# Excessive activation of the RAS/MAPK pathway triggers adult-onset motor axonal degeneration

**DOI:** 10.1016/j.isci.2026.117507

**Published:** 2026-09-21

**Authors:** Marije Been, Erwan Lambert, Ana Serna, Anouk C.G. Balvert, Simon Heisinger, Merli Kreshpani, Catherine te Dorsthorst-Maas, Paulina Paškevičiūtė, Henar Rodríguez Arias, Danique Beijer, Galuh Astuti, Kevin Kenna, Boyd van Reijmersdal, Sharon Gloudemans, Pascal van Lith, Céline Sijlmans, Patrik Verstreken, Jan Veldink, Stephan Züchner, Christian Gilissen, Annette Schenck, Erik Storkebaum

**Affiliations:** 1 Molecular Neurobiology Laboratory, Donders Institute for Brain, Cognition and Behaviour and Faculty of Science, Radboud University, Nijmegen, the Netherlands; 2 Dr. John T. Macdonald Foundation Department of Human Genetics and John P. Hussman Institute for Human Genomics, University of Miami Miller School of Medicine, Miami, FL 33136, USA; 3 Department of Human Genetics, Radboud Institute for Molecular Life Sciences, Radboud University Medical Center, Nijmegen, the Netherlands; 4 Department of Neurology, UMC Utrecht Brain Centre, University Medical Centre Utrecht, Utrecht, the Netherlands; 5 Department of Human Genetics, Donders Institute for Brain, Cognition and Behaviour, Radboud University Medical Center, Nijmegen, the Netherlands; 6 VIB-KU Leuven Center for Neuroscience and Department of Neurosciences, Leuven Brain Institute, KU Leuven, 3000 Leuven, Belgium

**Keywords:** axonal degeneration, neurodegenerative disease, neurodevelopmental disease, forward genetic screen, RAS/MAPK pathway, RASopathy

## Abstract

Axonal and synaptic degeneration are key hallmarks of neurodegenerative diseases, but the molecular mechanisms remain incompletely understood. Here, we performed an unbiased forward genetic mosaic screen in *Drosophila* to identify genes required for maintenance of adult motor axons and neuromuscular junctions (NMJs). We identified 49 mutations in 30 genes, including 8 causing adult-onset progressive degeneration. Loss of *pebbled* (*peb*), the *DrosophilaRAS-responsive element binding protein 1* (*RREB1*) ortholog, causes adult-onset motor axonal and NMJ degeneration and age-dependent motor deficits. *peb* encodes a C_2_H_2_ zinc-finger transcription factor that negatively regulates transcription of RAS/MAPK pathway target genes, and its loss resulted in RAS/MAPK pathway overactivation. Loss of other RAS/MAPK pathway negative regulators also induced adult-onset progressive NMJ degeneration and motor deficits. Importantly, treatment of adult flies with the MEK1/2 inhibitor mirdametinib induced a dosage-dependent rescue of *peb* mutant motor neurodegenerative phenotypes. Thus, RAS/MAPK pathway overactivation triggers adult-onset progressive neurodegeneration, which can be prevented by RAS/MAPK pathway inhibition.

## Highlights

• *Drosophila* genetic screen identifies 30 genes required for adult axonal maintenance
• Loss of *pebbled* function triggers adult-onset motor axonal and NMJ degeneration
• RAS/MAPK pathway overactivation drives adult-onset motor neurodegeneration
• Pharmacological or genetic MEK inhibition rescues *peb* mutant motor neurodegeneration

## Graphical abstract

**Figure:**
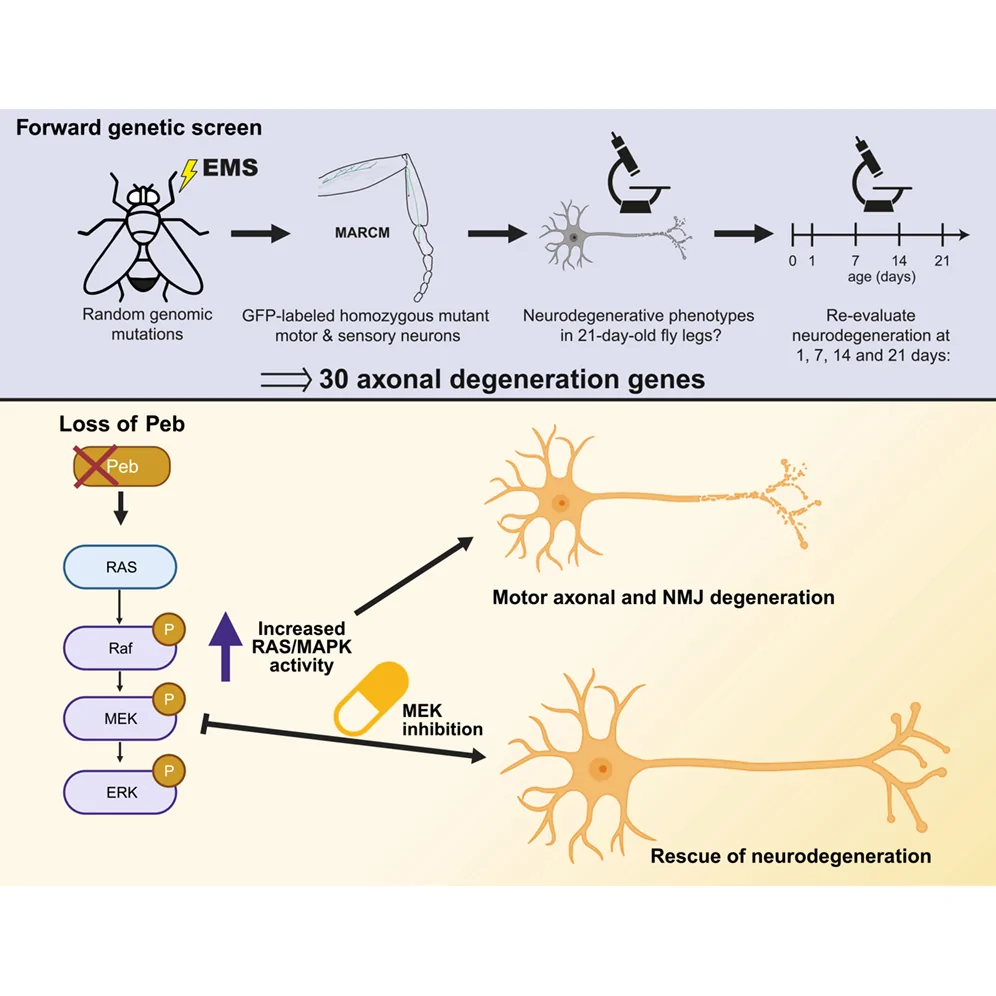

## Teaser

Biological sciences; Molecular neuroscience; Natural sciences; Neuroscience

## Introduction

Neurodegenerative disorders are typically adult-onset and progressive diseases, characterized by degeneration of specific neuronal populations. Axonal degeneration is a prominent pathological feature of many neurodegenerative diseases, with axons and presynaptic terminals often the neuronal compartments first affected.^1^ Neurodegenerative diseases have a high and increasing prevalence in the aging population, have devastating consequences for patients and their families, and result in high costs for society. The molecular mechanisms underlying neurodegenerative diseases are incompletely understood and, as a consequence, no or only minimally effective disease-modifying treatments are available for the vast majority of neurodegenerative diseases. Thus, there is an urgent need to gain deeper insight into the molecular mechanisms underlying neurodegeneration.

Here, we performed an unbiased, forward genetic mosaic screen to identify genes on the *Drosophila* X chromosome that are required for axonal maintenance. Ethyl methanesulfonate (EMS)-based forward genetic screens in *Drosophila* have been instrumental in uncovering the molecular mechanisms underlying key biological processes, including neuronal development, function, and maintenance.^2^^,^^3^^,^^4^ However, the screen presented here has a unique design allowing for the identification of gene mutations resulting in adult-onset degeneration of peripheral motor and sensory axons and neuromuscular junctions (NMJs) in the *Drosophila* leg. Our screen identified 30 genes that are involved in various molecular processes. Twenty nine (97%) of the identified genes have human orthologs, and for nineteen of these genes (66%), the human orthologs have previously been associated with neurodegenerative and/or neurodevelopmental disorders, confirming the disease relevance of our screen.

One of the identified genes is *pebbled* (*peb*), the *Drosophila* ortholog of human *RAS-responsive element binding protein 1* (*RREB1*). *Peb* and *RREB1* encode C_2_H_2_ zinc-finger transcription factors, and RREB1 negatively regulates the transcription of RAS/MAPK pathway target genes through binding to RAS-response elements (RREs) in their promoters.^5^ Haploinsufficiency of *RREB1* causes a Noonan-like RASopathy in humans and mice.^5^^,^^6^^,^^7^ RASopathies, including Noonan syndrome, affect ∼1/1,000 individuals and are characterized by short stature, facial dysmorphism, intellectual disability, and cardiac abnormalities.^8^^,^^9^ RASopathies are caused by germline mutations in genes encoding components or regulators of the RAS/MAPK pathway,^10^^,^^11^ either activating mutations in RAS/MAPK pathway components or loss-of-function mutations in negative regulators of the RAS/MAPK pathway. The latter category includes neurofibromatosis type 1 (NF1), Legius syndrome, and *LZTR1*-associated Noonan syndrome. NF1 is caused by heterozygous loss-of-function mutations in the *NF1* gene, which encodes neurofibromin, a RAS GTPase-activating protein (GAP) that stimulates the conversion of active RAS-GTP to inactive RAS-GDP.^11^ Legius syndrome is caused by heterozygous inactivating mutations in *SPRED1*, a negative regulator of the RAS/MAPK pathway, by inhibiting phosphorylation of Raf.^8^^,^^9^^,^^11^ Heterozygous and homozygous *LZTR1* mutations can cause Noonan syndrome. LZTR1 functions as a substrate receptor for cullin3 (CUL3)-RING ubiquitin ligase (CRL3) complexes.^12^ Because LZTR1 substrates include RAS and RIT1, LZTR1 enables RAS and RIT1 ubiquitination and subsequent degradation.^13^ The Noonan syndrome causative mutations impede LZTR1 substrate recognition, thus resulting in excessive RAS/MAPK pathway activation. Interestingly, excessive activation of the RAS/MAPK pathway was suggested to be implicated in LRRK2-associated Parkinson’s disease (PD),^14^ but *in vivo* evidence for a causative link between the RAS/MAPK pathway and neurodegeneration is missing.

We discovered that loss of function of *peb*, as well as other negative regulators of the RAS/MAPK pathway such as *Neurofibromin 1* (*Nf1*), *Spred*, *and Lztr1*, caused adult-onset progressive degeneration of motor axons and NMJs and resulted in age-related, progressive motor deficits. These findings identify overactivation of the RAS/MAPK pathway as a novel molecular mechanism underlying adult-onset progressive neurodegeneration. Importantly, the small molecule MEK1/2 inhibitor mirdametinib fully rescued the *peb* mutant axonal degeneration phenotype, motor deficit, and the downstream molecular defects in α-tubulin acetylation and autophagy. This indicates that pharmacological inhibition of the RAS/MAPK pathway could be explored as a novel therapeutic approach to mitigate progressive axonal degeneration in RASopathies and neurodegenerative diseases.

## Results

### An unbiased forward genetic screen of the *Drosophila* X chromosome identifies genes required for maintenance of peripheral motor axons

To identify essential genes required for axonal maintenance, we performed an unbiased, forward genetic mosaic screen of the *Drosophila* X chromosome. Using a low concentration of the chemical mutagen EMS to introduce random genomic mutations on a FRT19A chromosome, we established 8,892 stocks, of which 1,416 displayed hemizygous male lethality (Figures 1A and S1A–S1D). This corresponds to a saturation of approximately 88% of the X chromosome (see STAR Methods). To generate GFP-labeled motor and sensory neuron homozygous mutant clones in otherwise heterozygous flies, we applied mosaic analysis with a repressible cell marker (MARCM),^15^ using *asense-FLP*^16^ and *OK371-GAL4* to express membrane-tethered GFP (UAS-mCD8::GFP) in glutamatergic neurons, which include motor and sensory neurons in the *Drosophila* leg (Figures 1B and S1E). For each mutant line, we screened the legs of at least two 21-day-old flies for any motor neuron morphology defects using fluorescence microscopy (Figure S1F). This approach identified motor axonal and NMJ phenotypes in 130 mutant lines, including 58 lines in which no GFP-positive motor or sensory neurons were visible at days 1 and 21. These 58 lines presumably contain cell lethal mutations and were kept for mapping purposes (Figure S2; Table S1). In the remaining 72 mutants, both motor axonal and NMJ phenotypes were present (Figure 1C).

**Figure 1:**
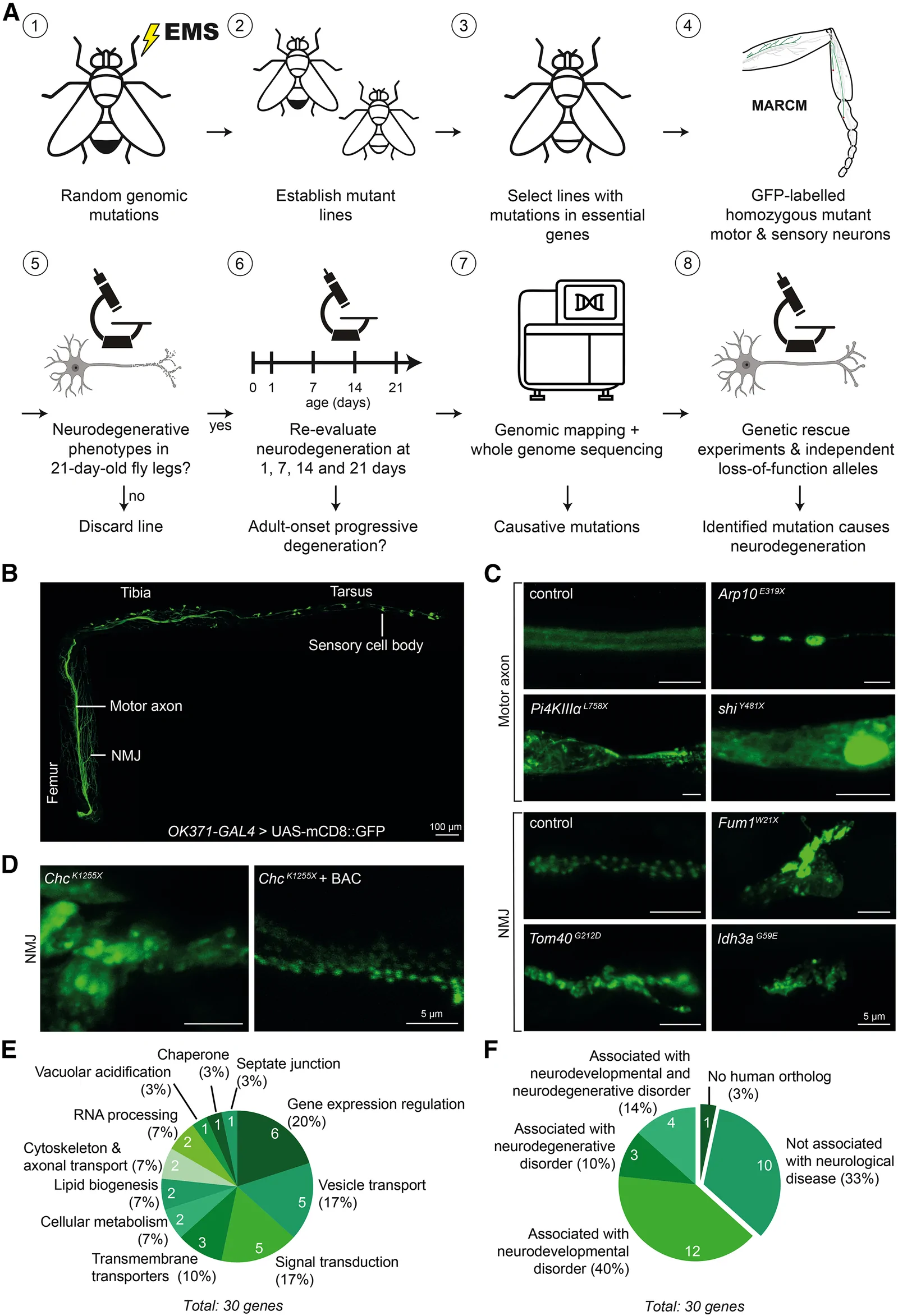
An unbiased forward genetic screen of the *Drosophila* X chromosome identifies genes involved in axonal maintenance (A) Flow chart of the genetic screening approach. See text for details. (B) Visualization of glutamatergic neurons in the fly leg, using *OK371-GAL4* to drive expression of membrane-bound GFP (mCD8::GFP). In the leg, glutamatergic neurons include motor neurons innervating leg muscles and sensory neurons projecting to the central nervous system. Scale bar, 100 μm. (C) Representative examples of motor axonal and NMJ degenerative phenotypes as compared to control (FRT19A). Note that control images are shared with Figures 2A, 3B, and 3C as all data were acquired in a single experiment using the same control samples. (D) Phenotypic rescue of *Chc*^*K1255X*^ NMJ phenotype by re-introduction of wild-type *Chc* in a BAC-transgene. Scale bars: 5 μm in (C) and (D). (E and F) Pie charts showing (E) the molecular function and (F) the disease-association of the genes identified in the screen. See also Figures S1, S2, and Table S1. *n* = (C and D) at least 6 legs of 6 flies per genotype.

Duplication and complementation mapping were performed, along with whole genome sequencing to identify the causative mutations (Figures S1G and S2; Table S1). To confirm that the identified gene mutation is responsible for the neuronal morphology defect, we evaluated whether a BAC transgene containing the wild-type gene rescued the degenerative phenotype (Figure 1D; Table S1). This approach identified 49 causative mutations in 30 genes (Table 1; Table S1), which are involved in diverse molecular processes (Figure 1E). Some of these pathways, such as gene expression regulation, vesicle transport, and cellular metabolism, have already been associated with neurodegeneration.^1^ In contrast, other identified pathways have not previously been linked to neuronal phenotypes. For example, the link between *Drosophila* smooth septate junctions and neurodegeneration remains to be characterized.

**Table 1:** List of fly genes identified in the screen, their human orthologs, and the molecular process they are involved in; see also Table S2

Fly gene	Human ortholog(s)	Molecular function
*Arp10*	*ACTR10*	cytoskeleton and axonal transport
*Arp8*	*ACTR8*	gene expression regulation
***AP-1-2β***	***AP1B1*** and *AP2B1*	vesicle transport
*AMPKα*	*PRKAA1* and *PRKAA2*	signal transduction
***βCOP***	***COPB2***	vesicle transport
***CkIIβ***	***CSNK2B***	signal transduction
***CG15890***	***SLC46A1***, *SLC46A2*, and ***SLC46A3***	transmembrane transporter
*CCT6*	***CCT6A*** and *CCT6B*	chaperone
***Chc***	***CLTC*** and *CLTC1*	vesicle transport
***comt***	***NSF***	vesicle transport
*crn*	*CRNKL1*	RNA processing
*egh*	none	lipid biogenesis
***Fum1***	***FH***	cellular metabolism
*Hr4*	*NR6A1*	signal transduction
***Idh3a***	***IDH3A***	cellular metabolism
***kz***	***DHX37***	RNA processing
***Nej***	***CREBBP*** and ***EP300***	gene expression regulation
***peb***	***RREB1***	gene expression regulation
***Pi4KIIIα***	***PI4KA***	lipid biogenesis
***Rala***	***RALA*** and *RALB*	signal transduction
***Rbcn-3B***	***WDR7*** and ***WDR72***	vacuolar acidification
***RpII215/Polr2A***	***POLR2A***	gene expression regulation
***shi***	***DNM1***, ***DNM2***, and *DNM3*	vesicle transport
*Sas10*	*UTP3*	gene expression regulation
*sno*	*SBNO* and *SBNO2*	signal transduction
*Tsp2A*	*TSPAN3*, *TSPAN8*, *TSPAN10*, *TSPAN16*, *TSPAN19*, and *TSPAN32*	septate junction
*Tim9a*	*TIMM9*	transmembrane transporter
***Tom40***	***TOMM40*** and *TOMM40L*	transmembrane transporter
***trr***	***KMT2C*** and ***KMT2D***	gene expression regulation
***β-Spec***	***SPTB***, ***SPTBN1***, ***SPTBN2***, and ***SPTBN4***	cytoskeleton and axonal transport

Human genes and the corresponding fly genes that are associated with disease are shown in bold.

Twenty nine (97%) of the identified genes have at least one human ortholog, corresponding to a total of 51 orthologous human genes. Nineteen (66%) of the *Drosophila* genes have human orthologs that have been associated with neurological disease (Figure 1F; Tables 1 and S2). By screening aged flies, we aimed to enrich for genes involved in neurodegeneration rather than genes involved in neuronal development. Surprisingly, only 3 (10%) of the identified *Drosophila* genes have orthologs that have exclusively been associated with neurodegenerative disease, while another 4 (14%) genes have orthologs that have been associated with both neurodevelopmental and neurodegenerative disorders. Furthermore, 12 (40%) genes have orthologs associated with neurodevelopmental disorders (Figure 1F; Table S2). Twelve (40%) of the thirty genes had not been identified in a previously reported X chromosome screen for genes with essential functions in neuronal development, function, and maintenance,^4^ indicating that our unique screening strategy for adult-onset progressive degeneration was successful in identifying novel axonal degeneration genes, while confirming others.

### Phenotypic characterization of the identified axonal degeneration mutants

Next, we phenotypically characterized the identified mutant lines in more detail. Lethal stage determination of the 46 non-cell lethal lines revealed that hemizygous males display embryonic lethality in 23 (50%), larval lethality in 21 (46%), and pupal lethality in 2 (4%) lines (Table S3). Remarkably, for only 5 (18%) of 28 axonal degeneration genes evaluated, transgenic RNAi-mediated knockdown in motor neurons (*OK371-GAL4*) induced a neurodegenerative phenotype similar to the mutant (Table S3). For 6 (21%) of the identified genes, transgenic RNAi knockdown induced a very mild phenotype, whereas for the remaining 17 (61%) genes, RNAi knockdown did not induce axonal or NMJ morphological phenotypes. This is likely attributable to incomplete RNAi-mediated knockdown that induces partial loss-of-function, whereas homozygous damaging mutations may induce full loss-of-function.

To discriminate between developmental and adult-onset phenotypes, we repeated the MARCM analysis for the 46 non-cell lethal mutant lines and examined motor axonal and NMJ morphology at 1, 7, 14, and 21 days of age (Figure 2A; Table S3). Representative images for all non-cell lethal mutant lines are shown in Data S1. Mutants were categorized as (1) developmental non-progressive when the phenotype was present on day 1 and did not become more pronounced with aging, (2) developmental progressive when the phenotype was present on day 1 and progressed with aging, and (3) adult-onset progressive when phenotype onset was after day 1 (Figure 2A). While the majority of the mutants displayed a developmental phenotype, mutations in 8 (29%) of the 28 non-cell lethal genes resulted in an adult-onset progressive phenotype (Figure 2B; Table S3). Remarkably, mutations in the majority of the 15 non-cell lethal genes for which the human orthologs are associated with neurodevelopmental disorders resulted in progressive neurodegeneration (Figure 2C). Indeed, 9 (60%) of the 15 genes showed a developmental progressive phenotype, while only 4 (27%) showed a developmental non-progressive phenotype. Mutations in 2 genes, Ras-like protein A (*Rala*) and *peb*, which regulate neurodevelopment in other contexts,^5^^,^^17^^,^^18^ showed an adult-onset progressive phenotype in the NMJ. These data suggest that many neurodevelopmental genes also play an important role in axonal maintenance.

**Figure 2:**
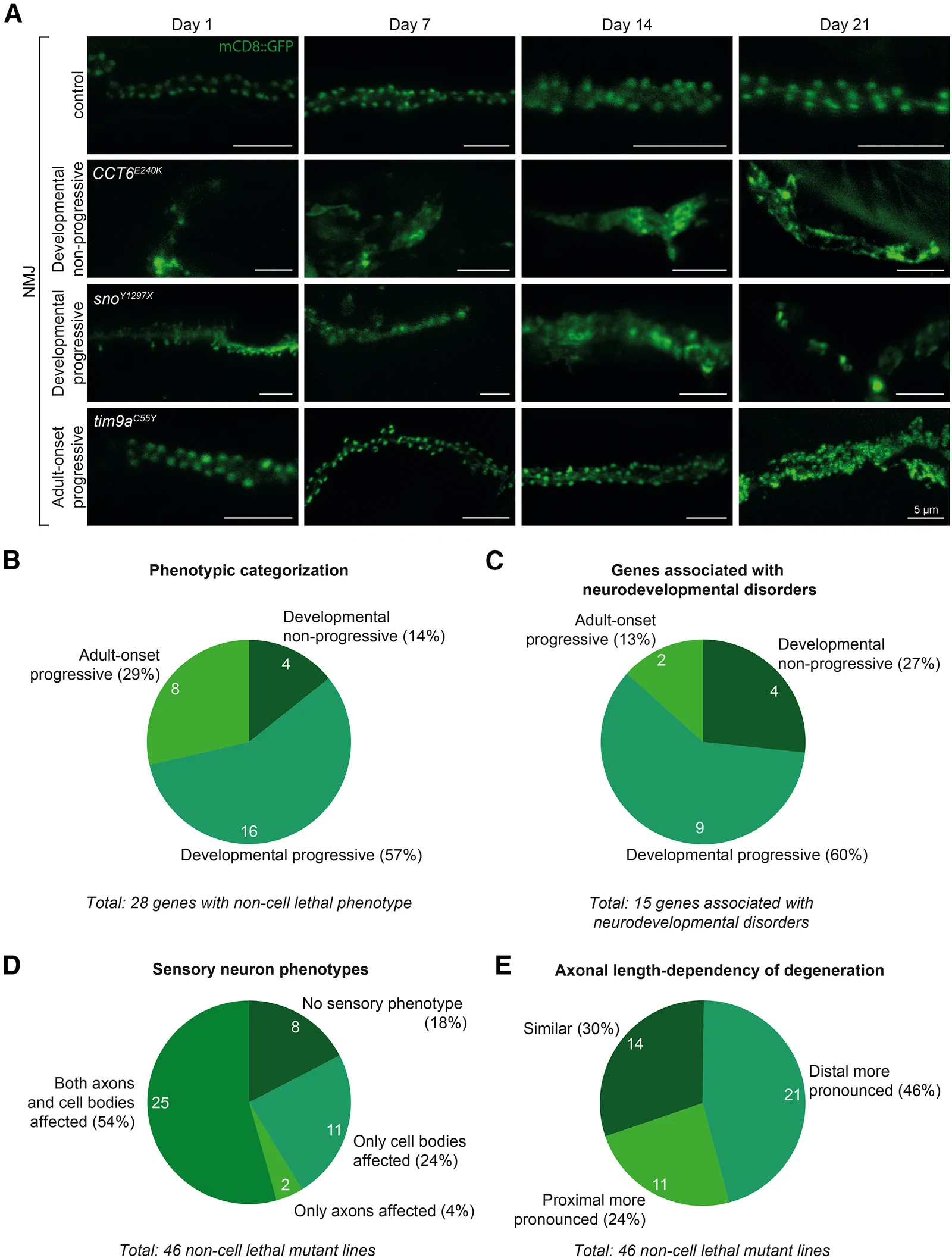
Phenotypic categorization of the identified axonal degeneration mutants (A) Time course of NMJ phenotypes in the femur of FRT19A control and representative mutants. Three phenotypic categories were distinguished: (1) phenotypes present on day 1 that do not progress with aging are classified as developmental non-progressive (e.g., *CTT6*^*E240K*^); (2) developmental phenotypes that degenerate further with age are developmental progressive (e.g., *sno*^*Y1297X*^); (3) mutants with a phenotype onset after day 1 are adult-onset progressive (e.g., *tim9a*^*C55Y*^). Scale bars, 5 μm. Note that control images are shared with Figures 1C, 3B, and 3C as all data were acquired in a single experiment using the same control samples. (B and C) Pie charts showing the time course phenotypic categorization of (B) all identified non-cell lethal genes and (C) mutants in genes associated with neurodevelopmental disorders. (D) Pie chart showing the sensory neuron phenotypes of the identified non-cell lethal mutant lines. (E) Pie chart showing axonal length-dependent degeneration characteristics of the identified non-cell lethal mutant lines, evaluated by comparing distal (tibia) versus proximal (femur) motor axonal and NMJ phenotypes. See also Table S3 and Data S1. *n* = (A) at least 6 legs of 6 flies per genotype.

We further assessed all mutants for morphological phenotypes in sensory cell bodies and axons in the *Drosophila* leg (Figure 2D; Table S3, Data S1). Interestingly, 8 (17%) of the 46 non-cell lethal mutants, including mutations in *Arp8*, *CkIIβ*, *CCT6*, *Hr4*, *Idh3a*, *Polr2A*, *peb*, and *Sas10*, did not show sensory neuron morphological defects, suggesting their selective requirement for motor neuron maintenance. The majority of the remaining mutants displayed morphological changes in both sensory axons and cell bodies, but in some mutants, the sensory neuron cell body or axon were selectively affected (Figure 2D; Table S3, Data S1). Finally, we compared the phenotypic severity of motor axon and NMJ degeneration in the femur and tibia to evaluate possible axonal length-dependent degeneration (the tibia is distal to the femur). This revealed that about half of the non-cell lethal mutants (21 out of 46, 46%) displayed more pronounced phenotypes in axons and NMJs innervating distal muscles, while 30% (14 out of 46) of the mutants displayed similar phenotypic severity in femur and tibia, and 24% (11 out of 46) displayed more pronounced proximal defects (Figure 2E; Table S3, Data S1).

### Loss of *peb* function results in adult-onset progressive motor neuron degeneration

One of the mutants we identified carries a nonsense mutation in *peb* (*peb*^*Q894X*^) (Figures 3A and S3A), encoding a C_2_H_2_ zinc-finger transcription factor that is orthologous to human *RREB1*. Homozygosity for *peb*^*Q894X*^ resulted in adult-onset progressive degeneration of motor axons and NMJs (Figures 3B–3D). We observed accumulation of GFP-positive puncta (labeled by membrane-bound mCD8::GFP) in motor axons by day 7, and NMJ degeneration was evident by day 14 (Figure S3B). An 80 kb BAC transgene containing a wild-type copy of *peb* (*peb*^*BAC*^) fully rescued *peb*^*Q894X*^ motor axonal and NMJ phenotypes (Figures 3E and 3F), thus confirming that these phenotypes are caused by loss of *peb* function. Furthermore, homozygosity for two additional *peb* loss-of-function alleles, *peb*^*E8*^^19^ and *peb*^*345x*^,^20^ resulted in a similar but more pronounced phenotype than the *peb*^*Q894X*^ mutant (Figures 3B–3D) that was fully rescued by *peb*^*BAC*^ (Figure 3F). This suggests that *peb*^*Q894X*^ is a partial loss-of-function (hypomorphic) allele, whereas *peb*^*E8*^ and *peb*^*345x*^ were reported to be full loss-of-function (amorphic) alleles.^20^^,^^21^

**Figure 3:**
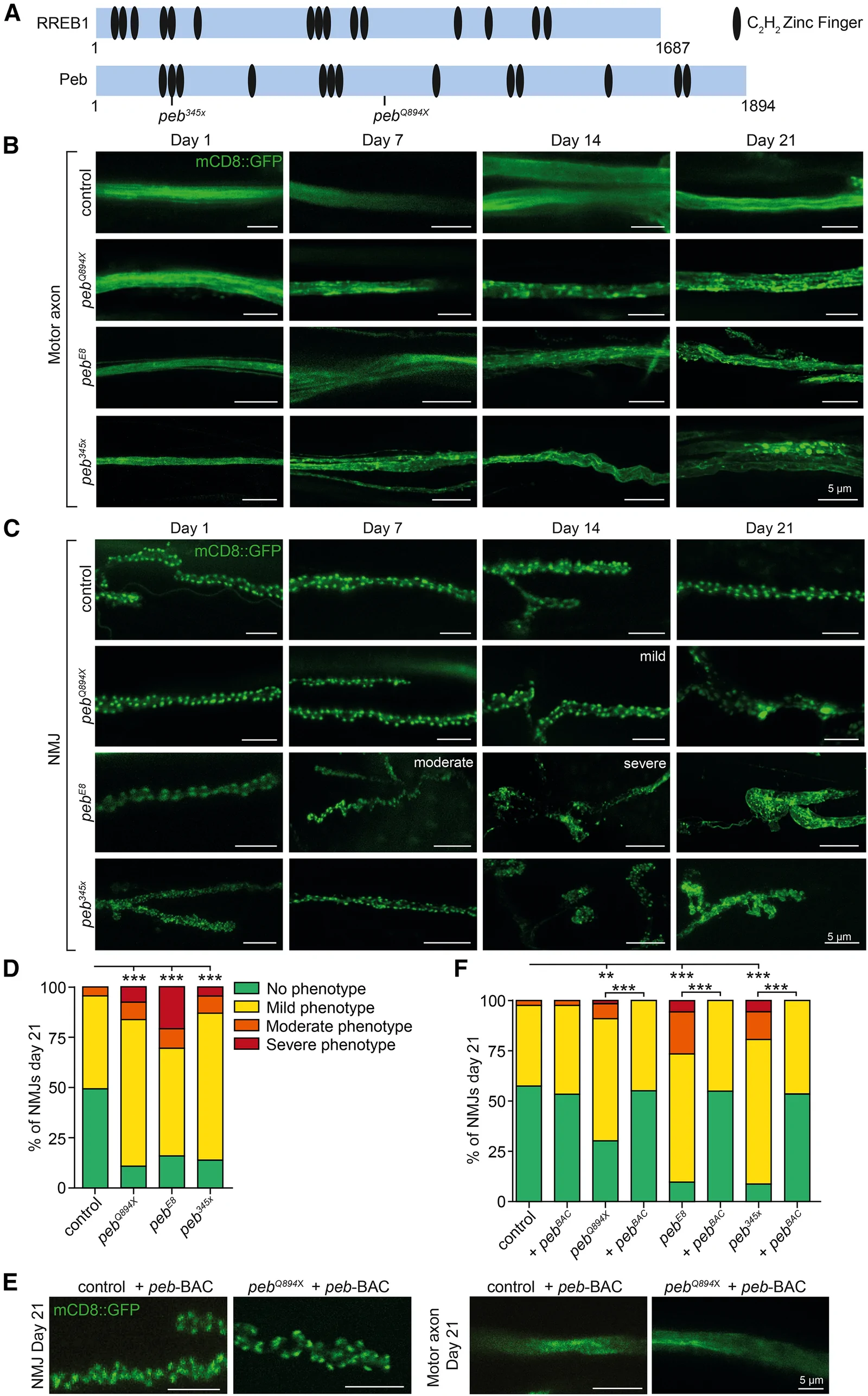
Loss of *pebbled* function results in adult-onset neurodegeneration (A) Schematic representation of Peb protein and its human ortholog RREB1, showing the location of the C_2_H_2_ zinc-finger domains and the *peb*^*345x*^ and *peb*^*Q894X*^ mutations. (B and C) Time course of (B) motor axon and (C) NMJ morphology in the femur of *peb*^*Q894X*^, *peb*^*E8*^, and *peb*^*345x*^ mutants versus control (FRT19A). Note that control images are shared with Figures 1C and 2A as all data were acquired in a single experiment using the same control samples. (D) Semi-quantitative analysis of NMJ degeneration in the femur of three independent *peb* mutants versus control (FRT19A). NMJ phenotype was scored as no, mild, moderate, or severe degeneration, as illustrated in (C). (E and F) (E) Representative images and (F) semi-quantitive analysis of rescue of *peb* mutant NMJ and axon morphology defects at 21 days of age by a BAC transgene containing the wild-type *peb* gene (*peb*-BAC). ∗∗*p* < 0.01, ∗∗∗*p* < 0.0001 by (D and F) Fisher’s exact test with Bonferroni correction. *n* = (B and C) at least 6 legs of 6 flies per genotype, (D) 38–123 NMJs of 6–10 animals per genotype, and (E andF) 71–107 NMJs of 6–10 animals per genotype. Data are represented as mean ± SEM. Scale bars, 5 μm. See also Figure S3.

Interestingly, loss of *peb* function did not affect sensory neuron morphology (Figure 4A), suggesting that motor neurons may be particularly sensitive to loss of *peb* function in *Drosophila*. Consistent with this notion, homozygous mutant clones of both the hypomorphic *peb*^*Q894X*^ and amorphic *peb*^*E8*^ in mushroom body β-lobe neurons did not display axonal degeneration in 21-day-old flies (Figure 4B), despite the fact that Fly Cell Atlas^22^ shows *peb* co-expression with the known mushroom body markers *FasII*, *drk*, *Pka-R2*, and *dco*.^23^

**Figure 4:**
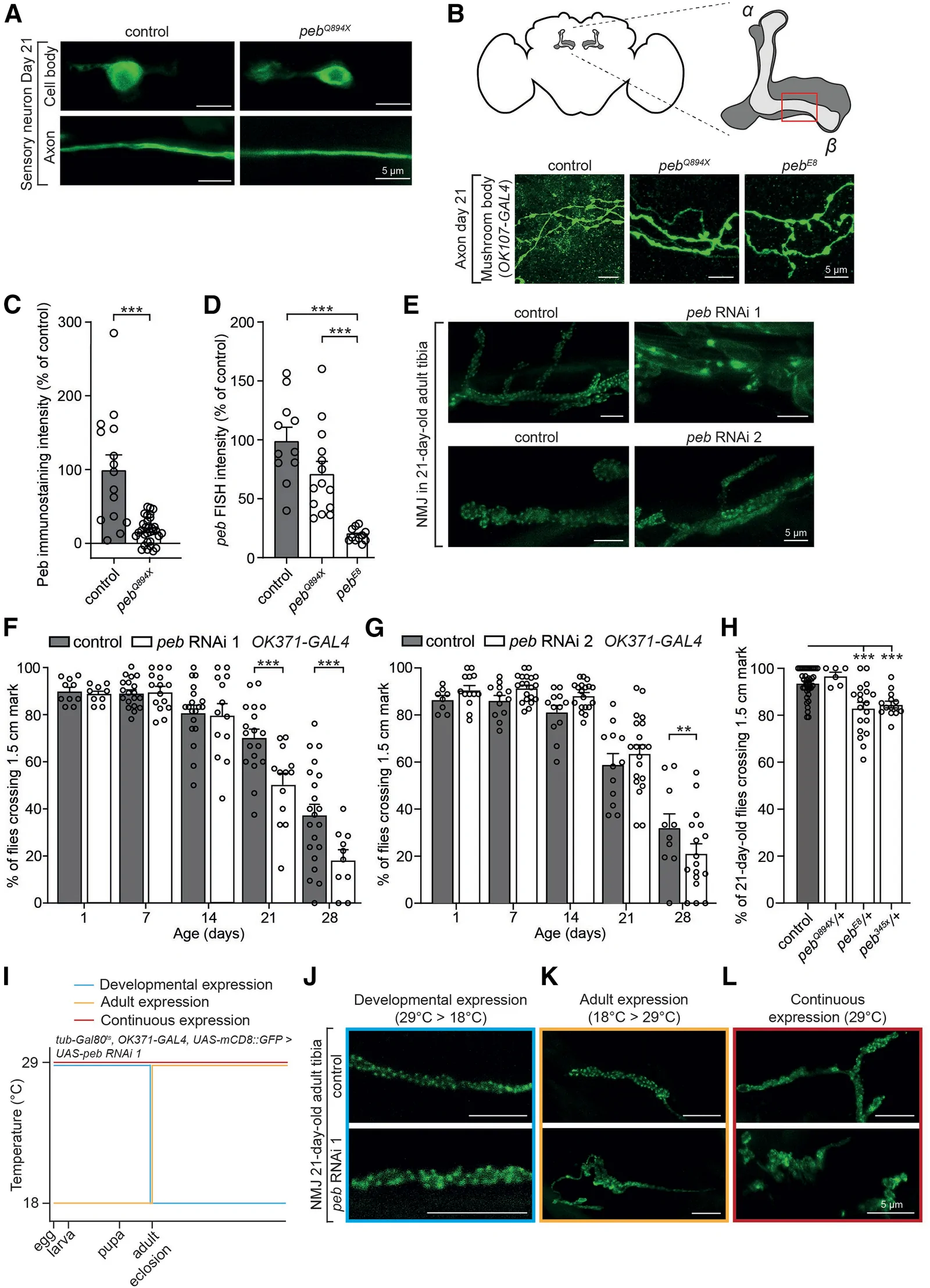
Partial loss of *peb* function induces NMJ degeneration and late-onset motor deficits (A) Representative images of cell bodies and axons of control (FRT19A) and *peb*^*Q894X*^ sensory neurons at 21 days of age. (B) Representative images of axons in control (FRT19A) and *peb* mutant MARCM clones in the β-lobe of the mushroom body (*OK107-GAL4*). Axons were imaged within the area indicated by the red square. (C) Quantification of Peb immunostaining intensity in cell bodies of control (FRT19A) versus *peb*^*Q894X*^ motor neuron clones in adult VNC at 21 days of age. Data were normalized to a control without peb antibody and are shown as percentage of control. (D) Quantification of signal intensity of fluorescent *in situ* hybridization (FISH) for peb transcript in cell bodies of *peb* mutant motor neuron clones in the adult VNC at 21 days of age. Data are shown as percentage of control (FRT19A). (E) Representative images of tibial NMJs of 21-day-old flies with *peb* knockdown in motor neurons (*OK371-GAL4*). Controls are driver-only in the corresponding genetic backgrounds (KK for *peb* RNAi 1 and GD for *peb* RNAi 2). (F–H) Motor performance in a negative geotaxis climbing assay, shown as percentage of flies crossing a 1.5 cm mark. (F and G) Flies with *peb* knockdown in motor neurons (*OK371-GAL4*) are compared to driver-only control in the corresponding genetic background (KK for *peb* RNAi 1 and GD for *peb* RNAi 2) at 1, 7, 14, 21, and 28 days of age using two independent RNAi lines. (H) Flies with heterozygous mutations in *peb* are compared to control (FRT19A) at 21 days of age. (I–L) (I) Schematic representation of experimental approach and (J–L) representative images of tibial NMJs in 21-day-old animals with *peb* knockdown in motor neurons (*OK371-GAL4*), either only during development (J), adulthood (K), or both (L). A temperature-sensitive GAL80 transgene was used to gain temporal control over *peb* RNAi transgene expression. Control represents *OK371-GAL4* in KK genetic background. ∗∗*p* < 0.01, ∗∗∗*p* < 0.005 by (C) unpaired *t* test with Welch’s correction, (D and H) Brown-Forsythe and Welch ANOVA with Dunnett’s T3 multiple comparisons test, and (F and G) two-way ANOVA with Šidák’s multiple comparisons test. *n* = (A, E, and J–L) at least 6 legs of 6 flies per genotype, (B) 3–4 brains per genotype, (C) 15–31 cells in 3 animals per genotype, (D) 11–14 animals per genotype, (F) 9–21 groups of 10 flies per genotype, (G) 8–20 groups of 10 flies per genotype, and (H) 6–41 groups of 5–10 flies per genotype. Data are represented as mean ± SEM. Scale bars, 5 μm. See also Figure S4.

We next evaluated the effect of the *peb*^*Q894X*^ mutation on Peb protein levels. Immunohistochemistry on *peb*^*Q894X*^ homozygous mutant versus wild-type motor neuron clones, using the monoclonal anti-Peb antibody 1G9 (Developmental Studies Hybridoma Bank [DSHB), revealed significantly reduced Peb staining intensity in *peb*^*Q894X*^ clones by ∼80% (Figures 4C and S3C). Because the 1G9 epitope is between amino acids 824 and 1,125, this Peb antibody may still detect truncated Peb protein produced by the *peb*^*Q894X*^ allele. To evaluate this possibility, we performed fluorescent *in situ* hybridization on ventral nerve cords (VNCs) of 21-day-old *peb*^*Q894X*^ and *peb*^*E8*^ MARCM flies. In homozygous mutant motor neuron clones, *peb*^*Q894X*^ mRNA levels were not significantly reduced as compared to control, whereas *peb*^*E8*^ mRNA levels were very low (Figures 4D and S3D). Thus, it is conceivable that the milder *peb*^*Q894X*^ neurodegenerative phenotype is due to the production of truncated Peb protein, which may be partially functional.

Next, we evaluated whether transgenic RNAi-mediated knockdown of *peb* mimics the mutant phenotype. Expression of two independent *peb* RNAi transgenes in motor neurons (*OK371-GAL4*) resulted in NMJ degeneration in the tibia of 21-day-old animals (Figure 4E), with *peb* RNAi 1 inducing more pronounced defects. To determine whether the NMJ morphology defects in *peb* knockdown animals resulted in motor deficits, we performed a negative geotaxis climbing assay.^24^ Both *peb* RNAi lines resulted in significant motor performance defects from 21 (*peb* RNAi 1) or 28 (*peb* RNAi 2) days of age onward (Figures 4F and 4G). In addition, heterozygous *peb*^*E8/+*^ and *peb*^*345x/+*^, but not *peb*^*Q894X/+*^ females, displayed motor performance deficits at 21 days of age (Figure 4H). Thus, loss of *peb* function results in progressive motor axonal and NMJ degeneration as well as motor deficits.

Finally, to confirm that adult NMJ phenotypes induced by *peb* knockdown in motor neurons are attributable to a role of Peb in adult axonal maintenance, rather than during development, we investigated the morphology of the NMJ on muscle 8 of third instar larvae (Figure S4A). We did not observe changes in NMJ length (Figures S4B and S4C), the distribution of the synaptic vesicle protein Synapsin (Figure S4D), the SNARE-binding protein Ras opposite (Rop) (Figure S4E), or the number of active zones (Figures S4F and S4G). Furthermore, using a temperature-sensitive GAL80 to gain temporal control over *peb* knockdown (Figure 4I), we found that *peb* knockdown in motor neurons only during development did not result in tibial NMJ degeneration at 21 days of age (Figure 4J), whereas *peb* knockdown only during adulthood was sufficient to induce NMJ degeneration (Figure 4K), similar to continuous knockdown throughout development and adulthood (Figure 4L). Together, these data demonstrate that Peb is required for adult axonal maintenance.

### *peb* mutant axonal degeneration is independent of apoptosis, the JNK signaling pathway, and Wallerian degeneration

Next, we explored possible molecular mechanisms by which loss of *peb* function induces axonal degeneration. Peb was shown to be a negative regulator of Jun kinase (JNK) signaling,^25^ a stress-signaling pathway that can induce apoptosis. Therefore, we performed the TUNEL (Terminal deoxynucleotidyl transferase dUTP nick end labeling) assay to detect apoptosis in homozygous *peb* mutant motor neuron clones in the VNC of 21-day-old flies and found no differences in the number of TUNEL-positive nuclei compared to control (Figure S5A). To exclude the possibility that *peb* mutant motor neurons have died before 21 days of age, we quantified the number of motor neurons in the motor neuron clusters in the VNC that innervate the leg muscles^26^ (Figure S5B) in 21-day-old animals with *peb* knockdown in motor neurons (*OK371-GAL4*) and found no difference as compared to control (Figure 5A). Furthermore, expression of a baculovirus anti-apoptotic p35 protein did not rescue *peb* mutant NMJ morphology defects (Figure 5B). Finally, heterozygosity for a loss-of-function allele of the JNK ortholog *bsk* did not modify *peb* mutant NMJ degeneration (Figure S5C). Together, these data indicate that *peb* mutant motor axonal degeneration is caused by a molecular mechanism independent of apoptosis or the JNK signaling pathway.

**Figure 5:**
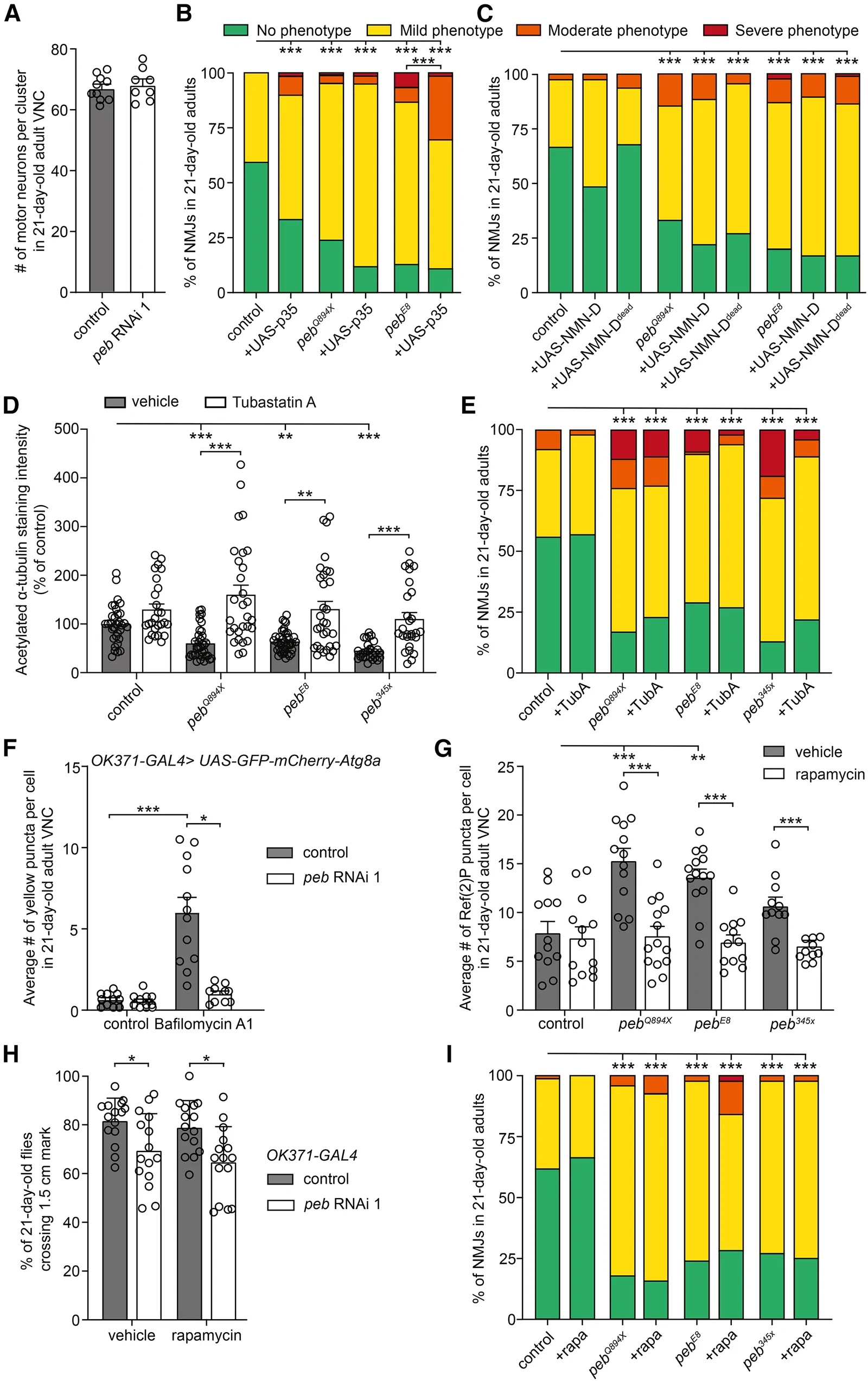
Apoptosis, Wallerian degeneration, defective α-tubulin acetylation, or autophagy do not underlie *peb* mutant phenotypes (A) Quantification of the number of motor neurons per cluster in 21-day-old adult VNCs with *peb* knockdown in motor neurons (*OK371-GAL4*) versus driver-only control (KK background). (B and C) Semi-quantitative analysis of femoral NMJ degeneration in control (FRT19A) and *peb* mutant motor neuron clones of 21-day-old animals expressing (B) the anti-apoptotic protein p35 or (C) the Wallerian degeneration suppressor NMN-D or NMN-D^dead^. (D) Quantification of immunostaining intensity of acetylated α-tubulin in control (FRT19A) versus *peb* mutant motor neuron clones in the VNC of 21-day-old flies treated with 10 μM Tubastatin A (TubA) or vehicle. Data were normalized to control. (E) Quantification of the effect of 10 μM TubA treatment versus vehicle on NMJ degeneration in control (FRT19A) versus *peb* mutant motor neuron clones in the femur of 21-day-old flies. (F) Quantification of the effect of *ex vivo* Bafilomycin A1 treatment versus vehicle on the average number of GFP-mCherry-Atg8a yellow puncta per motor neuron (*OK371-GAL4*) in the VNC of 21-day-old control (driver-only in KK genetic background) versus *peb* knockdown flies expressing the GFP-mCherry-Atg8a autophagy marker. (G) Quantification of the number of Ref(2)P-positive puncta in *peb* mutant versus control (FRT19A) motor neuron clones in the VNC of 21-day-old adult flies treated with either 50 μM rapamycin or vehicle. (H) Negative geotaxis motor performance (% of flies crossing a 1.5 cm mark) of 21-day-old flies with *peb* knockdown in motor neurons (*OK371-GAL4*) versus driver-only control (KK background), either treated with vehicle or 50 μM Rapamycin. (I) Semi-quantitative analysis of femoral NMJ degeneration in control (FRT19A) and *peb* mutant motor neuron clones of 21-day-old animals treated with either 50 μM rapamycin or vehicle. ∗*p* < 0.05, ∗∗*p* < 0.01, ∗∗∗*p* < 0.005 by (A) unpaired *t* test (B, C, E, and I) Fisher’s exact test with Bonferroni correction, (D and F) Kruskal-Wallis test with Dunn’s multiple comparisons test, (G) Brown-Forsythe and Welch ANOVA with Dunnett’s T3 multiple comparisons test, (H) two-way ANOVA with Šídák’s multiple comparisons test. *n* = (A) 8–10 animals per genotype, (B) 88–116 NMJs in 6–10 animals per genotype, (C) 53–131 NMJs in 6–10 animals per genotype, (D) 8–26 animals per experimental group, (E) 32–182 NMJs of 6–10 animals per experimental group, (F) 6 cells in 10–12 animals per experimental group, (G) 10–14 animals per experimental group, (H) 15 groups of 5–10 animals per experimental group and (I) 54–159 NMJs in 6–10 animals per experimental group. Data are represented as mean ± SEM. See also Figures S5 and S6.

Peb was further reported to be required for Wallerian degeneration, the programmed degeneration of the part of an axon distal to a nerve injury.^27^ Indeed, loss of *peb* function prevents axonal death in wing sensory axons after axotomy.^20^ To evaluate the possible involvement of the axon death pathway in *peb* mutant motor axonal degeneration, we generated *peb* mutant motor neuron clones while simultaneously altering the levels of dSarm, a conserved, pro-degenerative factor central to Wallerian degeneration that promotes axonal self-destruction through NAD^+^ depletion. Genetic modulation of dSarm levels was previously shown to modify *peb* mutant axonal death.^20^ Neither heterozygosity for the *dsarm*^*896*^ loss-of-function allele nor dSarm overexpression modified the NMJ degenerative phenotype of homozygous *peb*^*Q894X*^ or *peb*^*E8*^ motor neuron clones (Figures S5D and S5E). Furthermore, we expressed PncC, a bacterial enzyme with NMN-deamidase (NMN-D) activity that converts the dSarm-activating NMN (nicotinamide mononucleotide) to inactive NAMN (nicotinic acid mononucleotide), thus preventing dSarm activation and degeneration of severed axons.^28^ NMN-D expression did not rescue *peb* mutant NMJ degeneration (Figure 5C). Thus, the molecular mechanism underlying *peb* mutant motor neuron degeneration does not involve the axon death signaling pathway.

### Defects in α-tubulin acetylation induced by loss of *peb* function are not responsible for *peb* mutant NMJ degeneration

It was previously reported that loss of a nervous system-enriched Rreb1 transcript in mice is associated with decreased α-tubulin glutamylation and acetylation.^29^ Both of these posttranslational modifications are important for microtubule dynamics and axonal transport.^30^^,^^31^ Therefore, we investigated α-tubulin glutamylation and acetylation in homozygous *peb* mutant motor neuron clones in 21-day-old animals by quantifying immunostaining intensity. α-Tubulin glutamylation was significantly increased in *peb*^*Q894X*^ and *peb*^*E8*^ mutant motor neurons (Figures S5F and S5G), in contrast to decreased α-tubulin glutamylation in *Rreb1-nm3888*^−/−^ mice. However, we found a significant reduction in α-tubulin acetylation in both *peb*^*Q894X*^ and *peb*^*E8*^ homozygous mutant motor neuron clones in the VNC of 21-day-old flies (Figures S6A and S5B).

We, therefore, asked whether restoration of α-tubulin acetylation would be sufficient to rescue *peb* loss-of-function phenotypes. We treated flies with Tubastatin A, a frequently used inhibitor of the α-tubulin deacetylase HDAC6 (histone deacetylase 6),^32^ from the adult stage onward. As expected, Tubastatin A treatment rescued the decrease in α-tubulin acetylation in *peb* homozygous mutant motor neurons at 21 days of age (Figures 5D and S6C). However, *peb* mutant NMJ phenotypes were not significantly rescued by Tubastatin A treatment (Figure 5E). Thus, rescuing the reduced α-tubulin acetylation induced by loss of *peb* function is not sufficient to rescue the axonal degenerative phenotypes.

### Autophagy defects induced by loss of *peb* function are not responsible for *peb* mutant motor deficits

Because *Rreb1-nm3888*^−/−^ mice also displayed decreased autophagy,^29^ we evaluated whether defective autophagy might contribute to the neurodegenerative phenotype induced by loss of *peb* function. Immunostaining for the *Drosophila* p62 ortholog Ref(2)P revealed an increased number of Ref(2)P-positive puncta in *peb* mutant motor neuron clones in 21-day-old flies (Figures S6D and S6E), indicating accumulation of ubiquitinated proteins. To determine which step of the autophagy pathway is affected, we used an autophagy flux sensor, which allows to discriminate autophagosomes from autolysosomes and is based on the LC3 ortholog Atg8a tagged with GFP and mCherry.^33^ In the VNC of 21-day-old flies with *peb* knockdown in motor neurons, this sensor revealed no changes in the total number of Atg8a-positive puncta (Figures S6F and S6G), and no difference in autophagic flux (Figure S6H). To determine whether initiation of autophagy is affected, we performed *ex vivo* Bafilomycin A1 treatment on VNCs of 21-day-old flies expressing the autophagy flux sensor with or without knockdown of *peb* in motor neurons (Figure 5F). Bafilomycin A1 inhibits V-ATPase-dependent acidification and prevents autophagosome-lysosome fusion.^34^ While the number of autophagosomes was not changed in vehicle-treated control versus *peb* knockdown motor neurons, *peb* knockdown resulted in significantly decreased accumulation of autophagosomes after Bafilomycin A1 treatment (Figure 5F). This suggests that loss of *peb* function impairs the initiation of autophagy, resulting in the accumulation of ubiquitinated proteins.

To dissect the possible contribution of decreased autophagy to *peb* mutant phenotypes, we treated flies with rapamycin, an mTORC1 inhibitor that increases autophagy.^35^ Rapamycin treatment fully rescued Ref(2)P accumulation in 21-day-old *peb* mutant motor neuron clones (Figure 5G). Nevertheless, neither the motor performance defect induced by *peb* knockdown in motor neurons nor the NMJ degeneration induced by loss of *peb* function was rescued by rapamycin treatment (Figures 5H and 5I). Together, these data indicate that rescuing the autophagy defect is not sufficient to rescue *peb* mutant motor neurodegeneration, suggesting that autophagy defects are not a primary contributor to *peb* mutant phenotypes.

### RAS/MAPK pathway overactivation results in adult-onset motor axonal and NMJ degeneration

The human Peb ortholog RREB1 was reported to negatively regulate the transcription of RAS/MAPK pathway target genes, such as *JUN*, *MYC*, *FGFR4*, *HRAS*, and *MEK2*, by binding to RAS-responsive elements (RREs) in their promoters.^5^ Consistently, *RREB1* haploinsufficiency causes a Noonan-like RASopathy in *Rreb1*^*+/−*^ mice and humans.^5^^,^^6^^,^^7^ While *Rreb1*^*+/−*^ mice display reduced body length, orbital hypertelorism, and cardiac hypertrophy, neurodegenerative phenotypes have thus far not been reported in these mice.^5^ Because RREB1 can functionally replace Peb,^20^ they likely function in the same molecular pathways. Therefore, we evaluated whether excessive activation of the RAS/MAPK pathway could underlie axonal degeneration in *peb* mutants. Consistent with this hypothesis, RNAi-mediated knockdown of three known RAS/MAPK pathway suppressors in motor neurons—*Lztr1*, *Spred*, and *Nf1*—resulted in adult-onset NMJ degeneration (Figures 6A–6C). Furthermore, expression of a constitutively active form of RAS (Ras^R68Q^) induced a similar phenotype (Figures 6A–6C). Moreover, flies heterozygous for a loss-of-function allele of *Nf1*,^36^ a GAP for RAS that is associated with NF1, displayed an adult-onset climbing defect at 21 days of age (Figure 6D).

**Figure 6:**
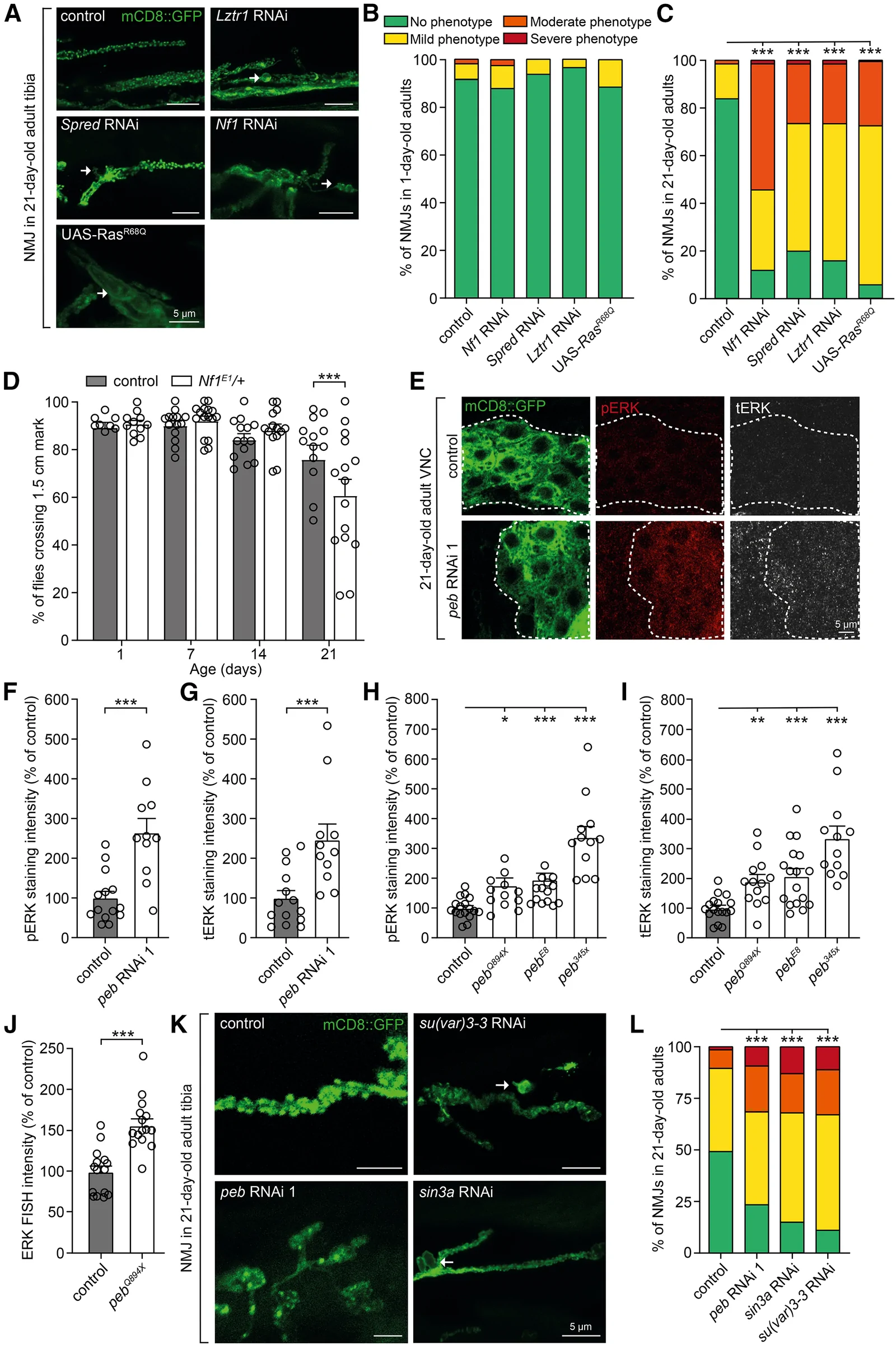
Overactivation of the RAS/MAPK pathway triggers adult-onset NMJ degeneration (A–C) (A) Representative images and (B and C) semi-quantitative analysis of NMJ morphology in the tibia of (B) 1-day-old or (A and C) 21-day-old flies upon knockdown in motor neurons (*OK371-GAL4*) of negative regulators of the RAS/MAPK pathway (*Lztr1*, *Spred*, and *Nf1*) or overexpression of constitutively active Ras (Ras^R68Q^). Arrows indicate NMJ swellings that are typically observed. Control is driver-only in GD genetic background. (D) Negative geotaxis motor performance (% of flies crossing a 1.5 cm mark) of heterozygous *Nf1*^*E1/+*^ flies as compared to genetic background control at 1, 7, 14, and 21 days of age. (E–I) (E) Representative images and (F–I) quantification of (E, F, and H) phospho-ERK (pERK) and (E, G, and I) total-ERK (tERK) immunostaining intensity in motor neuron cell bodies in the adult VNC at 21 days of age, (E–G) in the presence or absence of *peb* knockdown in motor neurons (*OK371-GAL4*) or (H and I) in *peb* mutant motor neuron clones. Data are normalized to control (E–G, driver-only, KK background and H and I, FRT19A). (J) Quantification of signal intensity of FISH for rl (rolled, the *Drosophila* ERK ortholog) transcript in the cell body of *peb*^*Q894X*^ mutant motor neuron clones in the adult VNC at 21 days of age. Data are shown as percentage of control (FRT19A). (K and L) (K) Representative images and (L) semi-quantitative analysis of NMJ morphology in the tibia of 21-day-old flies after knockdown of *peb*, *su(var)3-3*, or *sin3a* in motor neurons (*OK371-GAL4*). Control is driver-only in KK genetic background. ∗*p* < 0.05, ∗∗*p* < 0.01, ∗∗∗*p* < 0.005 by (B, C, and L) Fisher’s exact test with Bonferroni correction, (D) two-way ANOVA with Šidák’s multiple comparisons test, (F) unpaired *t* test with Welch’s correction, (G) Mann-Whitney test, (H and I) Brown-Forsythe and Welch’s ANOVA with Dunnett’s T3 test for multiple comparisons, and (J) unpaired *t* test. *n* = (B) 99–162 NMJs in 6–10 animals per genotype, (C) 88–131 NMJs in 6–10 animals per genotype, (D) 8–16 groups of 10 flies per genotype, (F and G) 11–14 animals per genotype, (H and I) 12–17 animals per genotype, (J) 15 animals per genotype, and (L) 139–204 NMJs of 6–10 animals per genotype. Data are represented as mean ± SEM. Scale bars: 5 μm in (A), (E), and (K).

To biochemically confirm RAS/MAPK pathway overactivation upon loss of *peb* function, we used phospho-ERK (pERK) and total-ERK (tERK) antibodies^37^ to quantify the levels of the ERK ortholog Rolled in motor neuron cell bodies in 21-day-old adult VNCs after *peb* knockdown or in *peb* mutant clones (Figures 6E–6I). We found a significantly increased staining intensity of both pERK (Figures 6E, 6F, and 6H) and tERK (Figures 6E–6G and 6I) following *peb* knockdown and in all three *peb* mutants. The increased tERK levels induced by loss of *peb* function suggest that Peb negatively regulates *rolled* transcription. Consistently, *rolled* mRNA levels are increased in the cell bodies of 21-day-old *peb*^*Q894X*^ motor neuron clones (Figure 6J). Taken together, our data show that RAS/MAPK pathway overactivation, in *peb* mutant conditions and otherwise, results in adult-onset motor neurodegeneration.

After binding to RREs, RREB1 recruits SIN3A (SIN3 transcription regulator family member A) and KDM1A (lysine demethylase 1A) to regulate histone H3K4 methylation at RAS/MAPK target gene promoters and reduce their expression.^5^ We, therefore, evaluated the effects of the knockdown of *sin3a* and *su(var)3-3*, the *Drosophila* orthologs of *SIN3A* and *KDM1A*, respectively. Similar to *peb* loss-of-function, knockdown of either *sin3a* or *su(var)3-3* in motor neurons induced an NMJ degenerative phenotype in 21-day-old adults (Figures 6K and 6L). These data indicate that in adult motor neurons, Peb likely functions in a complex with Sin3a and Su(var)3-3 to negatively regulate the transcription of RAS/MAPK pathway target genes.

### Pharmacological or genetic inhibition of the RAS/MAPK pathway rescues *peb* and *Nf1* loss-of-function phenotypes

To confirm that excessive activation of the RAS/MAPK pathway triggers adult-onset axonal degeneration in *peb* mutants, we evaluated the effect of pharmacological inhibition of the RAS/MAPK pathway using mirdametinib, an FDA-approved small molecule MEK1/2 inhibitor that is highly blood-brain-barrier penetrant.^38^^,^^39^ Treatment of adult flies with *peb* knockdown in motor neurons induced a dosage-dependent rescue of the motor performance deficit at 21 days of age, with 0.1 μM mirdametinib resulting in partial rescue and 1 μM mirdametinib inducing full rescue (Figure 7A). Furthermore, 1 μM mirdametinib fully rescued the NMJ degenerative phenotype of *peb* mutant motor neuron clones (Figures 7B and 7C). To confirm that the phenotypic rescue induced by mirdametinib treatment is attributable to inhibition of the *Drosophila* MEK1/2 ortholog *Dsor1*, we knocked down *Dsor1* in *peb* mutant motor neuron clones. As expected, *Dsor1* knockdown rescued NMJ degeneration induced by loss of *peb* function (Figure 7D). Importantly, NMJ degeneration in 21-day-old *Nf1*^*E1/+*^ heterozygous flies or flies with *Nf1* knockdown in motor neurons was also fully rescued by 1 μM mirdametinib (Figure 7E). Consistently, mirdametinib treatment rescued the increased levels of pERK in the VNC of 21-day-old *Nf1*^*E1/+*^ heterozygous flies (Figure 7F). Together, these data confirm that motor neurodegenerative phenotypes induced by loss of *peb* function or *Nf1* haploinsufficiency are attributable to overactivation of the RAS/MAPK pathway.

**Figure 7:**
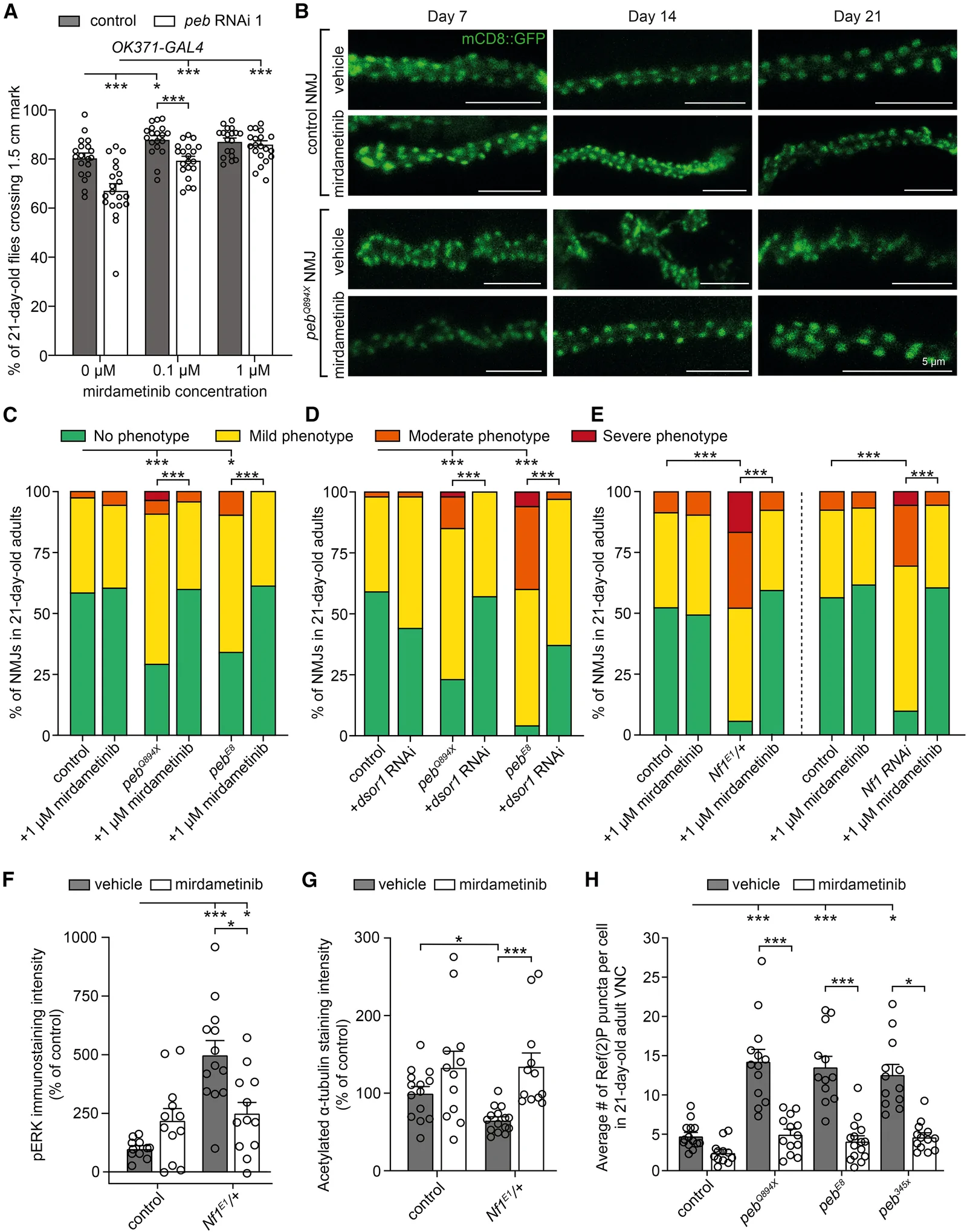
Pharmacological or genetic inhibition of the RAS/MAPK pathway rescues *peb* and *Nf1* loss-of-function phenotypes (A) Negative geotaxis motor performance of 21-day-old flies with *peb* knockdown in motor neurons (*OK371-GAL4*) versus driver-only control (KK-background), treated with vehicle or 0.1 μM or 1 μM mirdametinib. (B and C) (B) Representative images and (C) semi-quantitative analysis of the effect of 1 μM mirdametinib versus vehicle treatment on NMJ degeneration in *peb* mutant versus control (FRT19A) motor neuron clones in the femur of 21-day-old flies. (D) Semi-quantitative analysis of the effect of *Dsor1* knockdown on NMJ degeneration in *peb* mutant versus control (FRT19A) motor neuron clones in the femur of 21-day-old flies. (E) Semi-quantitative analysis of the effect of 1 μM mirdametinib treatment versus vehicle on NMJ degeneration in the tibia of 21-day-old heterozygous *Nf1*^*E1*^*/+* flies or flies with *Nf1* knockdown in motor neurons (*OK371-GAL4*) versus (driver-only) genetic background controls. (F) Quantification of phospho-ERK (pERK) immunostaining intensity in motor neuron cell bodies in the adult VNC of 21-day-old heterozygous *Nf1*^*E1/+*^ versus control (genetic background) flies, either treated with 1 μM mirdametinib or vehicle. Data are normalized to control. (G) Quantification of immunostaining intensity of acetylated α-tubulin in motor neurons of 21-day-old heterozygous *Nf1*^*E1/+*^ versus control (genetic background) flies, either treated with 1 μM mirdametinib or vehicle. Data were normalized to control. (H) Quantification of Ref(2)P-positive puncta in *peb* mutant versus control (FRT19A) motor neuron clones in the VNC of 21-day-old flies, either treated with 1 μM mirdametinib or vehicle. ∗*p* < 0.05, ∗∗∗*p* < 0.005 by (A, F, and G) Brown-Forsythe and Welch’s ANOVA with Dunnett’s T3 test for multiple comparisons, (C, D, and E) Fisher’s exact test with Bonferroni correction, or (G and H) Kruskal-Wallis test with Dunn’s multiple comparisons test. *n* = (A) 19–20 groups of 10 flies per experimental group, (C) 66–100 NMJs of 6–10 animals per experimental group, (D) 43–101 NMJs in 6–10 animals per experimental group, (E) 96–140 NMJs of 6–10 animals per experimental group, (F) 11–13 animals per experimental group, (G) 12–15 animals per experimental group, and (H) 12–15 animals per experimental group. Data are represented as mean ± SEM. Scale bars, 5 μm. See also Figure S7.

### Defects in α-tubulin acetylation and autophagy are downstream of RAS/MAPK pathway overactivation

The RAS/MAPK pathway has many downstream targets that may possibly be involved in neuronal maintenance, including HDAC6^40^ and mTOR.^41^ To determine whether the reduced α-tubulin acetylation levels and decreased autophagy are a consequence of RAS/MAPK pathway overactivation, we treated flies with mirdametinib or vehicle and evaluated acetylated α-tubulin levels and Ref(2)P-positive puncta at 21 days of age. Mirdametinib treatment restored acetylated α-tubulin levels in *peb*^*Q894X*^ mutant motor neuron clones (Figures S7A and S7B) and in heterozygous *Nf1*^*E1/+*^ animals (Figure 7G). Furthermore, increased Ref(2)P accumulation in *peb* mutant motor neuron clones was fully rescued by mirdametinib treatment (Figures 7H and S7C), thus demonstrating that the reduced α-tubulin acetylation levels and decreased initiation of autophagy are downstream of excessive RAS/MAPK signaling.

## Discussion

In this study, we performed a genetic screen to identify genes on the *Drosophila* X chromosome that are required for the maintenance of peripheral motor axons and NMJs. The deployed screening approach is unique because it utilizes microscopy imaging to identify neurodegenerative changes in motor and sensory neurons, their axons and NMJs in legs of 21-day-old flies. This phenotypic readout had not previously been used in *Drosophila* forward genetic screens. We identified mutations in 30 genes, of which 29 (97%) have human orthologs and 19 (66%) have previously been associated with neurological disorders, underscoring the disease-relevance of our screen. For these neurological disease-associated genes, the identified mutants may provide a *Drosophila* model for the respective diseases. For three identified genes (*Arp8*, *CG15890*, and *Tim9a*), no hemizygous lethal loss-of-function alleles were available until now.

Ten (34%) of the twenty nine identified genes with human orthologs have not yet been associated with neurological disease. Four of these genes (*Arp10*, *sno*, *Idh3a*, and *AMPKα*) have been linked to neurological phenotypes in animal models. For example, loss of function of the zebrafish *Arp10* ortholog *actr10* results in defective retrograde axonal transport of mitochondria and abnormal axon morphology,^42^^,^^43^ and cortical neuron-specific knockout of the *sno* ortholog *Sbno1* in mice resulted in hypotrophic axon bundles.^44^ Four other genes (*Arp8*, *Tim9a*, *sas10*, and *Hr4*) were reported to function in a pathway that was previously linked to neurodegeneration, but thus far, no neuronal phenotypes had been reported for these specific genes. For instance, the Arp8 ortholog ACTR8 has been shown to be a subunit of the INO80 chromatin remodeling complex, which plays an important role in DNA damage repair.^45^ Defective DNA damage repair has been extensively studied in the context of neurodegeneration,^46^ but ACTR8 loss-of-function has never before been directly linked to neurological phenotypes. Finally, for *Tsp2a*, a tetraspanin that localizes to smooth septate junctions in the *Drosophila* midgut,^47^ no neuronal functions had been described thus far. Thus, our genetic screen uncovered, for the first time, a neuronal function for five of the identified genes.

The fact that the majority of identified axonal degeneration genes have human orthologs associated with human neurodegenerative or neurodevelopmental disease suggests that the 10 identified genes that have not yet been linked to human neurological disease represent candidate disease genes. Indeed, while a fraction of recessively inherited or X-linked neurodegenerative and neurodevelopmental disease genes has been identified, more genes remain to be identified. Thus, the human orthologs of the identified genes may help prioritize candidate genes located in linked homozygous regions of human neurodegenerative and neurodevelopmental disease patients with a recessive inheritance pattern. Remarkably, even though our screening design was tailored to identify genes that cause neurodegeneration through a (partial) loss-of-function mechanism, 63% (22 out of 35) of the neurological disorders associated with the human orthologs of identified genes display an autosomal dominant inheritance pattern. This implies that the human orthologs of the 10 *Drosophila* genes that have hitherto not been associated with neurological disease may represent candidate genes for both recessive and dominantly inherited neurological disorders. Furthermore, these genes may constitute candidate (polygenic) risk genes for sporadic forms of human neurodegenerative diseases, e.g., in case they are located in a genomic region linked to a risk allele identified in genome-wide association studies (GWASs).^48^^,^^49^ Thus, the genes identified in our screen could help prioritize functional investigation of GWAS hits.

Remarkably, we identified mutations in 12 *Drosophila* genes with human orthologs associated with neurodevelopmental disorders that induce neurodegenerative phenotypes in *Drosophila*. On the one hand, altered neurodevelopmental processes might sensitize neurons to become more susceptible to degeneration with aging.^50^^,^^51^ On the other hand, proteins implicated in neurodegenerative disorders often play important roles in brain development, opening the possibility of shared pathogenic mechanisms.^52^ Indeed, for neurogenetic conditions associated with intellectual disability and autism spectrum disorders, emerging natural history data indicate that older adult patients may develop neurodegeneration. For instance, patients with Down syndrome (trisomy 21) develop neuropathological hallmarks of AD by 40 years of age, and subsequently often develop dementia.^50^ Currently, only a few of the genes identified in our screen that are associated with neurodevelopmental disorders have previously been associated with neurodegenerative or neuropathy phenotypes. One example is the *β-Spec* ortholog *SPTBN4*, which is associated with neurodevelopmental disorder with hypotonia, neuropathy, and deafness (NEDHND), a neurodevelopmental disorder with hypotonia, motor axonal neuropathy, and deafness.^53^^,^^54^ Our data suggest that other neurodevelopmental disorders may also be associated with neurodegeneration later in life. More research is needed to identify possible neurodegenerative features in individuals with neurodevelopmental disorders.

Taken together, our genetic screen provides important novel insight into the molecular mechanisms underlying axonal degeneration. Importantly, transgenic knockdown of identified axonal degeneration genes revealed that only a small minority of genes would have been identified in a transgenic RNAi screen. The most likely explanation is that RNAi-mediated knockdown is incomplete and induces only partial loss-of-function, whereas EMS-induced mutations may yield full loss-of-function alleles. This underscores the value of EMS-based genetic screens and illustrates the complementarity of EMS and RNAi-based genetic screens. We believe that it would be valuable to apply the same screening approach to the second and third chromosomes, which comprise about 80% of the *Drosophila* genome.

One of the identified axonal degeneration genes is *peb*, the *Drosophila* ortholog of human *RREB1*. We chose to focus on peb because it was one of only two genes identified in our screen that are associated with a neurodevelopmental disorder and for which loss of function resulted in adult-onset degeneration of motor axons and NMJs as well as motor performance deficits. These defects are mediated by excessive RAS/MAPK pathway activity. Peb expression is highly dynamic, and Peb mediates various functions depending on the specific cell type and life cycle stage.^55^^,^^56^ For example, Peb was reported to positively regulate Epidermal Growth Factor Receptor (EGFR) signaling and to downregulate JNK signaling in *Drosophila* embryos.^25^^,^^57^^,^^58^ In contrast, in adult motor neurons, we find that *peb* mutant phenotypes are not mediated by JNK-induced apoptosis. In addition, loss of *peb* function in adult motor neurons results in progressive degeneration that is unrelated to Wallerian degeneration, whereas in sensory neurons, *peb* loss-of-function does not induce degeneration (this study) and prevents Wallerian degeneration.^20^ The wide variety of Peb functions in different cell types may be due to Peb regulating different sets of target genes in different cell types and developmental stages, depending on chromatin accessibility and the protein complexes that Peb associates with. For instance, RREB1 has been shown to interact with several chromatin remodeling and histone modification complexes, such as HDAC1, HDAC2, KDM2A, and KDM3B.^5^

Our data suggest that overactivation of the RAS/MAPK pathway may be involved in neurodegenerative disease pathogenesis. Consistent with this notion, excessive RAS/MAPK pathway activation was previously implicated in *LRRK2*-associated PD. Indeed, in patient iPSC-derived dopaminergic neurons, the LRRK2 G2019S mutation was associated with increased ERK1/2 phosphorylation, and mirdametinib mitigated several PD-associated phenotypes in this cellular model.^14^ Furthermore, multiomic analysis suggested the MAPK pathway as an early disease mechanism in amyotrophic lateral sclerosis (ALS).^59^ However, our data are the first to show that increased RAS/MAPK signaling is sufficient to induce adult-onset neurodegeneration. Interestingly, loss of a nervous system-enriched *Rreb1* transcript (*V7* isoform) in mice induces progressive loss of cerebellar Purkinje cells,^29^ and in knock-in mouse models of Legius syndrome, a heterozygous P415A or P415V mutation in *Spred1* resulted in late-onset Purkinje cell degeneration leading to cerebellar ataxia.^60^ Our findings suggest that these phenotypes may be attributable to excessive activation of the RAS/MAPK pathway.

What may be the molecular mechanism downstream of excessive RAS/MAPK pathway activation that mediates axonal degeneration? Importantly, our data indicate that canonical degeneration pathways such as apoptosis and Wallerian degeneration are not detectably altered in *peb* mutant motor neurons, suggesting that the observed neurodegeneration proceeds through a distinct mechanism. Furthermore, we could show that autophagy is impaired and α-tubulin acetylation is reduced in *peb* mutant motor neurons as a consequence of RAS/MAPK pathway overactivation. However, pharmacological restoration of either pathway alone was insufficient to rescue NMJ degeneration, indicating that these defects are not the primary drivers of the phenotype. Instead, they likely represent secondary consequences of excessive RAS/MAPK pathway activation. One possibility is that simultaneous dysregulation of multiple downstream processes, including autophagy, microtubule stability, and potentially additional pathways, collectively contributes to axonal degeneration. This would explain why targeting a single pathway does not rescue. Notably, both autophagy and α-tubulin acetylation are known to be regulated, directly or indirectly, by RAS/MAPK signaling,^61^^,^^62^ reinforcing the idea that widespread signaling perturbations underlie the phenotype. Alternatively, axonal degeneration in *peb* mutants may be mediated by a distinct downstream mechanism of RAS/MAPK hyperactivation that is independent of both autophagy and α-tubulin acetylation. More work is needed to unravel the precise molecular mechanisms responsible for neurodegeneration downstream of RAS/MAPK pathway overactivation.

Our finding that loss of function of several negative regulators of the RAS/MAPK pathway induced motor axonal and NMJ degeneration suggests that neurodegenerative phenotypes may represent a currently underappreciated feature of RASopathies. In particular, our findings that heterozygous loss of *peb* or *Nf1* function is sufficient to induce age-dependent motor deficits in *Drosophila* suggest that haploinsufficiency of these genes—as is the case in *RREB1*-associated Noonan-like syndrome and NF1—is sufficient to induce axonal degeneration. Consistently, beyond cerebellar Purkinje cell degeneration in *RREB1*-mutant mice^29^ and mouse models of Legius syndrome,^60^ *Nf1*^*+/−*^ mice display motor deficits that are not attributable to cerebellar dysfunction.^63^

Interestingly, progressive neuropathic pain and muscle weakness associated with nerve enlargement were reported in children and adults with Noonan syndrome.^64^^,^^65^^,^^66^ Furthermore, a subset of NF1 patients displays neurofibromatous neuropathy, which is typically associated with large numbers of neurofibromas—the hallmark lesion of NF1—and disruption of the perineum.^67^^,^^68^ However, beyond tumor-dependent neuropathies, tumor-independent neuropathies affect ∼2% of NF1 patients.^69^ While the mechanism underlying neuropathy phenotypes in Noonan syndrome and related RASopathies remains unclear, our findings suggest that enhanced RAS/MAPK signaling in motor neurons may contribute to neuropathy phenotypes. In the future, this could be explored in clinical research and in RASopathy mouse models. Given that we could fully rescue all motor deficits by mirdametinib treatment in *Drosophila* RASopathy models, MEK1/2 inhibition might serve as a potential therapeutic approach, not only for Noonan syndrome but more broadly for patients with RASopathy, who suffer from neuropathies, and potentially also for patients suffering from neurodegenerative diseases.

### Limitations of the study

This study uses *Drosophila* as a genetic model organism to gain insight into the molecular mechanisms underlying axonal degeneration, with possible relevance for human neurodevelopmental and neurodegenerative diseases. In future studies, it is important to investigate whether overactivation of the RAS/MAPK pathway induces neurodegeneration in mammalian models (e.g., mouse models) and may causally contribute to human neurodegenerative diseases, and whether neurodegenerative features could be part of the phenotypic spectrum of RASopathies.

In addition, the primary genetic screen was restricted to females because the X-linked mutant lines were lethal in hemizygous males. Although males were included where possible (e.g., in RNAi knockdown experiments, see Table S5), sex-specific effects could not be assessed, which may limit the generalizability of these findings.

## Resource availability

### Lead contact

Further information and requests for resources and reagents should be directed to and will be fulfilled by the lead contact, Erik Storkebaum (erik.storkebaum@donders.ru.nl).

### Materials availability

*Drosophila* mutant lines generated in this study have been deposited to the Bloomington Drosophila Stock Center.

### Data and code availability

• Whole genome sequencing data have been deposited at ENA as ENA: PRJEB123363 and are publicly available as of the date of publication.
• All other data reported in this paper will be shared by the lead contact upon request.
• This paper does not report original code.
• Any additional information required to reanalyze the data reported in this paper is available from the lead contact upon reasonable request.

## Acknowledgments

We thank Marc Freeman, Lukas Neukomm, and Mikio Furuse for providing *Drosophila* lines; the General Instruments Facility (Faculty of Science, Radboud University) for advice on image acquisition and analysis; and Marijn Kuijpers for advice on Atg8a flux sensor analysis. We thank Youp van Oosterhout, Anna Bolhuis, Hanna Vreemann, Andrea Mlinar, Bram van Raalte, Yujin Hong, Vasilis Kougioumzoglou, Paulien Sloot, Nikki Smolders, Jimmy Vu, Kiki Peeters, Gino Hulshof, Dennis Colin, and Céline van Diermen for their help during their internships. M.B. and E.S. were supported by a grant from 10.13039/501100014076Stichting ALS Nederland; E.S. received an ERC Consolidator Grant (ERC-2017-COG 770244) and funding from the Radala Foundation, the “Prinses Beatrix Spierfonds” (W.OR22-03), the 10.13039/100005202Muscular Dystrophy Association (MDA 946876), AFM-Telethon, ARSLA, an NWO Open Competition ENW-M grant, an NWO Take-off WO Phase 1 grant, and a grant from the DI-NIN Fund.

## Author contributions

Conceptualization, M.B. and E.S.; methodology, M.B. and E.S.; software, M.B.; validation, M.B.; formal analysis, M.B., S.H., P.P., D.B., G.A., and K.K.; investigation, M.B., E.L., A. Serna, A.C.G.B., S.H., M.K., C.t.D.-M., P.P., H.R.A., B.v.R., S.G., P.v.L., and C.S.; resources, P.V., J.V., S.Z., C.G., A. Schenck, and E.S.; data curation, M.B.; writing – original draft, M.B.; writing – review and editing, M.B., D.B., P.V., J.V., S.Z., C.G., A.Schenck, and E.S.; visualization, M.B. and E.S.; supervision, A. Schenck and E.S.; funding acquisition, M.B. and E.S.

## Declaration of interests

The authors declare no competing interests.

## STAR★Methods

### Key resources table

**Table:** 

REAGENT or RESOURCE	SOURCE	IDENTIFIER
**Antibodies**		

Mouse monoclonal anti-Dlg1	DSHB	Cat#: 4F3; RRID: AB_528203
Mouse monoclonal anti-Brp	DSHB	Cat#: Nc82; RRID: AB_2314866
Mouse monoclonal anti-Syn	DSHB	Cat#: 3C11; RRID: AB_528479
Mouse monoclonal anti-Rop	DSHB	Cat#: 4F8; RRID: AB_1157869
Mouse monoclonal anti-Peb	DSHB	Cat#: 1G9; RRID:AB_528278
Rabbit polyclonal anti-Ref2P	Abcam	Cat#: Ab178440; RRID:AB_2938801
Mouse monoclonal anti-polyglutamylated alpha-tubulin, Clone GT355	Avantor	Cat#: USBI045161
Mouse monoclonal anti-acetylated alpha-tubulin, Clone 6-11B-1	Sigma Aldrich	Cat#: MABT868; RRID:AB_2819178
Mouse monoclonal anti-phospo-ERK	Sigma Aldrich	Cat#: M8159; RRID:AB_477245
Rabbit polyclonal anti-ERK	Cell Signaling	Cat#: 9102
Rabbit anti-GFP	Invitrogen	Cat#: A6455; RRID: AB_221570
Alexa Fluor goat anti-mouse-568	Invitrogen	Cat#: A11004; RRID: AB_2534072
Alexa Fluor Goat anti-rabbit-568	Invitrogen	Cat#: A11011; RRID: AB_143157
Alexa Fluor goat anti-rabbit-647	Invitrogen	Cat#: A21245; RRID: AB_2535813
Alexa Fluor Goat anti-mouse-647	Invitrogen	Cat#: A21235; RRID: AB_2535804
Alexa Fluor Goat anti-rabbit-488	Invitrogen	Cat#: A11008; RRID: AB_143165

**Chemicals, peptides, and recombinant proteins**		

Ethyl methanesulfonate	Sigma-Aldrich	M0880; CAS: 62-50-0
Sucrose	Sigma-Aldrich	S9378; CAS: 57-50-1
RNase A	Thermo Fisher	EN0531
Bovine Serum Albumin	Sigma-Aldrich	A9647
Formaldehyde solution	Sigma-Aldrich	252549
Bouin’s solution	Sigma-Aldrich	HT10132-1L
Goat serum	Gibco	16210064
Mirdametinib	MedChemExpress	HY-10254G
Rapamycin	MedChemExpress	HY-10219
Bafilomycin A1	MedChemExpress	HY-100558
Tubastatin-A	MedChemExpress	HY-13271A
Vectashield Antifade Mounting Medium	Vector Labs	H1000-10
Prolong Glass antifade Mountant	Thermo Fisher	P36980
Triton-X-100	Sigma-Aldrich	T8787; CAS:9036-19-5
Tween 20	Sigma-Aldrich	P1379 ; CAS: 9005-64-5
Dimethyl Sulfoxide (DMSO)	Sigma-Aldrich	D2438
Ultrapure SSC, 20X	Invitrogen	15557044
Sodium Citrate Dihydrate	Fisher Scientific	S279-500
100% ethanol	VWR Chemicals	20821.296; CAS:64-17-5
DNase RNase free 100% ethanol	Fisher Bioreagents	BP2818-500; CAS: 64-17-5
Liquid Proteinase K	Macherey-Nagel	740396
Nuclease-free water	Invitrogen	10977-035
Methanol	Fisher Chemical	M/4000/17; CAS:67-56-1
Jazz-Mix	Fisher Scientific	AS153

**Critical commercial assays**		

DNeasy Blood & Tissue kit	Qiagen	69504
Illumina Nextera Flex	Illumina	20018704
Illumina Nextera CD Indexes	Illumina	20018707
NextSeq 500/550 High Output Kit v2.5 (300 Cycles)	Illumina	20024908
In Situ Cell death detection kit, TMR red	Roche	12156792910
Qubit 1x dsDNA HS Assay Kit	Thermo Fisher	Q33231
Agilent DNA 12000 Kit	Agilent	5067-1508
Phusion Plus DNA polymerase	Thermo Fisher	F630L
FISH kit	Molecular instruments	N/A
GeneJET Gel Extraction and DNA cleanup Micro Kit	Thermo Scientific	K0832

**Deposited data**		

Whole genome sequencing	European Nucleotide Archive	ENA: PRJEB123363

**Experimental models: Organisms/strains**		

*Drosophila melanogaster*	See experimental model and study participant details and Table S4	N/A

**Oligonucleotides**		

Sanger sequencing primers	See Table S4	N/A

**Software and algorithms**		

GraphPad prism V10.2.1		N/A
Image J	Schindelin et al.^70^	https://imagej.net/software/fiji/
Deadeasy synapse	Sutcliffe et al.^71^	https://www.biosciences-labs.bham.ac.uk/hidalgo/Software.html
FastQC v0.11.5	Andrews^72^	https://www.bioinformatics.babraham.ac.uk/projects/fastqc/
Cutadapt v1.9.1	Martin^73^	https://cutadapt.readthedocs.io/en/stable/
Samtools v1.11	Li et al.^74^	https://www.htslib.org/
Picard v2.25.4	Broad Institute^75^	https://broadinstitute.github.io/picard/
BWA v0.712-r1039	Li^76^	https://github.com/lh3/bwa
Gatk v4.2.0.0	Poplin et al.^77^	https://gatk.broadinstitute.org/hc/en-us
SnpEff v5.0	Cingolani et al.^78^	https://pcingola.github.io/SnpEff/
SnpSift v5.0	Ruden et al.^79^	https://pcingola.github.io/SnpEff/#snpsift

### Experimental model and study participant details

Flies were maintained on standard fly food at 25°C unless otherwise described. Fly stocks were obtained from the Storkebaum lab collection, Schenck lab collection, Freeman lab collection, Furuse lab collection, Neukomm lab collection, Bloomington *Drosophila* Stock center (indicated with BDSC), Vienna *Drosophila* Resource Center (indicated with VDRC), or generated in this study (see below).

Storkebaum lab stocks:

*w*^*1118*^*, caz*^*2*^/FM7i, w∗, B; +/+; +/+.

*w[+]; OK371-GAL4*, UAS-mCD8::GFP, Tub-GAL80^ts^ ; +/+.

*w[+], OK371-GAL4;* +/+.

Freeman lab stocks:

FRT19A, tubGAL80; *OK371-GAL4*, UAS-mCD8::GFP; UAS-mCD8::GFP, ase-FLP/TM3, Sb, e.

*w[+], OK371-GAL4*, UAS-mCD8::GFP, UAS-mCD8::GFP; +/+.

*peb345x*, w∗, hsFLP, FRT19A/FM7i, B, w∗; +/+.

w∗; +/+ ; *peb*^*+tCH321-46J02*^ Attp2

Furuse lab stocks:

*y[2[, Tsp2a*^1^^,^^2^/FM7c

Neukomm lab stocks:

w; Sp/Cyo; dSarm[896], FRT2A, FRT82B/ TM3, Sb, e.

w ; Sp/CyO ; ase-FLP[3b]/TM3, Sbn,e.

w; UAS-dSarm(E)/Cyo; Dr/TM3, Sb, e.

w; UAS-NMN-D; +/+.

w; UAS-NMN-D^dead^ ; +/+.

Schenck lab stocks:

*w*^*1118*^*; +/+; Nf1*^*E1*^ (origin: Walker lab^36^).

*w*^*1118*^*; +/+; +/+ Nf1*^*E1*^ genetic background (origin: Walker lab^36^).

UAS-ras1^R68Q^ (origin: Steller lab^80^).

Bloomington stocks:

P{ry[+t7.2]=neoFRT}19A; ry[506] (BDSC_1709).

y[1] *peb[hnt-E8]/*FM7c (BDSC_3241).

y1 w1118; P{UASp-GFP-mCherry-Atg8a}2 (BDSC_37749).

P{ry[+t7.2]=hsFLP}1, P{w[+mC]=tubP-GAL80}LL1 w[∗] P{ry[+t7.2]=neoFRT}19A;

P{w[+mC]=UAS-mCD8::GFP.L}LL5/CyO; P{w[+mW.hs]=GawB}OK107 ey[OK107] (BDSC_44407).

y[1] w[∗] N[1]/FM7c, P{w[+mC]=GAL4-twi.G}108.4, P{UAS-2xEGFP}AX *OK371-GAL4* (BDSC_6873).

bsk[2] cn[1] bw[1] *speck[1]*/CyO, P{ry[+t7.2]=sevRas1.V12}FK1 (BDSC_5283).

w[∗]; P{w[+mC]=UAS-p35.H}BH2 (BDSC_5073).

VDRC stocks:

KK background (VDRC_60100).

GD background (VDRC_60000).

P{KK107575}VIE-260B (VDRC_101325).

w1118; P{GD1507}v3788 (VDRC_3788).

w1118; P{GD13892}v35877/TM3 (VDRC_35877).

w1118; P{GD4892}v13008 (VDRC_13008).

w1118; P{GD6885}v18024/TM3 (VDRC_18024).

P{KK100700}VIE-260B (VDRC_105852).

P{KK102965}VIE-260B (VDRC_106147).

P{KK102276}VIE-260B (VDRC_107276).

Table S1 lists mutants generated in this study. Duplication lines used for mapping, null alleles for complementation crosses, RNAi lines and other *Drosophila* genotypes are described in Table S4. Age and sex of flies (*Drosophila melanogaster*) used in this study, including experimental crosses, are described in Table S5.

### Method details

#### Isogenization and mutagenesis

Isogenized FRT19A/Y males were starved for 8 h before being treated with 10mM EMS (M0880, Sigma-Aldrich) in 1% sucrose (S9378, Sigma-Aldrich) solution on Whatmann paper for 16 h.^81^ After 8 h of recovery, EMS-treated males were crossed to *caz*^*2*^/Fm7i virgins. Parents were removed from the cross after 3 days. See Figure S1 for crossing scheme. Non-hemizygous lethal stocks were discarded. Saturation was calculated by S=p(N≥1)=1−e−(n/E∗λ), where S is the saturation.^81^ E equals the number of essential genes on the X chromosome, which is estimated to be around 690. n equals the number of genomes screened, thus 8892. λ can be calculated by the probability of receiving 0 lethal hits, P(N=0). This probability equals 0.84075 (8892 total stocks/ 7476 stocks that have not received a lethal hit). This results in a λ of 0.17. Thus, *S*=1-*e*- (8892/690 ∗ 0.17)=0.88.

#### *In vivo* leg imaging & screening

##### Initial MARCM screening

6 legs of at least two 21-day-old females were dissected and mounted in ProLong Glass antifade mountant (P36980, Thermo Scientific) and examined for motor axon and NMJ integrity under a fluorescence microscope (DM2500, Leica) as previously described.^82^ If a phenotype was observed, crosses were repeated to confirm the phenotype. If no GFP-labelled neurons were observed after three independent crosses, stocks were marked cell-lethal.

##### Confocal microscopy

For time course screening or phenotypic characterization of phenotypes in adult legs, 6 legs of at least 6 flies were examined and imaged on a Leica Sp8 Liachroic confocal microscope using a 63x objective. Axon images were taken from mid-femur or proximal tibia. NMJ images were taken from proximal femur or mid-tibia.

##### Semi-quantitative analysis

To quantify NMJ phenotypes, all legs of 6-10 flies were imaged by confocal microscopy (63x objective, 0.75x zoom). NMJs were scored as “no phenotype”, “mild phenotype”, “moderate phenotype” or “severe phenotype” based on the following criteria (representative images shown in Figure 3C):

**Table:** 

		Normal	Mild	Moderate	Severe
NMJ	Active zones	Aligned and consecutive	Partial loss of alignment	Completely misaligned	Not recognizable
		Round	Slightly distorted	Enlarged GFP punctae	
	Shape	Straight	Slightly deformed	Severely deformed, membrane swellings	Complete loss of structure
		Elongated		Reduced NMJ length	Almost fully degenerated

Approximately 100 NMJs per genotype were analyzed, collected from 5–6 animals. Statistical analysis was performed using a Fisher’s exact test on the pooled NMJ counts across genotypes. Investigators scoring the NMJ phenotypes were blinded for genotype.

#### Duplication and complementation mapping

All mutants that showed a motor axonal phenotype were subsequently crossed to the (1:Y) duplication stocks from the Bloomington Duplication Project^83^ (see Table S4 for all stocks used). Once rescued males were found, stocks were crossed to 1;3 linked P[acman] BAC duplications within the rescued regions. If no (1;Y) rescue was observed, stocks were crossed to 1;3 P[acman] BAC duplications covering regions that were not covered by (1;Y) duplications. All rescued stocks were crossed *inter se* with other mutants rescued by the same duplication to establish complementation groups. Additionally, rescued mutant males were crossed to all non-rescued and cell-lethal mutants for complementation mapping (see Figure S2).

#### Genome isolation

Genomes of heterozygous (FRT19A∗/FRT19A) and transheterozygous (FRT19A∗^1^/FRT19A∗^2^) mutants were isolated, together with the isogenized FRT19A. 50 virgins were collected and starved for 8 hours on Whatmann paper with water. Flies were flash-frozen and crushed using mortar and pestle. DNeasy Blood & Tissue Kit (69504, Qiagen) was used for subsequent genomic isolation. 180 μl of buffer ATL and 20 μl of ProtK was added to the crushed flies and incubated overnight at 56 °C. ProtK was heat-inactivated for 20 min at 75°C. 20 μL RNAse A (EN0531, Thermo Scientific) was added and samples were incubated for 2h at 37°C. 200 μl buffer AL and 200 μl DNAse-RNAse-free 100% ethanol (BP2818, Fisher Bioreagents) were then added and mixed. The mixture was pipetted with a cut pipet tip onto a spin column and centrifuged at 6000 x g for 1 min. 500 μl buffer AW1 was added before centrifuging again at 6000 x g for 1 min. 500 μl buffer AW2 was added and centrifuged at 20.000 x g for 3 min. The column was air-dried for several minutes. 50 μl of preheated nuclease free water (10977-035, Invitrogen) was added and incubated for 15 min at 70°C. DNA was eluted by centrifugation for 1 min at 6.000 x g. The elution step was repeated to obtain a higher DNA yield. Nanodrop, Qubit (Q33231, Thermo Fisher) and agarose gel were used to assess quality and quantity of DNA.

#### Library prep and whole-genome sequencing

All mutants that showed a motor axonal phenotype, as well as non-complementing cell-lethal mutants were used for whole-genome sequencing (See also Figure S2). Mutants were sequenced transheterozygously as described.^84^ Illumina Nextera Flex (20018704, Illumina) was used for library preparations together with Nextera CD Indexes (20018707, Illumina) according to manufacturer’s instructions. Library concentrations were quantified using Qubit and fragment size was estimated by Bioanalyzer (5067-1508, Agilent). Libraries were loaded on a 300 cycle High Output flowcell (20024908, Illumina) and sequenced on a NextSeq 500.

#### Whole genome sequencing data analysis

To assess the quality of Illumina sequencing reads, FastQC (version 0.11.5) was used.^72^ Cutadapt (version 1.9.1)^73^ was then used to trim adapter sequences wherever necessary. Alignment against the reference genome (*Drosophila melanogaster* release 6.32 plus ISO1 MT, Refseq: GCF_000001215.4) was performed using Burrows-Wheeler Aligner software (BWA version 0.7.12-r1039) using the BWA-MEM algorithm.^76^ Samtools (version 1.11)^74^ was used for sorting and indexing of the aligned reads. Duplicates were removed using Picard (version 2.25.4) MarkDuplicates. Genome Analysis Toolkit (GATK version 4.2.0.0) was used for base quality recalibration and subsequent variant calling using HaplotypeCaller.^77^ SnpEff (version 5.0)^78^ and SnpSift (version 5.0)^79^ were used for variant annotation and filtering respectively.

For rescued mutants, genomic coordinates of the 1;3 P[acman] duplications were used for filtering purposes. For non-rescued mutants, variants were filtered for nonsense single nucleotide variances (SNVs) or INDELS over the entire X-chromosome. Only heterozygous SNVs or INDELS with quality score >25 were considered. All identified SNVs were compared to the isogenized FRT19A genome and all other mutant genomes to remove any pre-existing, non-disease-causing variants. Human orthologs were identified through Ensembl,^85^ MARRVEL^86^ and Alliance.^87^

#### Phenotypic rescue

1;3 P[acman] duplication lines of all candidate disease-causing variants were introduced on the third chromosome of each mutant to determine 1) whether the lethality was rescued and 2) whether the axonal/NMJ degeneration phenotype was rescued (See Table S4 for the duplication lines used). To assess the phenotypic rescue, MARCM crosses were repeated and 21-day-old females carrying both the mutation and the duplication were screened for motor axonal and NMJ phenotypes using confocal microscopy.

#### Sanger sequencing confirmation

To confirm the presence of the disease-causing mutation in each mutant, sanger sequencing was performed on heterozygous mutant flies (FRT19A∗/FRT19A). Phusion Plus DNA polymerase (F630L, Thermo Fisher) was used to amplify each fragment (see Table S3 for oligonucleotide sequences) before PCR cleanup (GeneJET Gel Extraction and DNA cleanup Micro Kit, Thermo Scientific) and sanger sequencing.

#### Motor performance

To assess motor performance, a Rapid Iterative Negative Geotaxis (RING) assay was performed as described previously.^24^ This assay is based on the innate escape response of flies to climb up the wall of a vial after being tapped down to its bottom. Flies are transferred into test tubes without anesthesia and five or six iterative measurements are video recorded on a Nikon Z50 camera.

##### *peb* RNAi and *Nf1* mutant motor performance

For analysis of *peb* RNAi and *Nf1*^*E1/+*^ motor performance, *OK371-GAL4* virgins were collected and crossed to control, RNAi, or *Nf1*^*E1*^ males. From those crosses, males were collected and housed with 6-10 flies per vial. Because flies stopped climbing around 21 days of age, speed was not an accurate measure. Therefore, we manually counted the number of flies per vial that crossed a 1.5 cm mark within 9 seconds versus flies that did not cross. Per vial a percentage of flies crossing was calculated for three videos, and averaged to obtain one data point for the respective vial. Per timepoint, at least 8 vials were analyzed per genotype.

##### Motor performance to characterize heterozygous *peb* mutants

For characterization of dominant motor performance deficits, mutant FRT19A∗/FM7i virgins were crossed to FRT19A/Y males. FRT19A∗/FRT19A virgins were collected in groups of 10 per vial. 10 to 15 vials of 1-day-old and 21-day-old flies were tested per genotype. Analysis was performed as described above.

#### Lethal stage determination

For determination of the developmental stage of lethality, all mutant stocks were rebalanced with an *FM7c, twist-GAL4*>UAS-eGFP balancer (BL6873), that expresses eGFP in embryonic stages. 100-150 flies per genotype were kept O/N in a cage for egg-laying on an agar plate supplemented with yeast paste at 25 °C. The next day (day 2), ∼100 GFP-positive and ∼100 GFP-negative embryos were transferred to petri dishes containing Jazz-mix *Drosophila* medium (AS153, Fisher Scientific). On day 5, 2^nd^ instar larvae were counted and transferred to fresh medium, and on day 7 3^rd^ instar larvae were counted and transferred to regular food vials. Total number of pupae and eclosing adults were counted in the subsequent days. FRT19A was used as control. A developmental stage was marked lethal if <5% of animals survived the corresponding stage.

#### Fluorescent in situ hybridization (FISH)

Hybridization chain reaction (HCR) RNA-FISH (Molecular Instruments) was used to detect peb or *rl* and *VGlut1* mRNA. MARCM crosses were set with *peb* mutants, and non-balancer females were collected and aged for 21 days at 25 °C. A previously described protocol^88^ was adapted for *Drosophila* adult VNCs. Briefly, adult VNCs were dissected in PBS-T (PBS pH 7.2, 0.1% Tween 20 (P1370, Sigma Aldrich)), and fixed for 20 min in ice-cold 4% Formaldehyde (FA; 52549, Sigma-Aldrich) in PBS, followed by 20 min in ice-cold 4% FA in PBS-T. Three 10-minute washes with PBS-T were performed. Subsequently, VNCs were incubated for 3 min in 50% MeOH (M/4000/17, Fisher Chemical)/ 50% PBS-T, 3 min PBS-T, 3 min 100% MeOH, 3 min 50%MeOH / 50% PBS-T, and 3 min PBS-T. Another 20 min of fixing in 4% FA in PBS-T was performed before four 10-min washes in PBS-T. Prehybridization was performed by 14 minutes incubation in 4 μg/ml protK (740396, Macherey-Nagel). ProtK was washed away by rinsing twice with PBS-T, followed by two 5-minute PBS-T washes. A final 20 min 4% FA in PBS-T fixing step was performed before five 5-minute washes with PBS-T. VNCs were then incubated for 30 min in preheated probe hybridization buffer (Molecular Instruments) at 37 °C. Per sample, 1.6 pmol of each probe set (*peb* or *rl* and *VGlut1*) was added to 200μl of probe hybridization buffer and incubated O/N at 37 °C. The following day, four 15-minute washes with probe wash buffer were performed at 37 °C, followed by two 5-minute washes in SSC-T (5x sodium chloride sodium citrate (15557044, Invitrogen), 0.1% Tween-20) at RT. VNCs were then incubated with amplification buffer for 10 minutes. 6 pmol of hairpin H1 and 6pmol of hairpin H2 per sample were prepared separately by heating at 95 °C for 90s and cooled down for 30 minutes in the dark. Hairpins were added to 100 μl amplification buffer, and samples were incubated O/N in the dark. Hairpins were then removed by two 5-minute, two 30-minute and one 5-minute wash with SSCT. Samples were mounted in Vectashield (H1000-10, Vector Labs) and imaged on a Leica Sp8 Liachroic confocal microscope using a 63x objective. For quantification, the GFP channel was used to draw a circle around the plasma membrane in the plane where the nucleus diameter was the largest, and the mean intensity within this area was measured for each fluorescent channel for 4-10 cells per animal. *peb* or *rl* intensity was normalized to *VGlut1* intensity per cell. Then, the average normalized intensity of all cells per animal was used to obtain one datapoint. For *peb*, the normalized intensity of control animals without addition of the probe was averaged and used for background subtraction. Subsequently, data were normalized to FRT19A control.

#### Immunohistochemistry

##### Larval NMJ

*OK371-GAL4* (for anti-Dlg1) or *OK371-GAL4*, UAS-mCD8::GFP; UAS-mCD8::GFP (all other larval NMJ immunostainings) virgins were crossed to control or *peb* RNAi males. Third instar larvae were dissected in HL3 buffer (70 mM NaCl, 5mM KCl, 1.5 mM CaCl_2_, 20mM MgCl_2_, 10 mM NaHCO_3_, 5 mM trehalose, 115 mM sucrose, and 5 mM Hepes 5 (pH 7.2)), and fixed in Bouin’s (HT10132, Sigma-Aldrich) for 3 min (for anti-Dlg1 and anti-Brp) or fixed in 4% FA (52549, Sigma-Aldrich) in PBS, pH 7.2, for 30 min (for anti-Syn and anti-Rop). Three 15 min washes were performed in PBT (PBS pH7.2, 0.2% Triton-X-100 (T8787, Sigma-Aldrich)), before blocking in 10% goat serum (16210064, Gibco) in PBT for 1 h. Incubation with primary antibody – either anti-Dlg1 (mouse monoclonal 4F3; DSHB; 1:200), anti-Brp (mouse monoclonal nc82; DSHB; 1:200), anti-Synapsin (mouse monoclonal 3C11; DSHB; 1:200) or anti-Rop (mouse monoclonal 4F8; DSHB; 1:100) – was performed in blocking solution O/N at 4 °C. The following day, three 15-minute washes in PBT were followed by a 3 h incubation with secondary antibody, either Alexa Fluor goat anti-mouse-488 (A-11001; Thermo Fisher; 1:500) or Alexa Fluor goat anti-mouse-568 (A11004; Thermo Fisher; 1:500). Finally, samples were washed 3x 15 min and mounted in Vectashield (H1000-10, Vector Labs). Images were taken of muscle 8 in abdominal segment 5 using a Leica SP8 Liachroic confocal microscope with a 20x Plan-Apochromat objective.

For calculation of NMJ length, maximum intensity projections of z-stacks comprising the entire NMJ were used to measure synapse length in ImageJ. For quantification of active zones, ImageJ plugin ‘DeadEasy Synapse’^71^ was adjusted to work with two-channel confocal images as input and then used to calculate the volume occupied by Bruchpilot voxels in cubic microns.

##### Adult ventral nerve cord

21-day-old adult flies from either MARCM or RNAi crosses were put on ice, briefly submerged in 100% ethanol (20821.296, VWR chemicals) before VNCs were dissected in PBT (PBS pH 7.2, 0.2% Triton-X-100 (T8787, Sigma-Aldrich)). Samples were fixed on ice for 30 min in 4% FA (52549, Sigma-Aldrich) in PBS, pH 7.2. Three 15-minute washes were performed in PBT before blocking (PBT, 2% w/v BSA (A9647, Sigma Aldrich), 5% goat serum (16210064, Gibco)) for 2 h, followed by incubation in primary antibodies in blocking solution for ∼64 h at 4 °C. Samples were then washed 6 times in blocking solution for 20 min followed by a 2 h incubation with secondary antibodies in blocking solution. After another six 20 min washes, VNCs were mounted in Vectashield (H1000-10, Vector Labs) and imaged on a Leica Sp8 Liachroic confocal microscope using a 63x objective.

Primary antibodies used were: anti-Peb (Mouse monoclonal 1G9; DHSB; 1:5), anti-tERK (rabbit polyclonal 9102S; Cell Signaling; 1:50), anti-pERK (mouse monoclonal M8159; Sigma; 1:200), anti-polyglutamylated alpha-tubulin (mouse monoclonal GT335; VWR; 1:500), anti-acetylated alpha-tubulin (mouse monoclonal 6-11B-1; Sigma; 1:800) and anti-Ref2P antibody (rabbit polyclonal ab178440; Abcam; 1:400).

Secondary antibodies used are Alexa Fluor goat anti-mouse-647 (A21235; Invitrogen; 1:500), Alexa Fluor goat anti-mouse-568 (A11004, Invitrogen; 1:500), Alexa Fluro goat anti-rabbit-568 (A11011; Invitrogen; 1:500) and Alexa Fluor goat anti-rabbit-647 (A-21245; Invitrogen; 1:500).

For Peb immunostaining, background was subtracted based on a control without addition of the primary antibody. If the GFP signal in any of the immunostainings was variable, normalization against GFP was performed before normalization against controls.

For quantification of Ref(2)P (P62), the number of Ref(2)P positive puncta was counted in 6 cells per animal by going through all planes of that cell. This number was averaged per animal to obtain one datapoint.

For quantification of fluorescence intensity for all other antibodies, the GFP channel was used to draw a circle around the plasma membrane in the plane where the nucleus diameter was the largest, and the mean intensity within this area was measured for each fluorescent channel for 4-10 cells per animal. Average intensity of all measured cells per animal was used to obtain one datapoint.

#### TUNEL

For the TUNEL (Terminal deoxynucleotidyl transferase-mediated dUTP Nick End Labeling) assay, 21-day-old adults from MARCM crosses were put on ice, briefly submerged in ethanol before VNCs were dissected in PBT (PBS pH 7.2, 0.2% Triton X-100 (T8787, Sigma-Aldrich)). The protocol was adapted from Denton et al.^89^ Briefly, samples were fixed at RT for 20 min in 4% FA (252549, Sigma-Aldrich) in PBS, pH 7.2. Three 10-minute washes were performed in PBT followed by one wash in 0.5PBT (PBS pH 7.2, 0.5% Triton-X-100). Permeabilization was performed in 100 mM sodium citrate (S279, Fisher Scientific, freshly prepared, in PBT). Three 5-minute washes in PBT were performed before preparing the TUNEL (*In Situ* Cell Death Detection Kit, TMR red, Roche) reaction according to manufacturer’s instructions. Briefly, we used 10 μl of enzyme and 90 μl of labelling mix per sample, and incubated for 3 h at 37°C. Finally, three 10-minute washes were performed in PBT before mounting VNCs in Vectashield (H1000-10, Vector Labs). VNCs were imaged on a Leica Sp8 Liachroic confocal microscope using a 40x objective. Control flies which had received a 1.5 h heat shock at 37°C were included as a positive control for TUNEL staining. For quantification, the total number of homozygous mutant clones per animal and the number of TUNEL positive homozygous mutant clones were counted. The percentage of positive cells was calculated per animal to obtain one data point.

#### Autophagy flux sensor

A UAS-GFP-mCherry-Atg8a transgene was used as an autophagy flux sensor that is based on the LC3 ortholog Atg8a tagged with GFP and mCherry.^33^^,^^90^ This sensor allows to discriminate autophagosomes from autolysosomes, because GFP fluorescence is quenched in the acidic milieu of lysosomes, while mCherry fluorescence persists. Therefore, yellow punctae (positive for both GFP and mCherry) represent autophagosomes, whereas red punctae correspond to autolysosomes.

Control and *peb* RNAi virgins were crossed to *OK371-GAL4*, UAS-GFP-mCherry-Atg8a/CyO males. 21-day-old non-balancer males were anesthetized on ice and briefly submerged in ethanol before VNCs were dissected in PBT (PBS pH 7.2, 0.2% Triton-X-100 (T8787, Sigma-Aldrich)). Samples were fixed on ice for 30 min in 4% FA (252549, Sigma-Aldrich) in PBS, pH 7.2. Three 10-minute washes were performed in PBS before mounting VNCs in Vectashield (H1000-10, Vector Labs). VNCs were imaged on a Leica Sp8 Liachroic confocal microscope using a 63x objective. Total number of GFP + mCherry (yellow, autophagosomes) and mCherry only (autolysosomes) puncta were counted in all planes for 6 cells per animal. The total number of Atg8a puncta was determined per cell and averaged per animal to obtain one data point. For calculation of autophagic flux, the total number of autolysosomes was divided by total number of autophagosomes per animal to obtain one data point.

For *ex vivo* treatment with Bafilomycin A1, VNCs were dissected as described above. Dissected VNCs were kept for 6 h at 25 °C in either HL3 buffer (70 mM NaCl, 5mM KCl, 1.5 mM CaCl_2_, 20mM MgCl_2_, 10 mM NaHCO_3_, 5 mM trehalose, 115 mM sucrose, and 5 mM Hepes 5 (pH 7.2)), or HL3 buffer containing 200 nM Bafilomycin A1 (HY-100558, MedChemExpress). After treatment, samples were fixed on ice for 30 min in 4% FA (252549, Sigma-Aldrich) in PBS, pH 7.2. Three 10-minute washes were performed in PBS before mounting VNCs in Vectashield (H1000-10, Vector Labs). VNCs were imaged on a Leica Sp8 Liachroic confocal microscope using a 63x objective. Quantification was performed as described above.

#### Mushroom body MARCM

Control (FRT19A) or *peb* mutant virgins were crossed to hs-FLP, tubP-GAL80, FRT19A; UAS-mCD8::GFP/CyO; *OK107-GAL4* males. Heat shock was performed for 1h at 37 °C 6 days after hatching. Female offspring was collected and aged for 21 days before brains were dissected in PBT (PBS, 0.2% Triton-X-100, pH7.2) and subsequentially fixed for 30 min in ice-cold 4% FA (252549, Sigma-Aldrich) in PBS, pH 7.2. Three 10-minute washes were performed in PBT, before blocking for 1h in 10% goat serum in PBT on a rocking platform. Samples were incubated with rabbit anti-GFP (Invitrogen; A6455; 1:200) in blocking buffer O/N at 4°C. Another three 10-minute washes were performed in PBT before incubation for 3h in goat anti-rabbit-488 secondary antibody (Invitrogen; A11008; 1:500). Finally, three 10-minute washes in PBS were performed, before mounting in Vectashield (H1000-10, Vector Labs). Mushroom body β-lobes were imaged on a Leica Sp8 Liachroic confocal microscope using a 63x objective.

#### Drug feeding experiments

*OK371-GAL4* virgins were crossed to control or *peb* RNAi males to obtain offspring for motor performance assays, while mutant virgins were crossed to FRT19A, Tub-GAL80*; OK371-GAL4*, UAS-mCD8::GFP; ase-FLP, UAS-mCD8::GFP males. Newly eclosed males (for RNAi crosses) or females (for MARCM crosses) were collected and 1-day-old flies were transferred to regular food or Jazz mix food (AS153, Fisher Scientific) prepared according to manufacturer’s instructions, containing either the drug-of-interest or an identical quantity of solvent (vehicle). Flies were weekly transferred to freshly prepared food.

For Mirdametinib treatment, mirdametinib (HY10254G, MedChemExpress) was dissolved in 100% ethanol (20821.296, VWR chemicals) and used at a final concentration of 0.1 or 1 μM. For Tubastatin A (HY-13271A, MedChemExpress) treatment, Tubastatin A was dissolved in DMSO (D2438, Sigma-Aldrich), and used in a final concentration of 10 μM. For Rapamycin (HY-10219, MedChemExpress) treatment, DMSO was used as a solvent and a final concentration of 50 μM was used.

#### Temporal peb RNAi expression

For expression of *peb* RNAi selectively during development, adulthood or both, *OK371-GAL4*, UAS-mCD8::GFP, tub-GAL80^ts^ virgins were crossed to *peb* RNAi 1 or control males. For developmental expression of the RNAi, crosses were set at 29°C. Newly eclosed male flies were transferred to 18°C to switch off UAS transgene expression. For selective expression in adult flies, crosses were set at 18°C and newly eclosed male flies were transferred to 29°C. For continuous expression, crosses were set at 29°C and newly eclosed male flies were kept at 29°C. For developmental expression of the RNAi, 19-day-old males were transferred to 29°C to allow for UAS-mCD8::GFP expression. Legs of 21-day-old males were dissected, mounted in Prolong Glass antifade mountant (P36980, Thermo Scientific) and imaged on a Leica Sp8 Liachroic confocal microscope using a 63x objective.

### Quantification and statistical analysis

All results of statistical analysis are presented as mean ± standard error of the mean. Differences were considered significant when p < 0.05. Statistical details of individual experiments are described in the figure legends. No statistical methods were used to pre-determine sample sizes. Whenever possible, data collection and analysis were performed by investigators blinded to the genotype of the experimental animals. Before analysis, a Robust regression and Outlier removal method was performed to detect and exclude outliers. Assumptions of normality were tested by Shapiro-Wilk, Anderson-Darling and Kolmogorov-Smirnov tests, whereas assumptions on homogeneous variances (heteroscedasticity) were tested by Brown-Forsythe and Barlett’s test. Statistical tests were only performed if all their assumptions were met, the statistical test used per panel is described in each figure legend. Statistical analysis was performed using GraphPad Prism v10.2.1.

For comparison of normally distributed data between two groups, a two-tailed unpaired t-test was performed. When normality assumptions were not met, a Mann-Whitney test was performed.

For comparisons of normally distributed data between more than two groups and one factor (genotype), One-Way ANOVA with Tukey test for multiple comparisons was performed. If normality assumptions were not met, Kruskal-Wallis with Dunn’s multiple comparisons test was used. If heteroscedasticity assumptions were not met, a Brown-Forsythe and Welch ANOVA with Dunnett’s T3 multiple comparisons test was used.

For comparisons between more than two groups with two factors (genotype and treatment), two-way ANOVA with Geisser-Greenhouse correction and Tukey multiple comparison test was used. If normality assumptions were not met, Kruskal-Wallis with Dunn’s multiple comparisons test was used. If heteroscedasticity assumptions were not met, a Brown-Forsythe and Welch ANOVA with Dunnett’s T3 multiple comparisons test was used. For longitudinal motor performance data, normality and heteroscedasticity assumptions were tested individually for each relevant comparison.

## Supplemental information

Document S1. Figures S1–S7 and supplemental references

Data S1. Representative images of time-line screening of motor axonal, NMJ, and sensory neuron degenerative phenotypes for all non-cell lethal mutant lines

Data S2. Original unedited images

Table S1. Mutants identified in the X chromosome genetic screen, related to Figure 1

Table S2. Human orthologs and disease associations of axonal degeneration genes identified in the genetic screen, related to Table 1

Table S3. Phenotypic characterization of mutants identified in the screen, related to Figure 2

Table S4. *Drosophila* stocks, primers for Sanger sequencing confirmation, related to STAR Methods

Table S5. Age and sex of animals per figure and experimental crosses used per figure, related to STAR Methods
