# Supplementary material for: Excessive activation of the RAS/MAPK pathway triggers adult-onset motor axonal degeneration
Source: iScience. 2026 Sep 21;29(10):117507. doi: 10.1016/j.isci.2026.117507 (PMC13631674; doi:10.1016/j.isci.2026.117507)
Supplement: Supplementary file 1 [file mmc1.pdf]

iScience, Volume *29*

Supplemental information

Excessive activation of the RAS/MAPK pathway triggers adult-onset motor axonal degeneration

Marije Been, Erwan Lambert, Ana Serna, Anouk C.G. Balvert, Simon Heisinger, Merli Kreshpani, Catherine te Dorsthorst-Maas, Paulina Paškevičiūtė, Henar Rodríguez Arias, Danique Beijer, Galuh Astuti, Kevin Kenna, Boyd van Reijmersdal, Sharon Gloudemans, Pascal van Lith, Céline Sijlmans, Patrik Verstreken, Jan Veldink, Stephan Züchner, Christian Gilissen, Annette Schenck, and Erik Storkebaum

SUPPLEMENTAL FIGURES AND LEGENDS

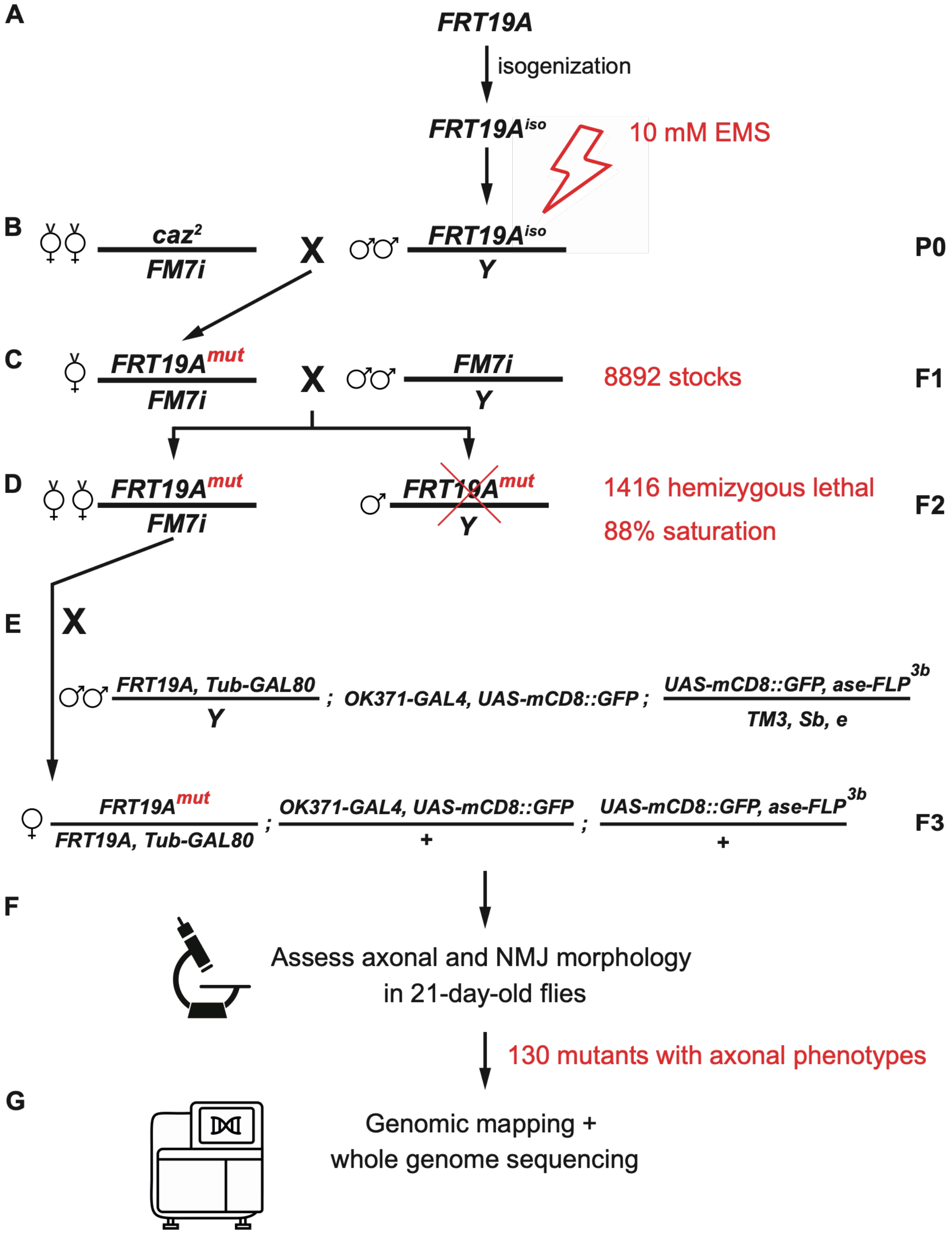

Figure S1. Schematic overview of the F3 adult mosaic genetic screen of the *Drosophila* X chromosome. Related to Figure 1.

(A) The FRT19A chromosome was isogenized. Male flies were mutagenized and **(B)** crossed to balancer virgins to obtain **(C)** FM7i balanced females with unique mutations. **(D)** Stable stocks were built and assessed for hemizygous lethality. 1416 X-linked recessive lethal stocks were identified and **(E)** crossed to MARCM males to **(F)** assess axonal and NMJ morphology in 21-day-old flies. **(G)** 130 mutants with motor axonal phenotypes were identified (including putative cell-lethal mutants in which no GFP-labeled axons or NMJs could be observed) and used for genomic mapping and whole genome sequencing to identify the causative gene. *caz*^{2}/*FM7i* virgins were used because the *caz*^{2} allele is homozygous lethal [S1].

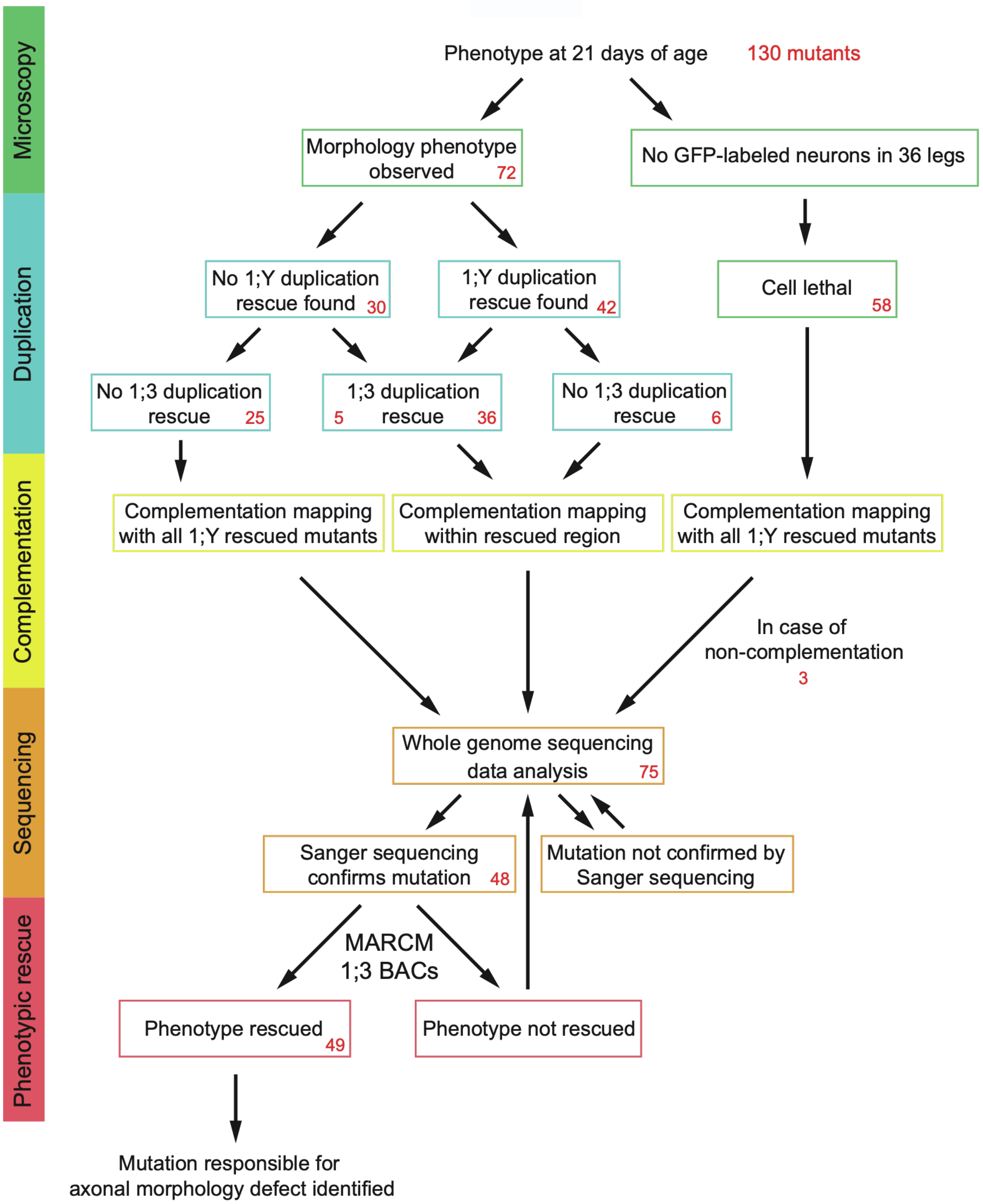

Figure S2. Flow chart showing the steps towards identification of the causative mutations in identified neurodegeneration mutants. Related to Figure 1.

The subsequent steps involving microscopy analysis, duplication mapping, complementation mapping, whole genome and Sanger sequencing, and phenotypic rescue experiments are shown. The number of mutants in each step is indicated in red.

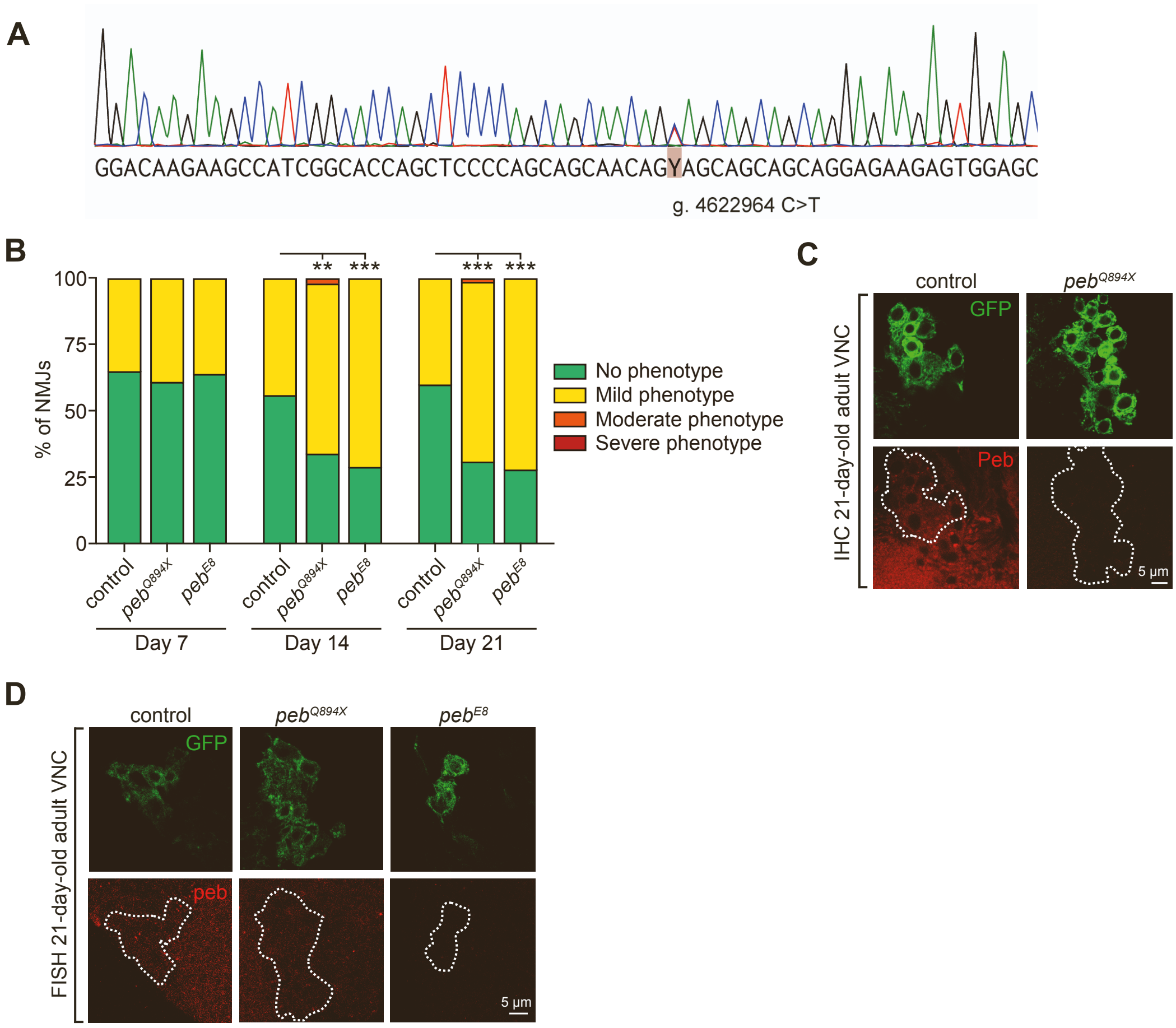

Figure S3. Characterization of *peb* loss-of-function alleles. Related to Figure 3.

(A) Sanger sequencing results to confirm the peb^{Q894X} heterozygous mutation. **(B)** Semi-quantitative analysis of NMJ degeneration in the femur of peb^{Q894X} and peb^{E8} mutants versus control (FRT19A) at 7, 14, and 21 days of age. **P<0.01, ***P<0.001 by Fisher's exact test with Bonferroni correction. n= 54-133 NMJs of 6-7 animals per genotype. **(C,D)** Representative images of (C) Peb immunohistochemistry and (D) FISH for peb transcript in motor neuron cell bodies in the VNC of 21-day-old adult flies. *OK371-GAL4*-driven GFP expression labels MARCM-induced homozygous mutant motor neuron clones. Scale bars: 5 µm.

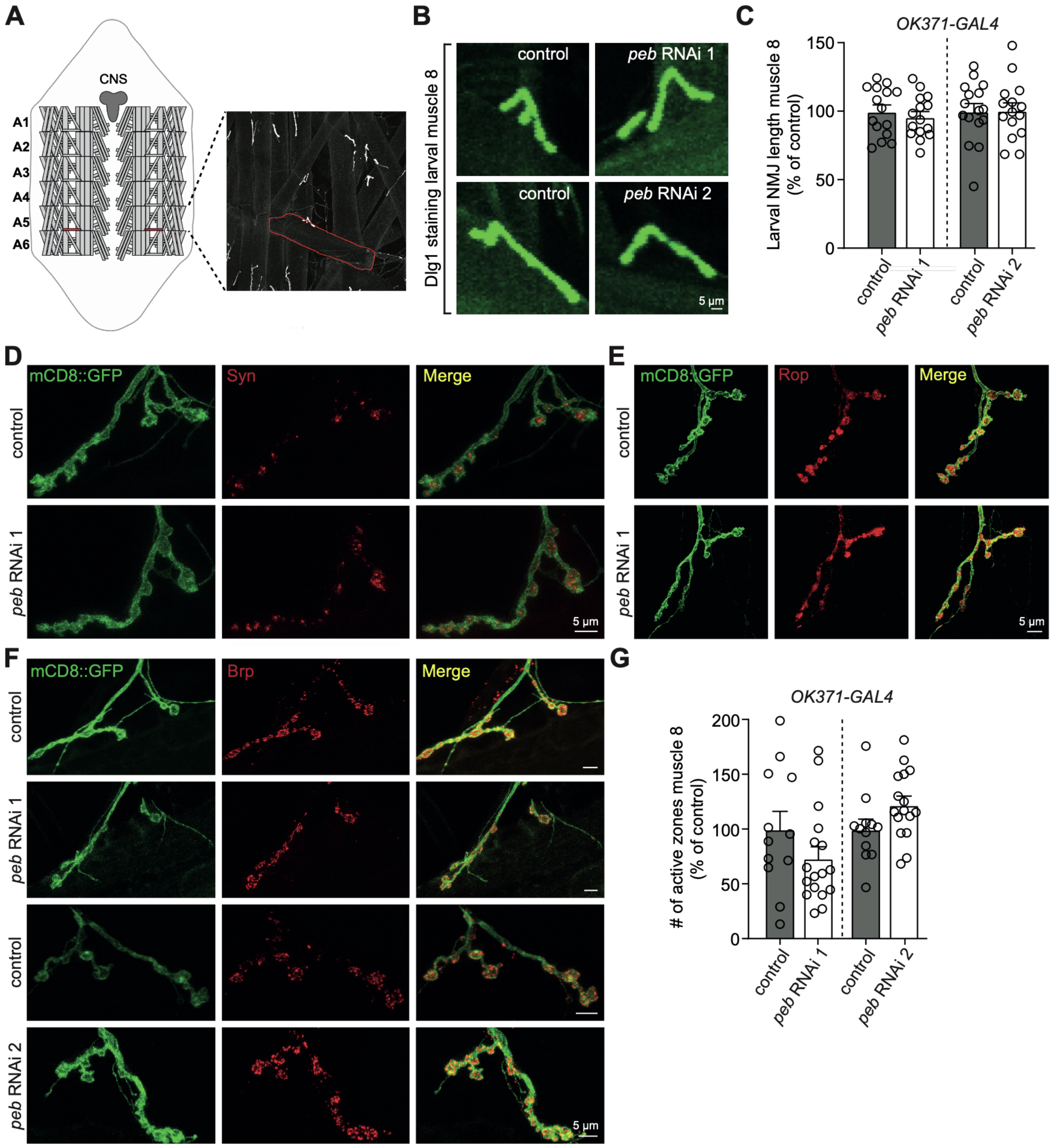

Figure S4. Loss of *peb* function results in an adult-onset phenotype. Related to Figure 4.

(A) Overview of the muscle architecture in the larval body wall. Imaging and quantification were performed in muscle 8 in abdominal segment 5 of third instar larvae (delineated in red). **(B)** Representative images and **(C)** quantification of NMJ length in larvae with *peb* knockdown in motor neurons (*OK371-GAL4*) using two independent RNAi lines versus genetic background controls (KK or GD background, respectively). NMJs were visualized using immunostaining for the postsynaptic marker Dlg1. **(D-F)** Representative images of immunostaining for presynaptic markers, including (D) synaptic vesicle protein Synapsin (Syn), (E) SNARE-binding protein Ras opposite (Rop) and (F) active zone marker Bruchpilot (Brp) on the NMJ on larval muscle 8, in the presence or absence of *peb* knockdown in motor neurons (*OK371-GAL4*). **(G)** Quantification of the number of active zones in the larval NMJ after *peb* knockdown in motor neurons (*OK371-GAL4*) using two independent RNAi lines versus genetic background controls (KK or GD background, respectively). P=not significant by (C,G) unpaired t-test. n= (C) 15-16 animals per genotype and (G) 12-17 animals per genotype. Data are represented as mean ± SEM. Scale bars: 5 µm.

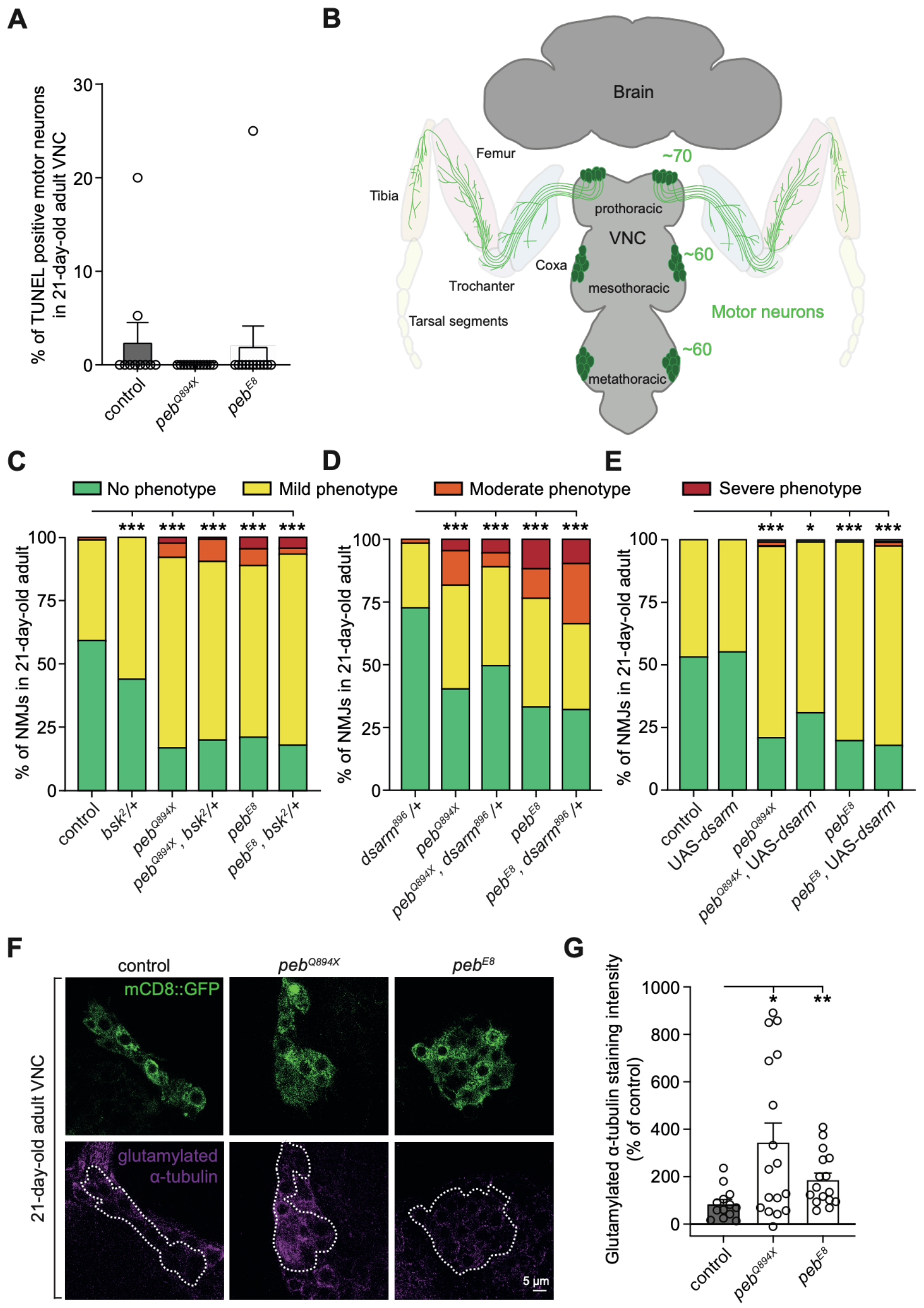

Figure S5. *peb* mutant phenotypes are independent of Wallerian degeneration, cell death pathways or reductions in glutamylated α-tubulin. Related to Figure 5.

(A) Quantification of the percentage of TUNEL positive nuclei in *peb* mutant versus control (FRT19A) motor neuron clones in 21-day-old adult VNCs. **(B)** Schematic overview of motor neuron clusters in the *Drosophila* ventral nerve cord (VNC). Each leg is innervated by 60-70 motor neurons [S2]. **(C)** Semi-quantitative analysis of femoral NMJ degeneration in control (FRT19A) and *peb* mutant motor neuron clones of 21-day-old animals in the presence or absence of a heterozygous loss-of-function mutation in the JNK ortholog *bsk.* **(D,E)** Semi-quantitative analysis of femoral NMJ degeneration in control (FRT19A) and *peb* mutant motor neuron clones in 21-day-old adult flies, in the presence or absence of (D) a heterozygous *dsarm* mutation, or (E) UAS-*dsarm*. **(F)** Representative images and **(G)** quantification of immunostaining intensity of glutamylated α-tubulin in cell bodies of *peb* mutant versus control (FRT19A) motor neuron clones in 21-day-old adult VNCs. Data are shown as percentage of control. Scale bar in (F): 5 µm. *$P<0.05$, **$P<0.01$, ***$P<0.005$ by (A) Kruskal-Wallis test with Dunn's multiple comparisons test, (C,D,E) Fisher's exact test with Bonferroni correction or (G) Brown-Forsythe and Welch ANOVA with Dunnett's T3 multiple comparisons test. n= (A) 10-12 animals per genotype, (C) 73-114 NMJs in 6-10 animals per genotype, (D) 38-141 NMJs in 6-10 animals per genotype, (E) 86-108 NMJs in 6-10 animals per genotype and (G) 13-17 animals per genotype. Data are represented as mean ± SEM.

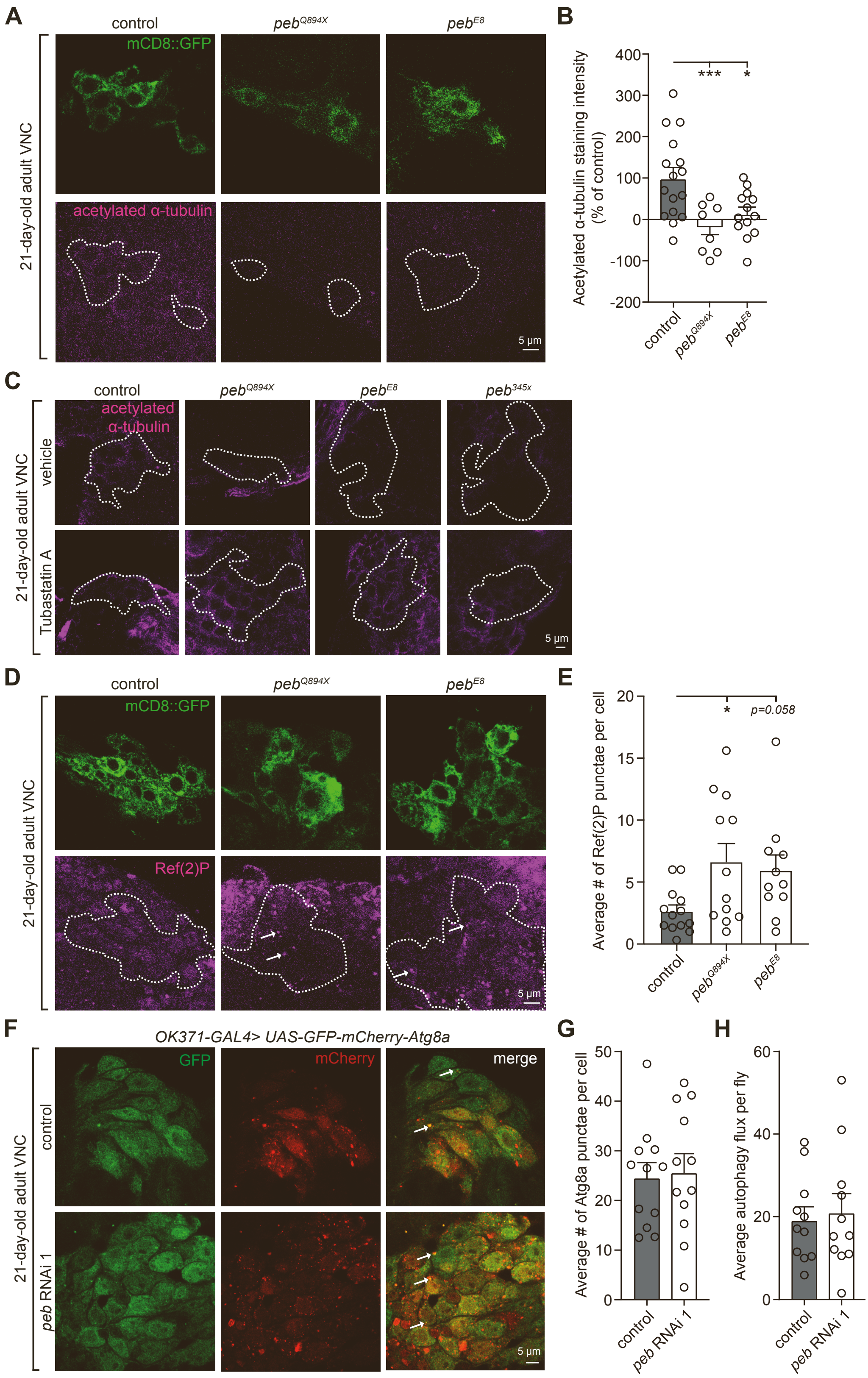

A
control
peb^Q894X
peb^E8
21-day-old adult VNC
mCD8::GFP
acetylated α-tubulin
5 µm
B
Acetylated α-tubulin staining intensity (% of control)
400
300
200
100
0
-100
-200
***
*
control
peb^Q894X
peb^E8
C
control
peb^Q894X
peb^E8
peb^345x
acetylated α-tubulin
vehicle
Tubastatin A
21-day-old adult VNC
5 µm
D
control
peb^Q894X
peb^E8
mCD8::GFP
Ref(2)P
21-day-old adult VNC
5 µm
E
Average # of Ref(2)P punctae per cell
20
15
10
5
0
*
p=0.058
control
peb^Q894X
peb^E8
F
OK371-GAL4> UAS-GFP-mCherry-Atg8a
GFP
mCherry
merge
control
peb RNAi 1
21-day-old adult VNC
5 µm
G
Average # of Atg8a punctae per cell
50
40
30
20
10
0
control
peb RNAi 1
H
Average autophagy flux per fly
60
40
20
0
control
peb RNAi 1

Figure S6. Loss of *peb* function results in defects in α-tubulin acetylation and autophagy. Related to Figure 5.

(A) Representative images and **(B)** quantification of immunostaining intensity of acetylated α-tubulin in control (FRT19A) versus *peb* mutant motor neuron clones in the VNC of 21-day-old flies. Data was normalized to control without primary antibody, and shown as percentage of control (FRT19A). **(C)** Representative images of immunostaining intensity of acetylated α-tubulin in control (FRT19A) versus *peb* mutant motor neuron clones in the VNC of 21-day-old flies treated with 10 μM Tubastatin A or vehicle. **(D)** Representative images and **(E)** quantification of Ref(2)P-positive punctae (indicated by arrows in D) in *peb* mutant versus control (FRT19A) motor neuron clones in the VNC of 21-day-old flies. **(F)** Representative images and **(G)** quantification of the average number of mCherry-positive Atg8a puncta per motor neuron (*OK371-GAL4*) in the ventral nerve cord of 21-day old flies expressing the GFP-mCherry-Atg8a autophagy marker, with or without simultaneous *peb* knockdown. **(H)** Quantification of autophagic flux, calculated by dividing the total number of red puncta (autolysosomes) per animal by the total number of yellow puncta (autophagosomes) per animal. *P<0.05, ***P<0.005 by (B) one-way ANOVA with Dunnett's multiple comparisons test or (E) Brown-Forsythe and Welch ANOVA with Dunnett's T3 multiple comparisons test or (G,H) two-tailed unpaired t-test. n= (B) 8-16 animals per genotype, (E) 11-13 animals per genotype and (G,H) 11-12 animals per genotype. Data are represented as mean ± SEM. Scale bars: 5 μm.

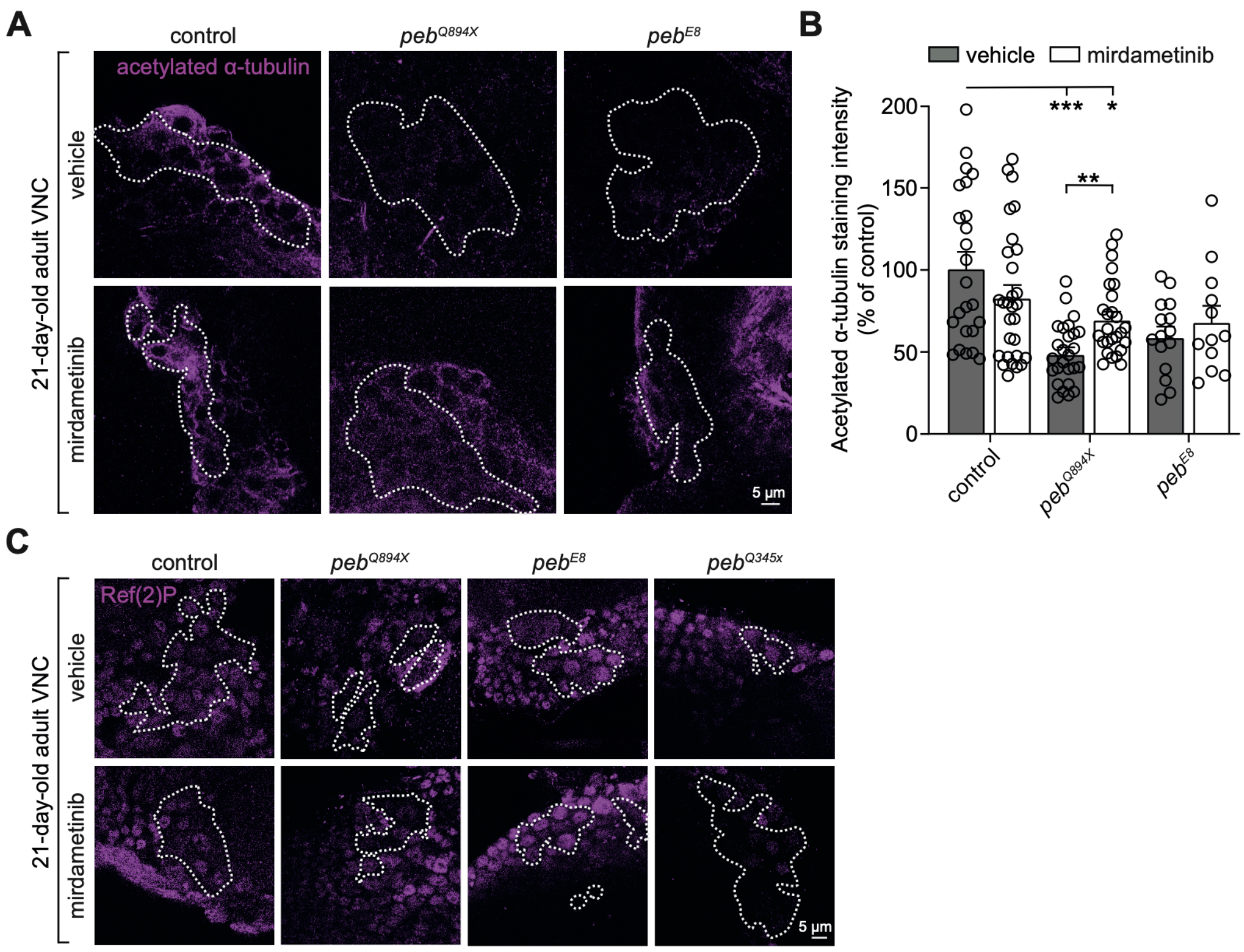

Figure S7. Defects in α-tubulin acetylation and autophagy are downstream of excessive RAS/MAPK signaling. Related to Figure 7.

(A) Representative images and **(B)** quantification of immunostaining intensity of acetylated α-tubulin in control (FRT19A) versus *peb* mutant motor neuron clones in the VNC in 21-day-old flies treated with 1μM mirdametinib or vehicle. Data were normalized to control. **(C)** Representative images of Ref(2)P-positive puncta in *peb* mutant versus control (FRT19A) motor neuron clones in the VNC of 21-day-old flies, treated with 1μM mirdametinib or vehicle. *P<0.05, **P<0.01, ***P<0.005 by (B) Brown-Forsythe and Welch ANOVA with Dunnett's T3 multiple comparisons test. n= (B) 12-29 animals per experimental group. Data are represented as mean ± SEM. Scale bars: 5 μm.

SUPPLEMENTAL REFERENCES

S1: Frickenhaus, M., Wagner, M., Mallik, M., Catinozzi, M., and Storkebaum, E. (2015). Highly efficient cell-type-specific gene inactivation reveals a key function for the Drosophila FUS homolog cabeza in neurons. Sci Rep *5*, 9107. https://doi.org/10.1038/srep09107.

S2. Guan, W., Venkatasubramanian, L., Baek, M., Mann, R.S., and Enriquez, J. (2018). Visualize Drosophila Leg Motor Neuron Axons Through the Adult Cuticle. J Vis Exp. https://doi.org/10.3791/58365.
