# Supplementary material for: Excessive activation of the RAS/MAPK pathway triggers adult-onset motor axonal degeneration
Source: iScience. 2026 Sep 21;29(10):117507. doi: 10.1016/j.isci.2026.117507 (PMC13631674; doi:10.1016/j.isci.2026.117507)
Supplement: Supplementary file 2 [file mmc2.pdf]

control

Data S1: Representative images of time-line screening of motor axonal , NMJ, and sensory neuron degenerative phenotypes for all non-cell lethal mutant lines.

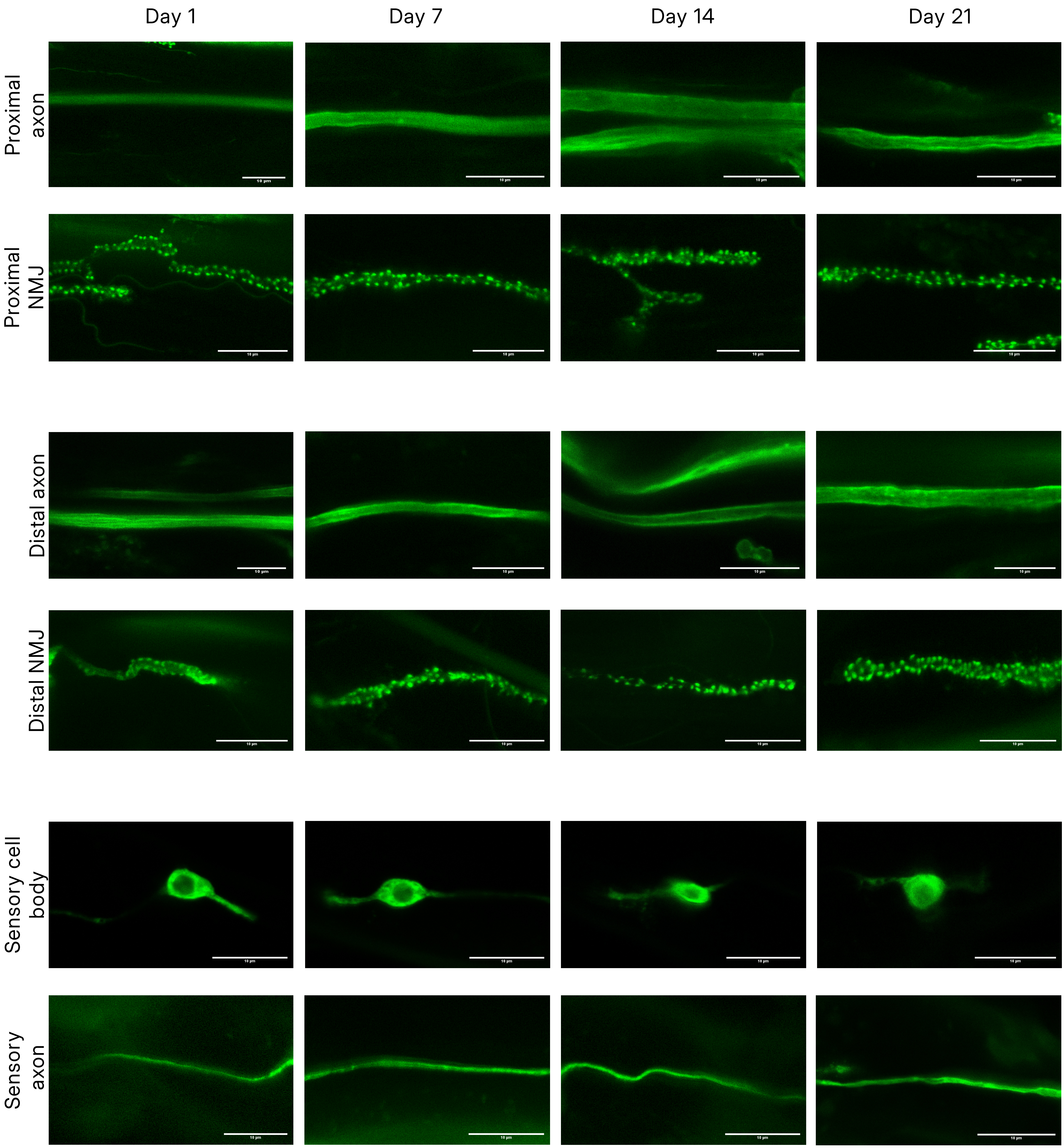

Scale bars: 10 µm

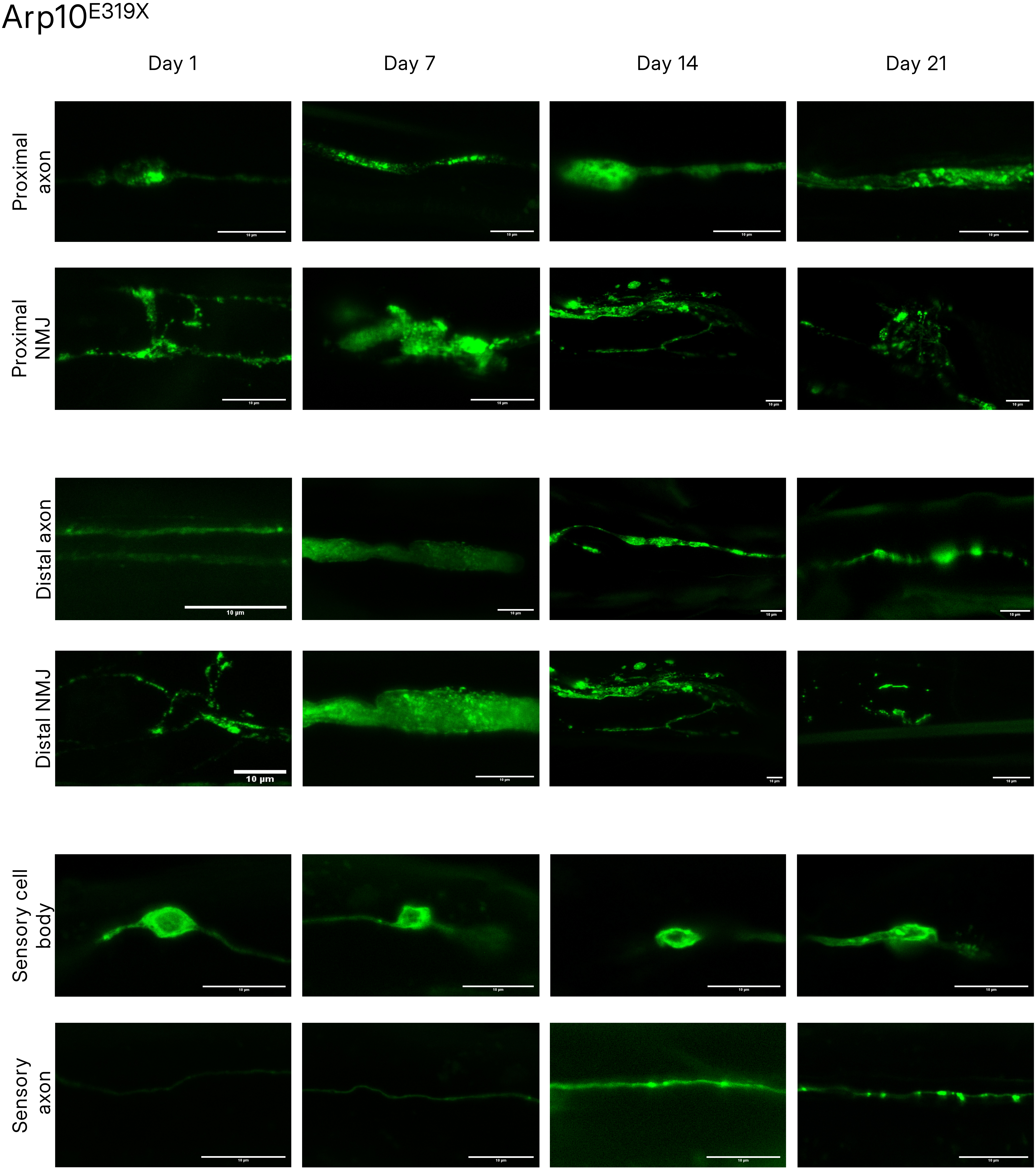

Arp10E319X
Day 1
Day 7
Day 14
Day 21
Proximal axon
Proximal NMJ
Distal axon
Distal NMJ
Sensory cell body
Sensory axon
10 µm

Arp10^{F321X}

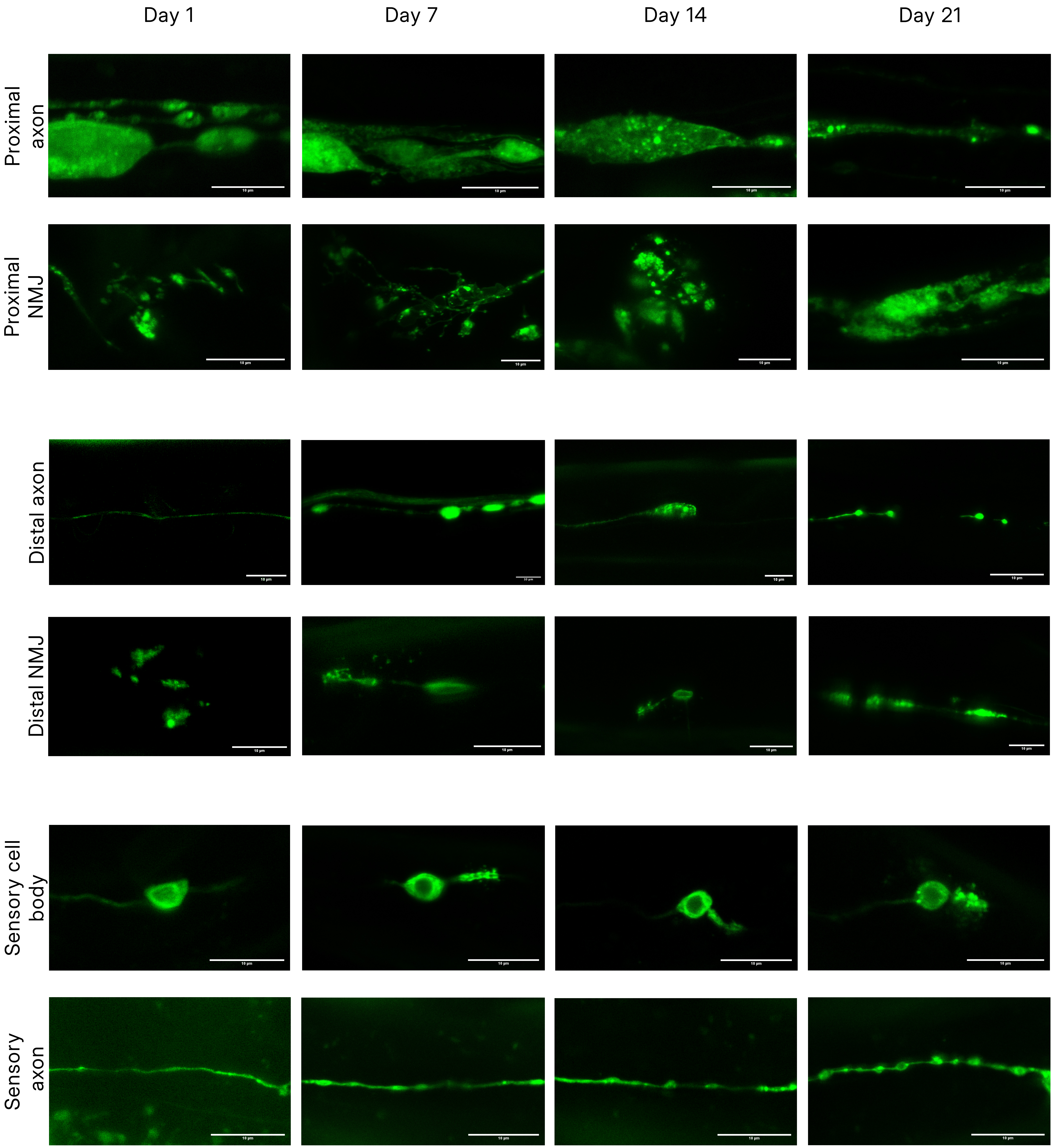

Day 1
Day 7
Day 14
Day 21
Proximal axon
Proximal NMJ
Distal axon
Distal NMJ
Sensory cell body
Sensory axon

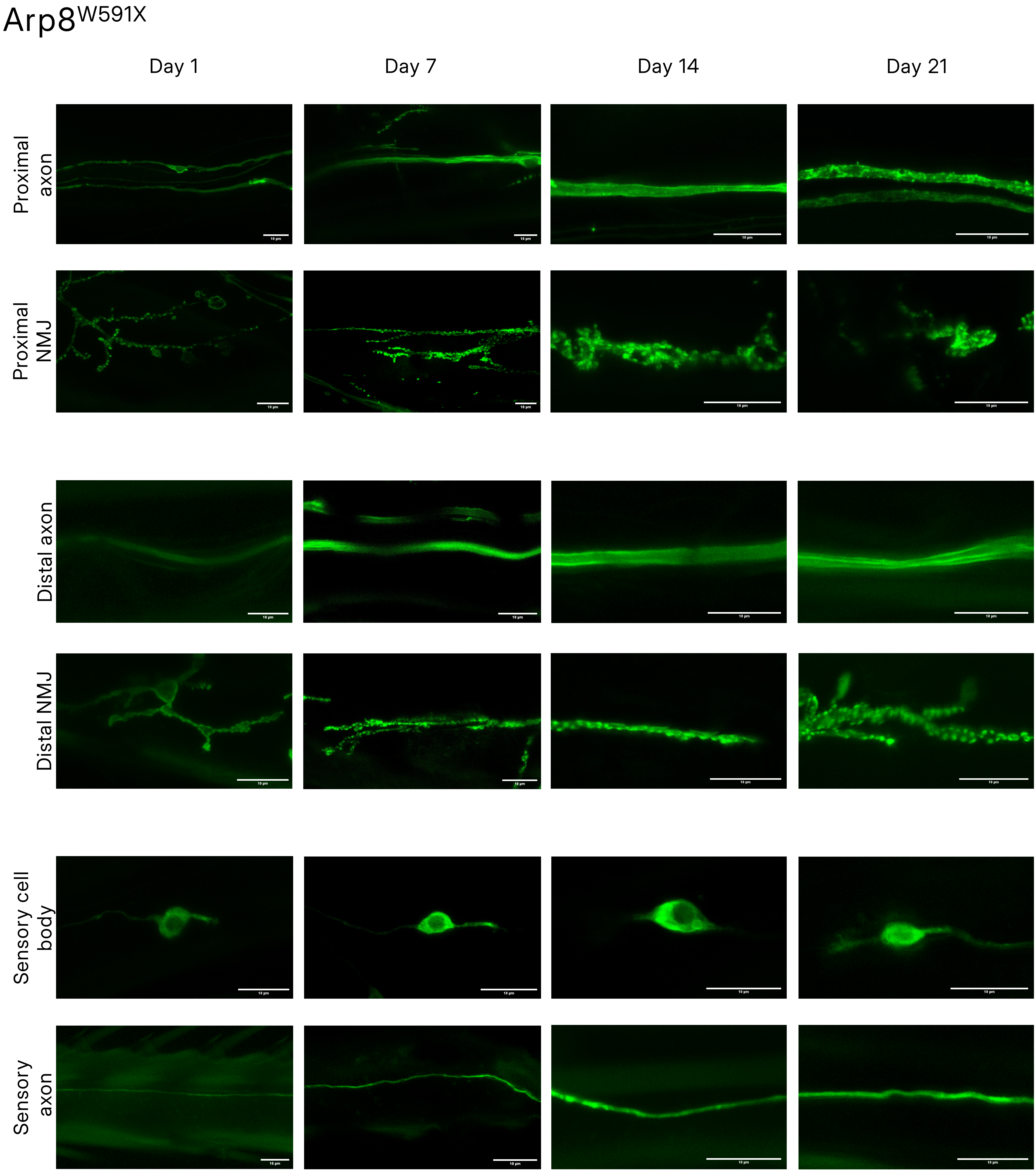

Arp8^{W591X}
Day 1
Day 7
Day 14
Day 21
Proximal axon
Proximal NMJ
Distal axon
Distal NMJ
Sensory cell body
Sensory axon

AP-1-2-β^{W528X}

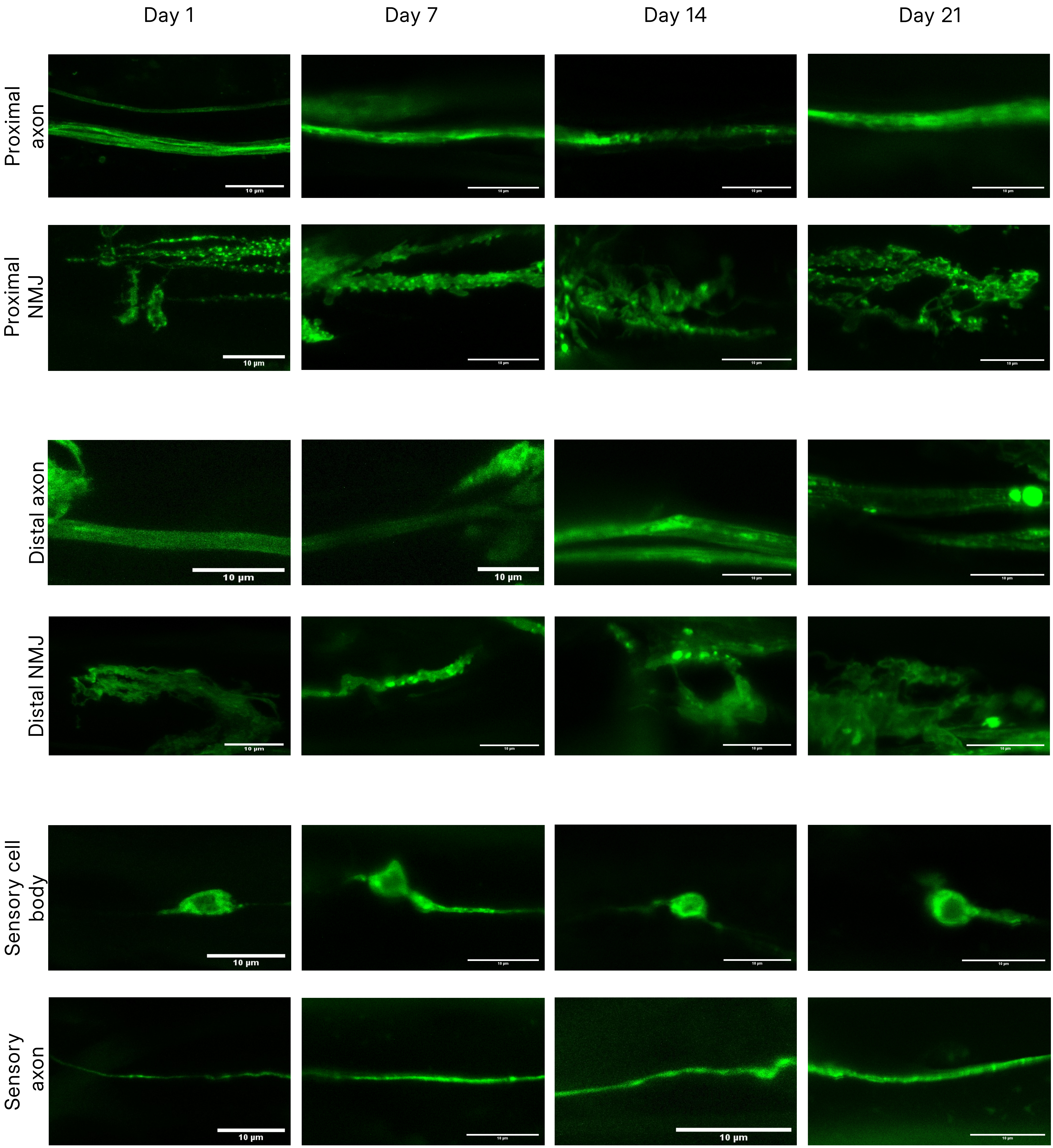

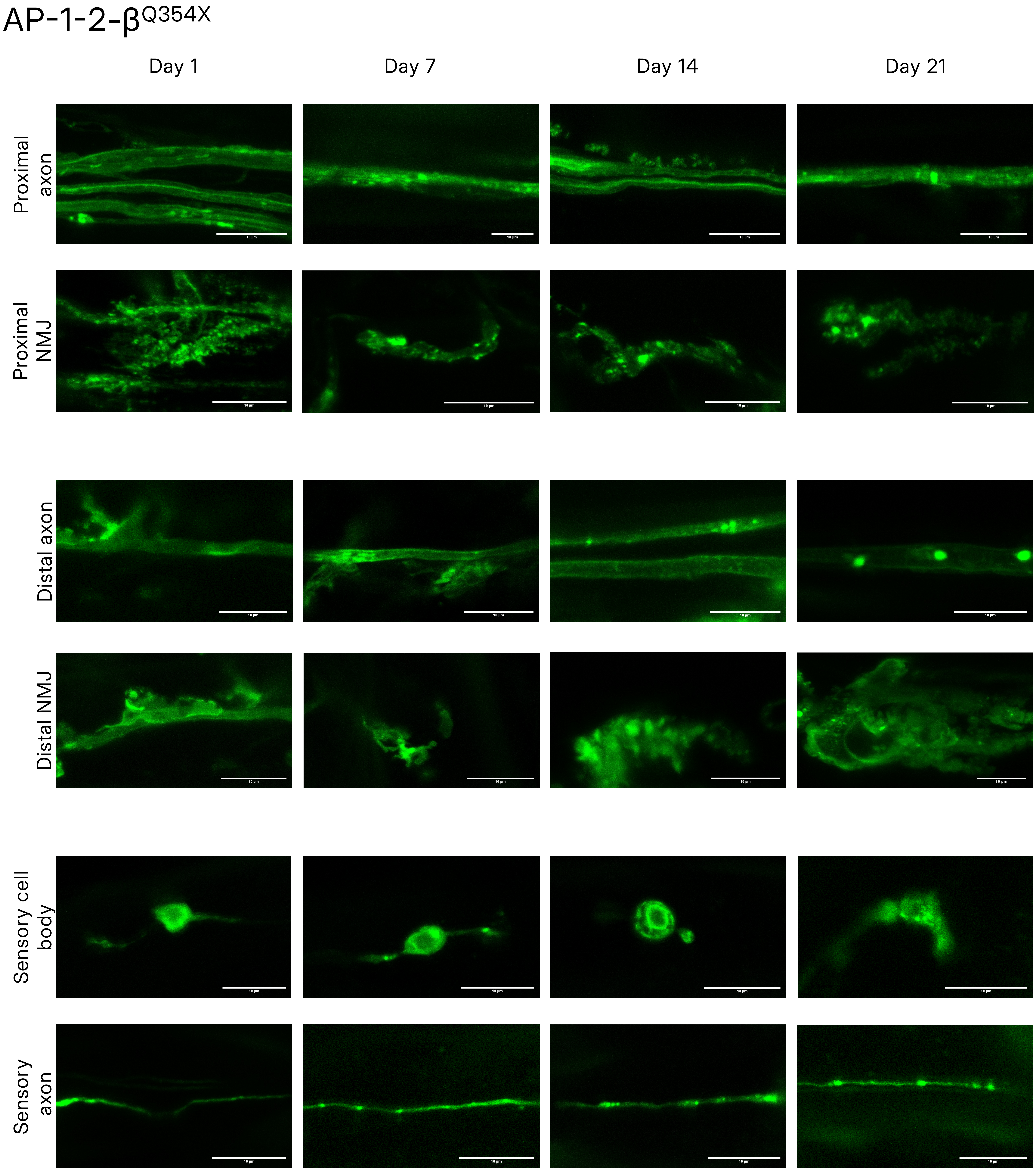
AP-1-2-β^{Q354X}
Day 1
Day 7
Day 14
Day 21
Proximal axon
Proximal NMJ
Distal axon
Distal NMJ
Sensory cell body
Sensory axon

AMPKα^{C212Y}

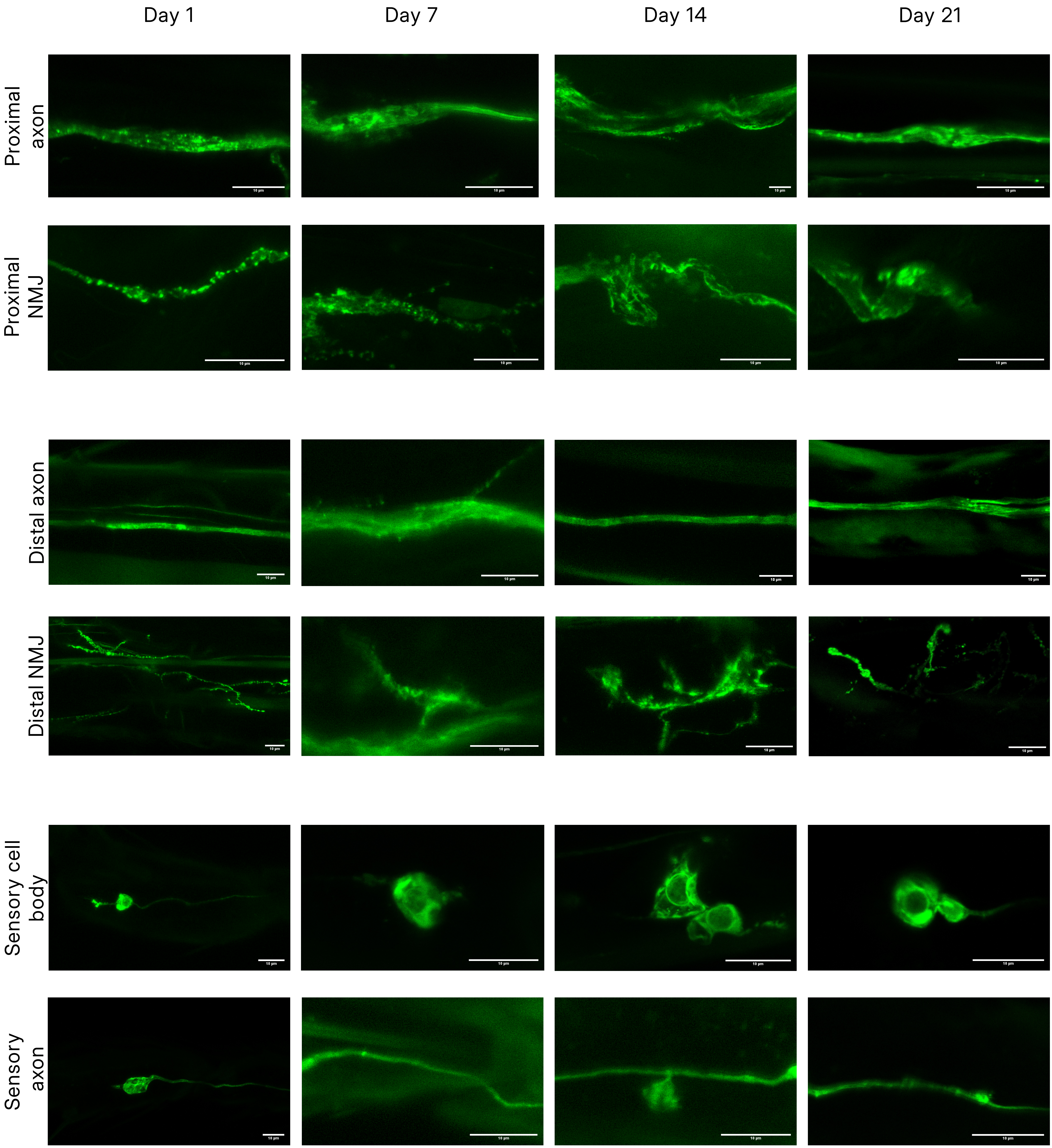

Day 1
Day 7
Day 14
Day 21
Proximal axon
Proximal NMJ
Distal axon
Distal NMJ
Sensory cell body
Sensory axon

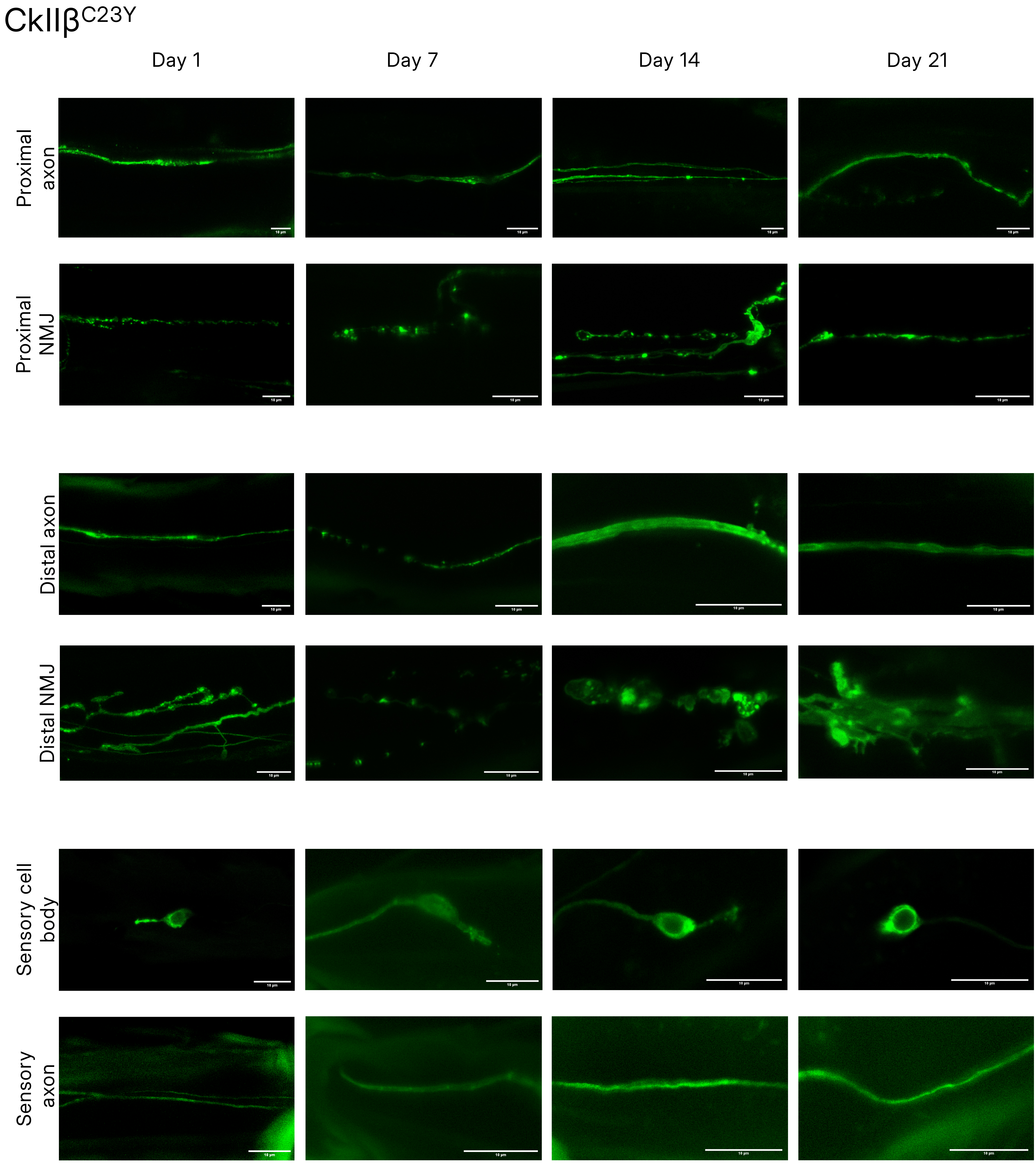

CkIIβ^C23Y
Day 1
Day 7
Day 14
Day 21
Proximal axon
Proximal NMJ
Distal axon
Distal NMJ
Sensory cell body
Sensory axon

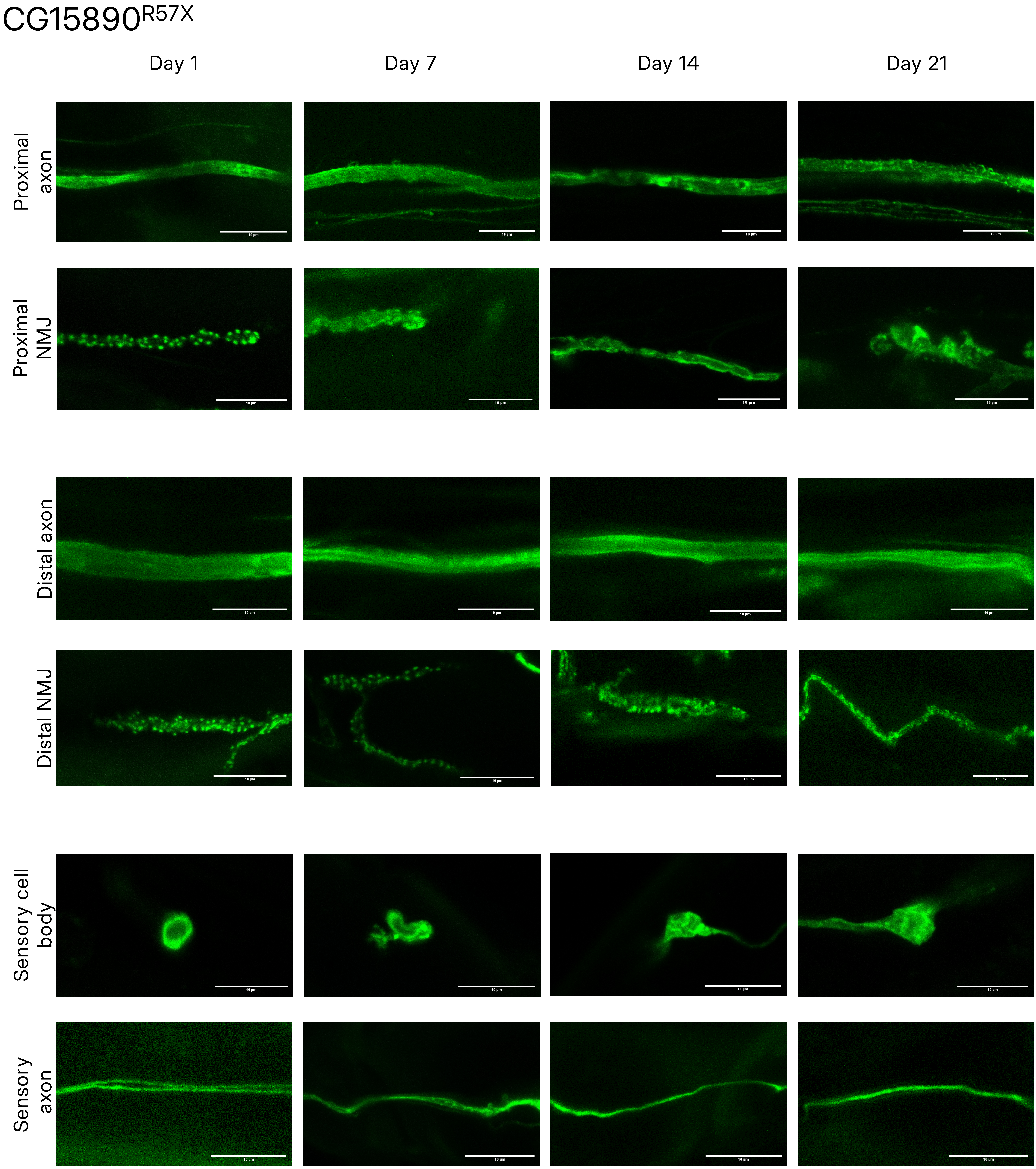

CG15890R57X
Day 1
Day 7
Day 14
Day 21
Proximal axon
Proximal NMJ
Distal axon
Distal NMJ
Sensory cell body
Sensory axon

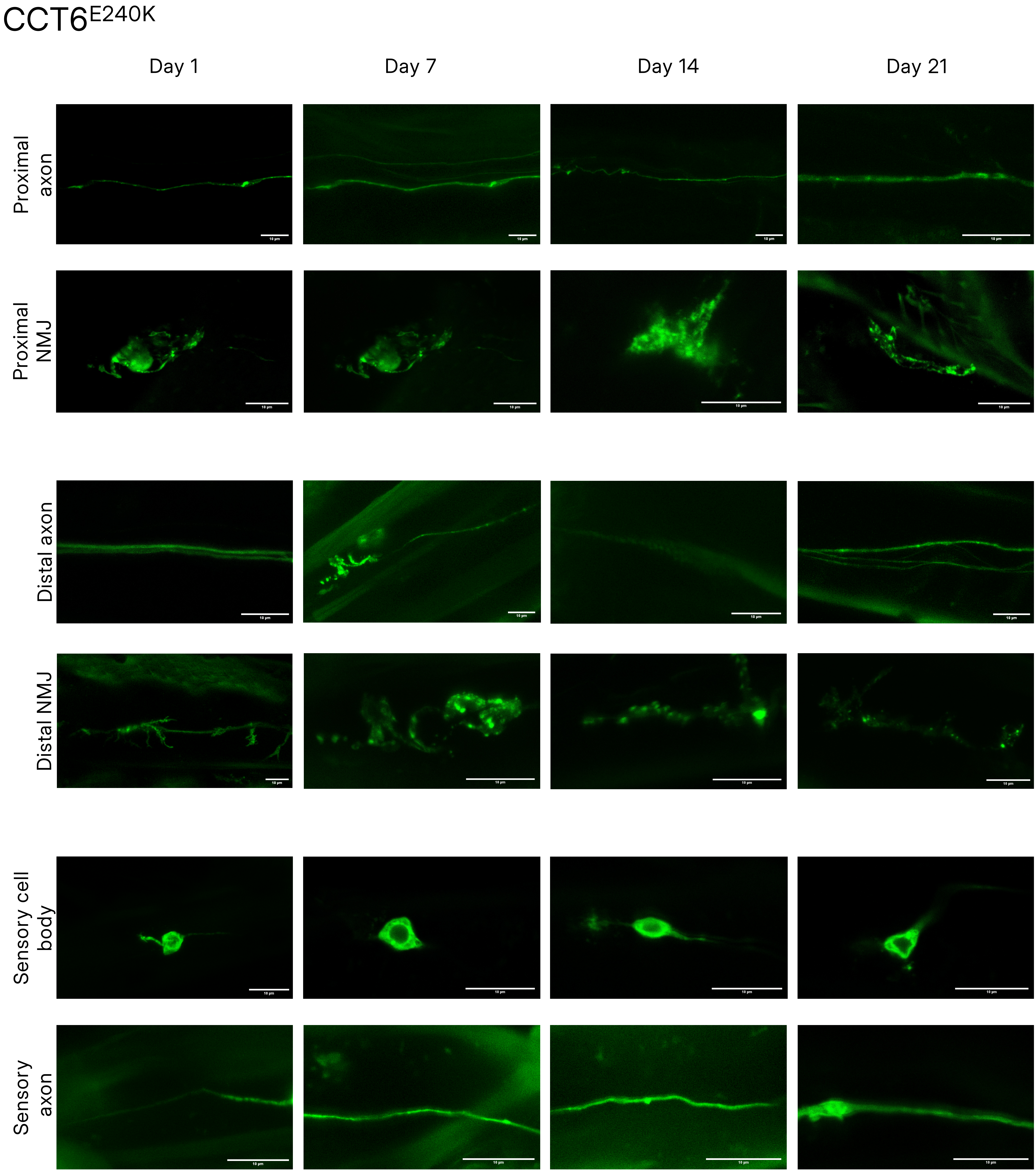

CCT6^{E240K}
Day 1
Day 7
Day 14
Day 21
Proximal axon
Proximal NMJ
Distal axon
Distal NMJ
Sensory cell body
Sensory axon

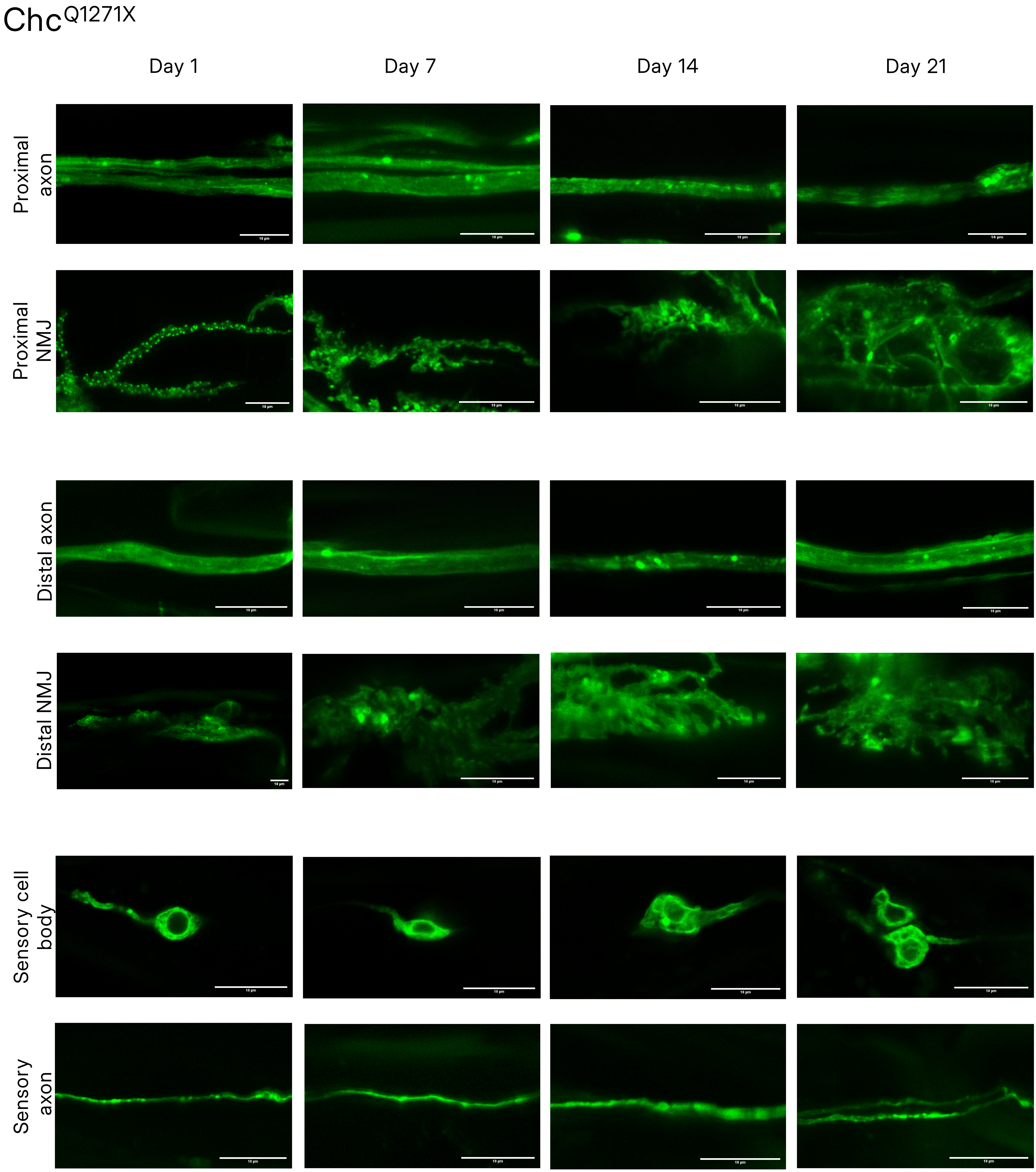
Chc^Q1271X
Day 1
Day 7
Day 14
Day 21
Proximal axon
Proximal NMJ
Distal axon
Distal NMJ
Sensory cell body
Sensory axon

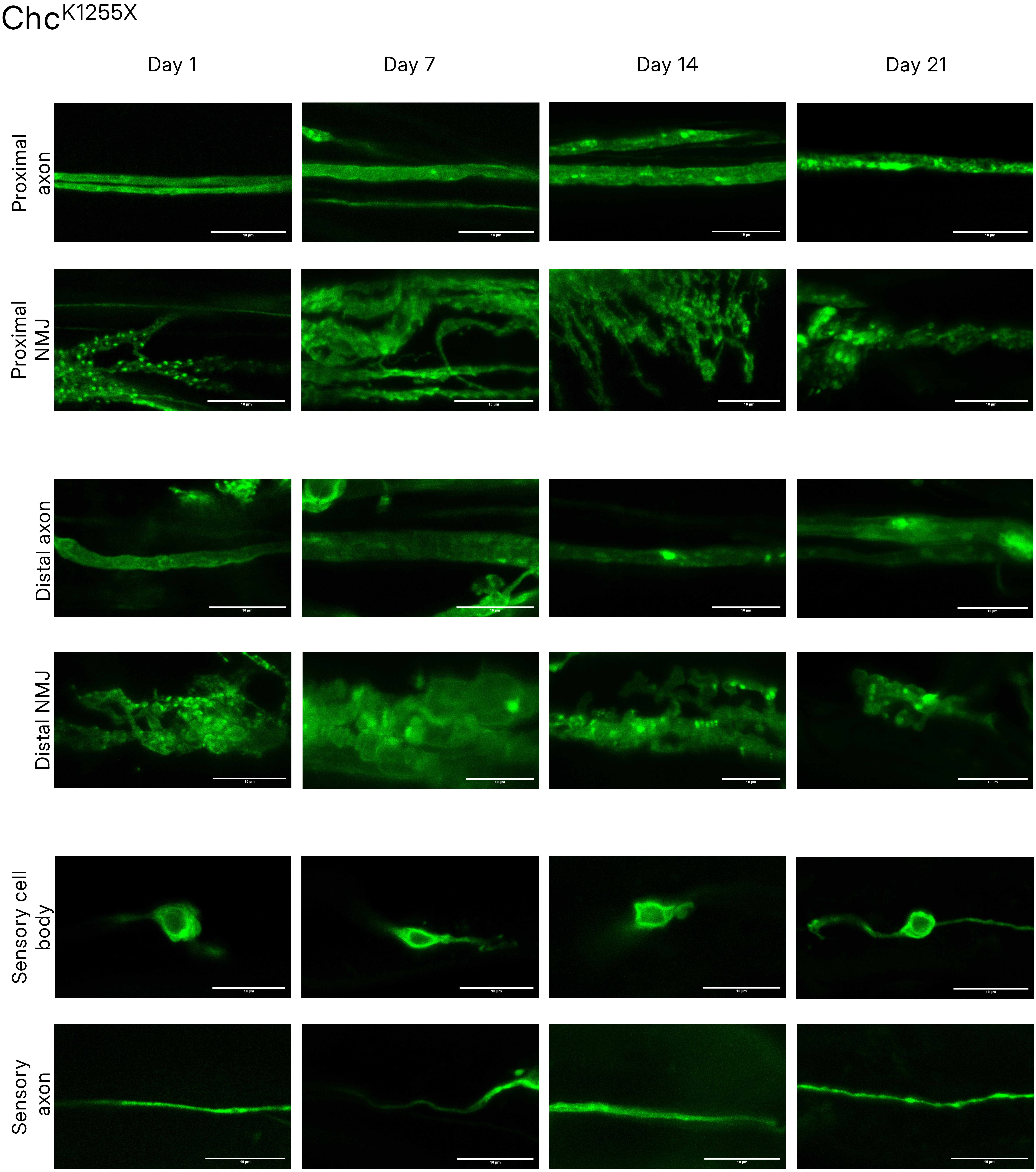

Chc^{K1255X}
Day 1
Day 7
Day 14
Day 21
Proximal axon
Proximal NMJ
Distal axon
Distal NMJ
Sensory cell body
Sensory axon

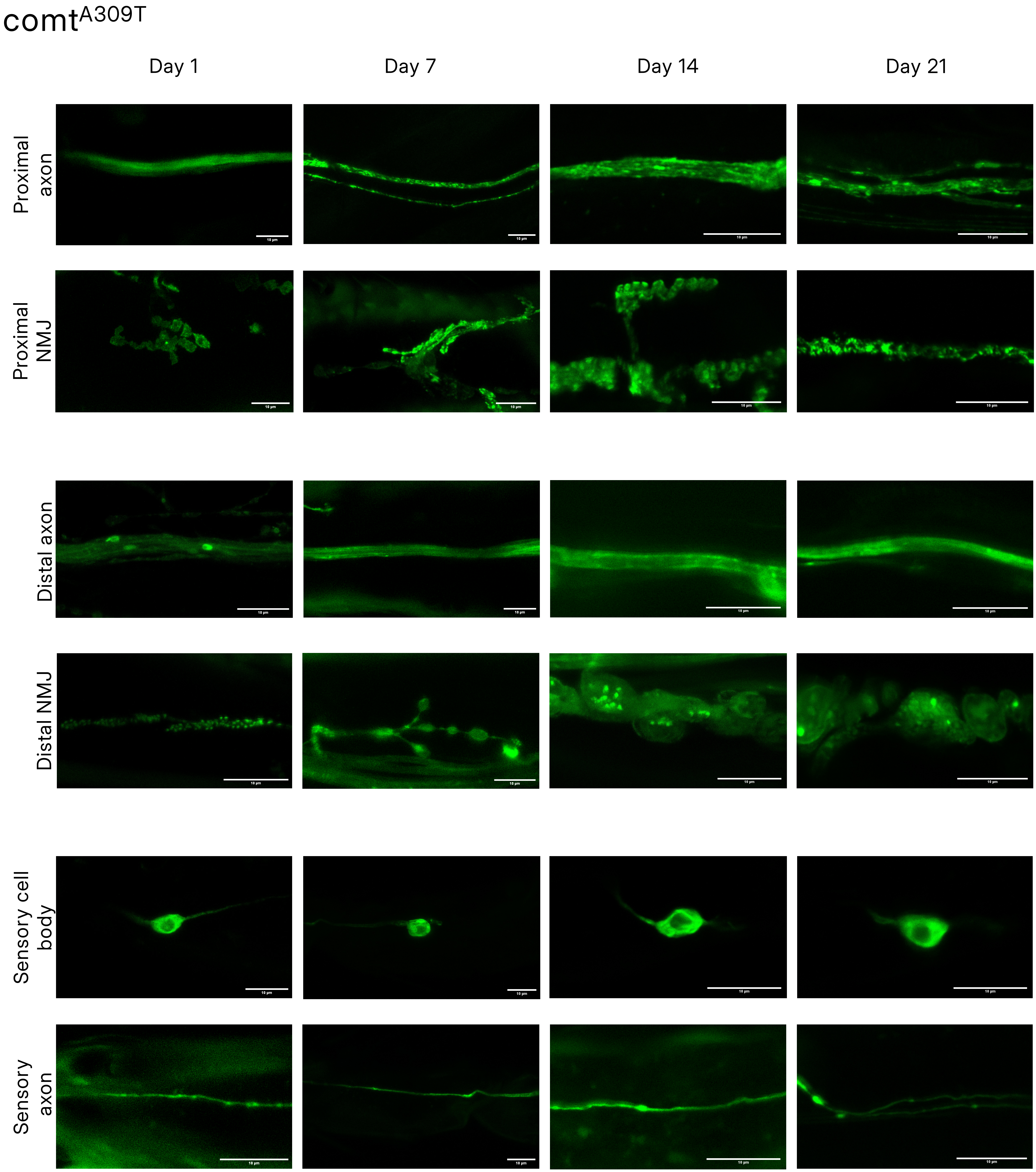
comt^A309T
Day 1
Day 7
Day 14
Day 21
Proximal axon
Proximal NMJ
Distal axon
Distal NMJ
Sensory cell body
Sensory axon

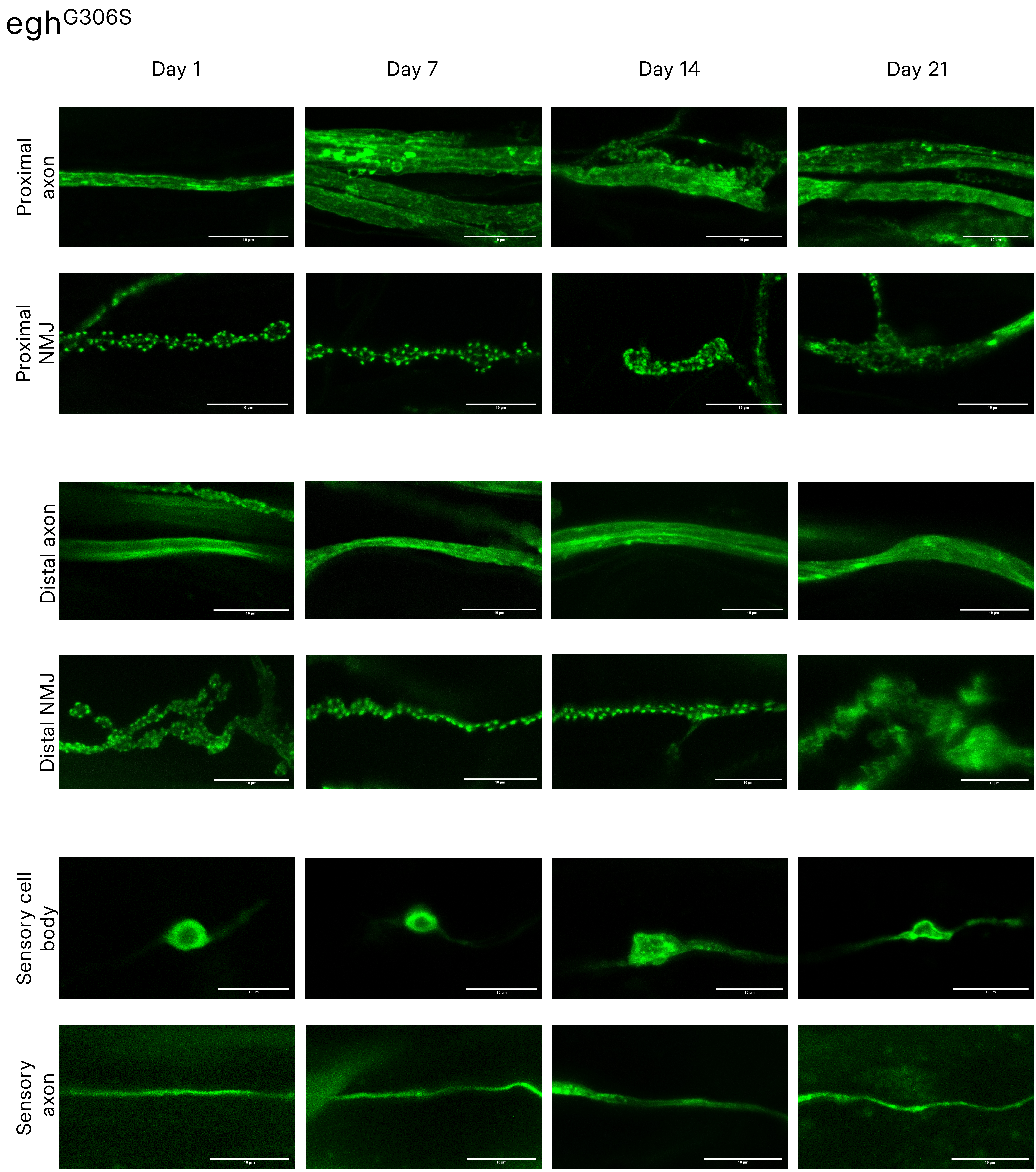
egh^{G306S}
Day 1
Day 7
Day 14
Day 21
Proximal axon
Proximal NMJ
Distal axon
Distal NMJ
Sensory cell body
Sensory axon

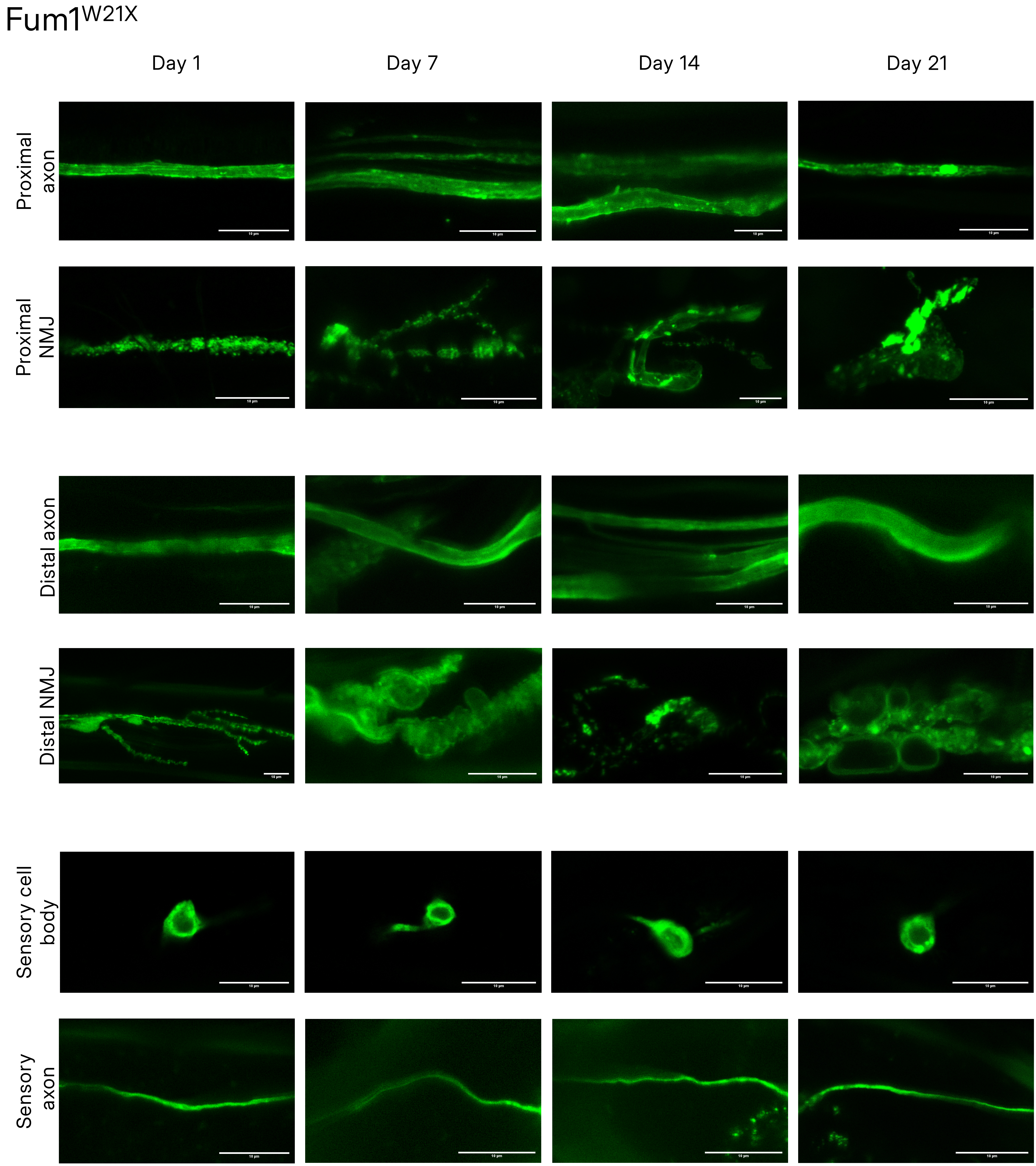
Fum1W21X
Day 1
Day 7
Day 14
Day 21
Proximal axon
Proximal NMJ
Distal axon
Distal NMJ
Sensory cell body
Sensory axon

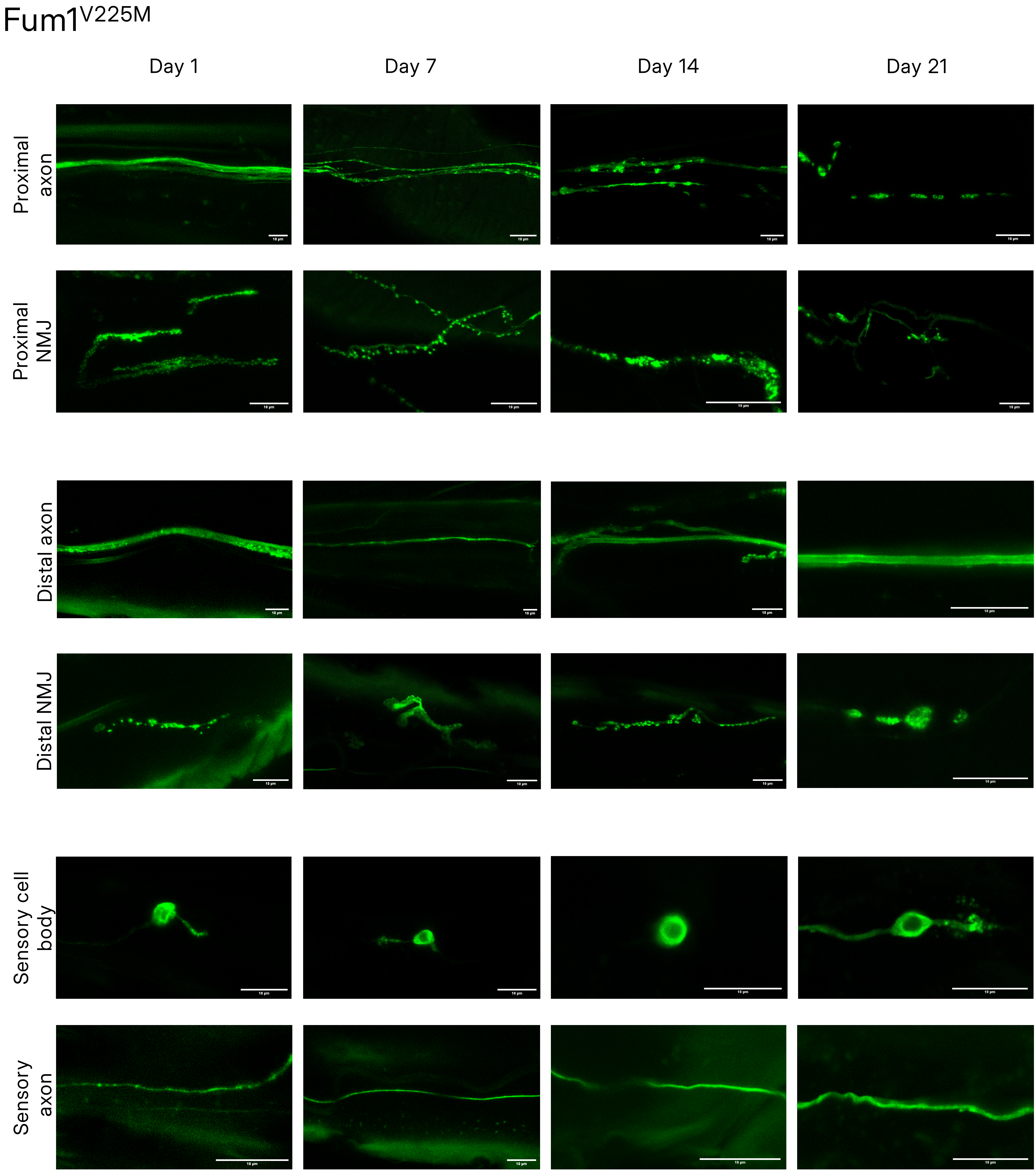

Fum1V225M
Day 1
Day 7
Day 14
Day 21
Proximal axon
Proximal NMJ
Distal axon
Distal NMJ
Sensory cell body
Sensory axon

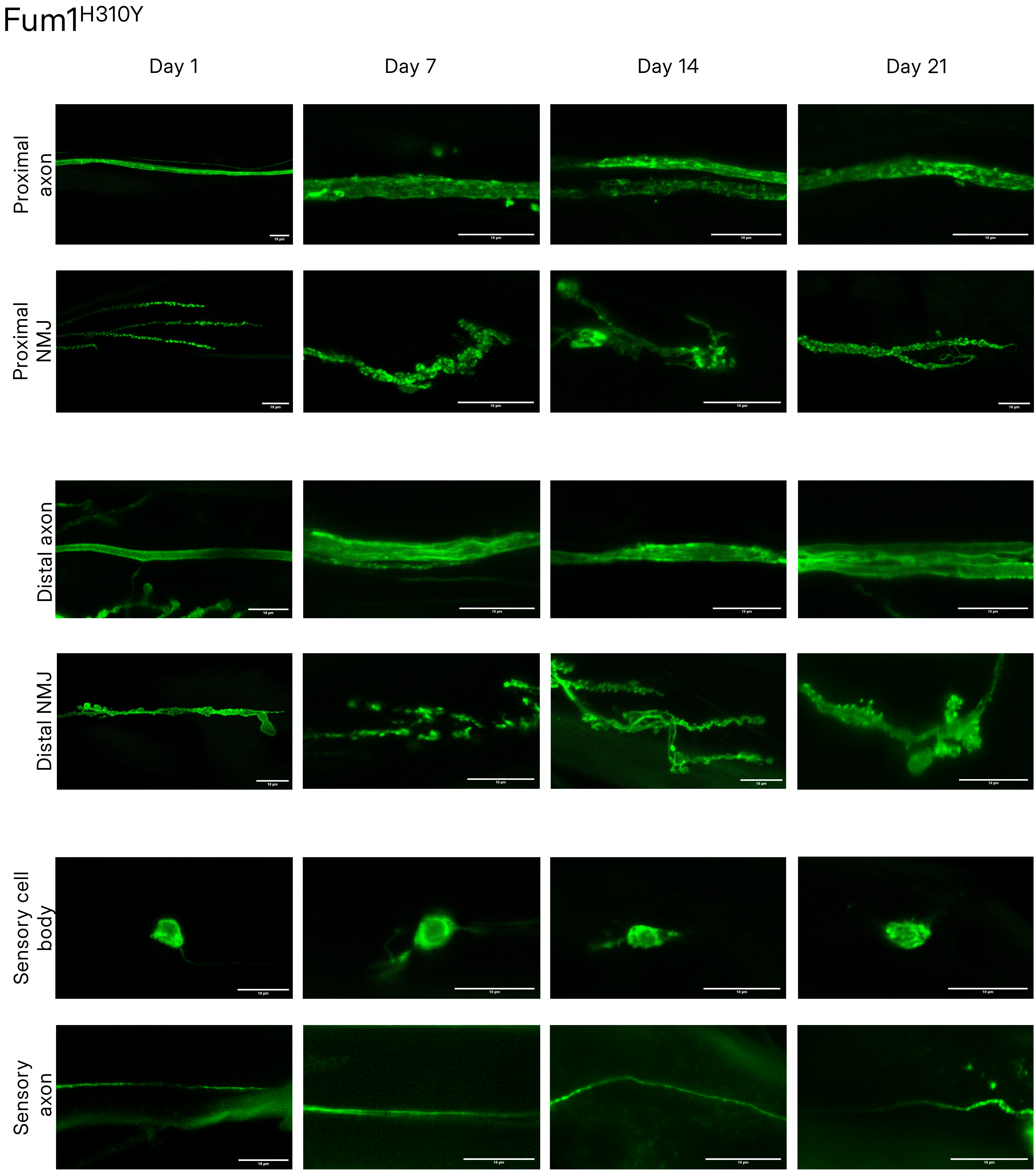
Fum1H310Y
Day 1
Day 7
Day 14
Day 21
Proximal axon
Proximal NMJ
Distal axon
Distal NMJ
Sensory cell body
Sensory axon

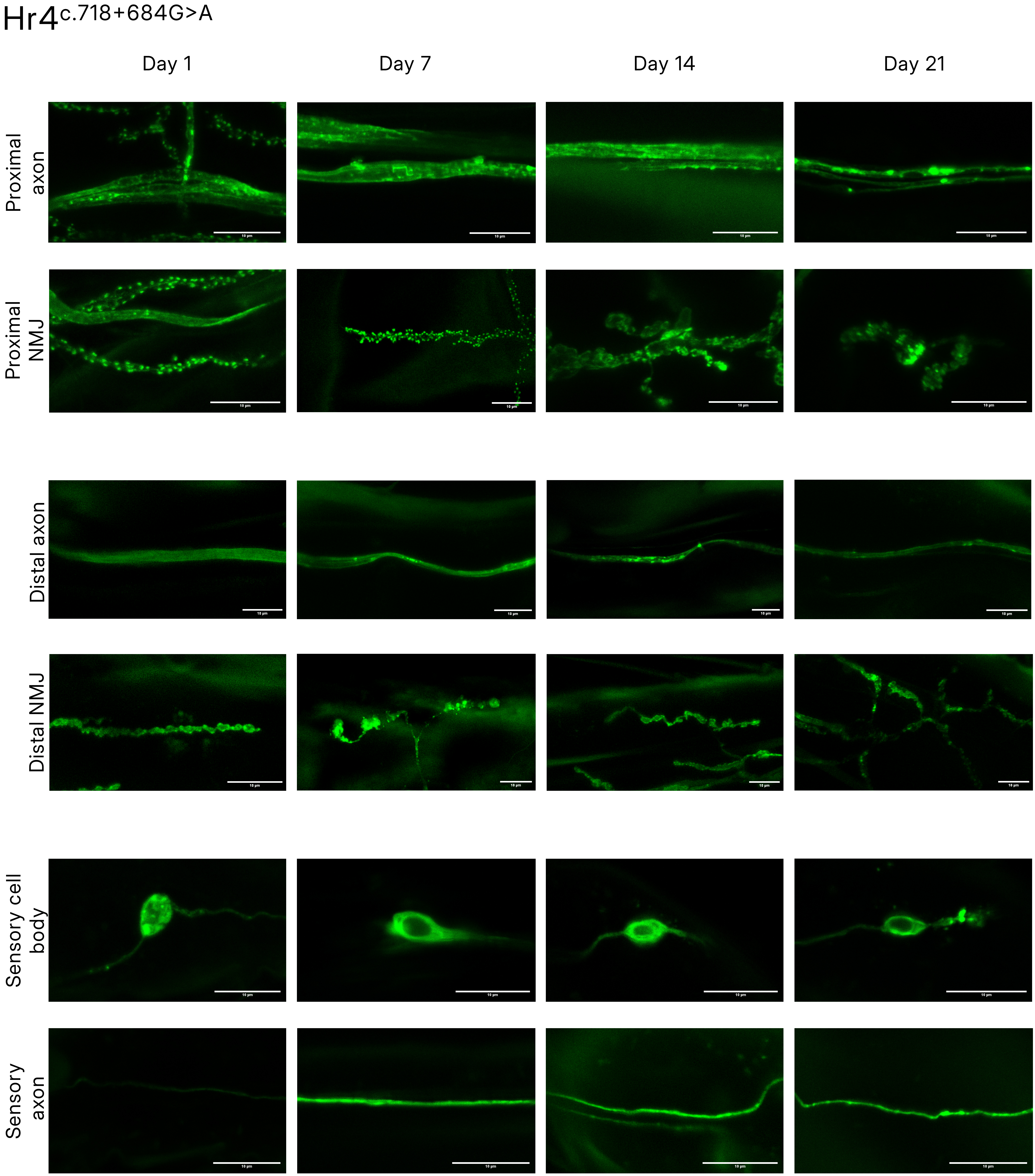

Hr4c.718+684G>A
Day 1
Day 7
Day 14
Day 21
Proximal axon
Proximal NMJ
Distal axon
Distal NMJ
Sensory cell body
Sensory axon

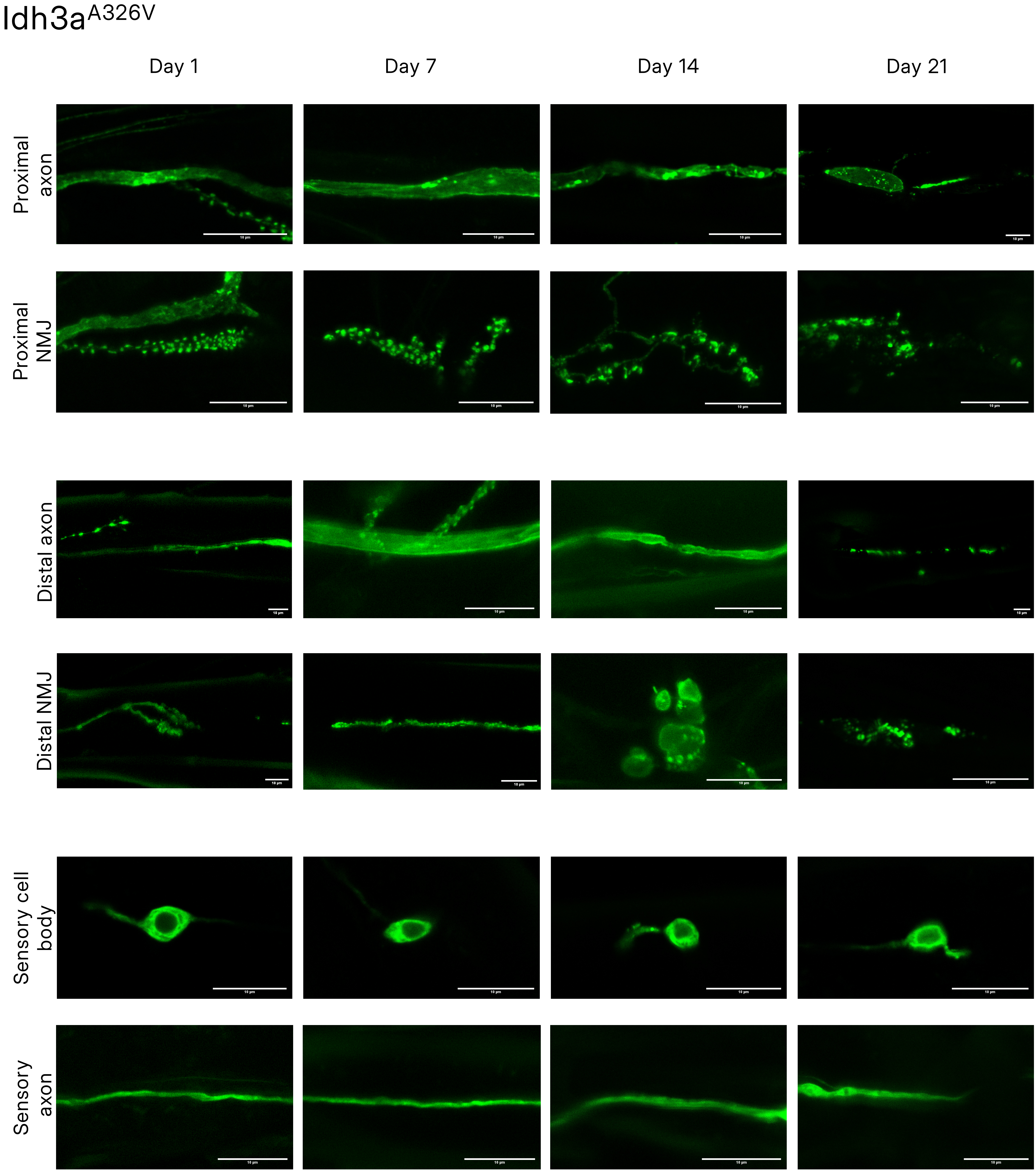
Idh3a^{A326V}
Day 1
Day 7
Day 14
Day 21
Proximal axon
Proximal NMJ
Distal axon
Distal NMJ
Sensory cell body
Sensory axon

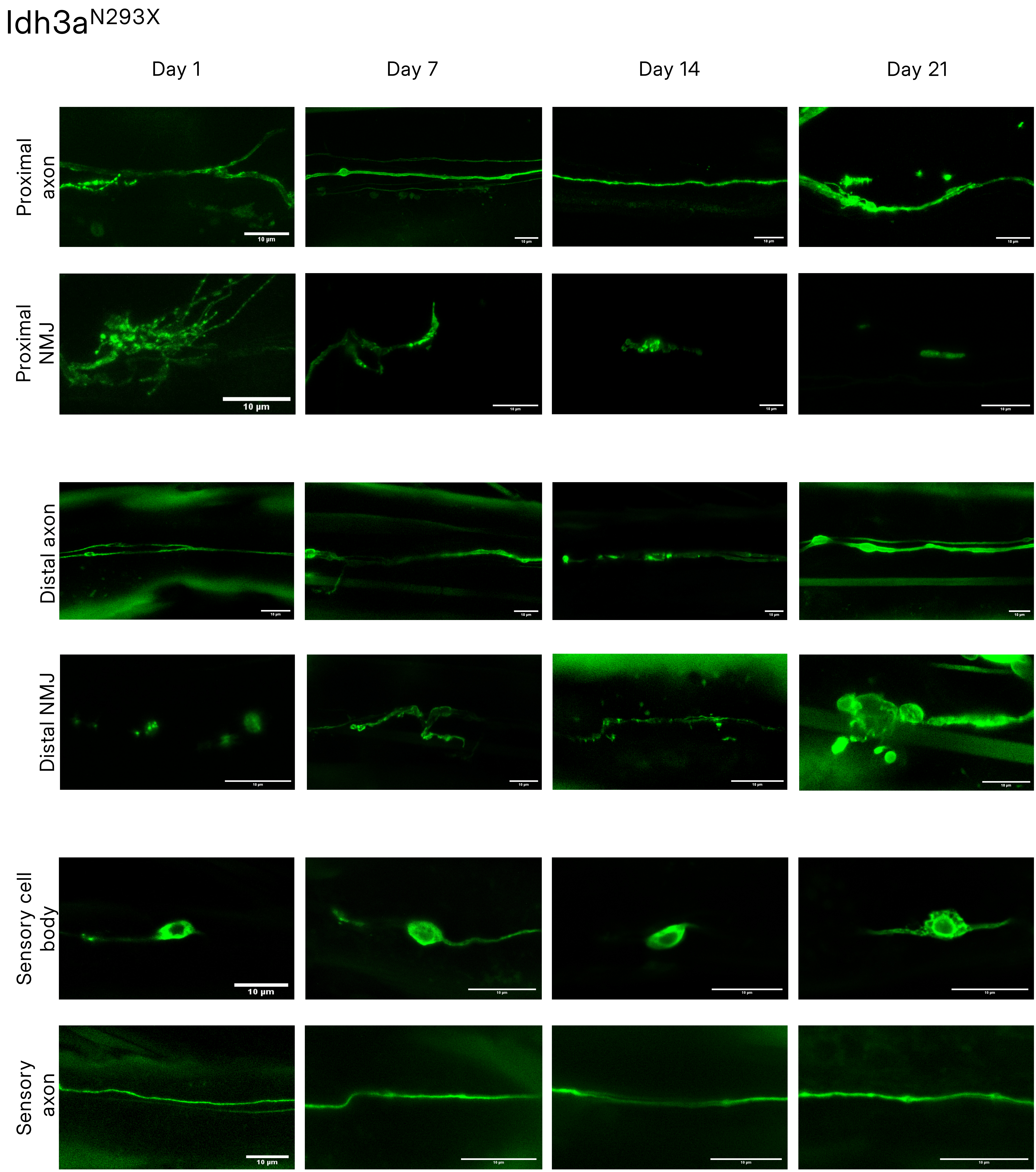
Idh3a^N293X
Day 1
Day 7
Day 14
Day 21
Proximal axon
Proximal NMJ
Distal axon
Distal NMJ
Sensory cell body
Sensory axon
10 µm

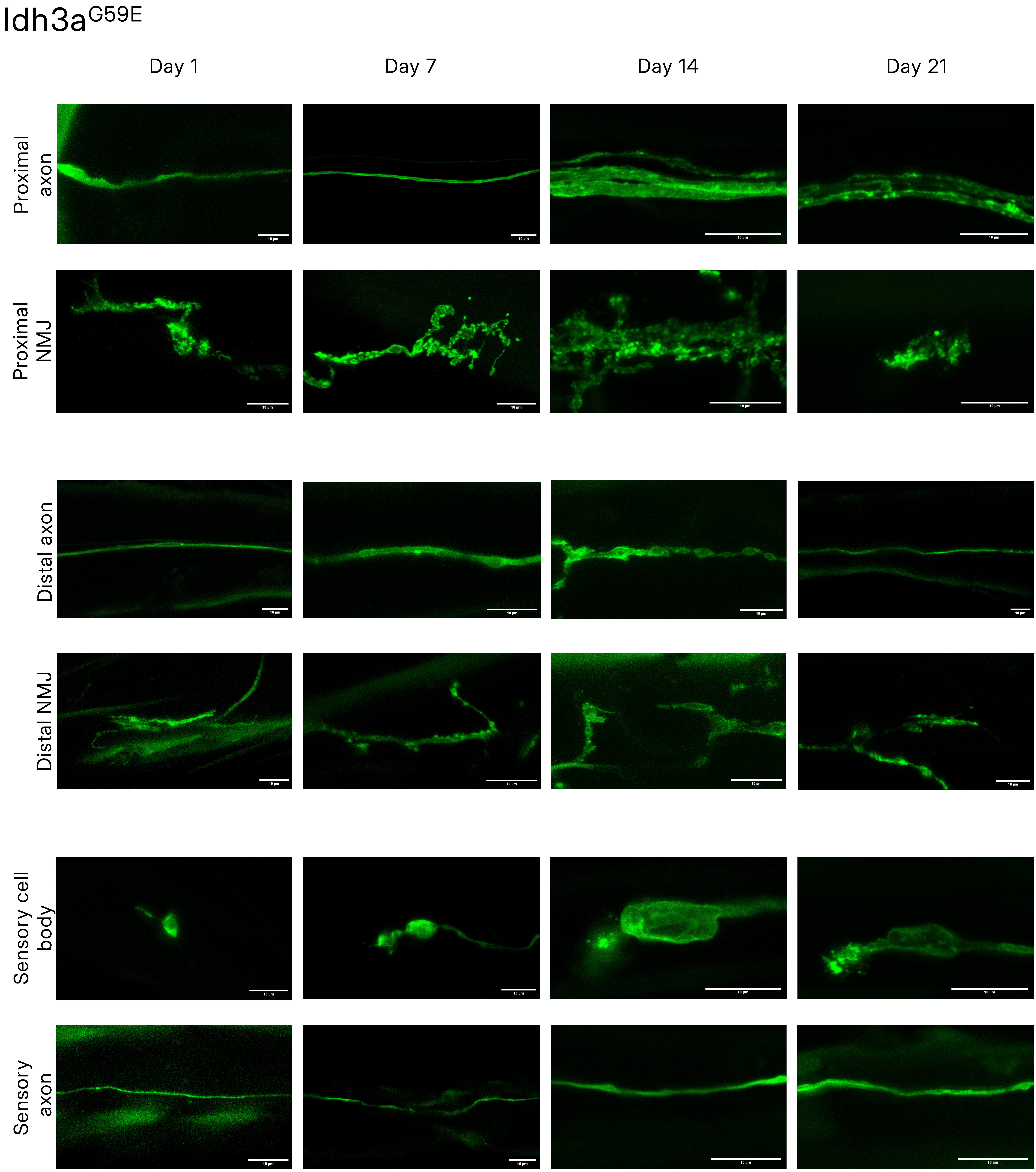
Idh3a^{G59E}
Day 1
Day 7
Day 14
Day 21
Proximal axon
Proximal NMJ
Distal axon
Distal NMJ
Sensory cell body
Sensory axon

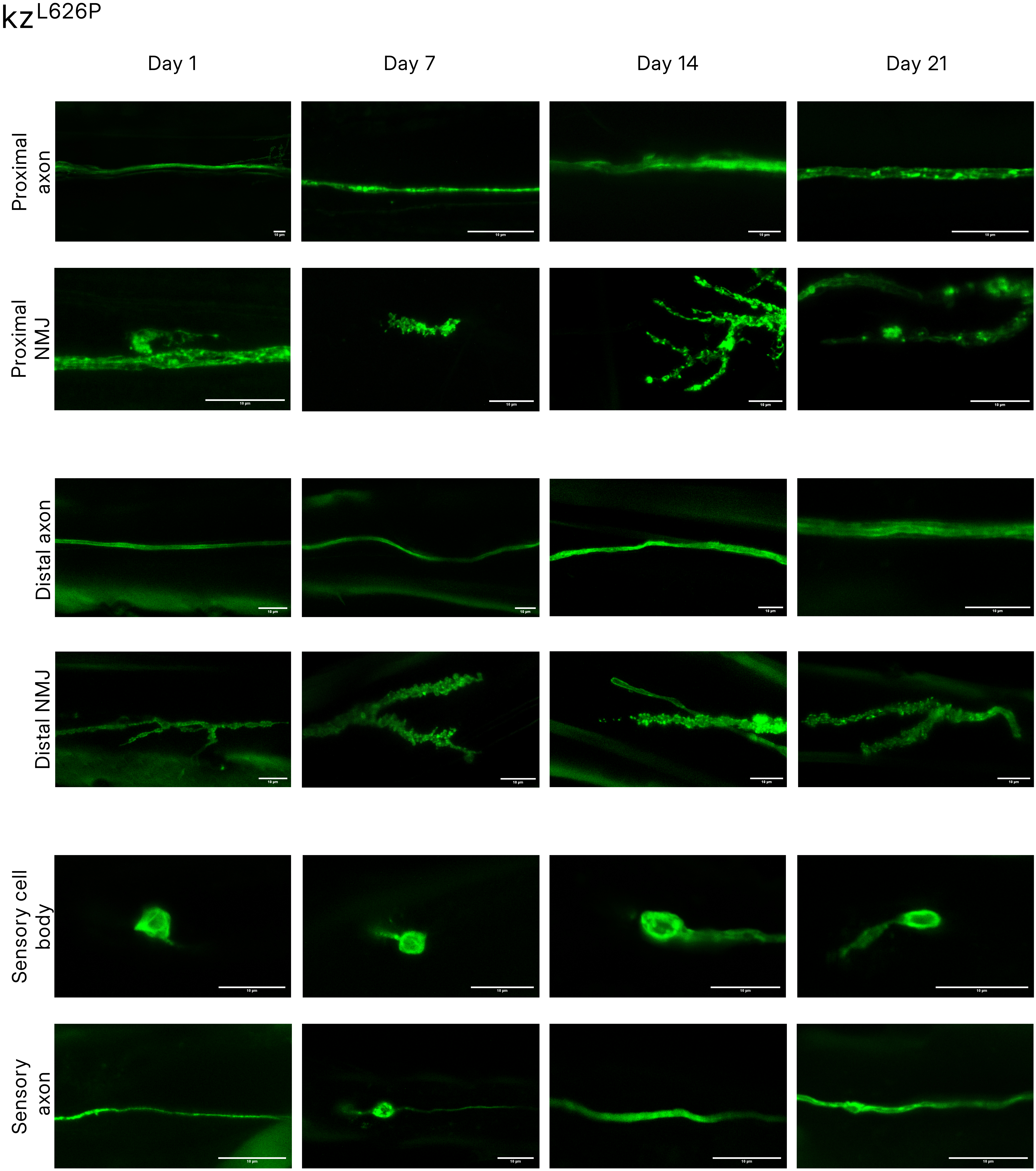

kz L626P
Day 1
Day 7
Day 14
Day 21
Proximal axon
Proximal NMJ
Distal axon
Distal NMJ
Sensory cell body
Sensory axon

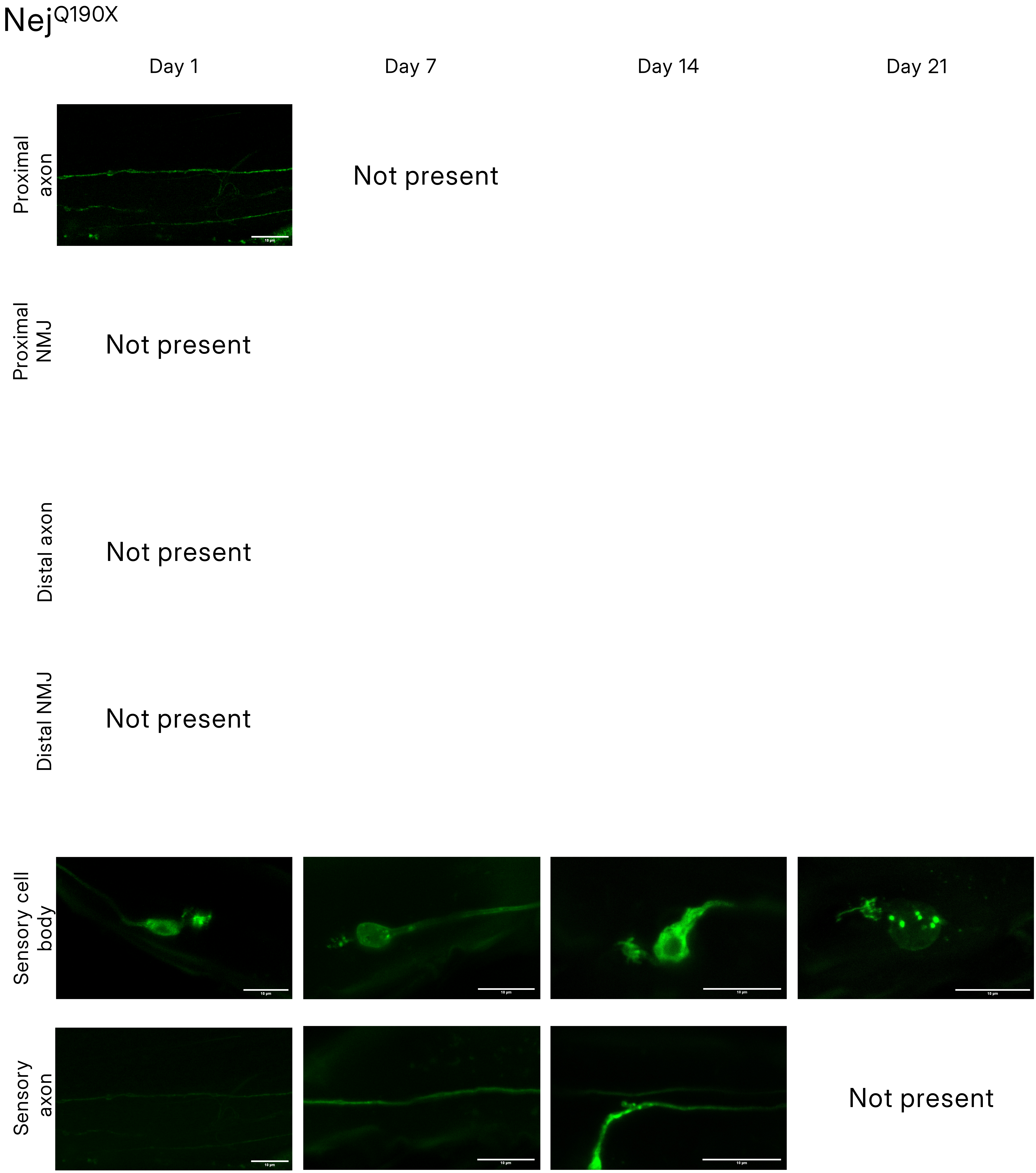
Nej Q190X
Day 1
Day 7
Day 14
Day 21
Proximal axon
Not present
Proximal NMJ
Not present
Distal axon
Not present
Distal NMJ
Not present
Sensory cell body
Sensory axon
Not present

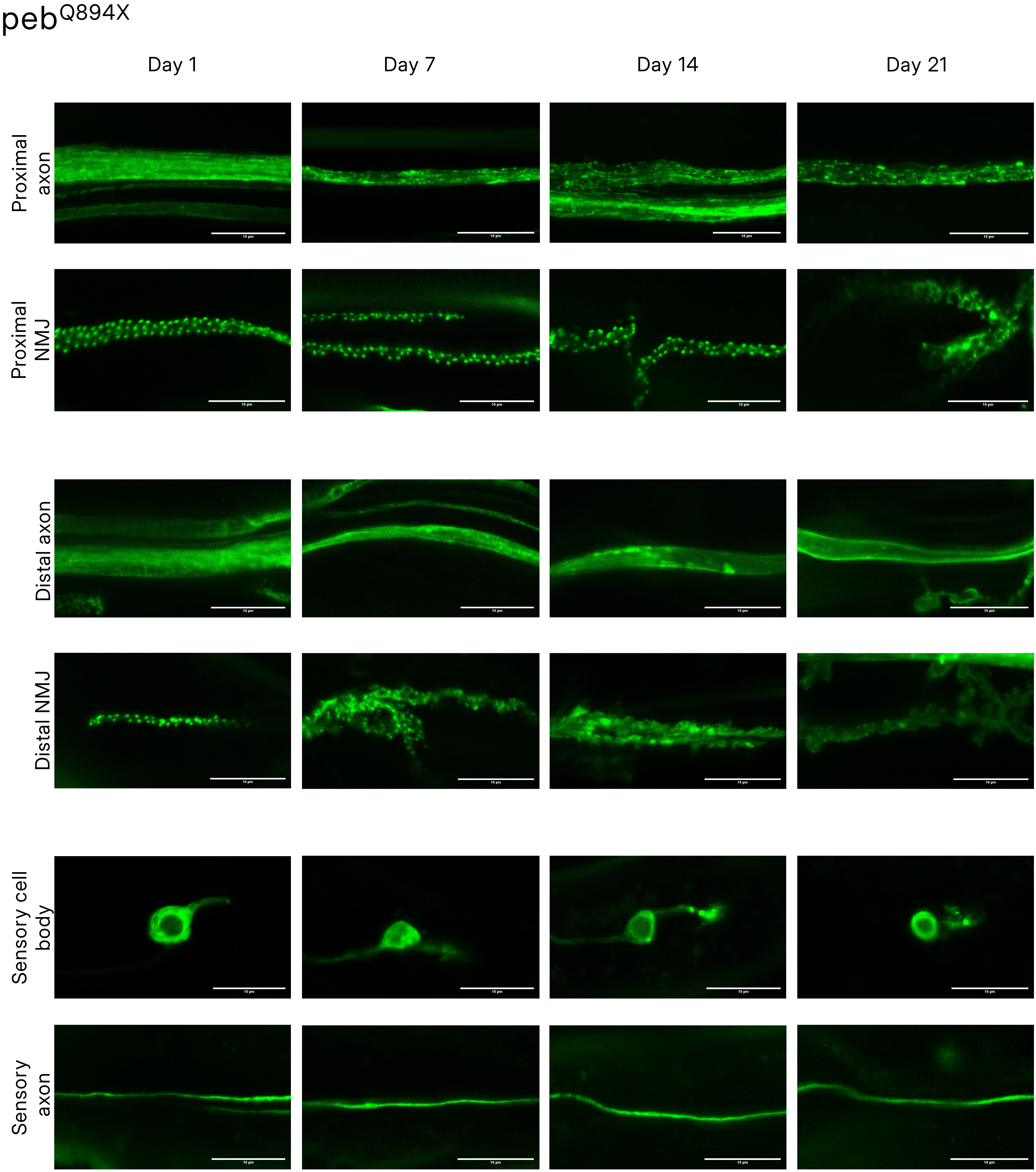
pebQ894X
Day 1
Day 7
Day 14
Day 21
Proximal axon
Proximal NMJ
Distal axon
Distal NMJ
Sensory cell body
Sensory axon

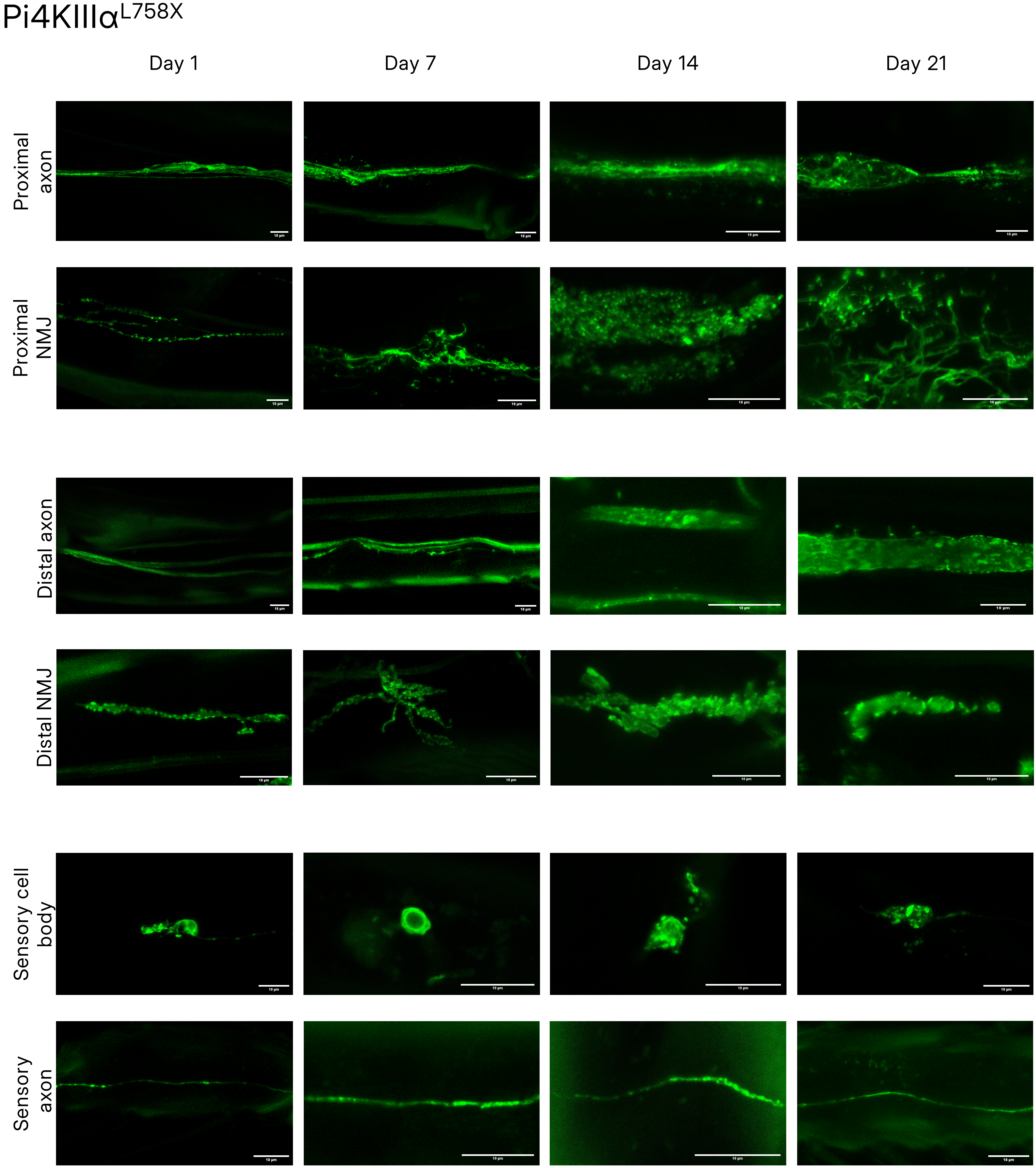
Pi4KIIIα^{L758X}
Day 1
Day 7
Day 14
Day 21
Proximal axon
Proximal NMJ
Distal axon
Distal NMJ
Sensory cell body
Sensory axon
10 μm

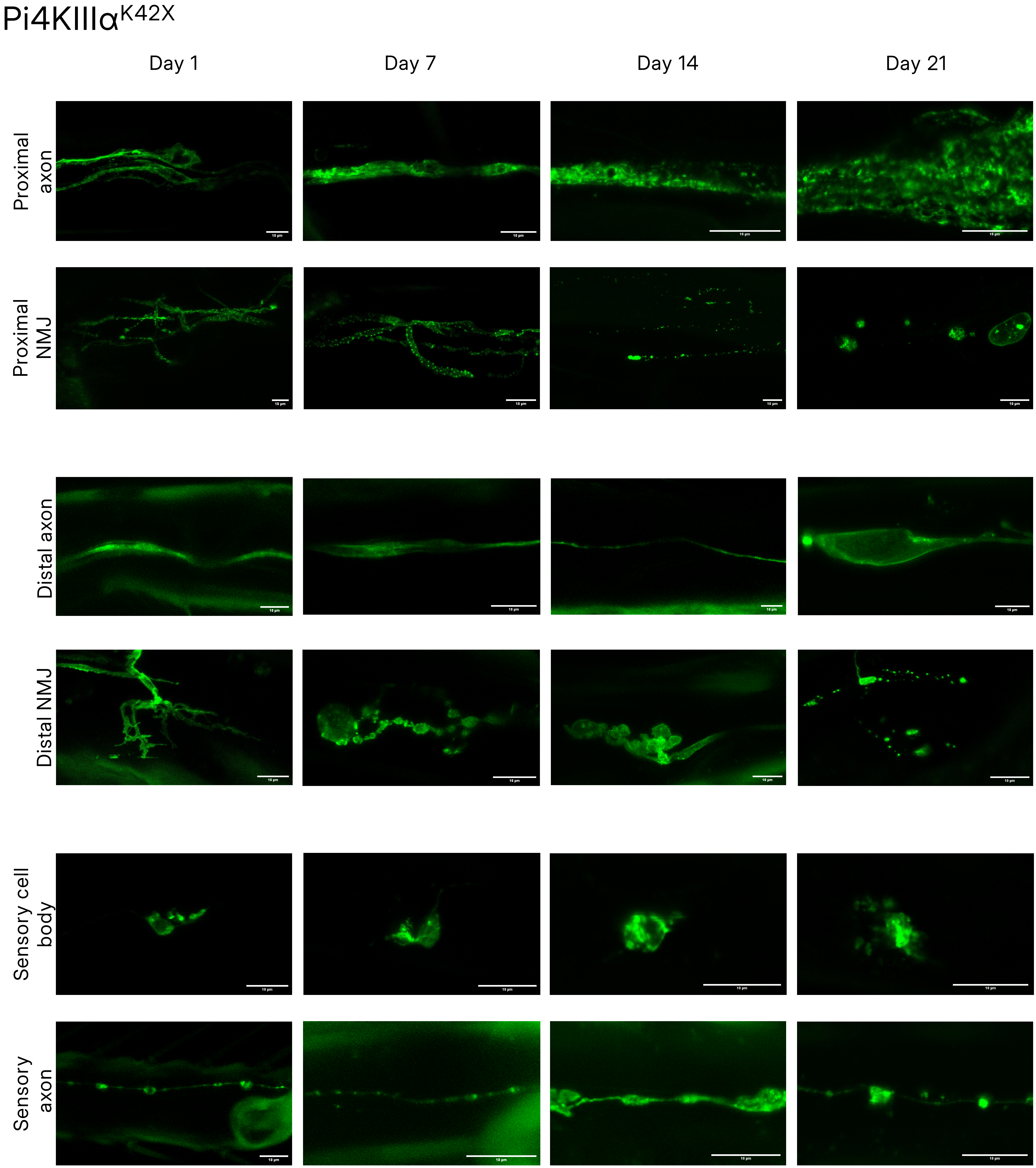
Pi4KIIIα^{K42X}
Day 1
Day 7
Day 14
Day 21
Proximal axon
Proximal NMJ
Distal axon
Distal NMJ
Sensory cell body
Sensory axon

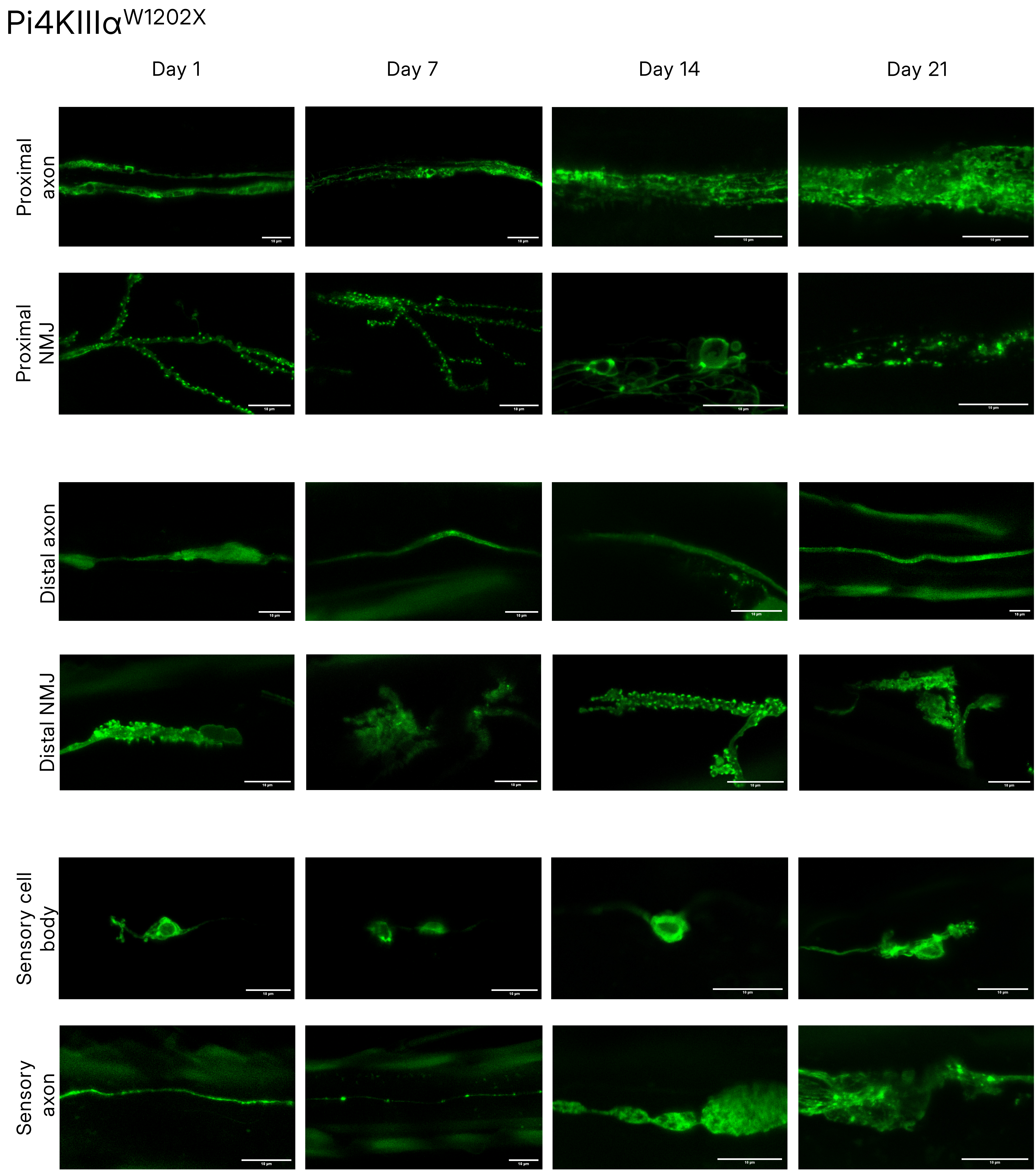
Pi4KIIIα W1202X
Day 1
Day 7
Day 14
Day 21
Proximal axon
Proximal NMJ
Distal axon
Distal NMJ
Sensory cell body
Sensory axon

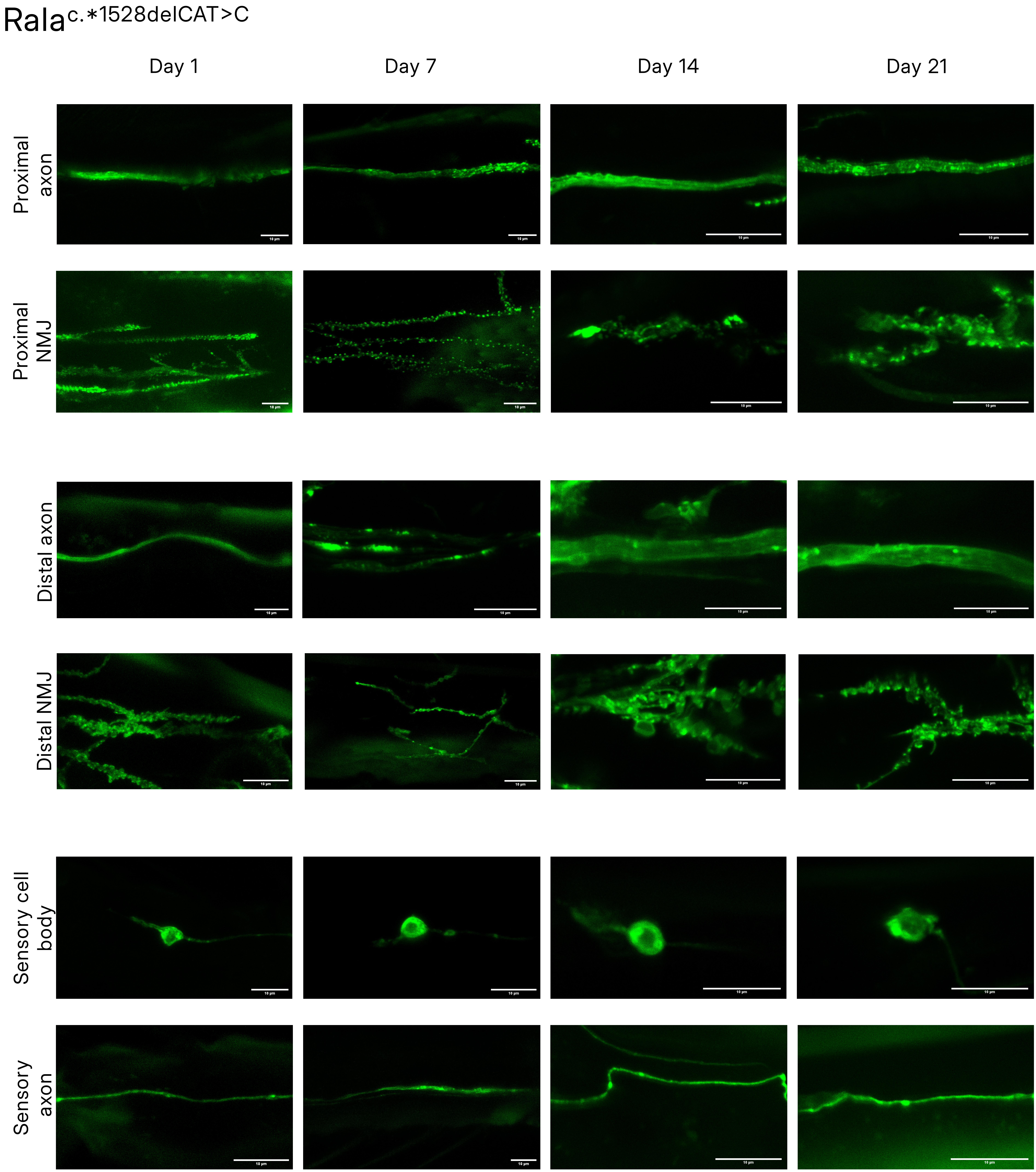
Rala c.*1528delCAT>C
Day 1
Day 7
Day 14
Day 21
Proximal axon
Proximal NMJ
Distal axon
Distal NMJ
Sensory cell body
Sensory axon

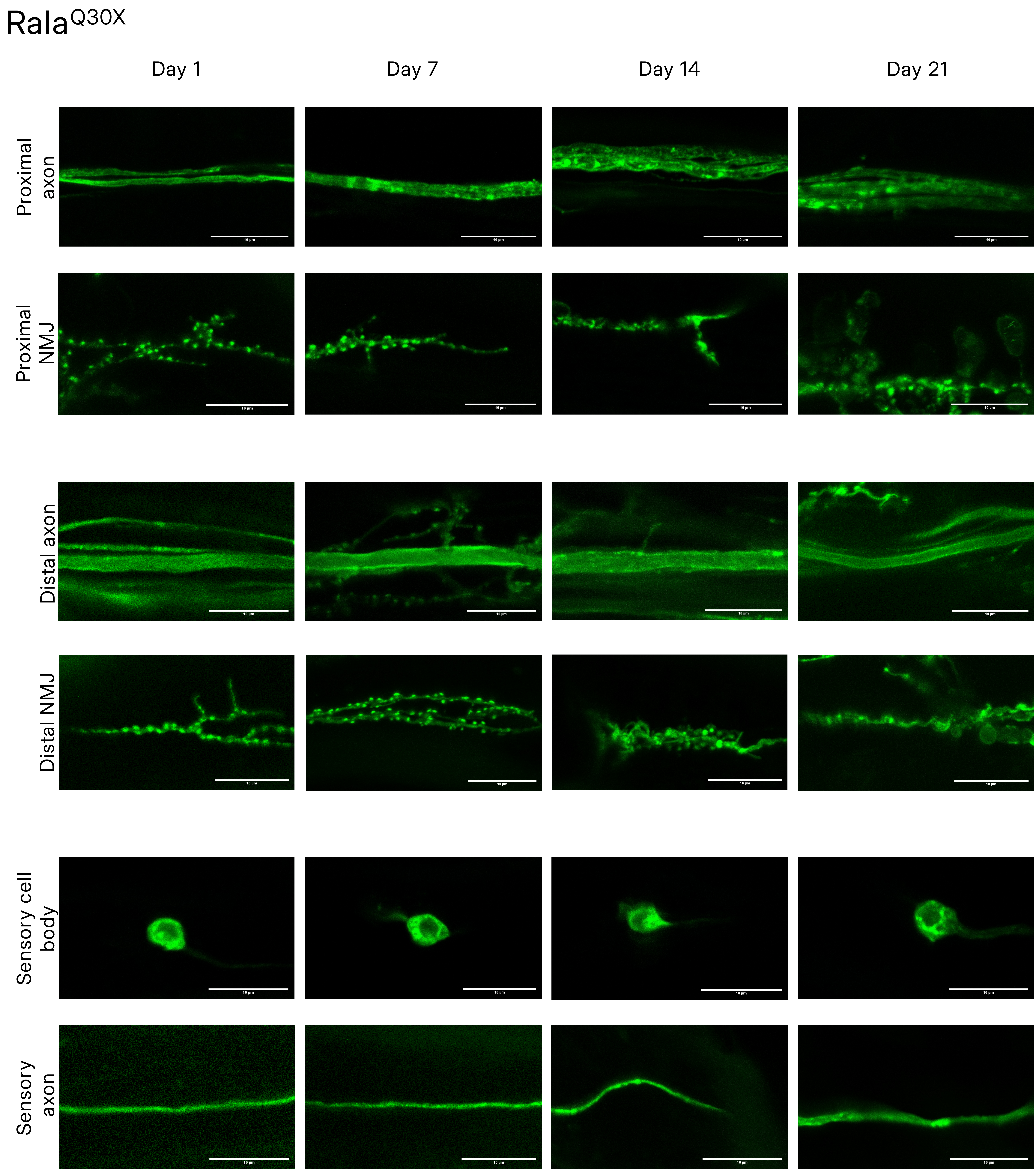

Rala^{Q30X}
Day 1
Day 7
Day 14
Day 21
Proximal axon
Proximal NMJ
Distal axon
Distal NMJ
Sensory cell body
Sensory axon

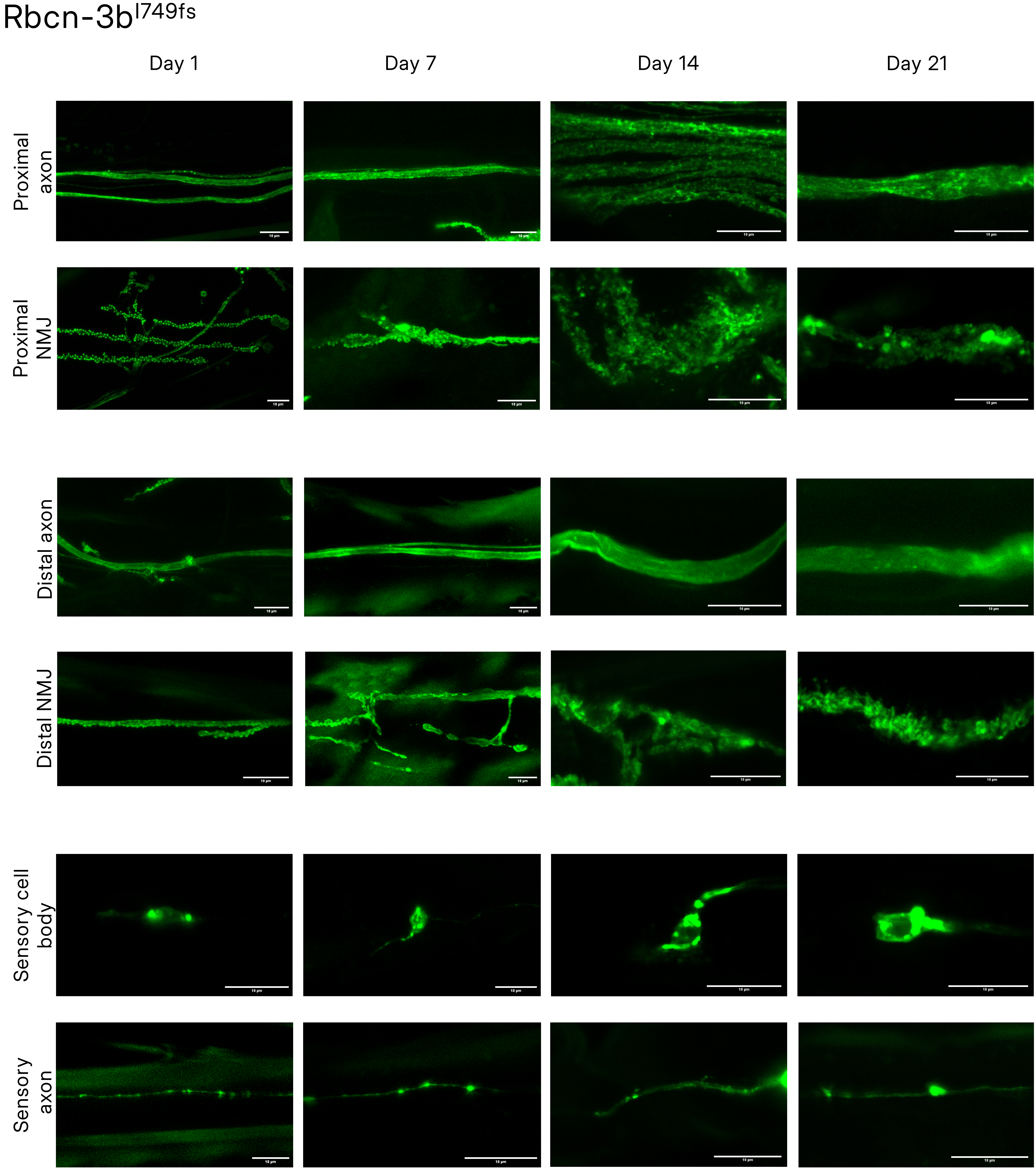
Rbcn-3b^{I749fs}
Day 1
Day 7
Day 14
Day 21
Proximal axon
Proximal NMJ
Distal axon
Distal NMJ
Sensory cell body
Sensory axon

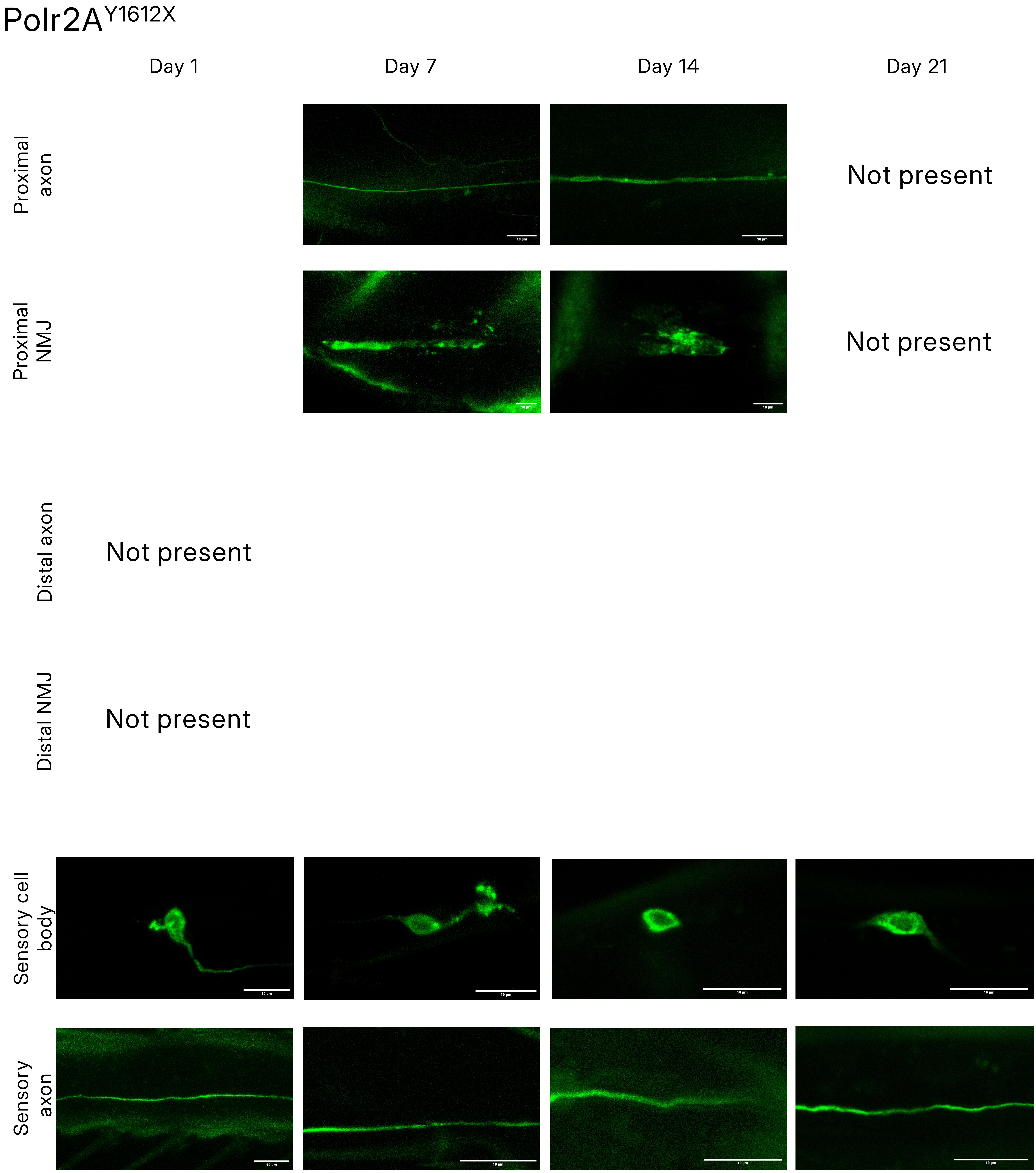
Polr2A^{Y1612X}
Day 1
Day 7
Day 14
Day 21
Proximal axon
Not present
Proximal NMJ
Not present
Distal axon
Not present
Distal NMJ
Not present
Sensory cell body
Sensory axon

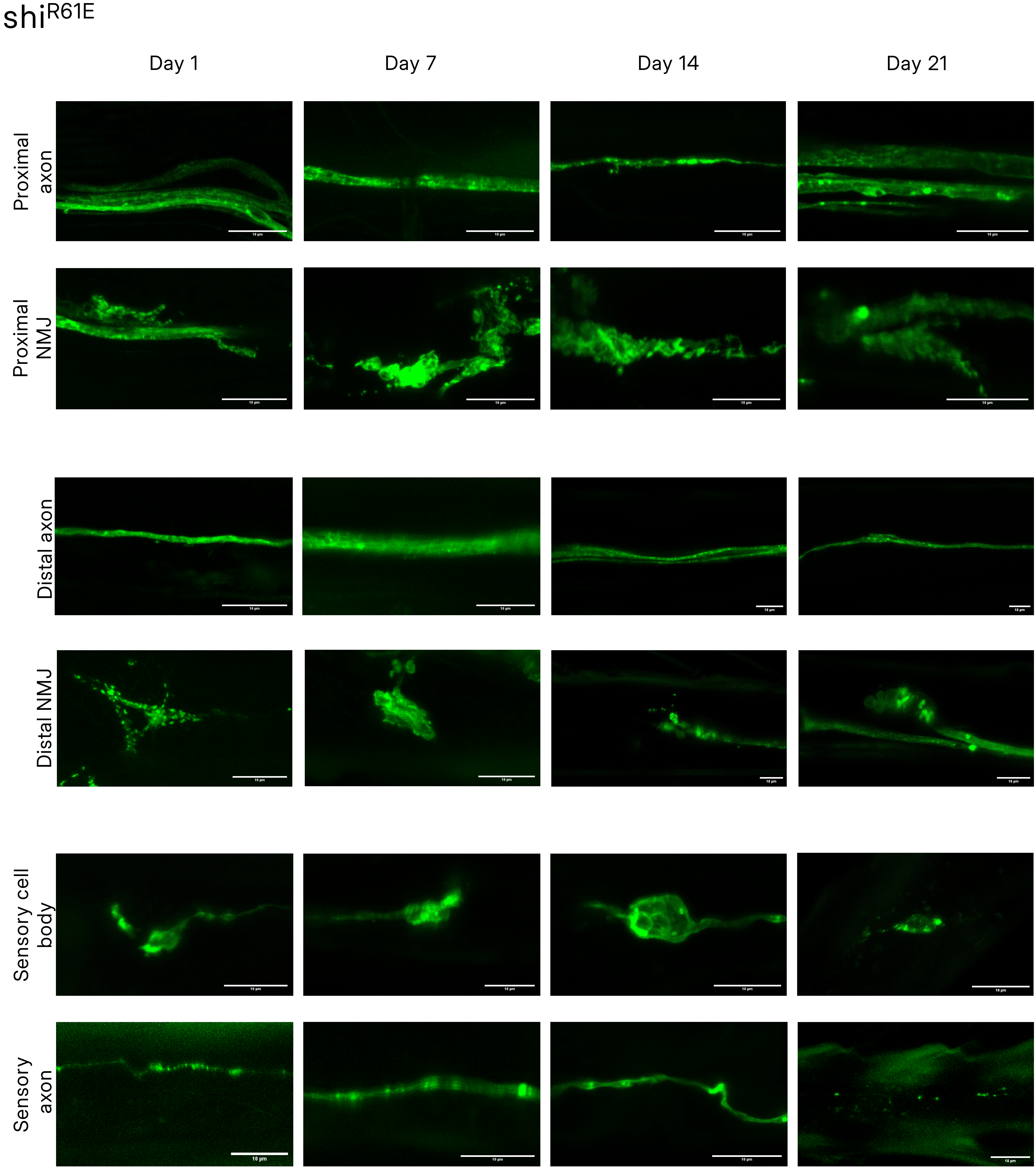
shiR61E
Day 1
Day 7
Day 14
Day 21
Proximal axon
Proximal NMJ
Distal axon
Distal NMJ
Sensory cell body
Sensory axon

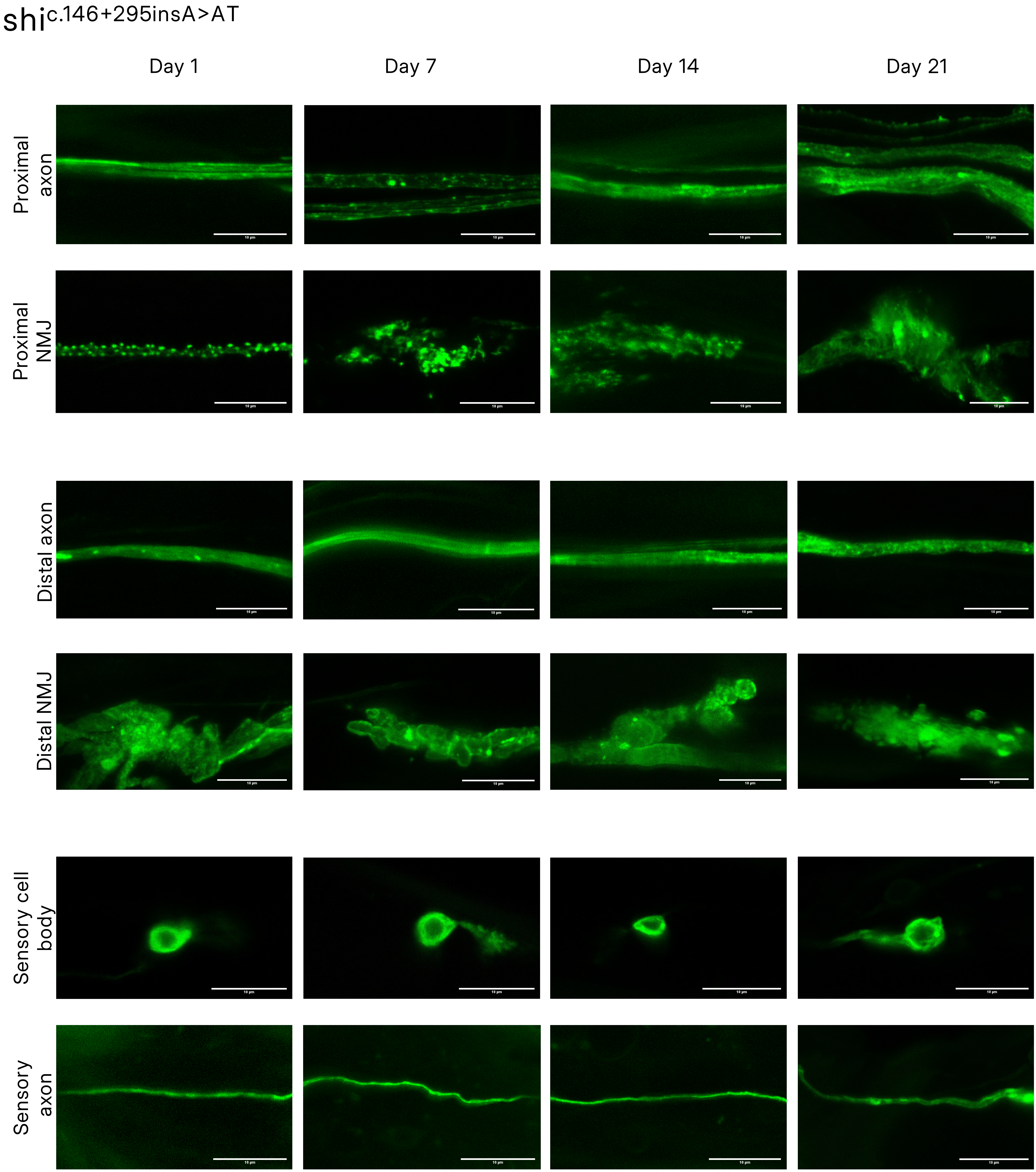

$shi^{c.146+295insA>AT}$
Day 1
Day 7
Day 14
Day 21
Proximal axon
Proximal NMJ
Distal axon
Distal NMJ
Sensory cell body
Sensory axon

$shi^{c.1959+1108insA>AAAAAAAAAAC}$

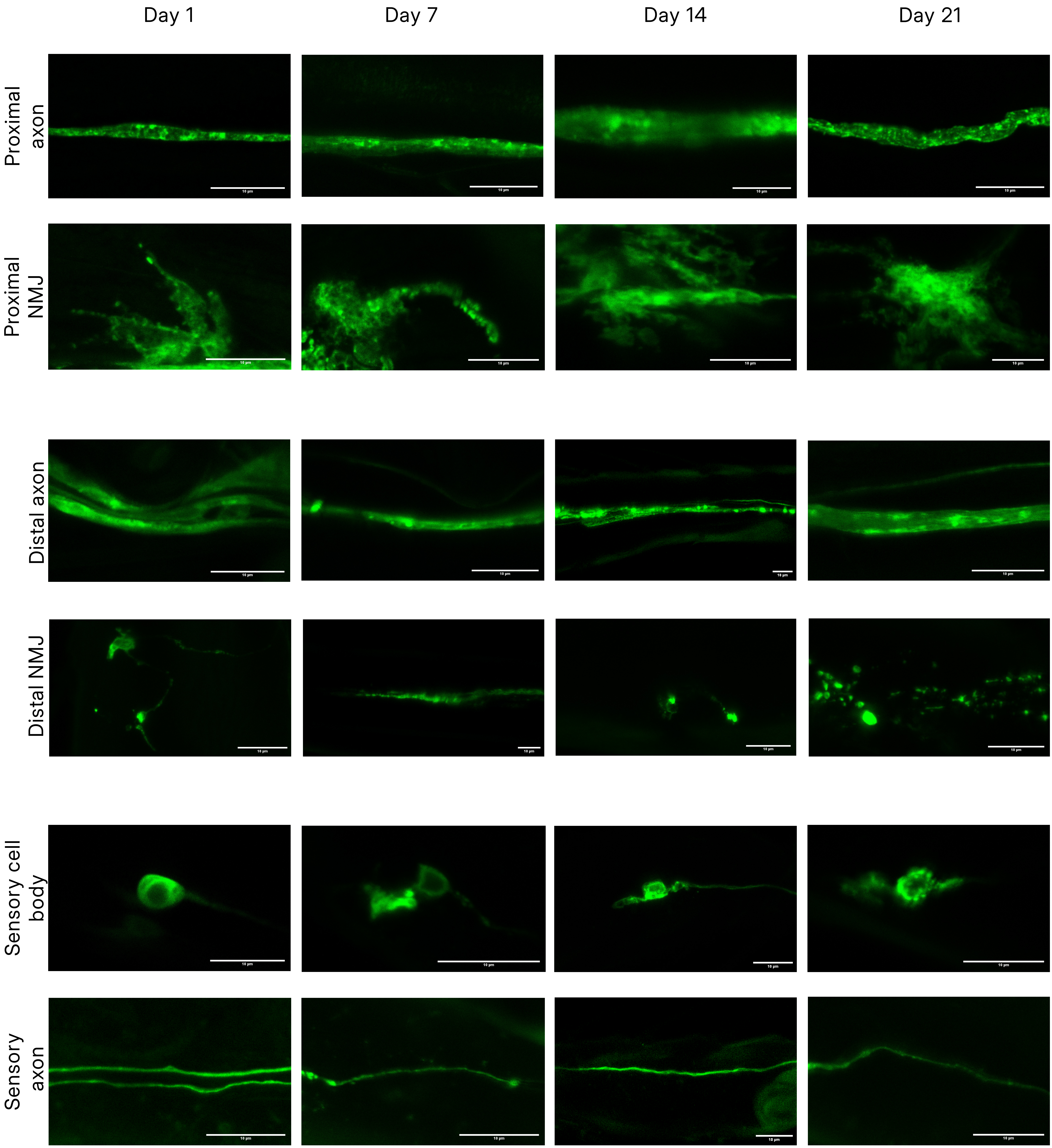

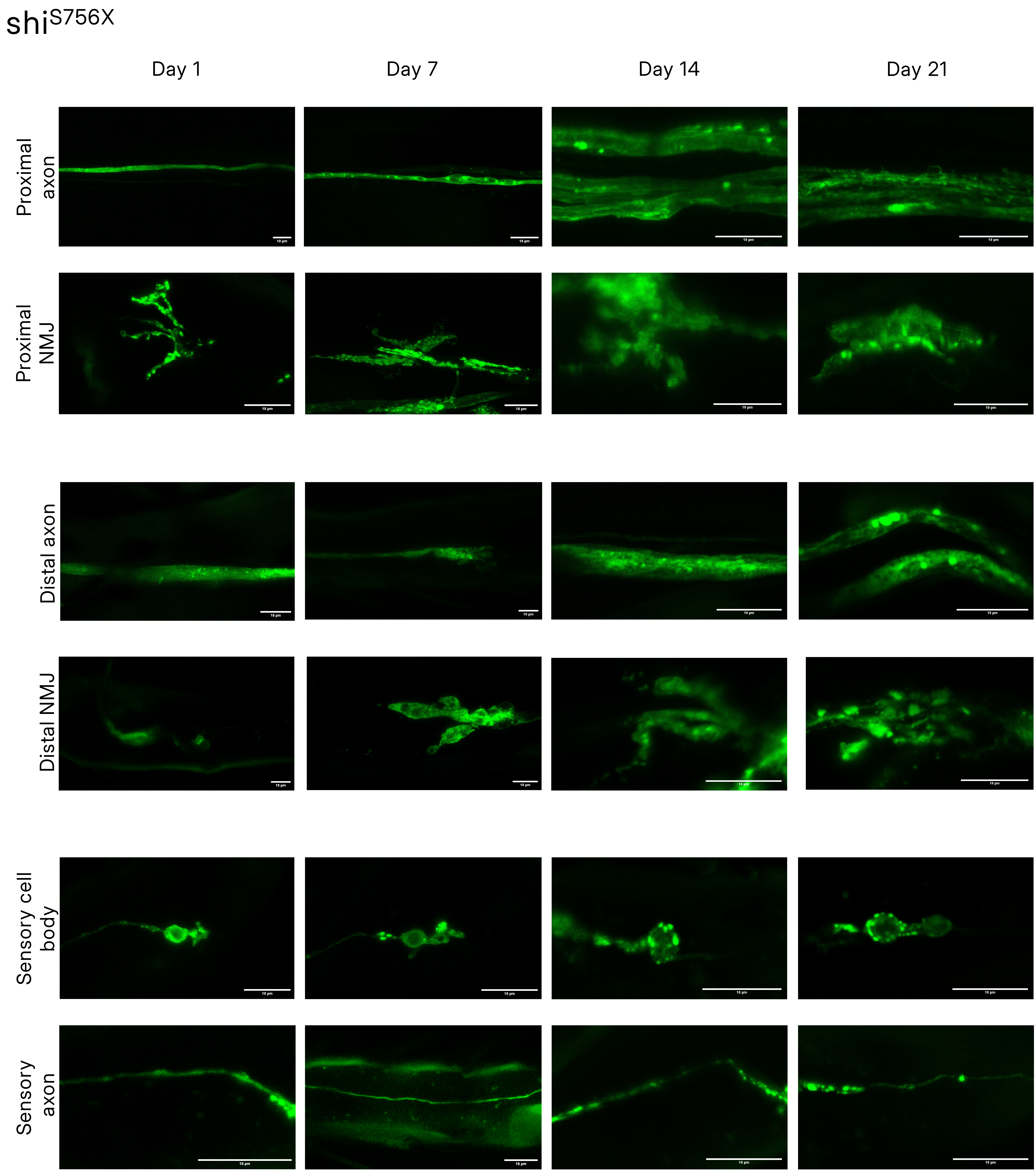
shi^{S756X}
Day 1
Day 7
Day 14
Day 21
Proximal axon
Proximal NMJ
Distal axon
Distal NMJ
Sensory cell body
Sensory axon

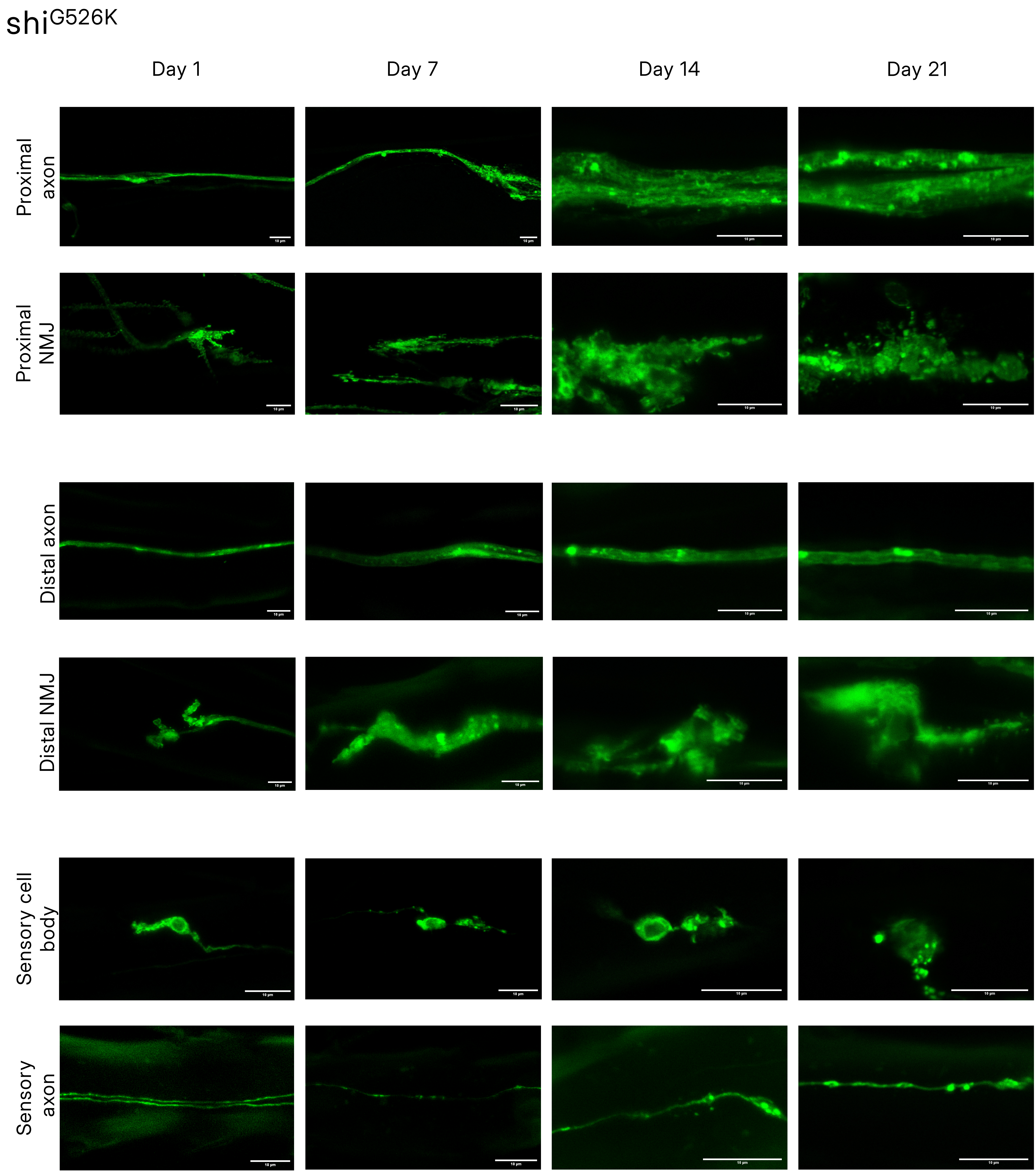
shi^G526K
Day 1
Day 7
Day 14
Day 21
Proximal axon
Proximal NMJ
Distal axon
Distal NMJ
Sensory cell body
Sensory axon

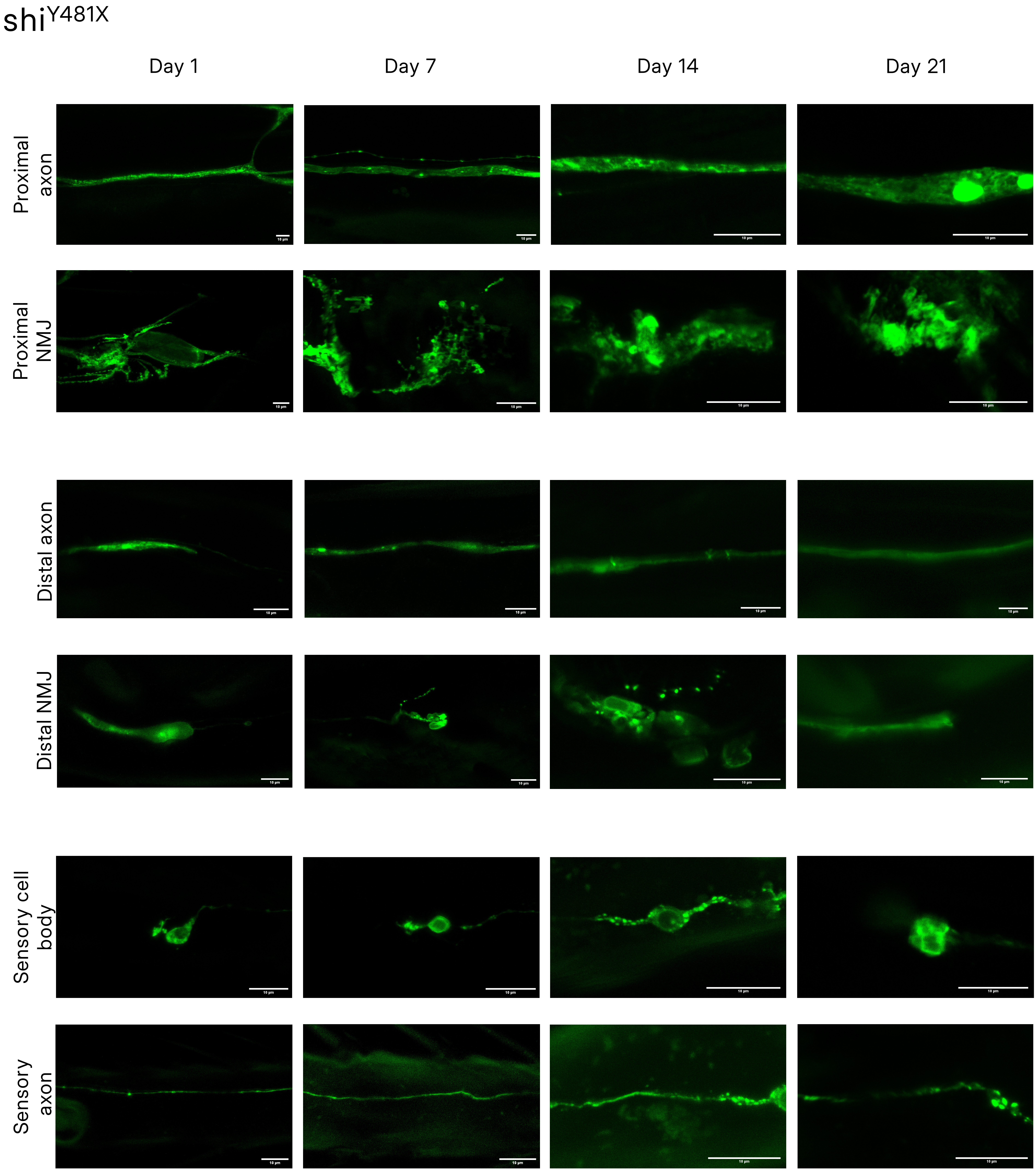

shi^{Y481X}
Day 1
Day 7
Day 14
Day 21
Proximal axon
Proximal NMJ
Distal axon
Distal NMJ
Sensory cell body
Sensory axon

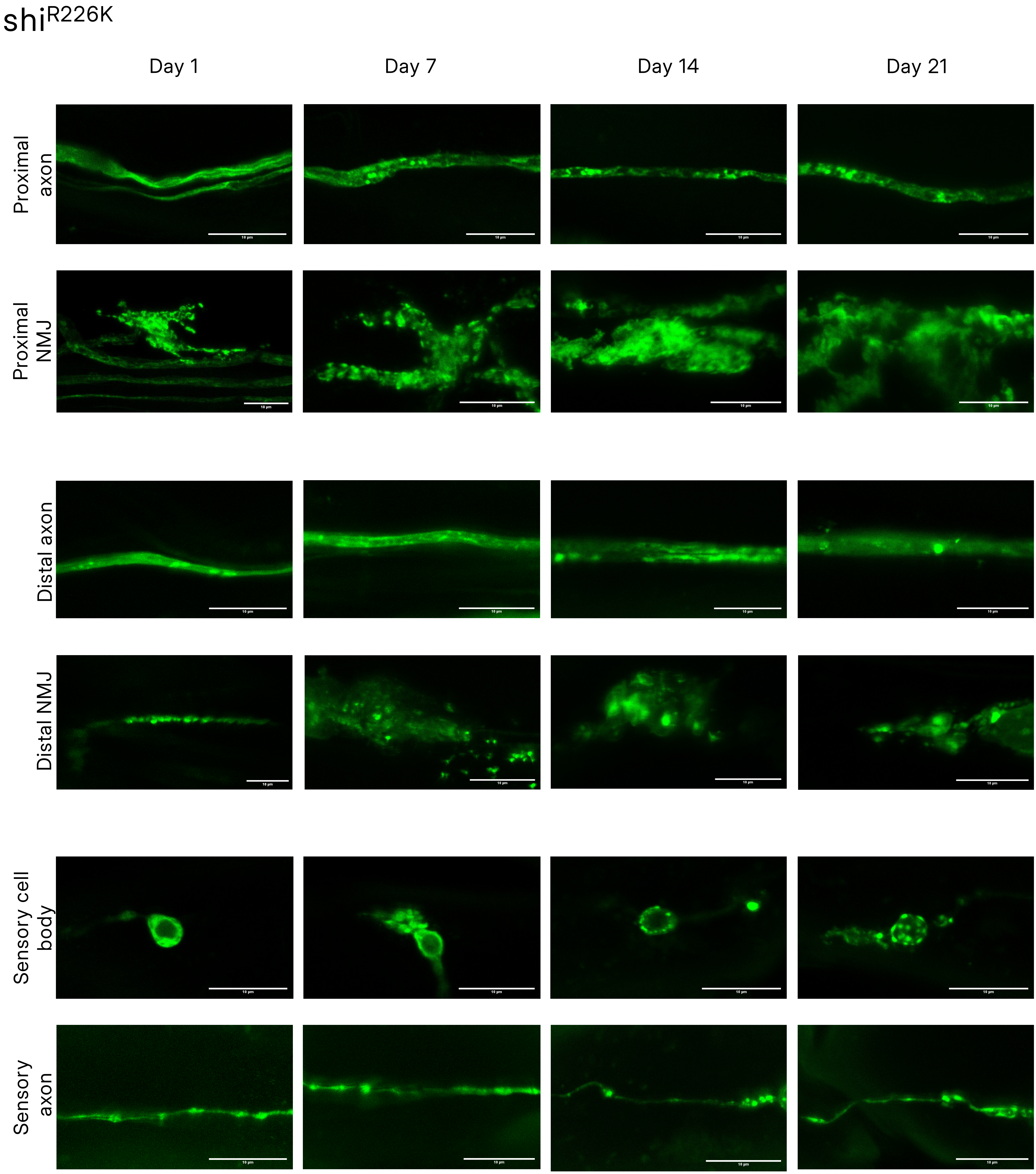
shi^R226K
Day 1
Day 7
Day 14
Day 21
Proximal axon
Proximal NMJ
Distal axon
Distal NMJ
Sensory cell body
Sensory axon

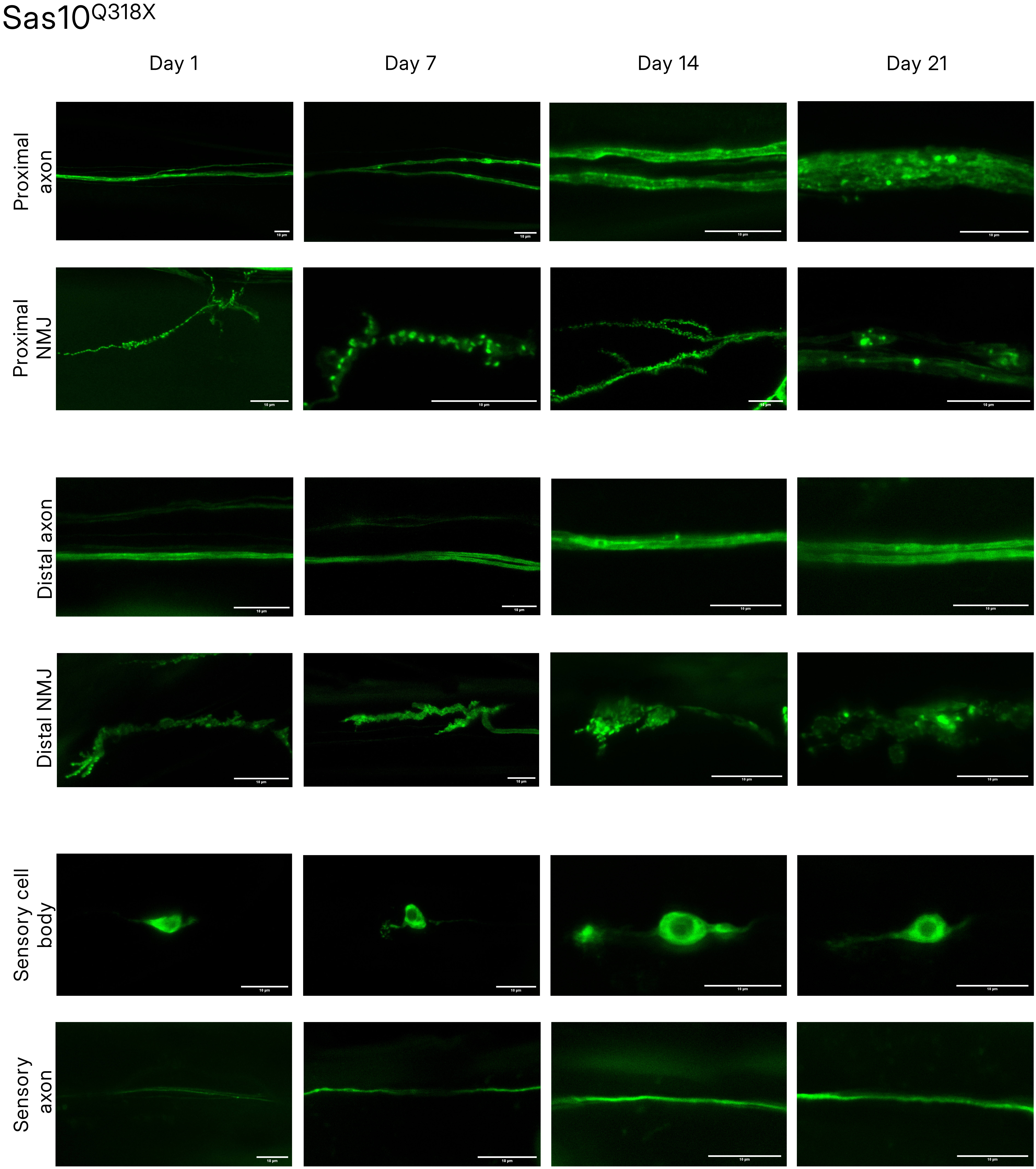
Sas10Q318X
Day 1
Day 7
Day 14
Day 21
Proximal axon
Proximal NMJ
Distal axon
Distal NMJ
Sensory cell body
Sensory axon

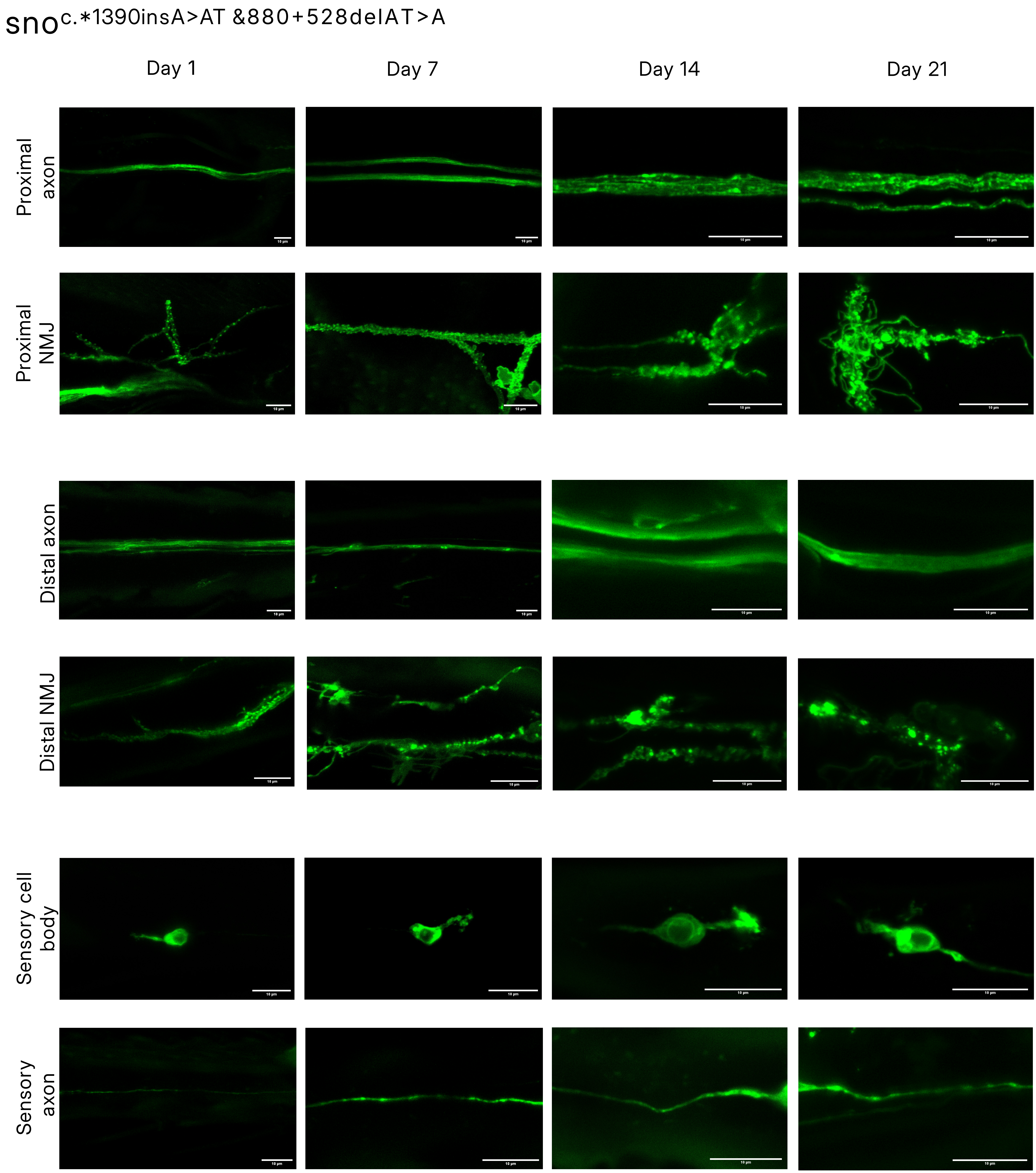
sno c.*1390insA>AT &880+528delAT>A
Day 1
Day 7
Day 14
Day 21
Proximal axon
Proximal NMJ
Distal axon
Distal NMJ
Sensory cell body
Sensory axon

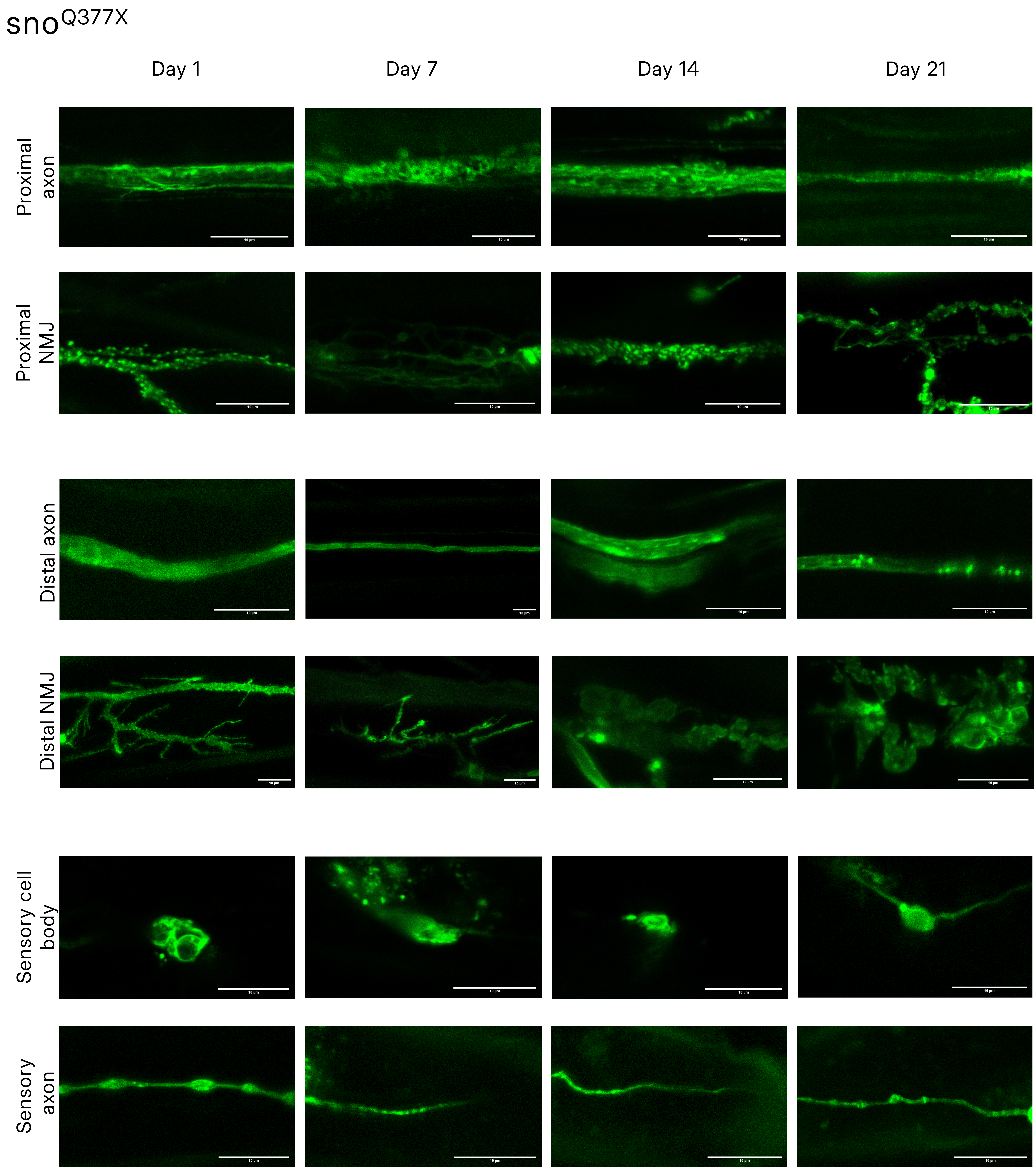

sno^{Q377X}
Day 1
Day 7
Day 14
Day 21
Proximal axon
Proximal NMJ
Distal axon
Distal NMJ
Sensory cell body
Sensory axon

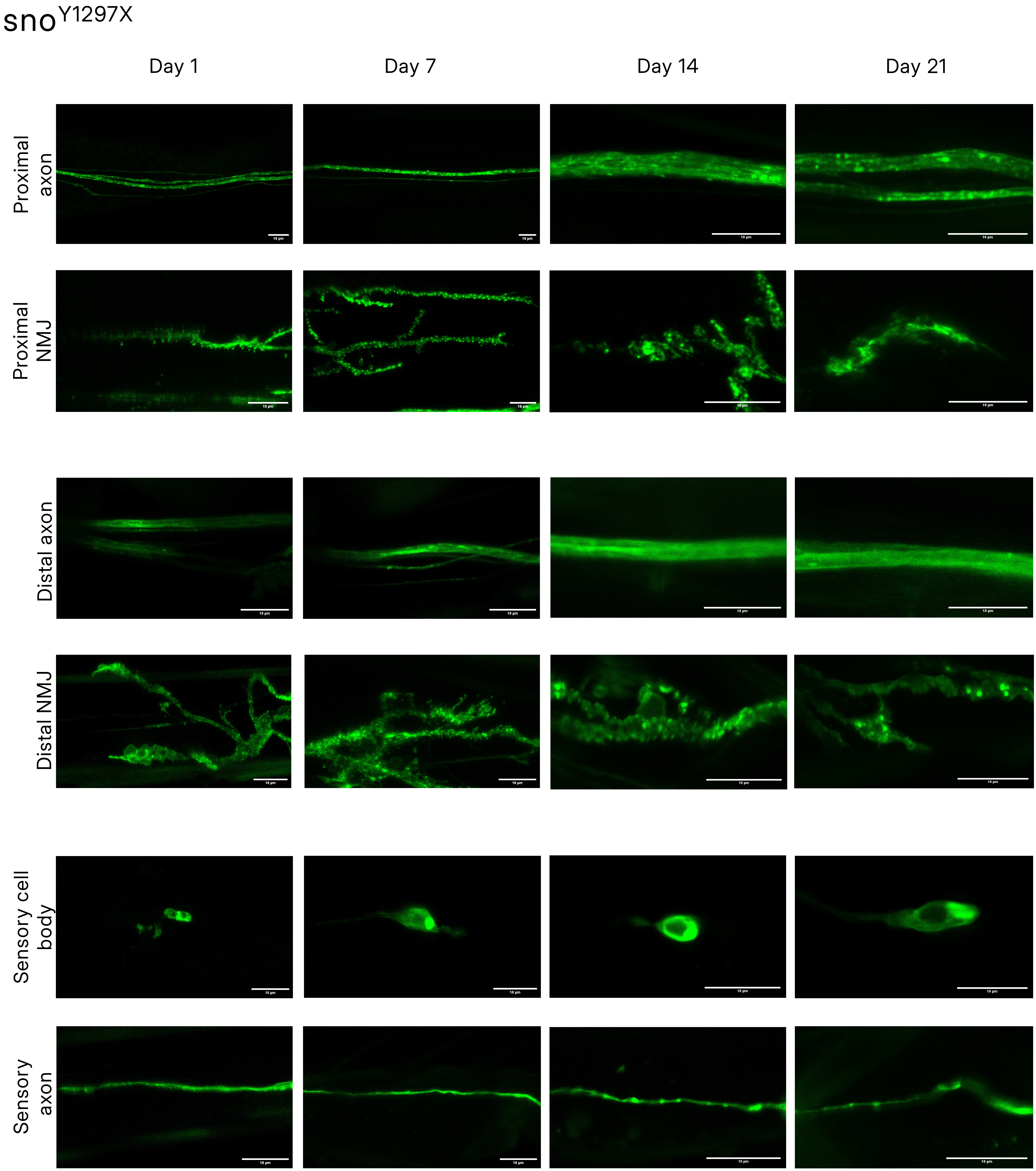
sno^{Y1297X}
Day 1
Day 7
Day 14
Day 21
Proximal axon
Proximal NMJ
Distal axon
Distal NMJ
Sensory cell body
Sensory axon

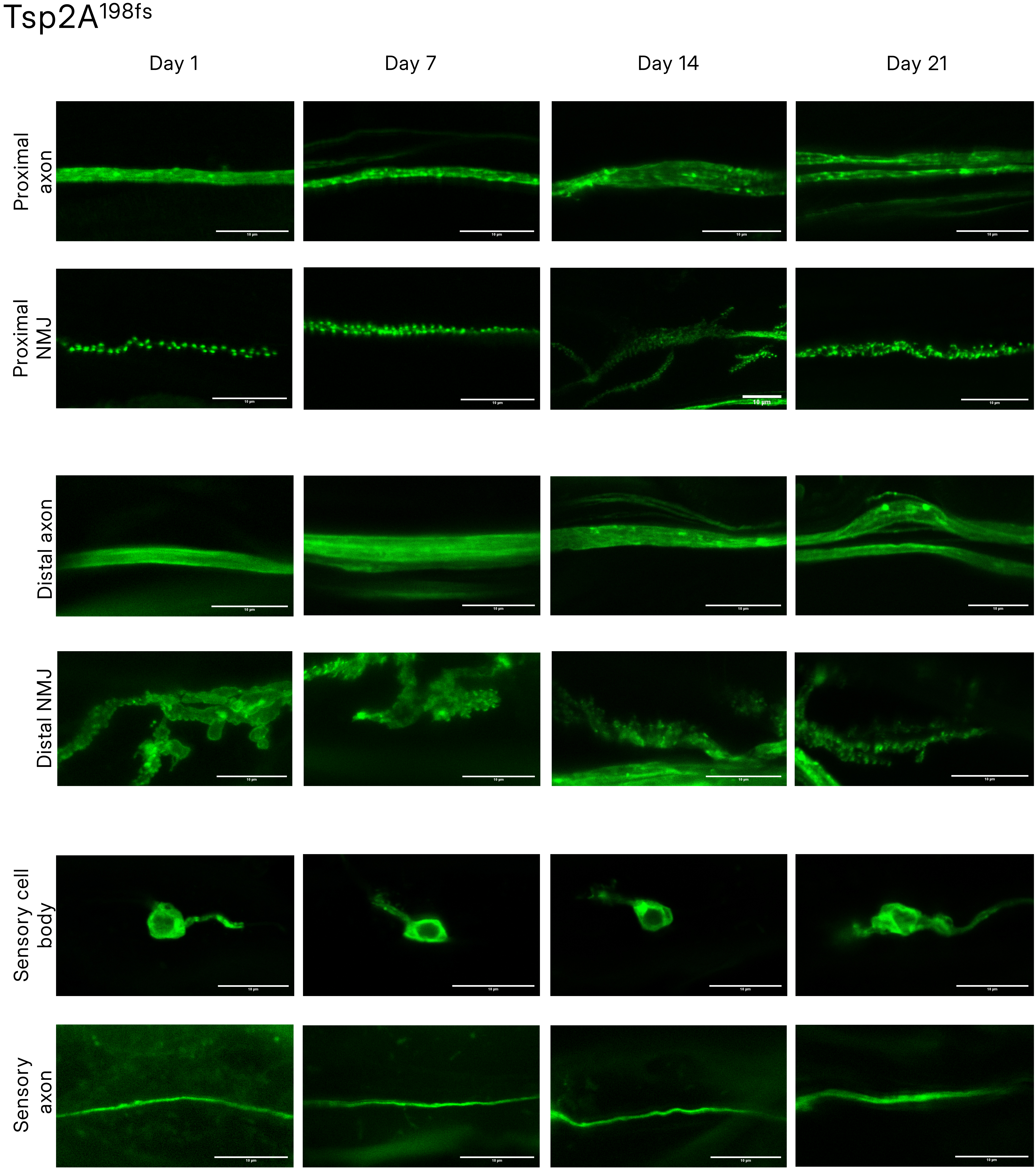

Tsp2A198fs
Day 1
Day 7
Day 14
Day 21
Proximal axon
Proximal NMJ
Distal axon
Distal NMJ
Sensory cell body
Sensory axon
10 µm

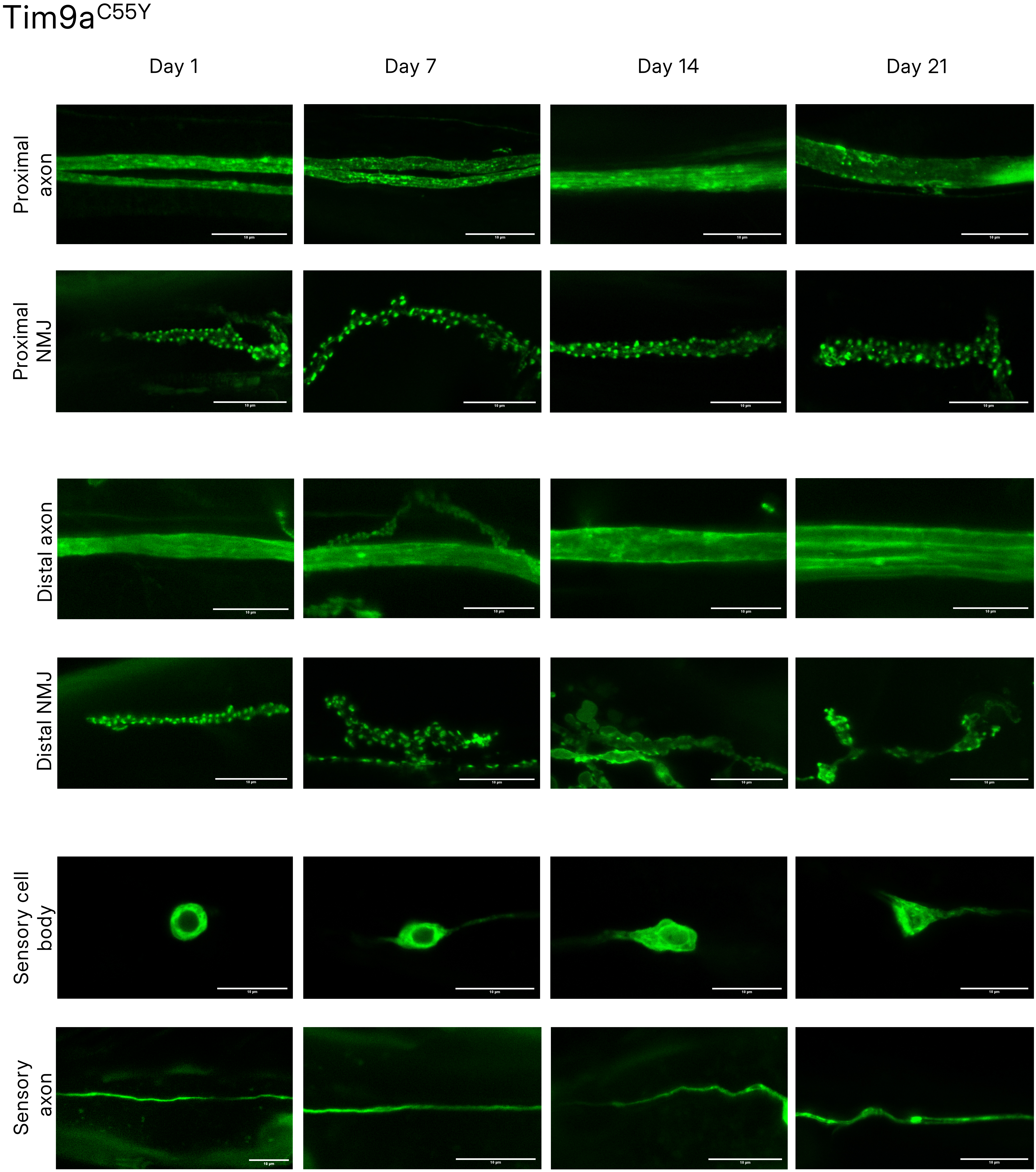

Tim9a^{C55Y}
Day 1
Day 7
Day 14
Day 21
Proximal axon
Proximal NMJ
Distal axon
Distal NMJ
Sensory cell body
Sensory axon

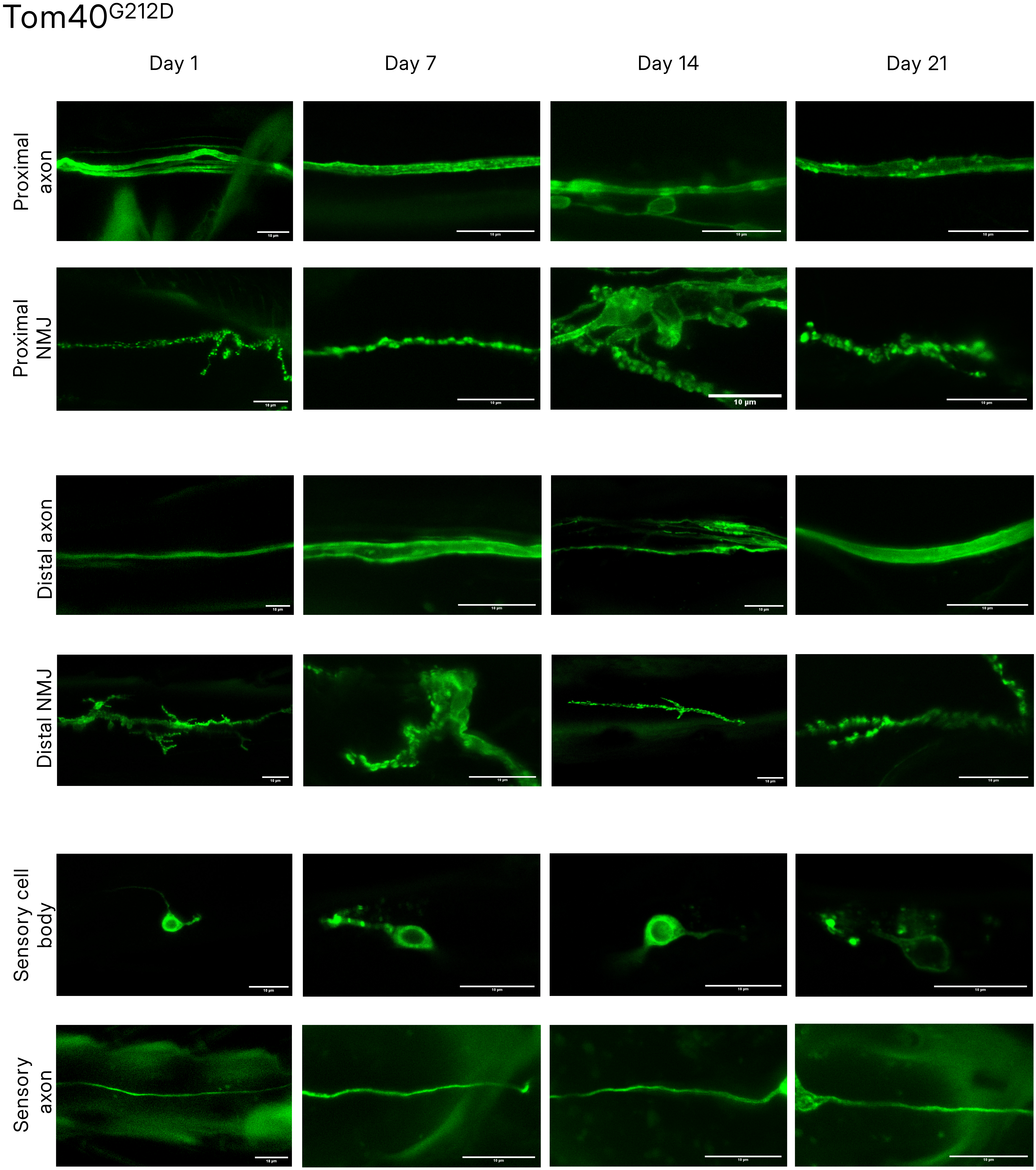
Tom40G212D
Day 1
Day 7
Day 14
Day 21
Proximal axon
Proximal NMJ
Distal axon
Distal NMJ
Sensory cell body
Sensory axon
10 μm

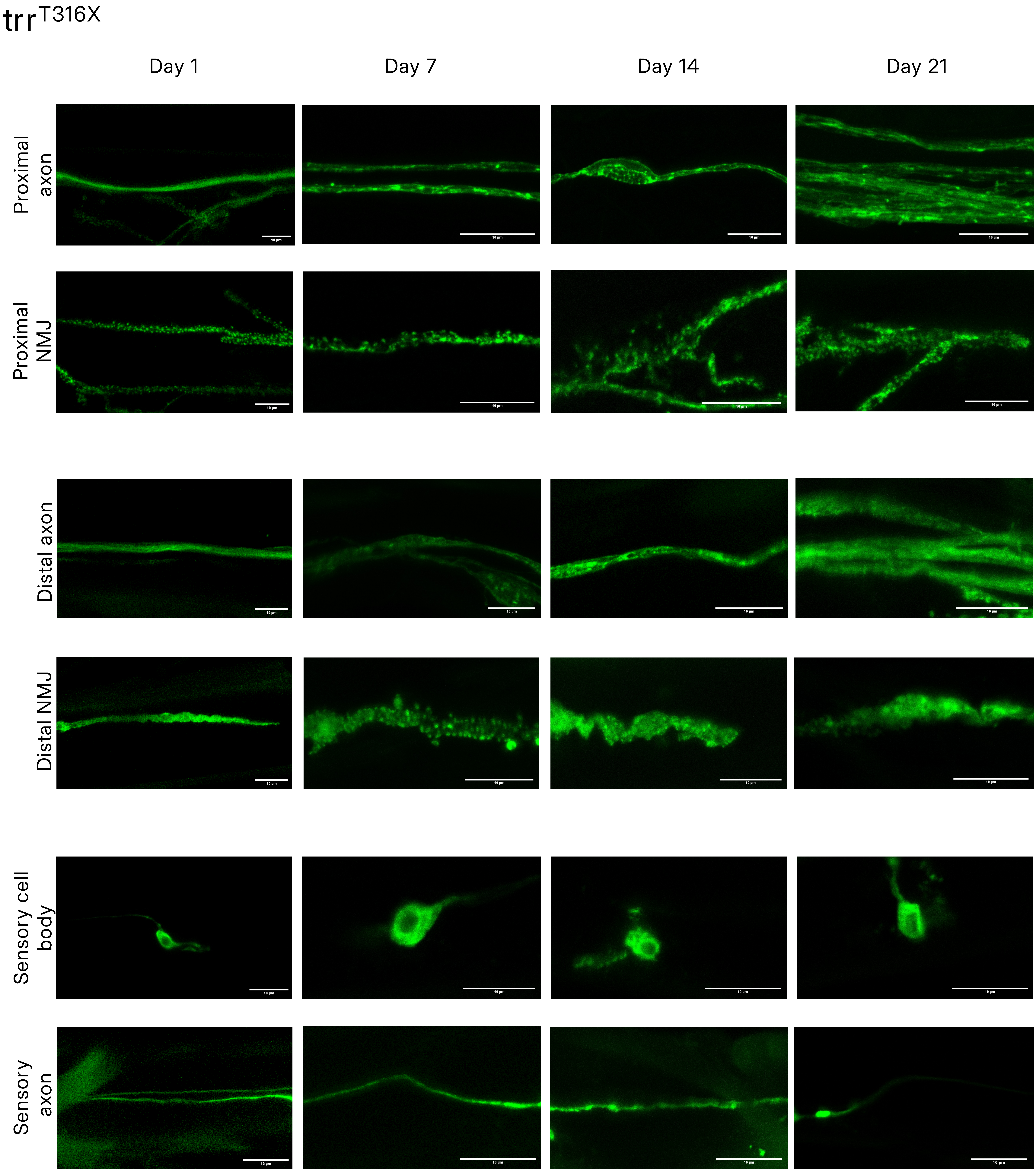
trr^{T316X}
Day 1
Day 7
Day 14
Day 21
Proximal axon
Proximal NMJ
Distal axon
Distal NMJ
Sensory cell body
Sensory axon

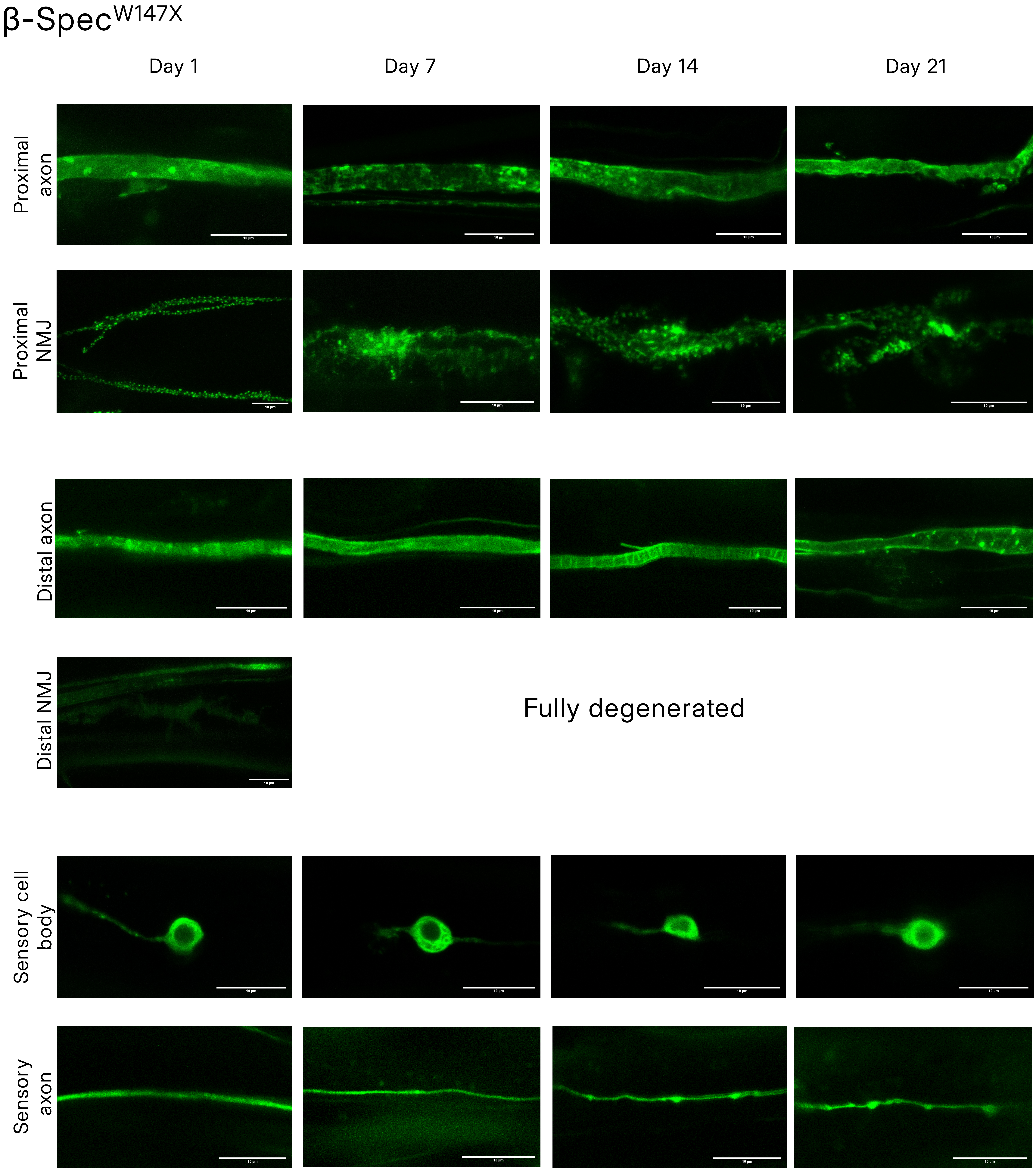
β-Spec^{W147X}
Day 1
Day 7
Day 14
Day 21
Proximal axon
Proximal NMJ
Distal axon
Distal NMJ
Fully degenerated
Sensory cell body
Sensory axon
