# Supplementary material for: Excessive activation of the RAS/MAPK pathway triggers adult-onset motor axonal degeneration
Source: iScience. 2026 Sep 21;29(10):117507. doi: 10.1016/j.isci.2026.117507 (PMC13631674; doi:10.1016/j.isci.2026.117507)

Data S2: Original unedited images

Figure 1B

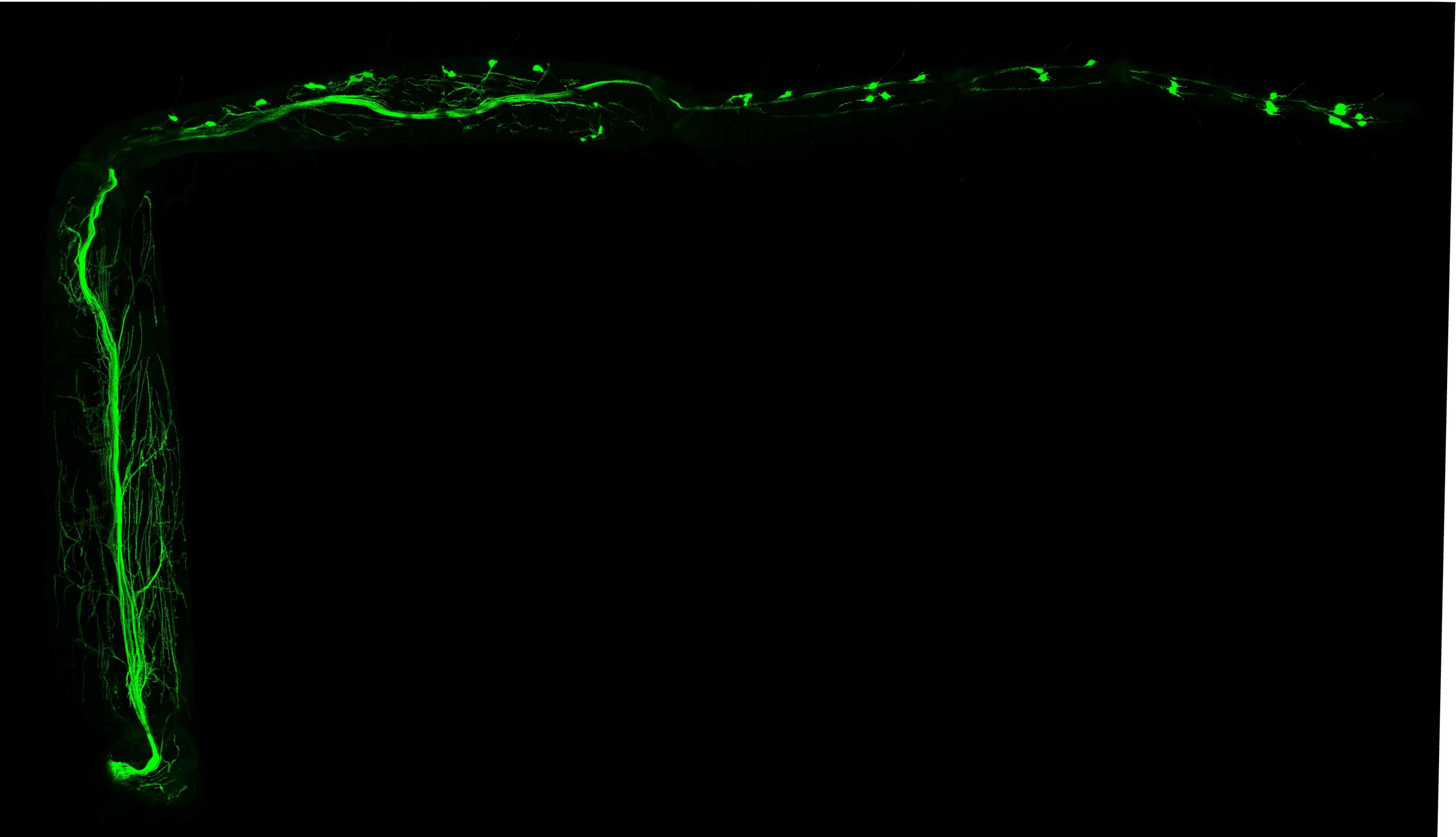

1C_1.tif

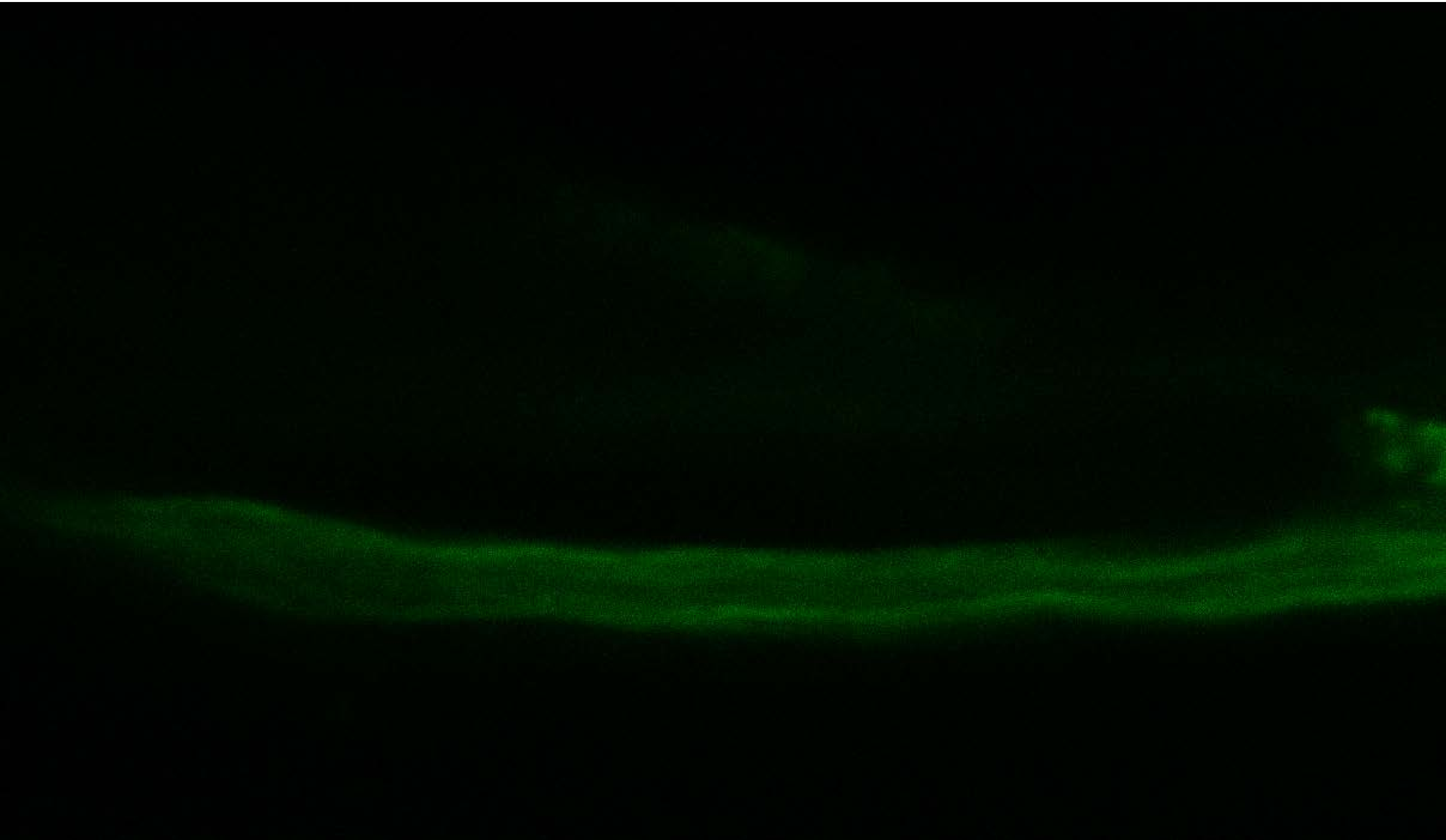

1C_2.tif

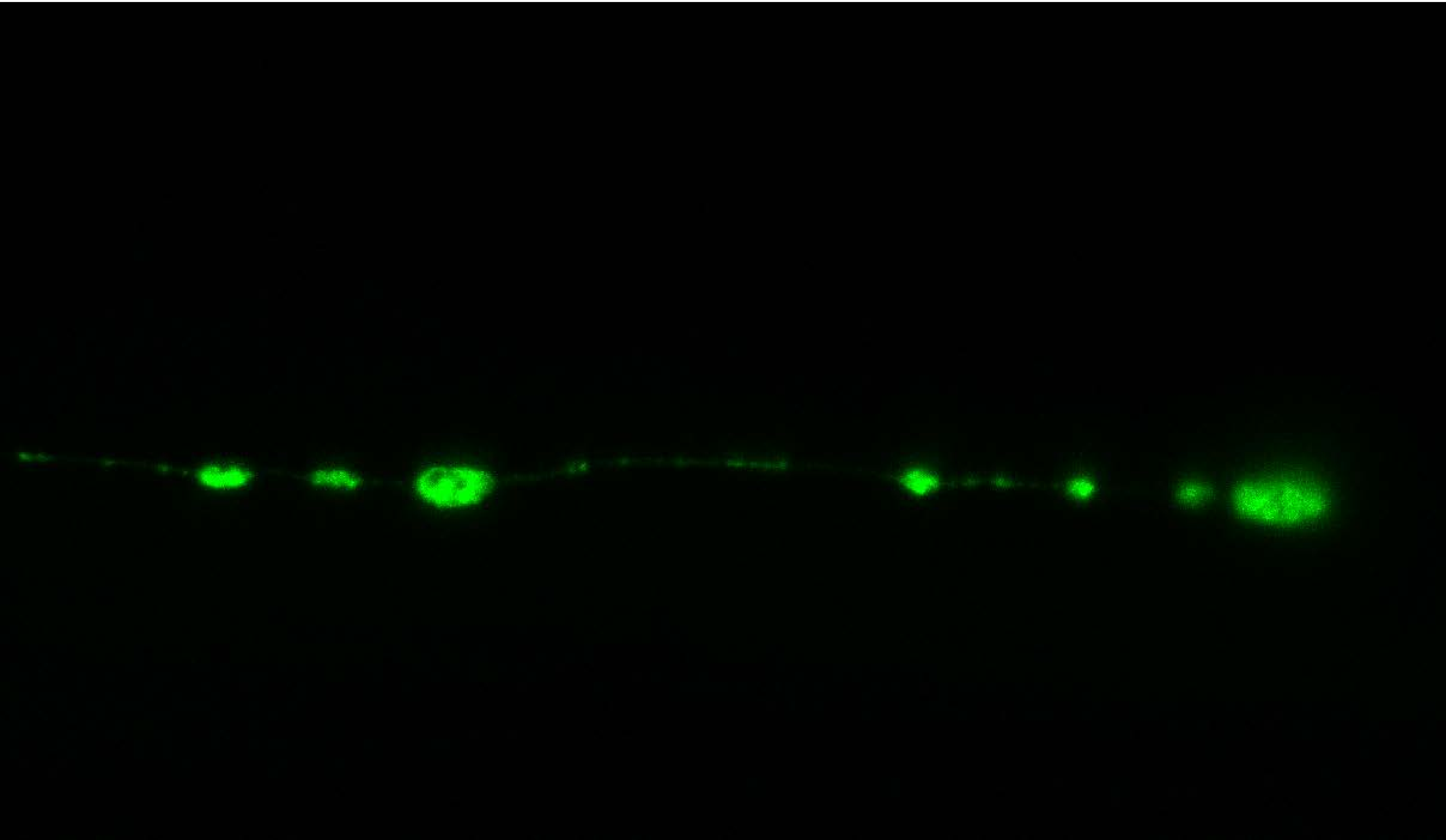

1C_3.tif

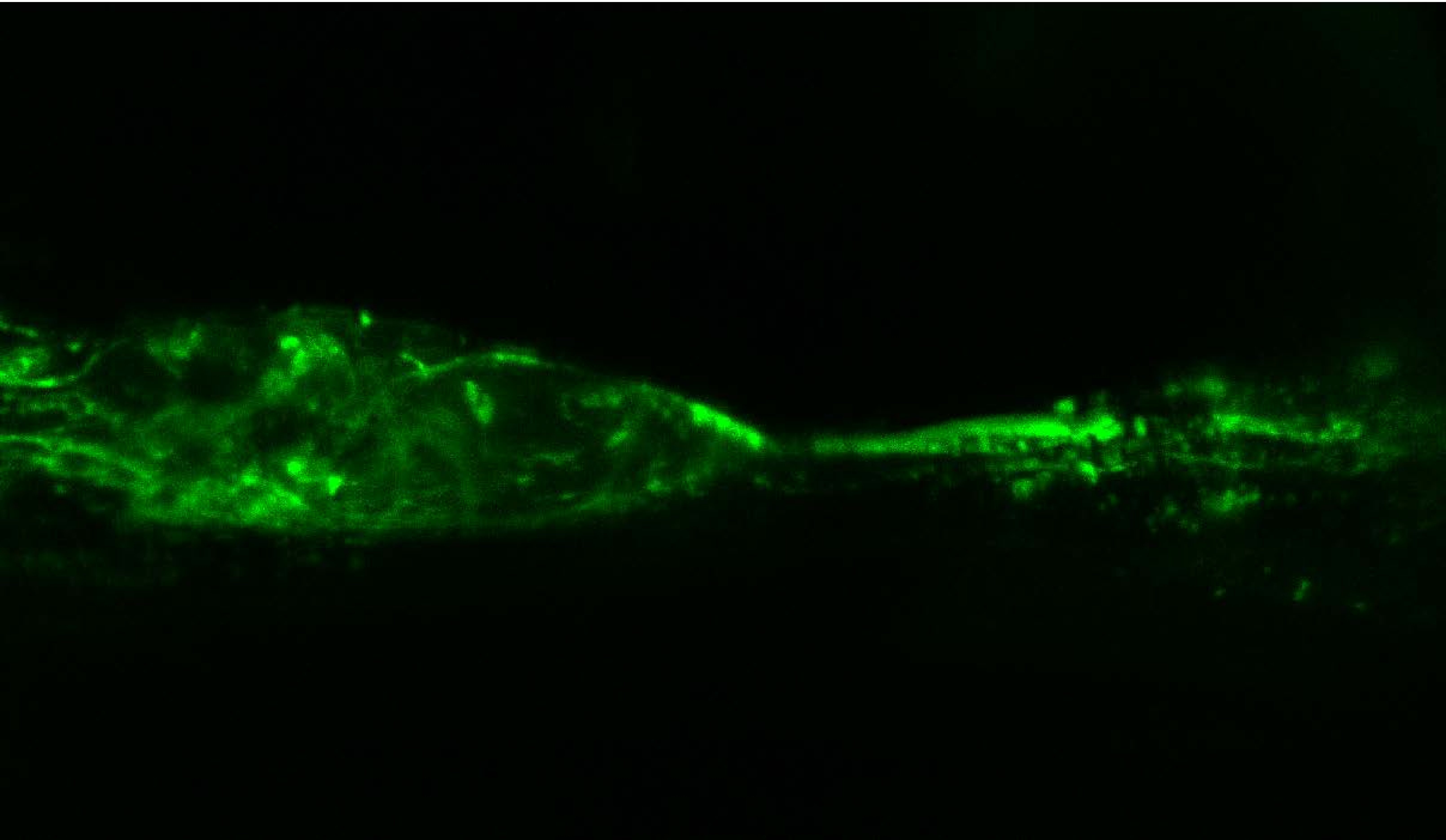

1C_4.tif

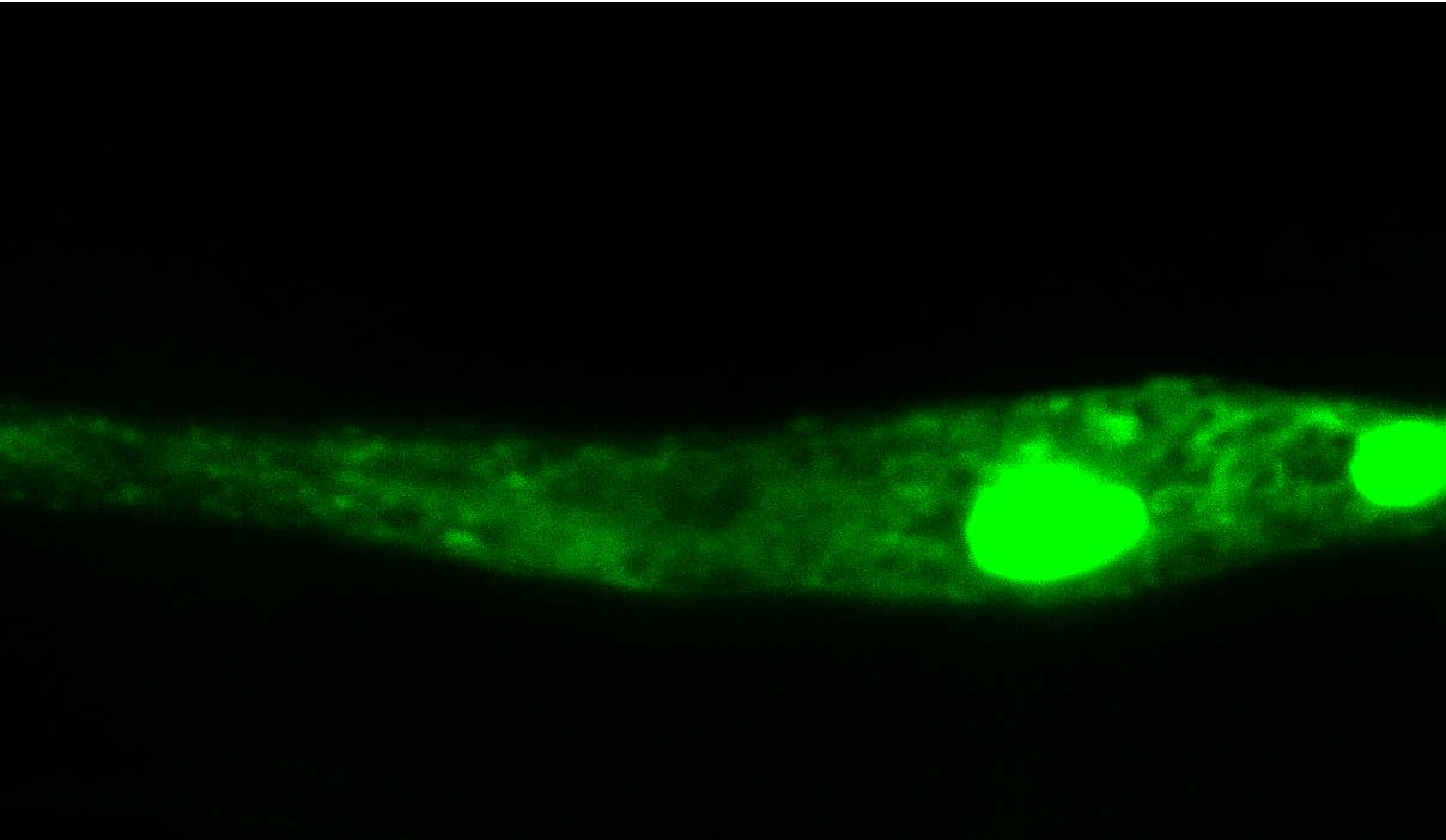

1C_5.tif

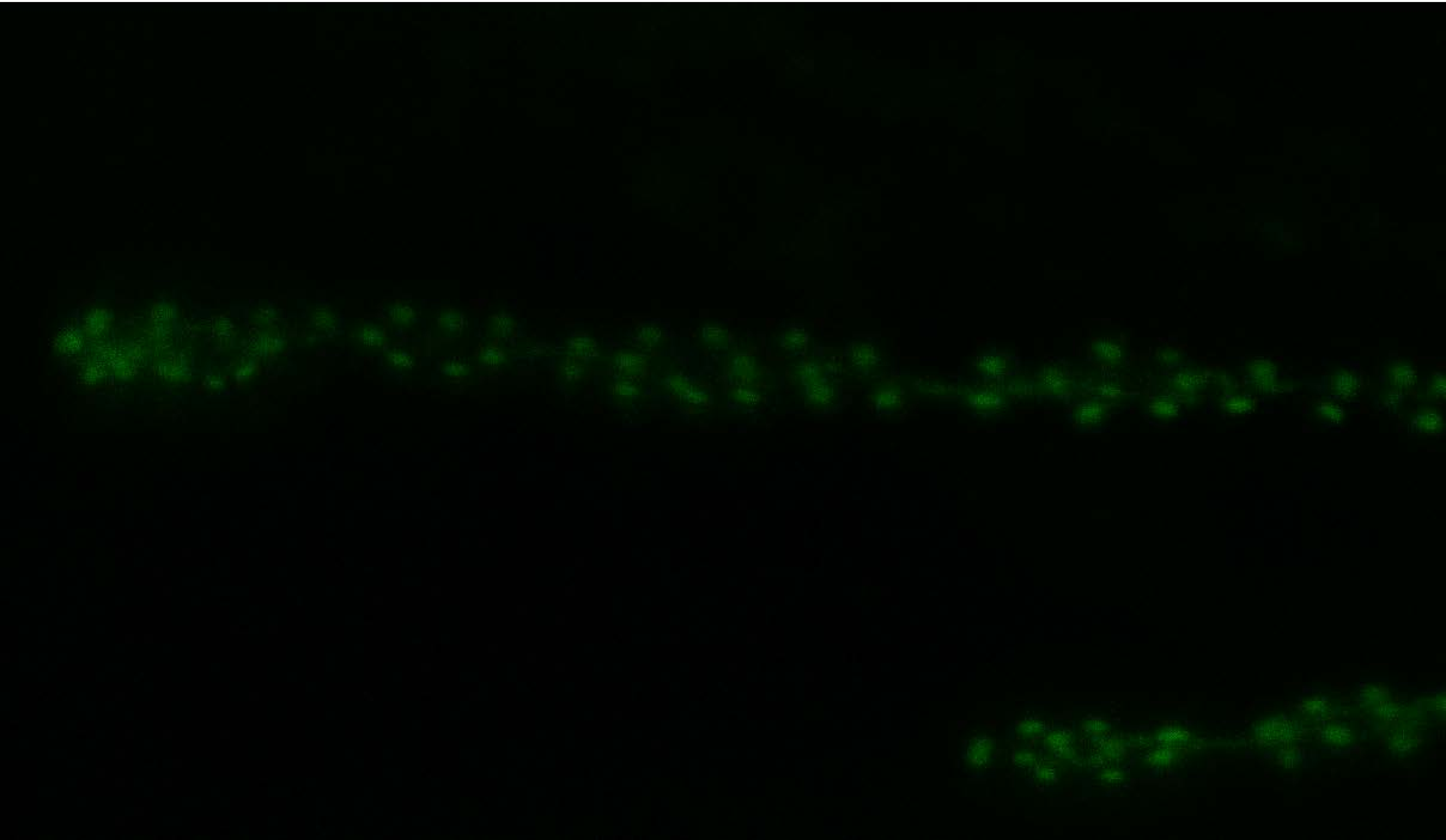

1C_6.tif

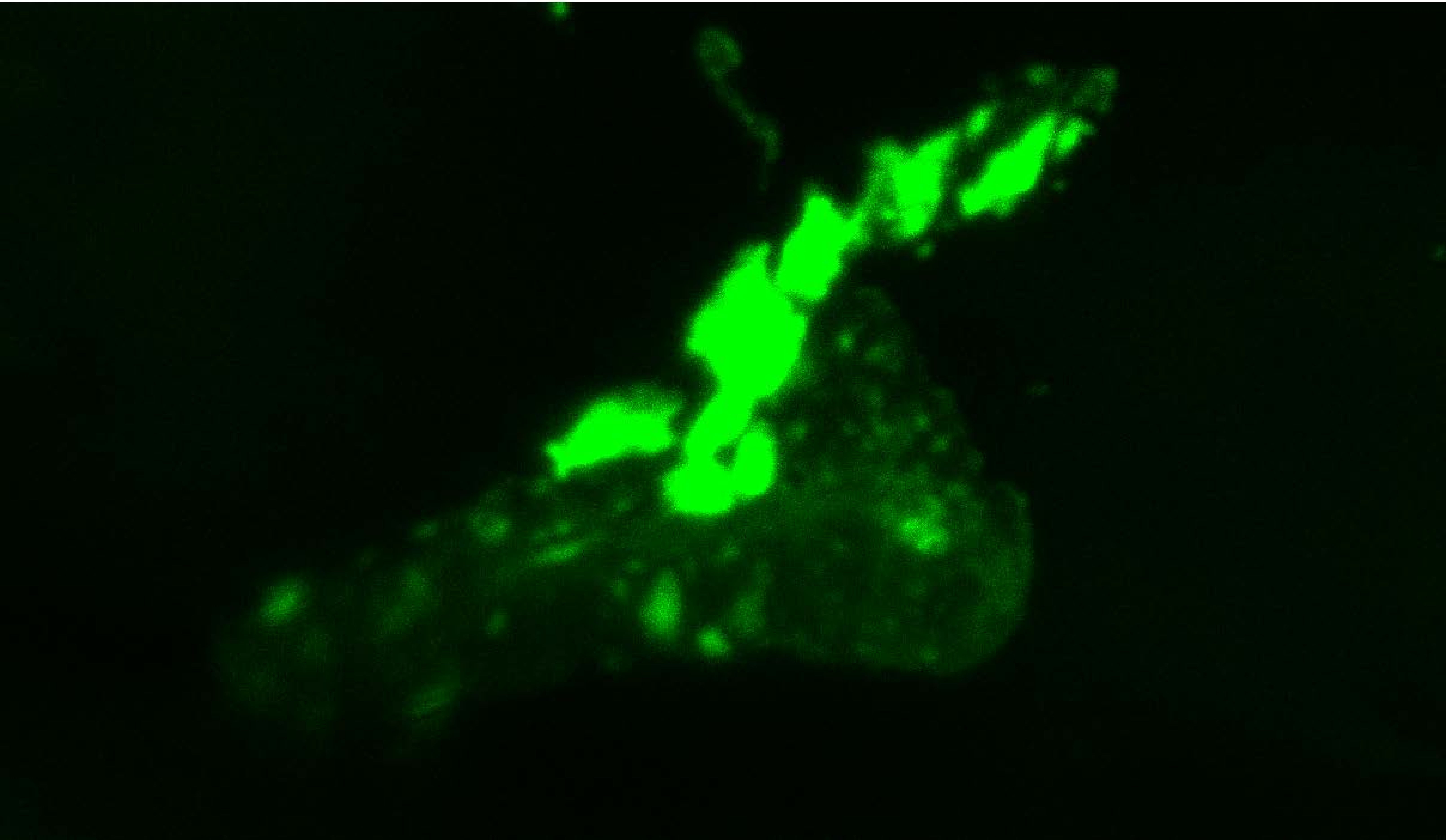

1C_7.tif

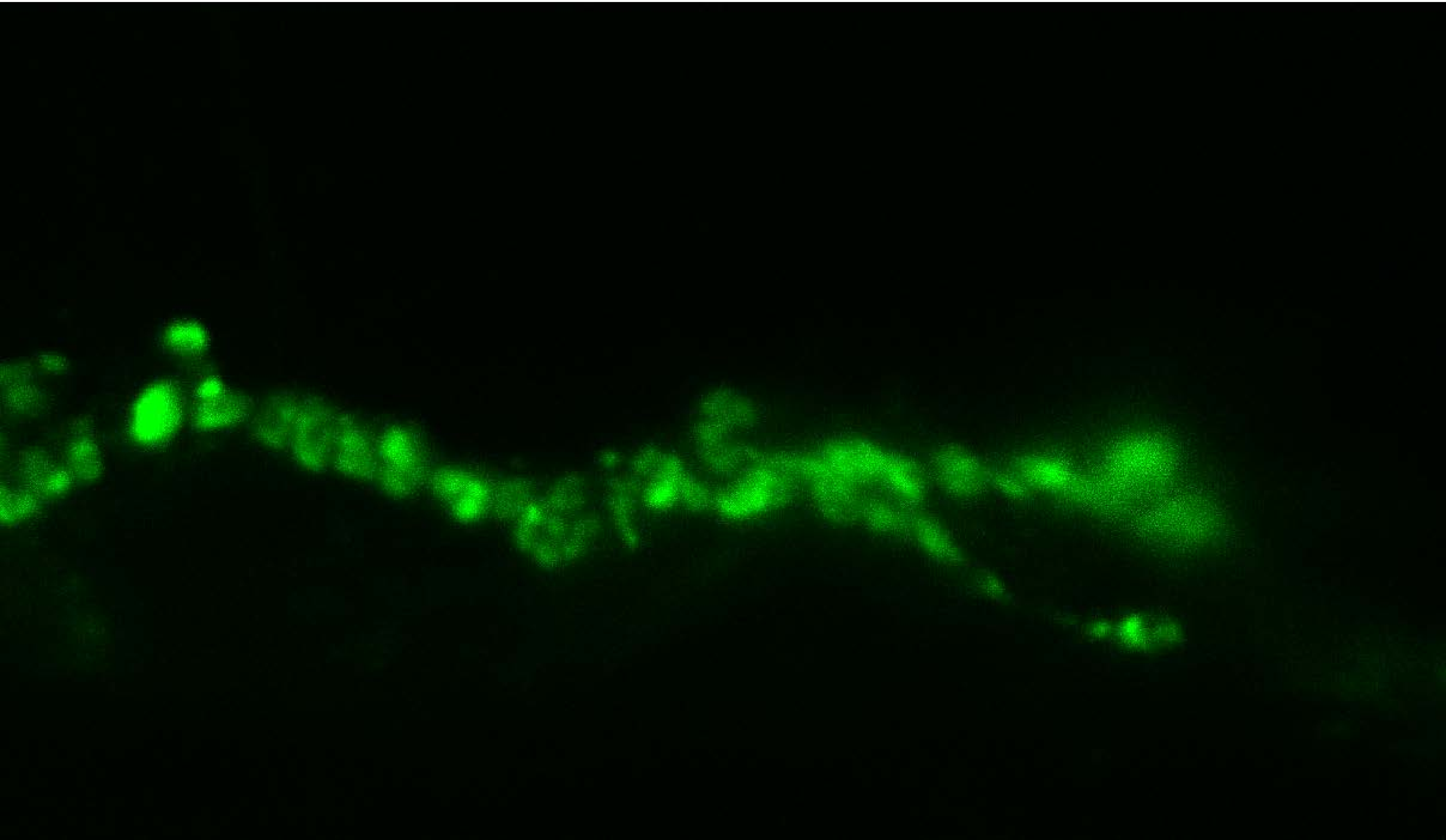

1C_8.tif

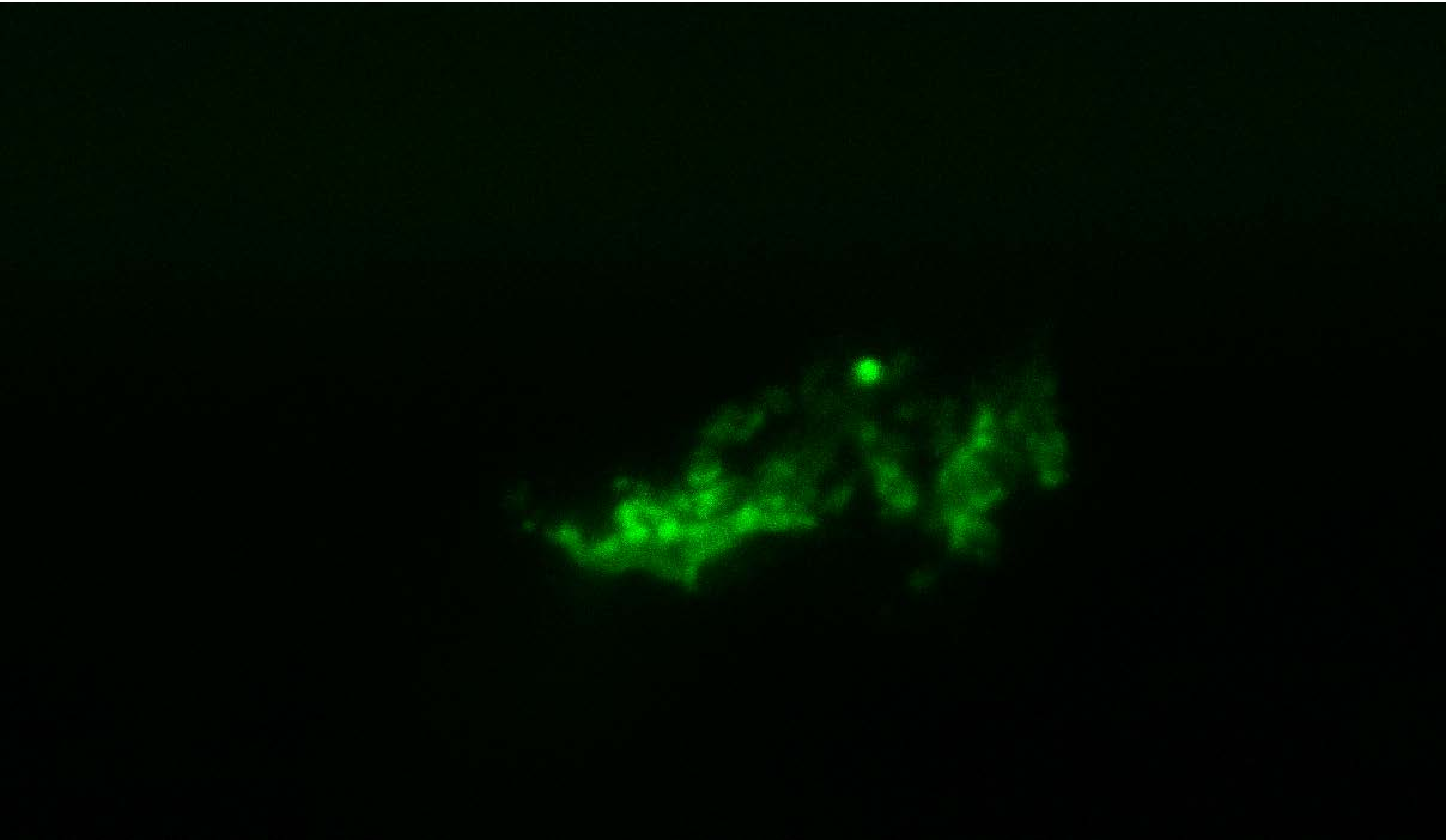

1D_1.tif

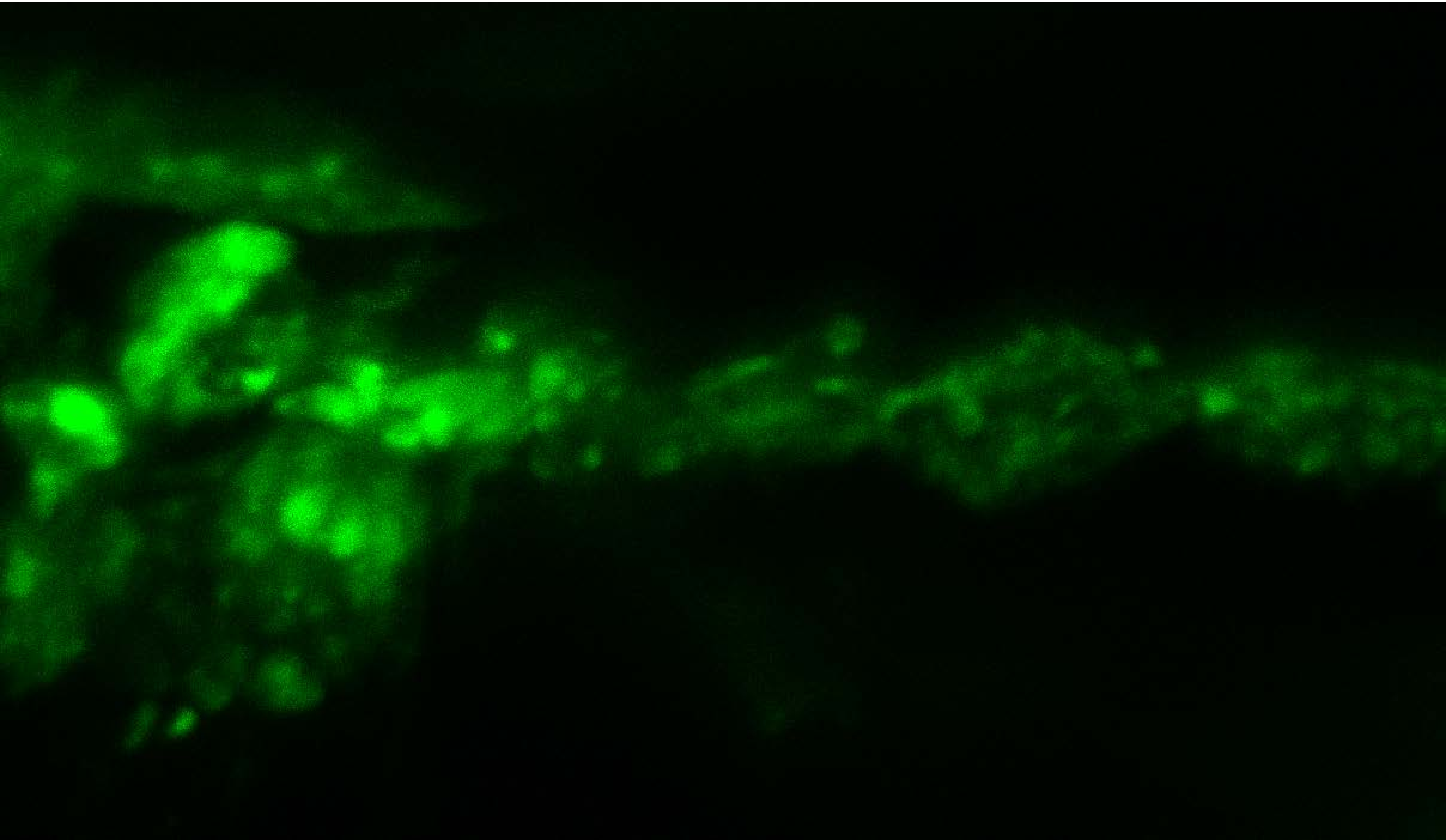

1D_2.tif

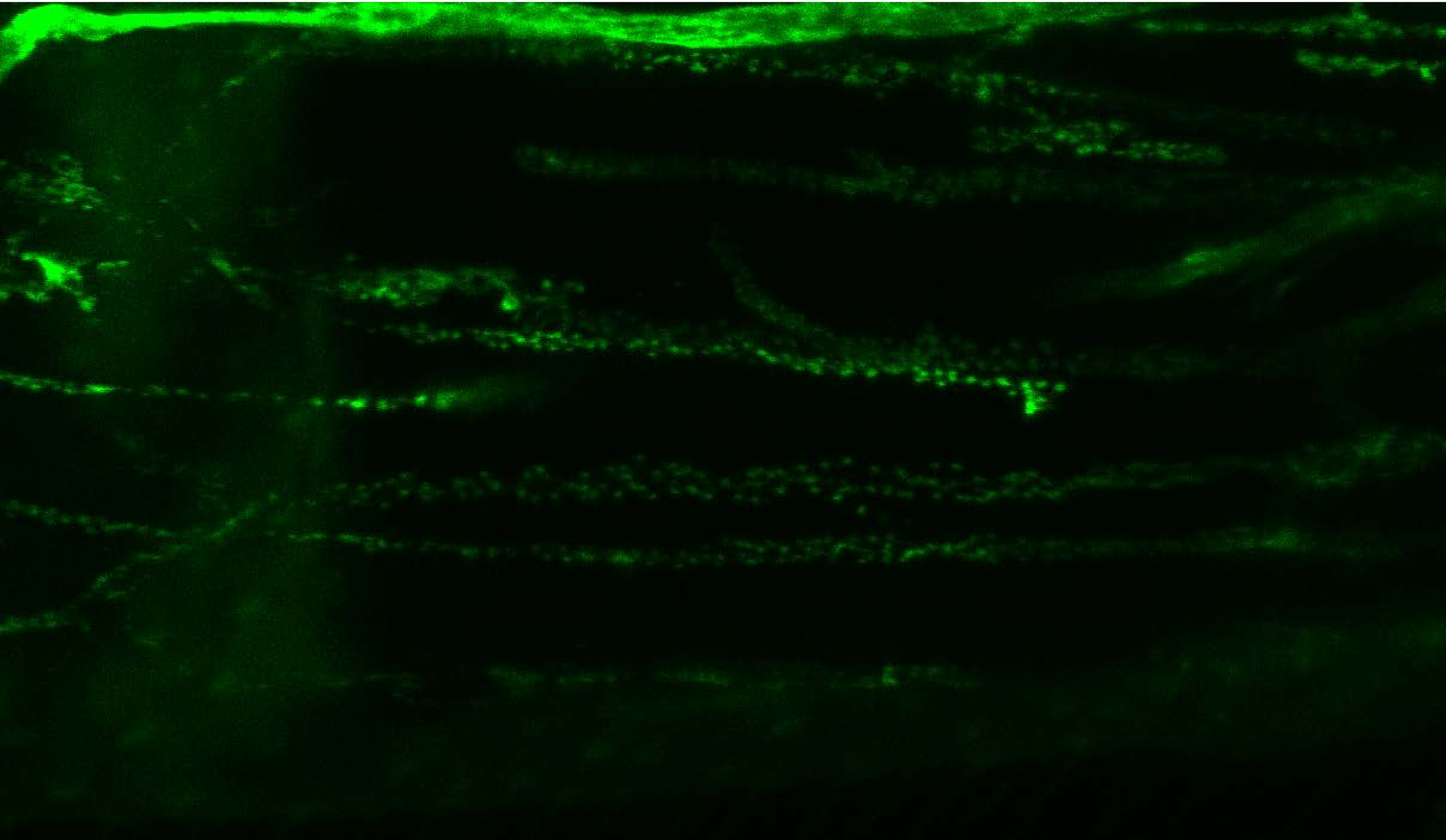

2A_1.tif

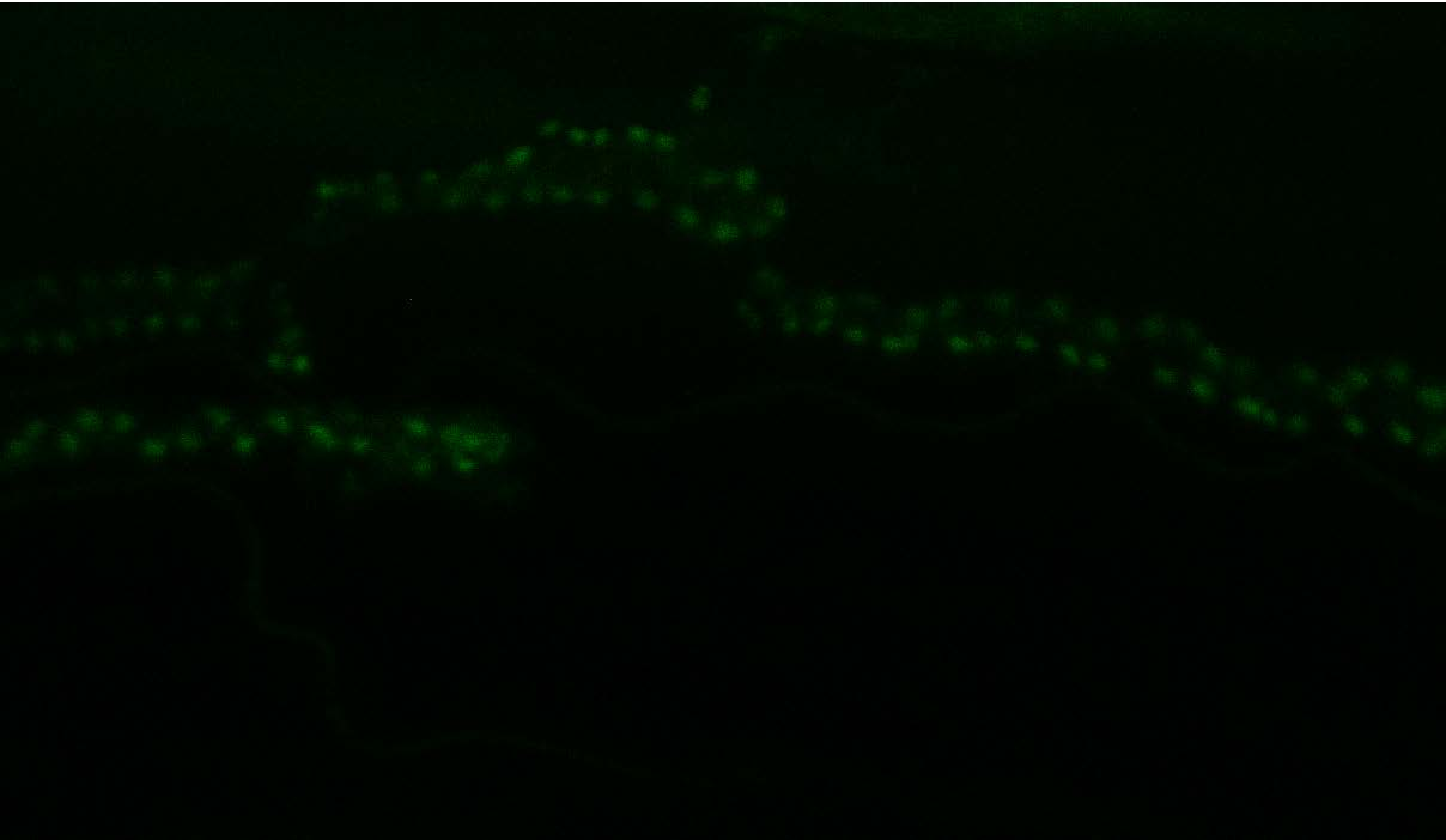

2A_2.tif

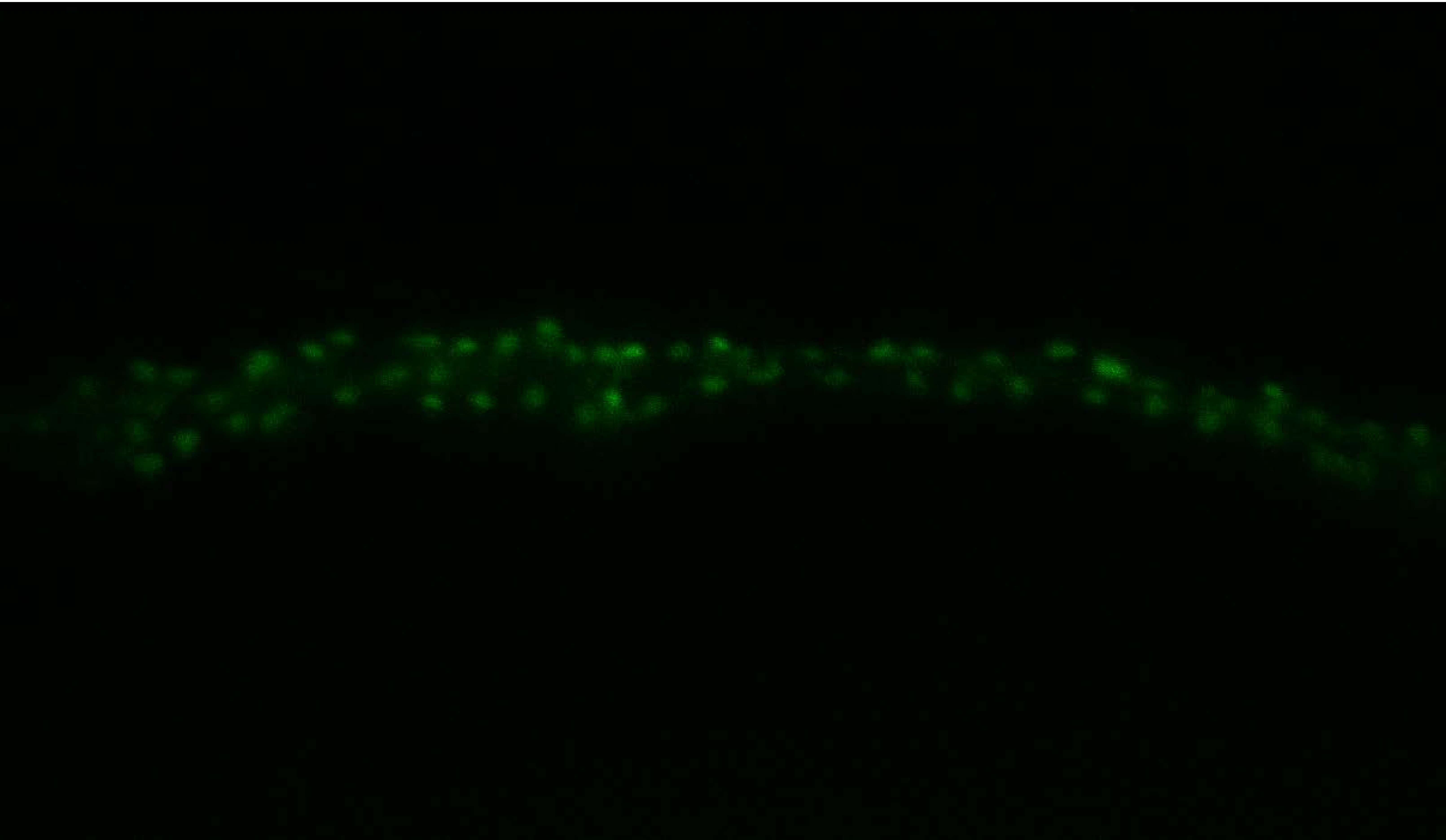

2A_3.tif

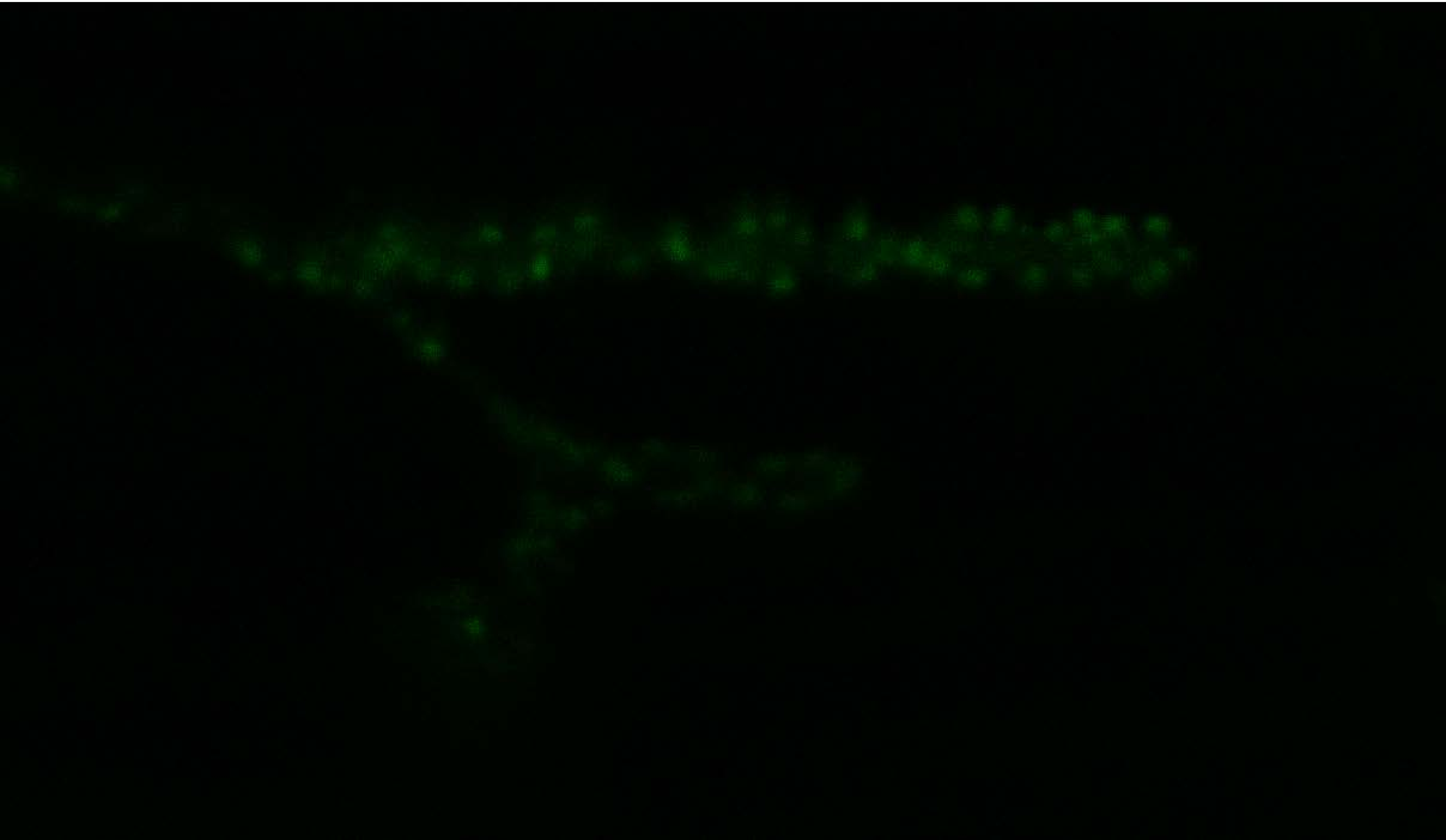

2A_4.tif

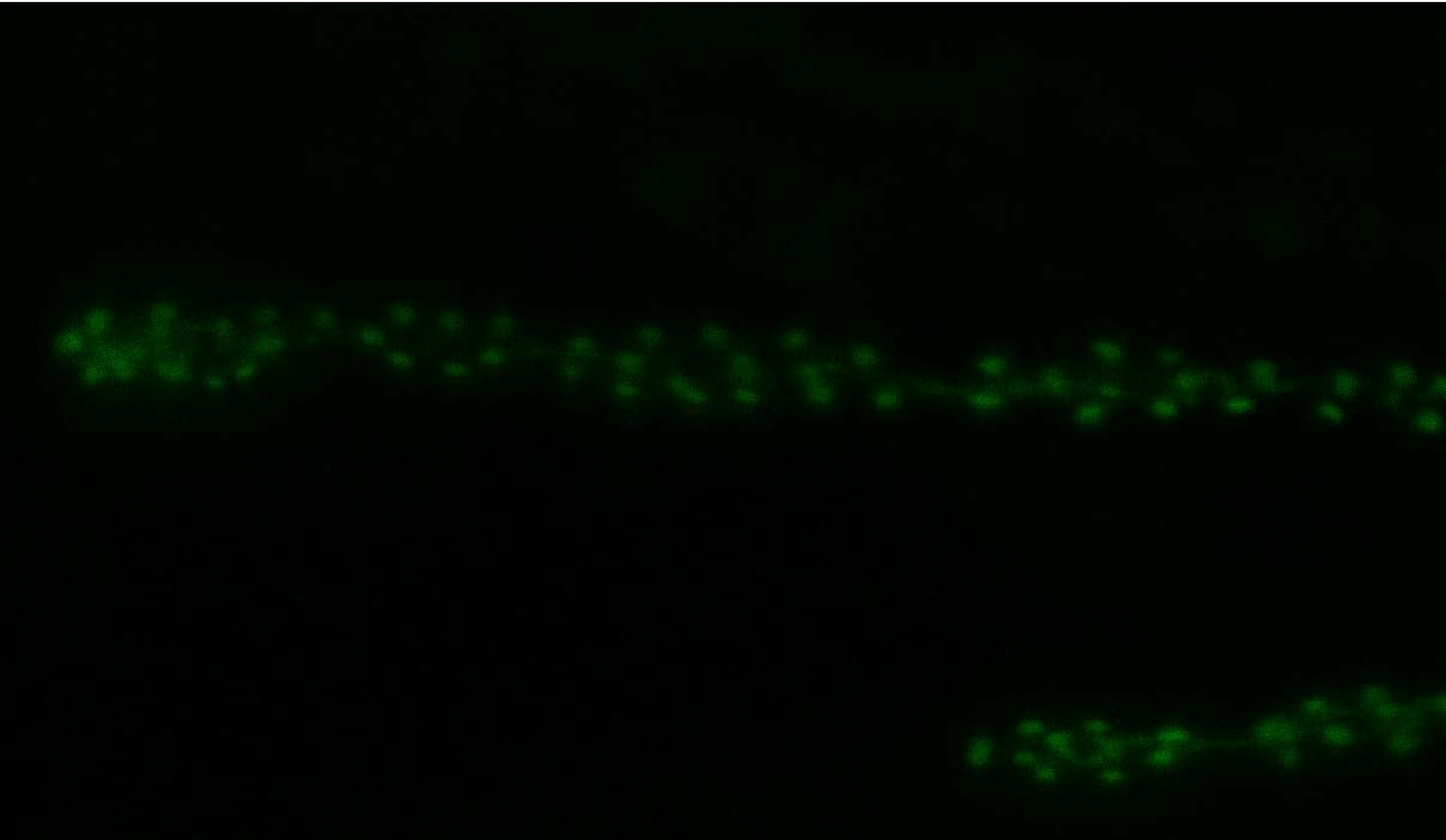

2A_5.tif

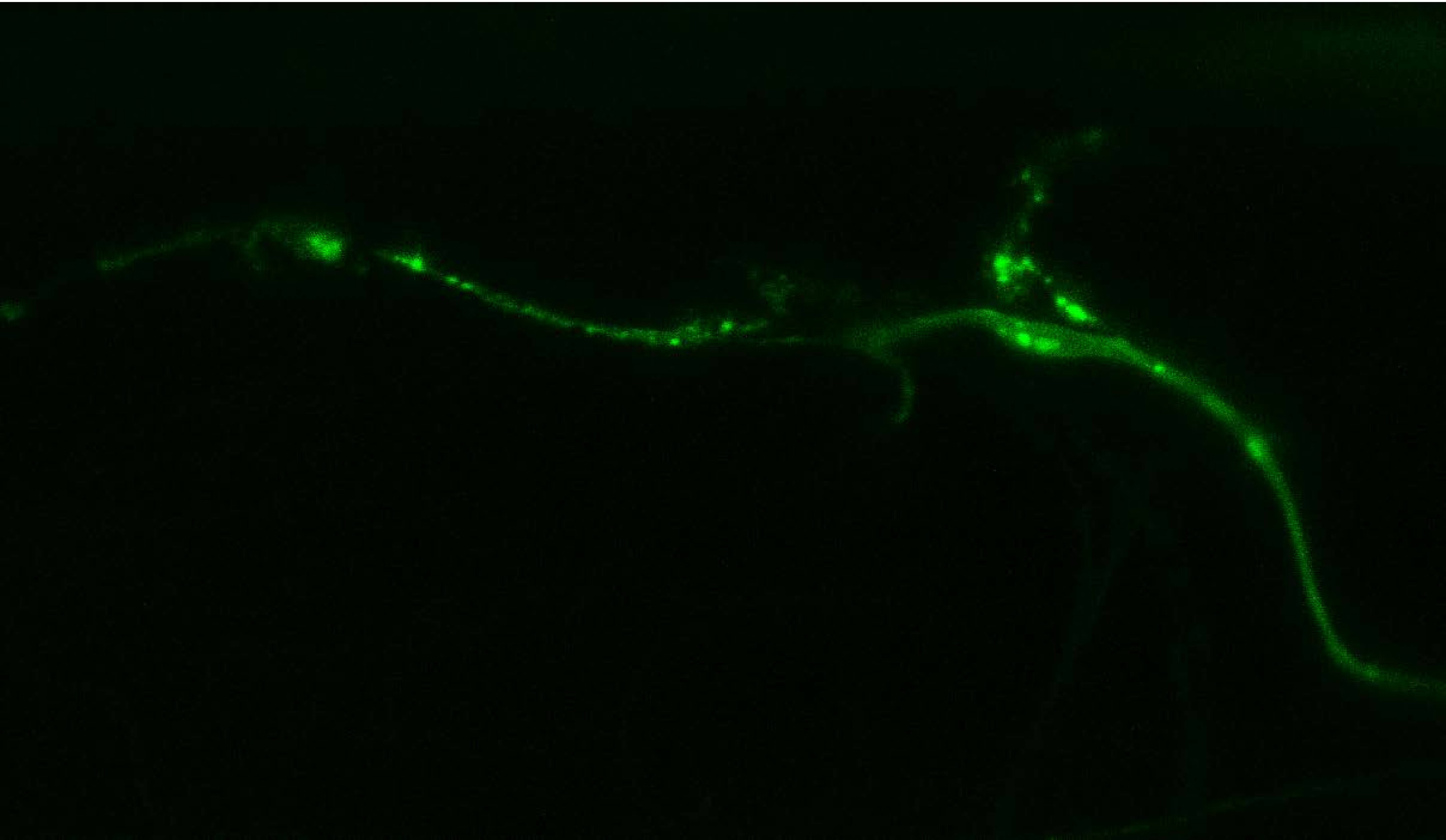

2A_6.tif

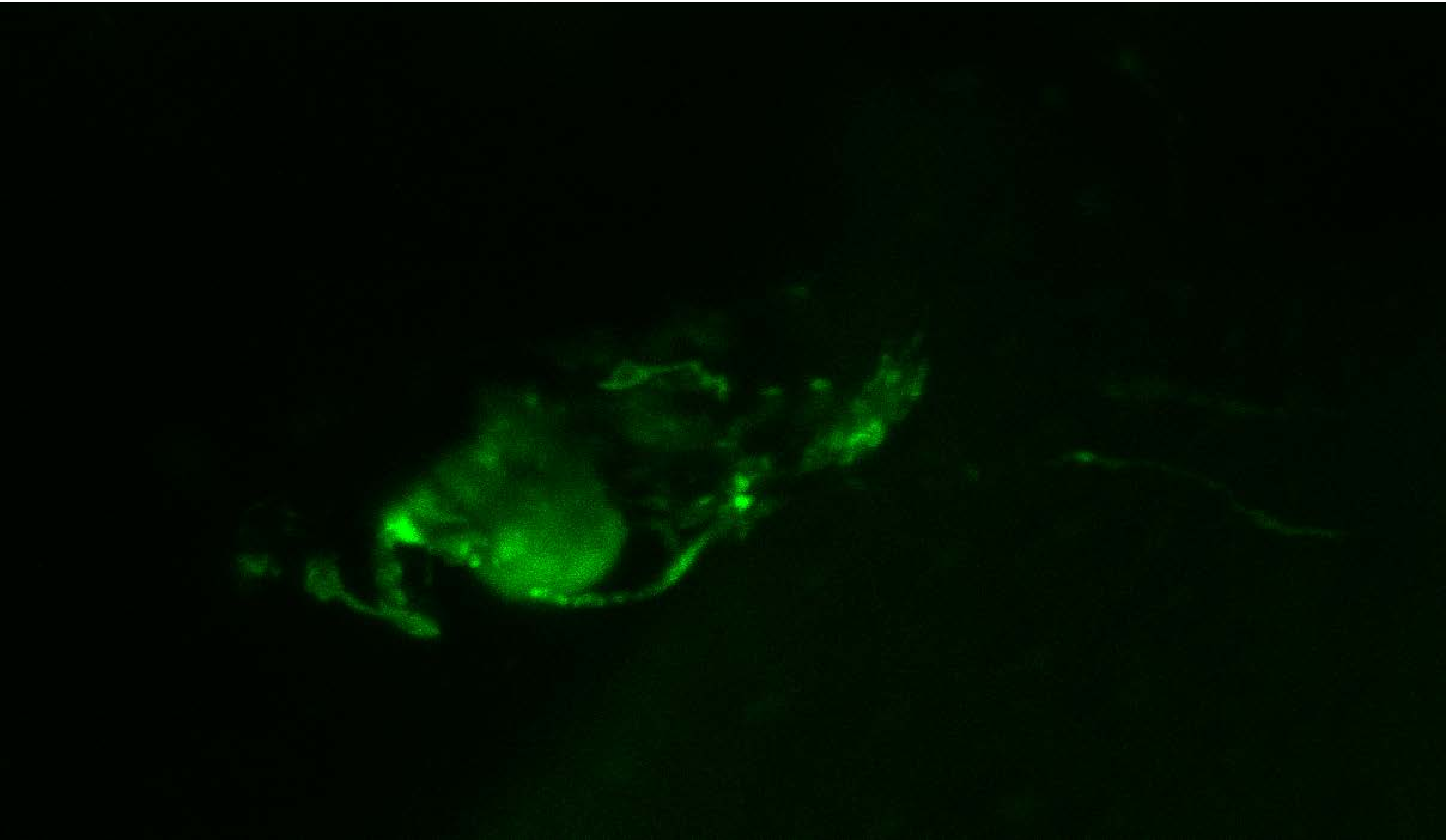

2A_7.tif

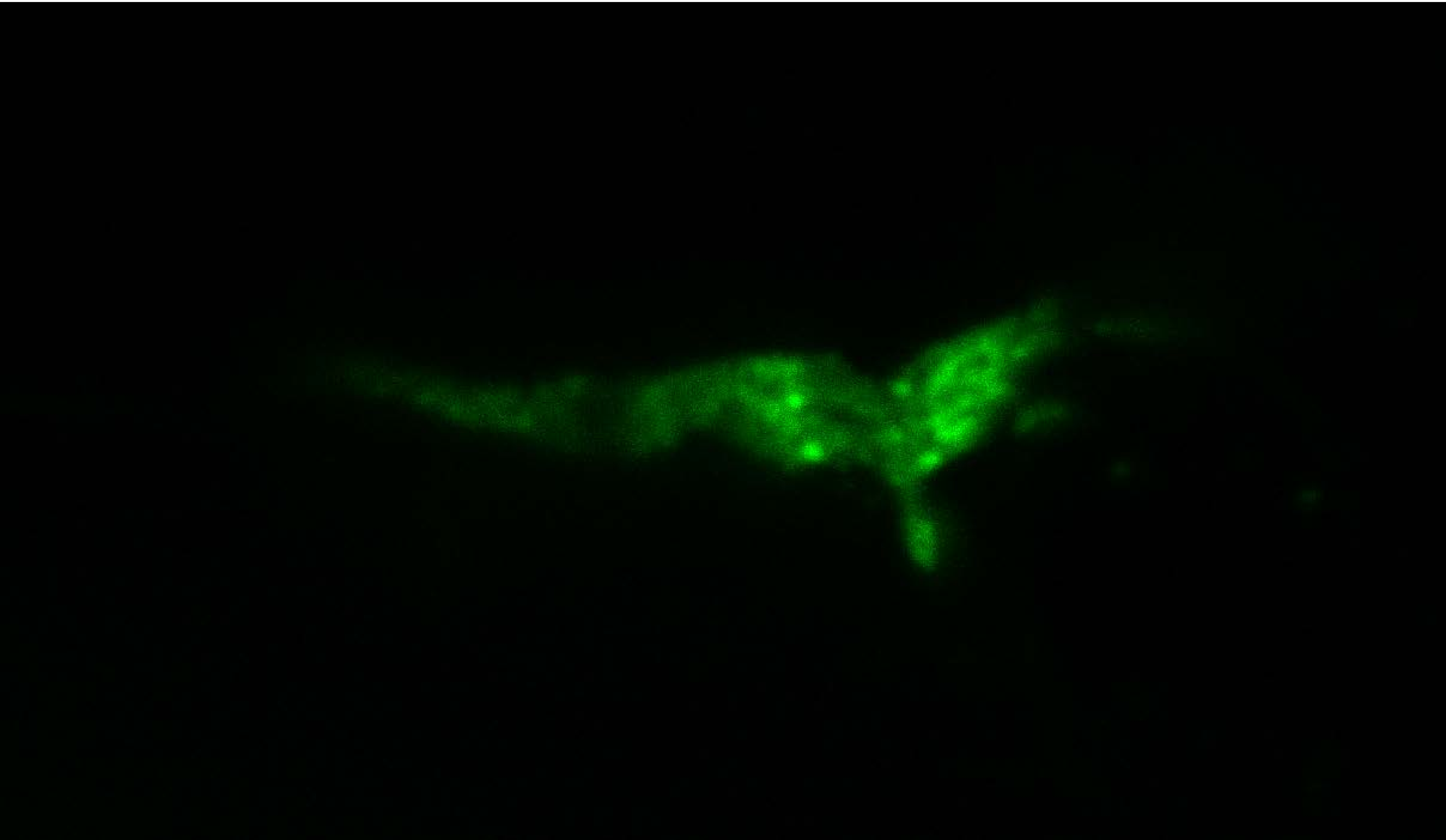

2A_8.tif

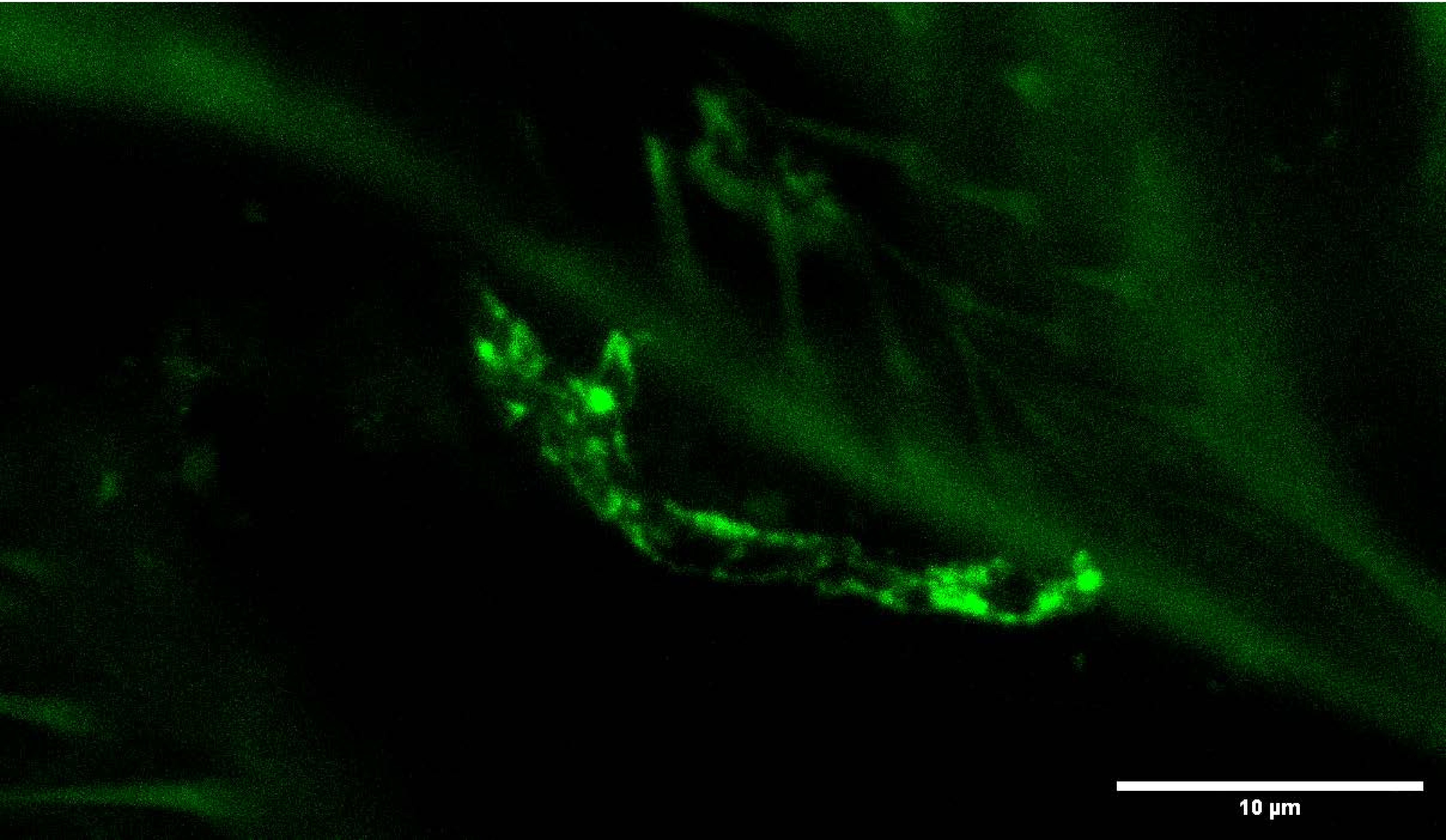

2A_9.tif

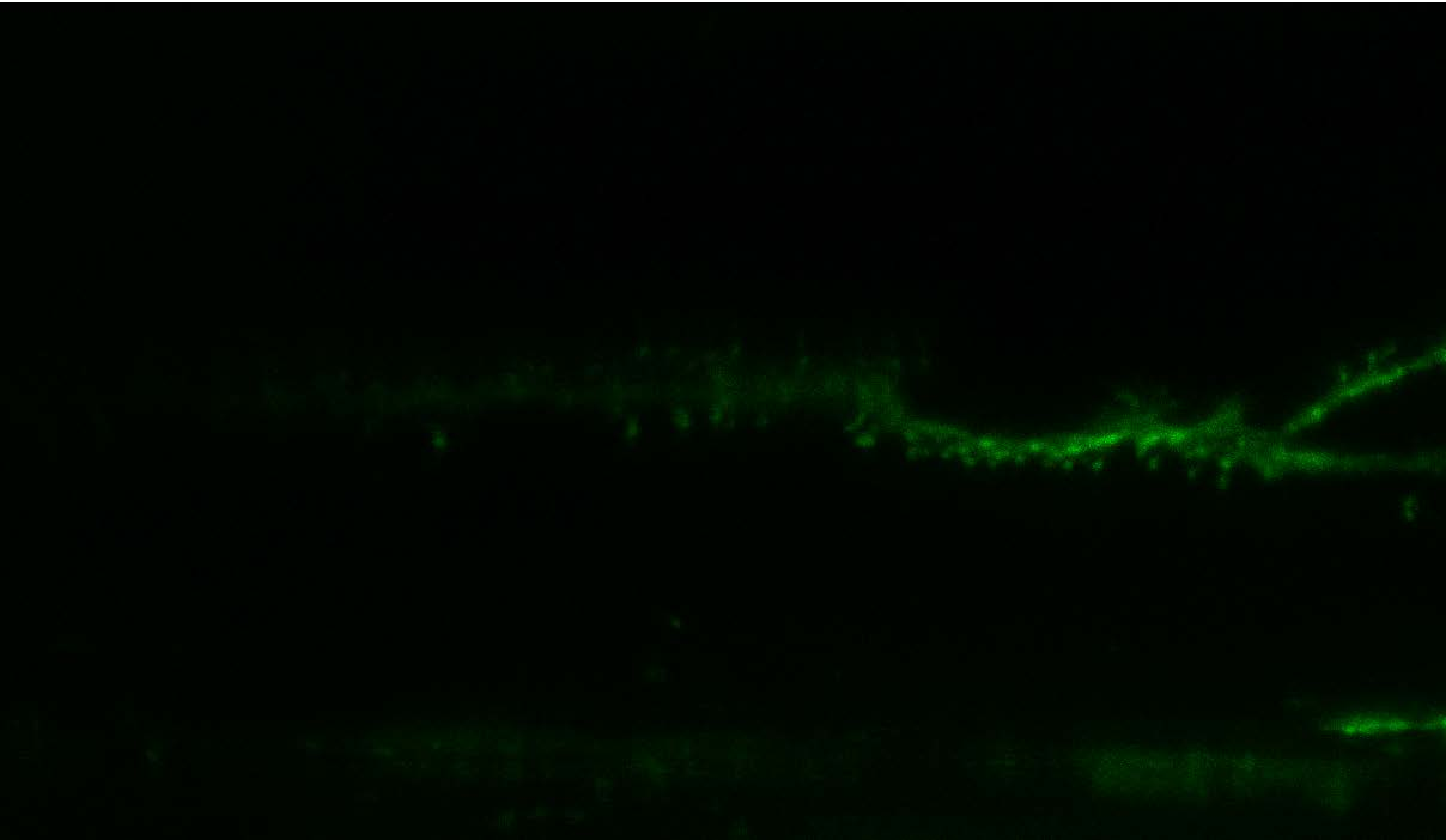

2A_10.tif

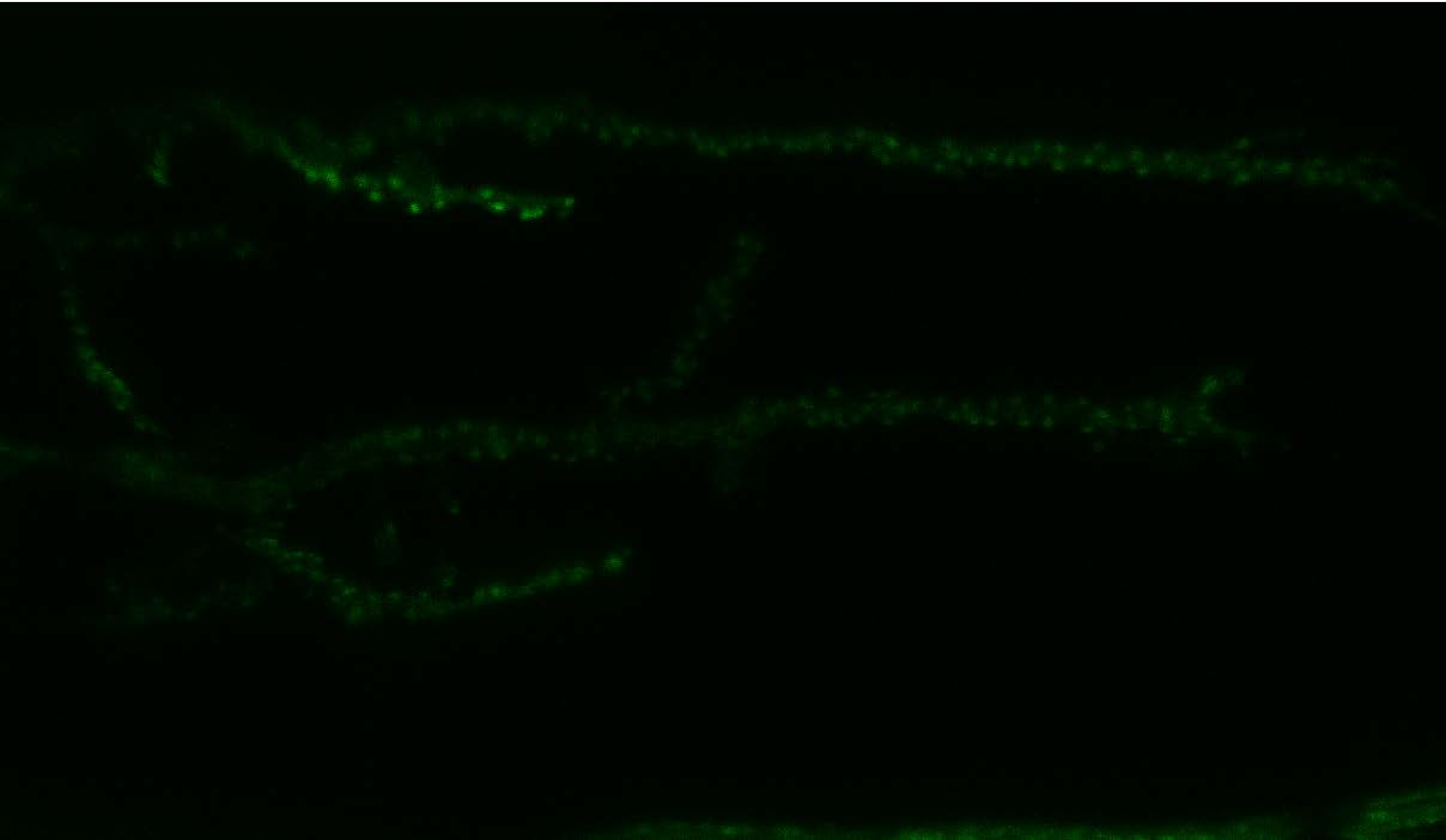

2A_11.tif

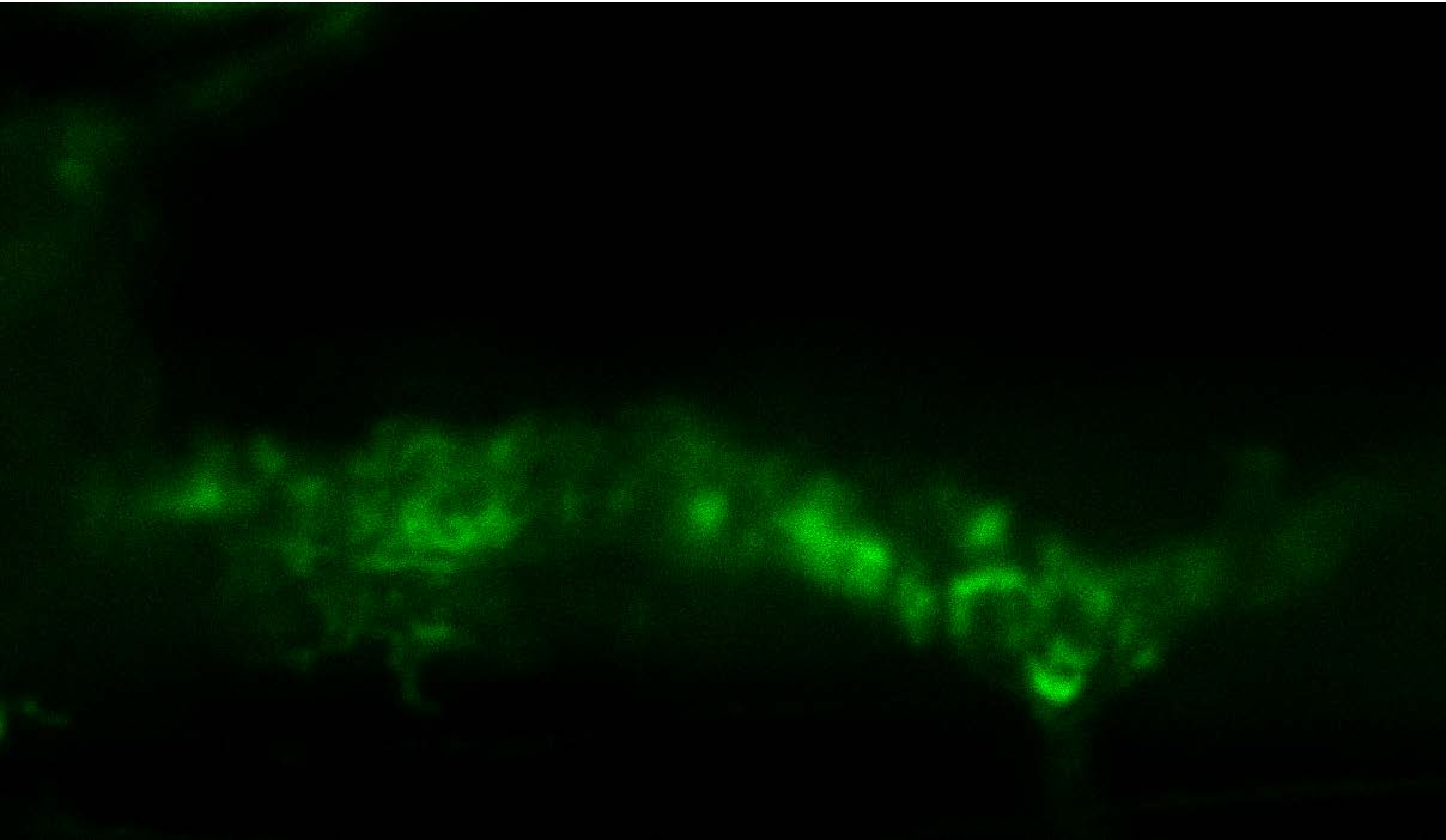

2A_12.tif

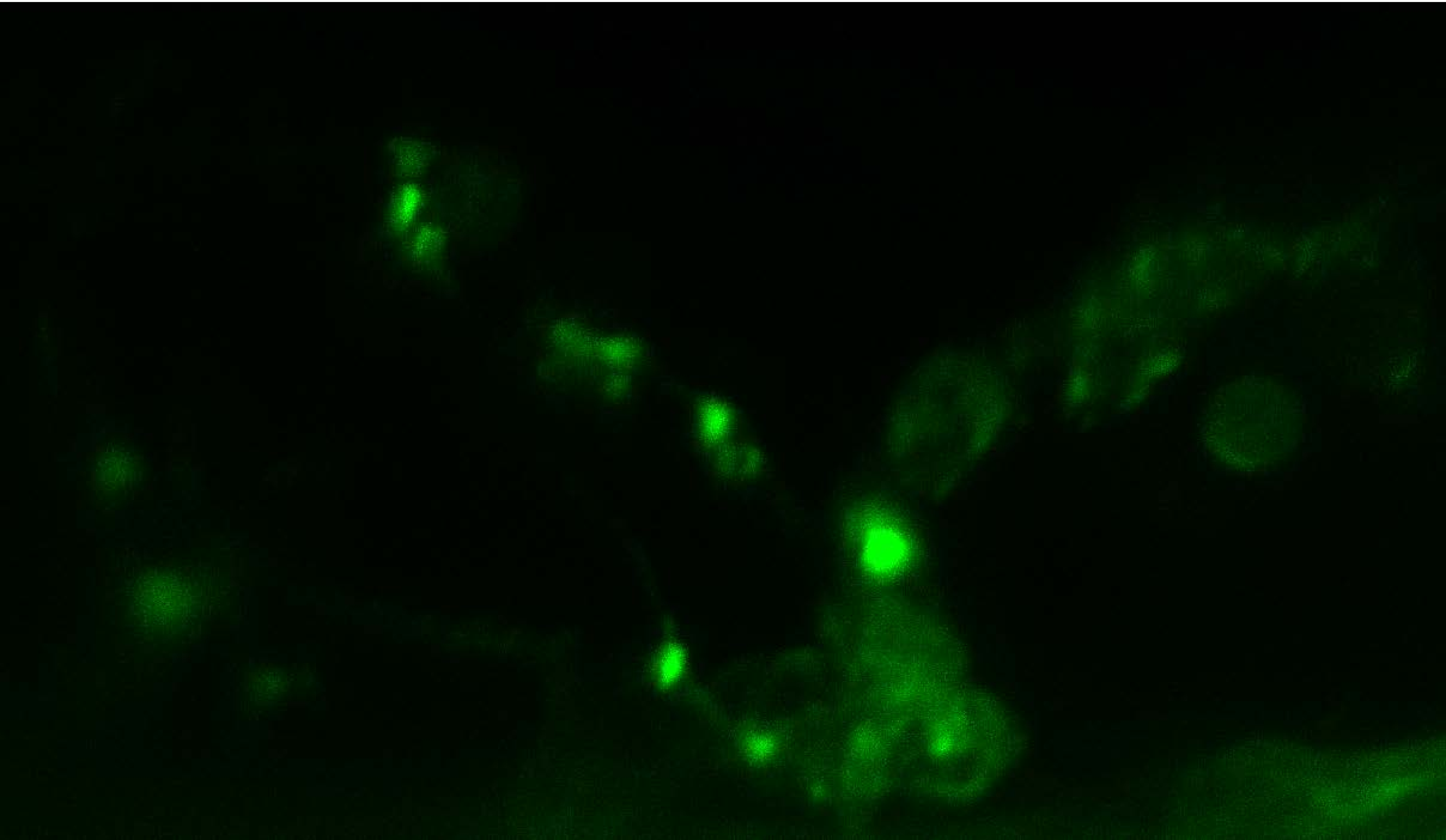

2A_13.tif

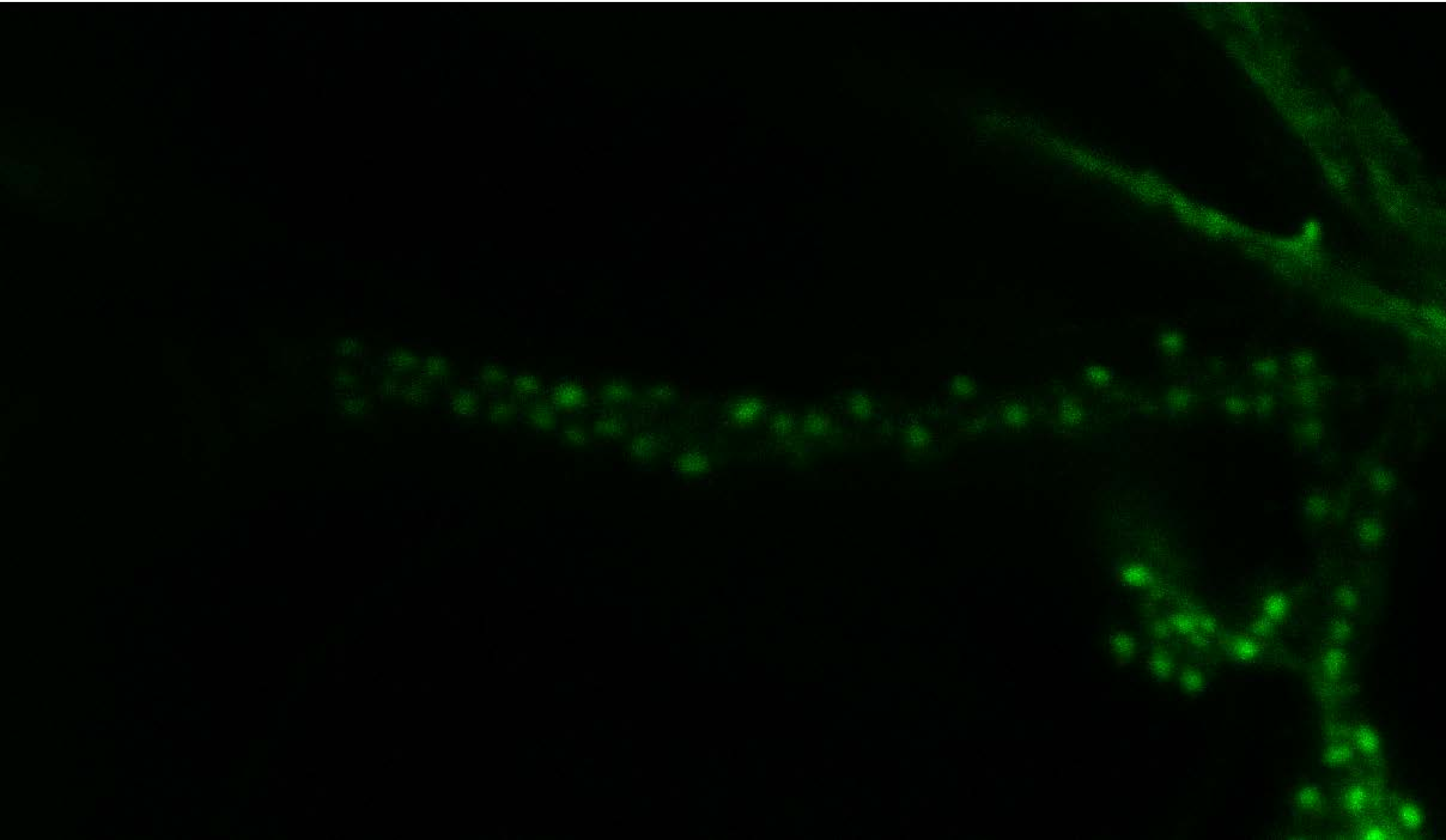

2A_14.tif

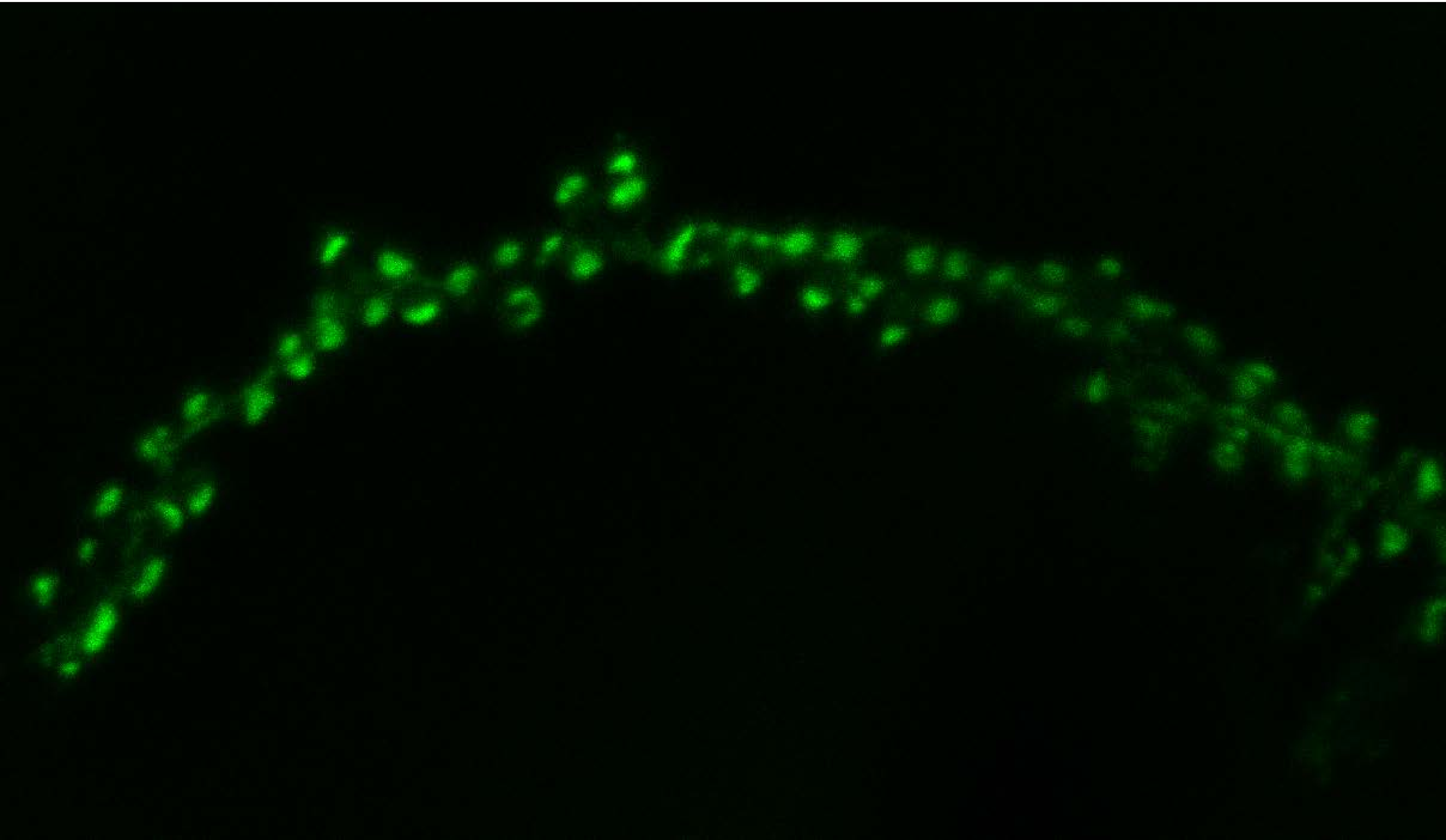

2A_15.tif

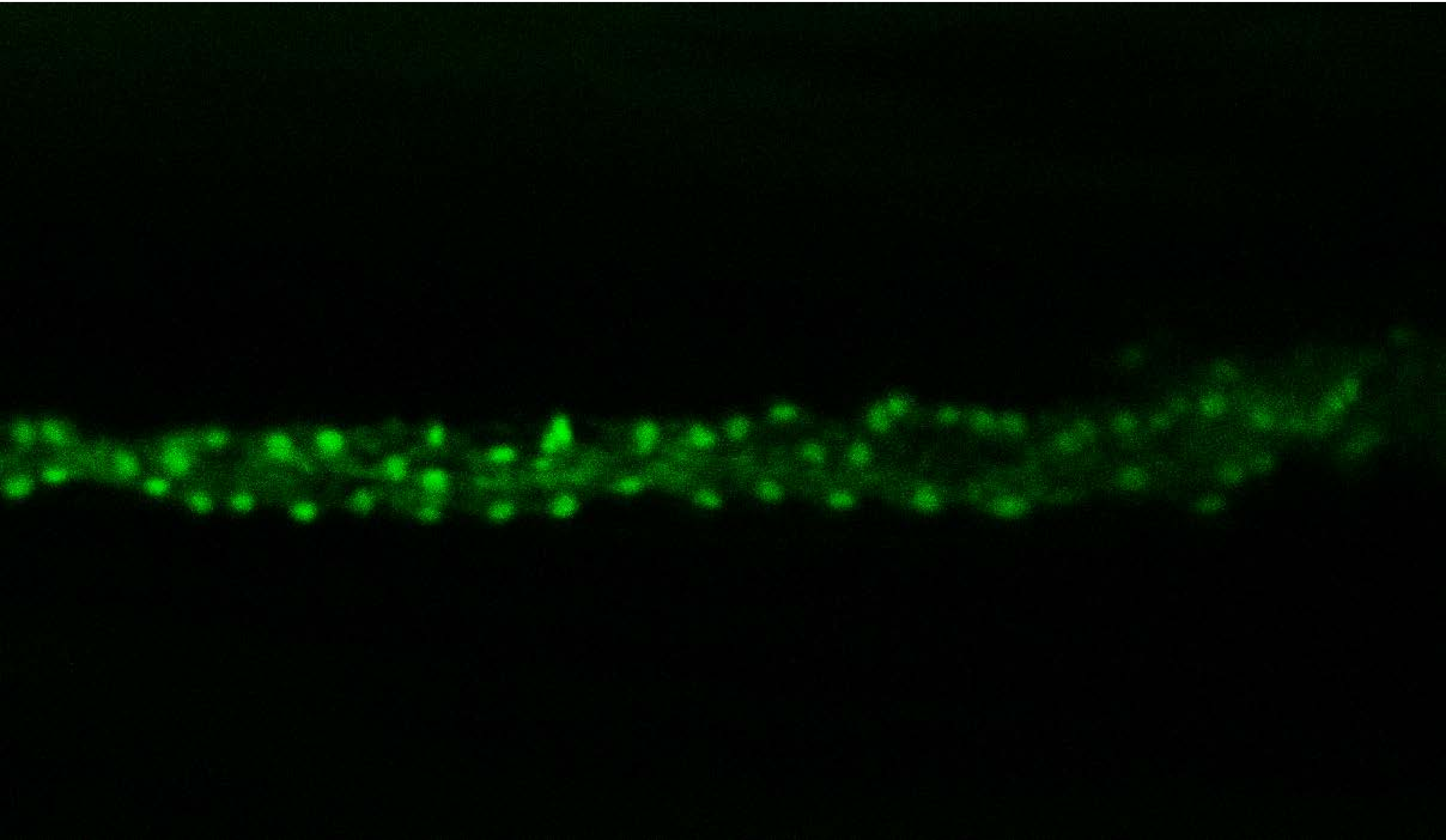

2A_16.tif

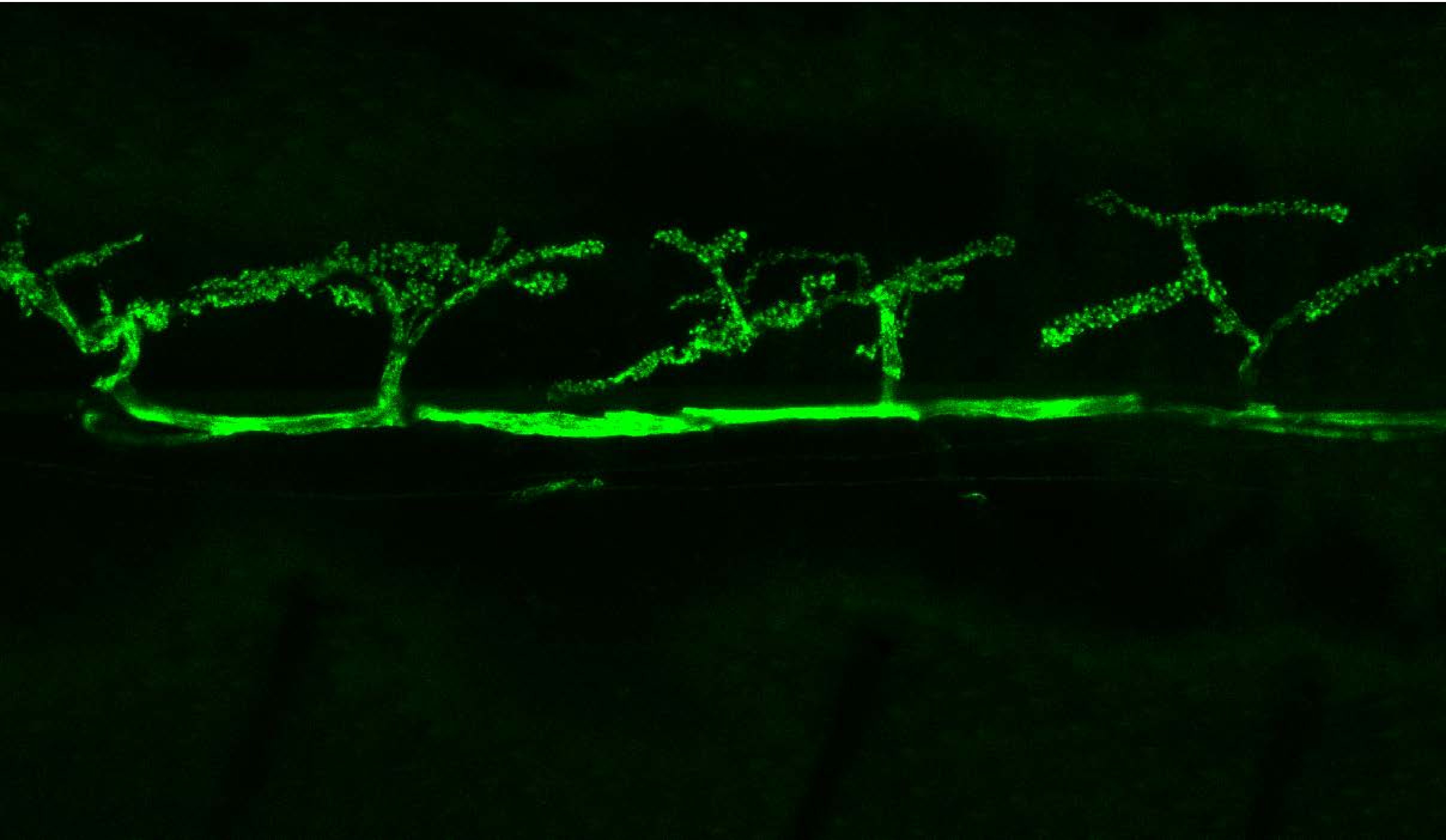

3B_1.tif

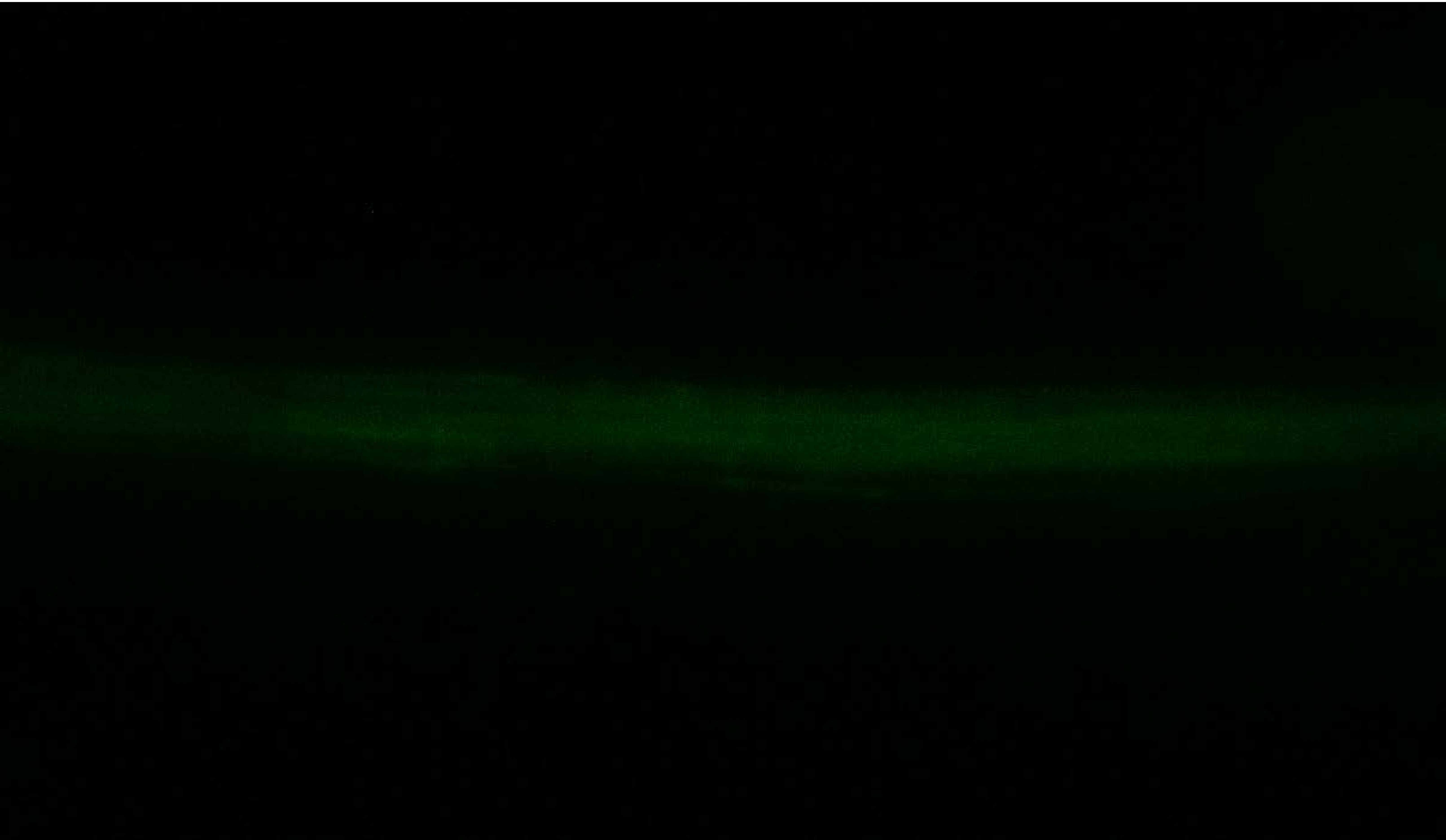

3B_2.tif

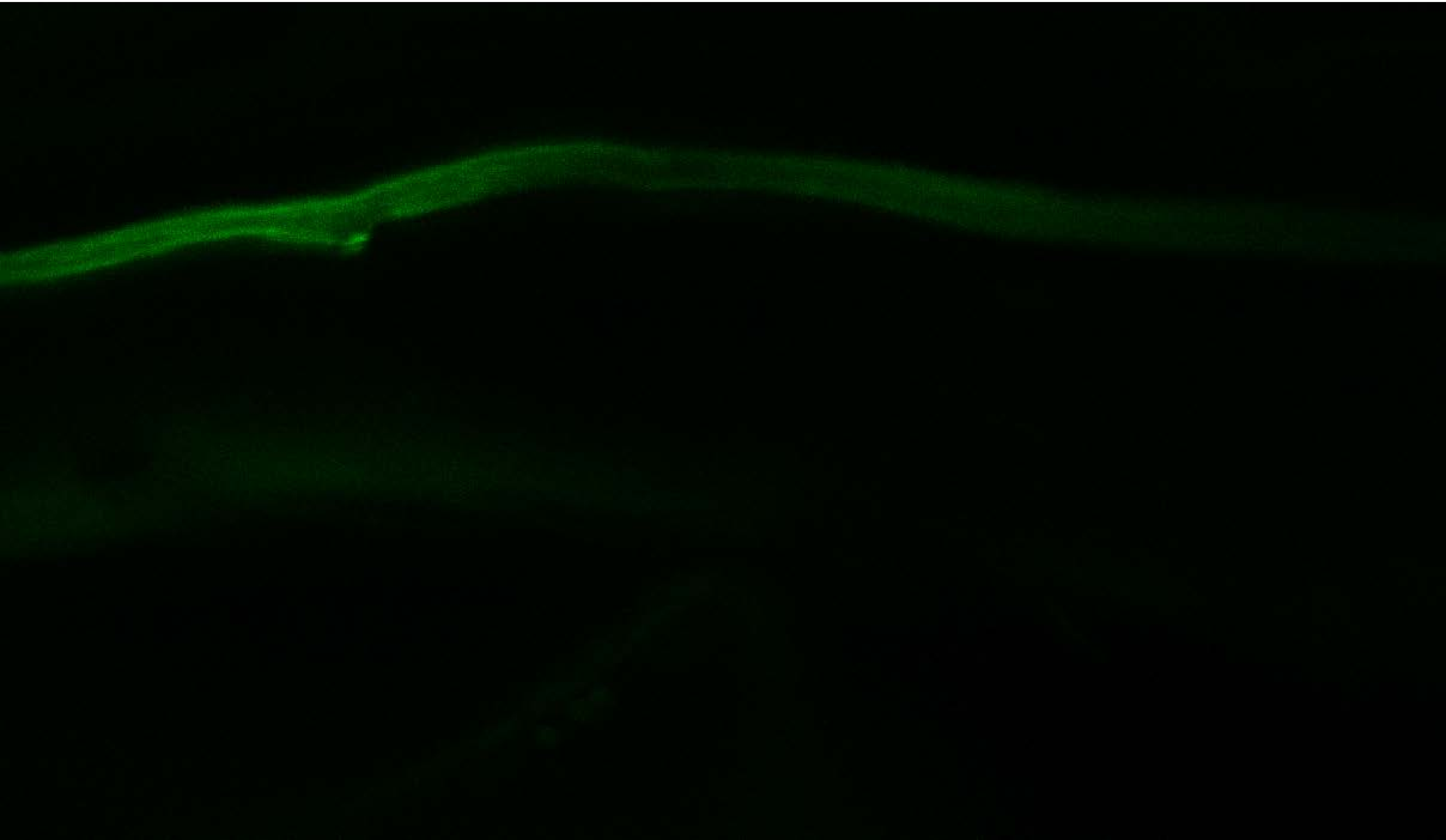

3B_3.tif

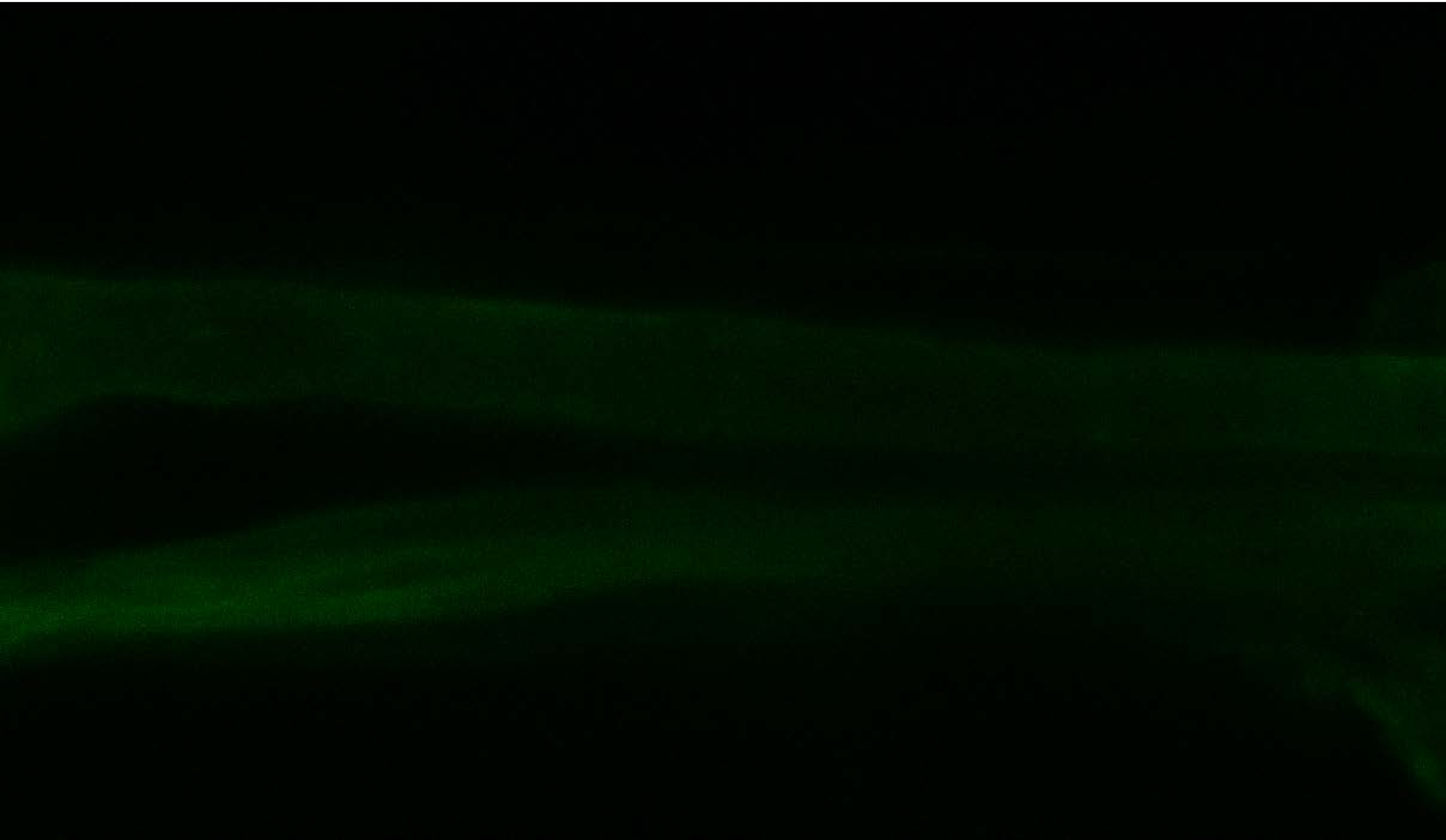

3B_4.tif

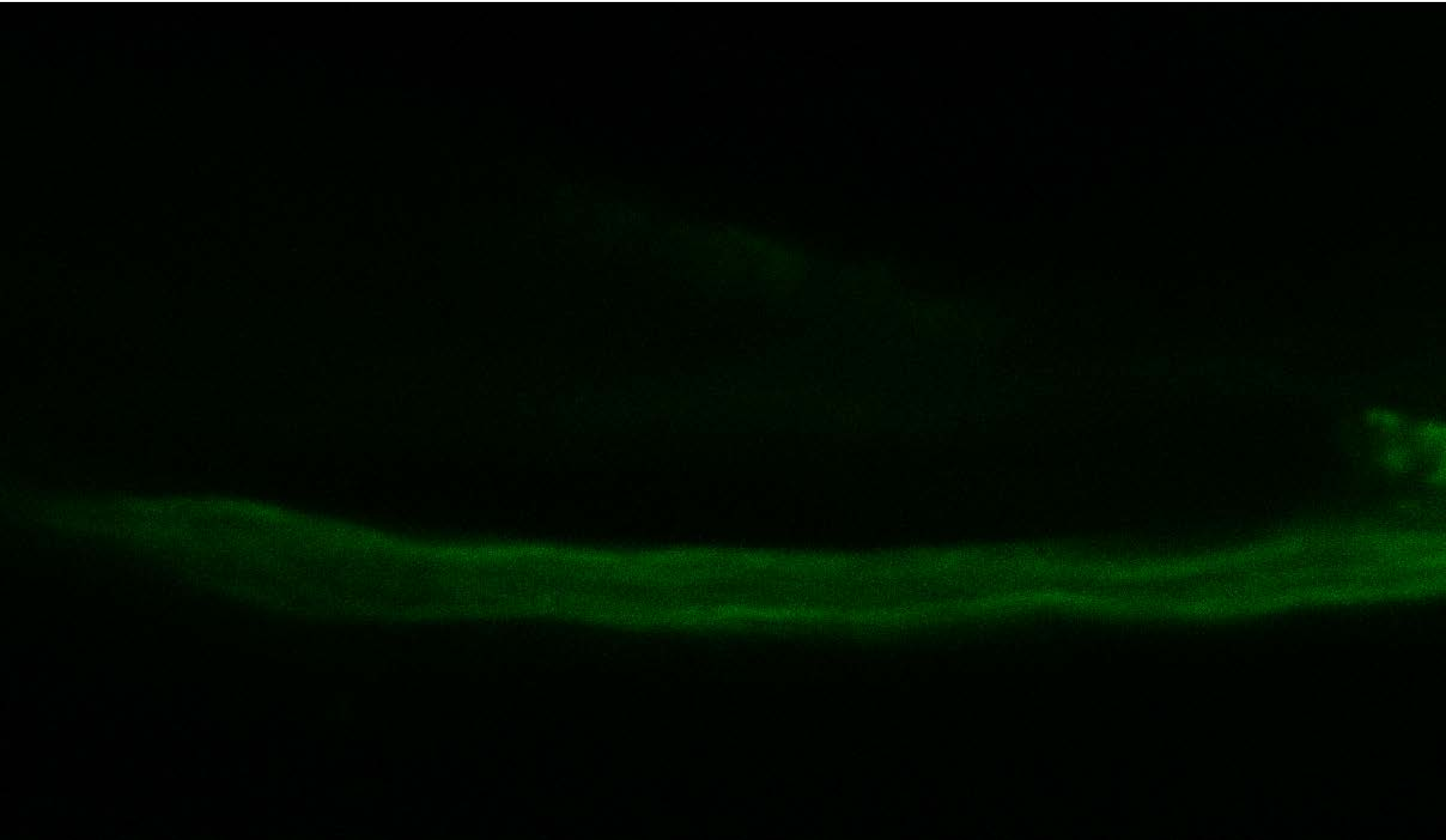

3B_5.tif

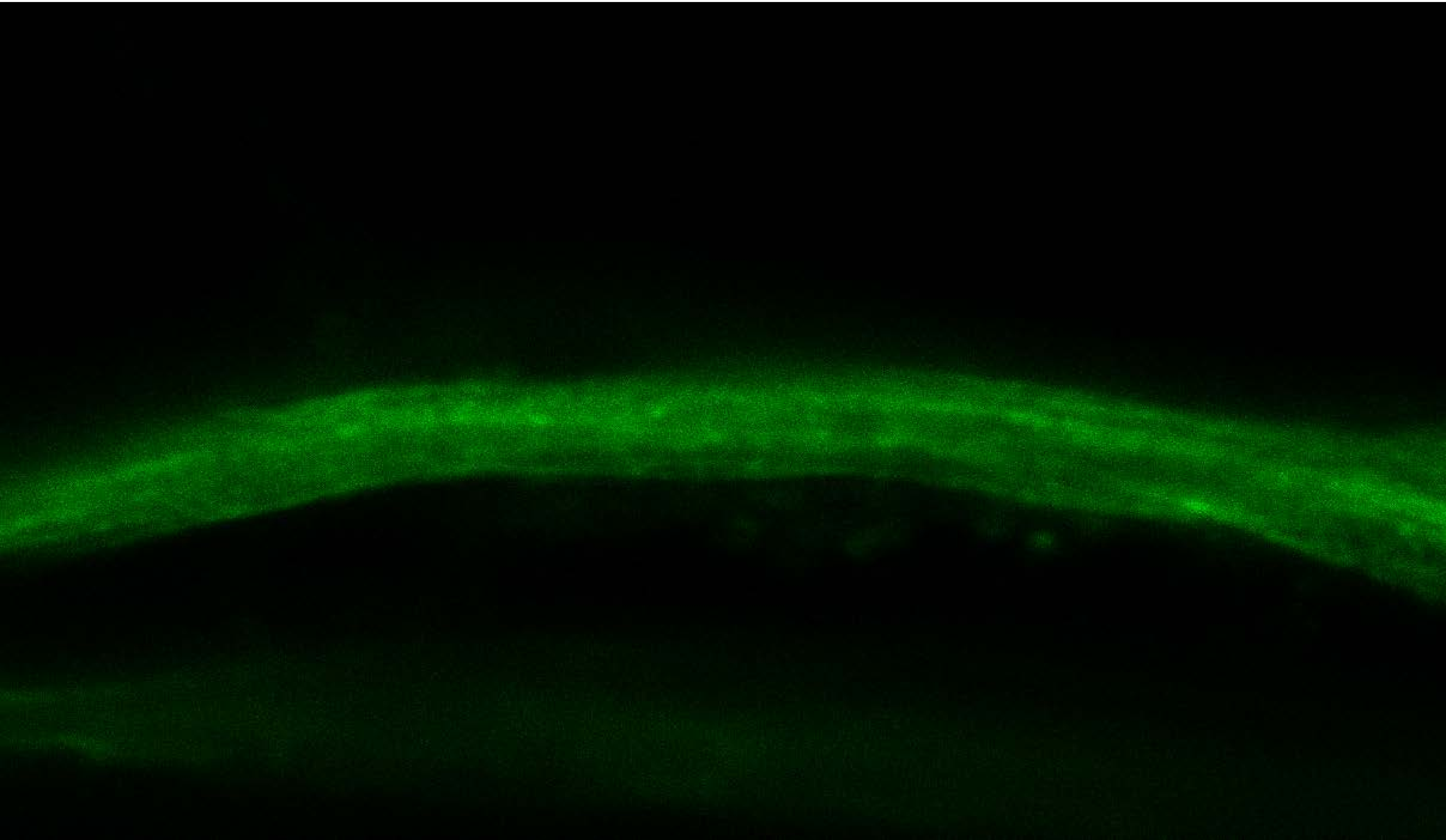

3B_6.tif

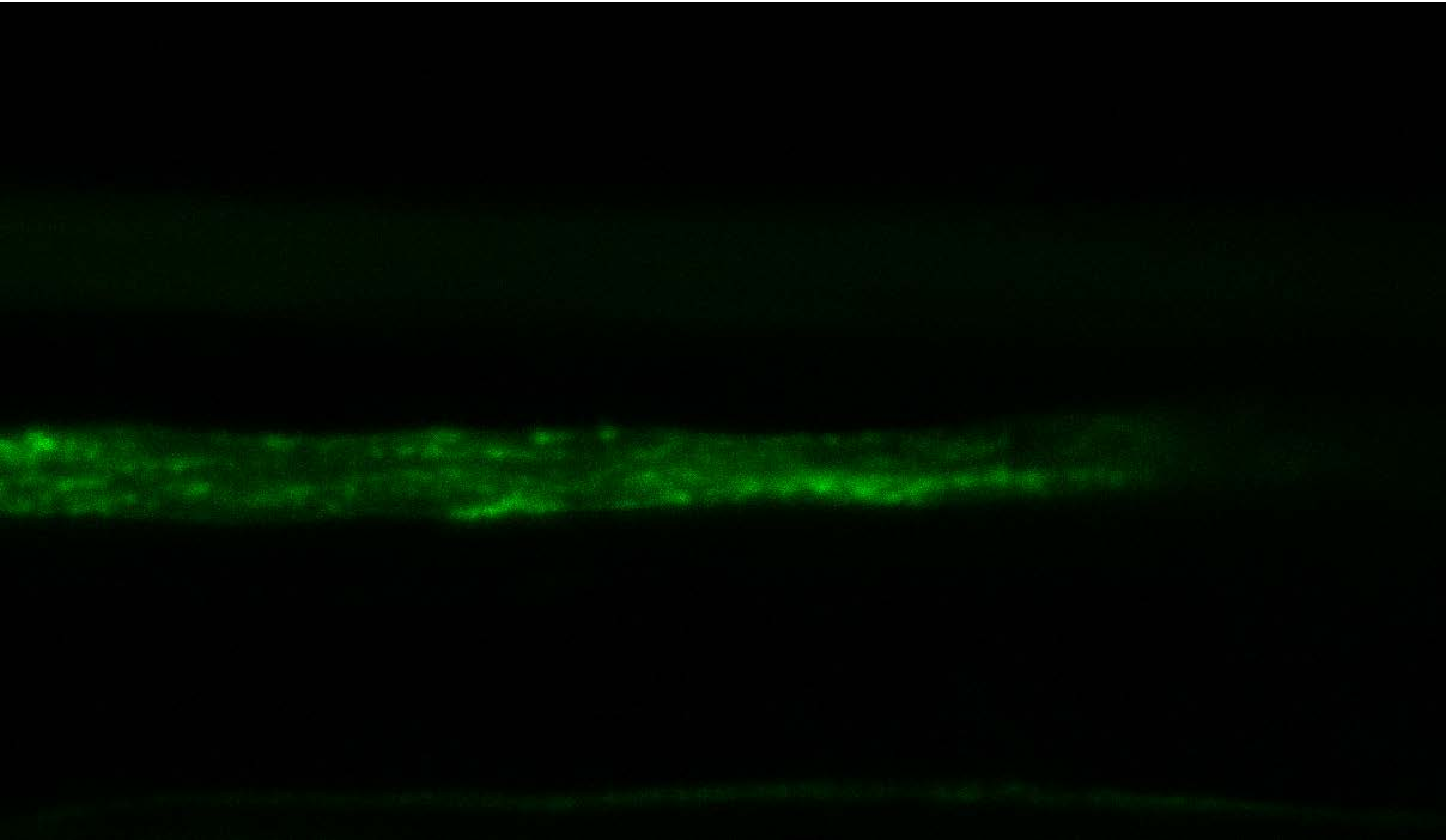

3B_7.tif

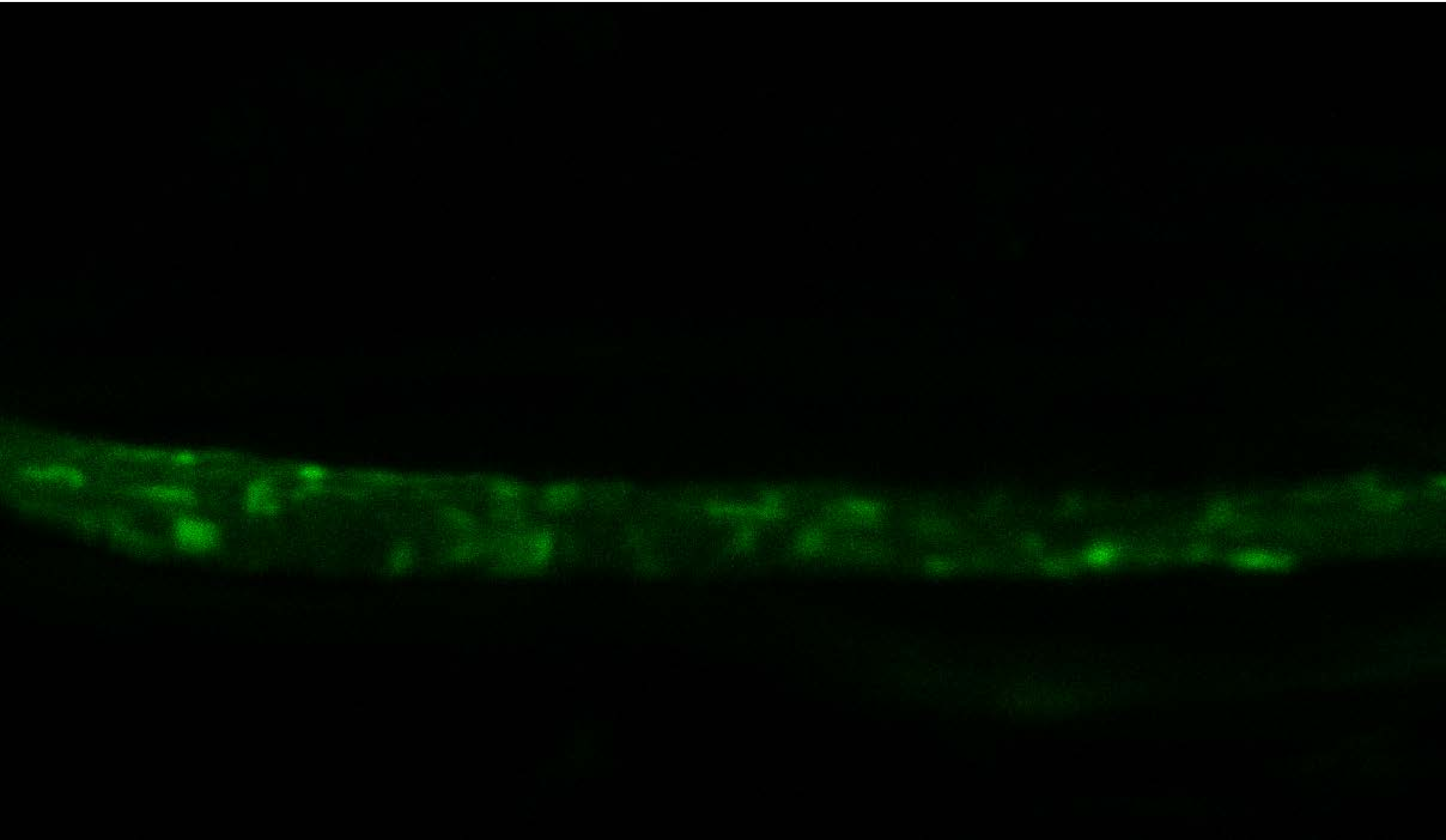

3B_8.tif

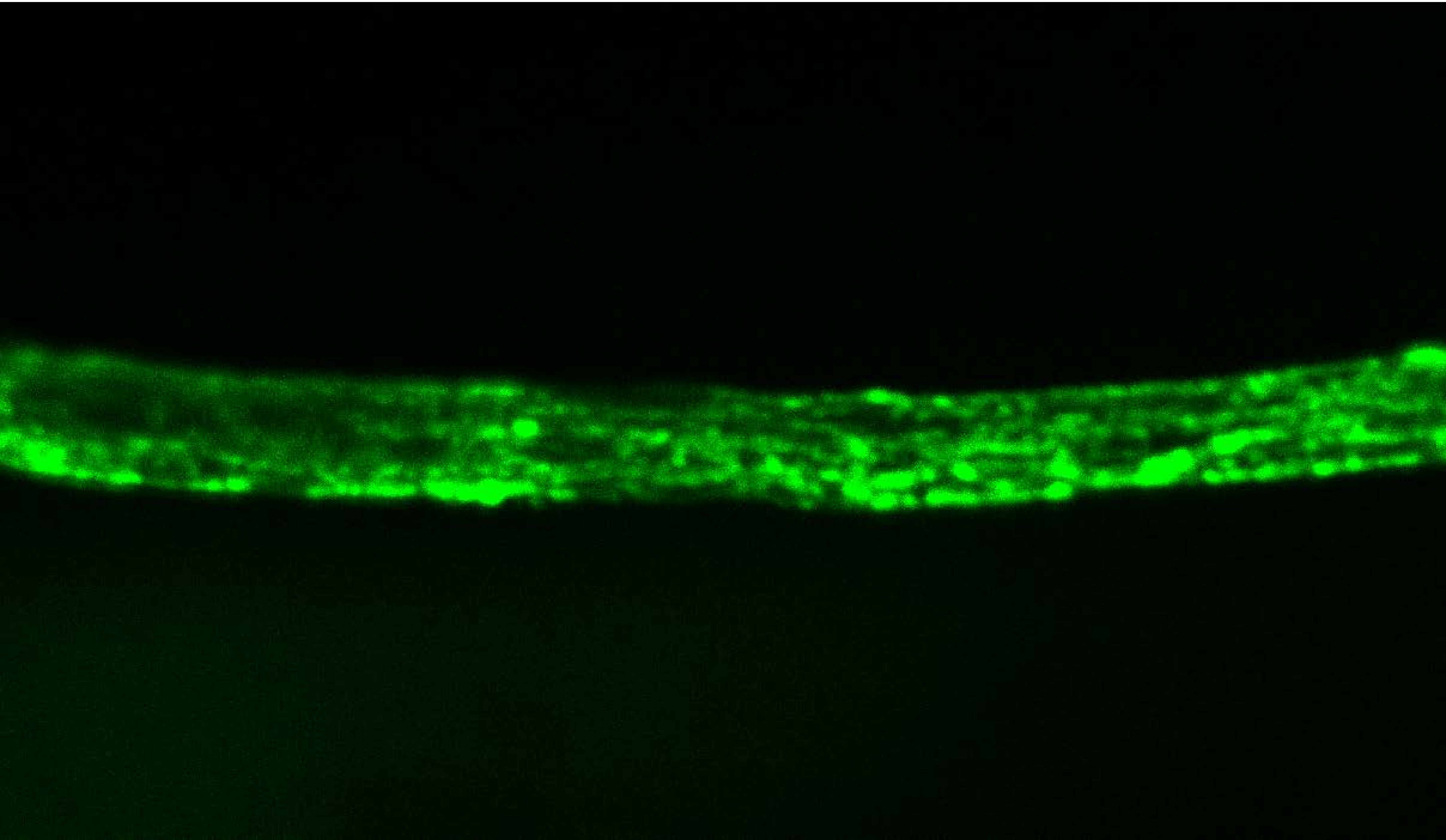

3B_9.tif

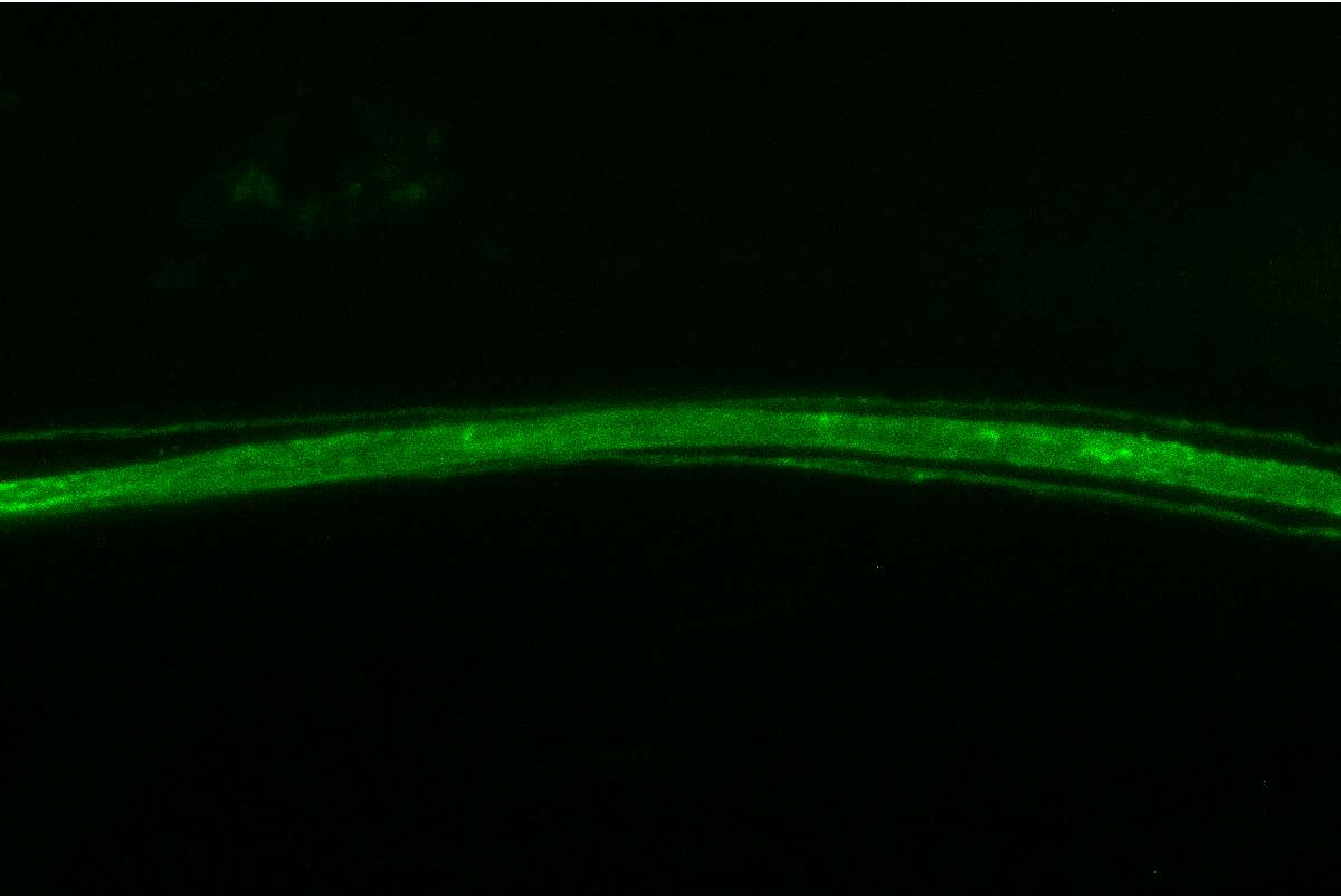

3B_10.tif

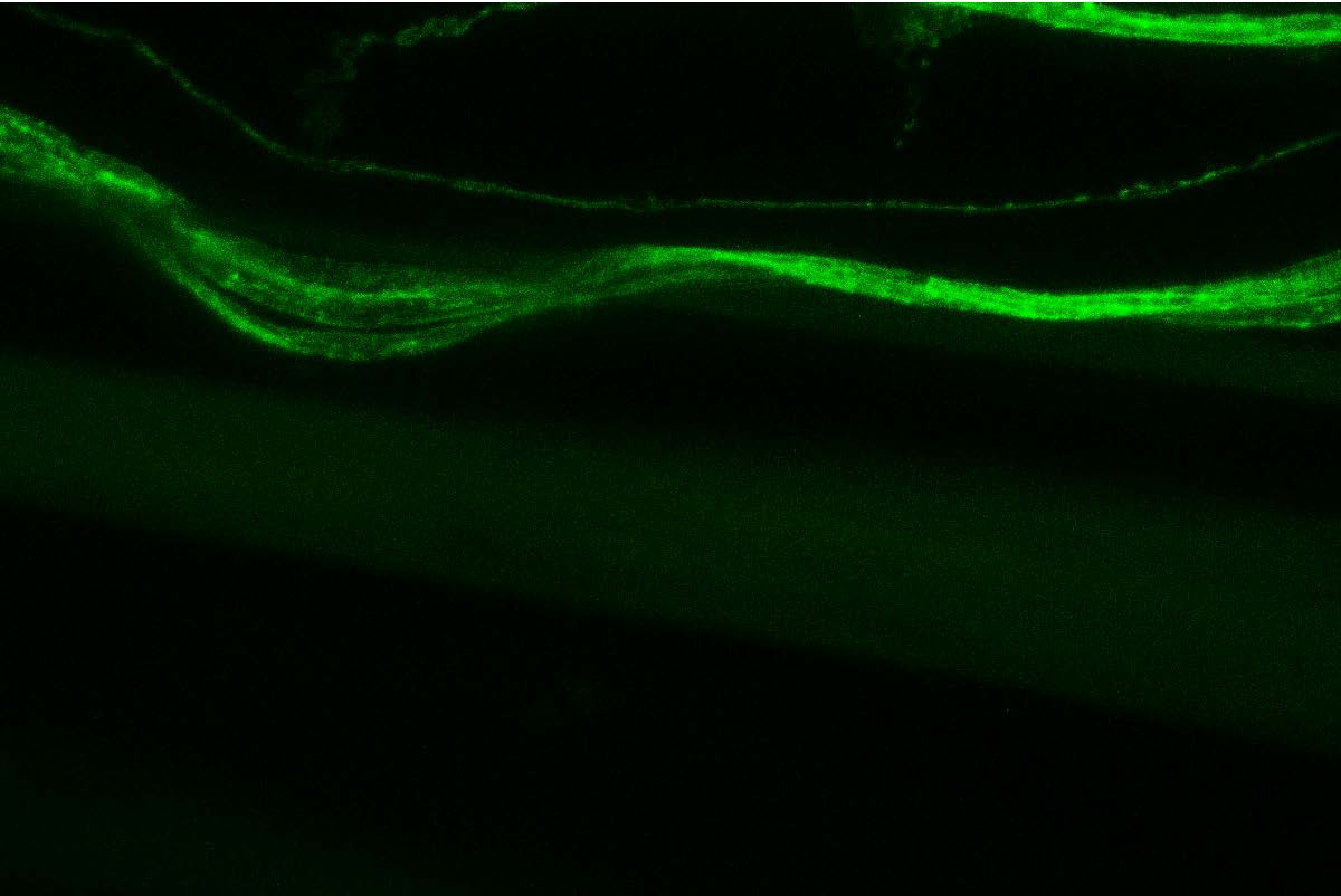

3B_11.tif

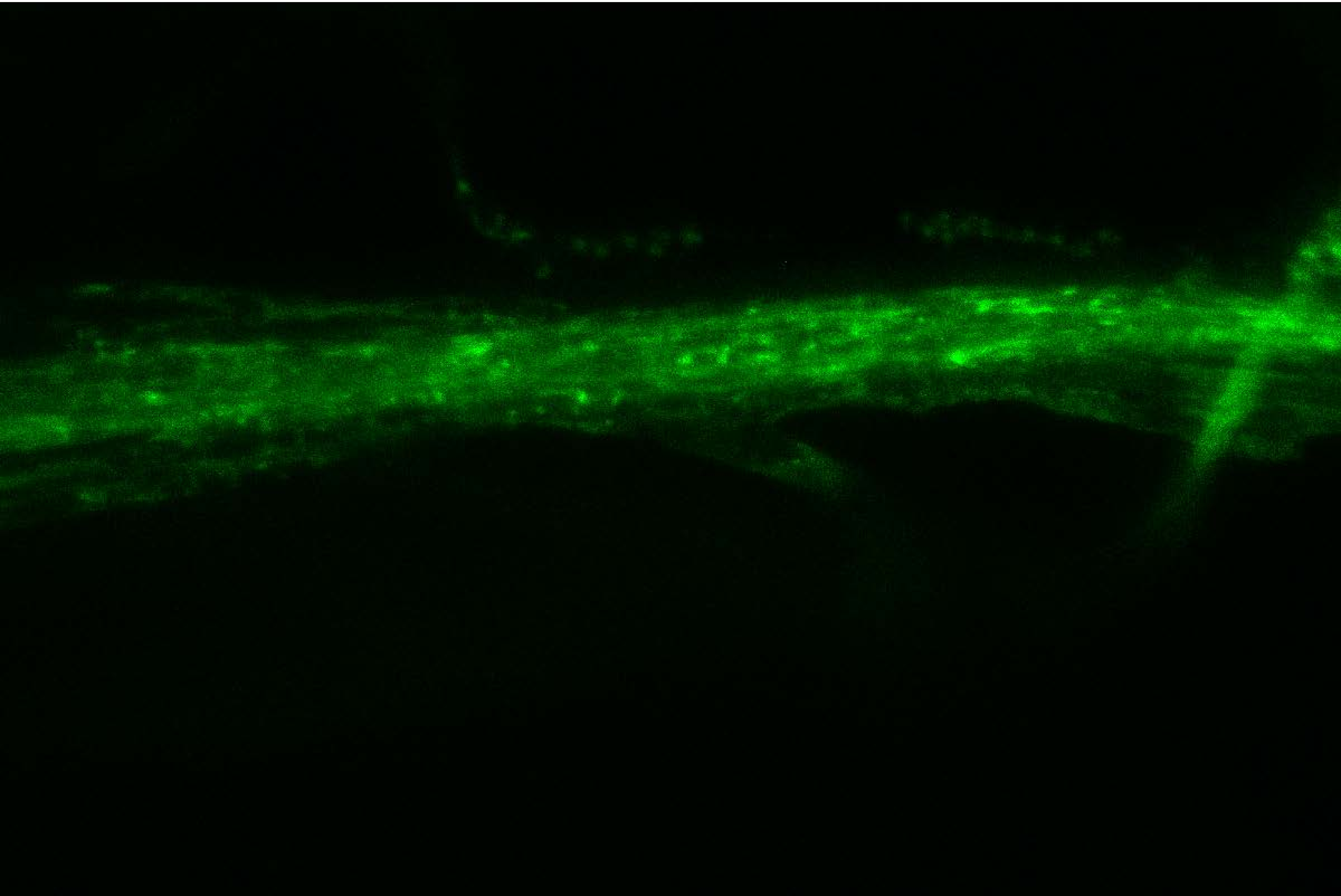

3B_12.tif

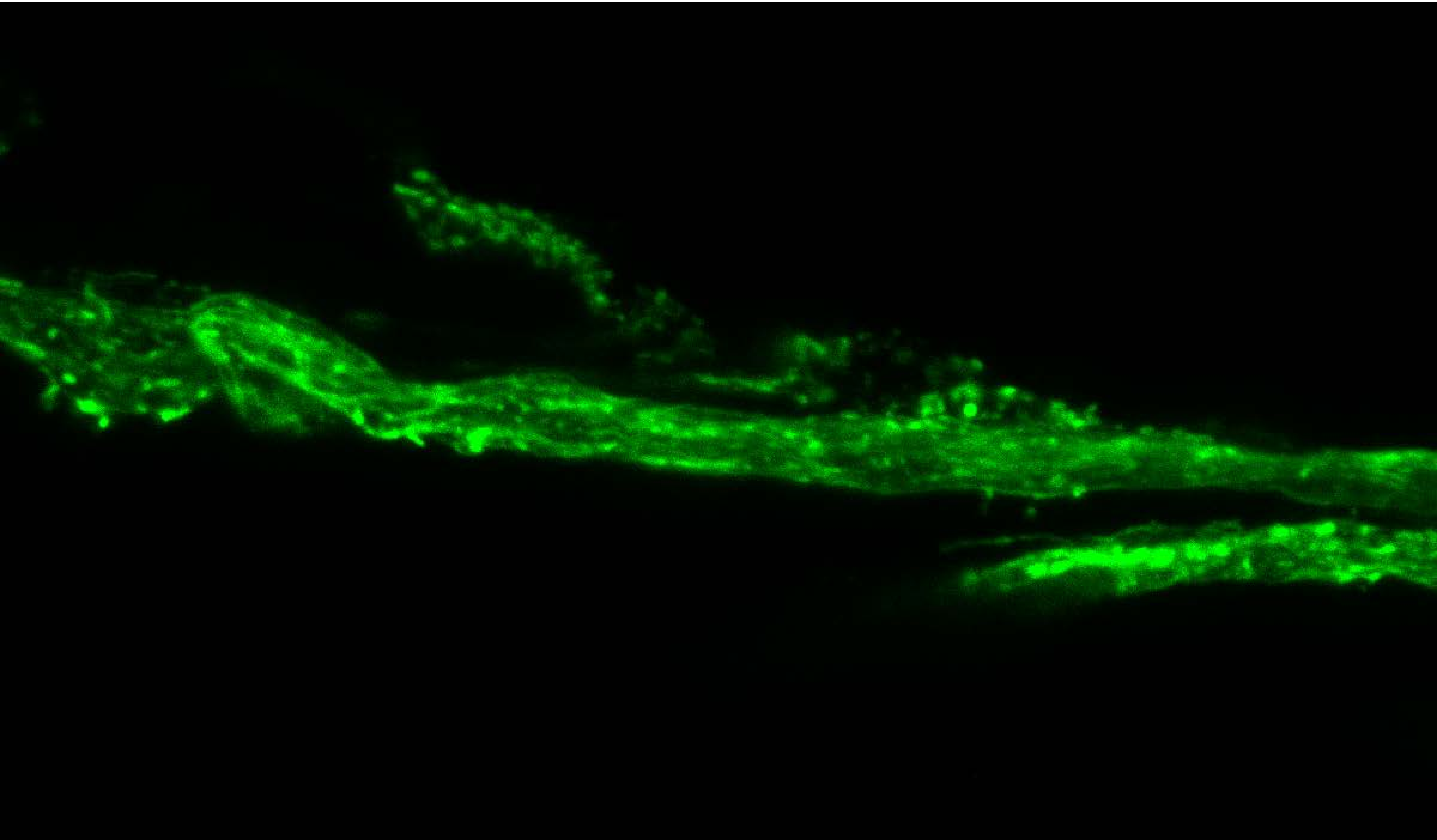

3B_13.tif

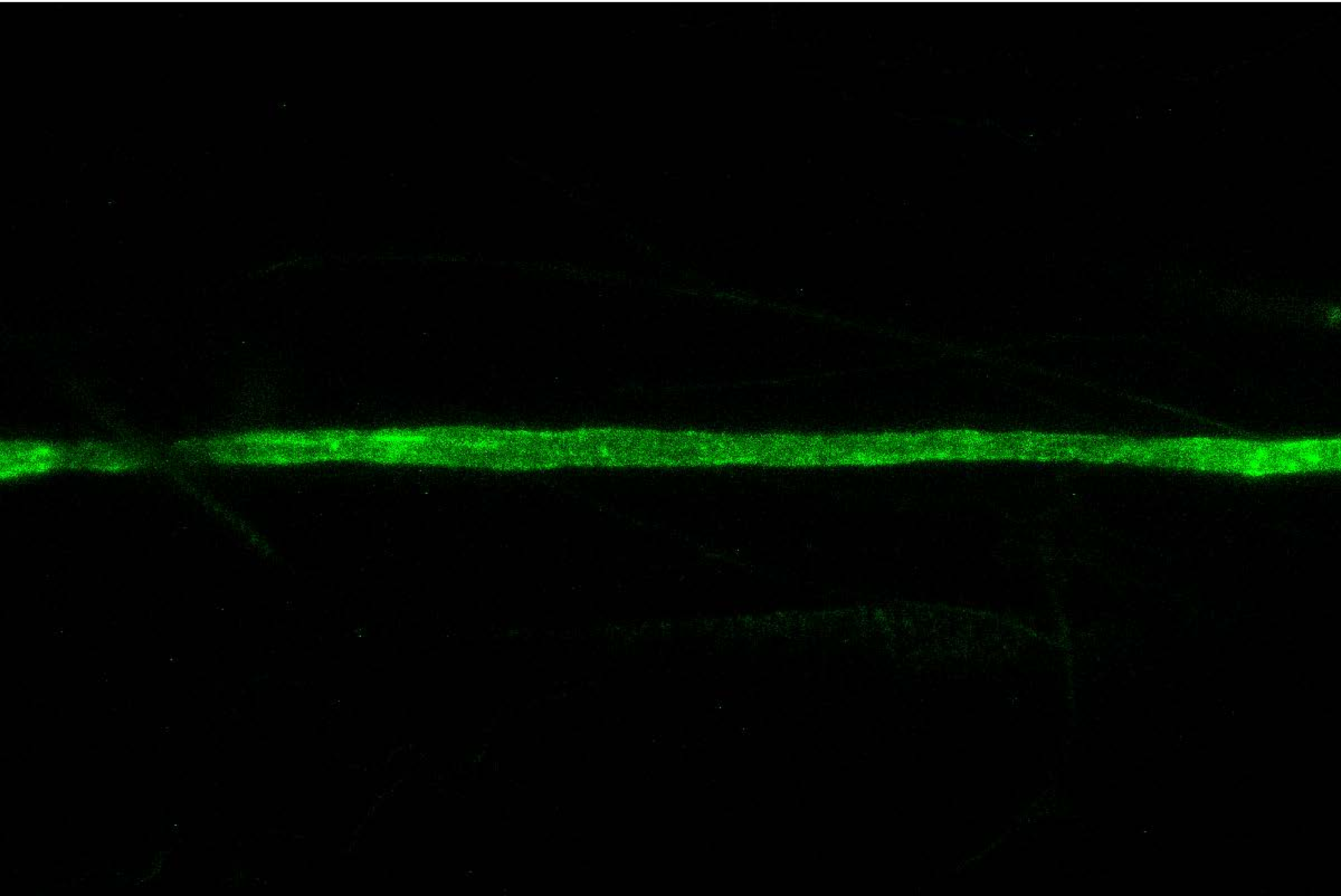

3B_14.tif

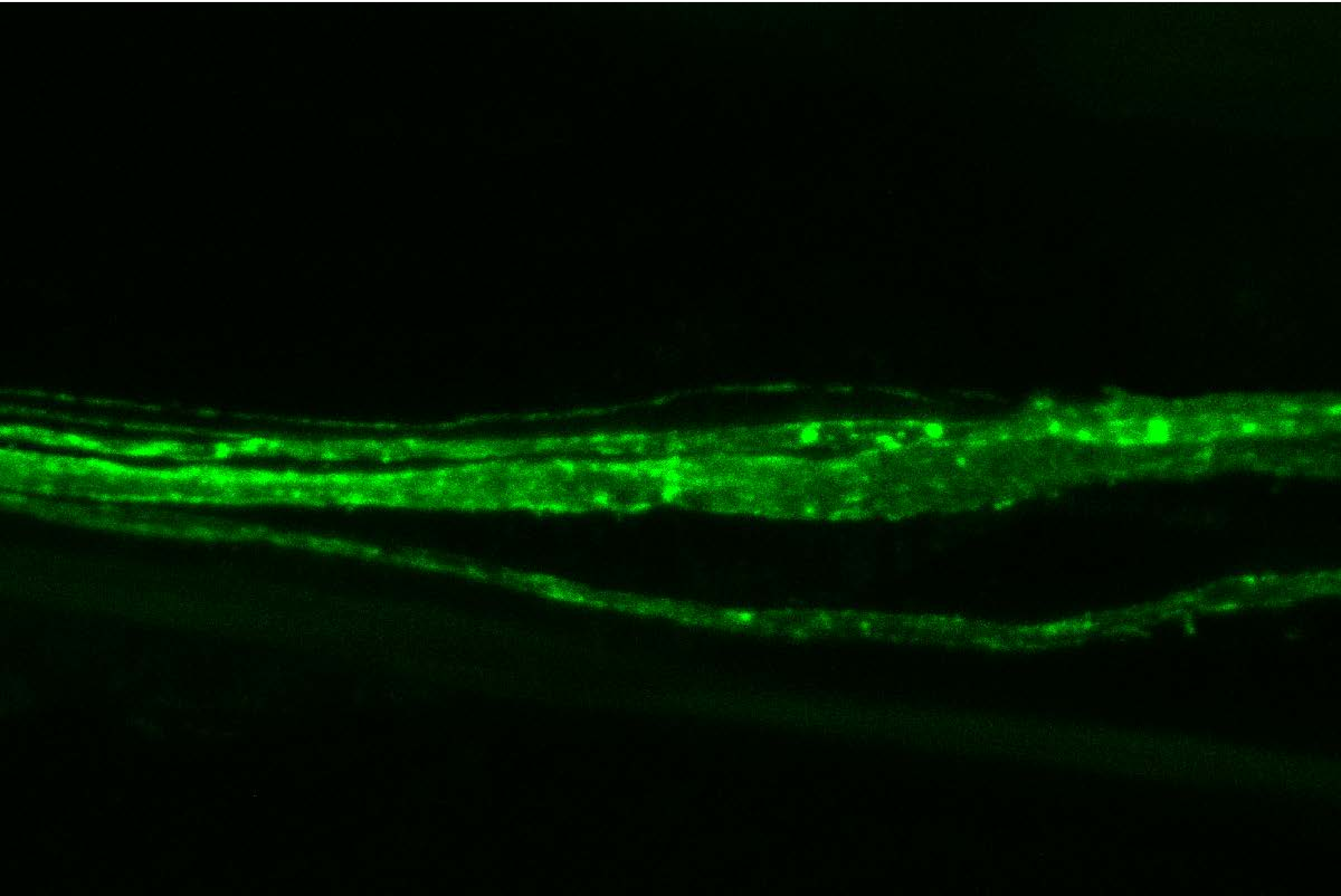

3B_15.tif

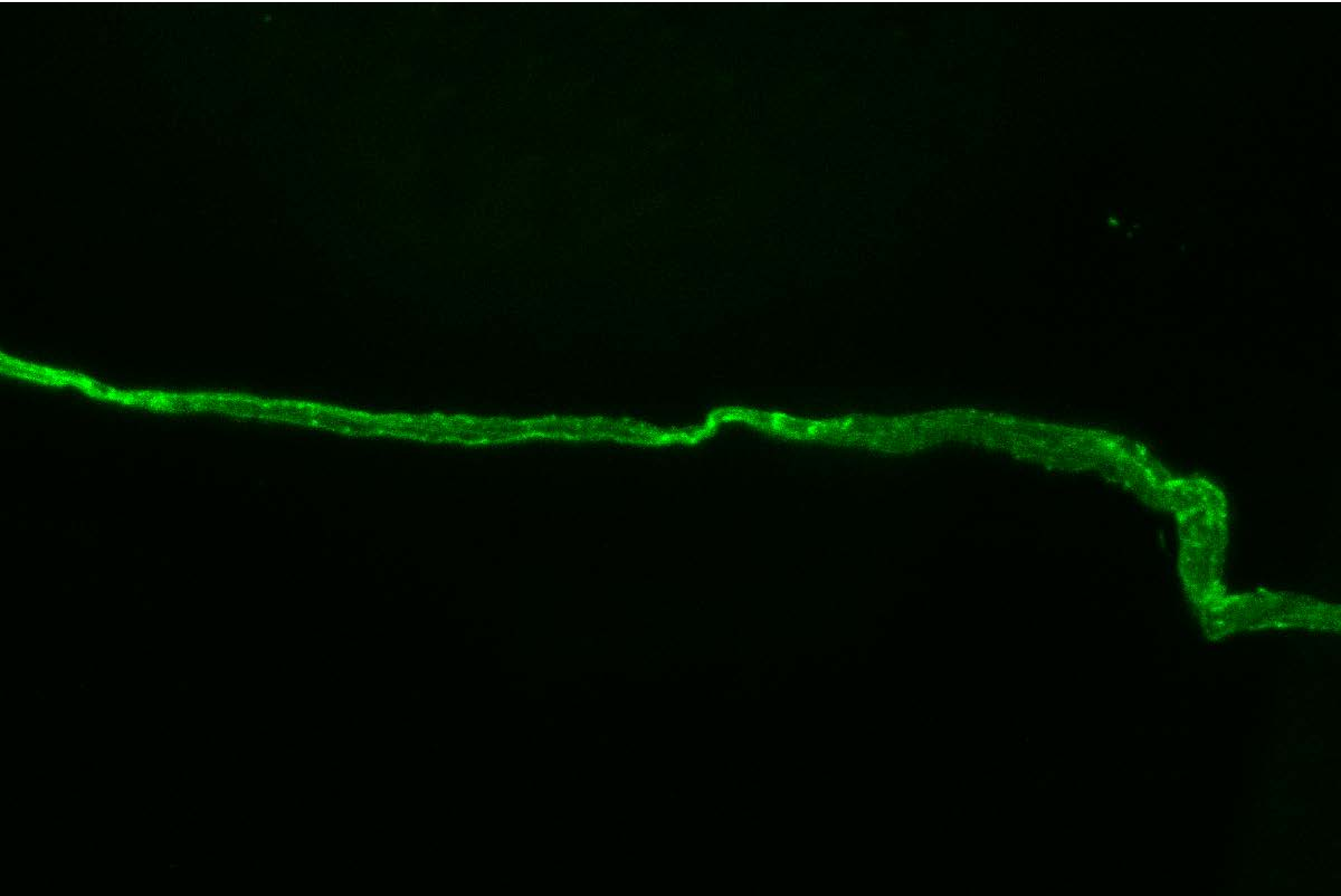

3B_16.tif

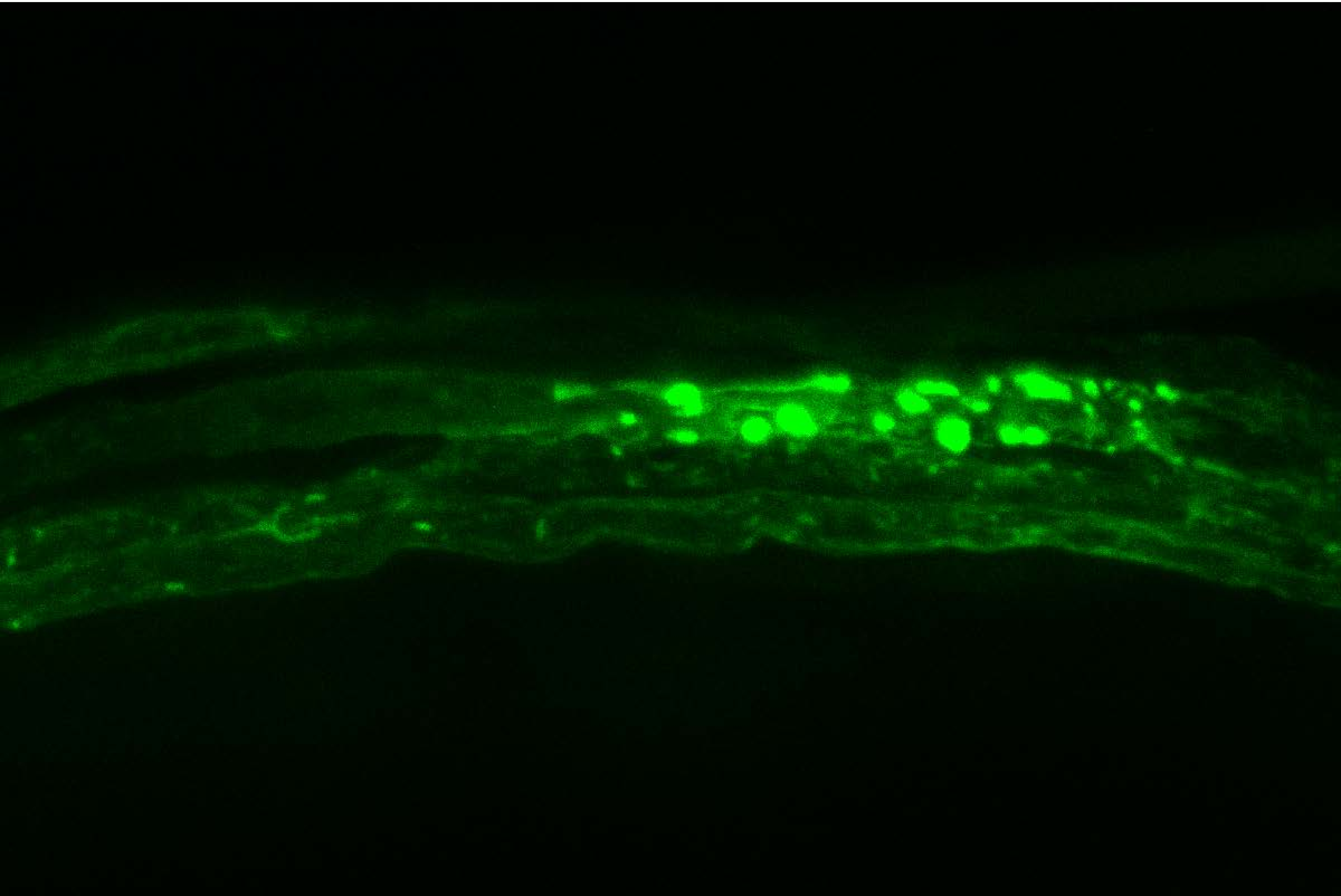

3C_1.tif

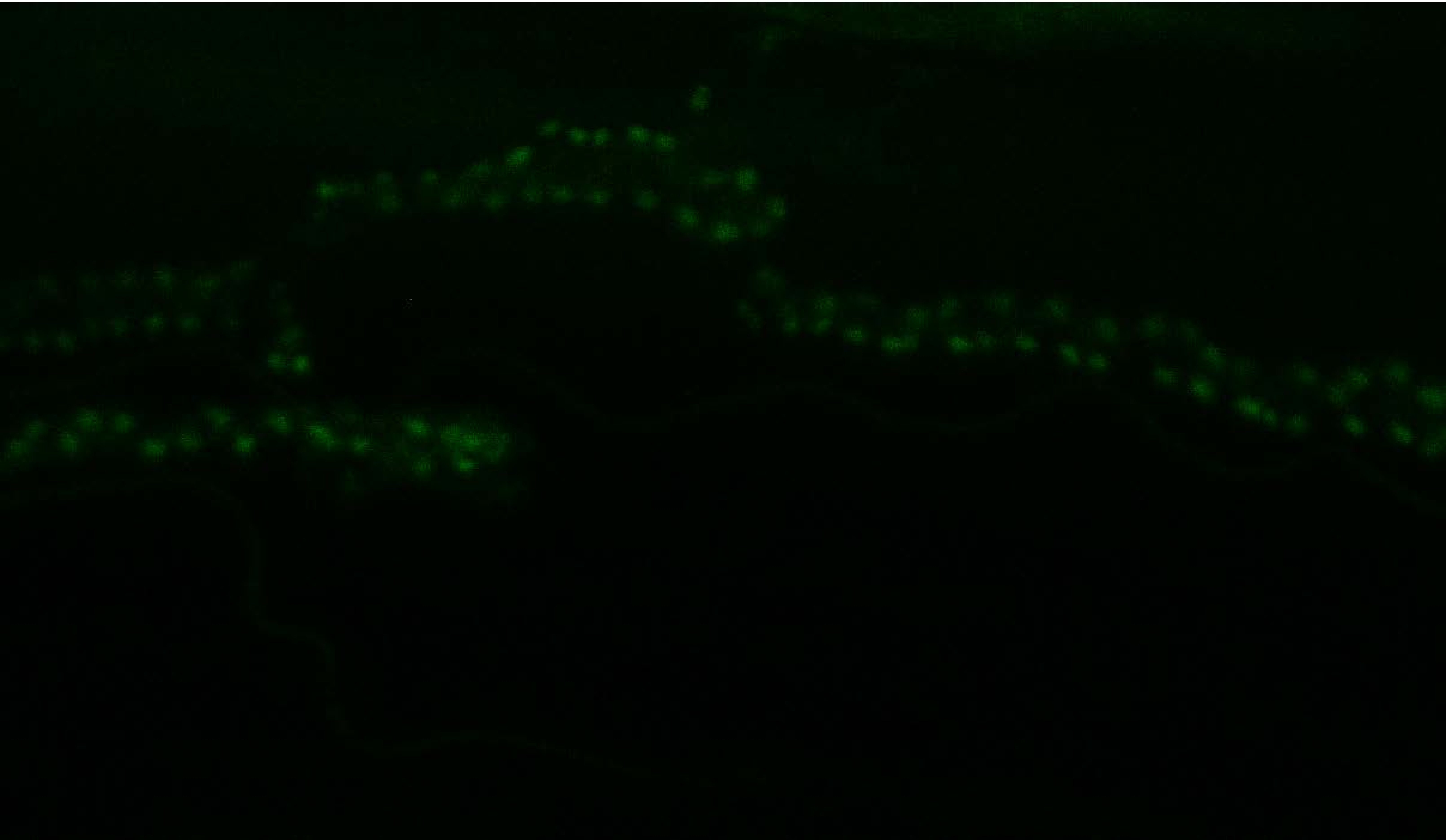

3C_2.tif

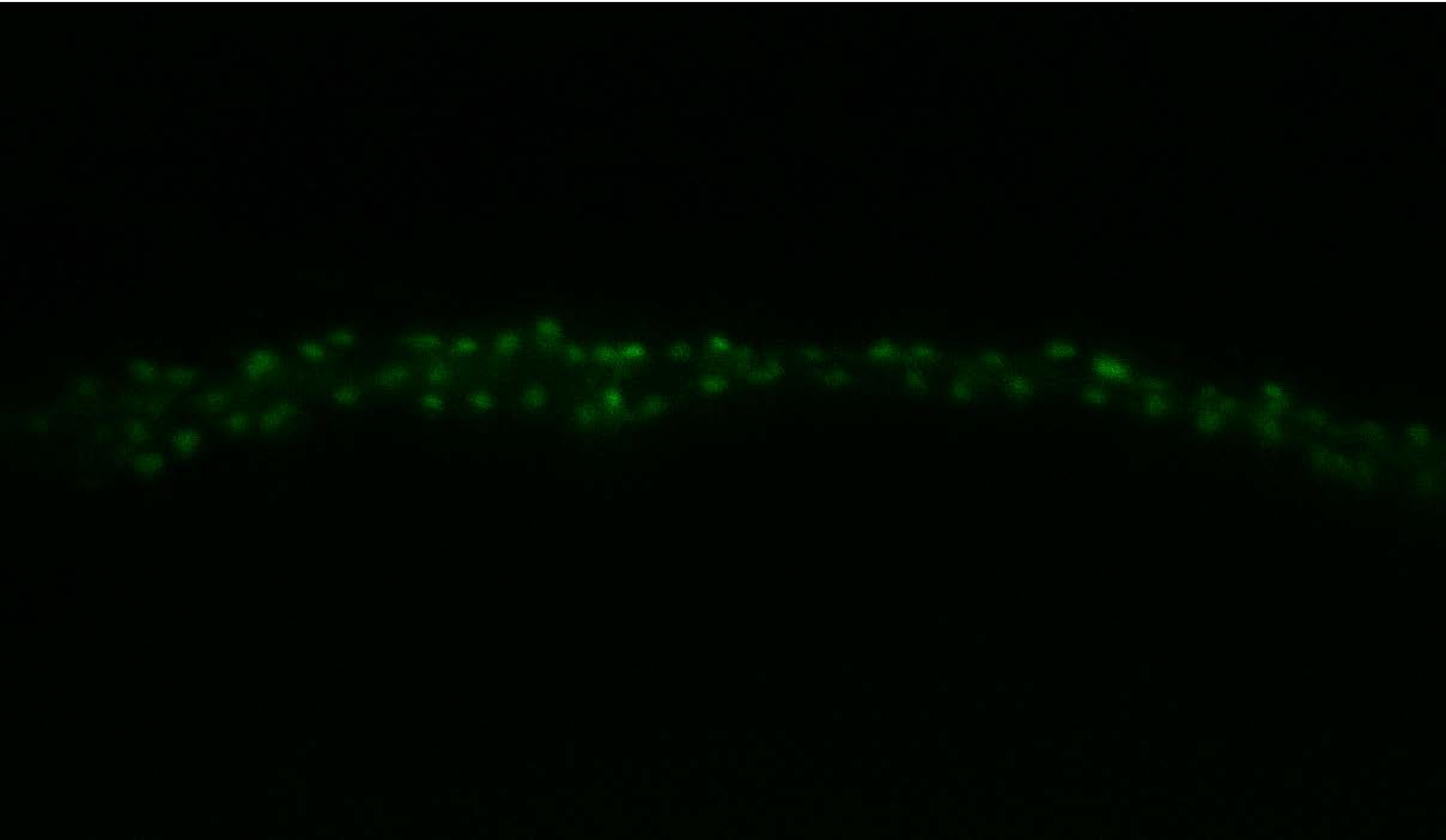

3C_3.tif

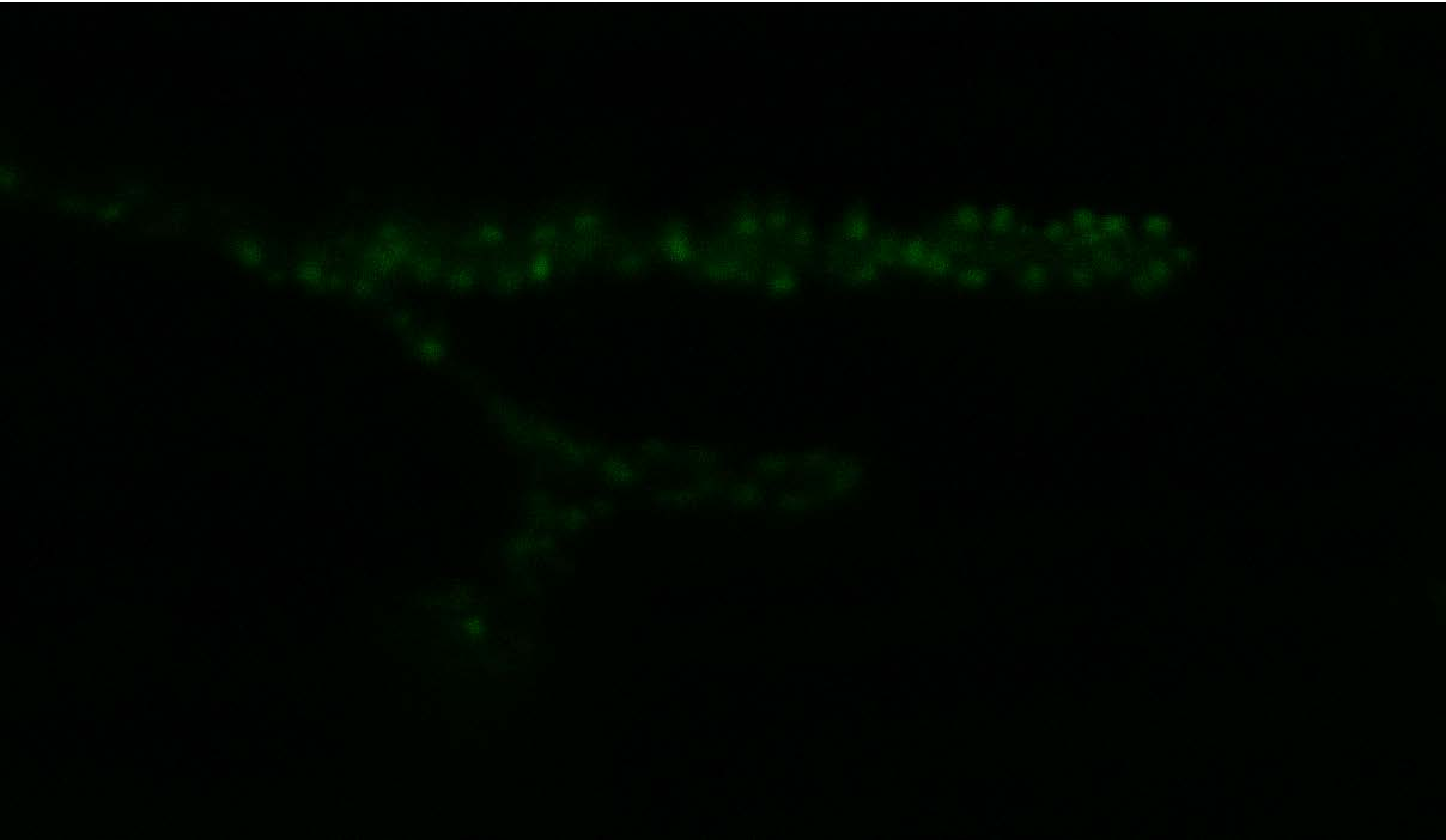

3C_4.tif

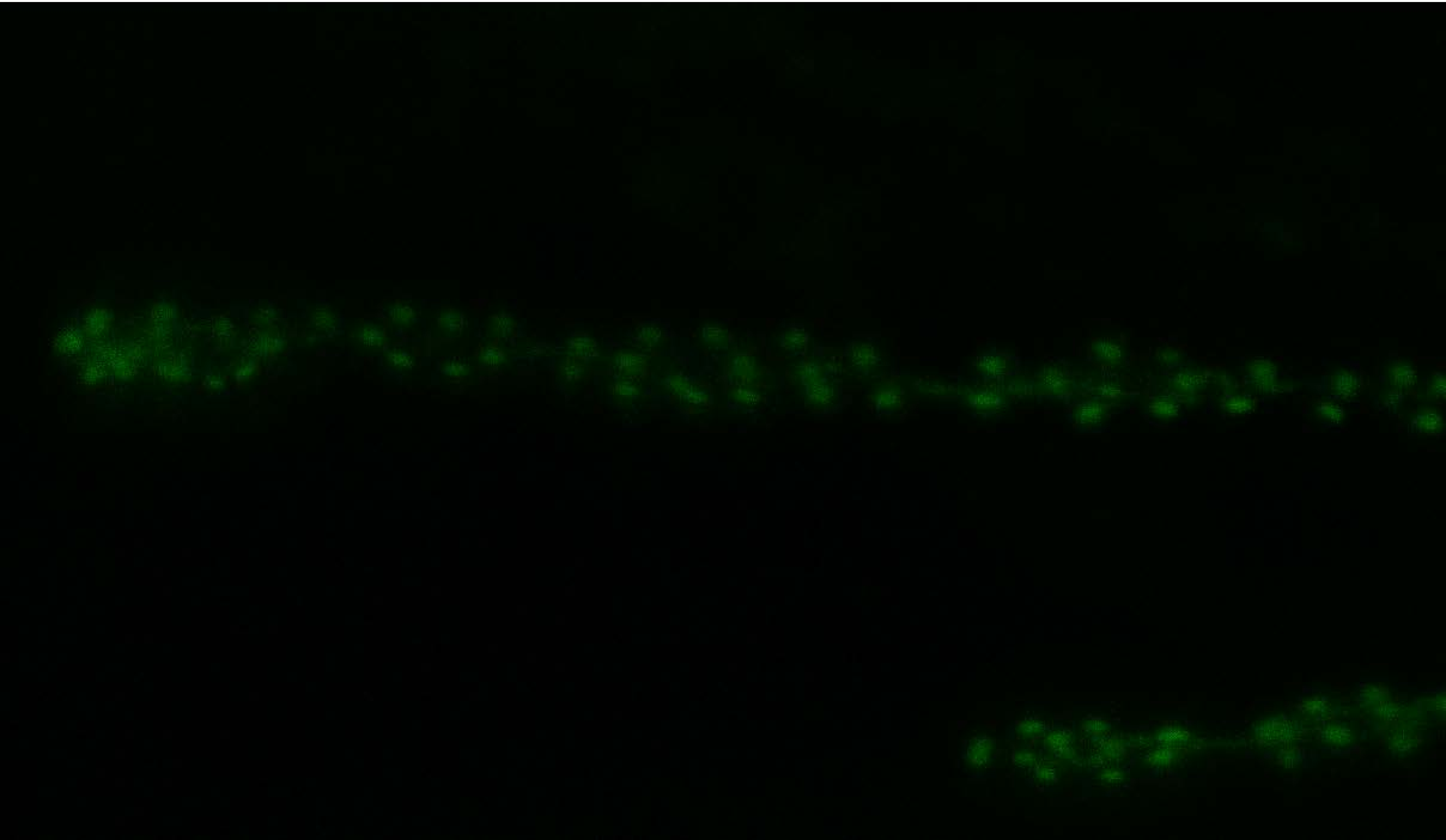

3C_5.tif

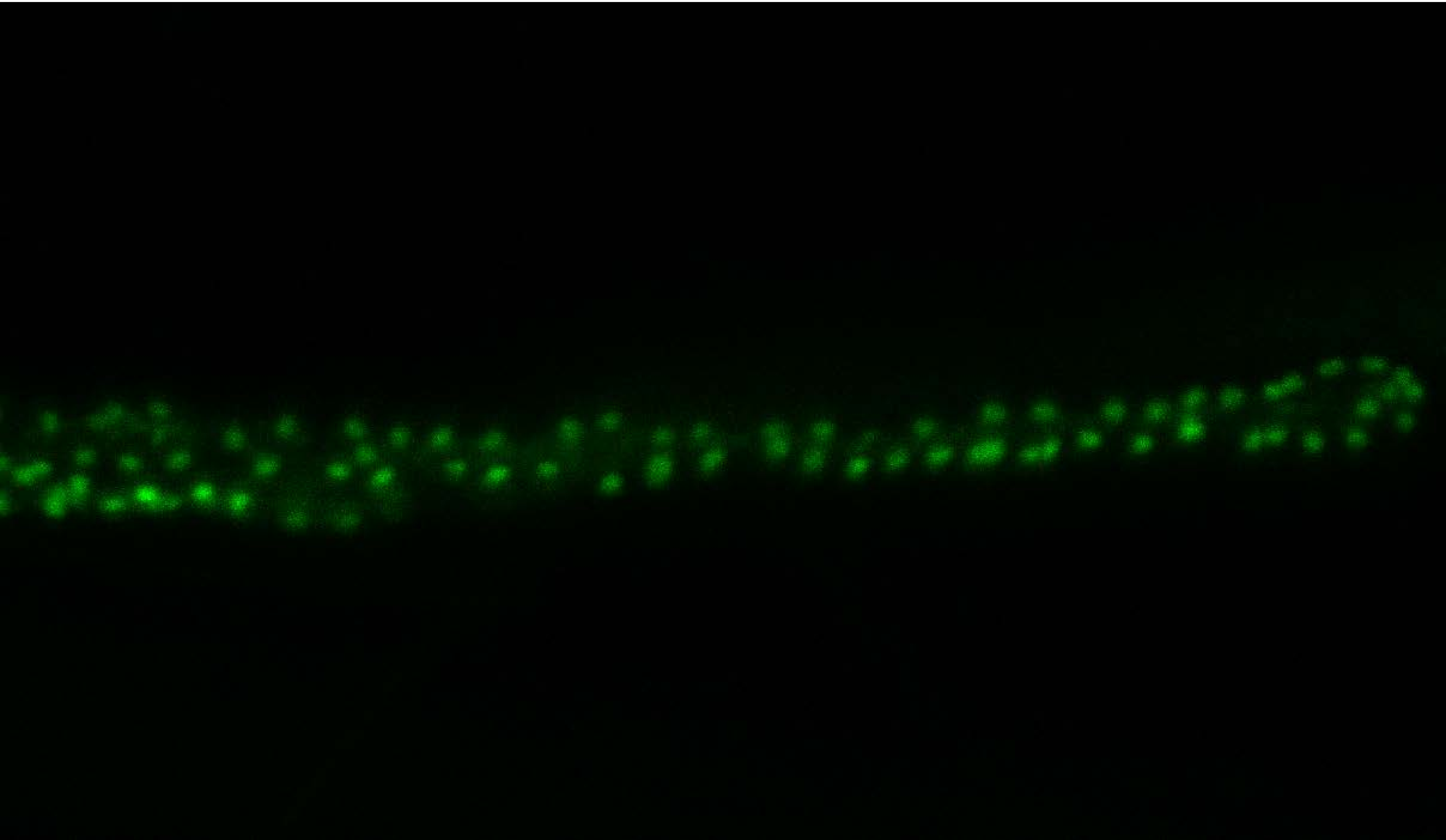

3C_6.tif

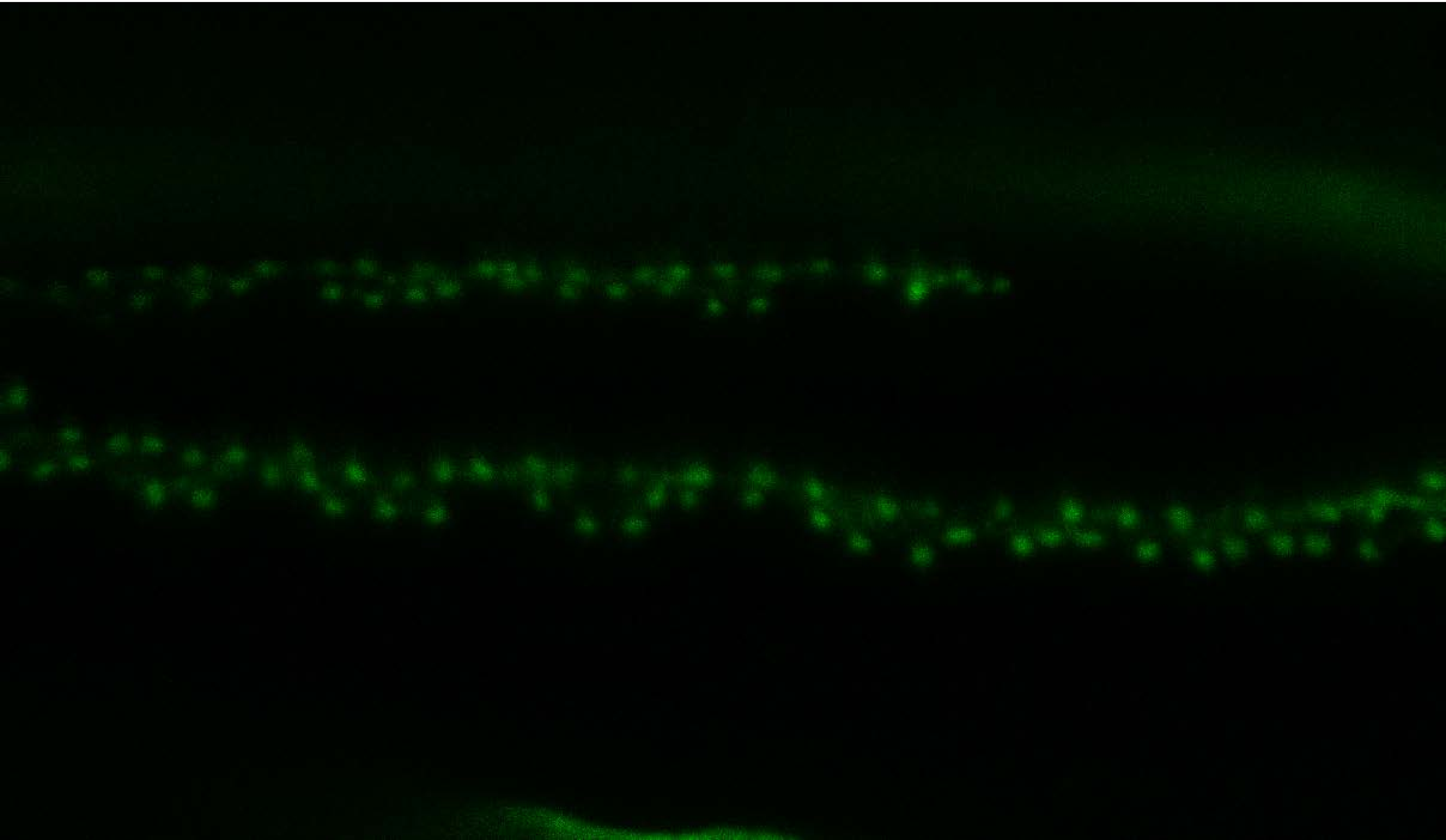

3C_7.tif

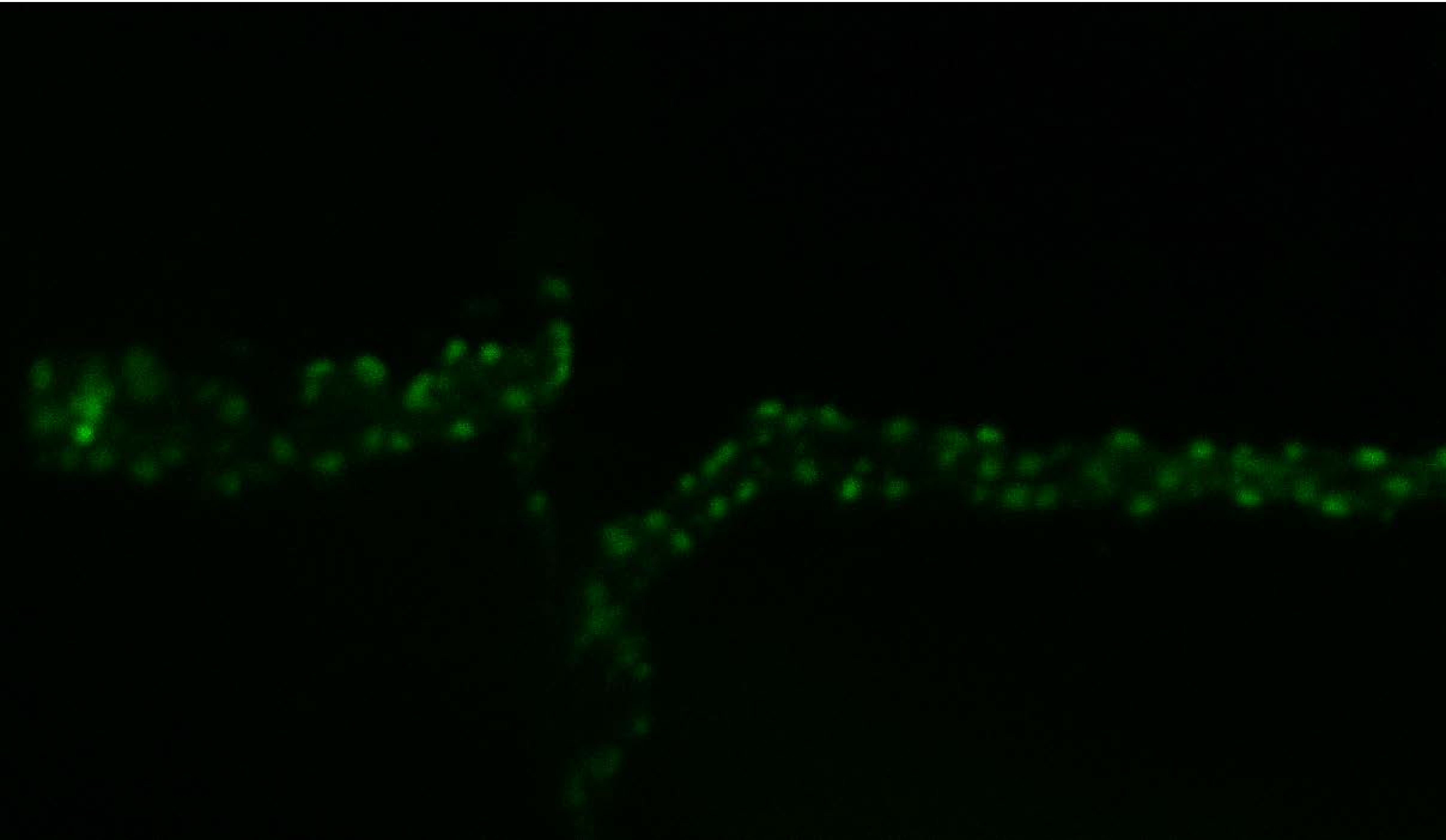

3C_8.tif

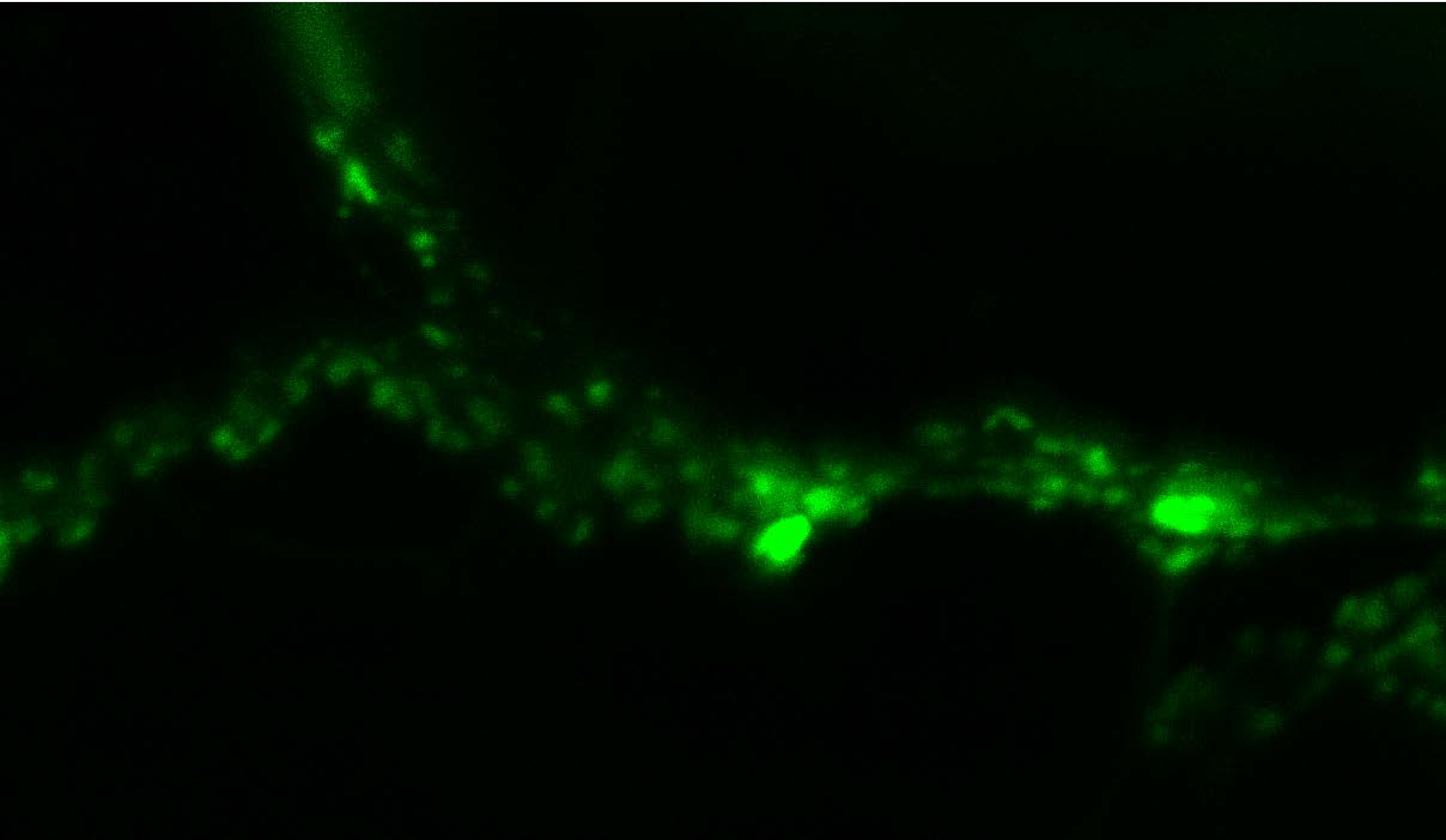

3C_9.tif

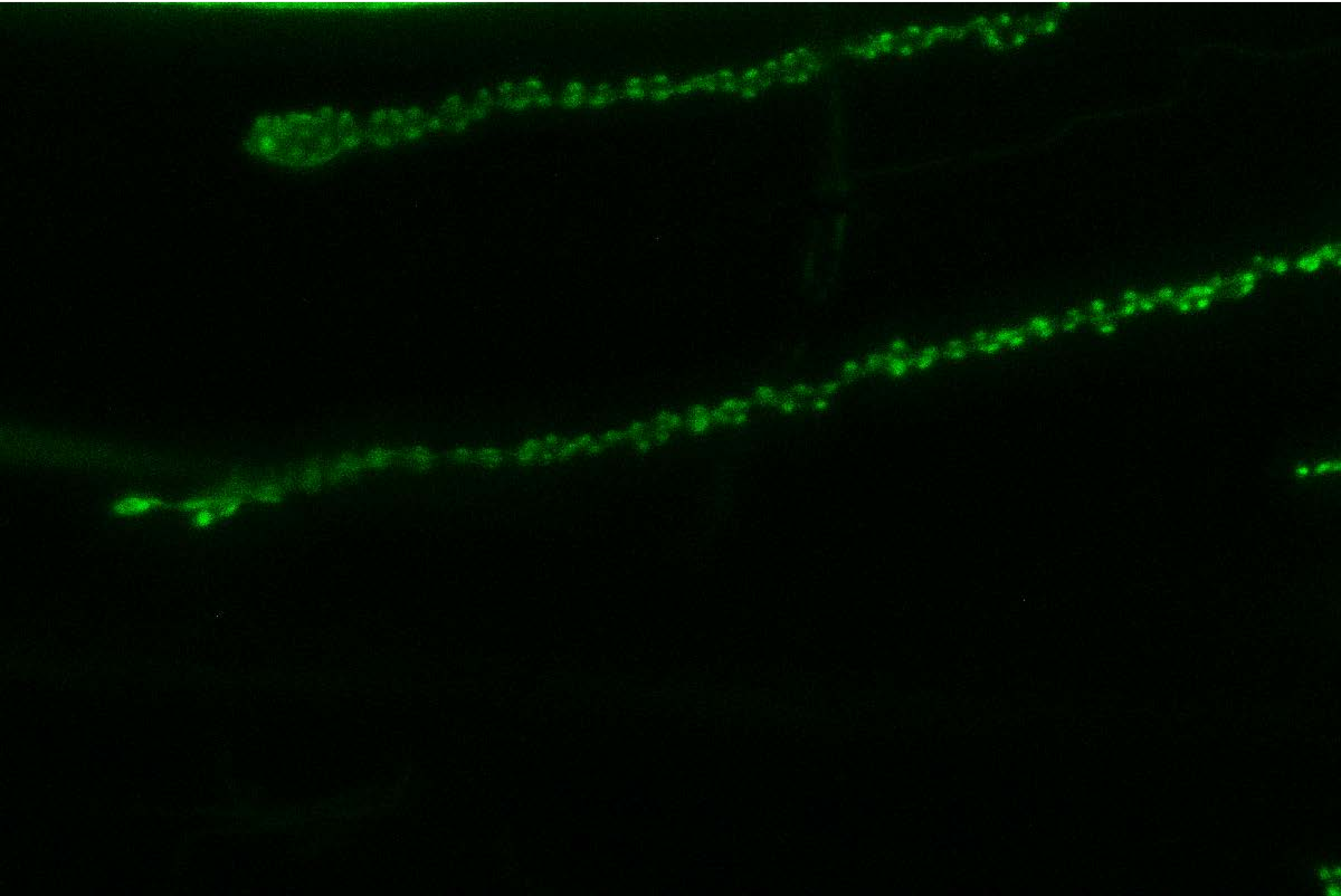

3C_10.tif

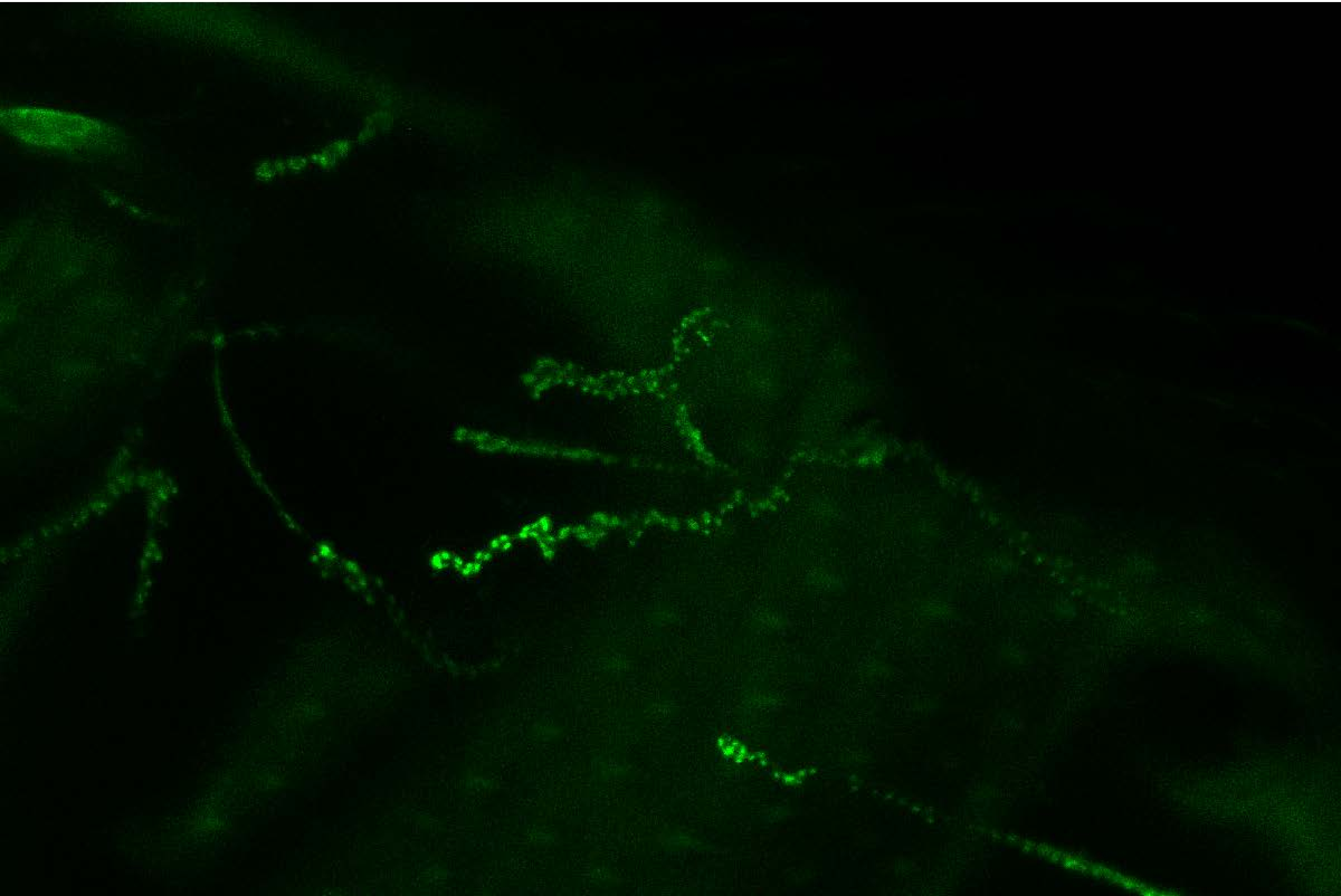

3C_11.tif

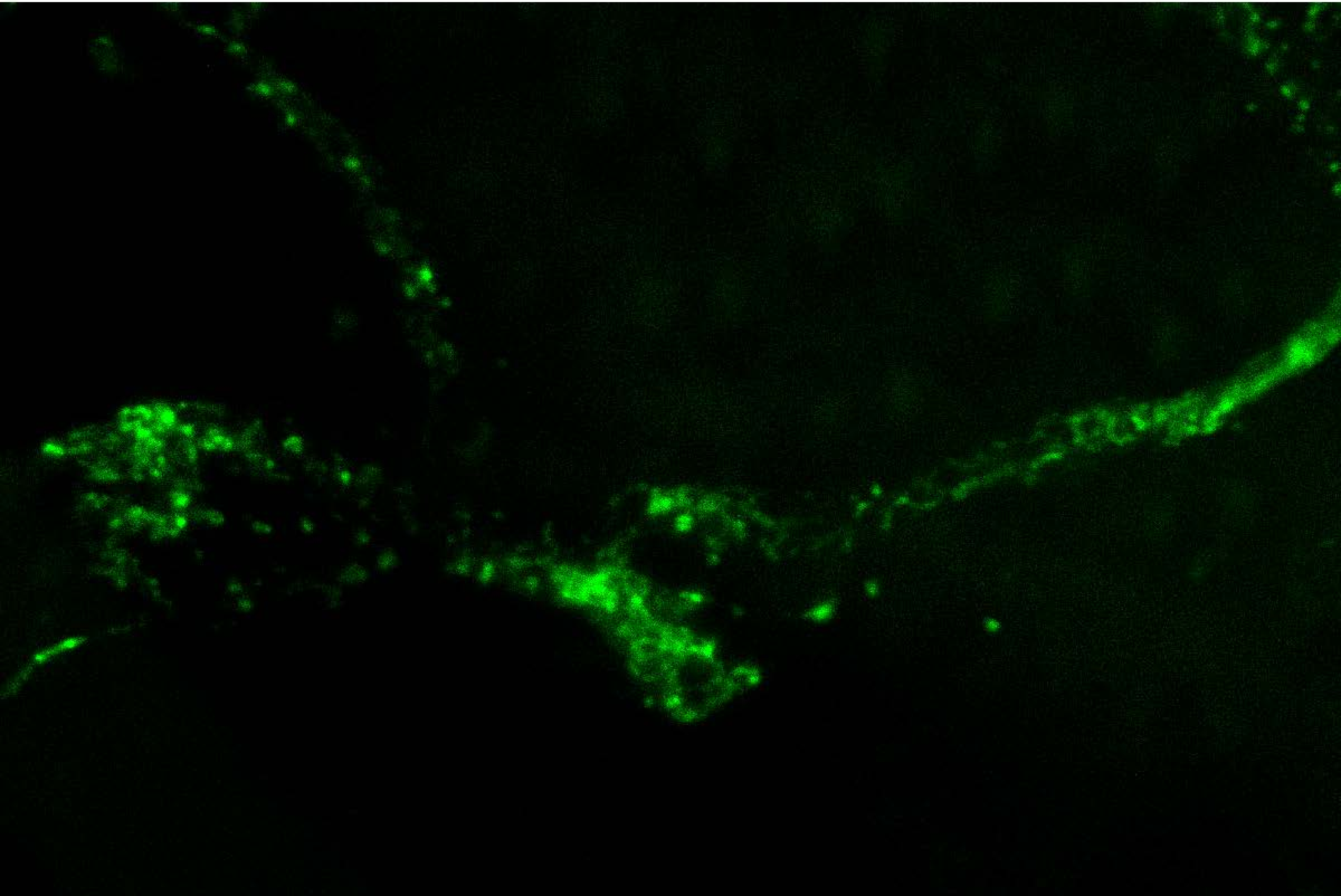

3C_12.tif

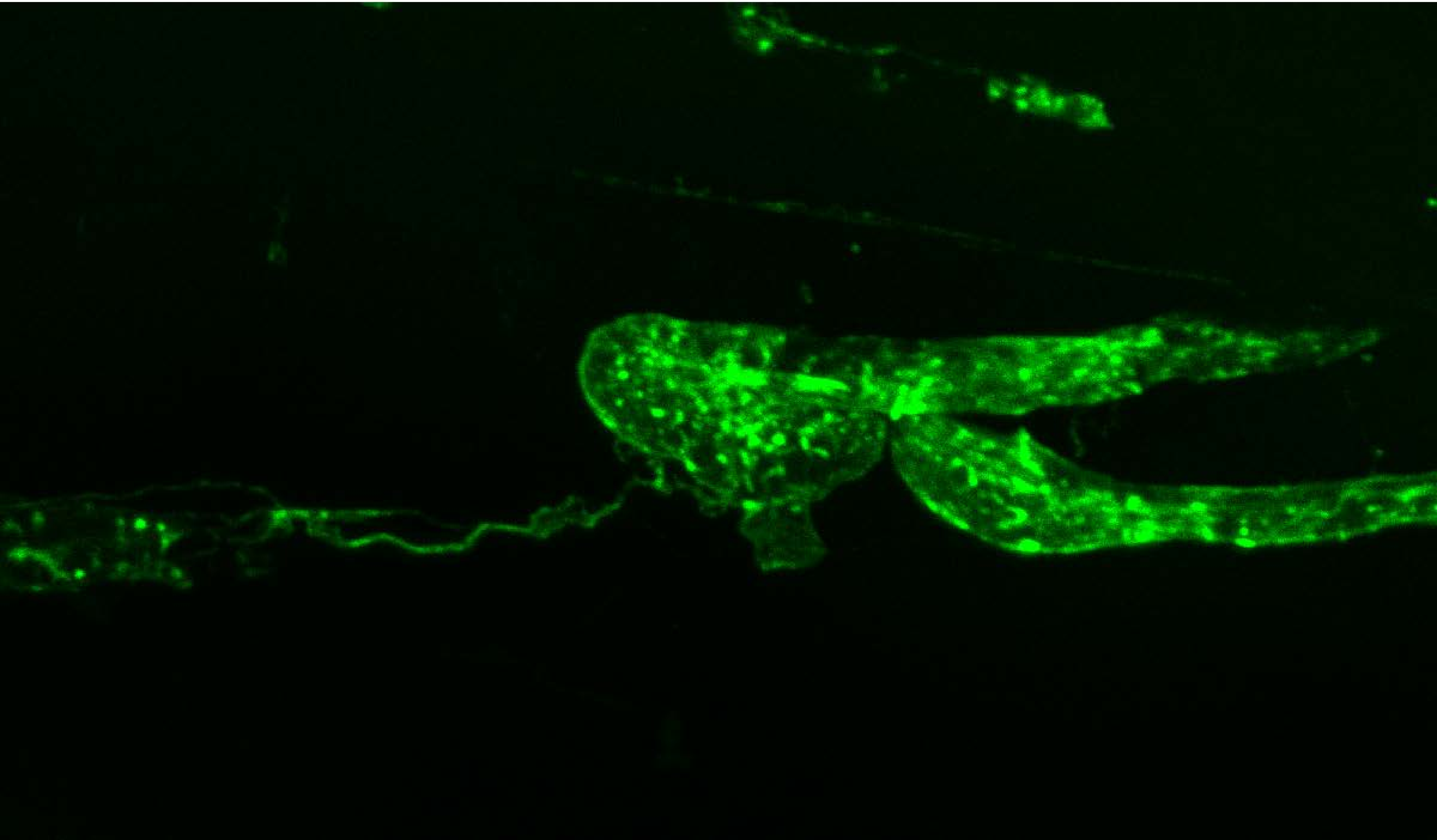

3C_13.tif

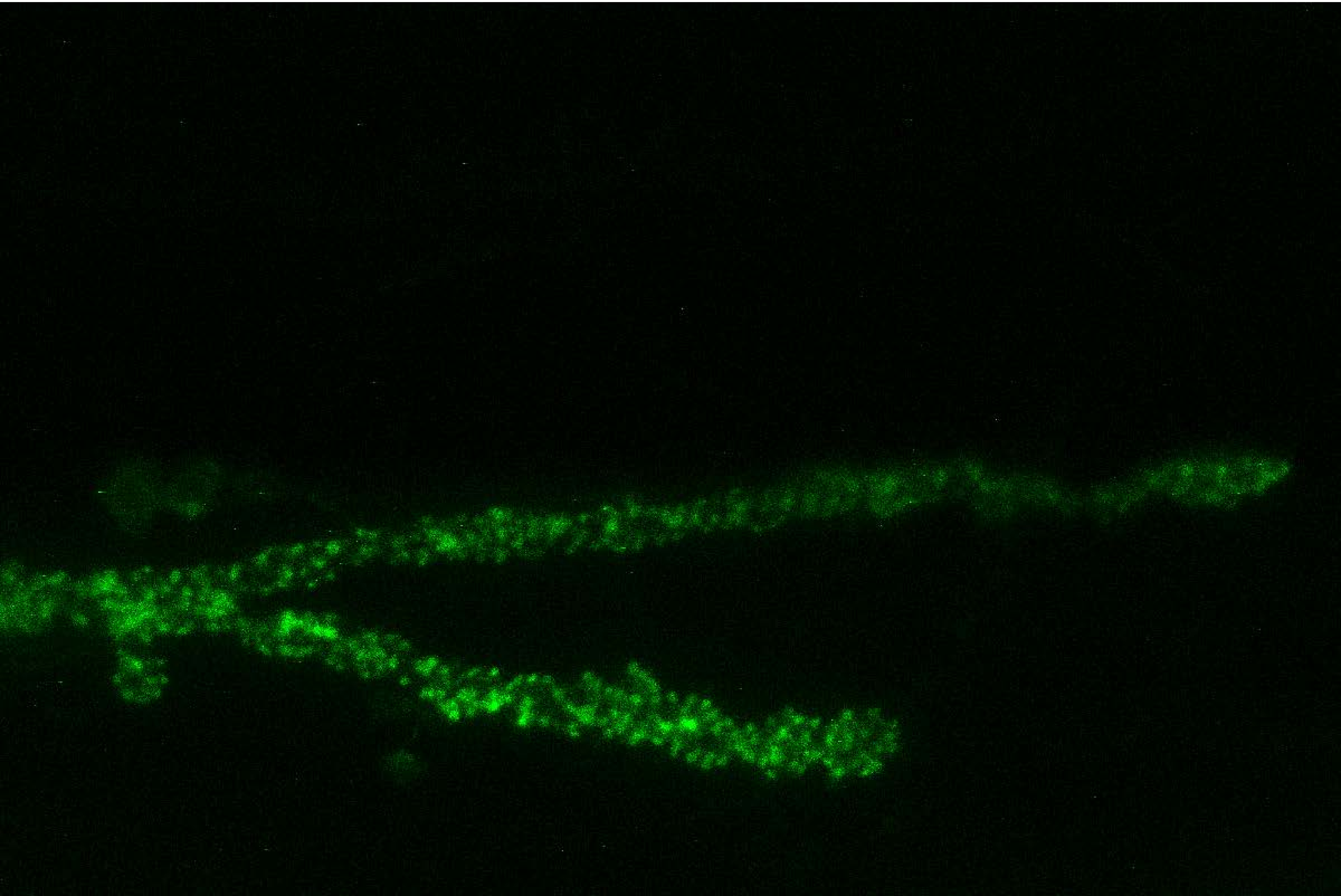

3C_14.tif

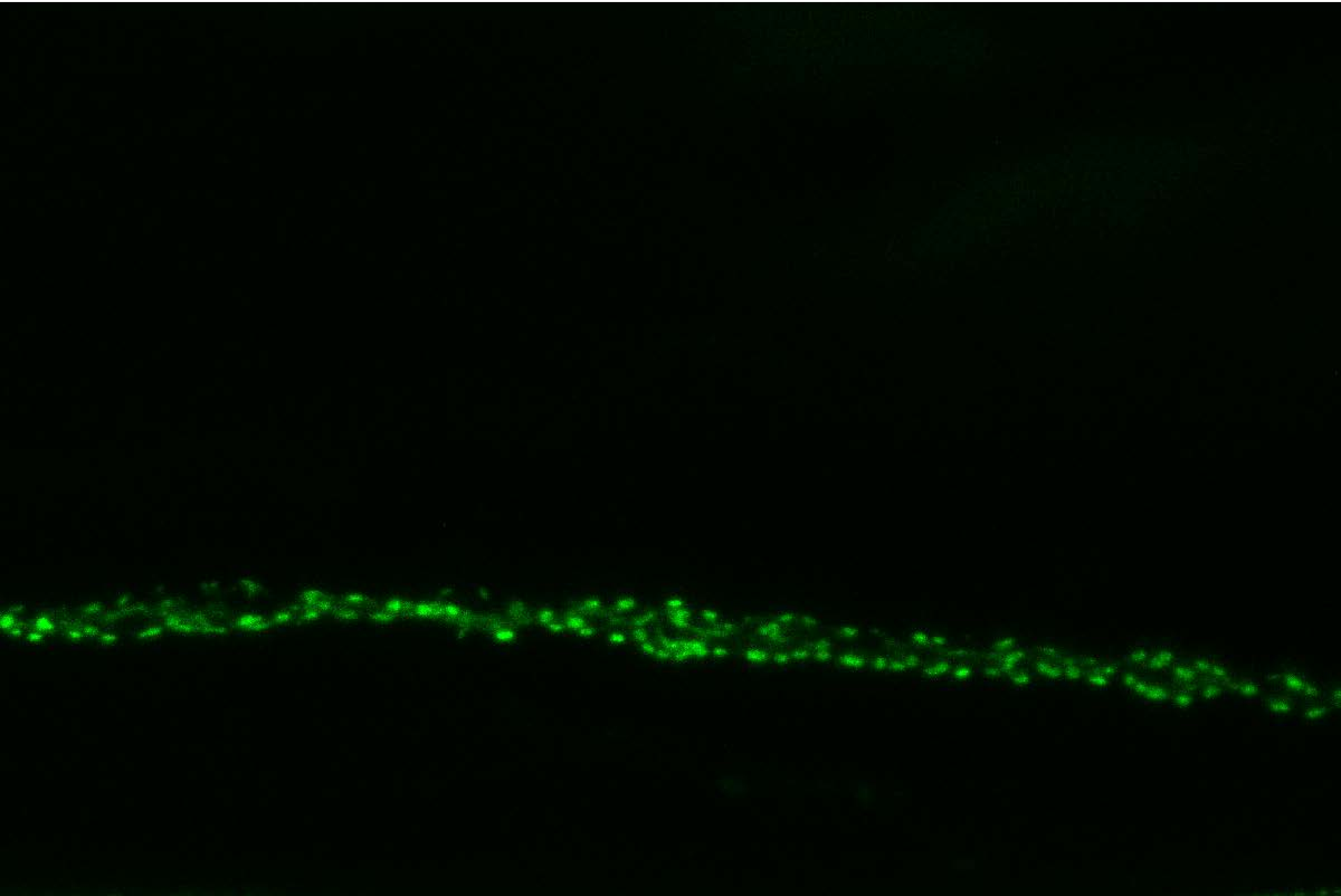

3C_15.tif

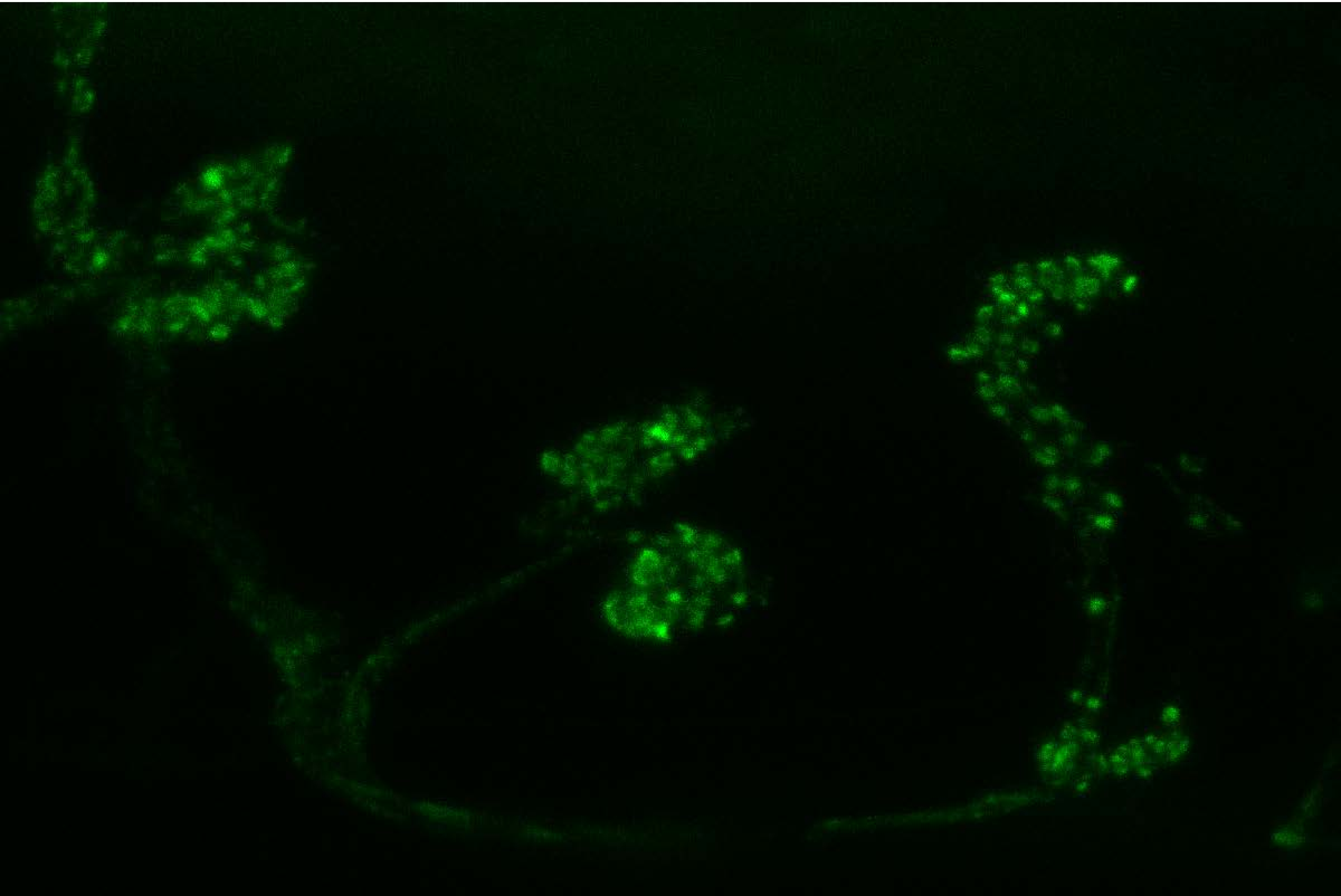

3C_16.tif

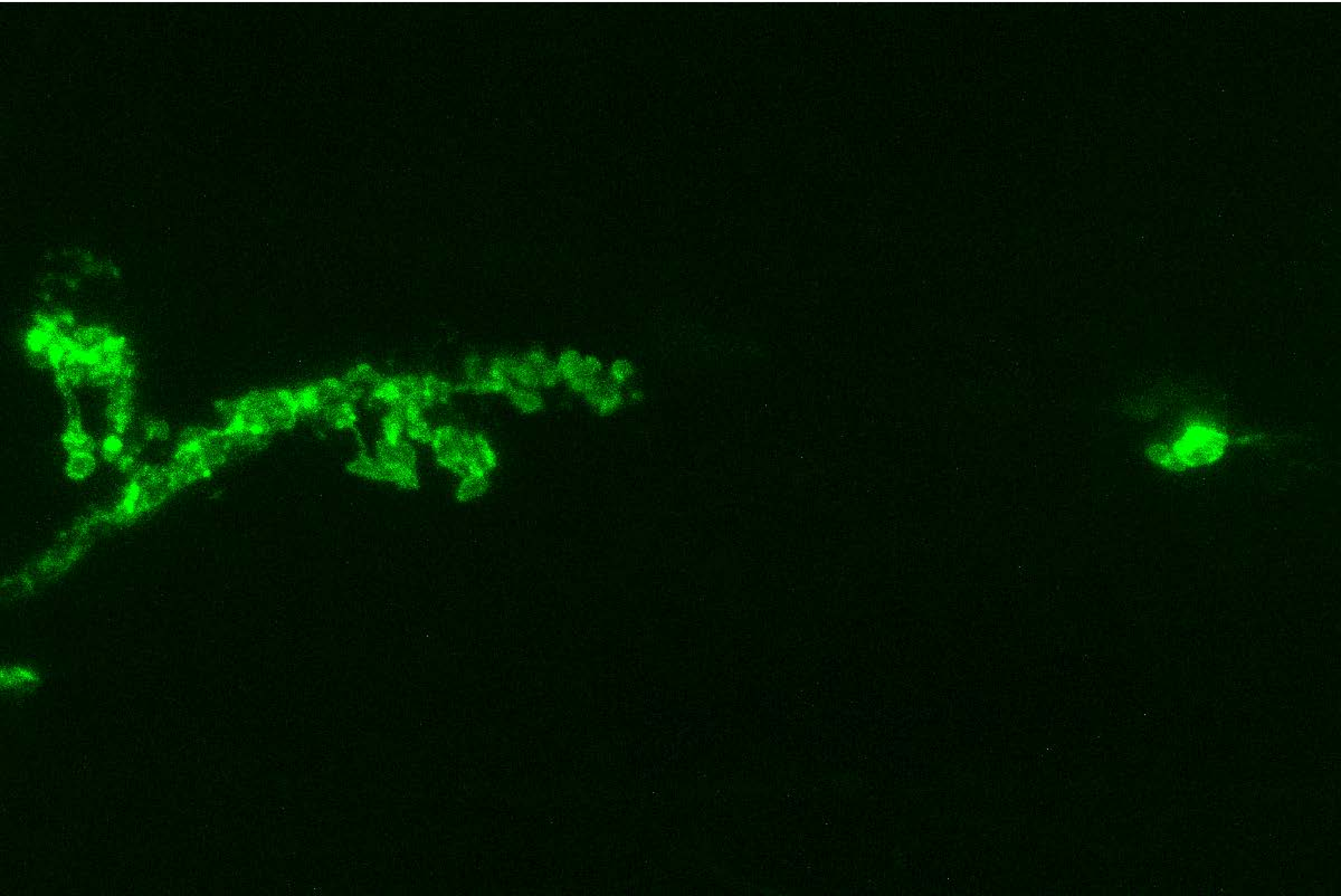

3E_1.tif

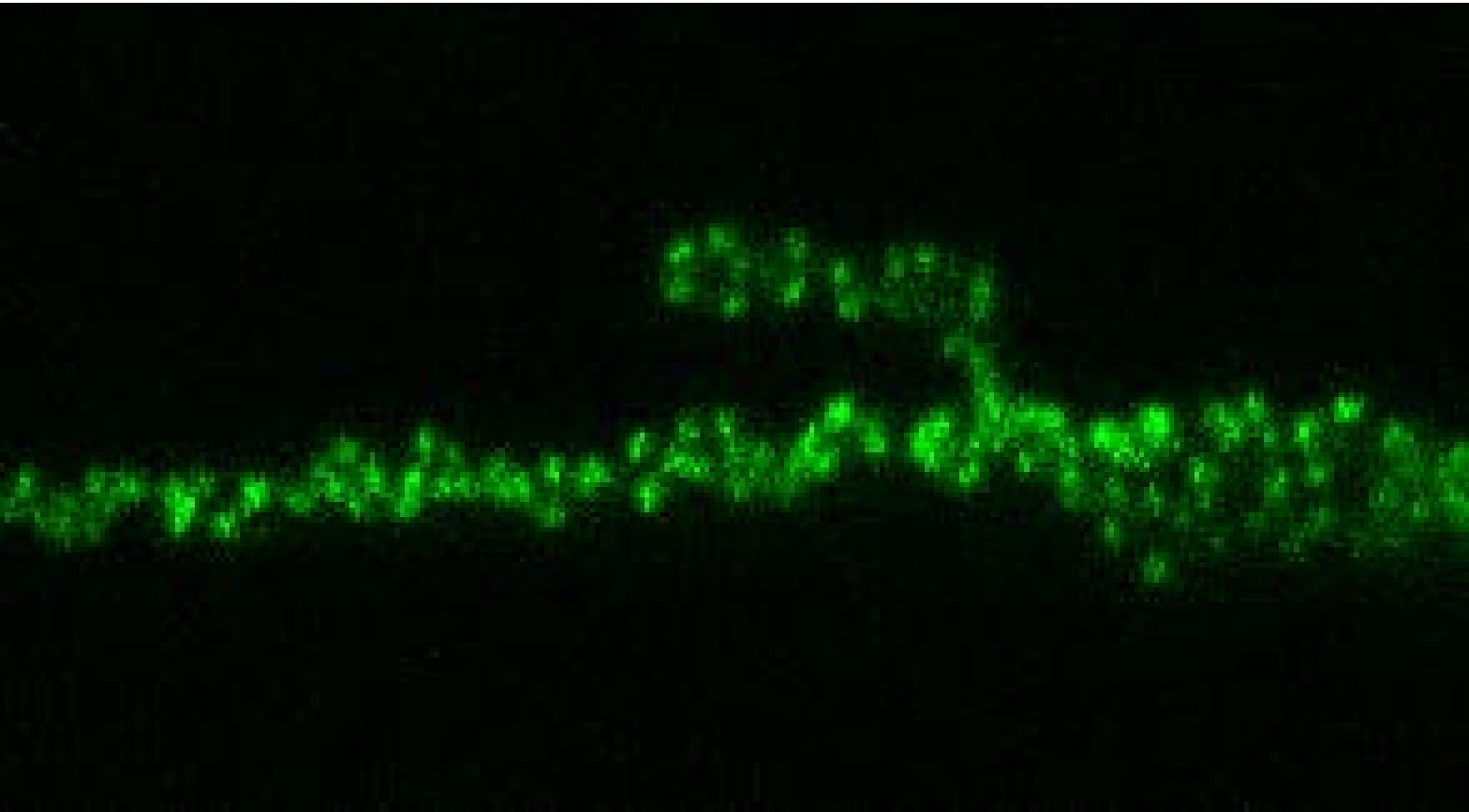

3E_2.tif

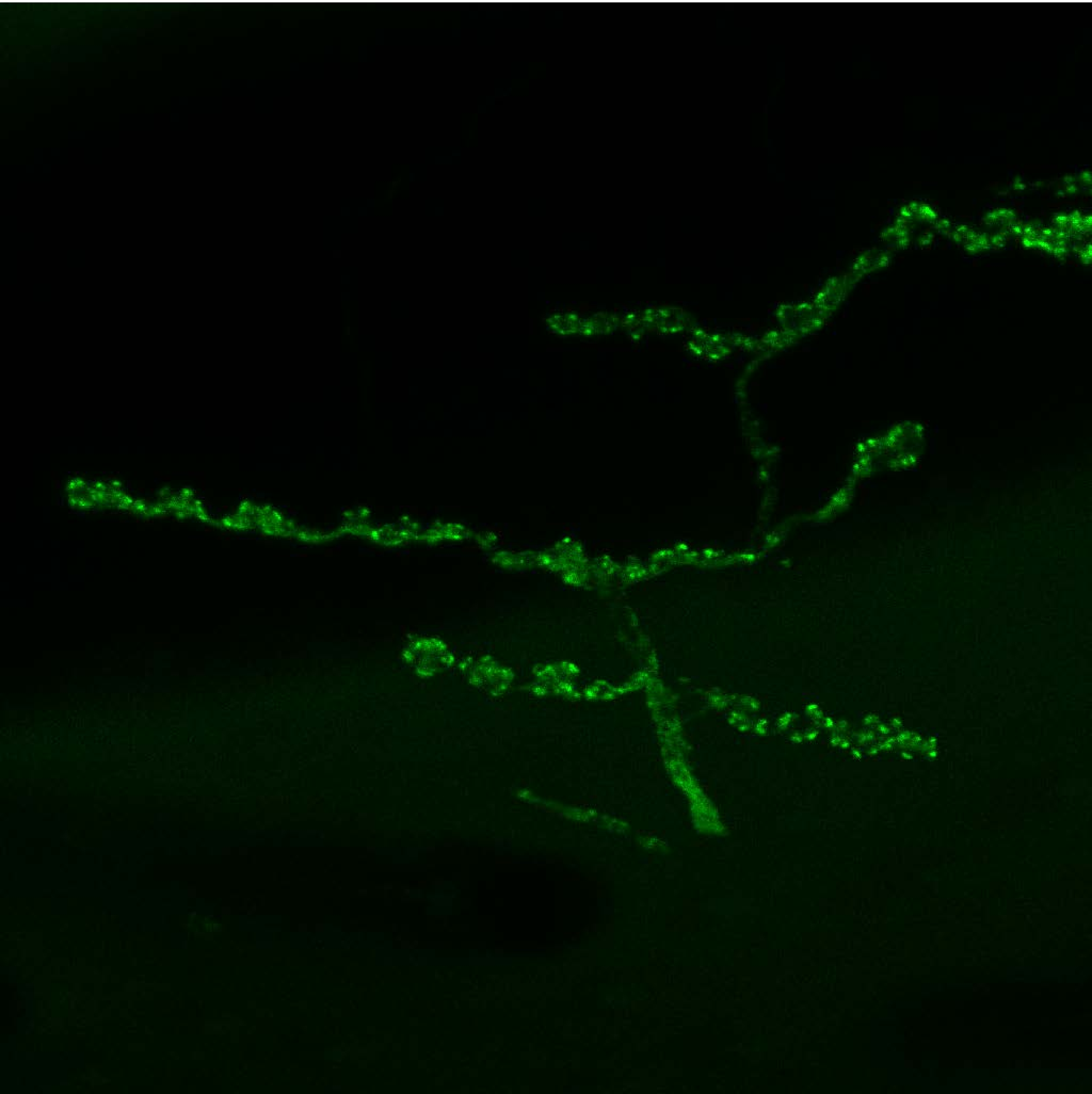

3E_3.tif

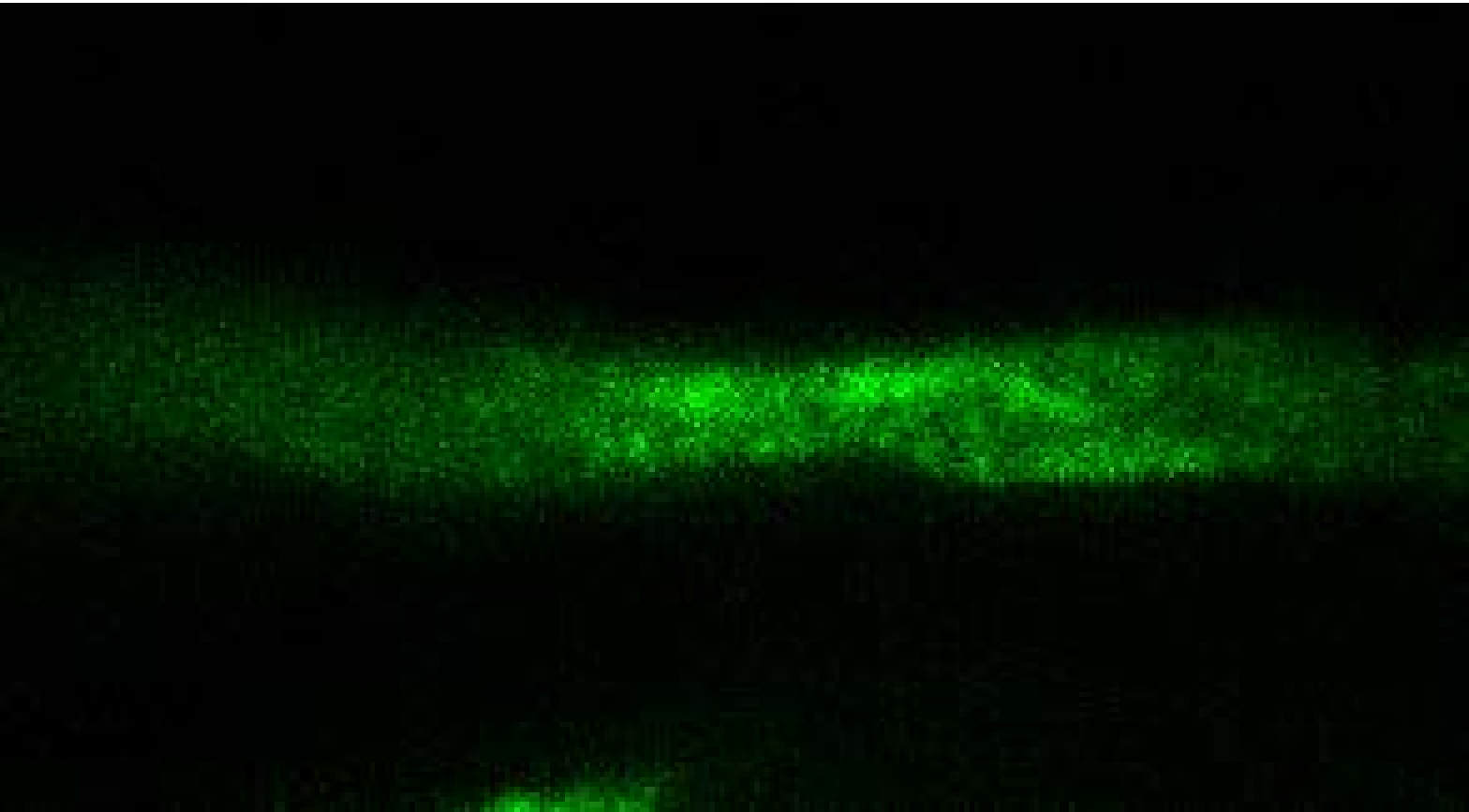

3E_4.tif

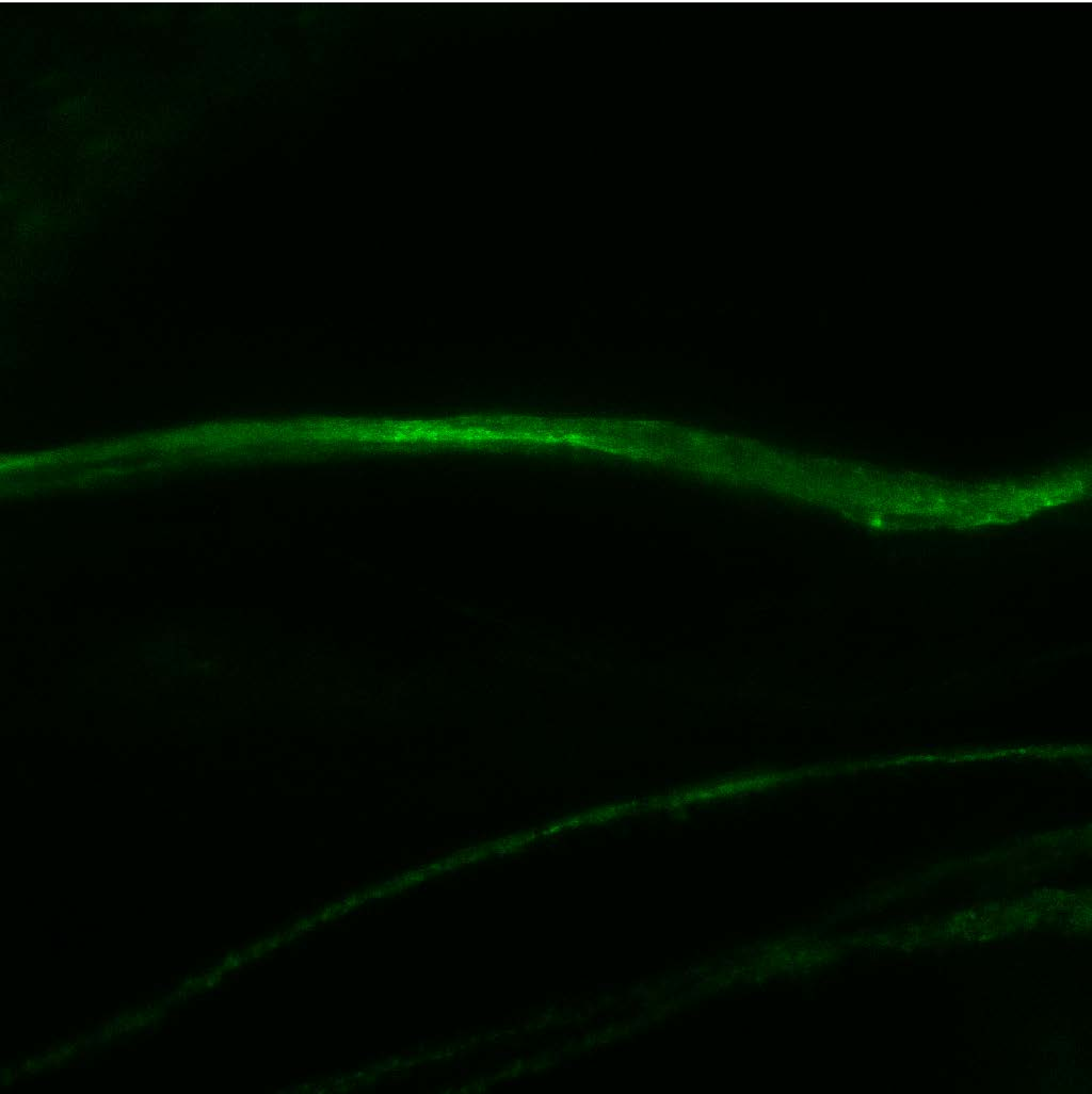

4A_1.tif

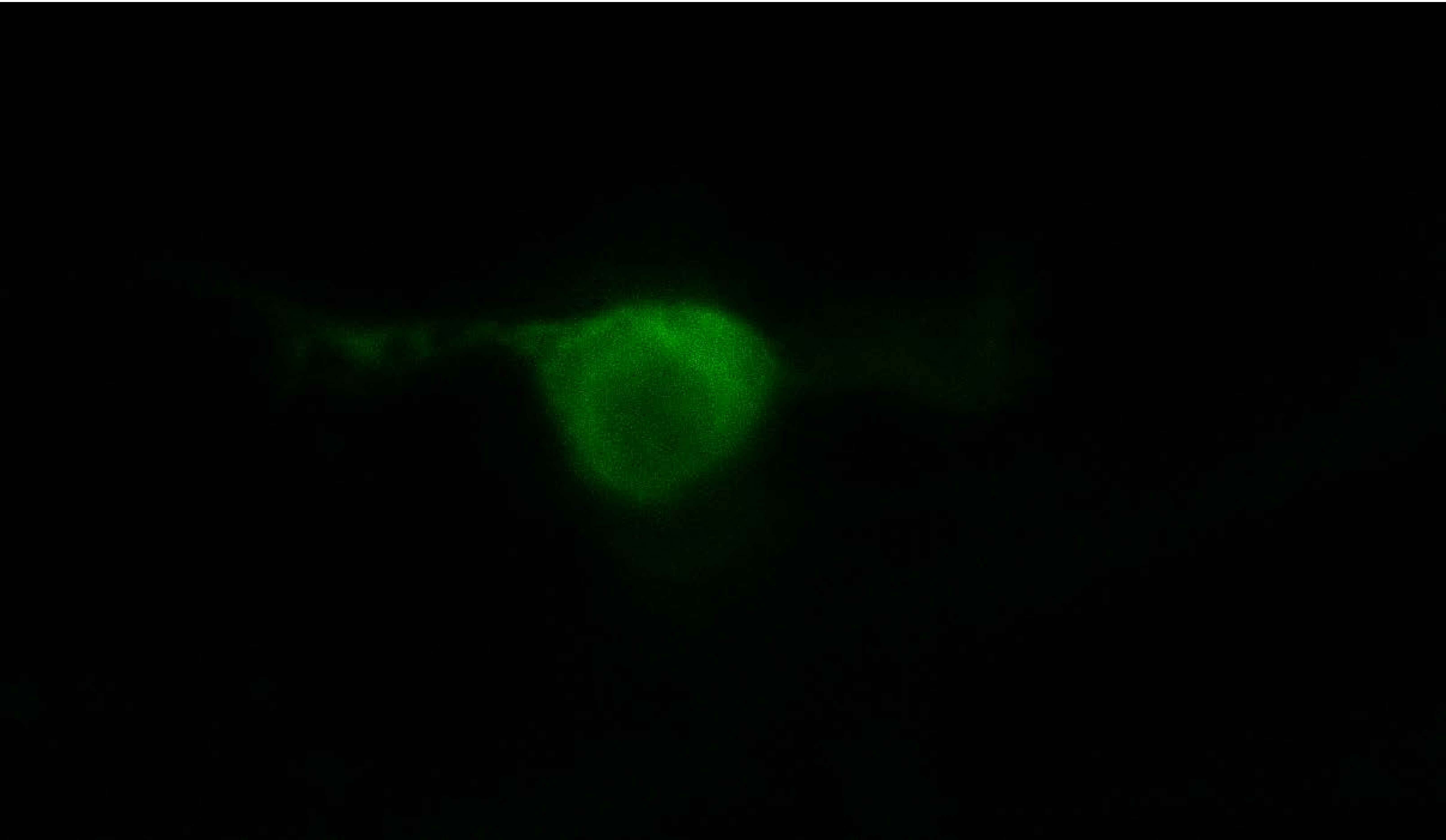

4A_2.tif

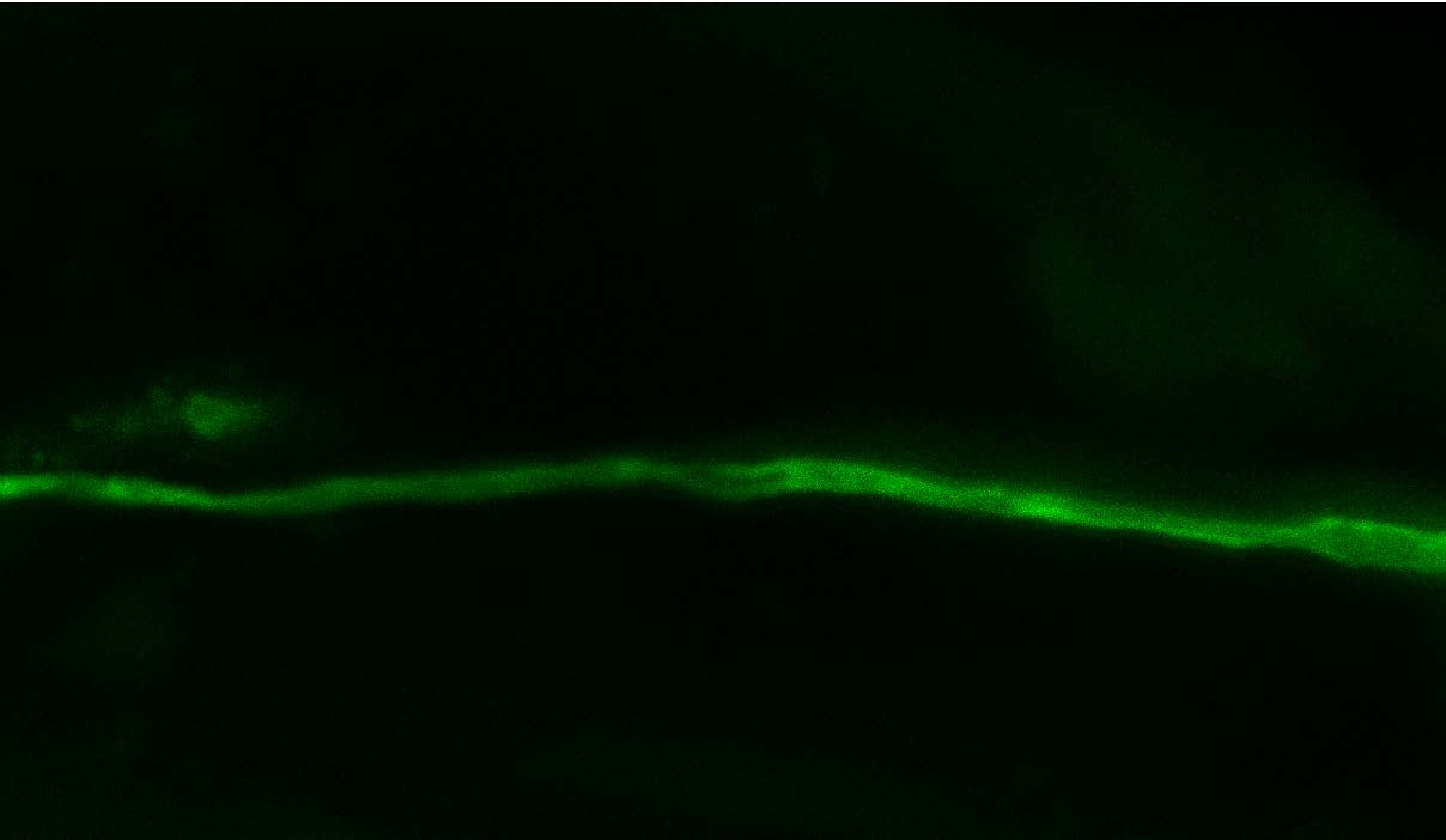

4A_3.tif

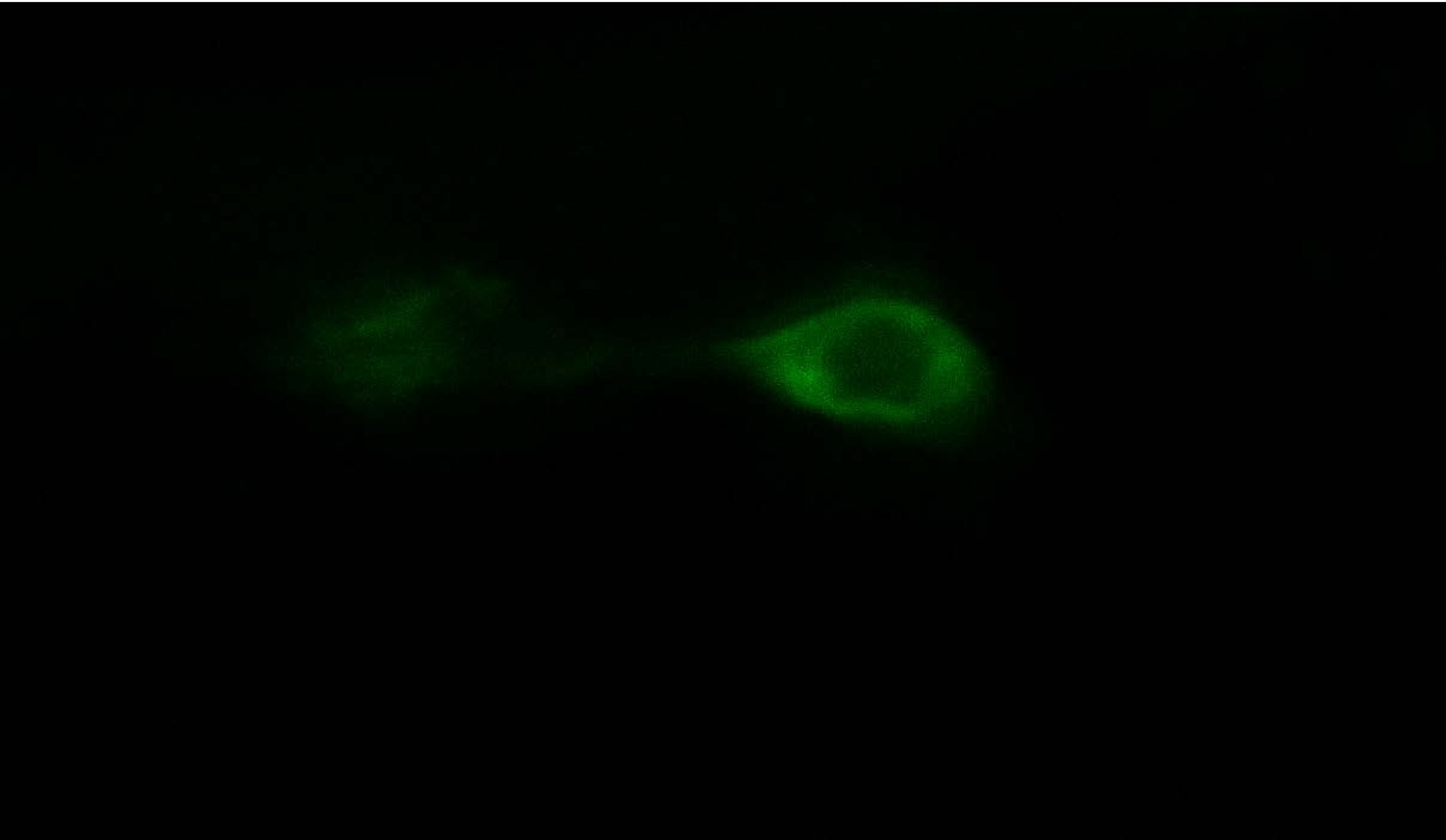

S3D_4.tif

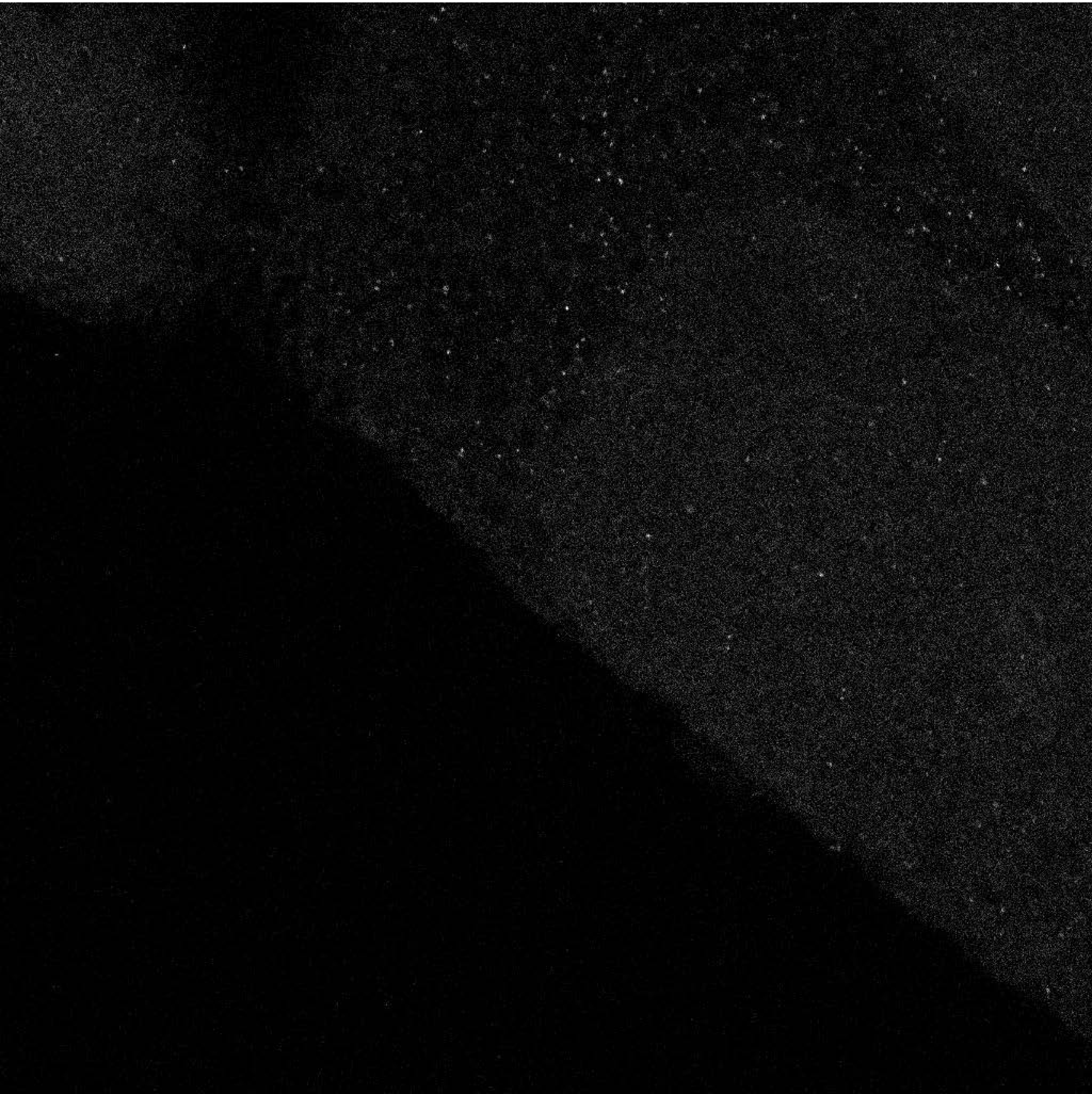

S3D_5.tif

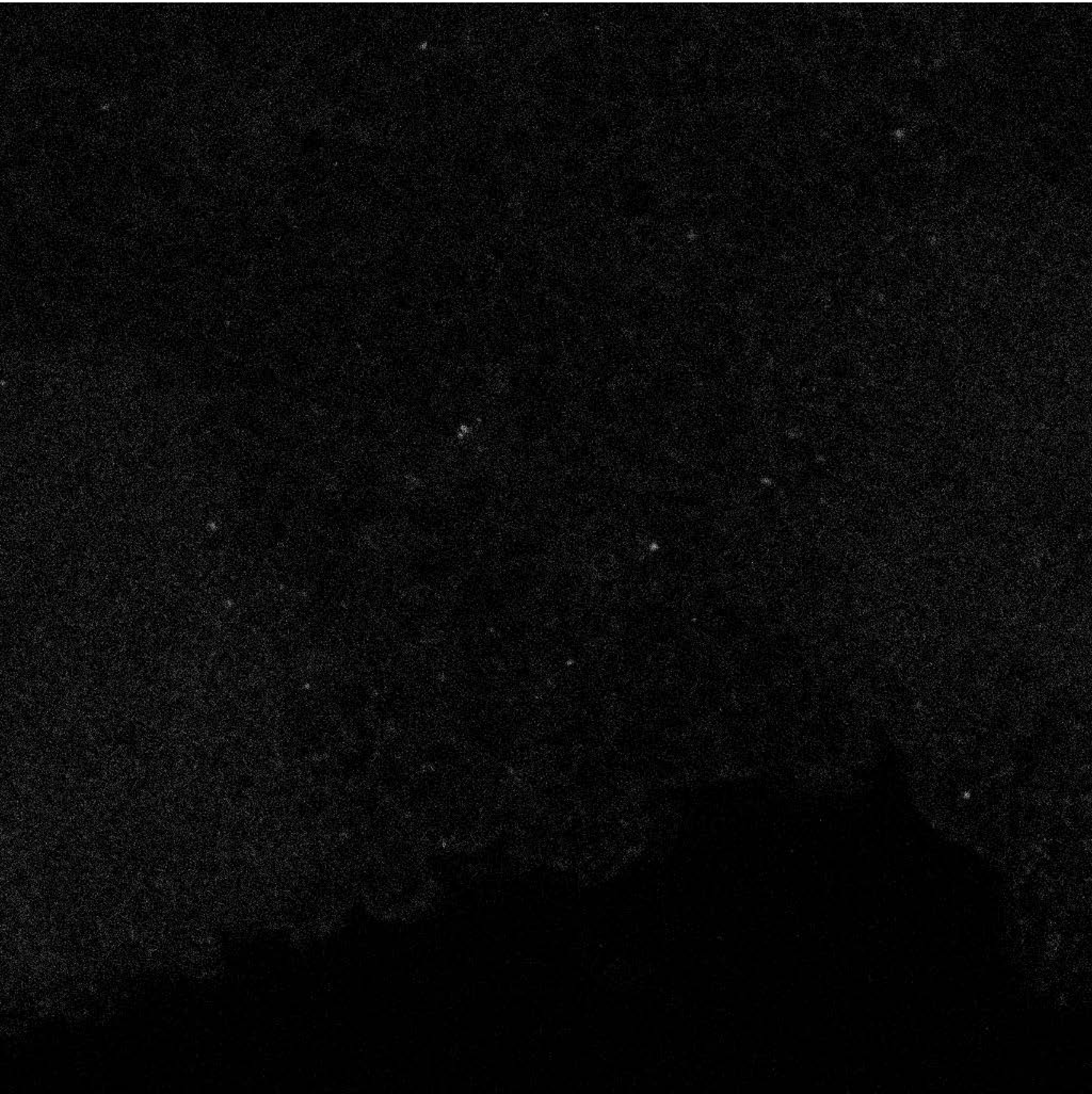

S3D_6.tif

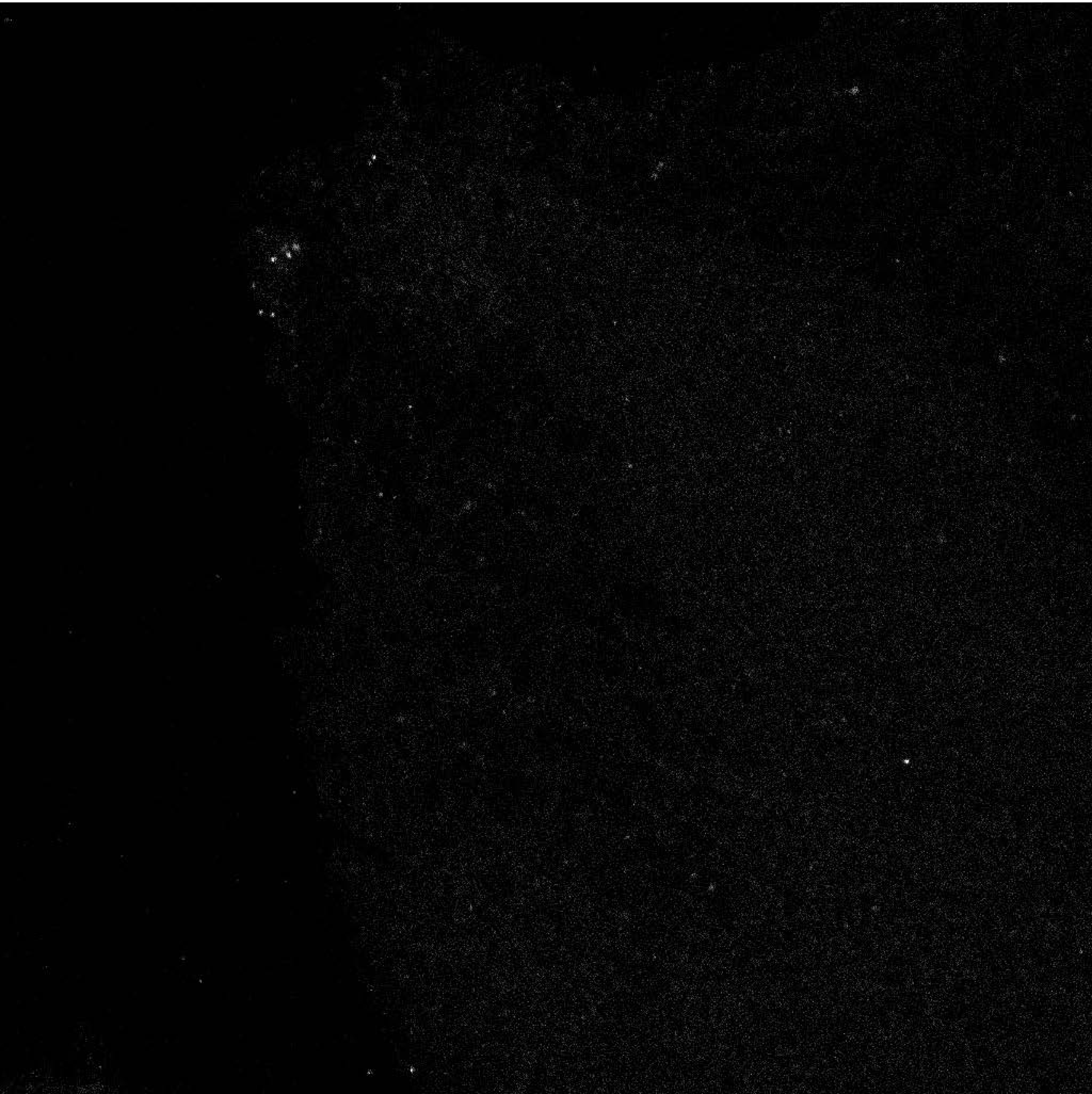

S4B_1.tif

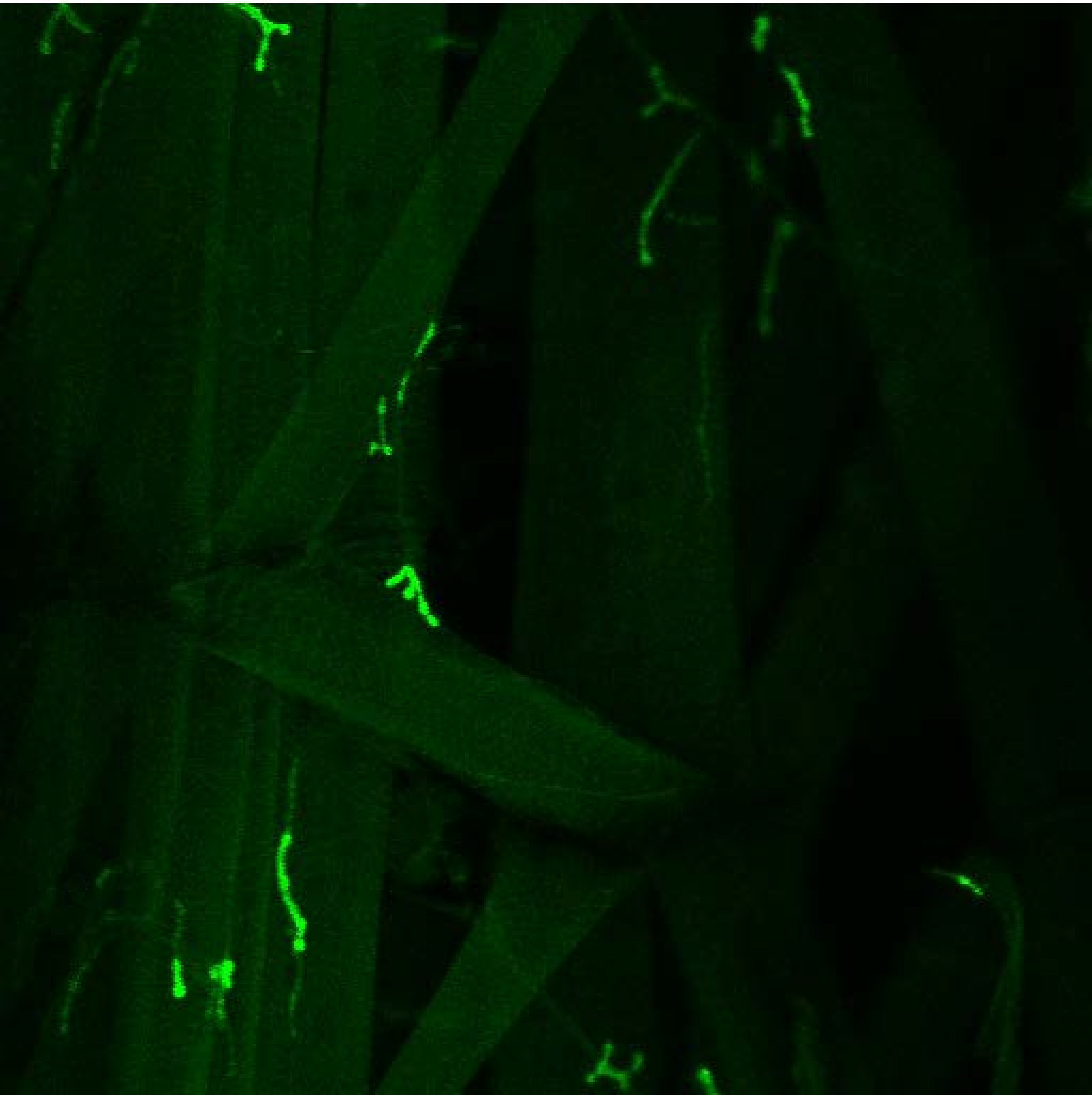

S4B_2.tif

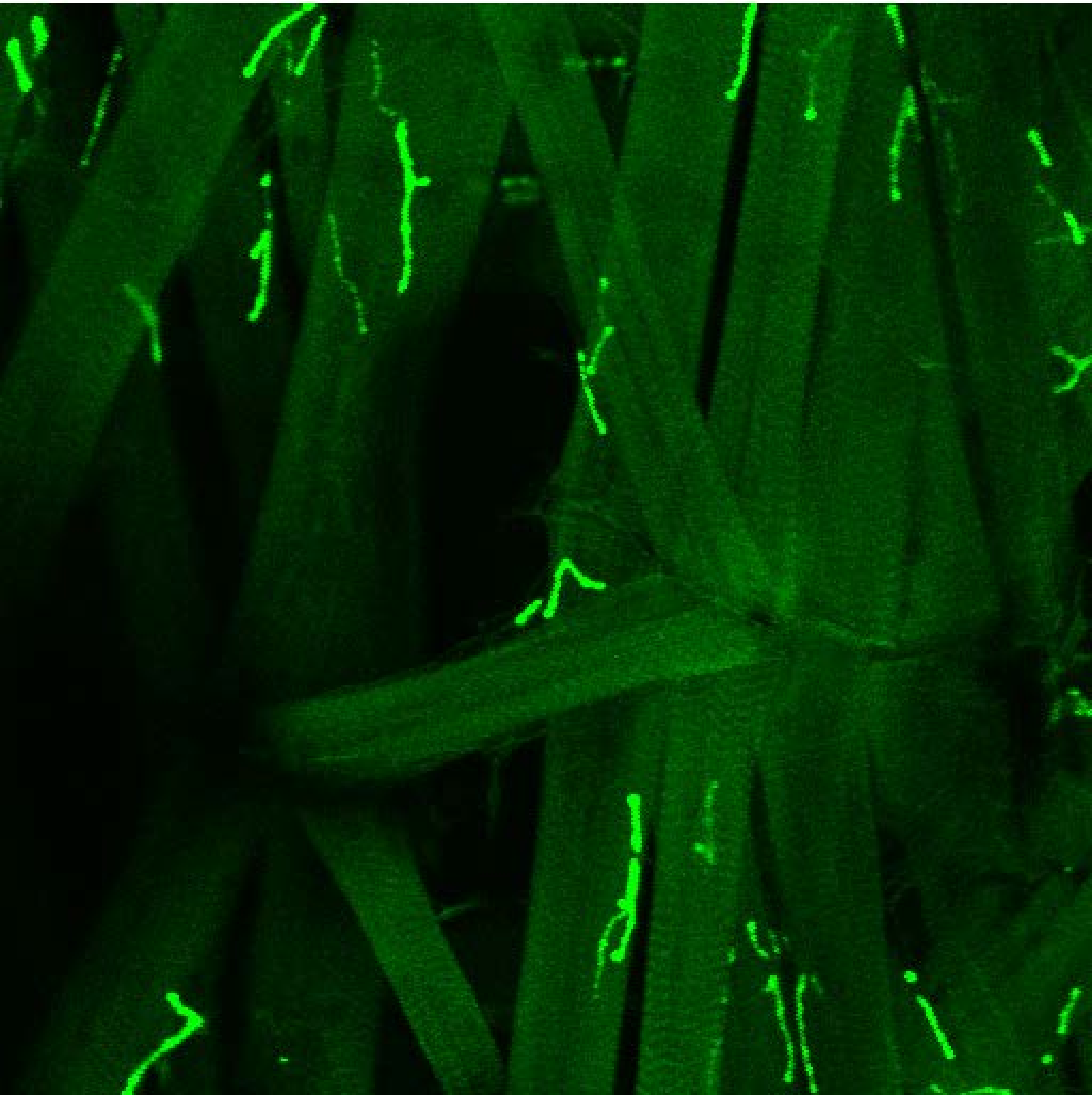

S4B_3.tif

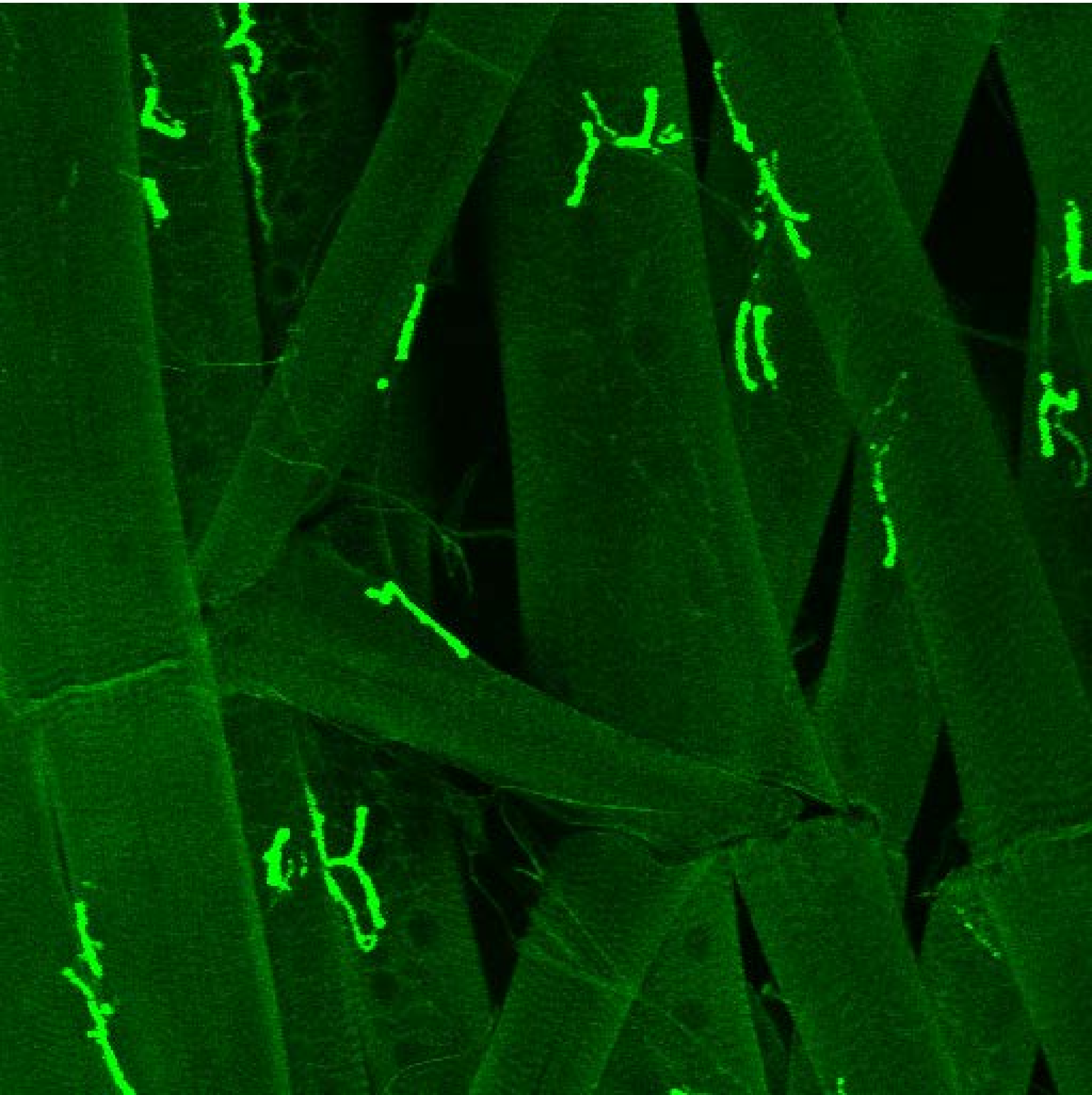

S4B_4.tif

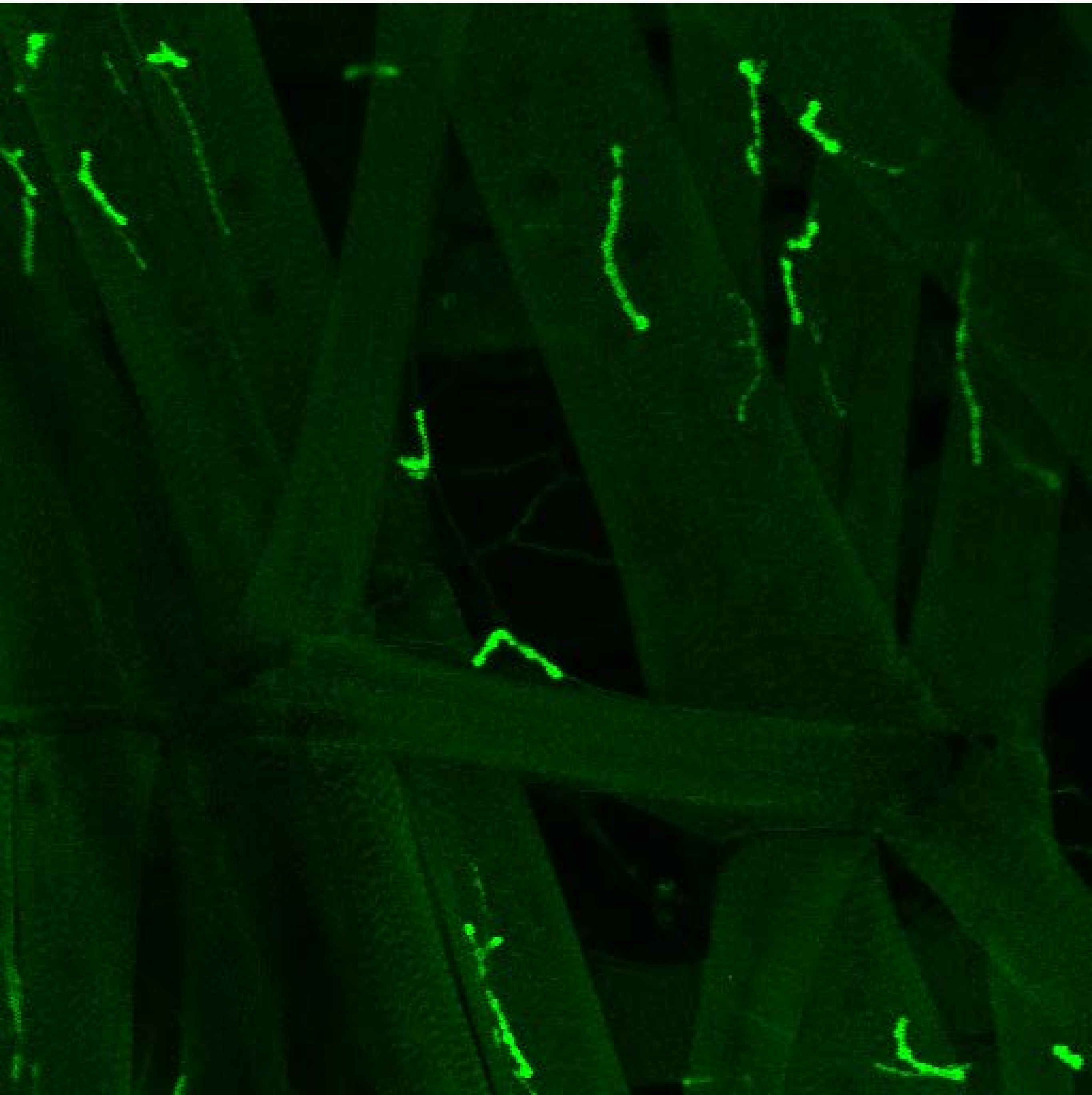

S4D_1.tif

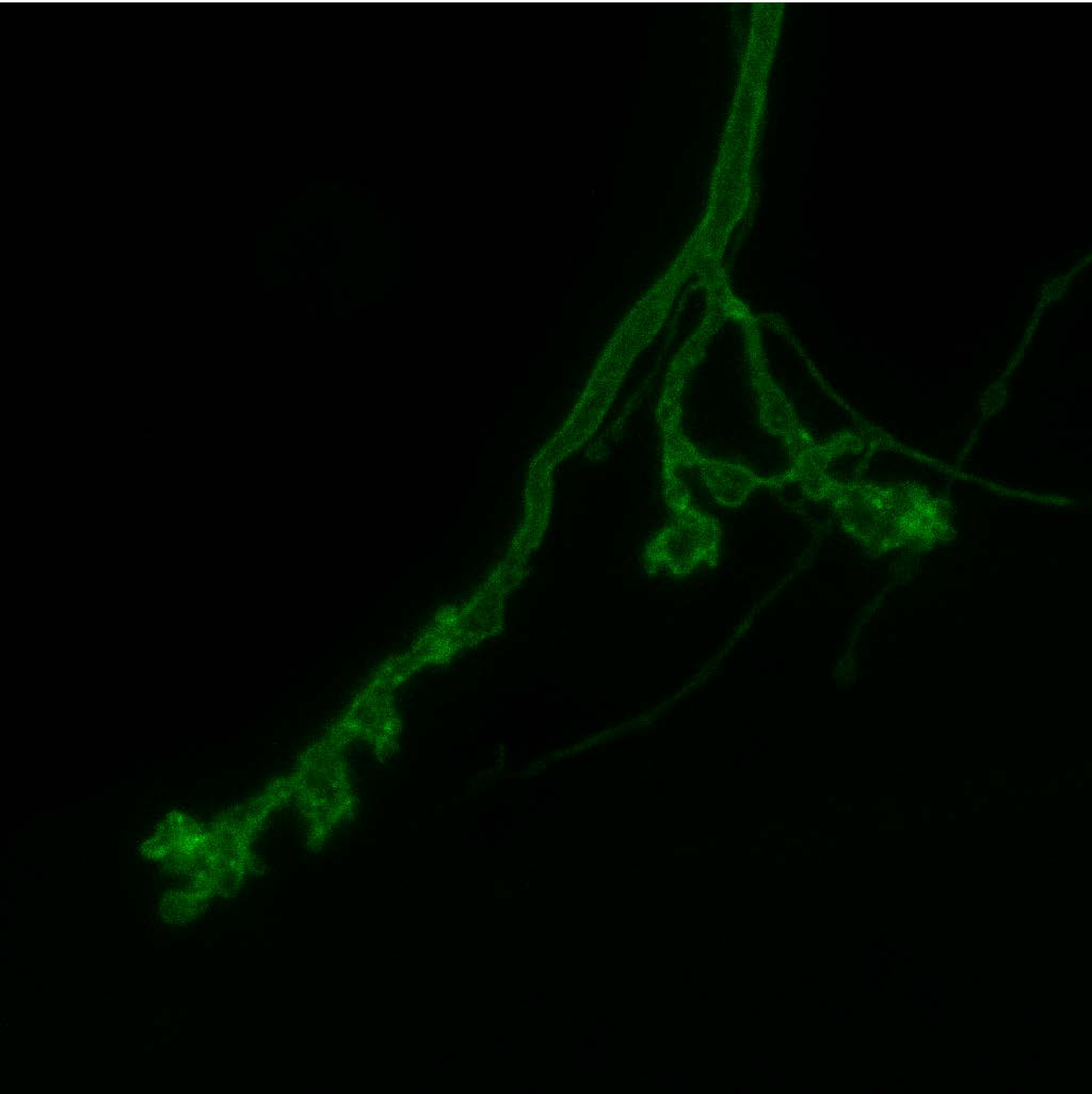

S4D_2.tif

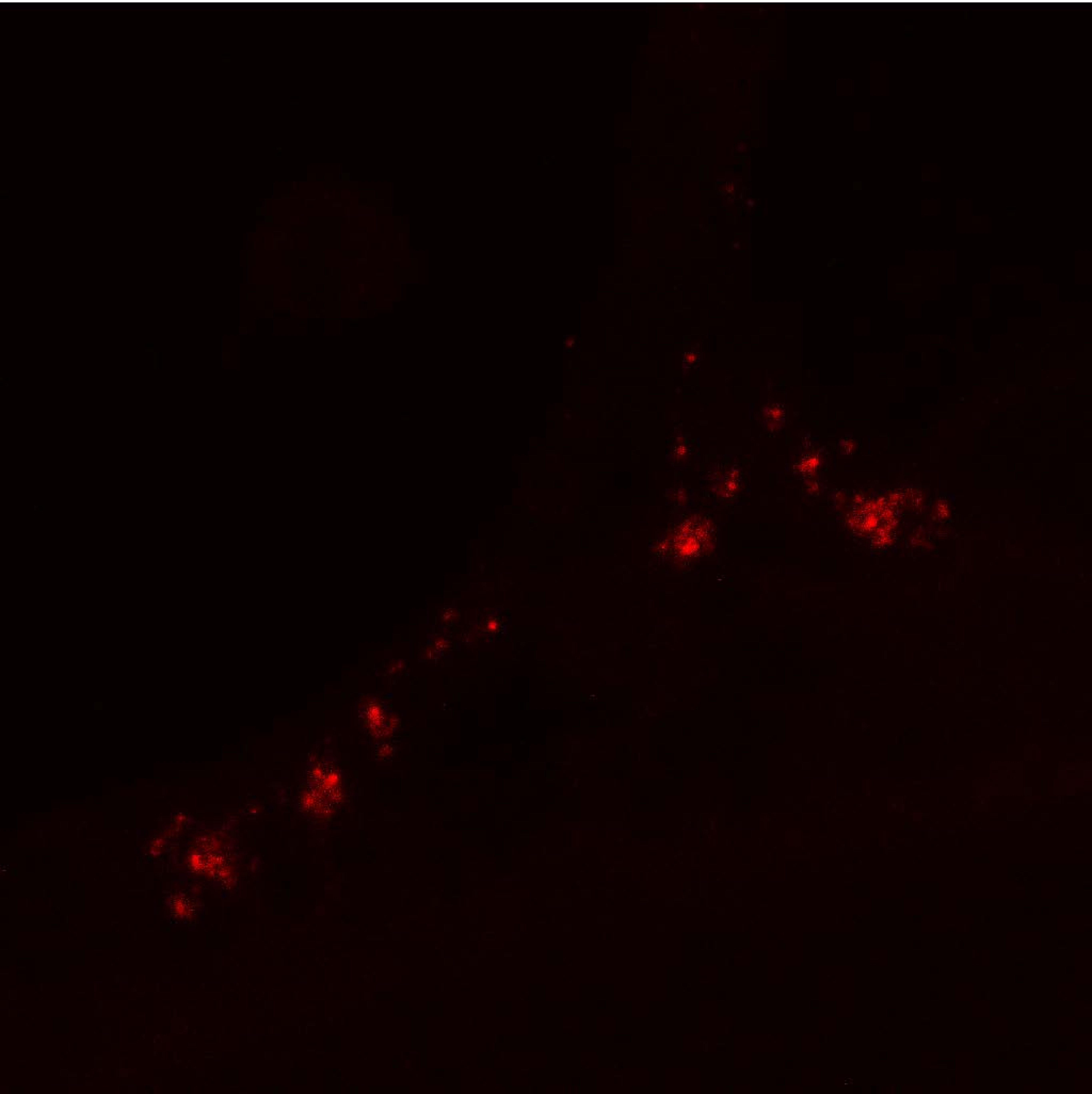

S4D_3.tif

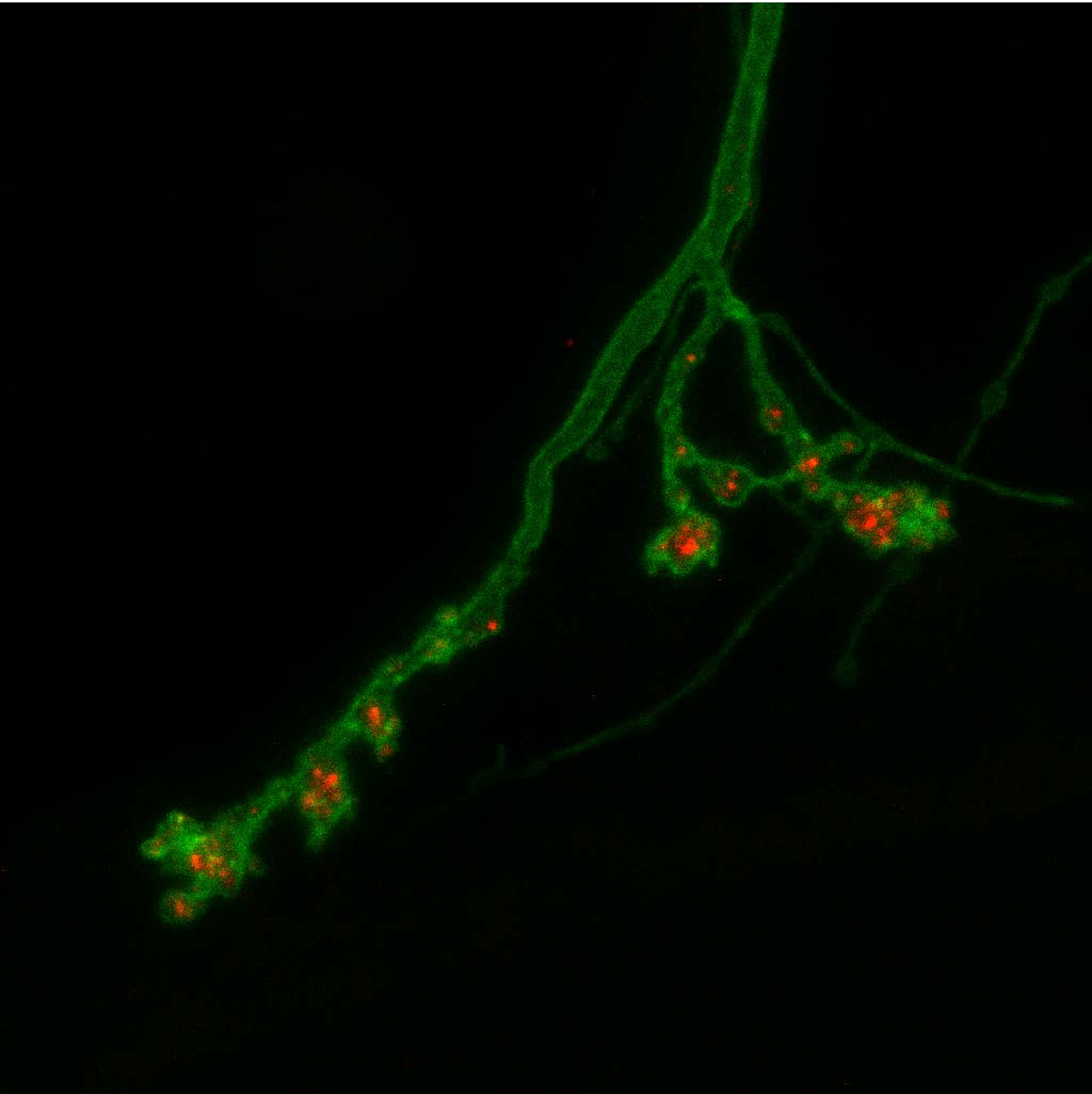

S4D_4.tif

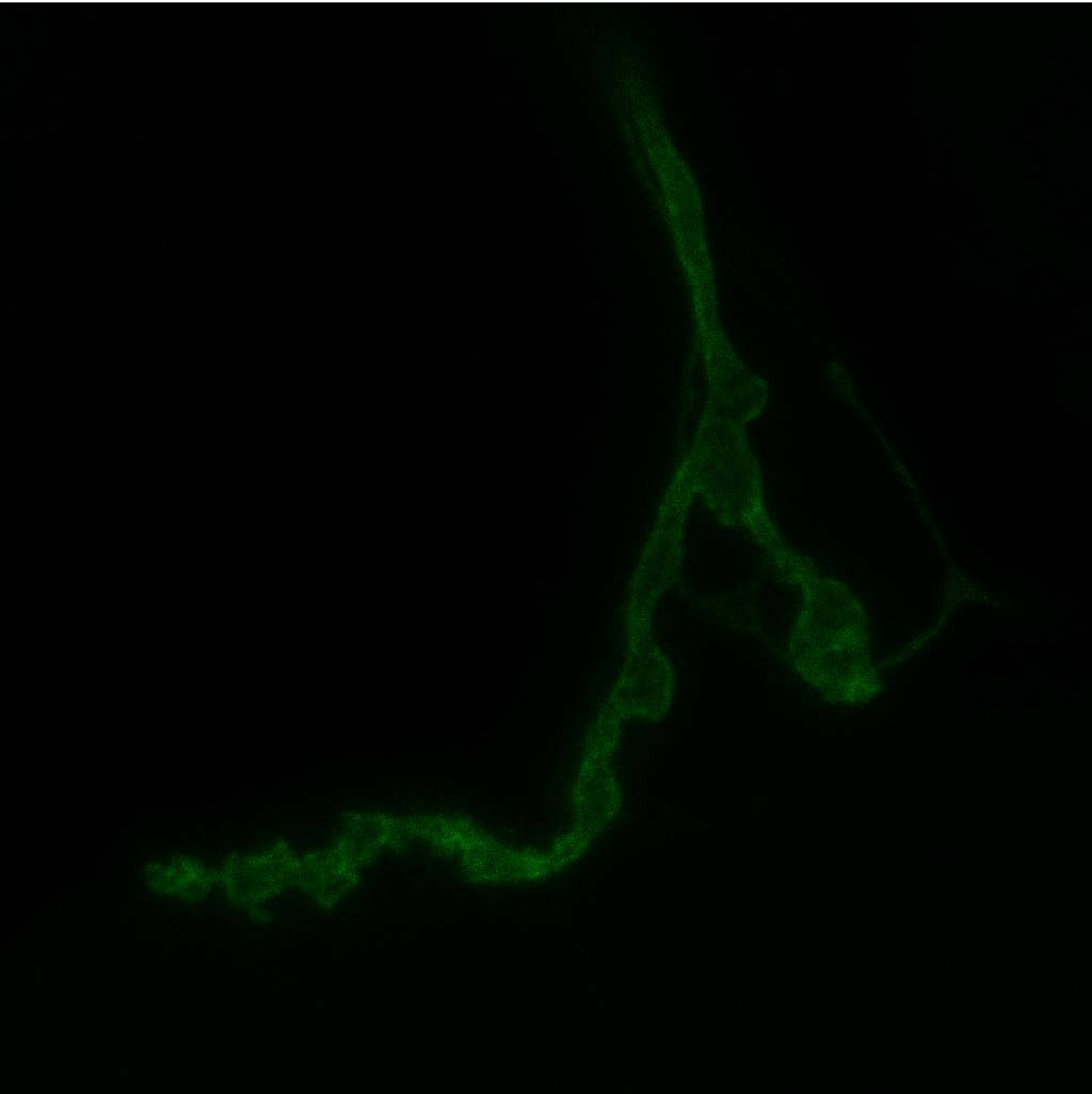

S4D_5.tif

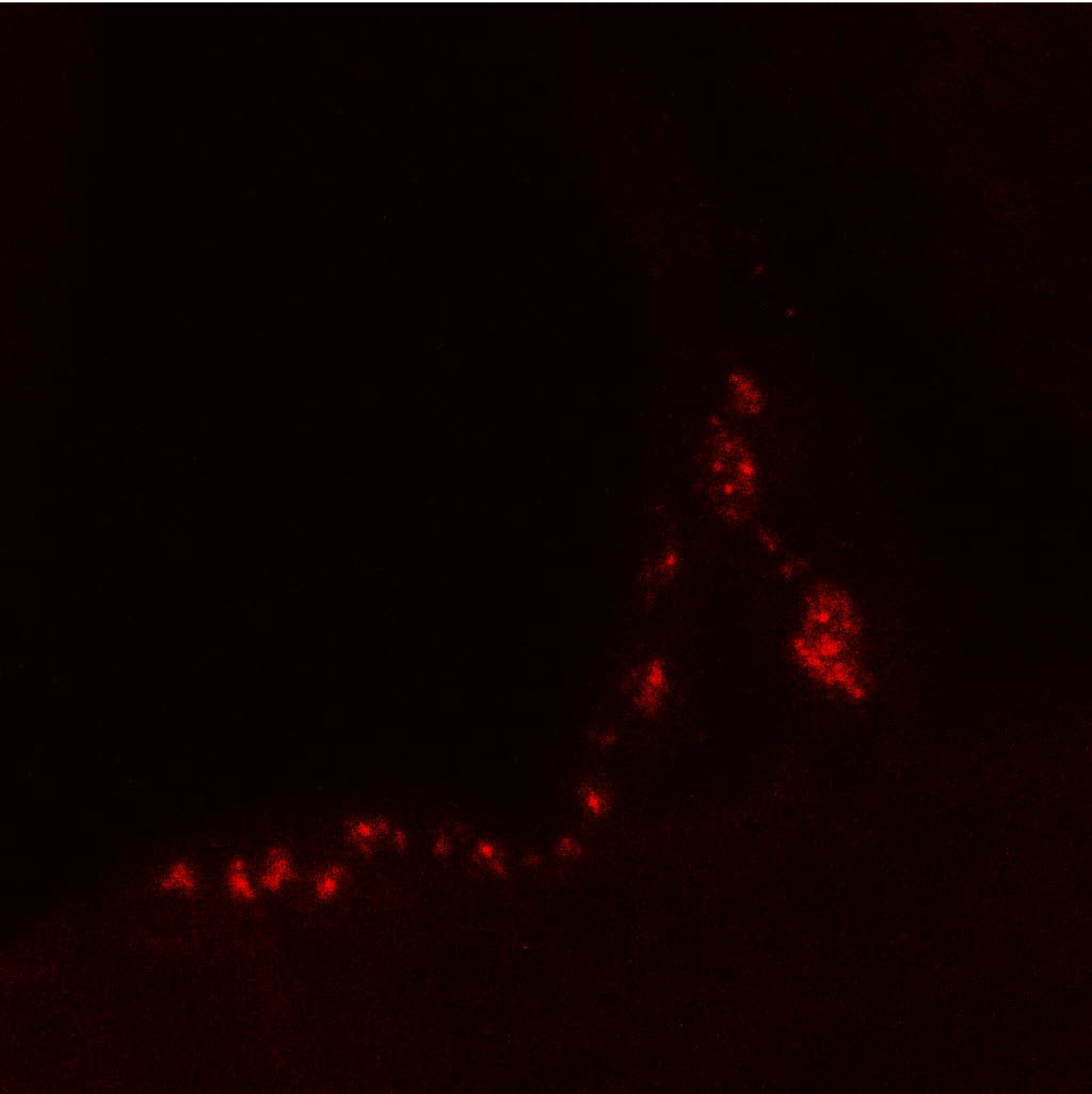

S4D_6.tif

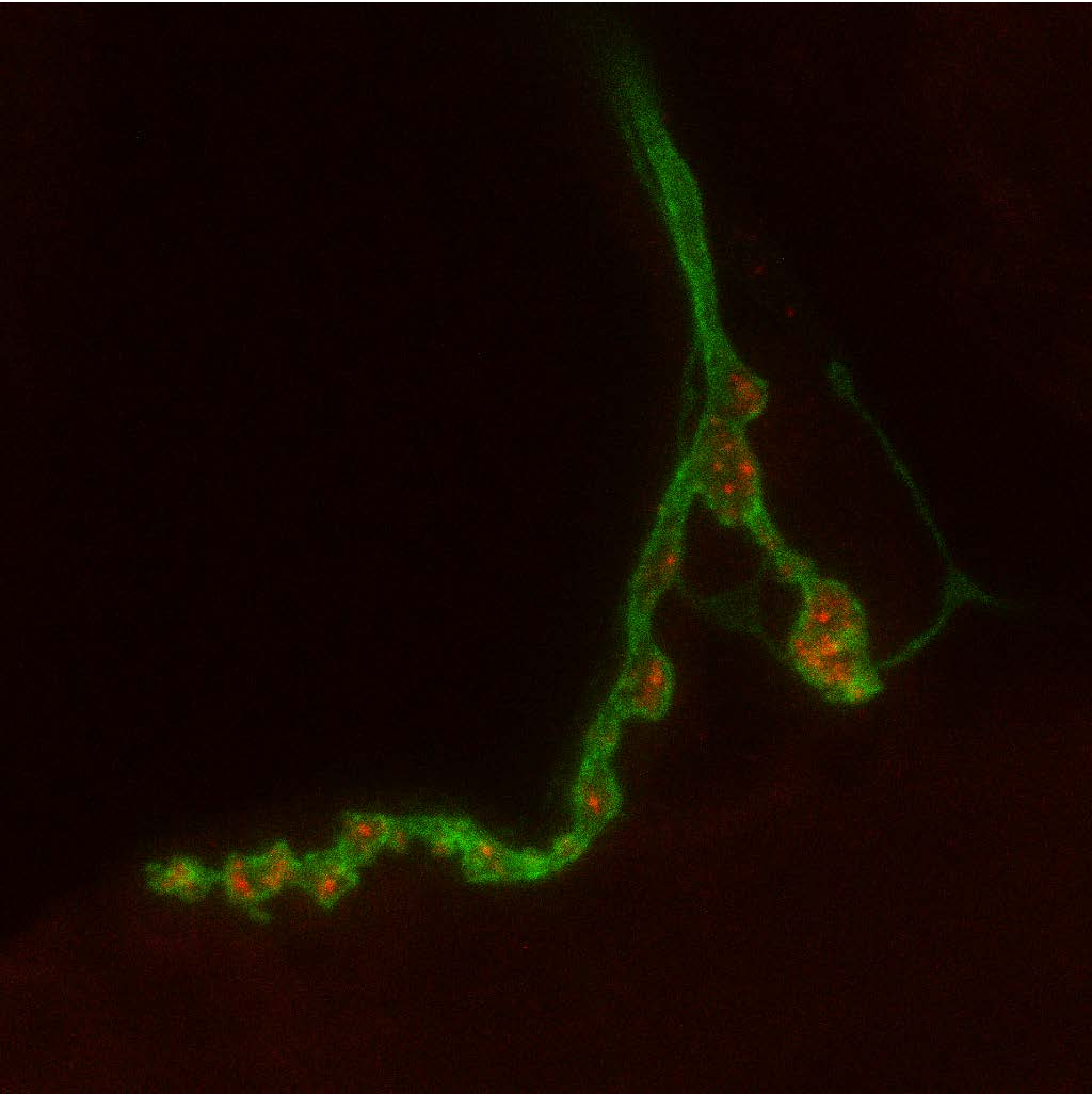

S4E_1.tif

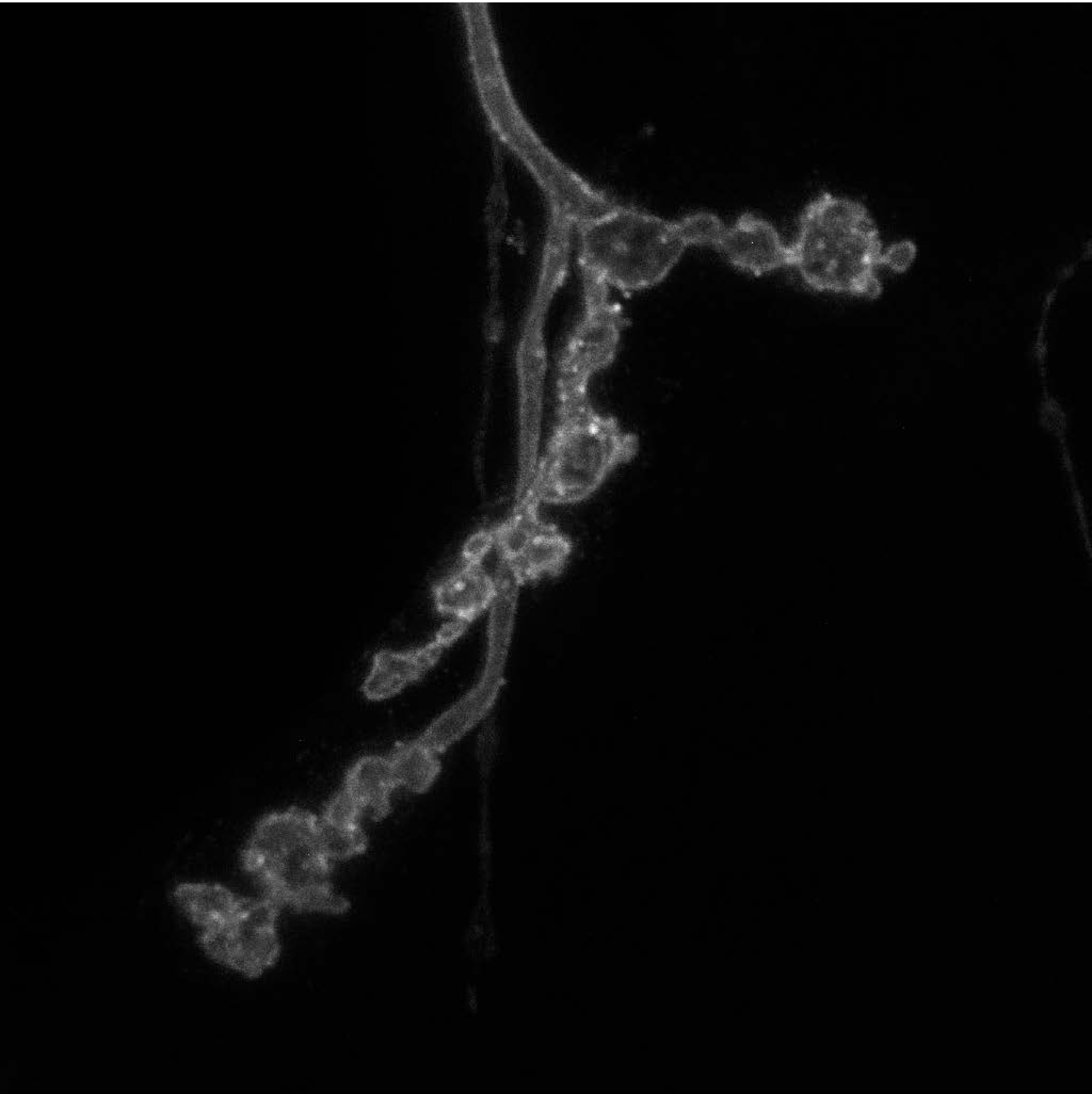

S4E_2.tif

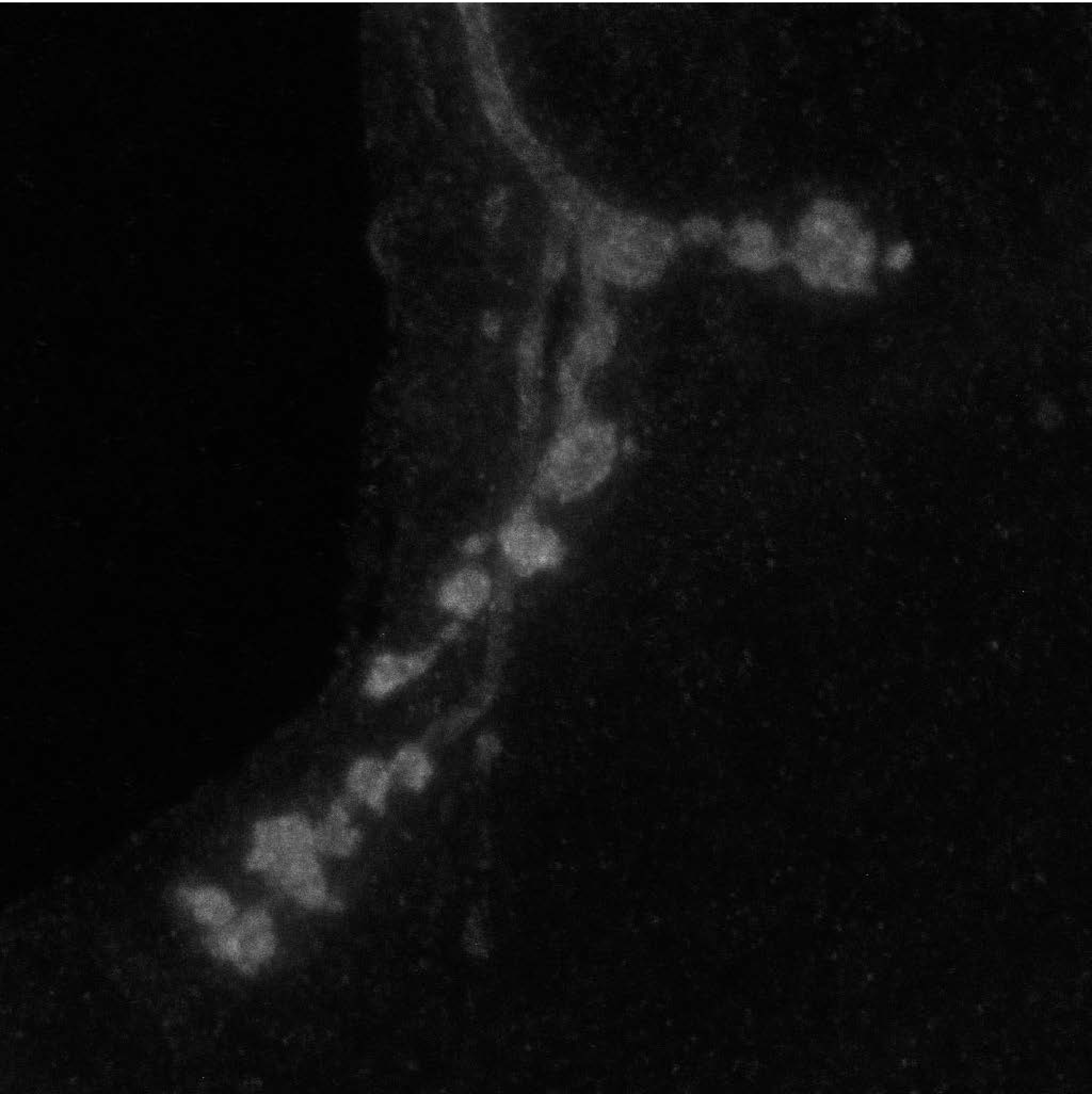

S4E_4.tif

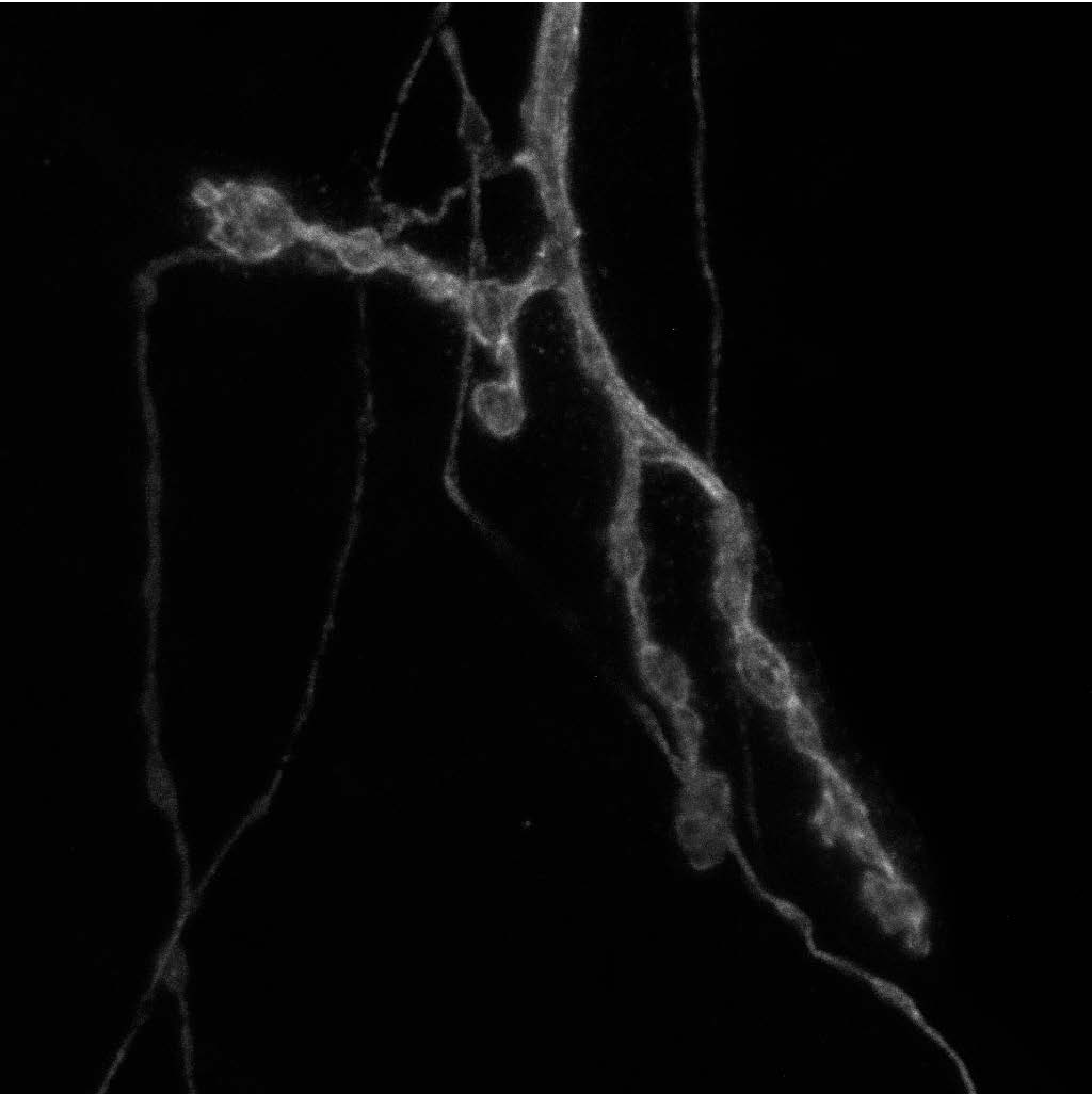

S4E_5.tif

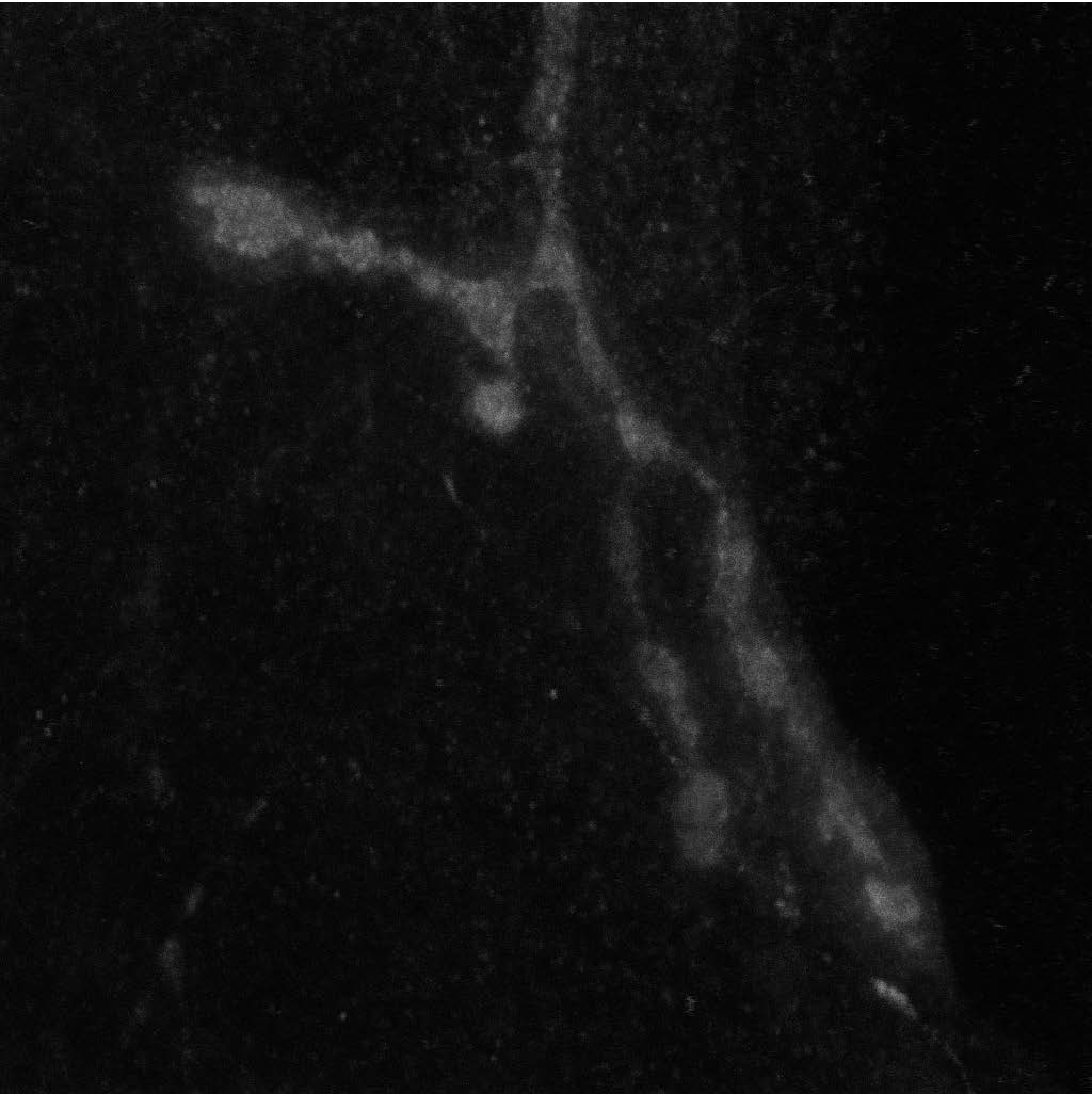

S4F_1.tif

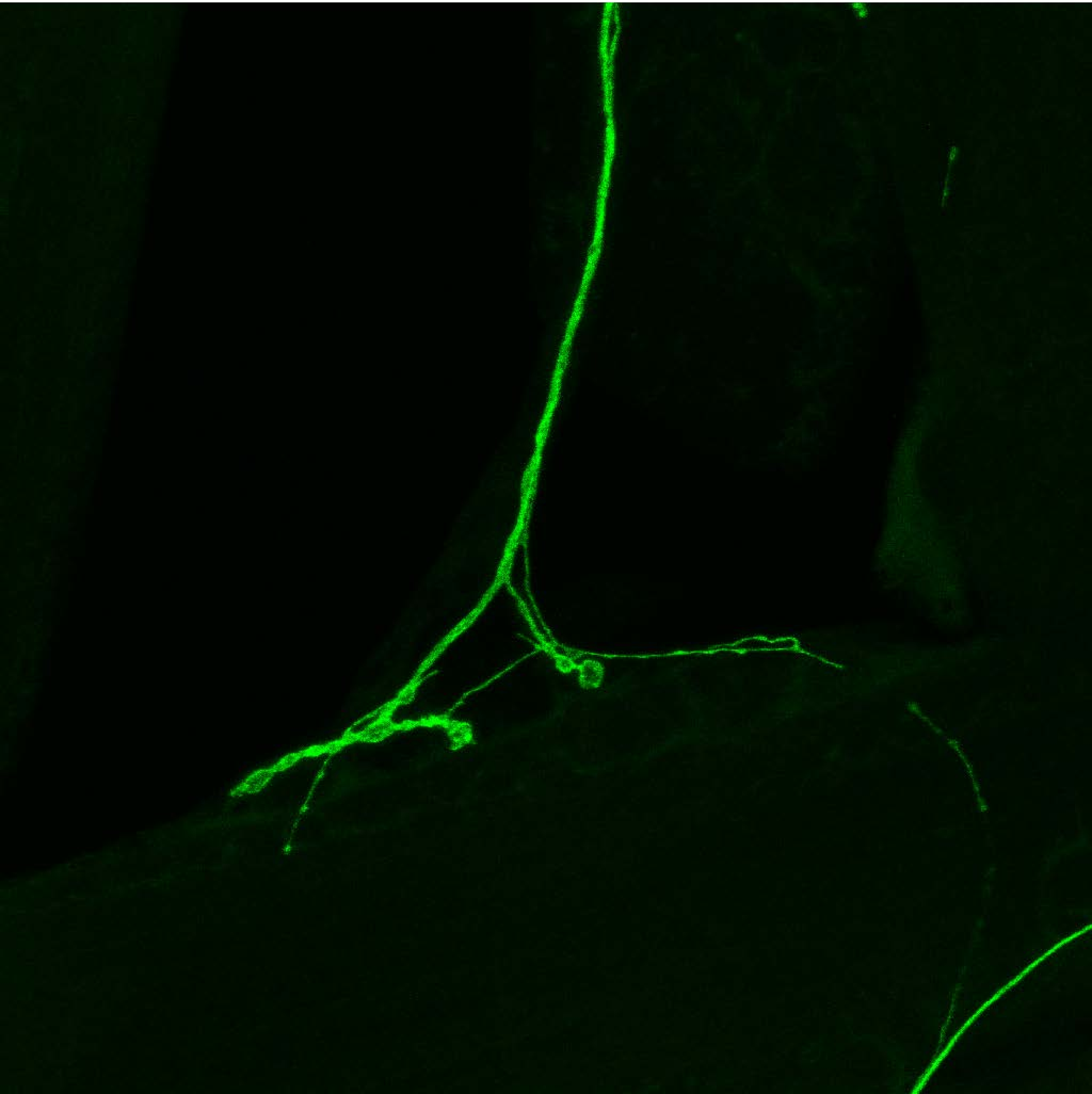

S4F_2.tif

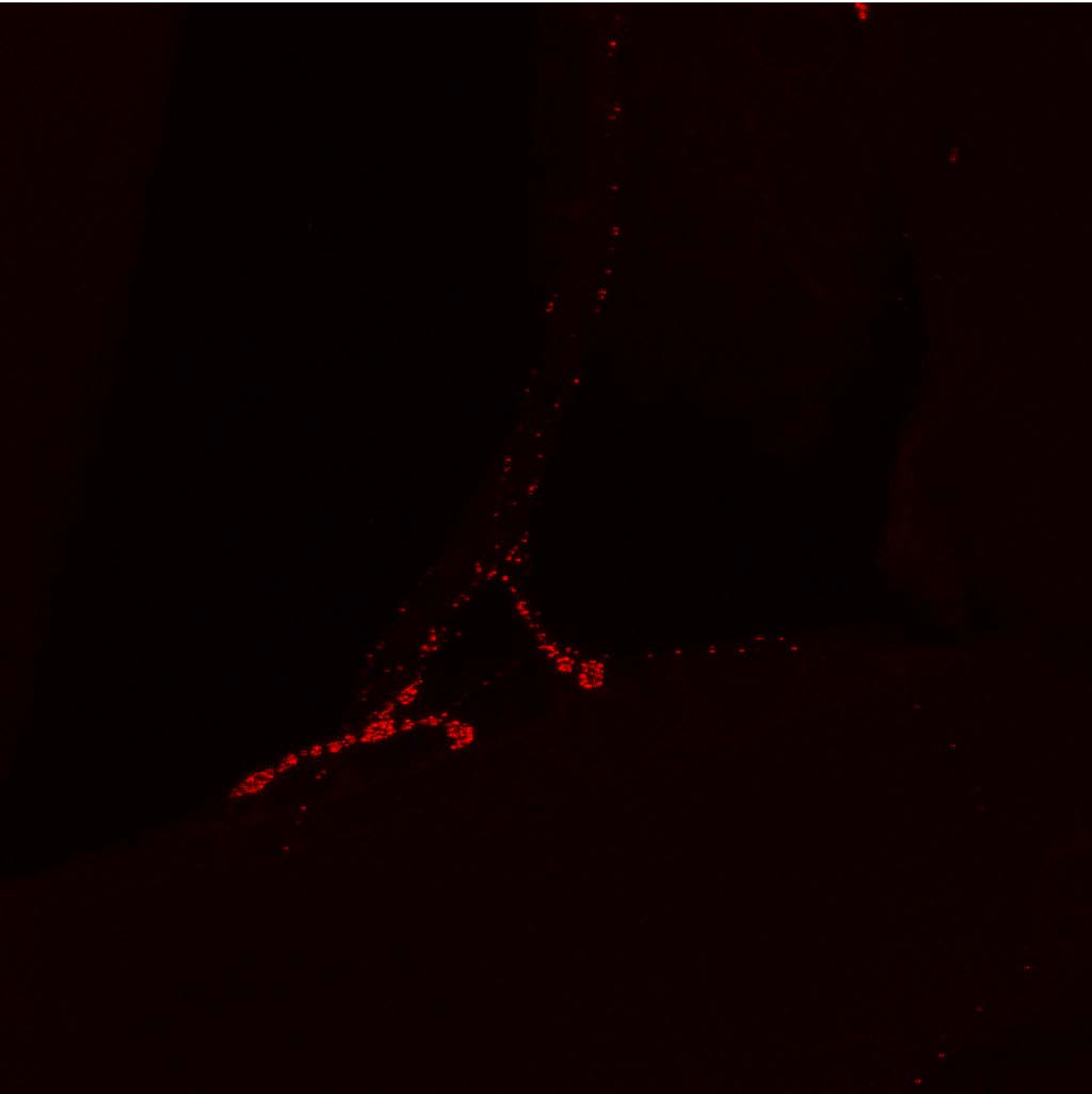

S4F_3.tif

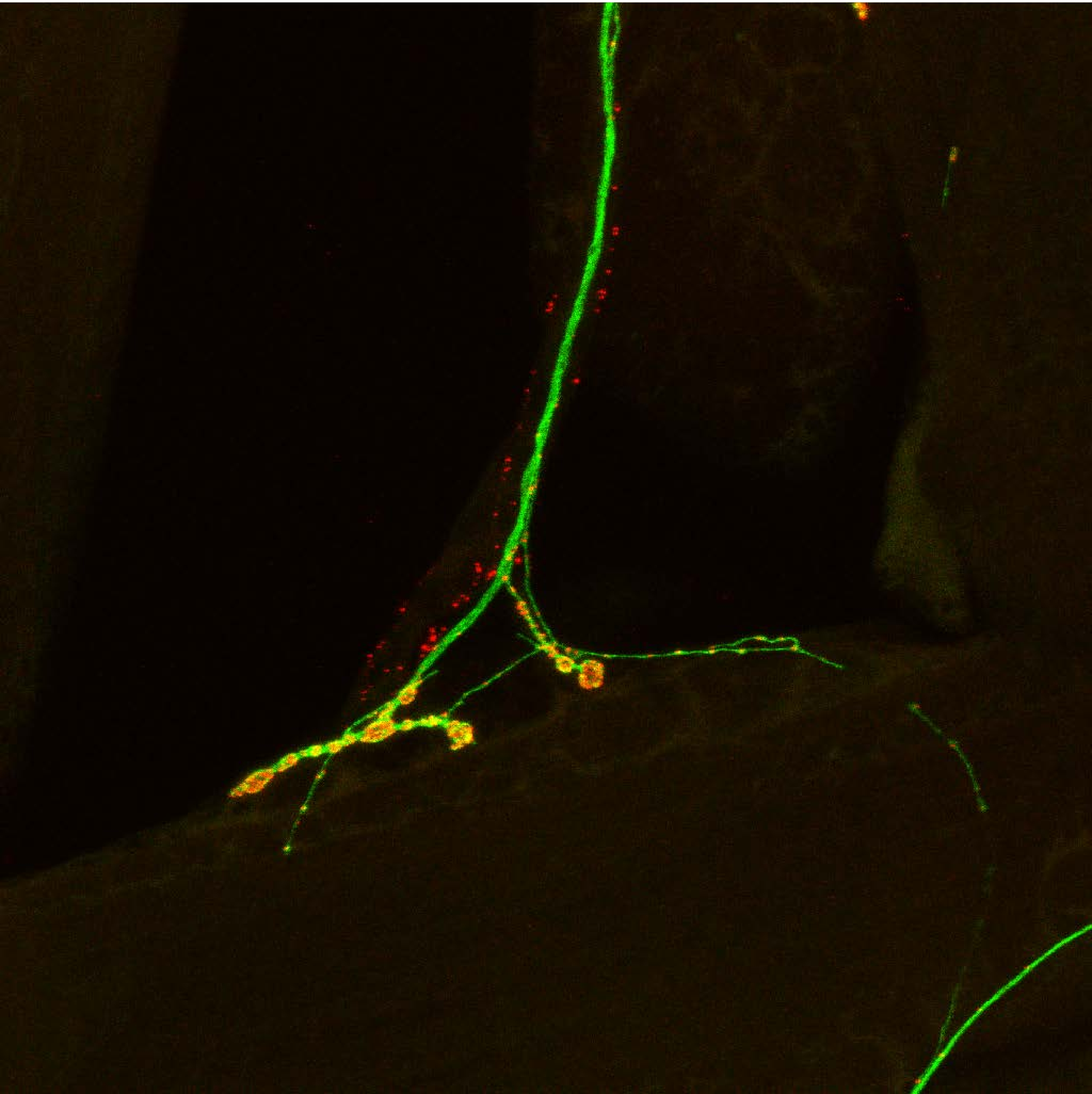

S4F_4.tif

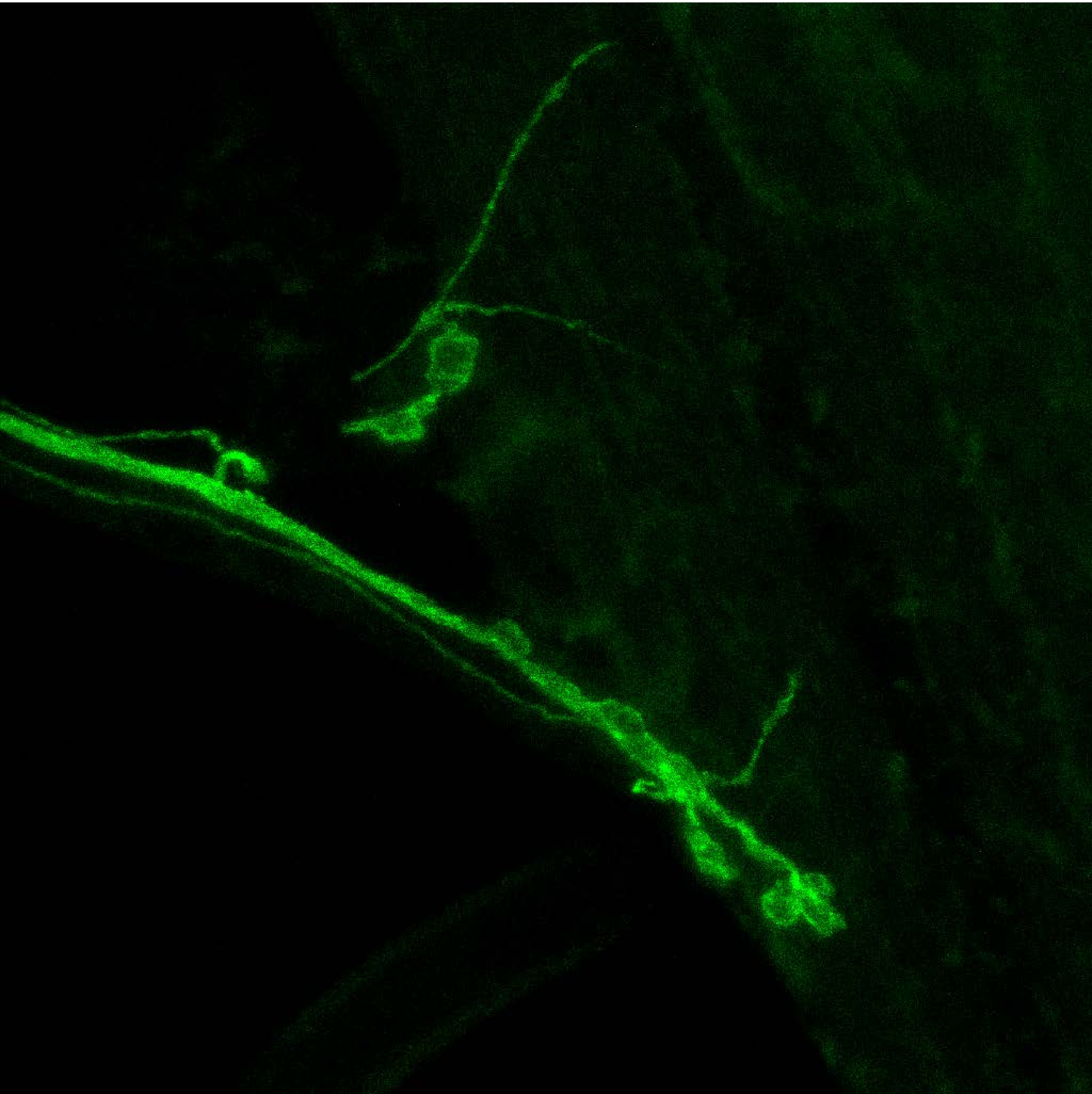

S4F_5.tif

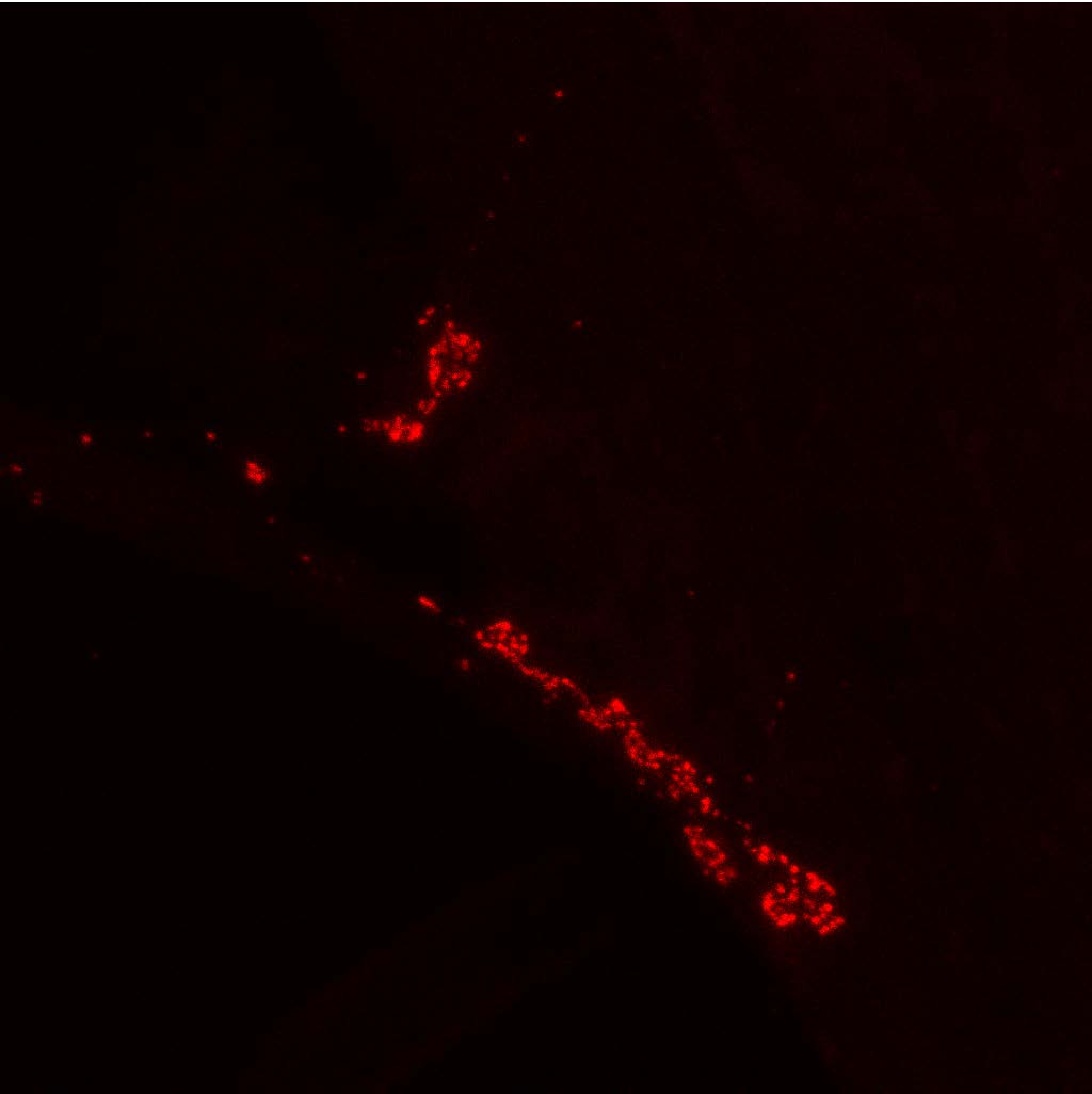

S4F_6.tif

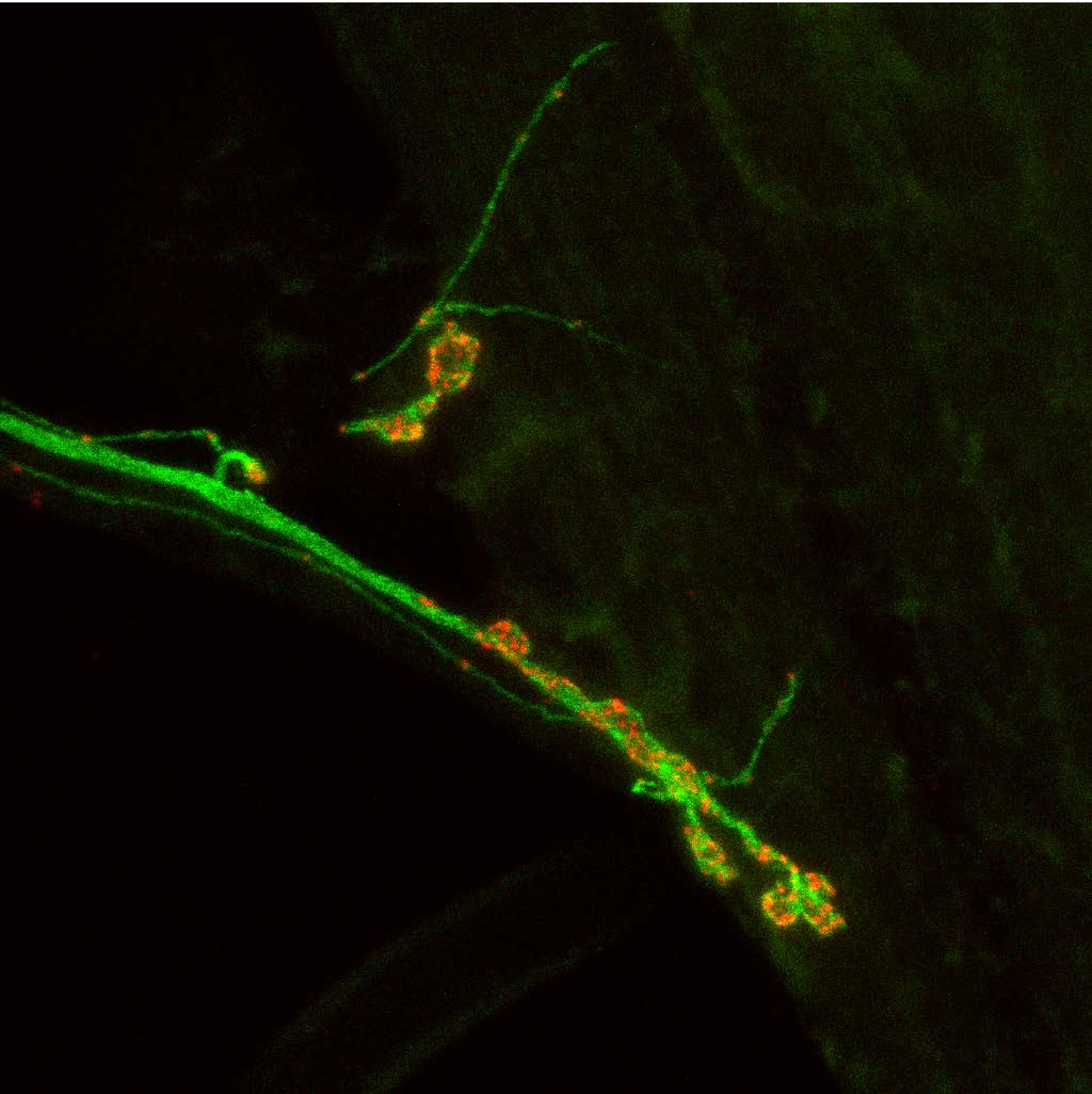

S4F_7.tif

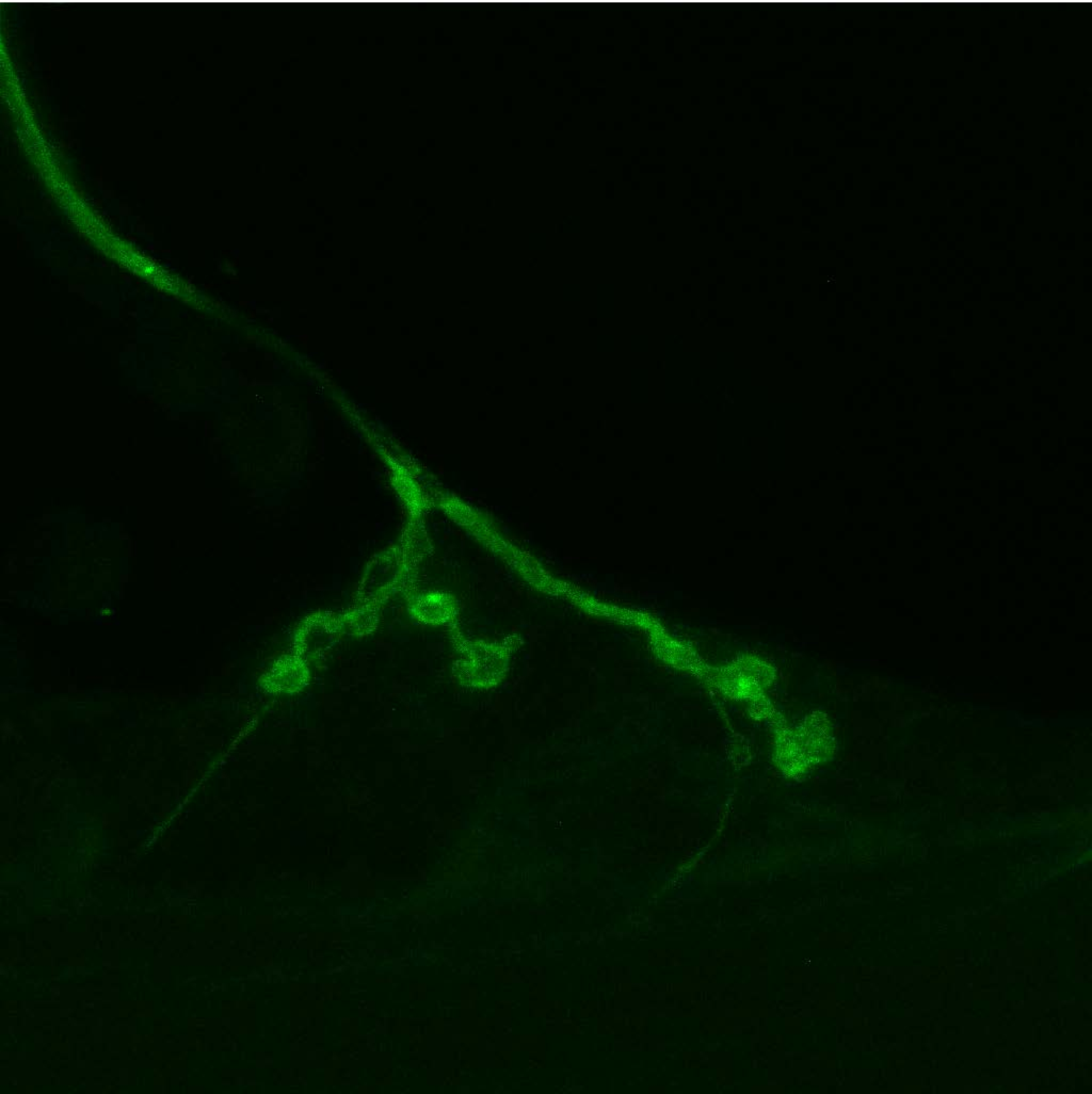

S4F_8.tif

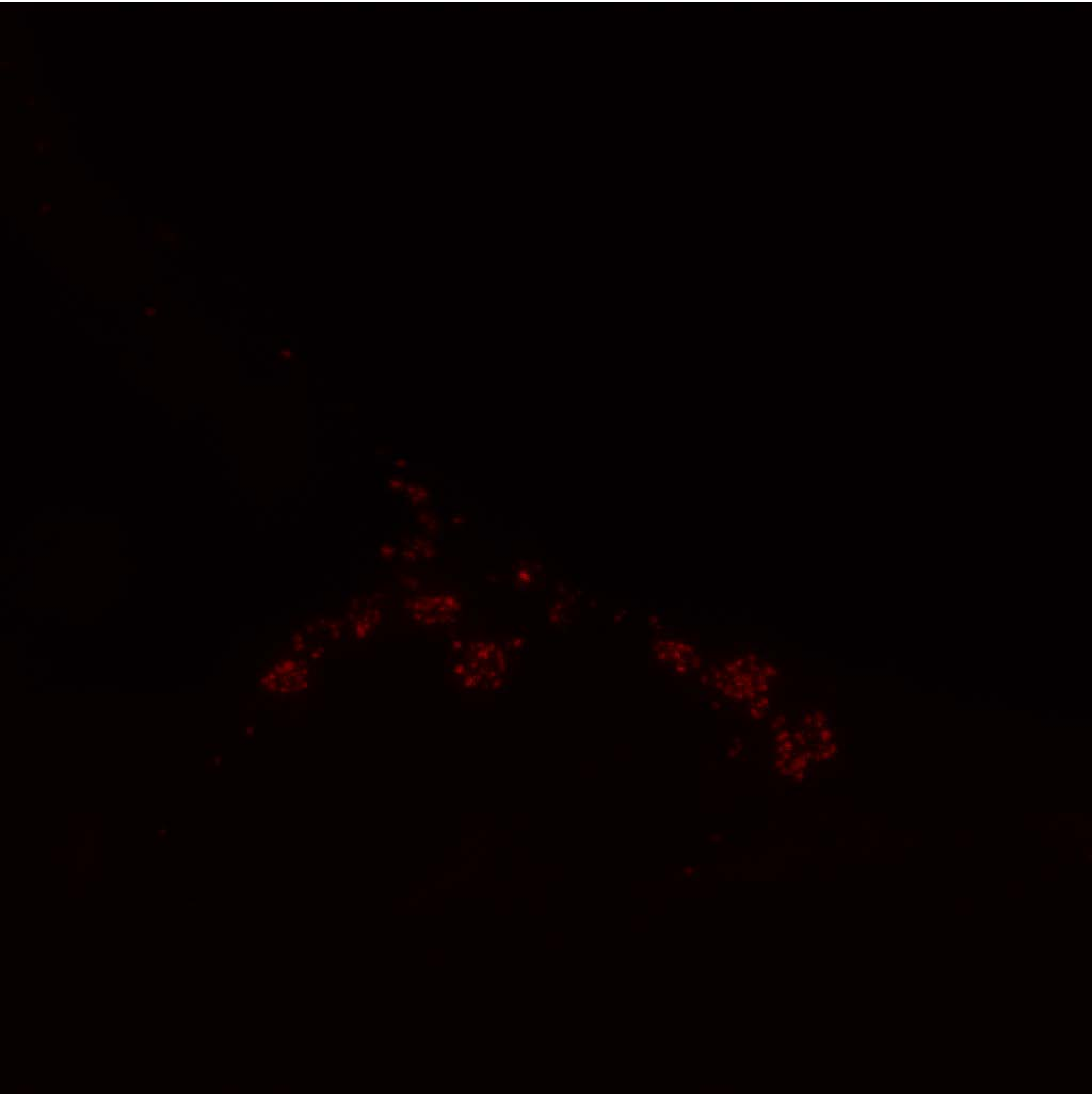

S4F_9.tif

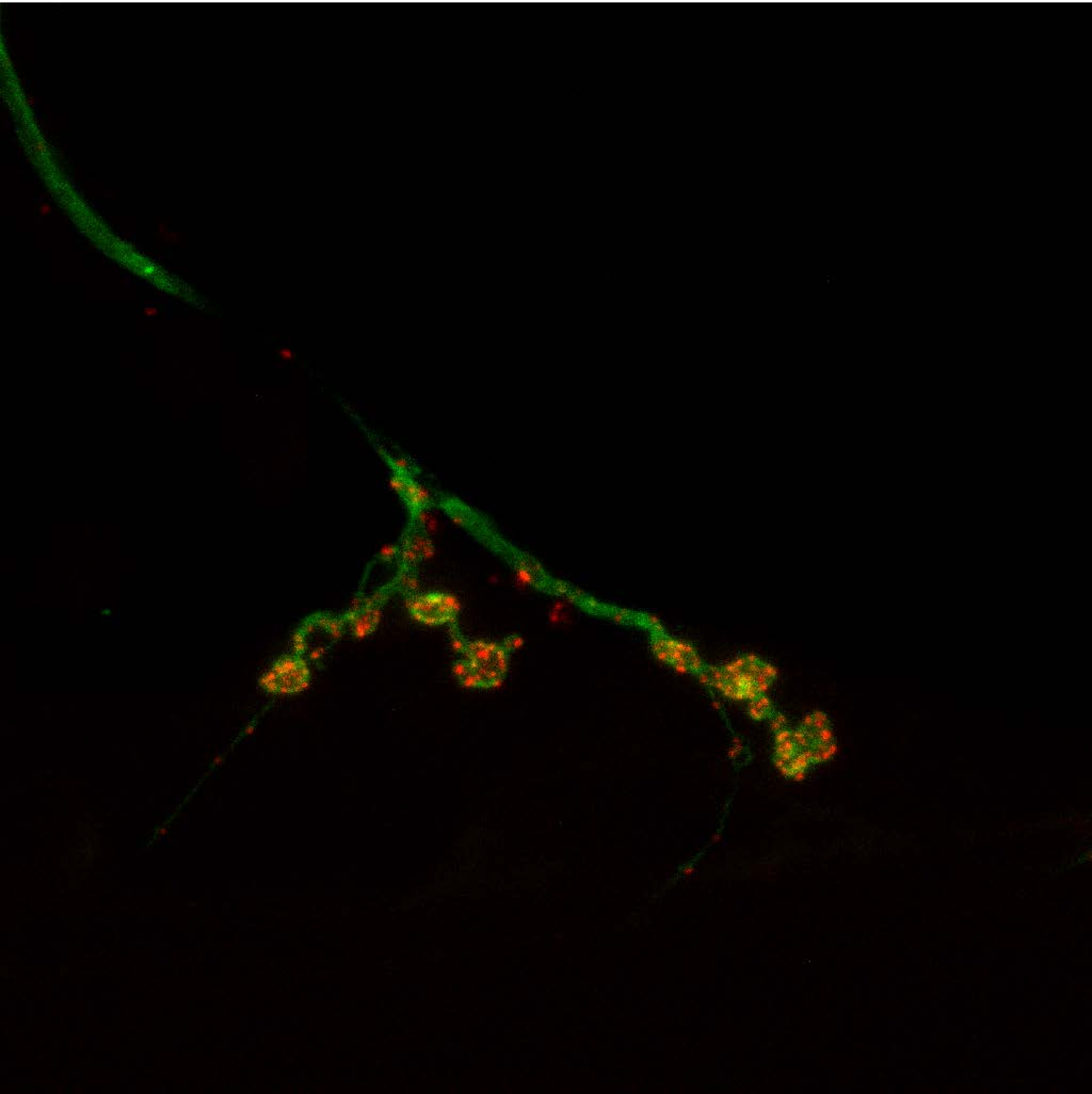

S4F_10.tif

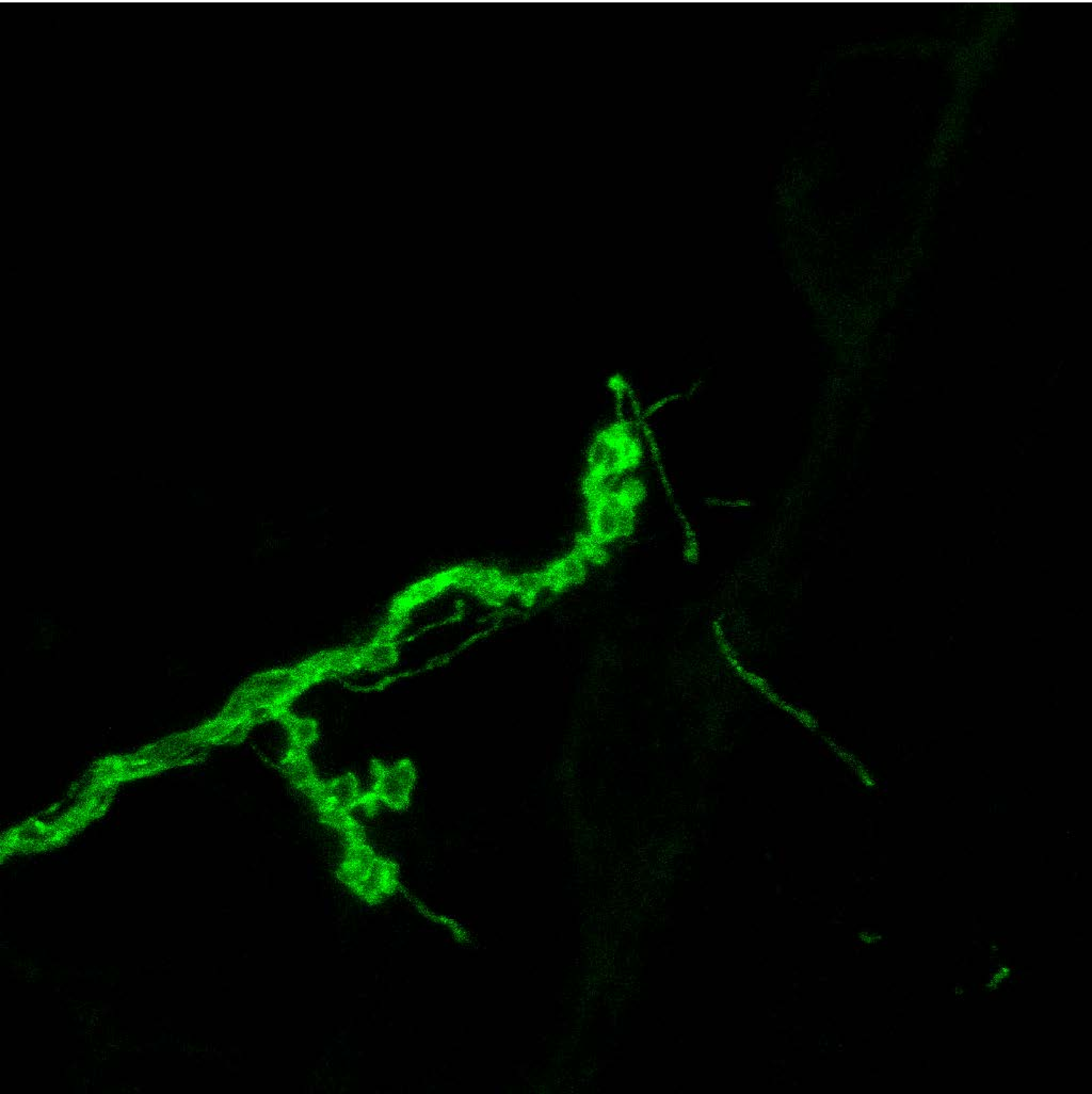

S4F_11.tif

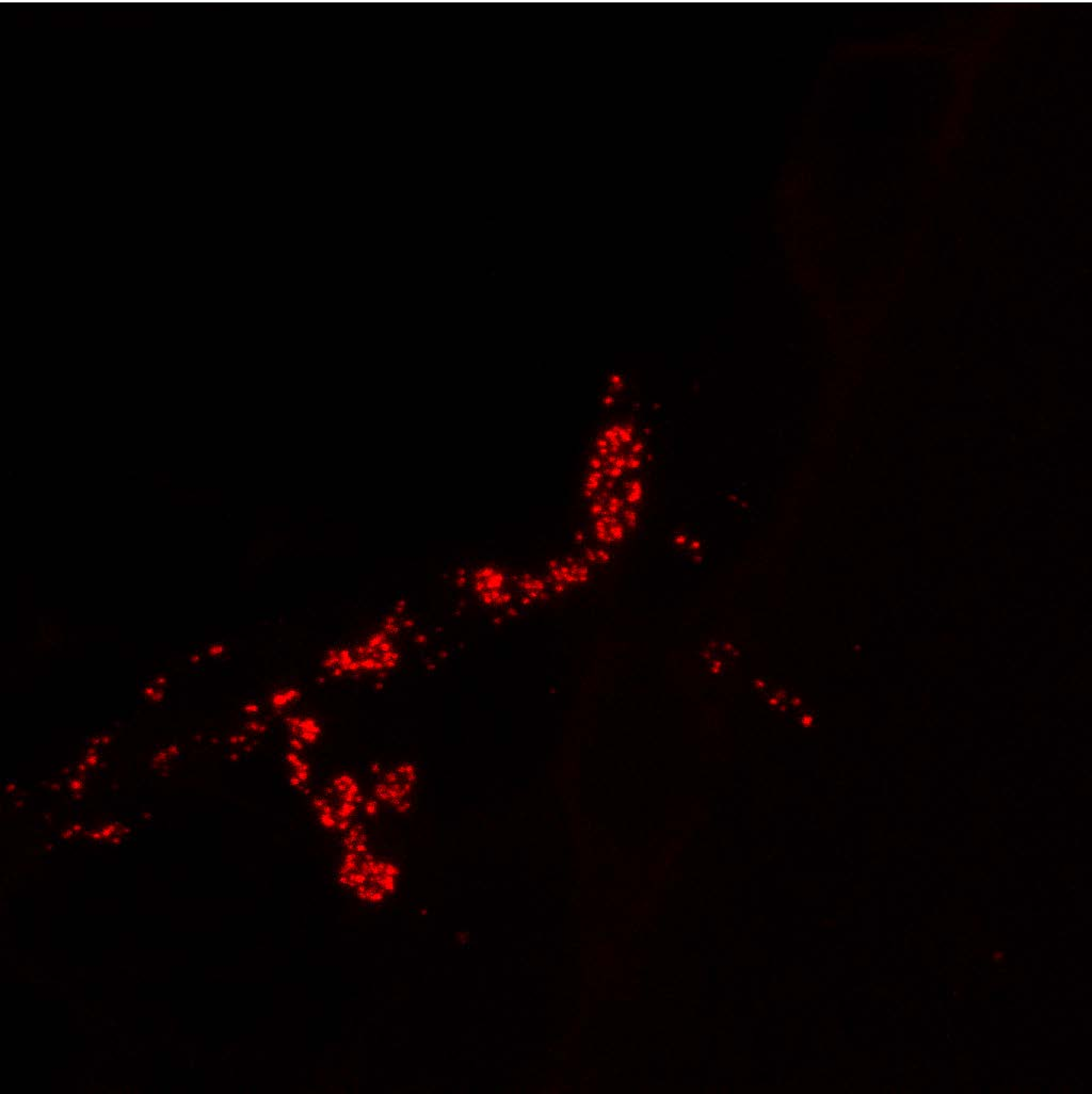

S4F_12.tif

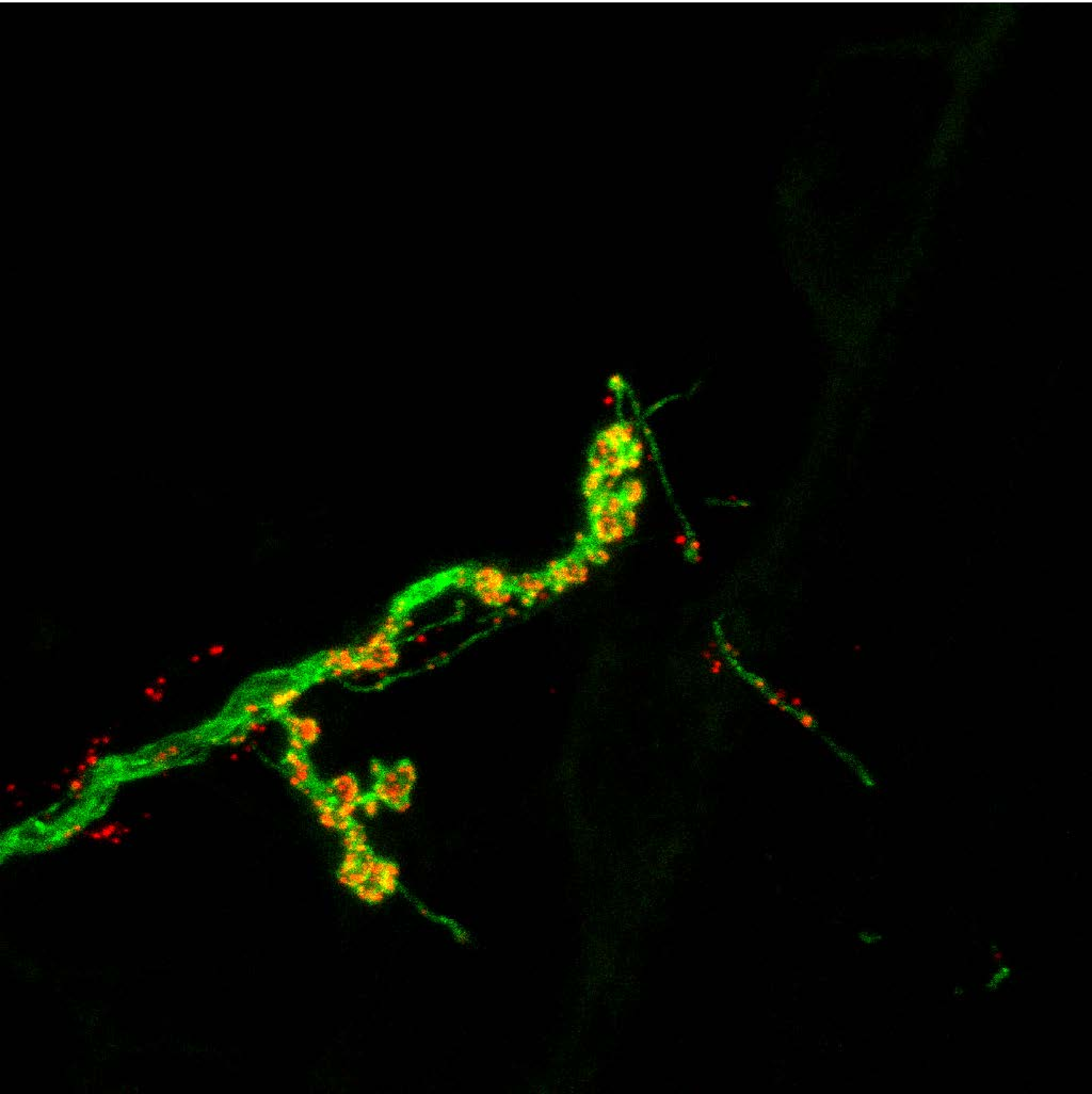

S5F_1.tif

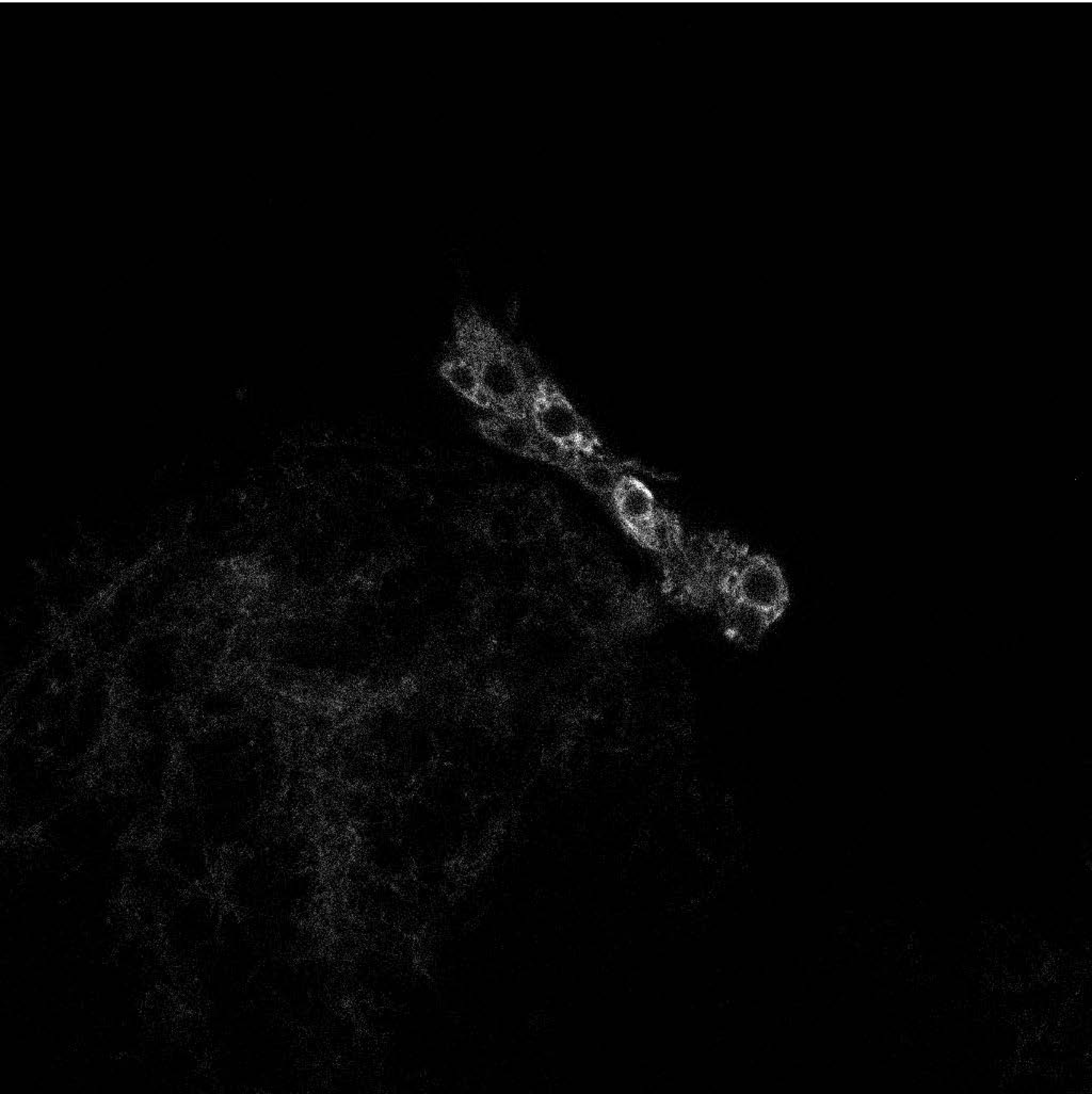

S5F_2.tif

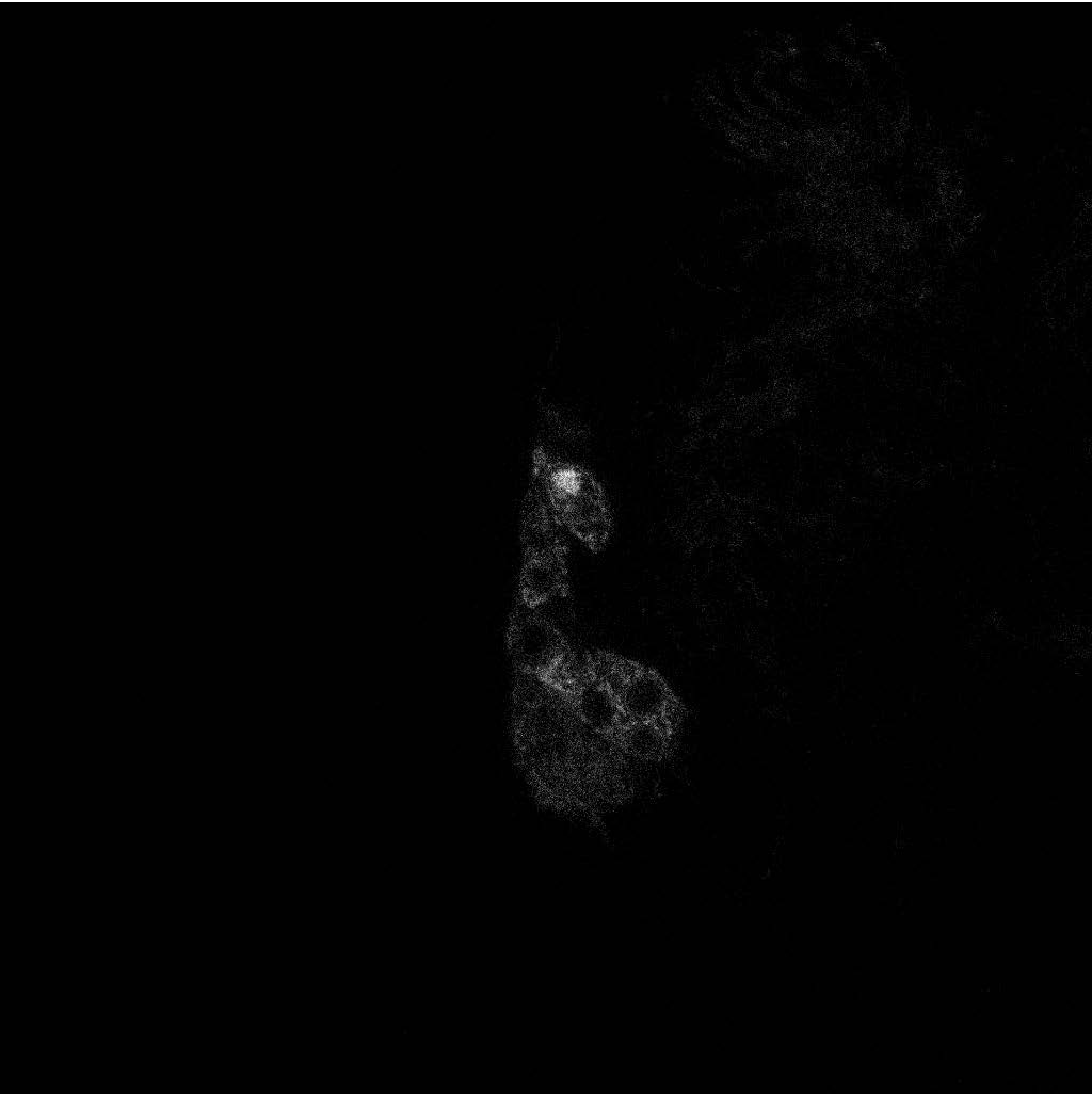

S5F_3.tif

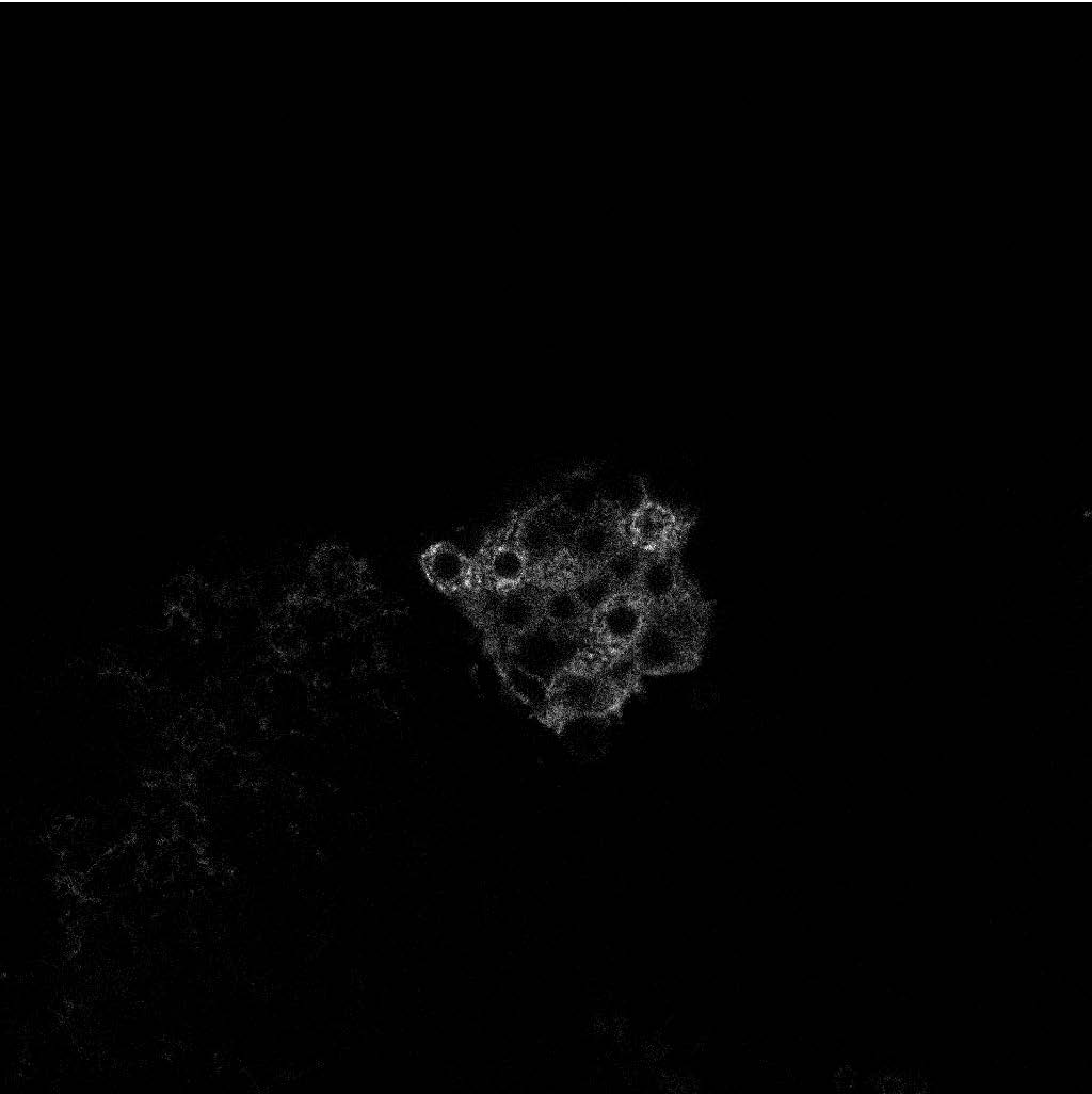

S5F_4.tif

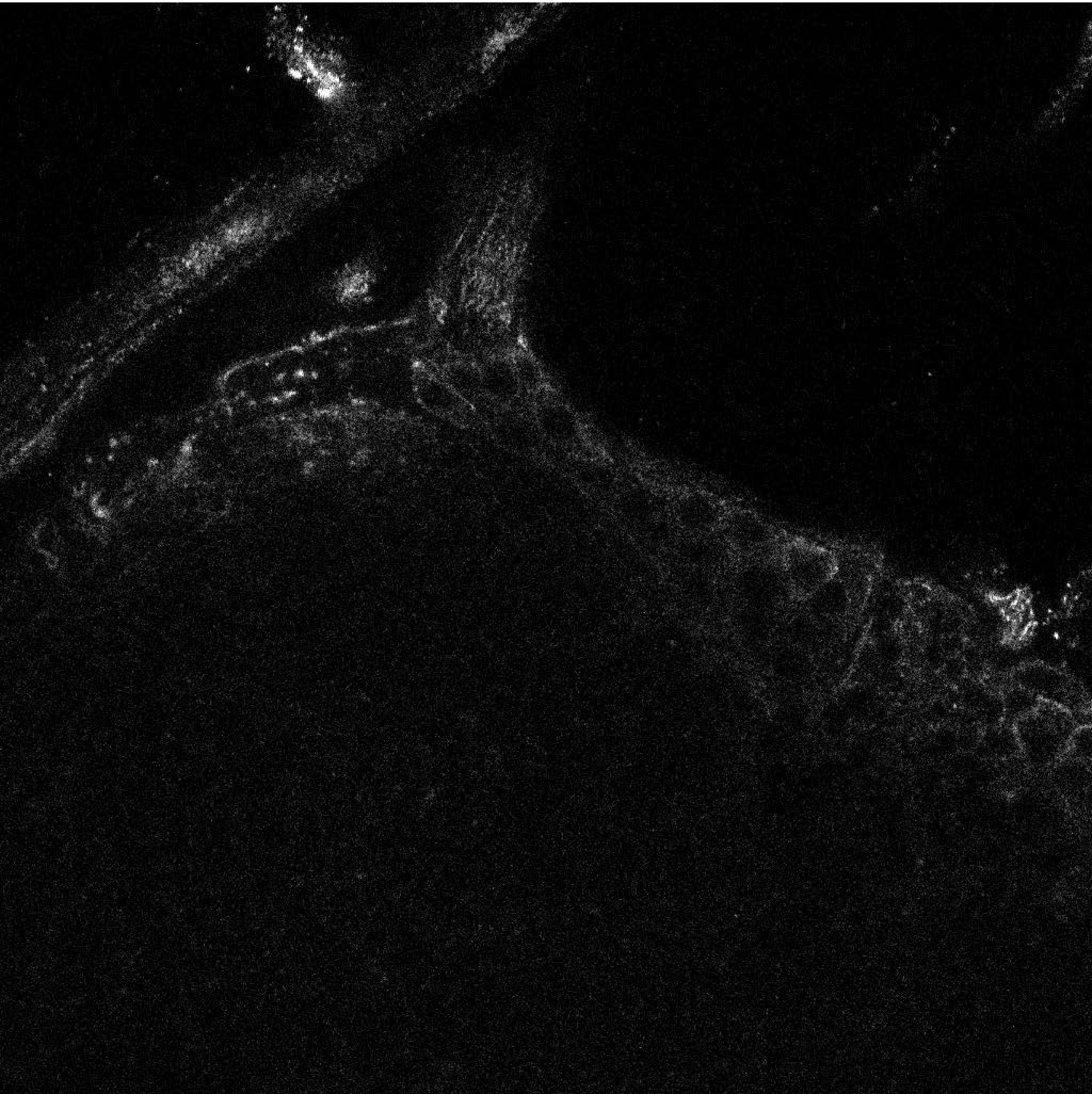

S5F_5.tif

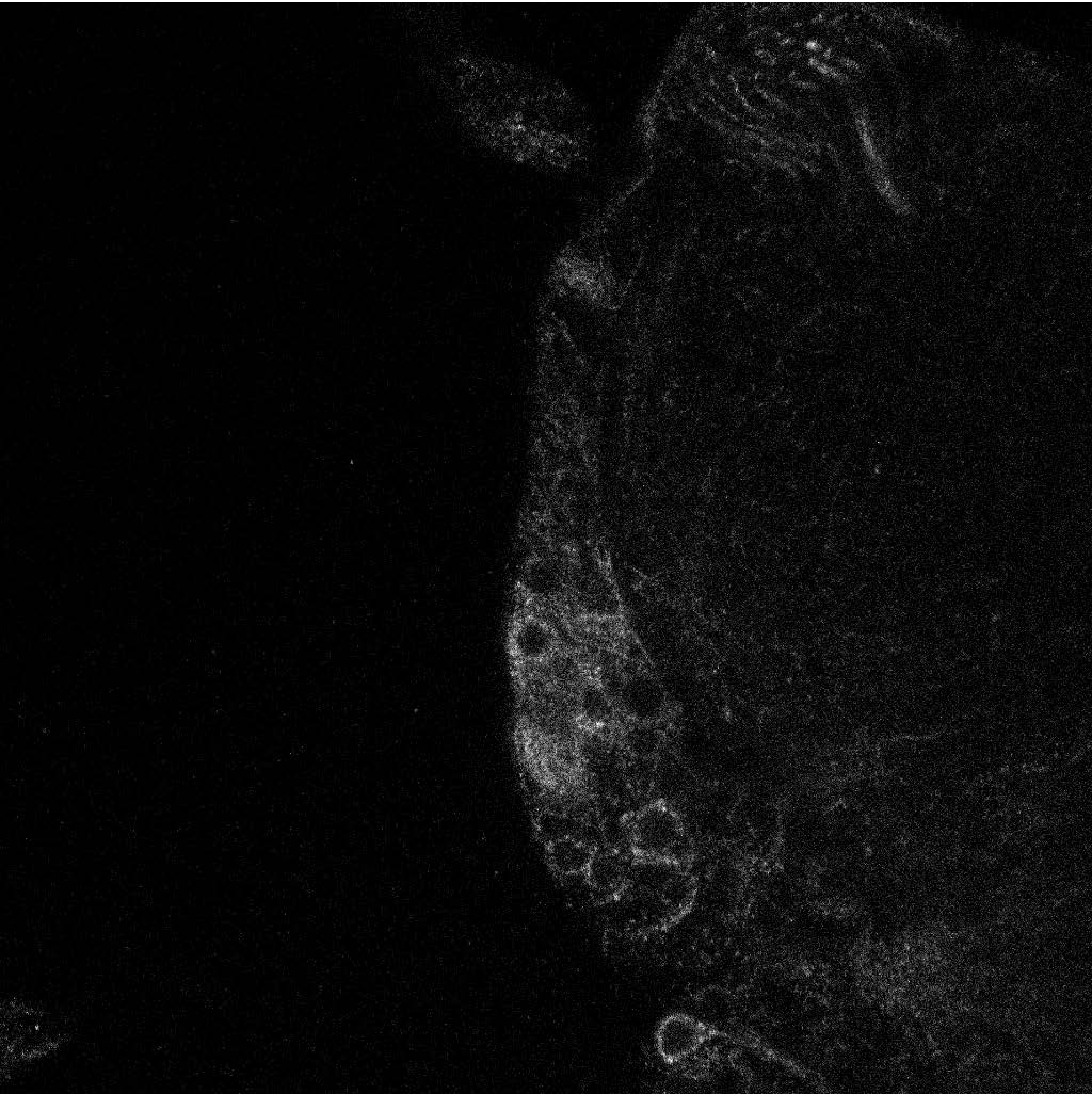

S5F_6.tif

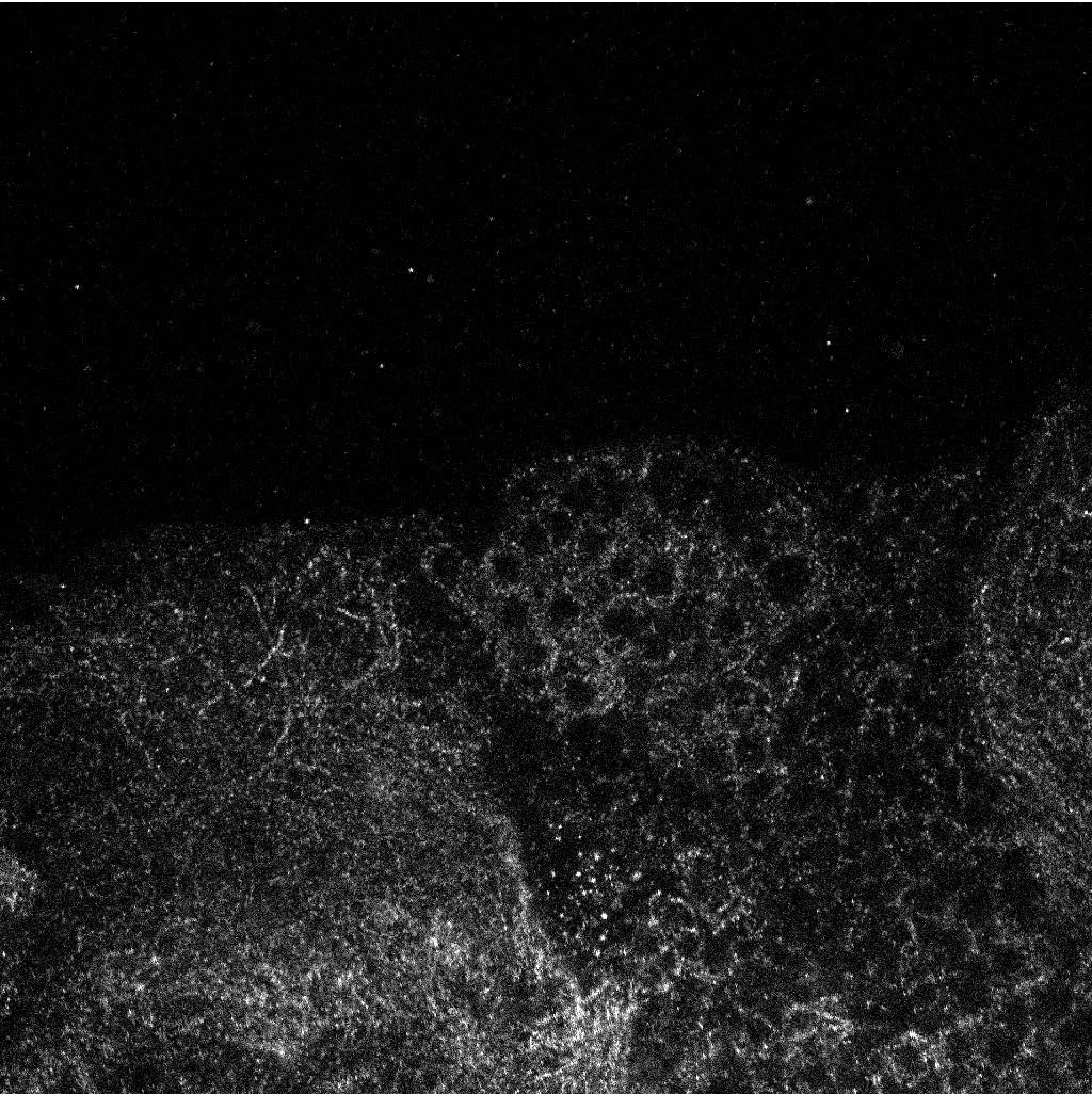

S6A_1.tif

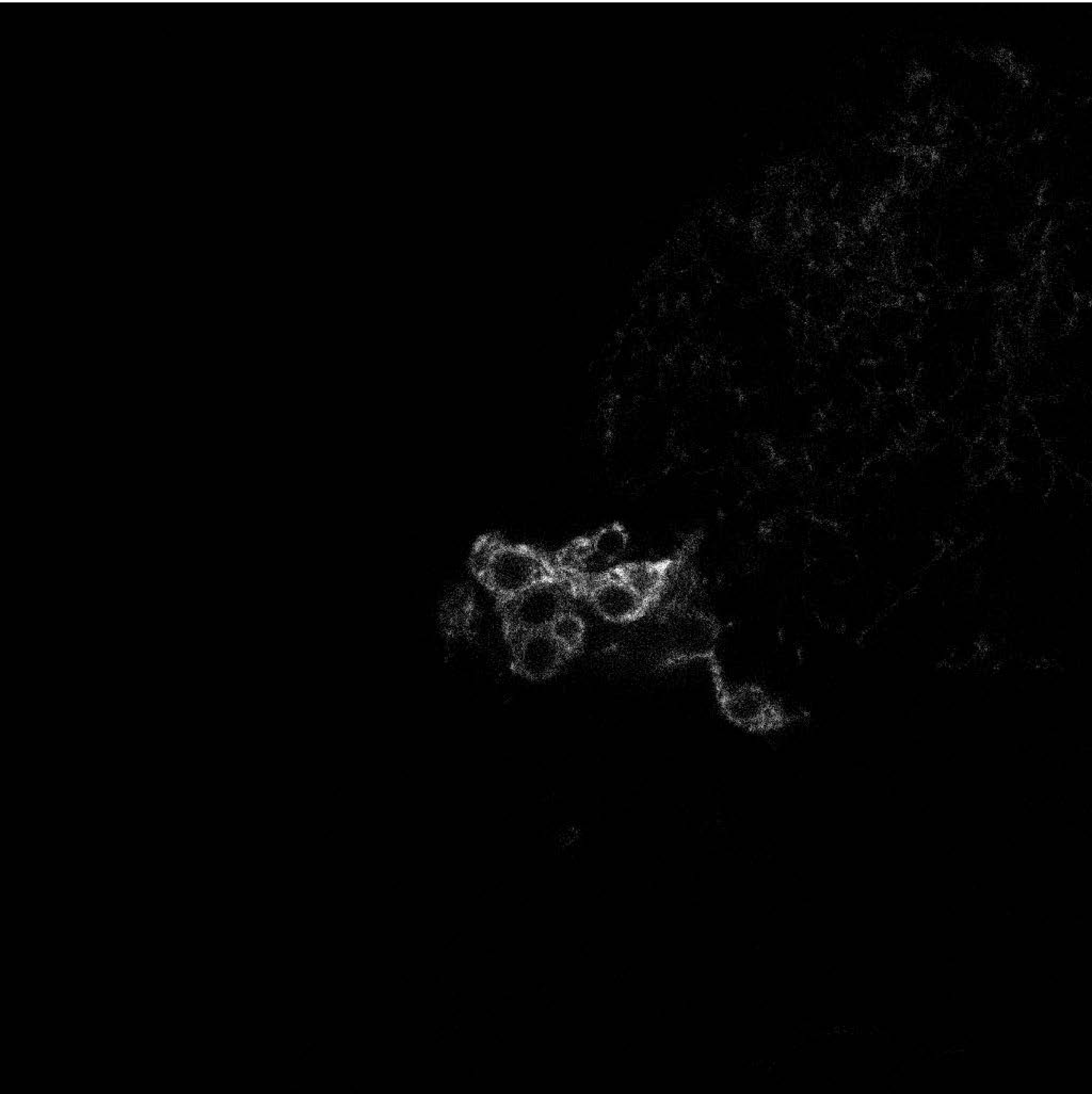

S6A_2.tif

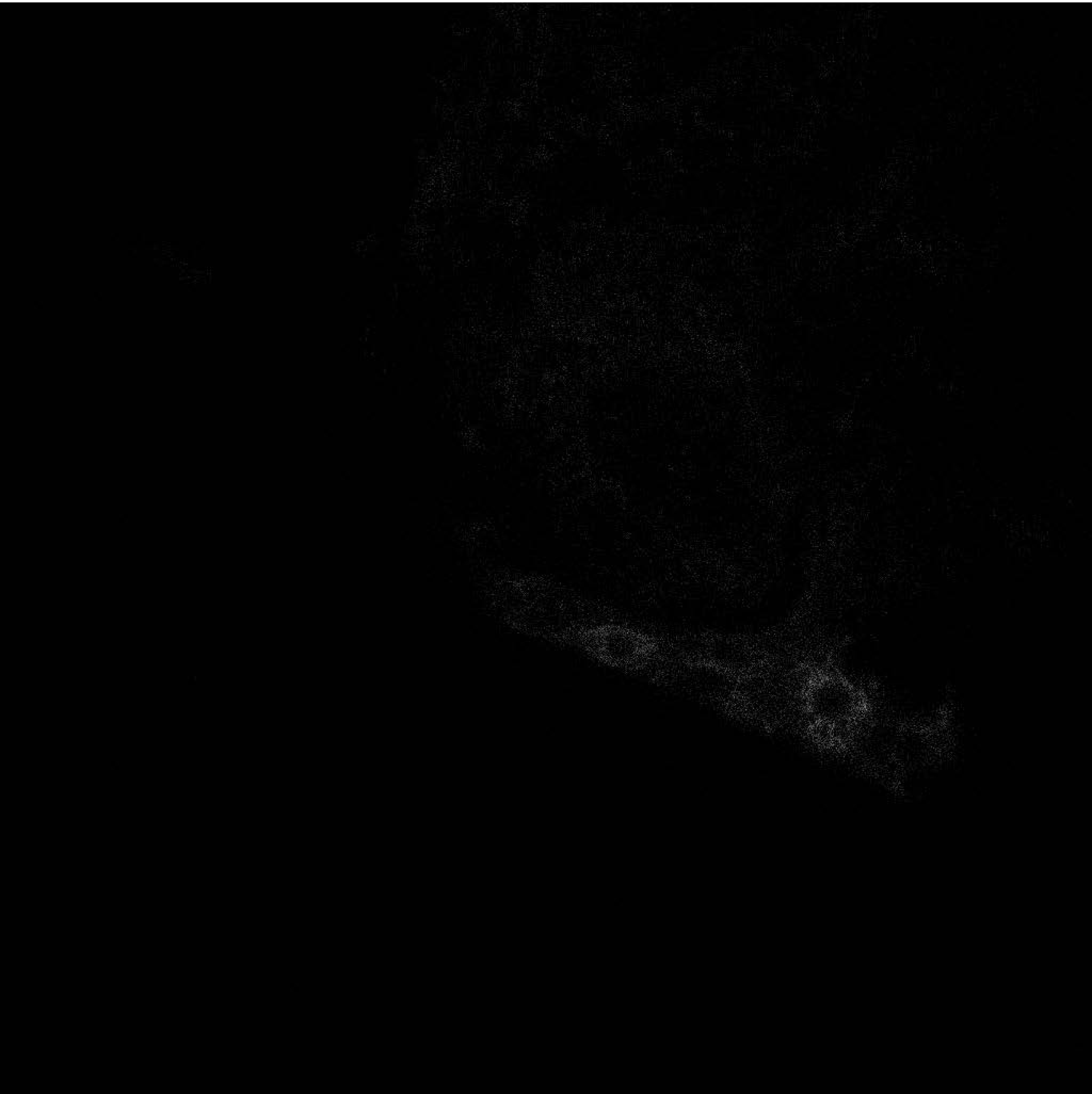

S6A_3.tif

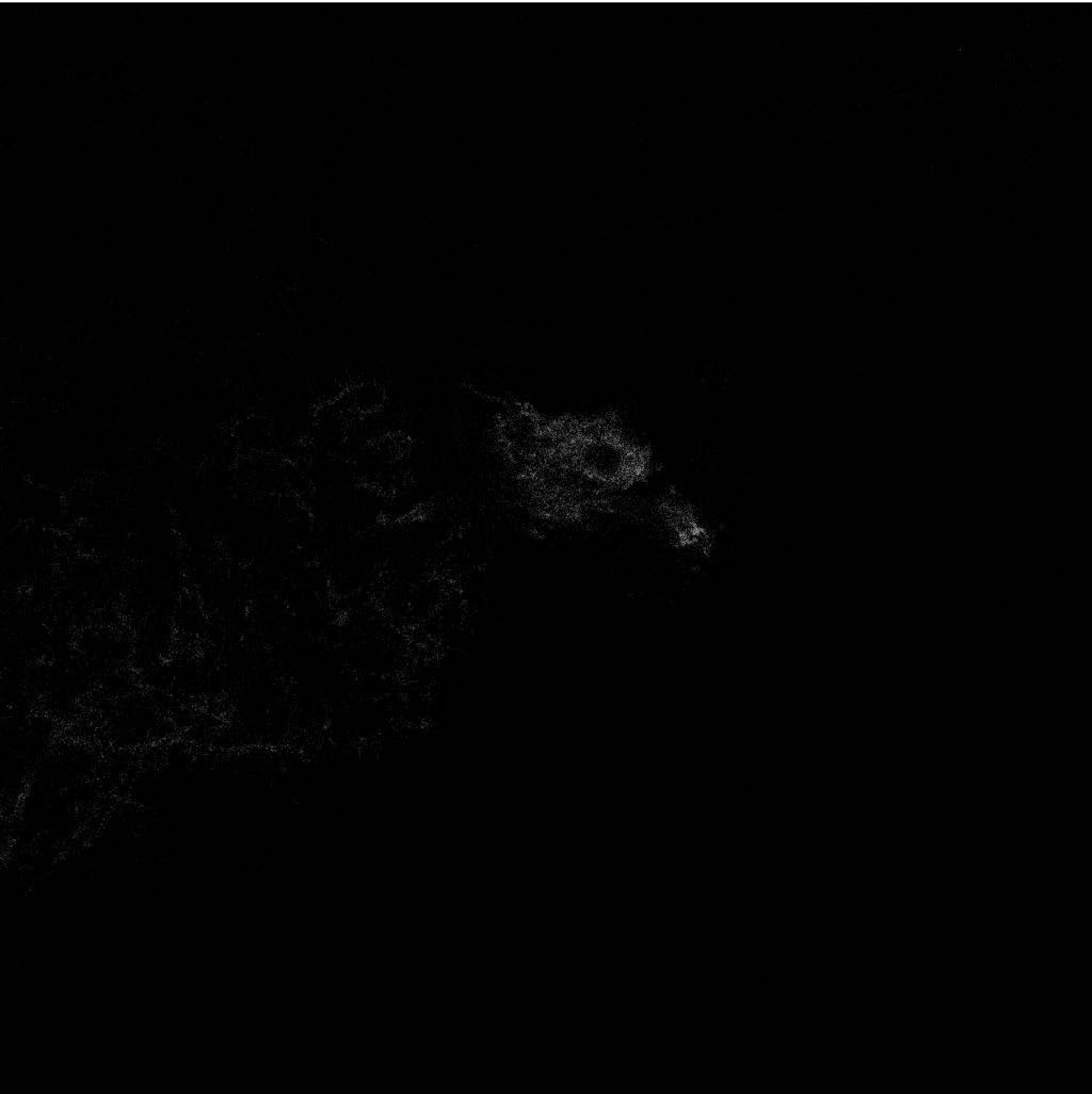

S6A_4.tif

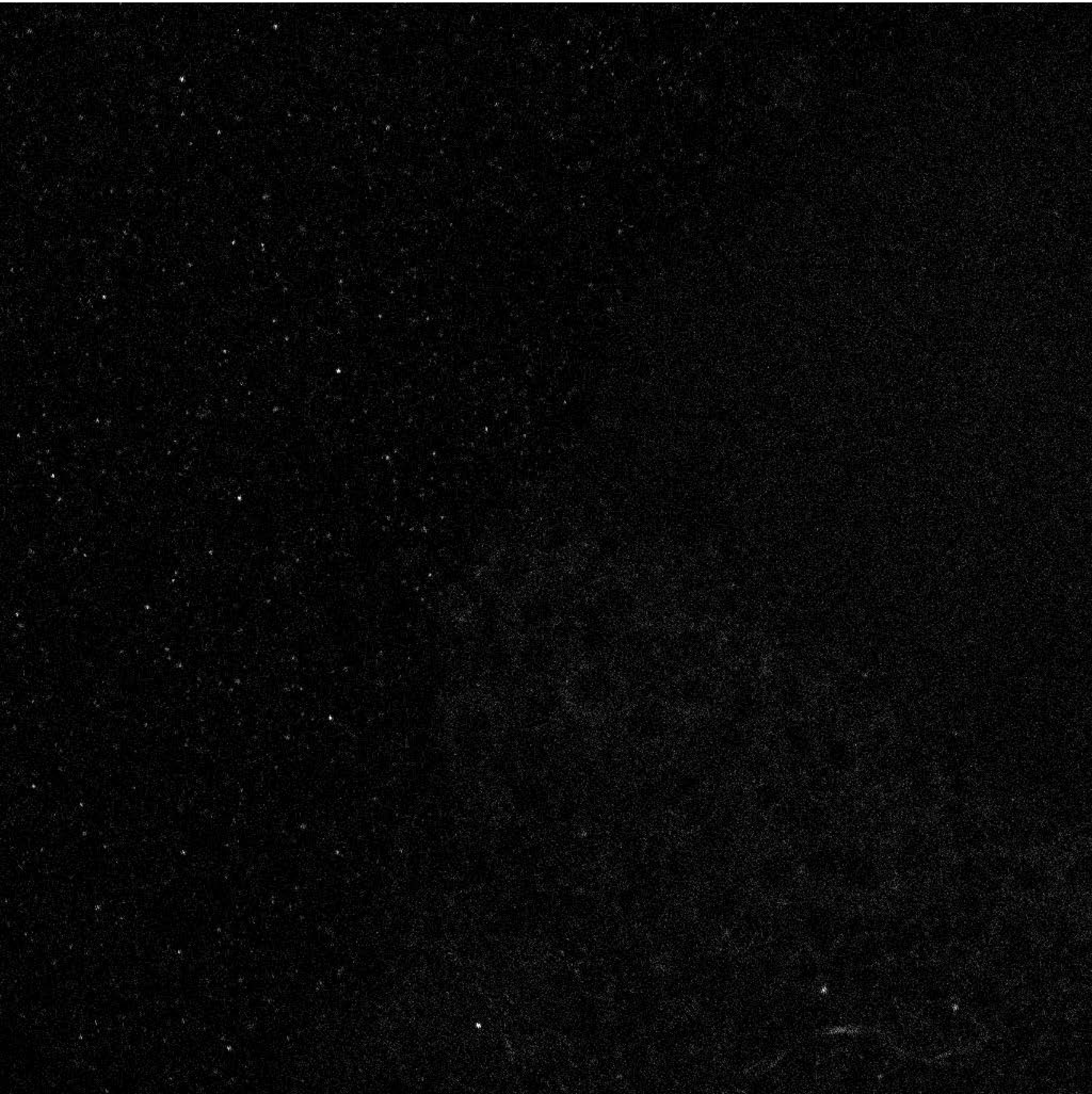

S6A_5.tif

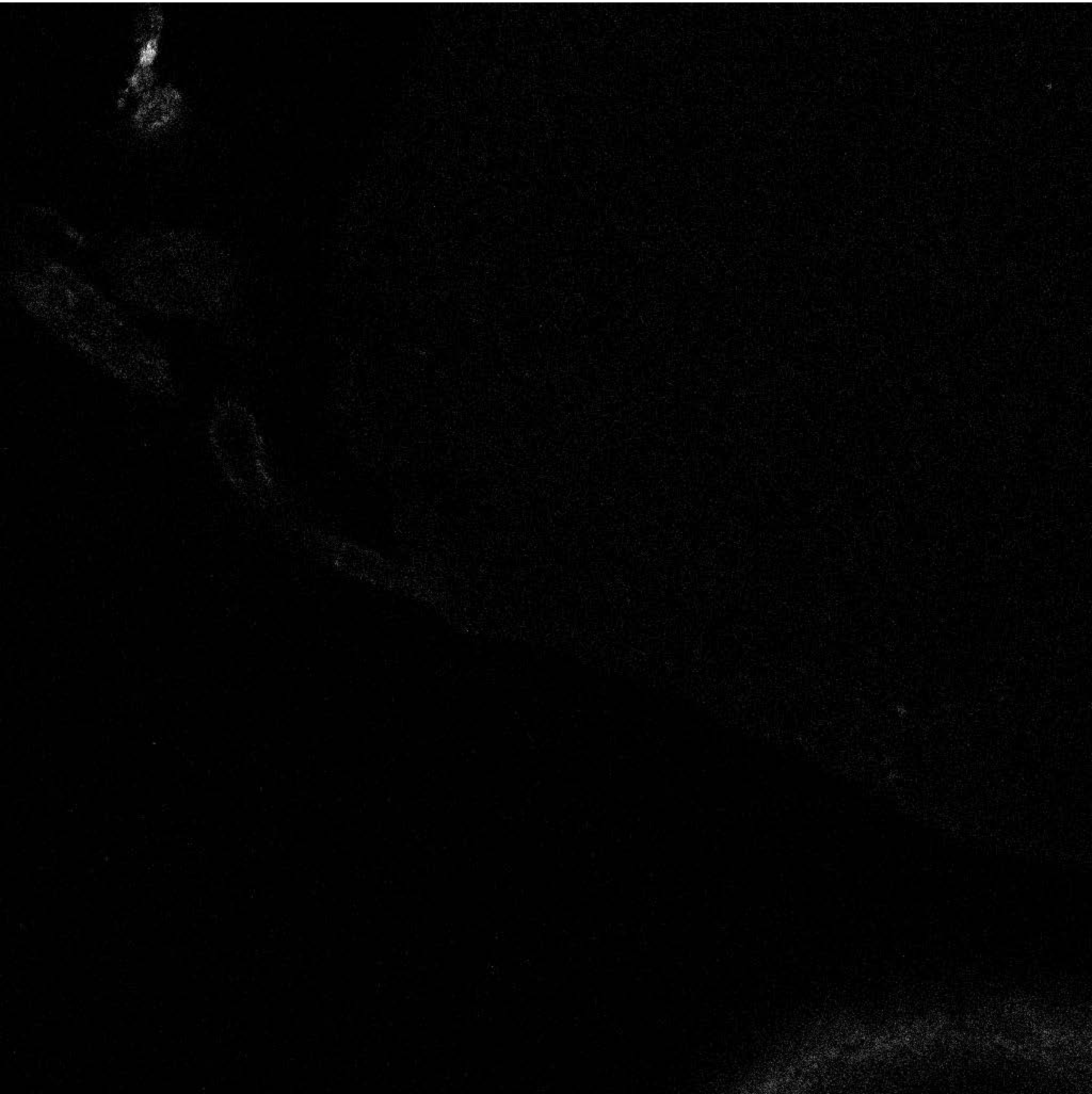

S6A_6.tif

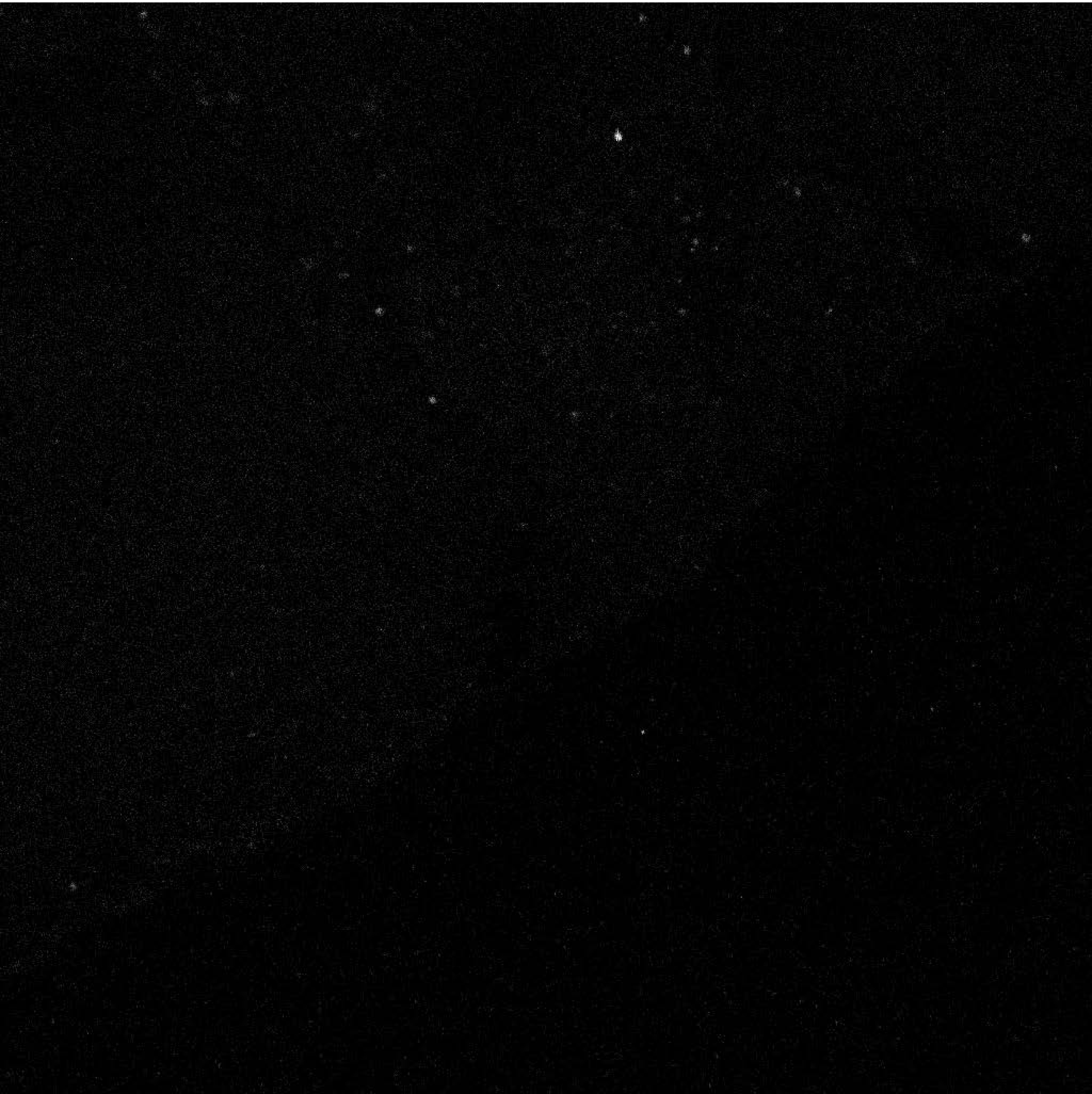

S6C_1A.tif

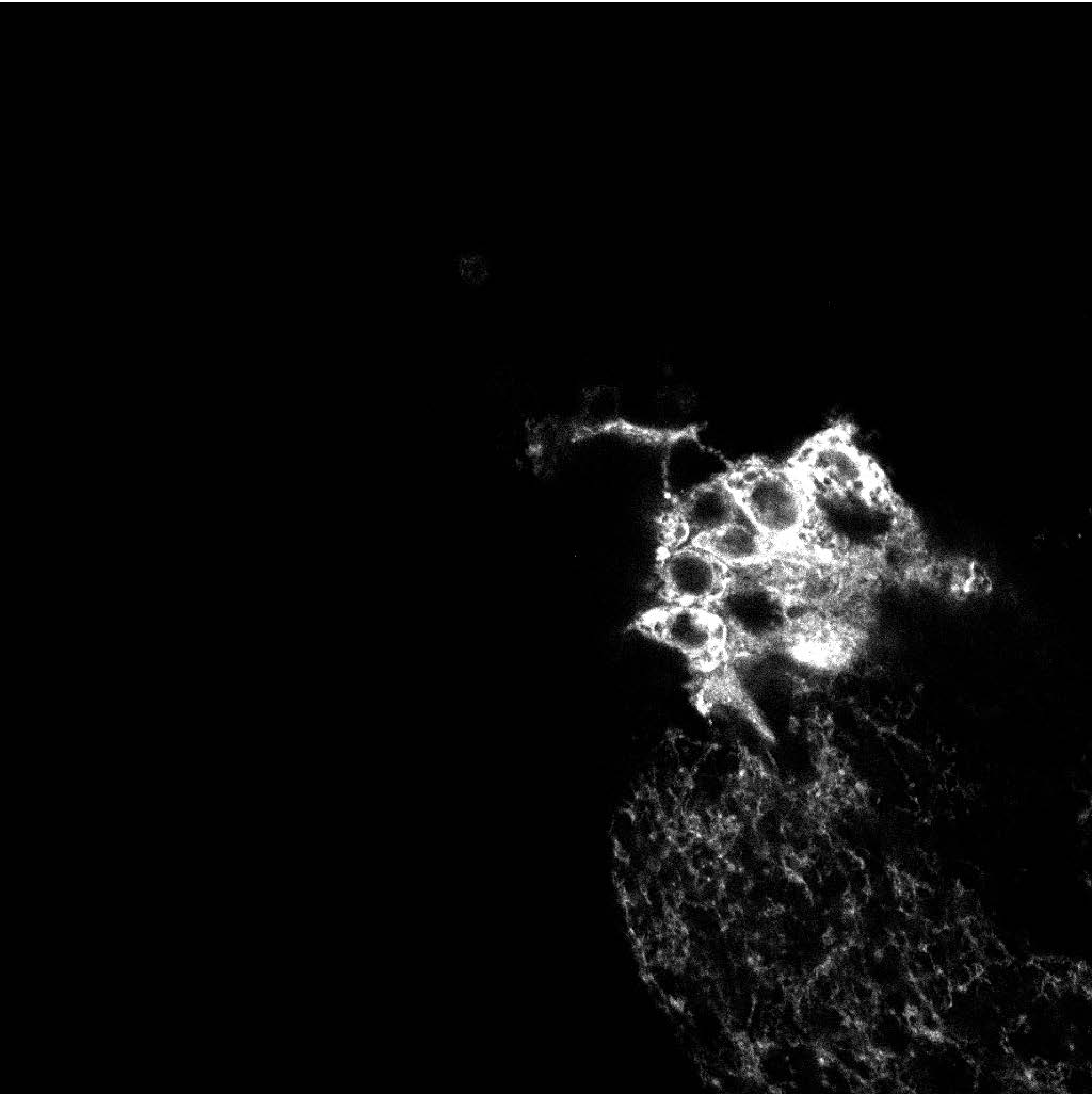

S6C_1B.tif

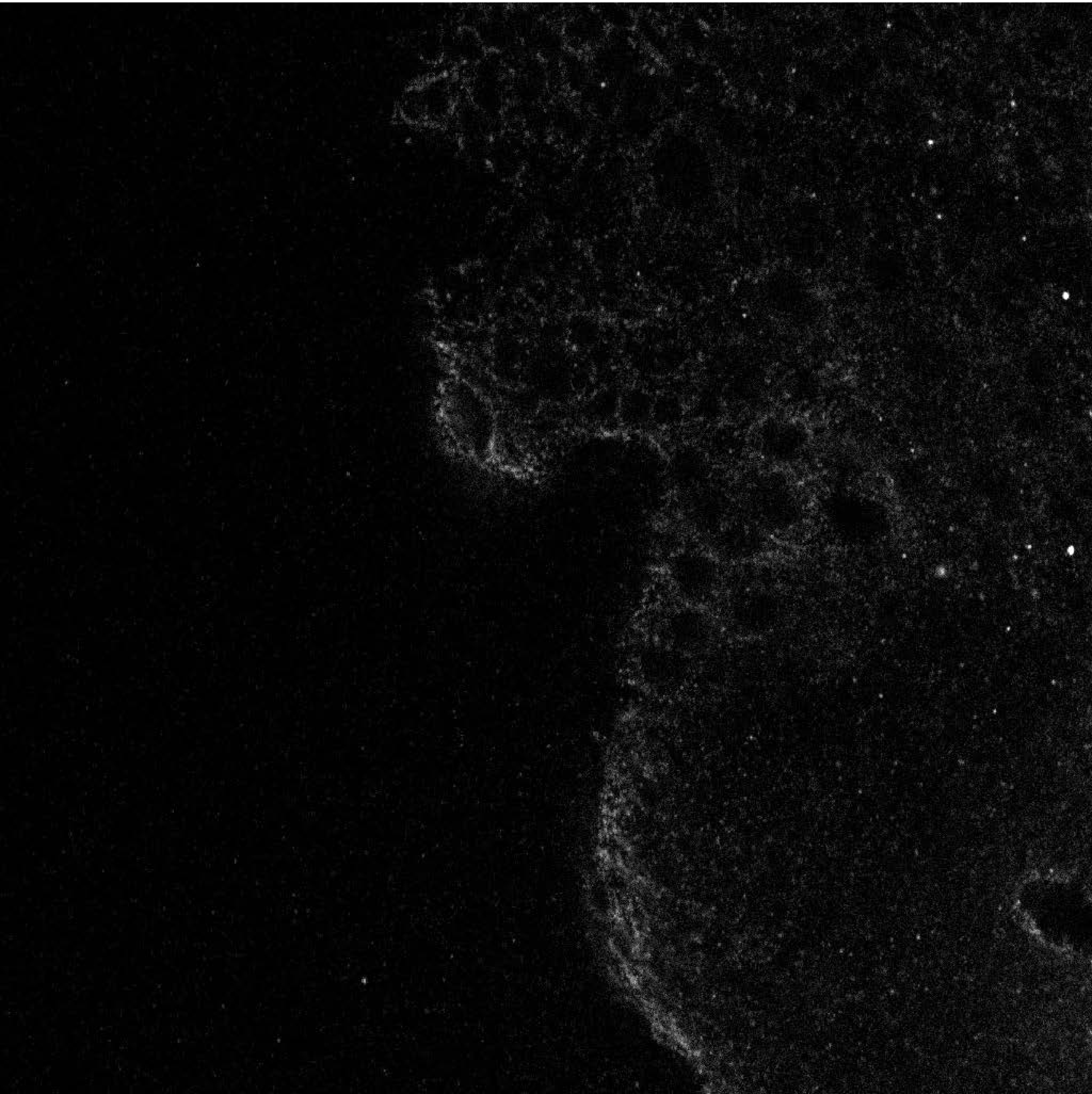

S6C_2A.tif

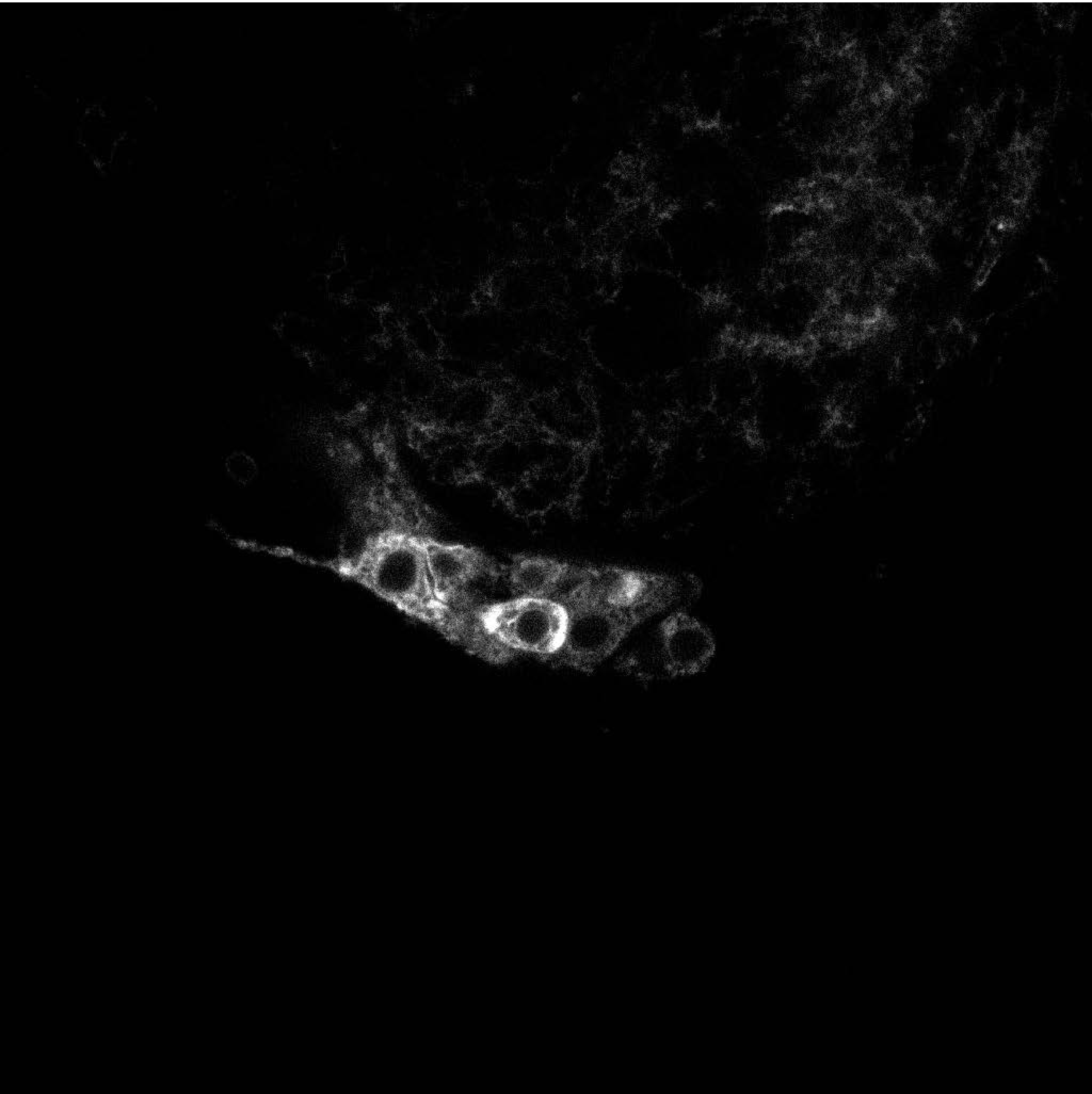

S6C_2B.tif

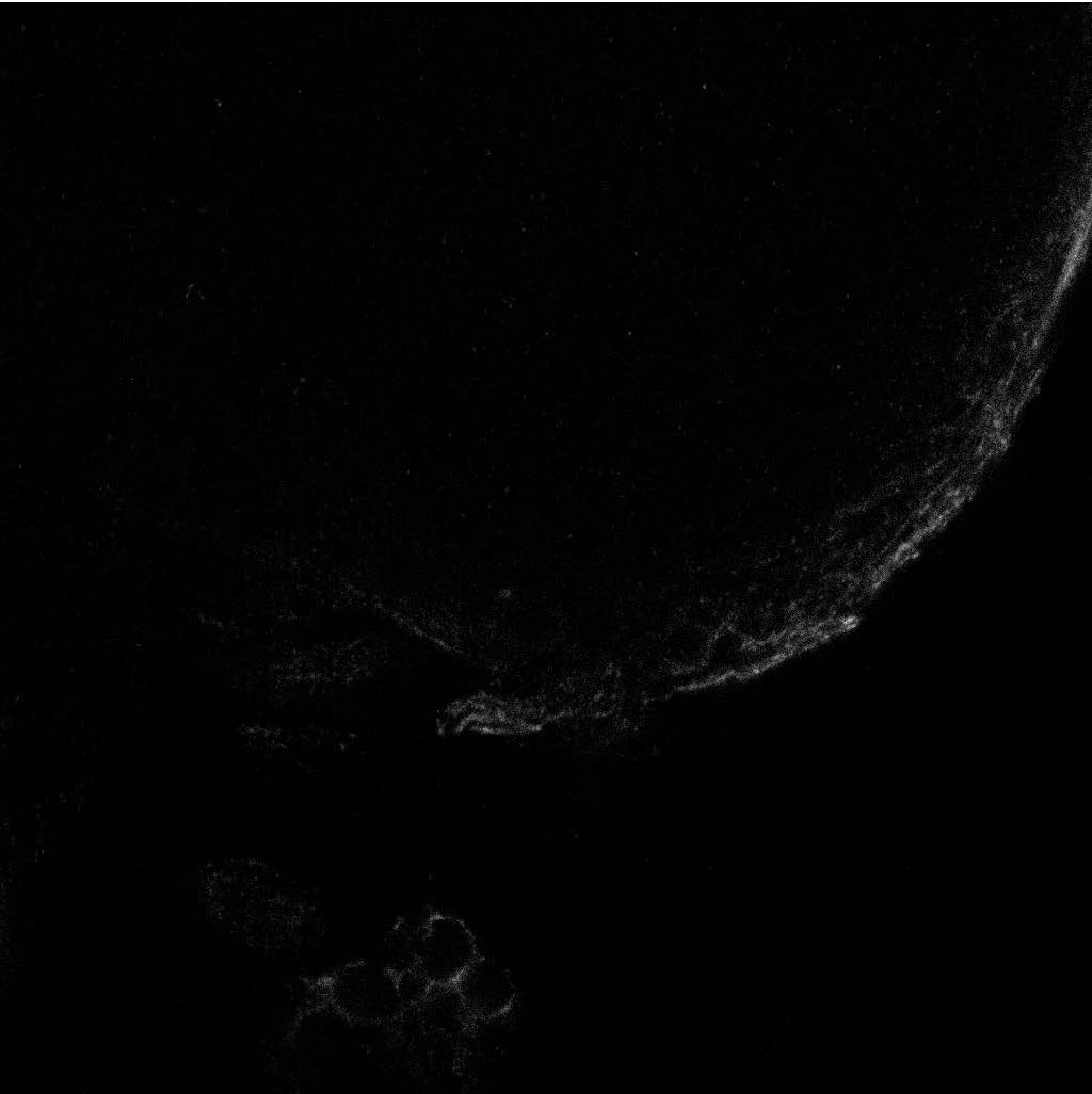

S6C_2.tif

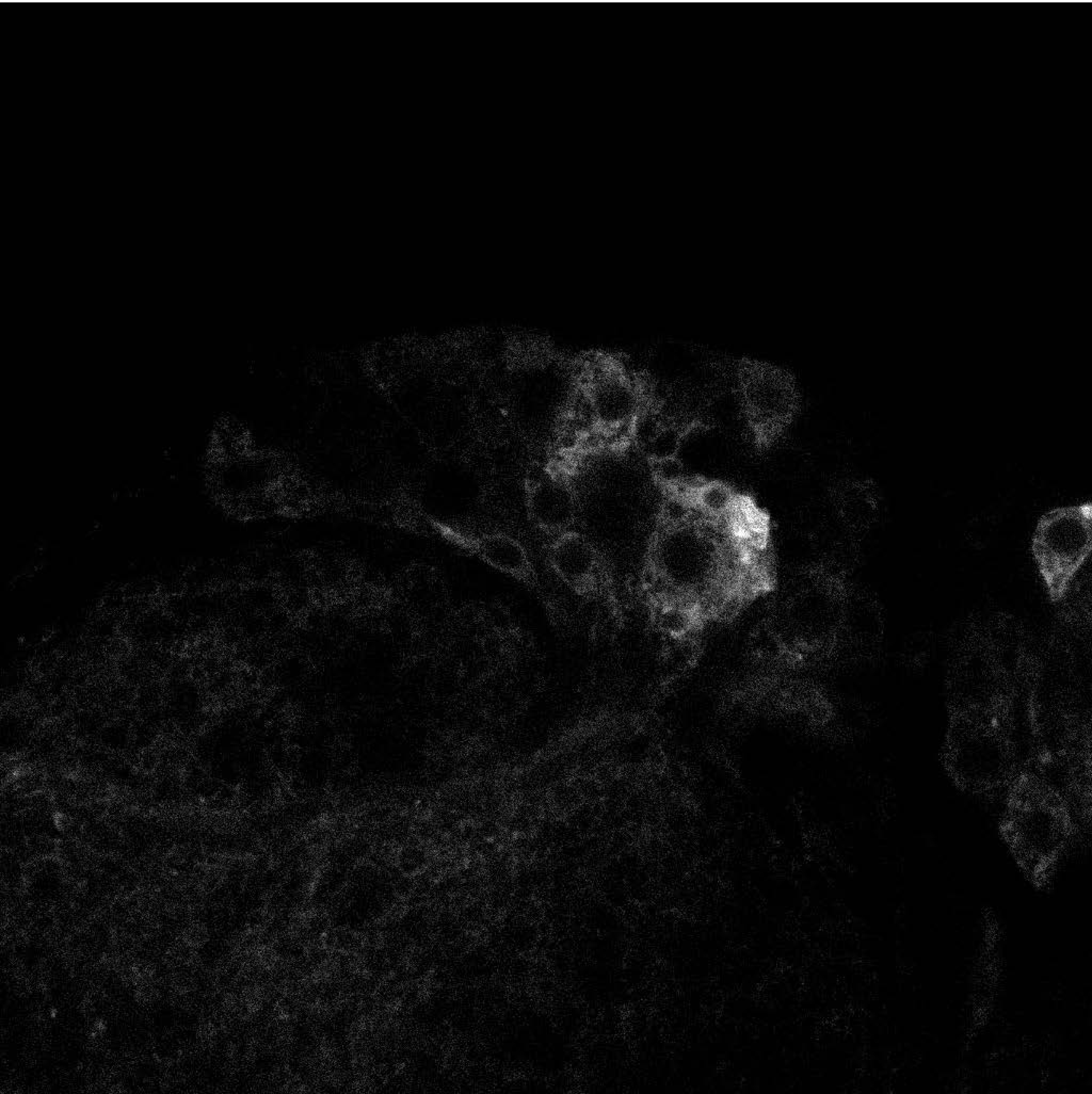

S6C_3A.tif

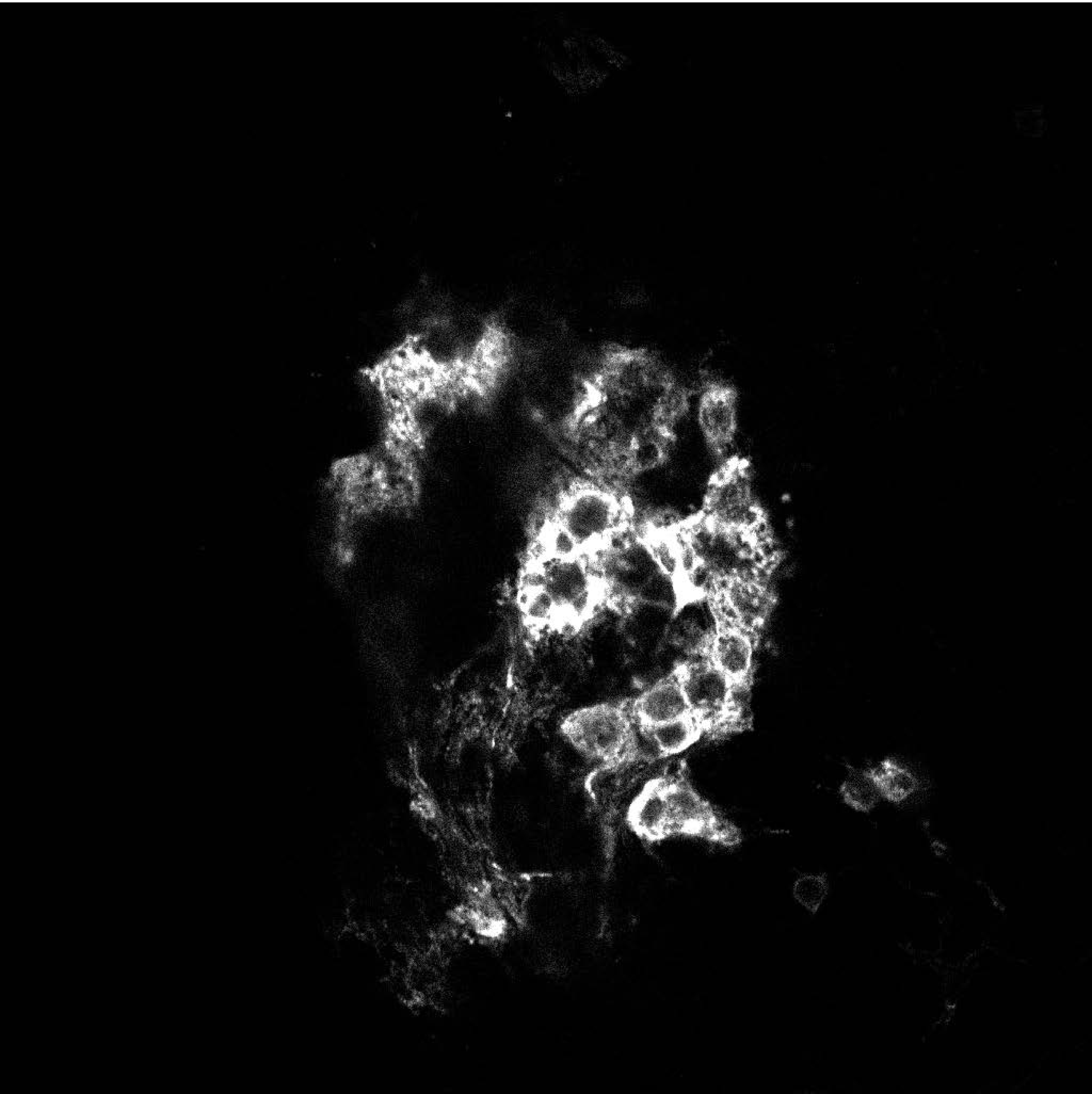

S6C_3B.tif

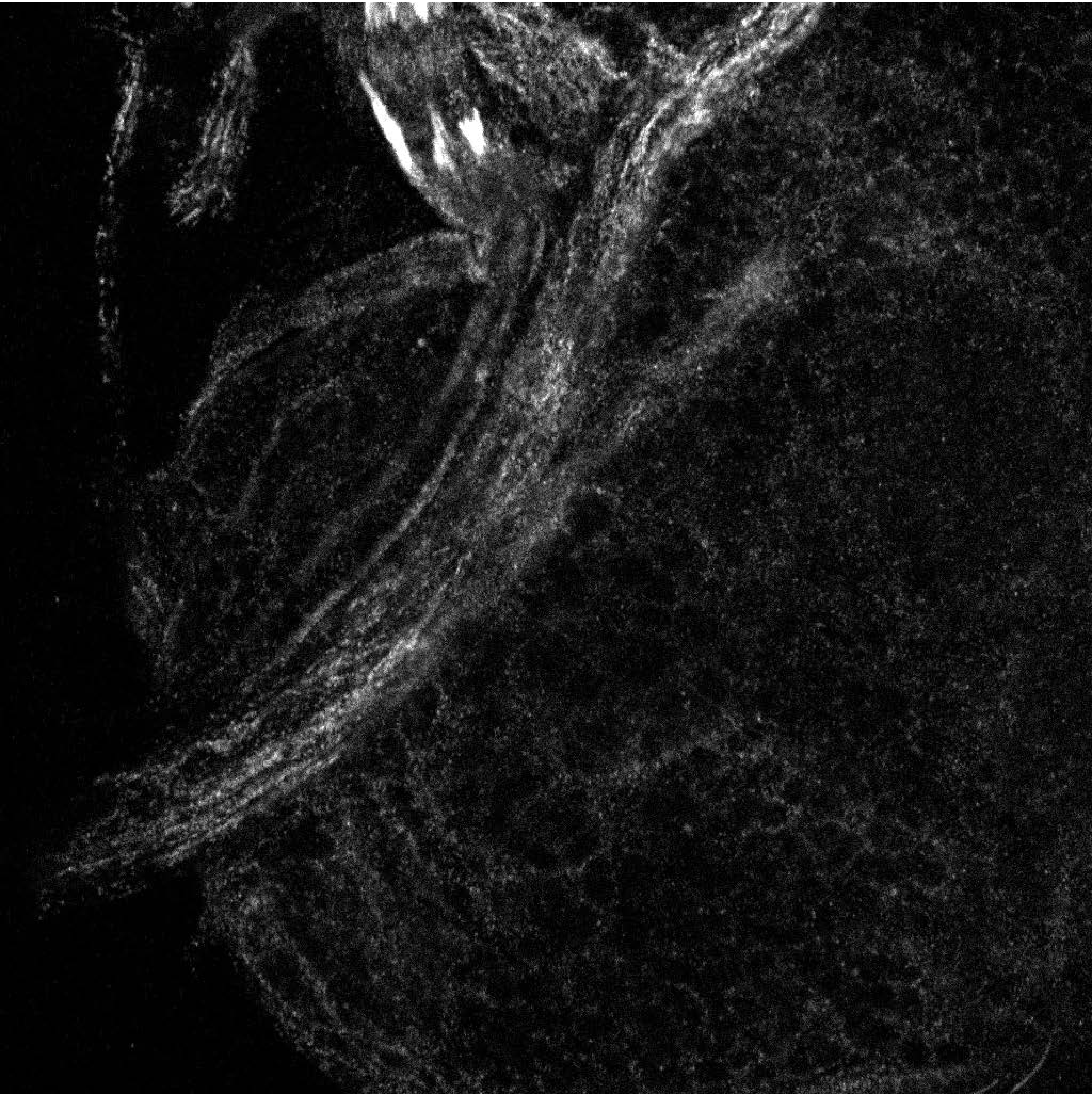

S6C_3.tif

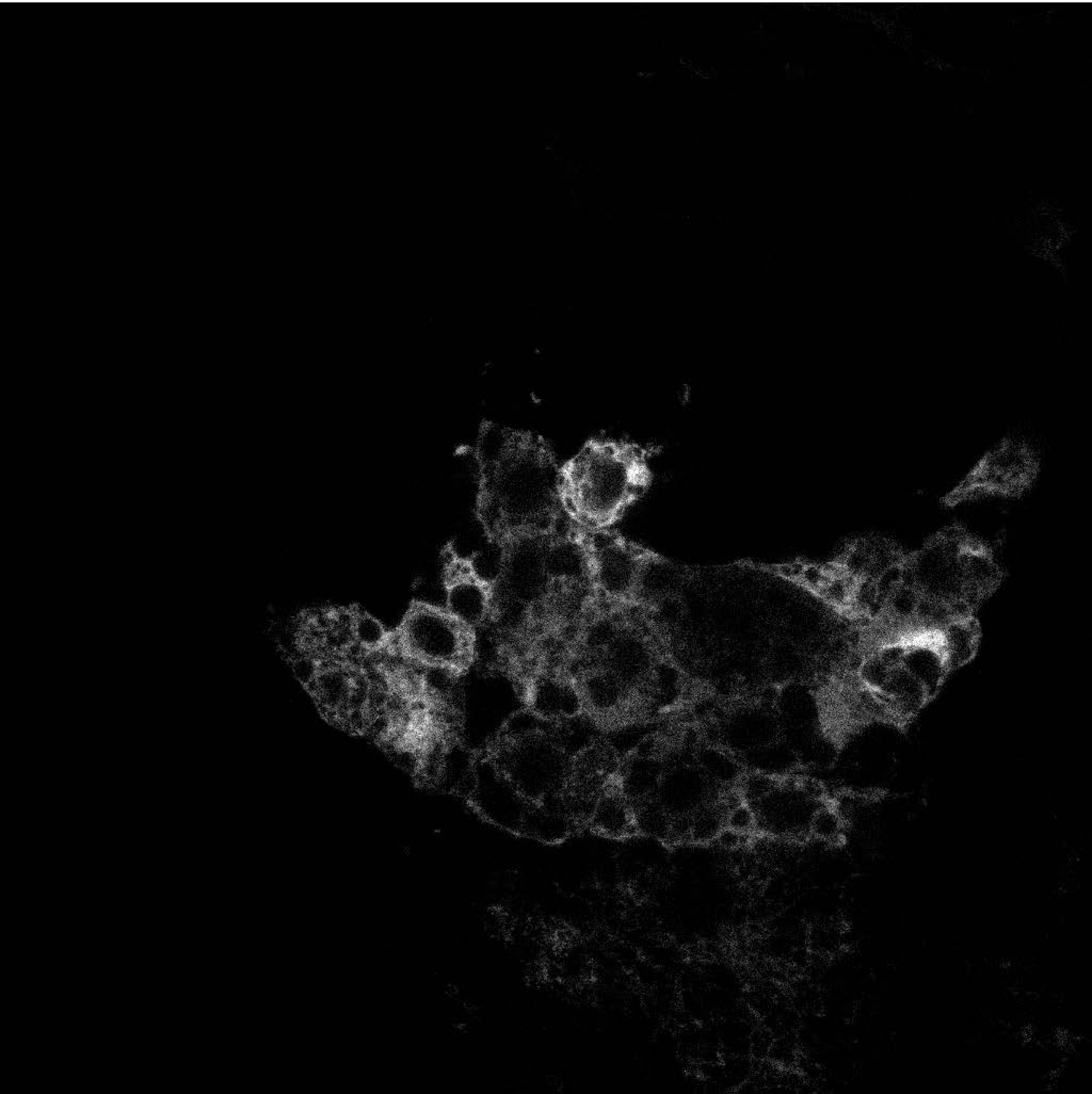

S6C_4A.tif

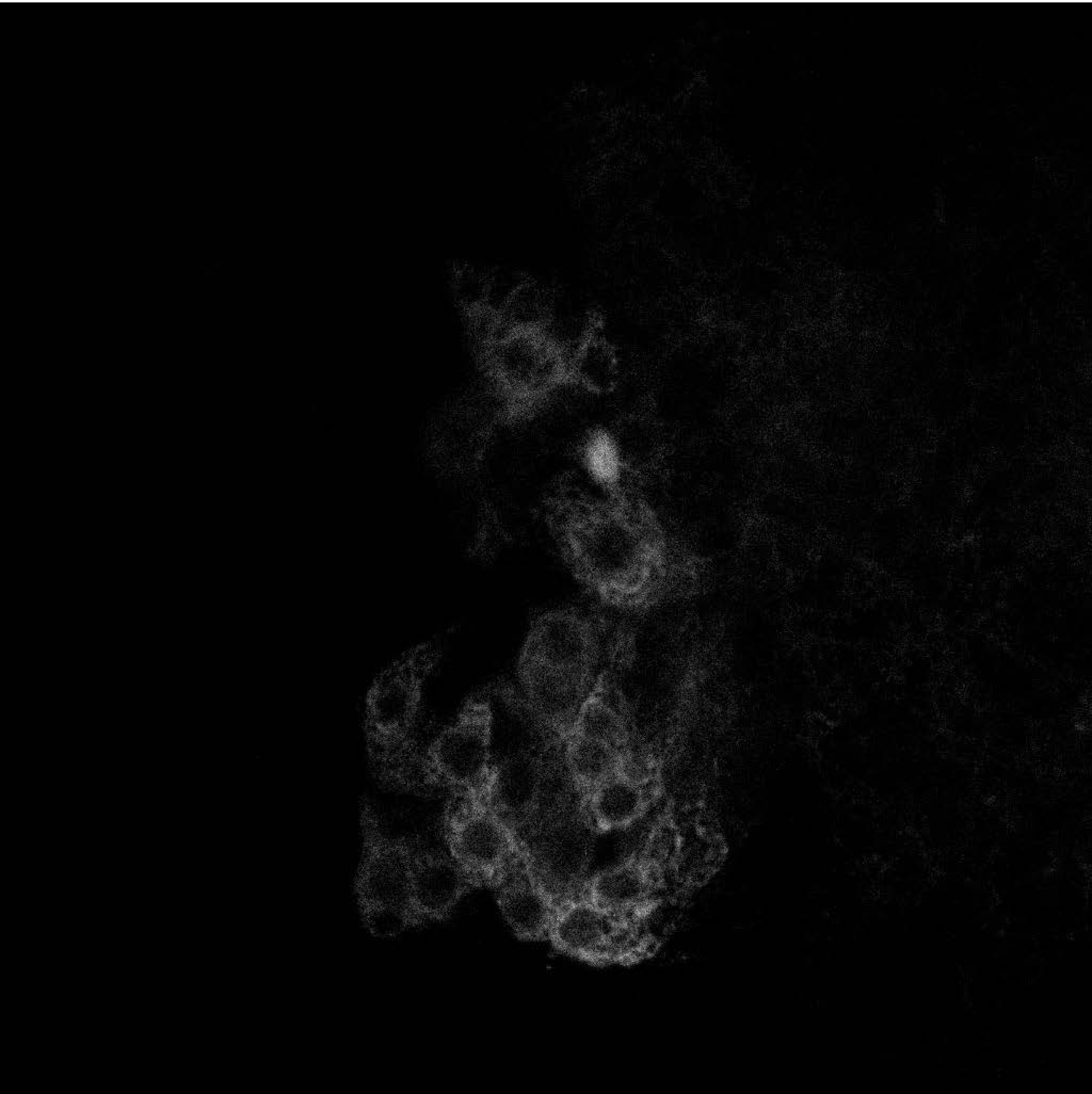

S6C_4B.tif

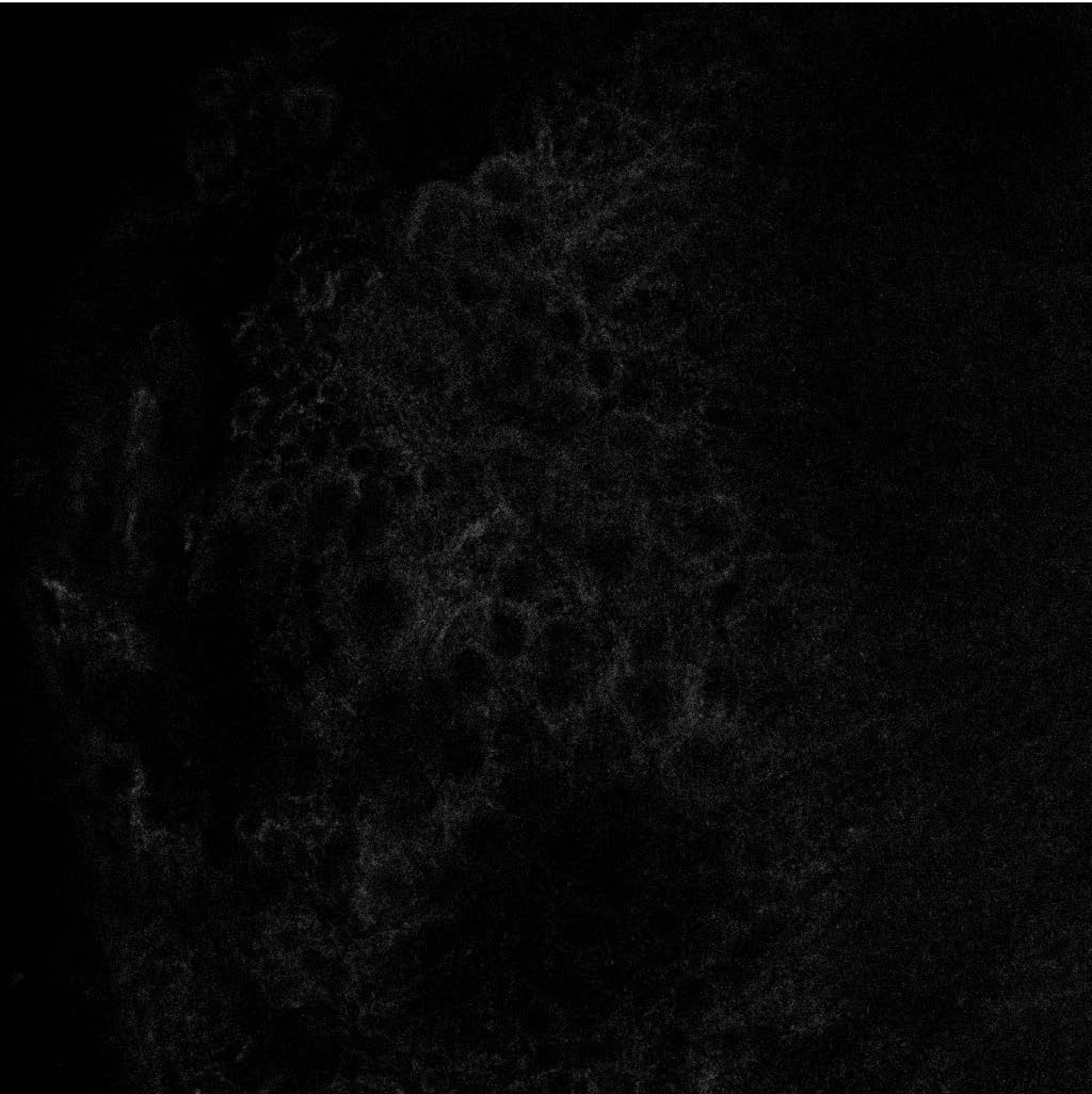

S6C_5A.tif

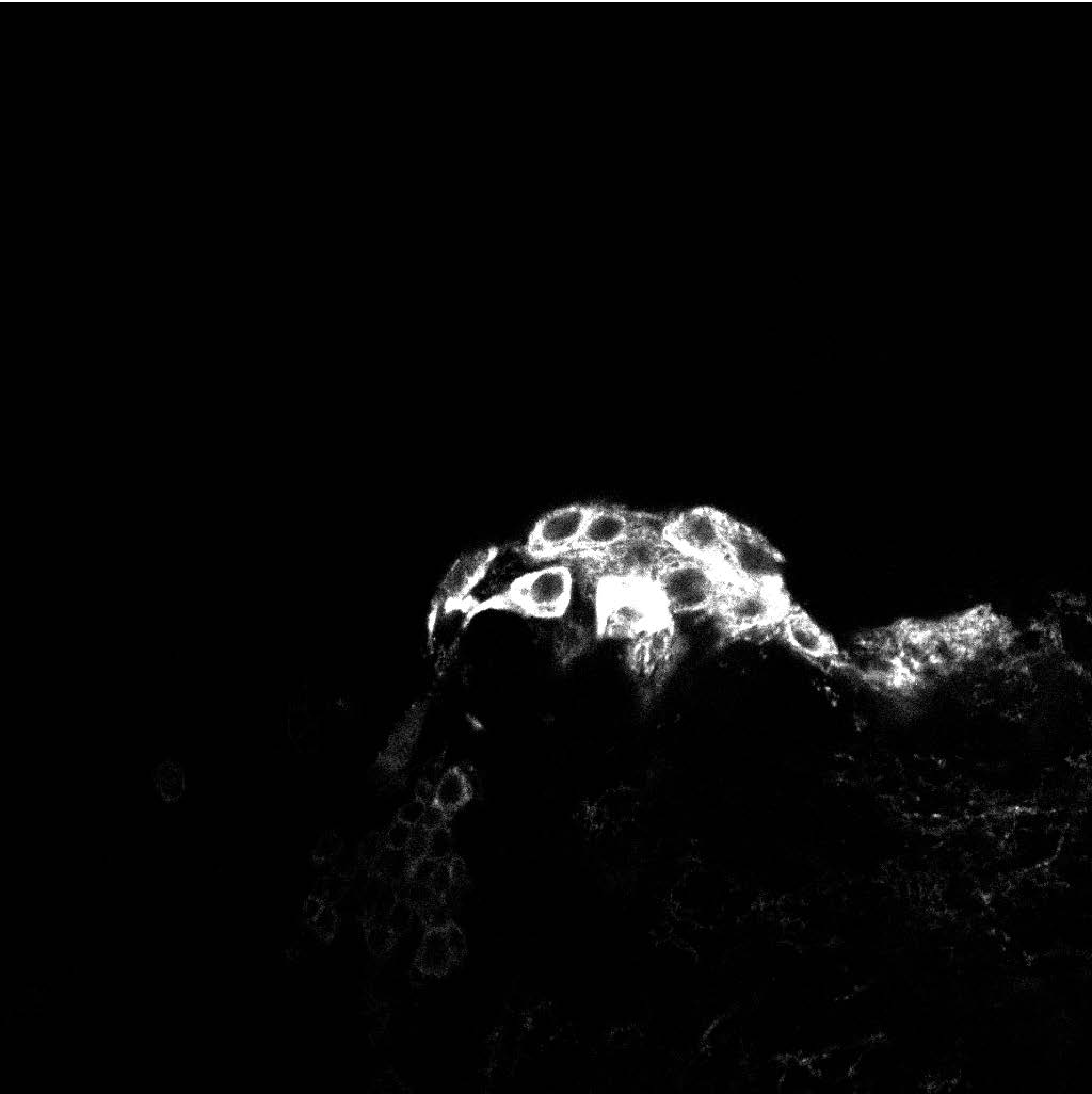

S6C_5B.tif

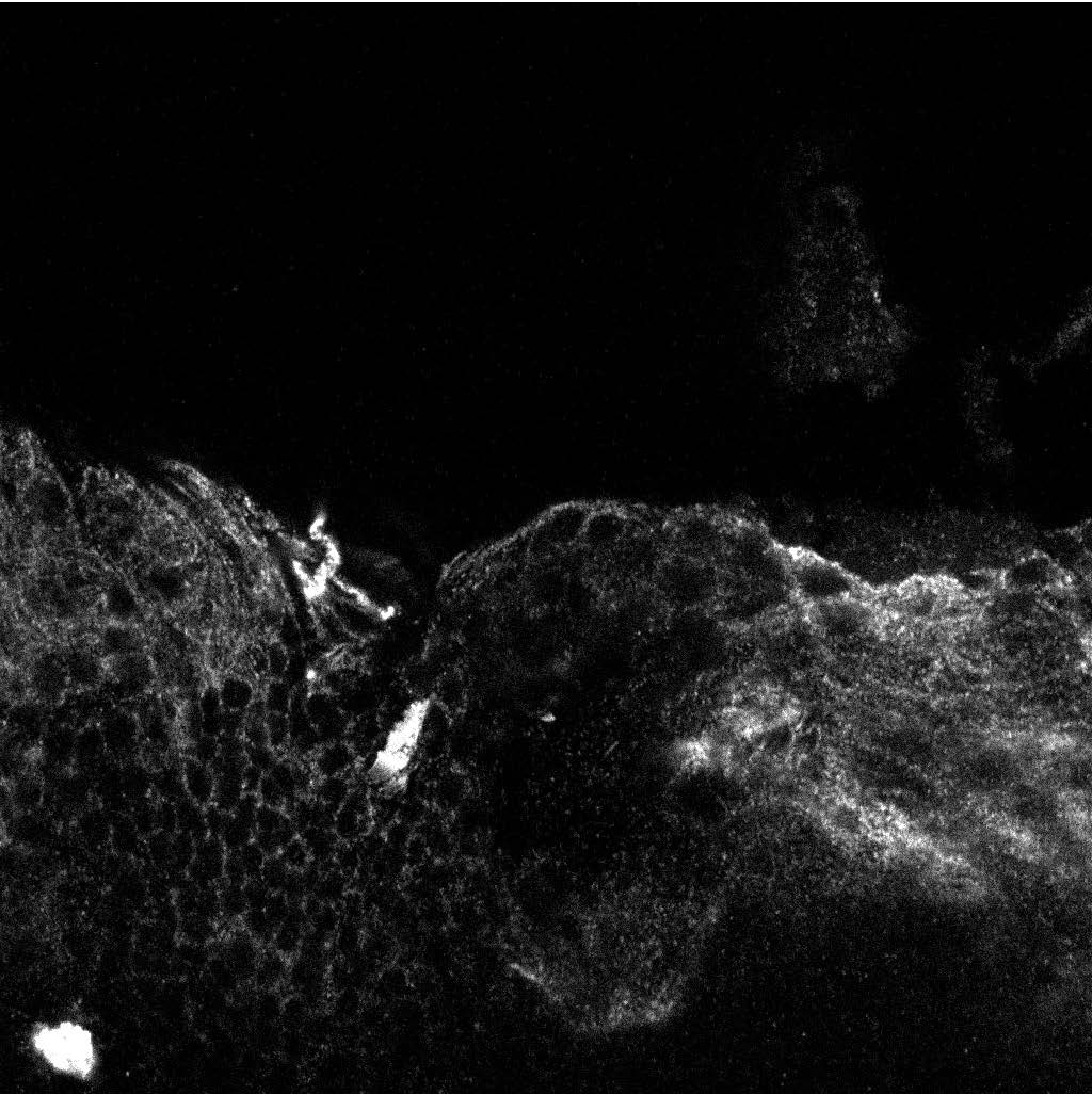

S6C_5.tif

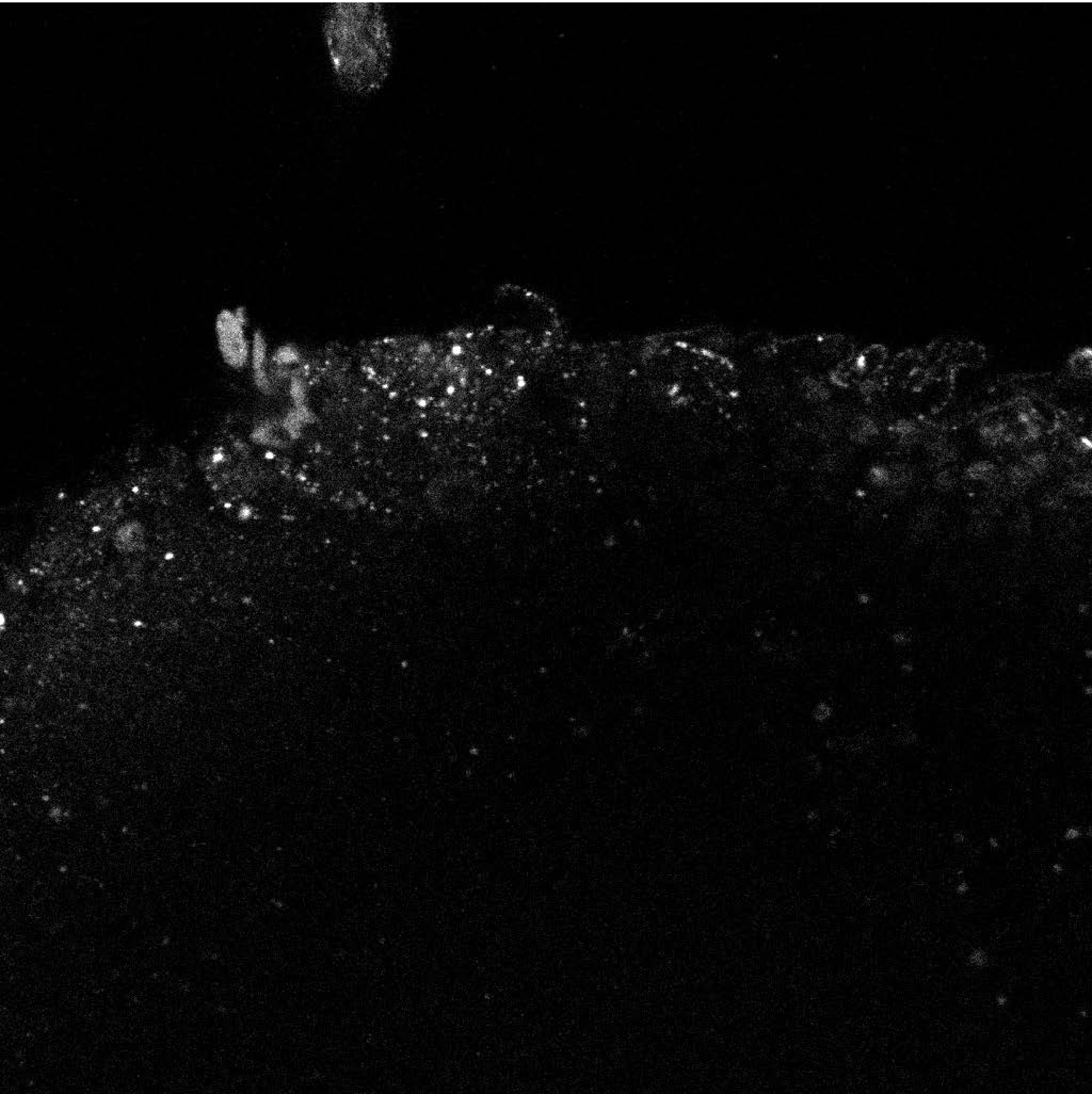

S6C_6A.tif

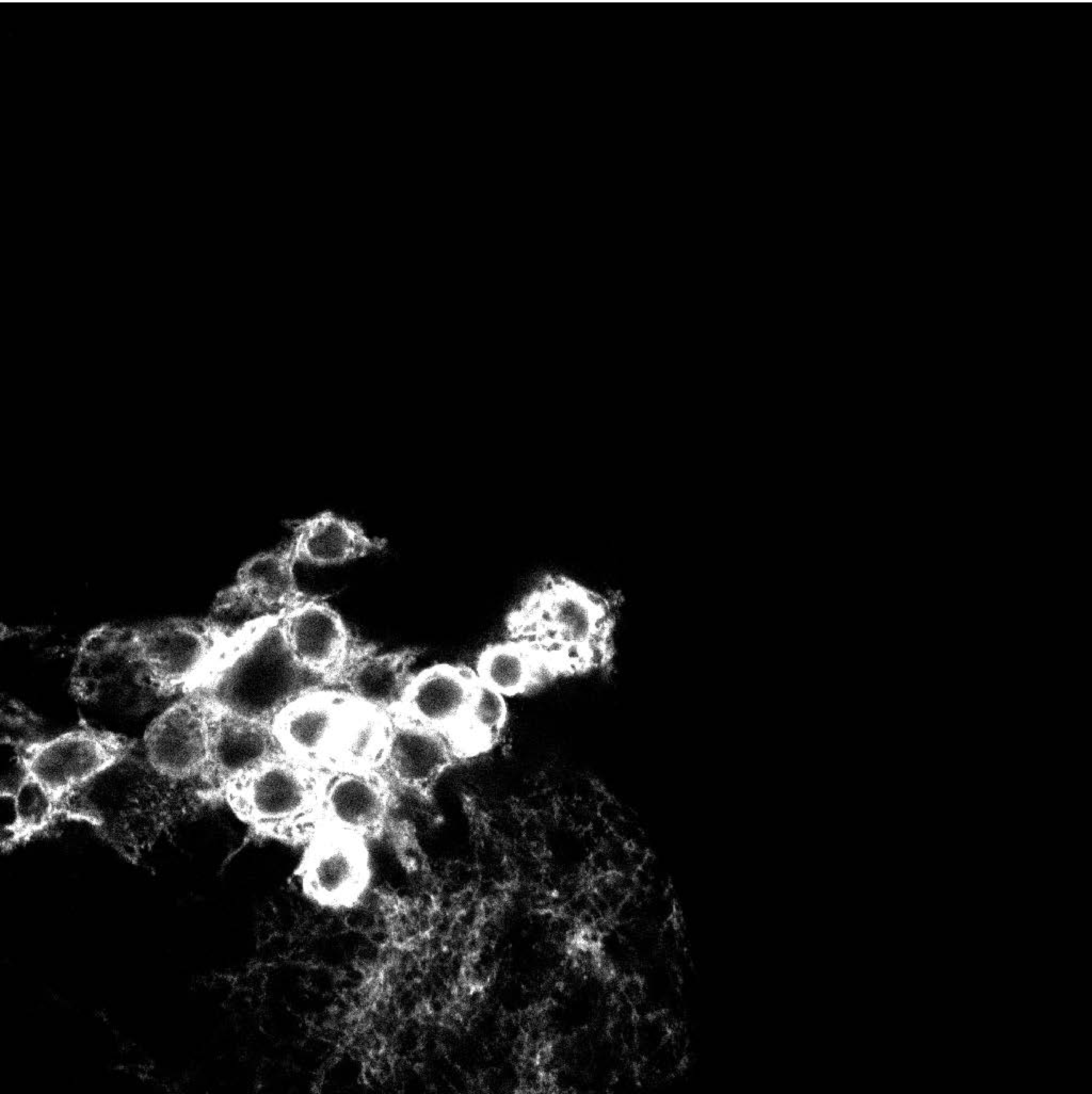

S6C_6B.tif

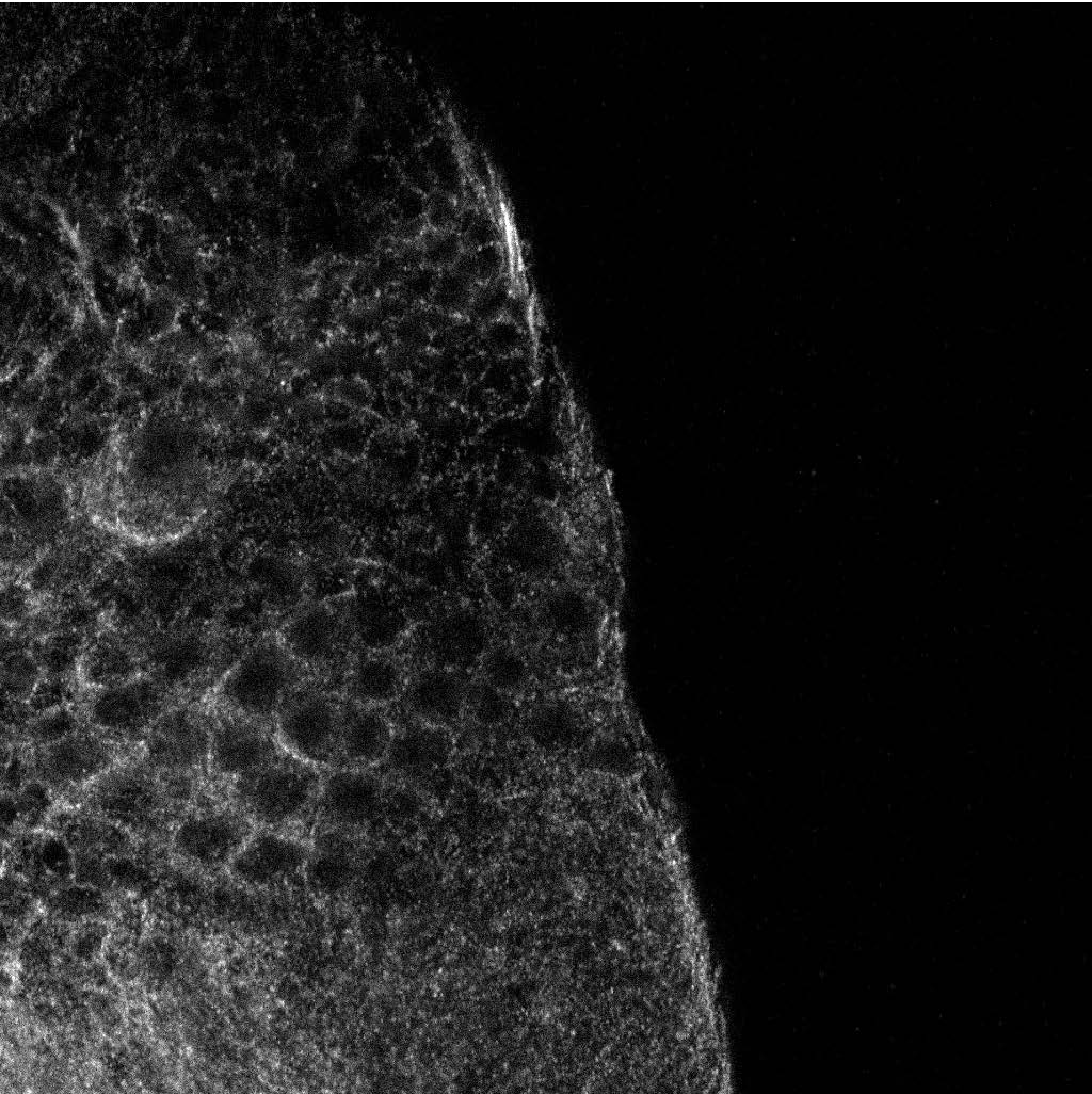

S6C_6.tif

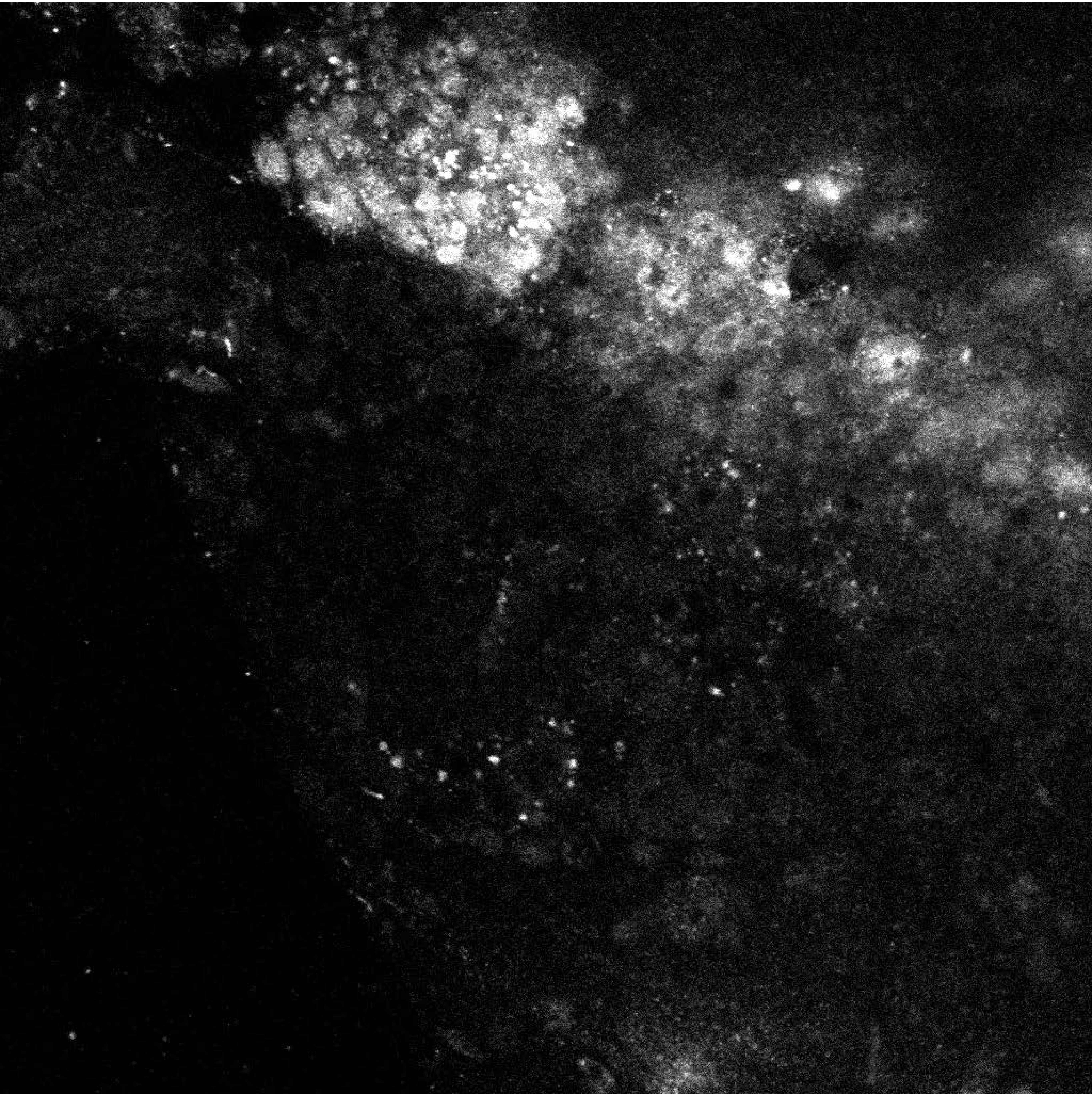

S6C_7A.tif

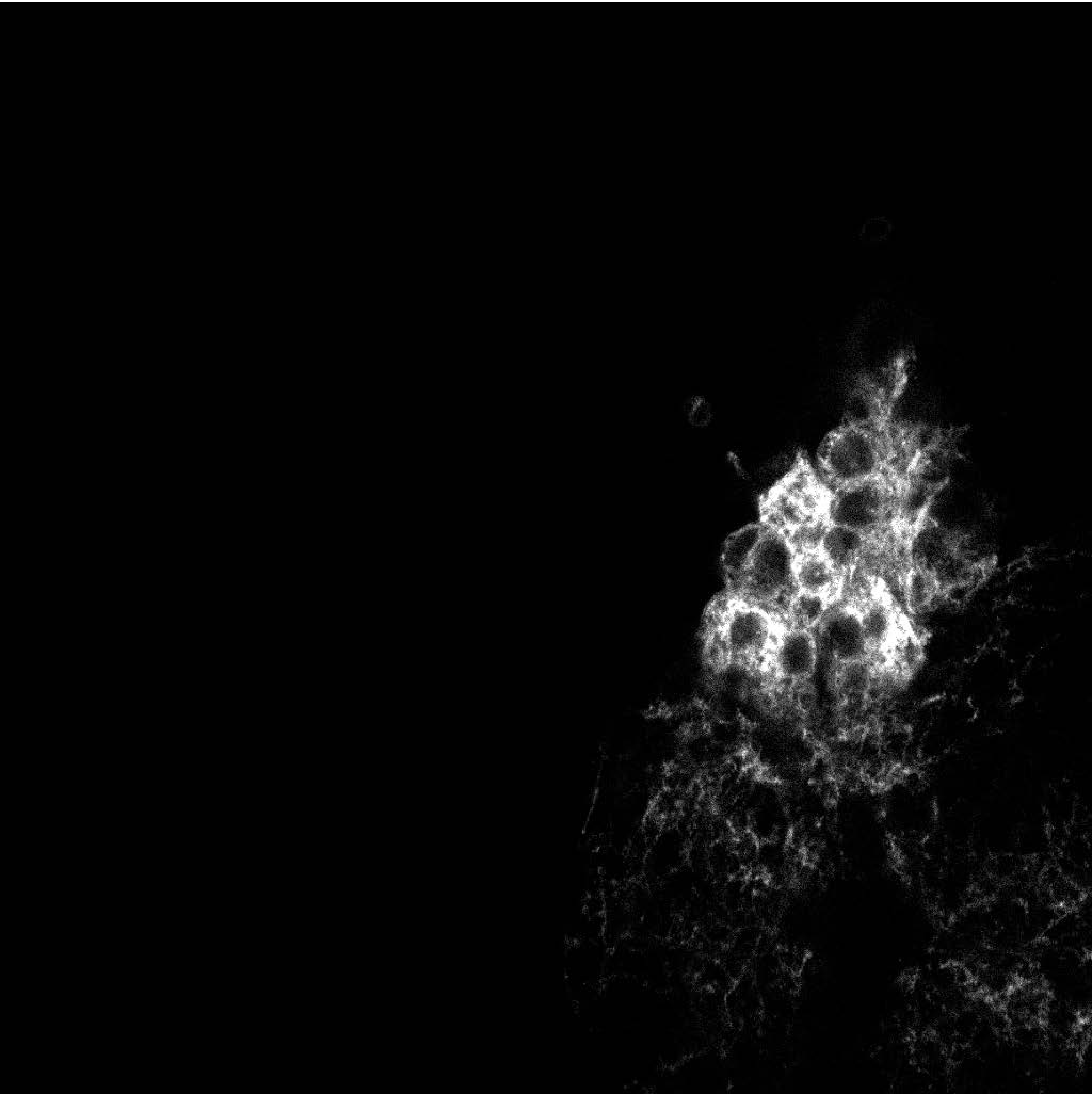

S6C_7B.tif

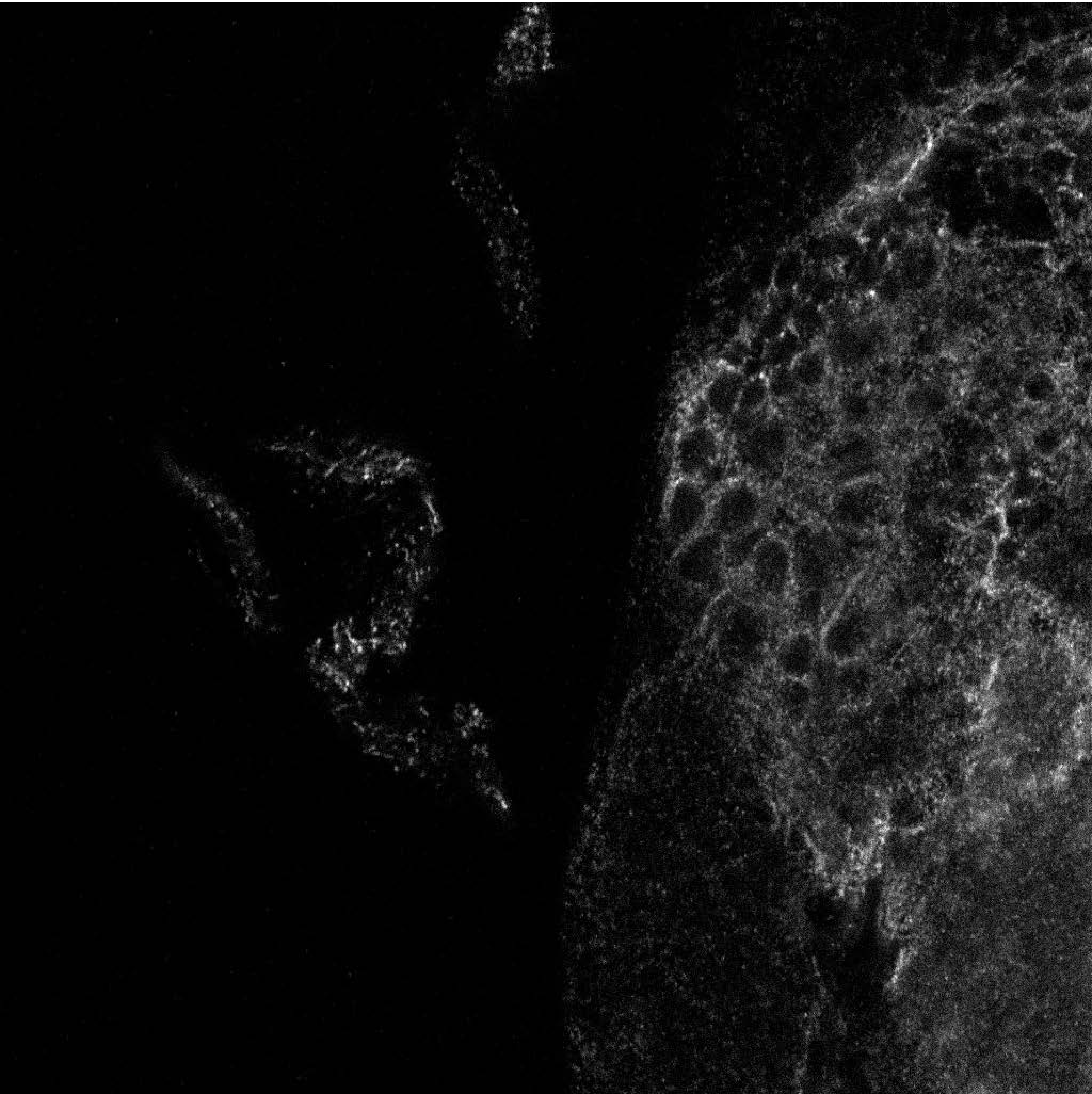

S6C_8A.tif

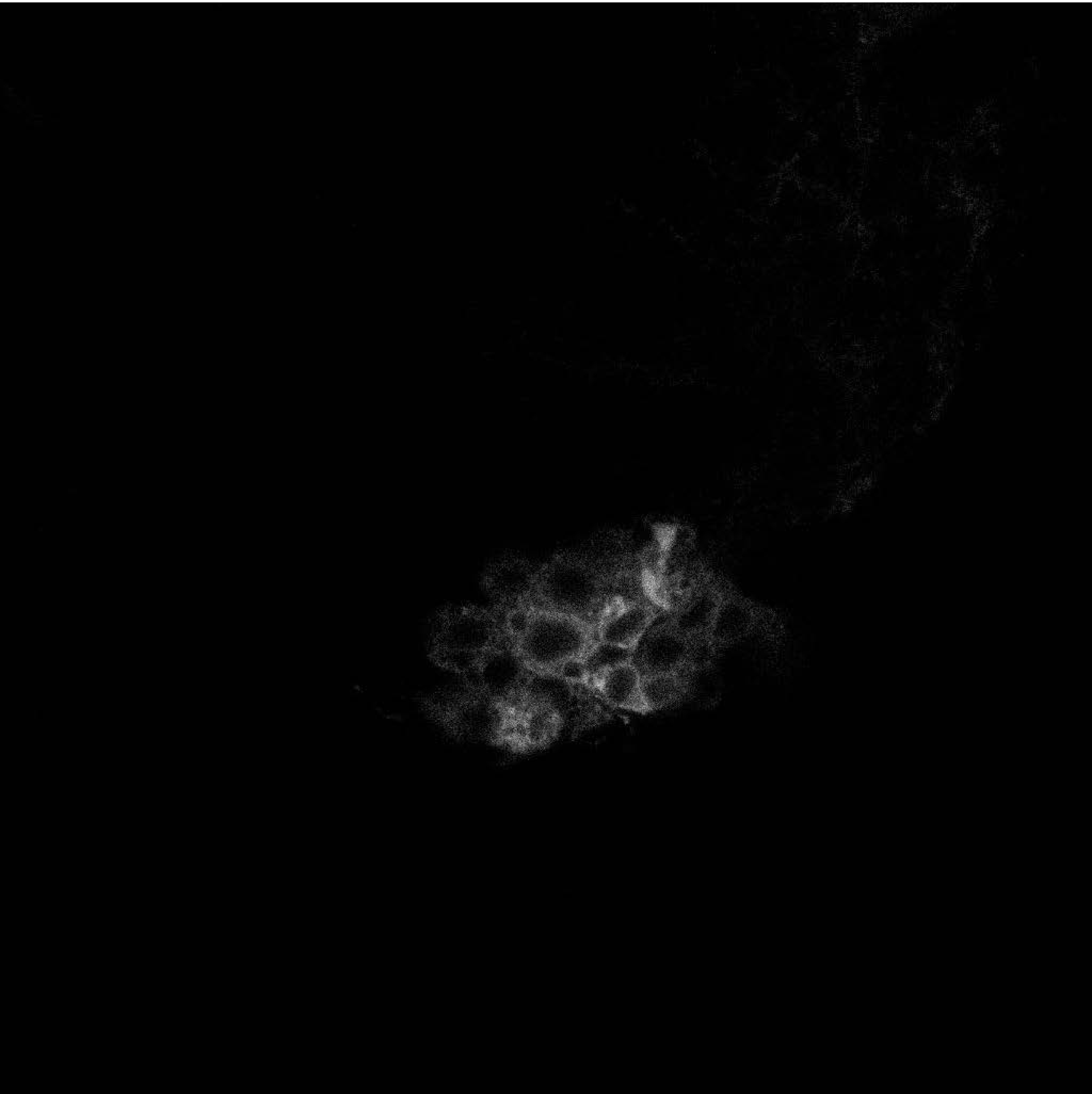

S6C_8B.tif

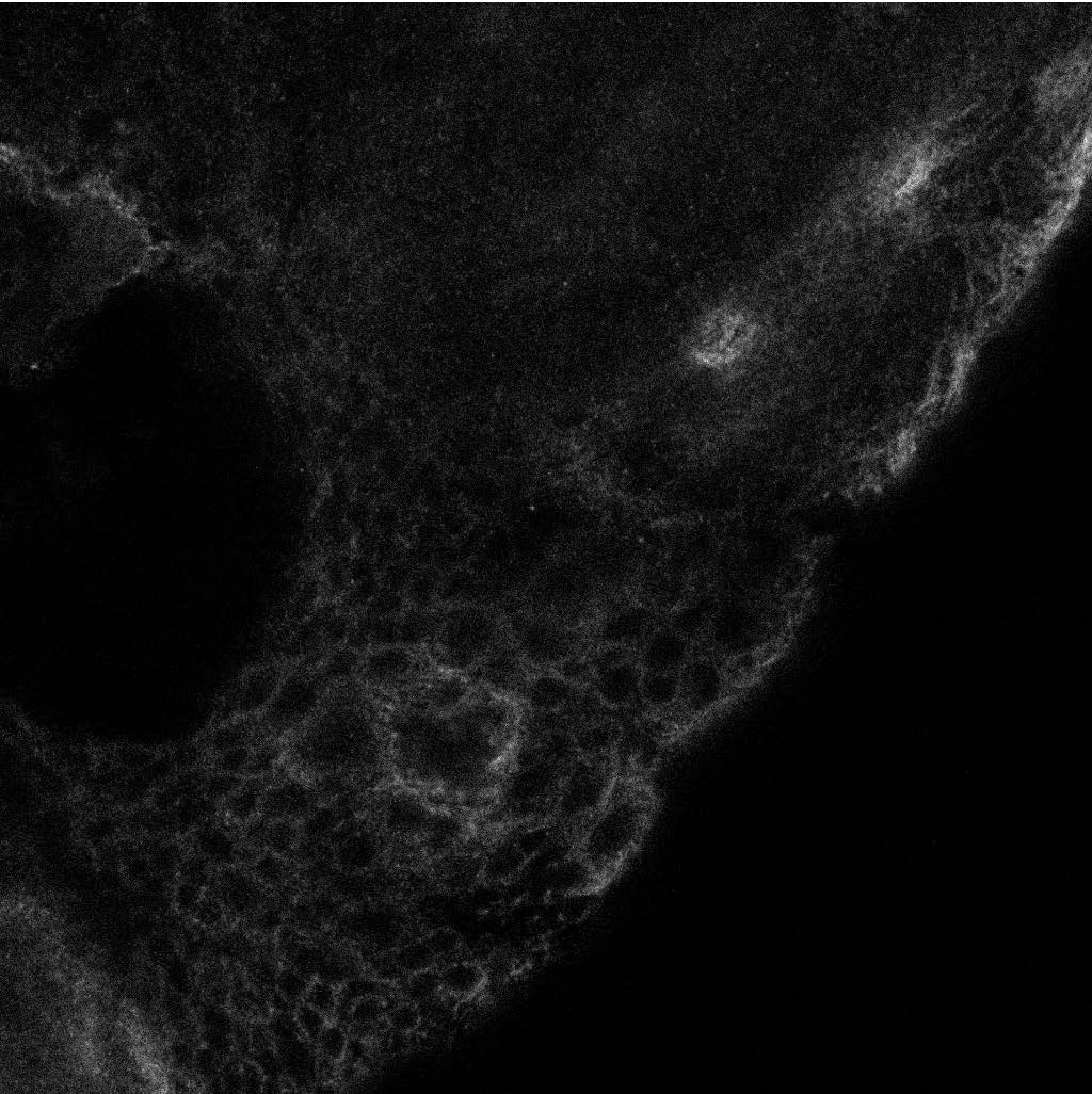

S6D_1.tif

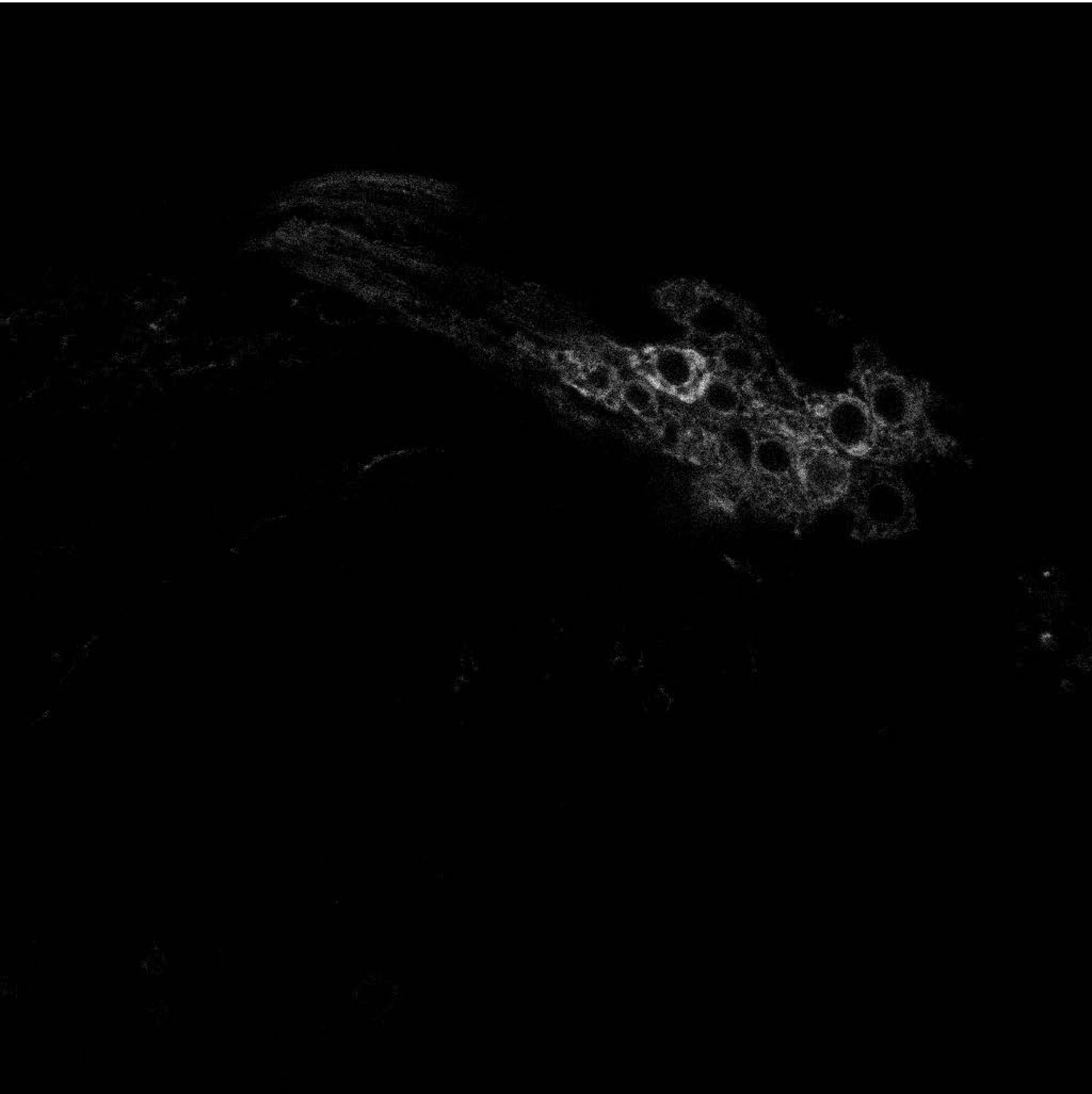

S6D_4.tif

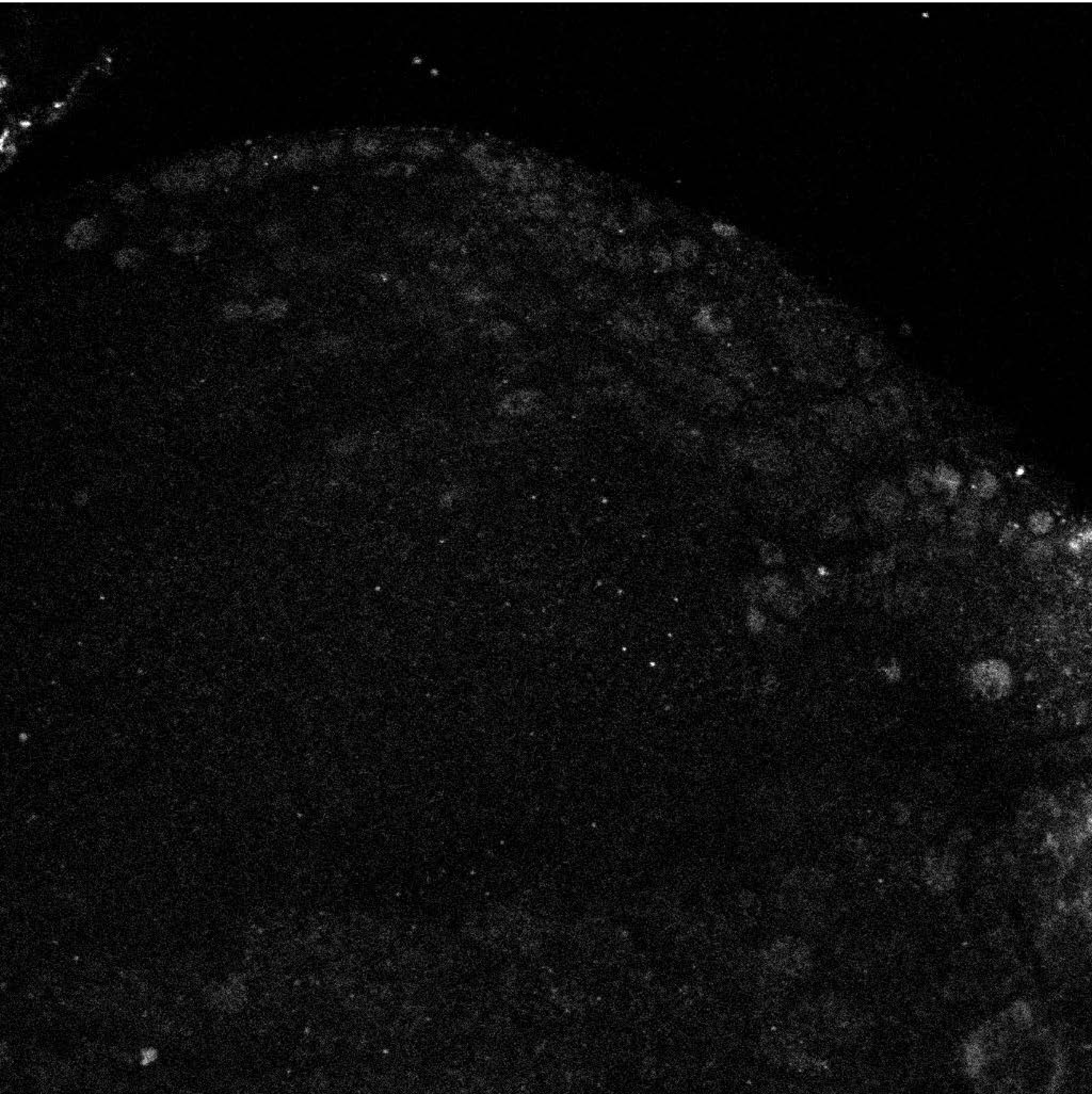

S6F_1.tif

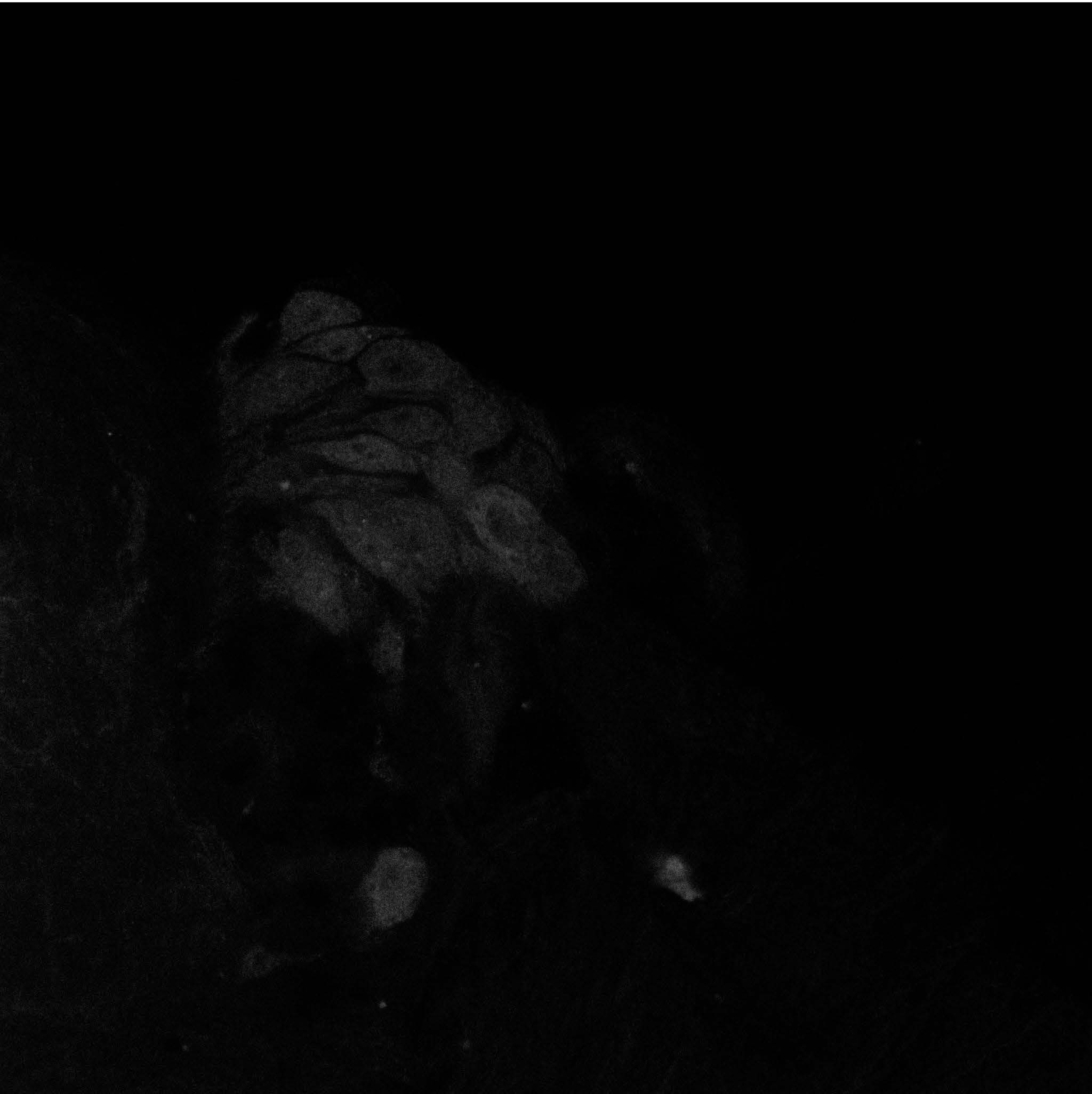

S6F_2.tif

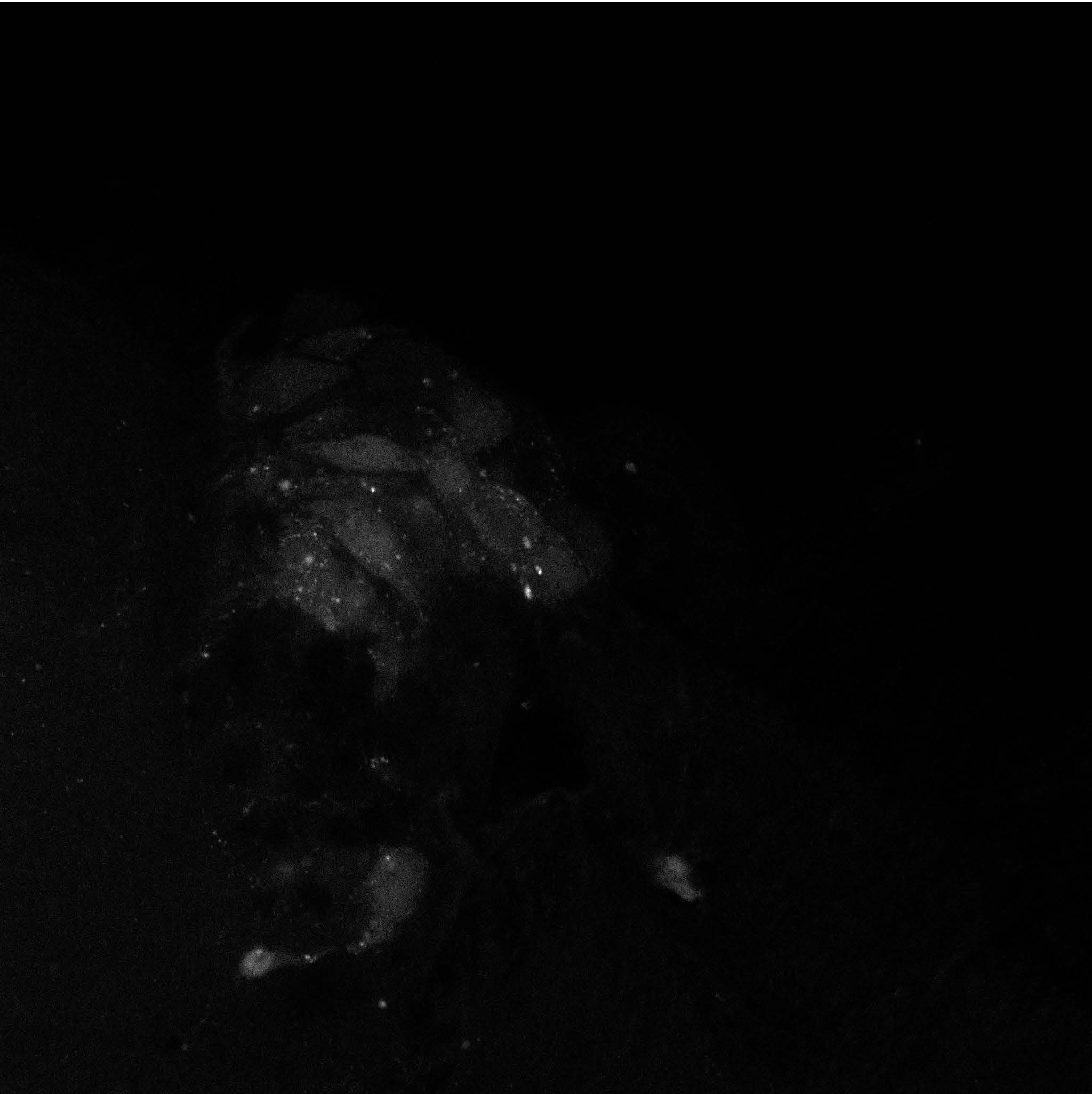

S6F_4.tif

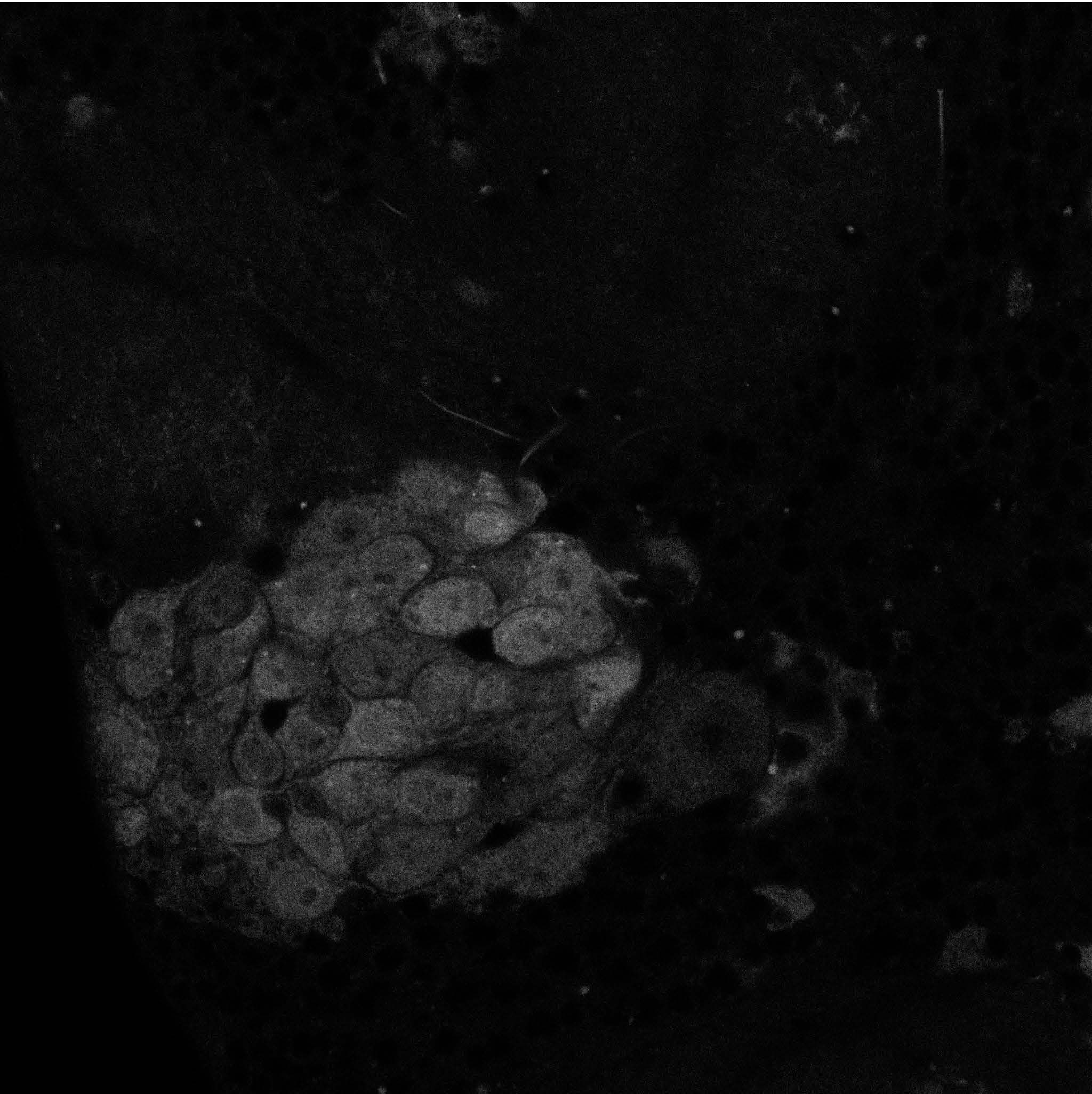

S6F_5.tif

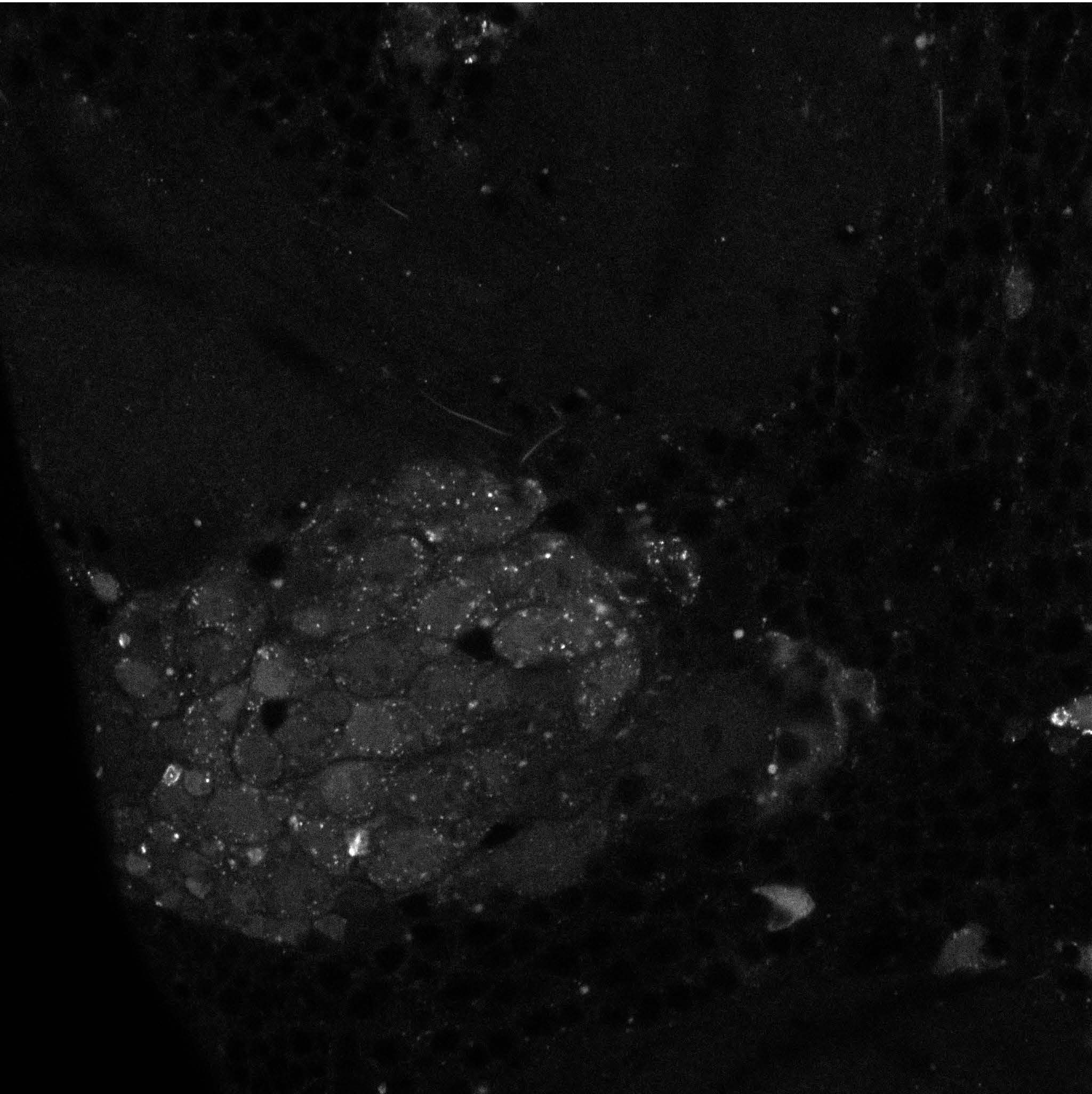

S7A_1A.tif

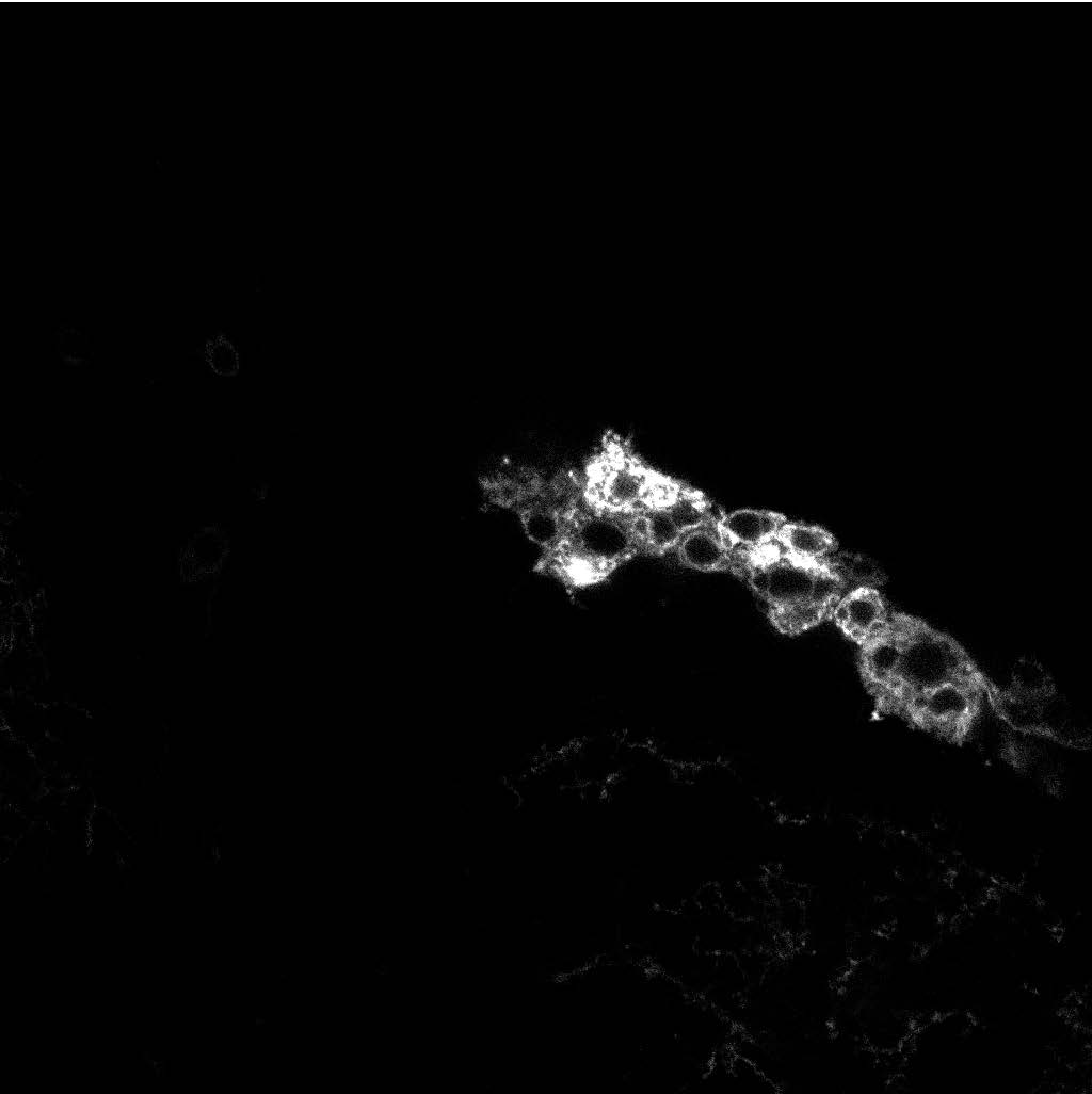

S7A_1B.tif

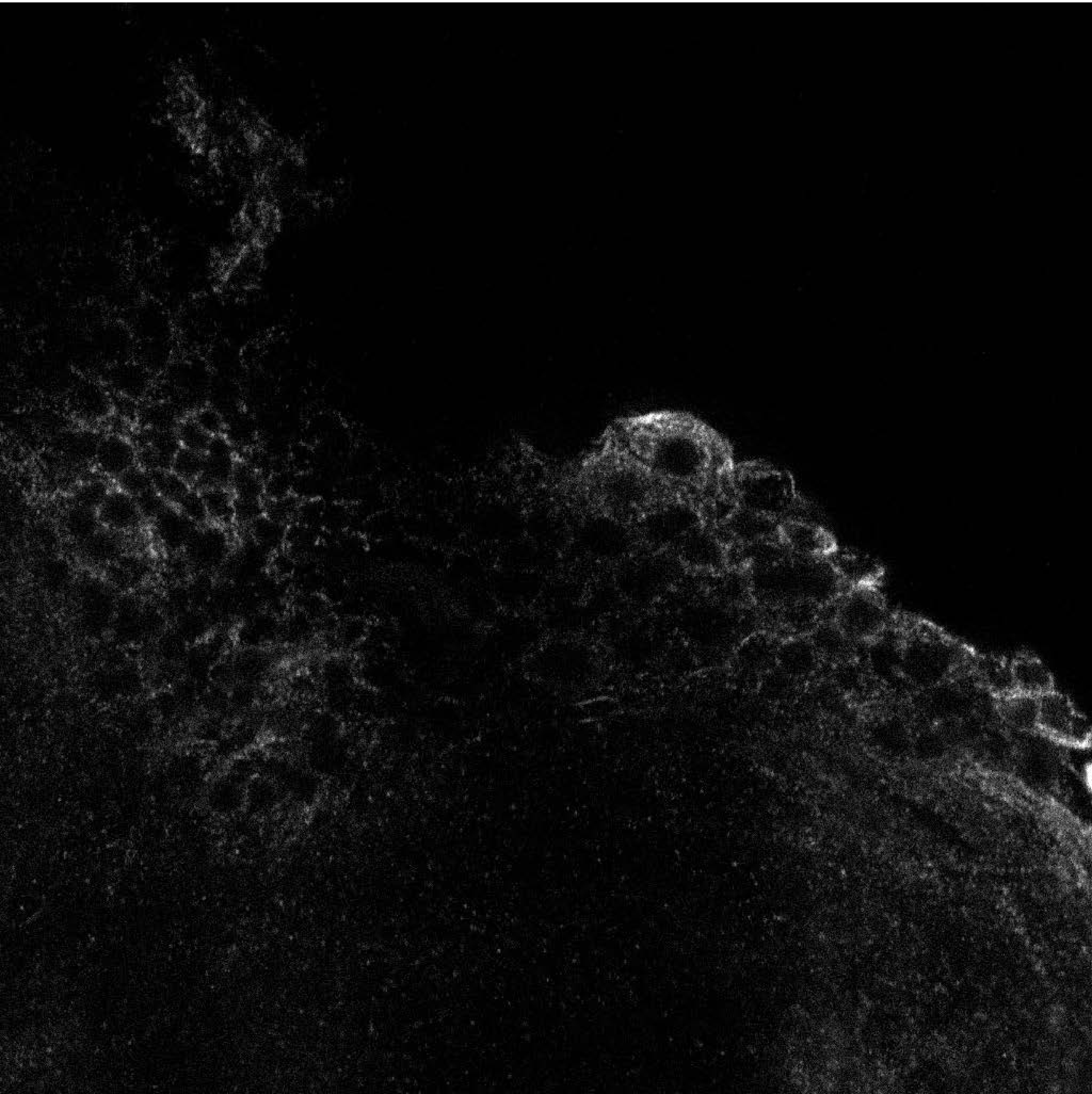

S7A_2A.tif

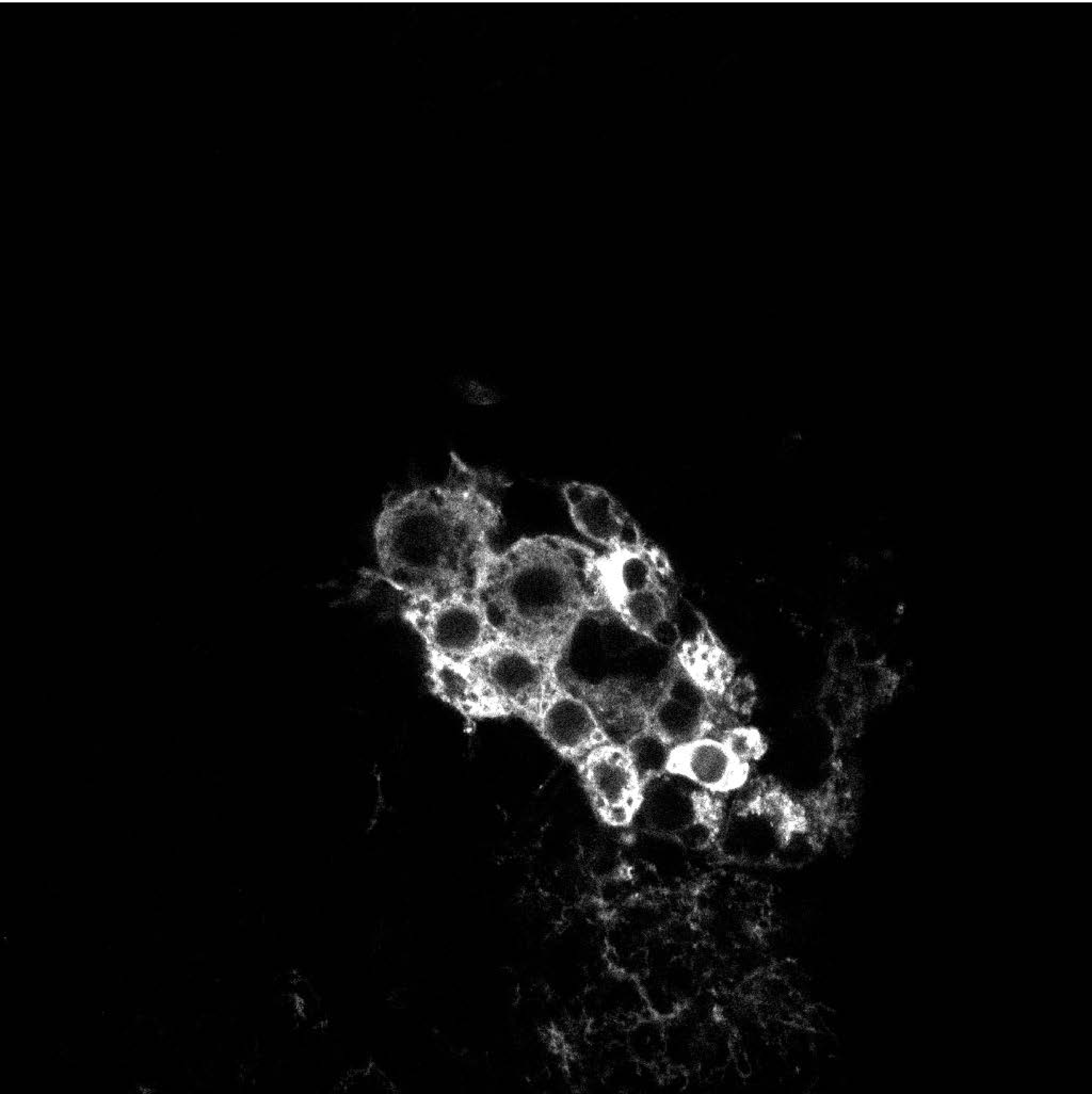

S7A_2B.tif

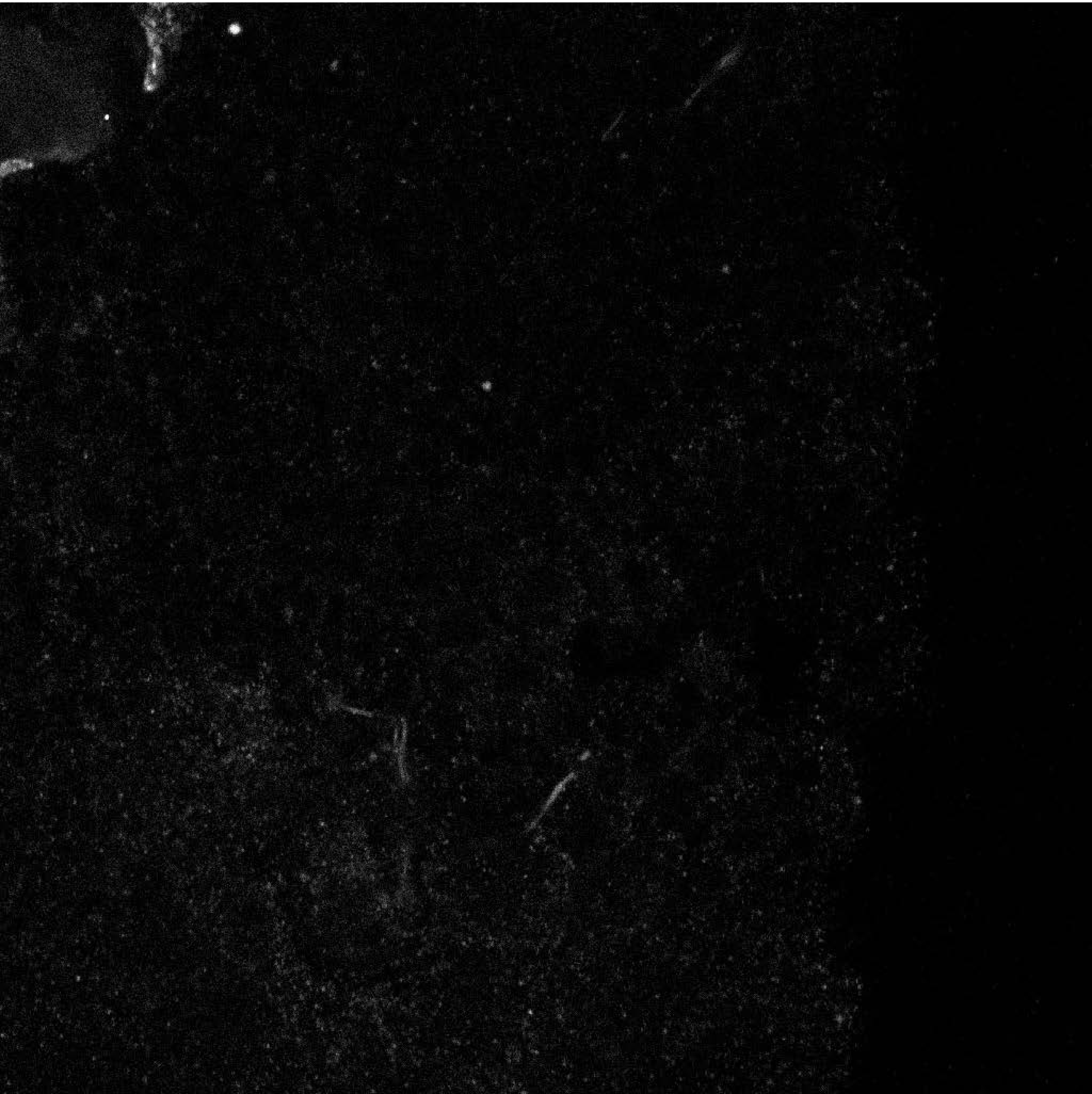

S7A_3A.tif

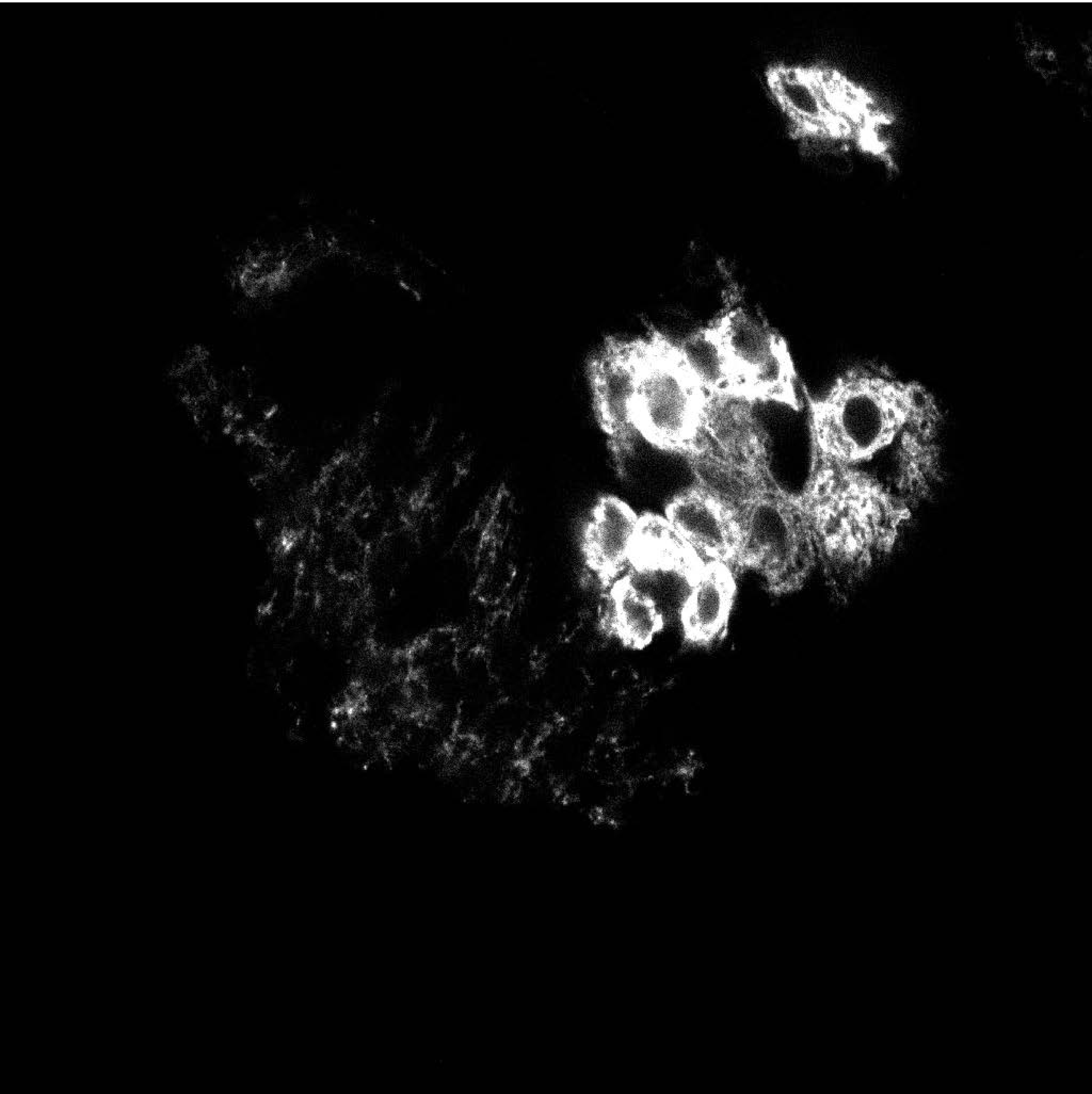

S7A_3B.tif

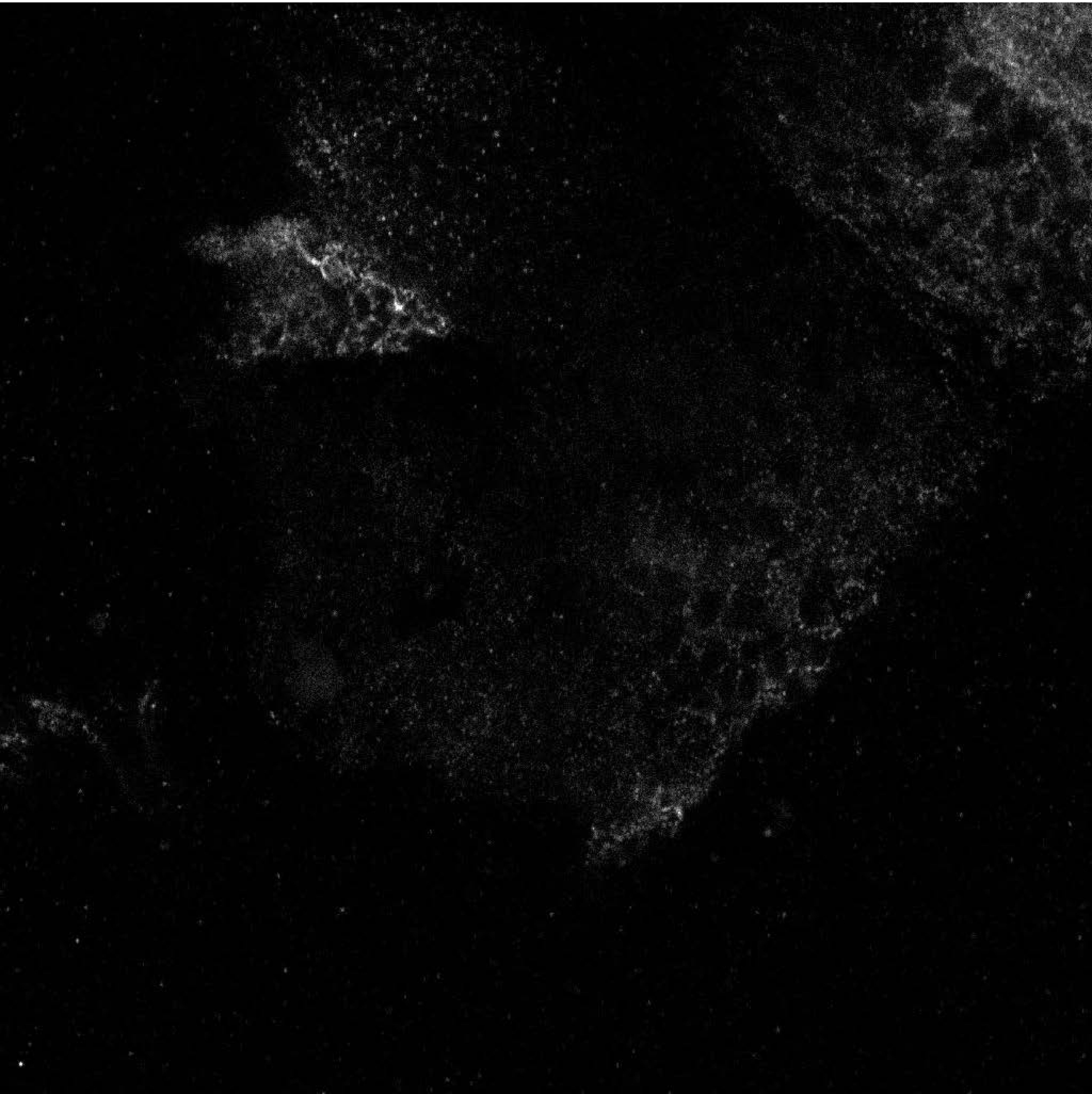

S7A_4A.tif

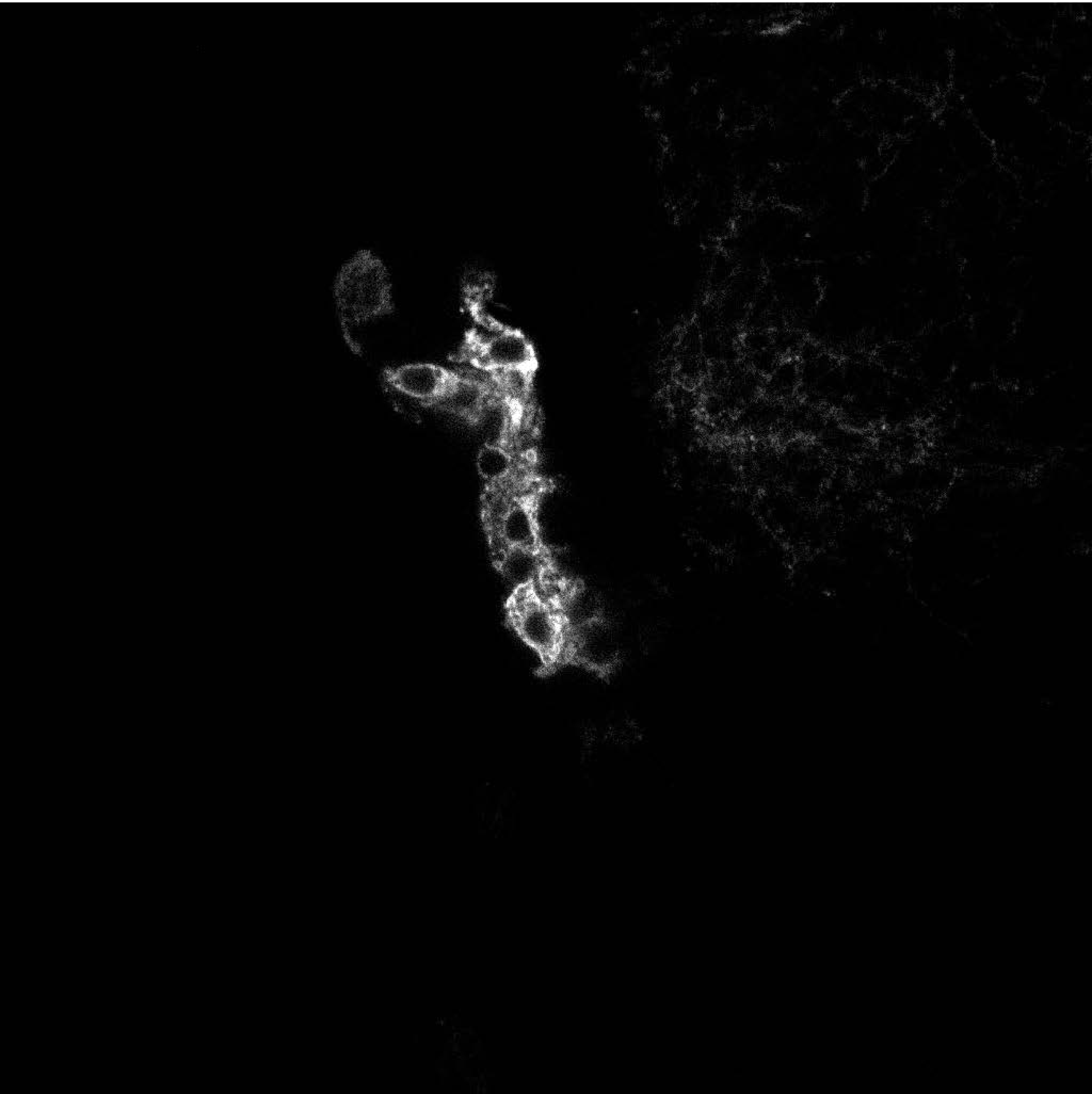

S7A_4B.tif

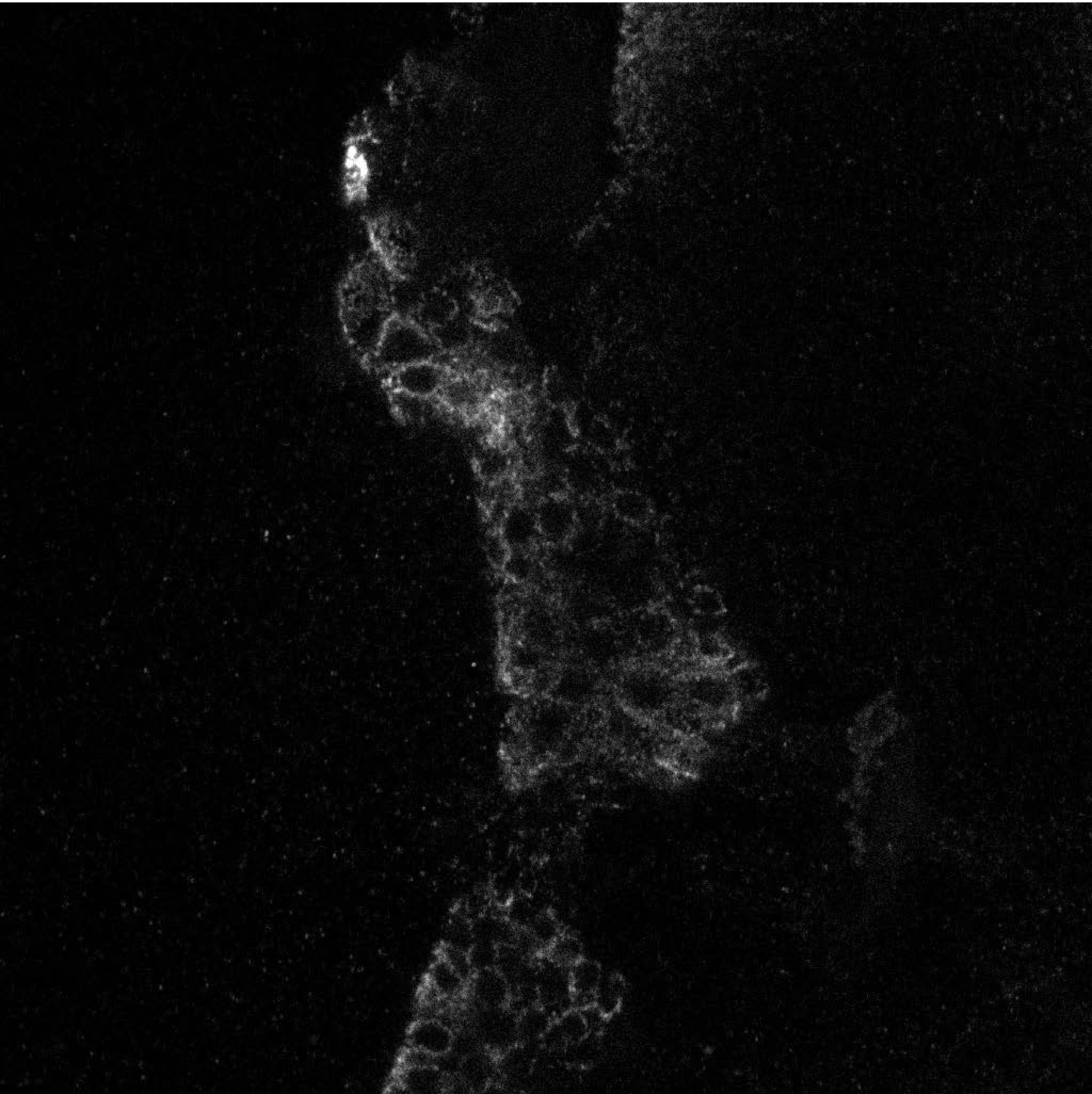

S7A_5A.tif

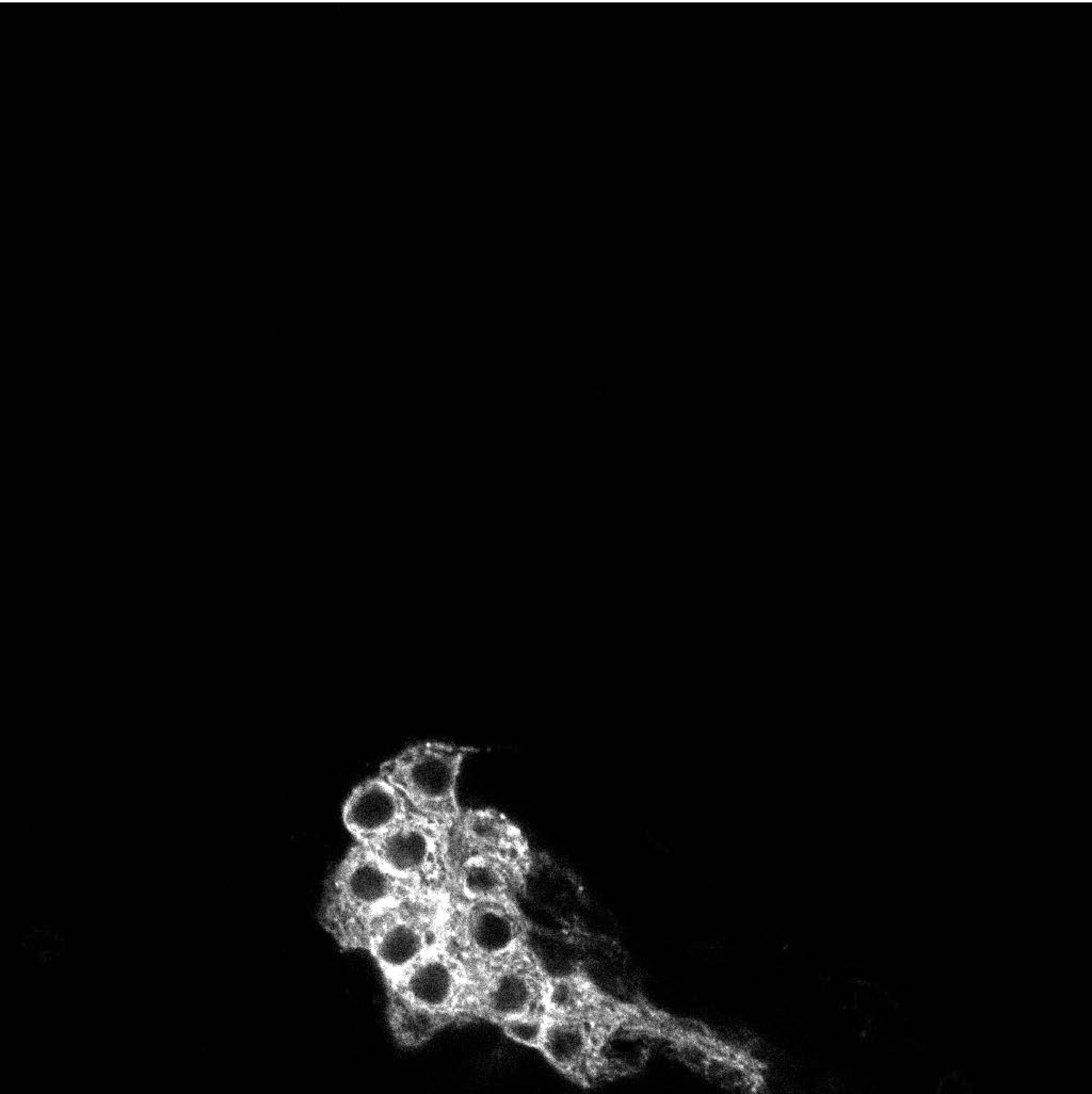

S7A_5B.tif

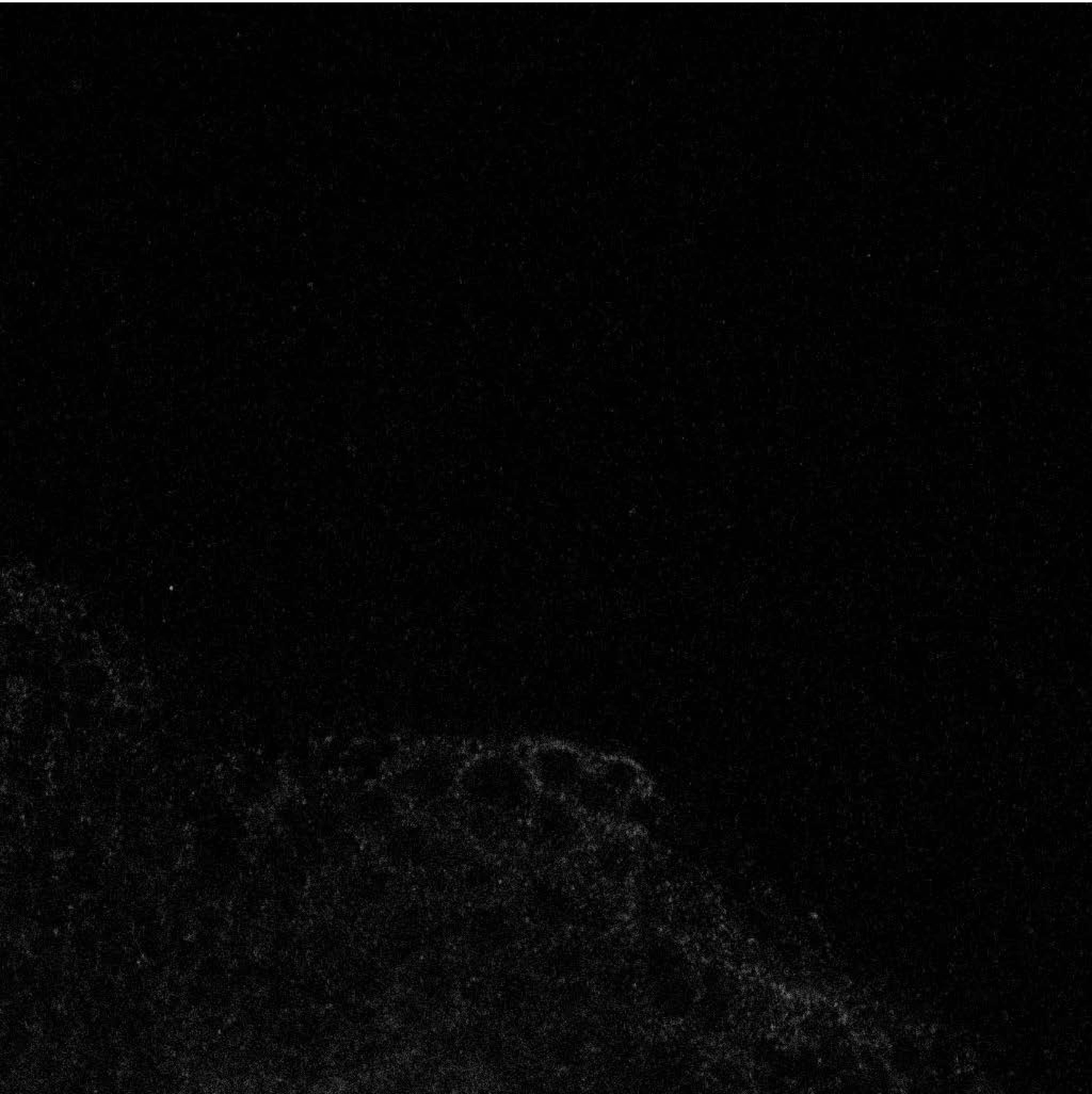

S7A_6A.tif

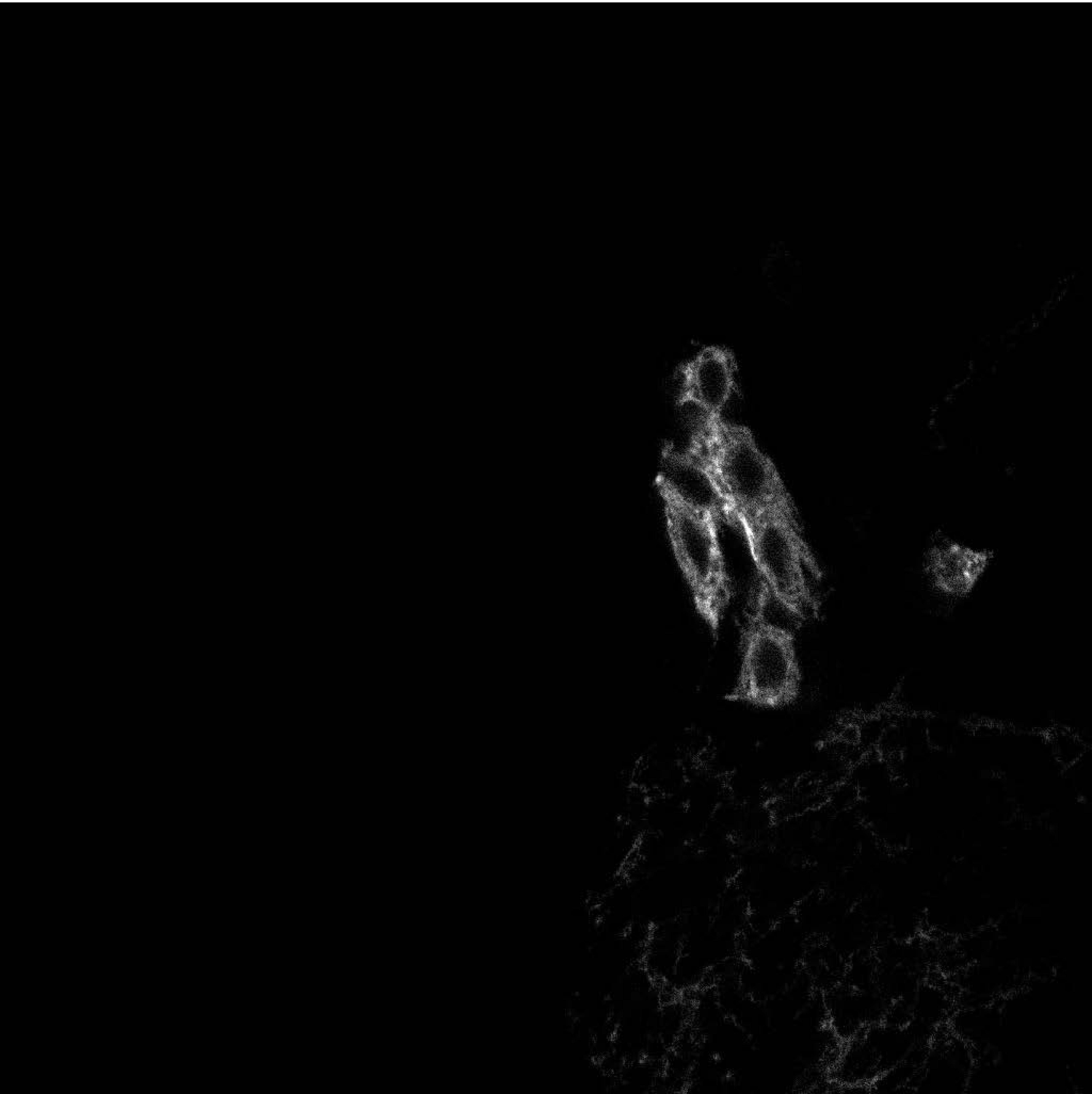

S7A_6B.tif

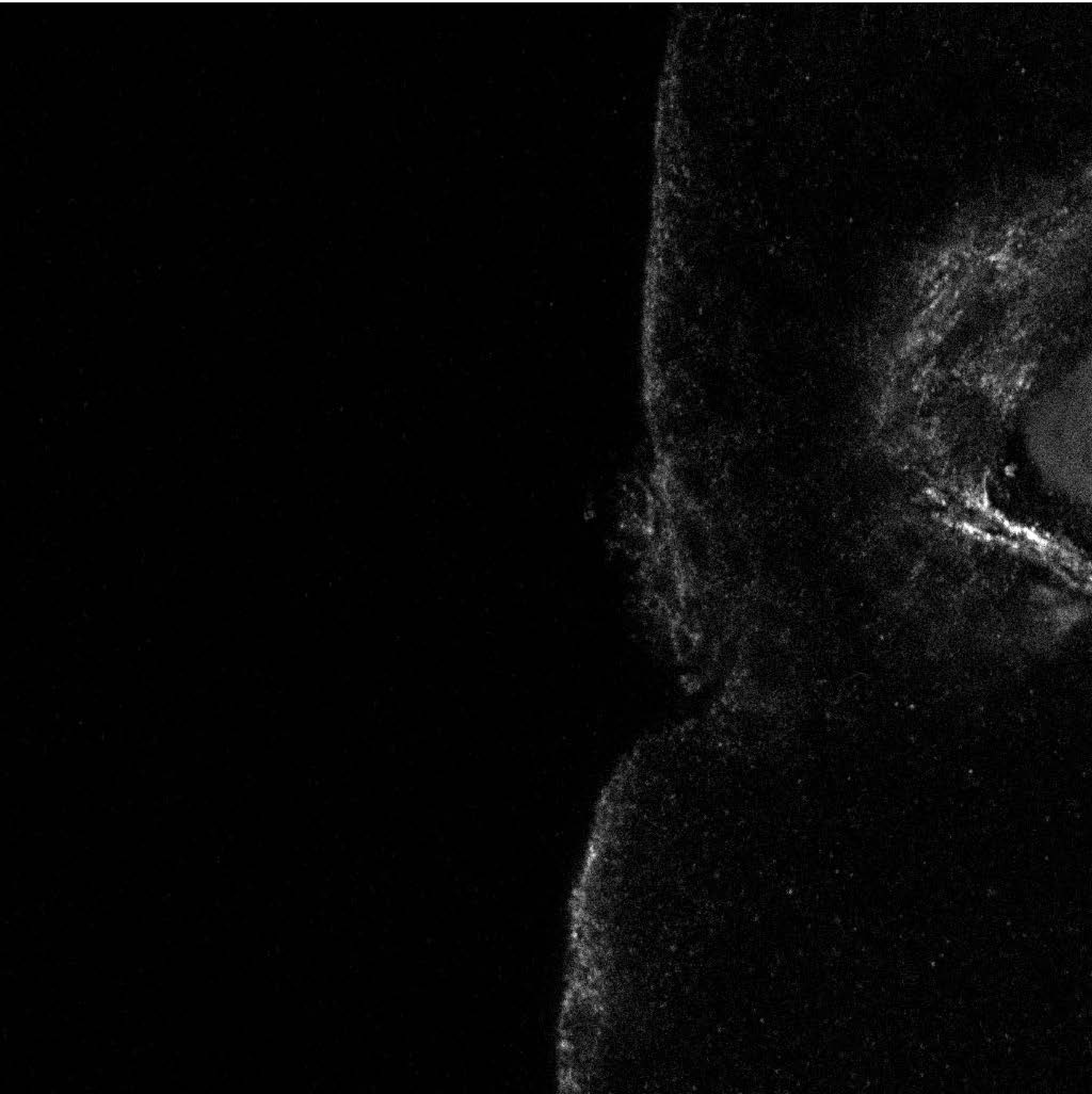

S7C_1A.tif

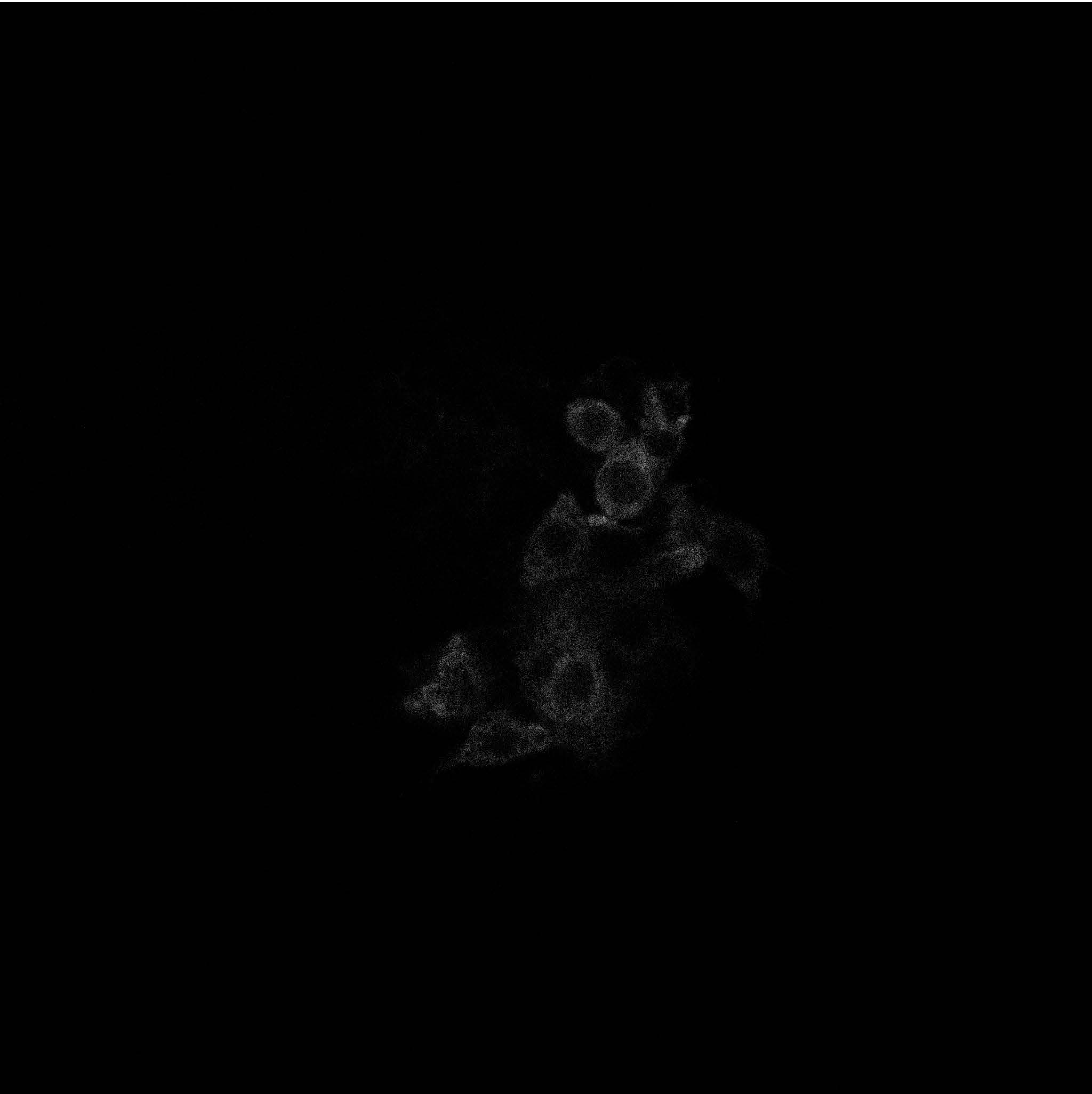

S7C_1B.tif

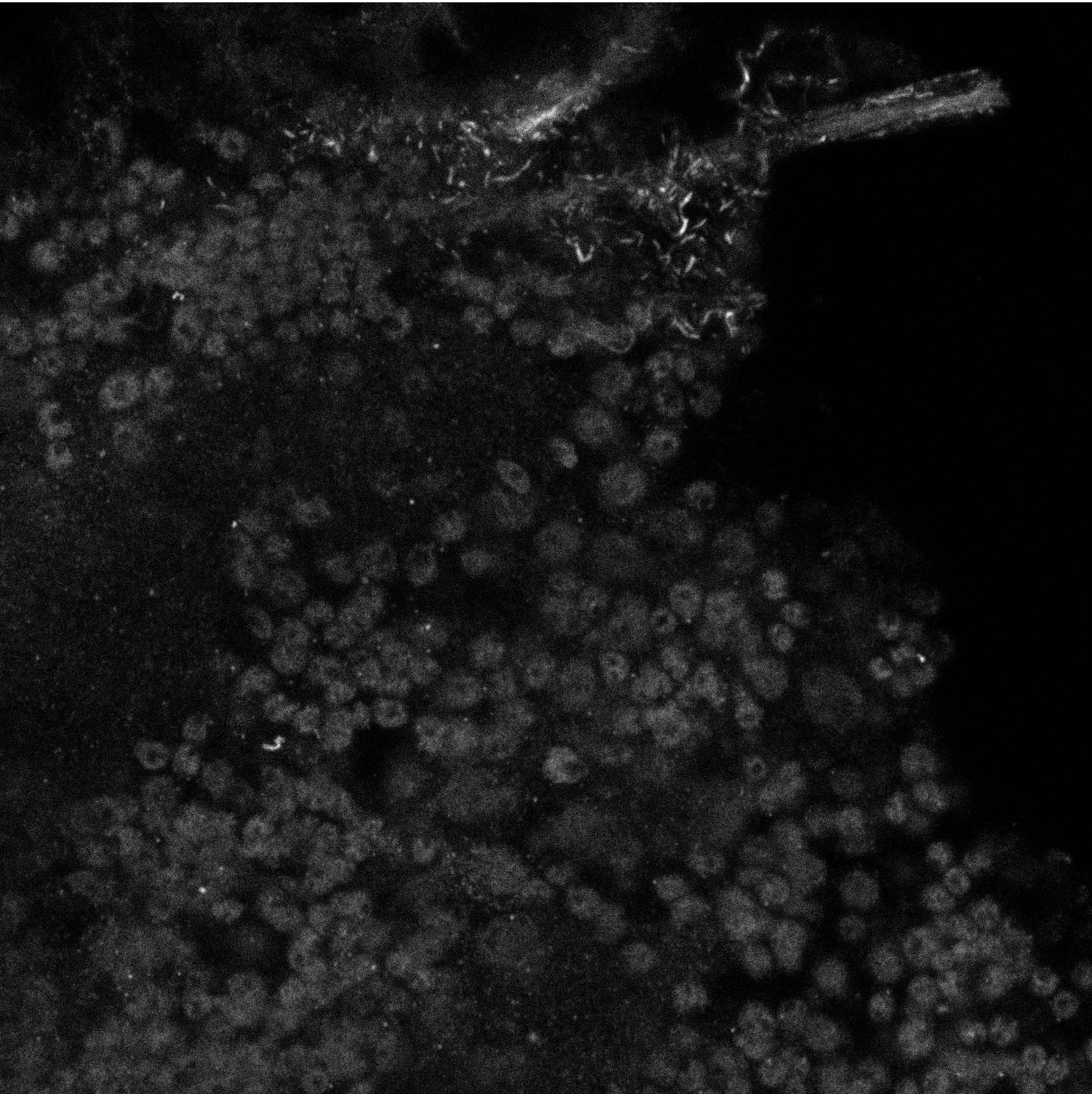

S7C_2A.tif

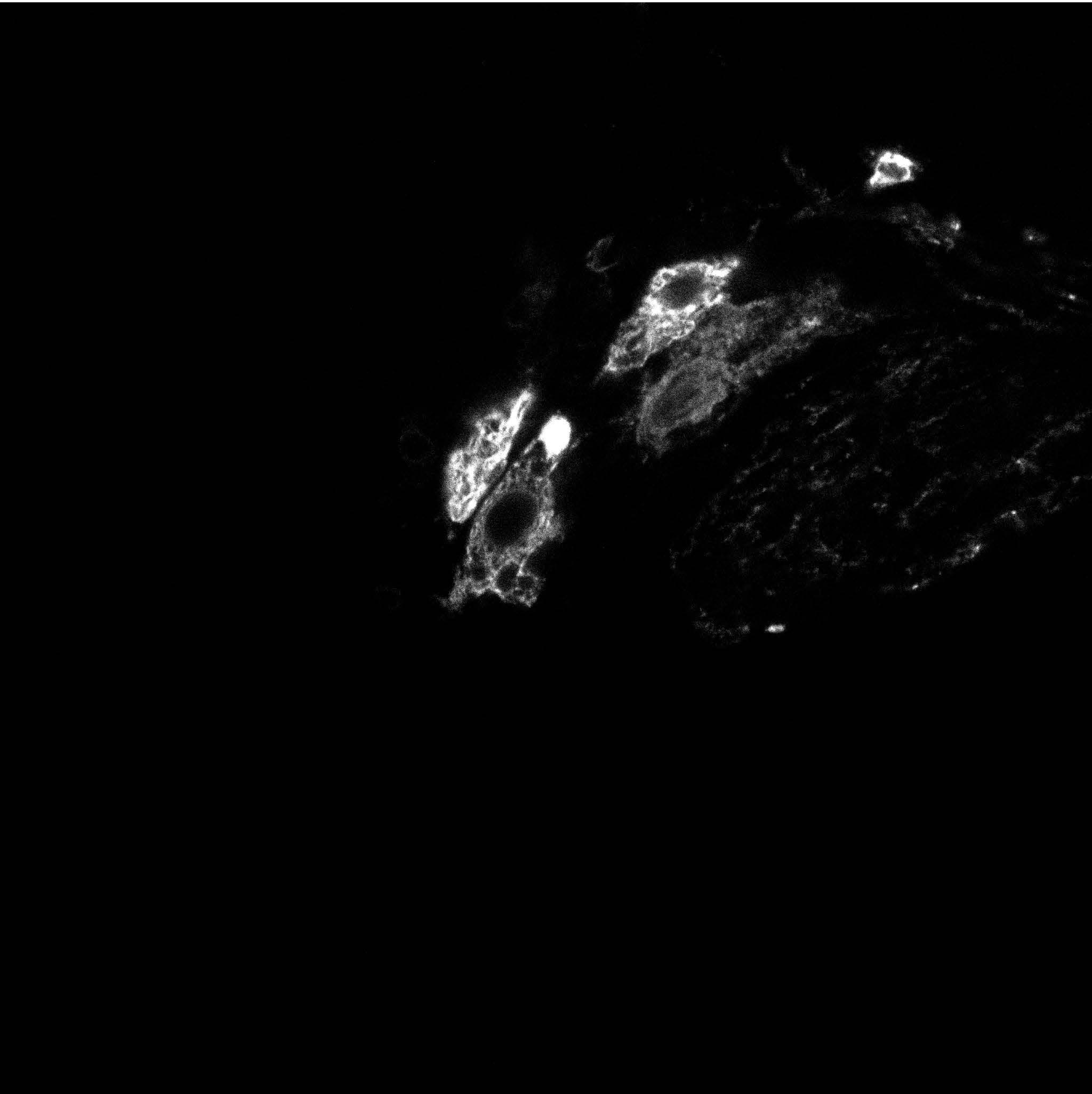

S7C_2B.tif

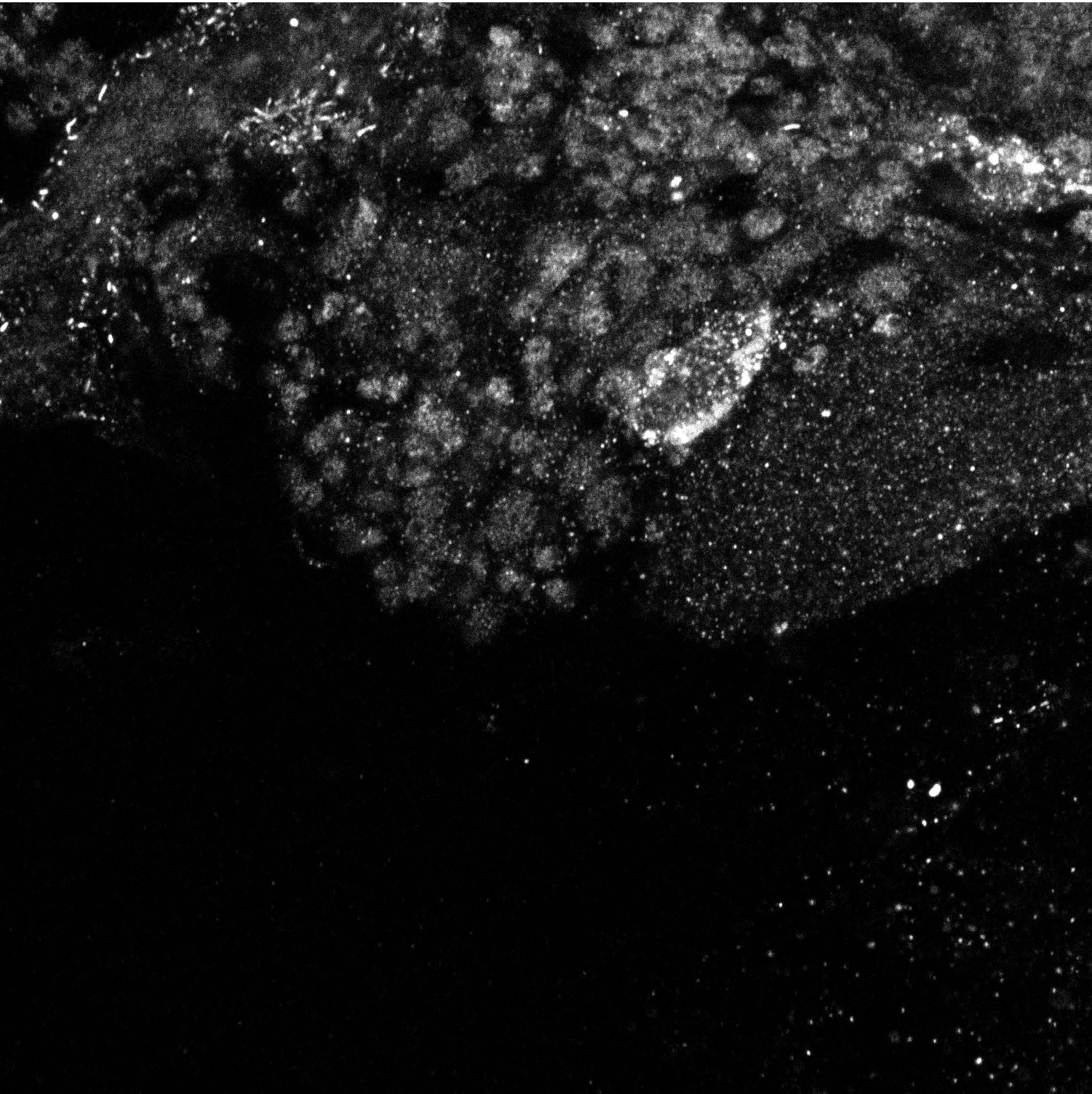

S7C_3A.tif

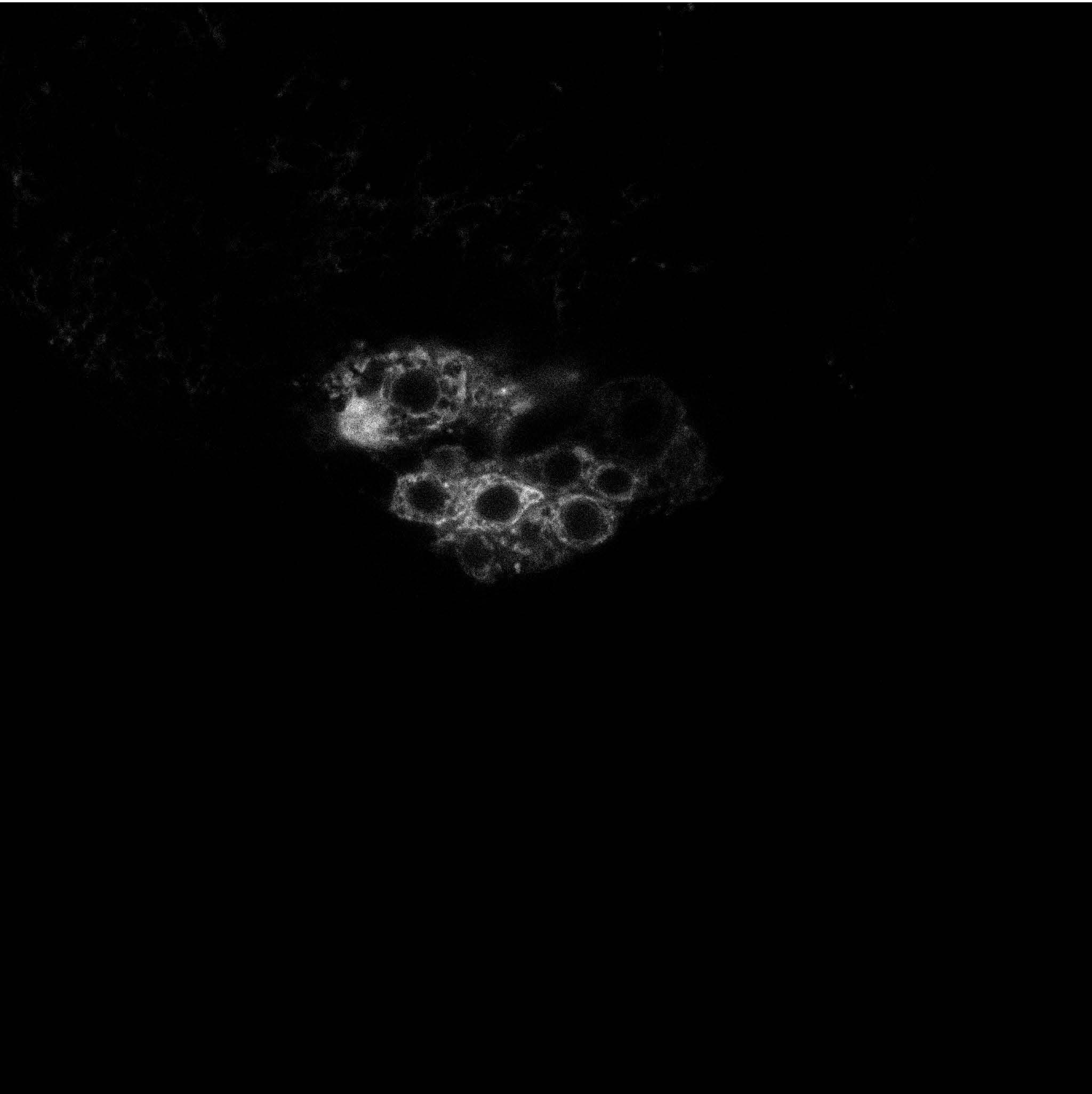

S7C_3B.tif

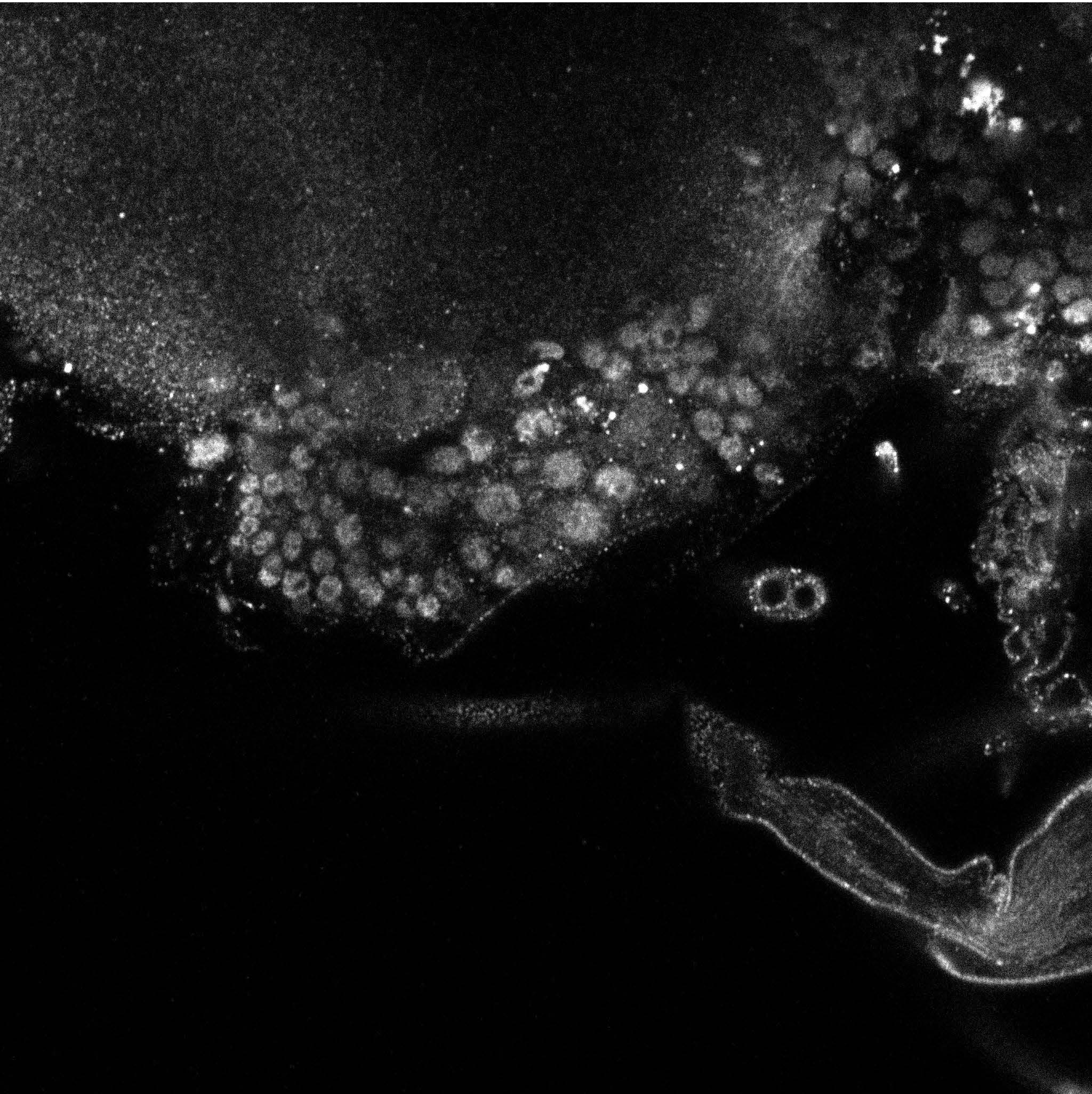

S7C_4A.tif

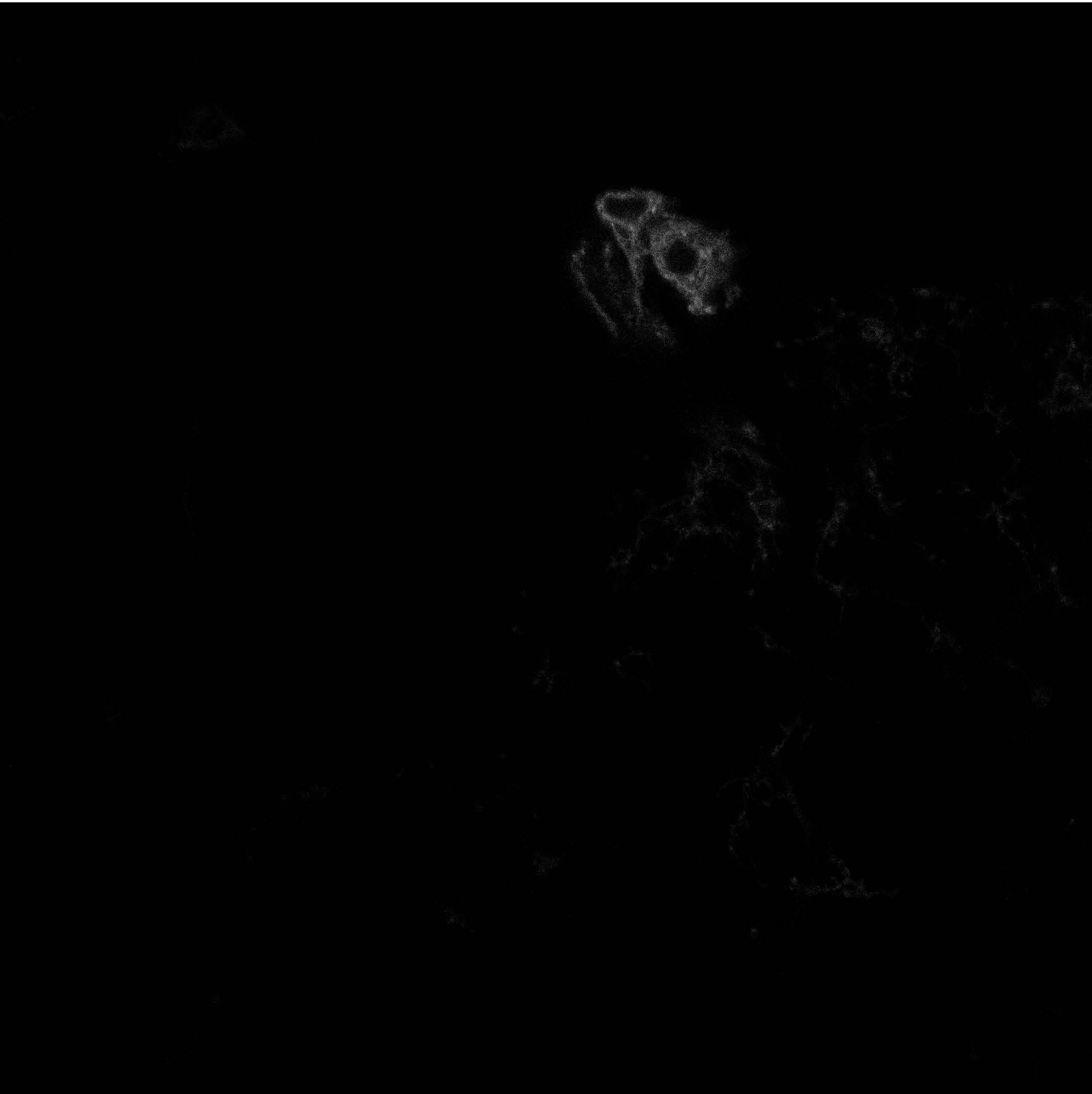

S7C_4B.tif

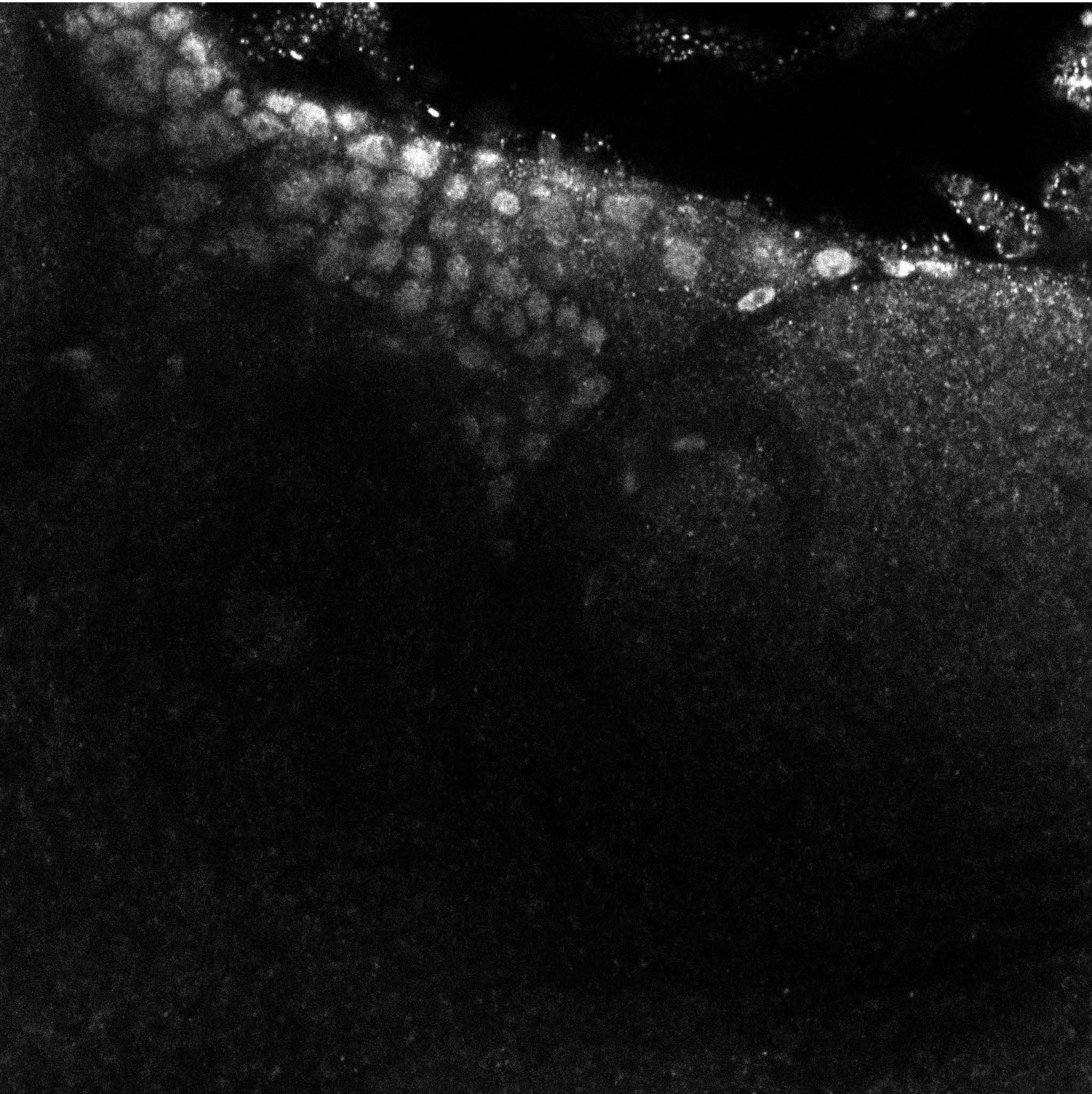

S7C_5A.tif

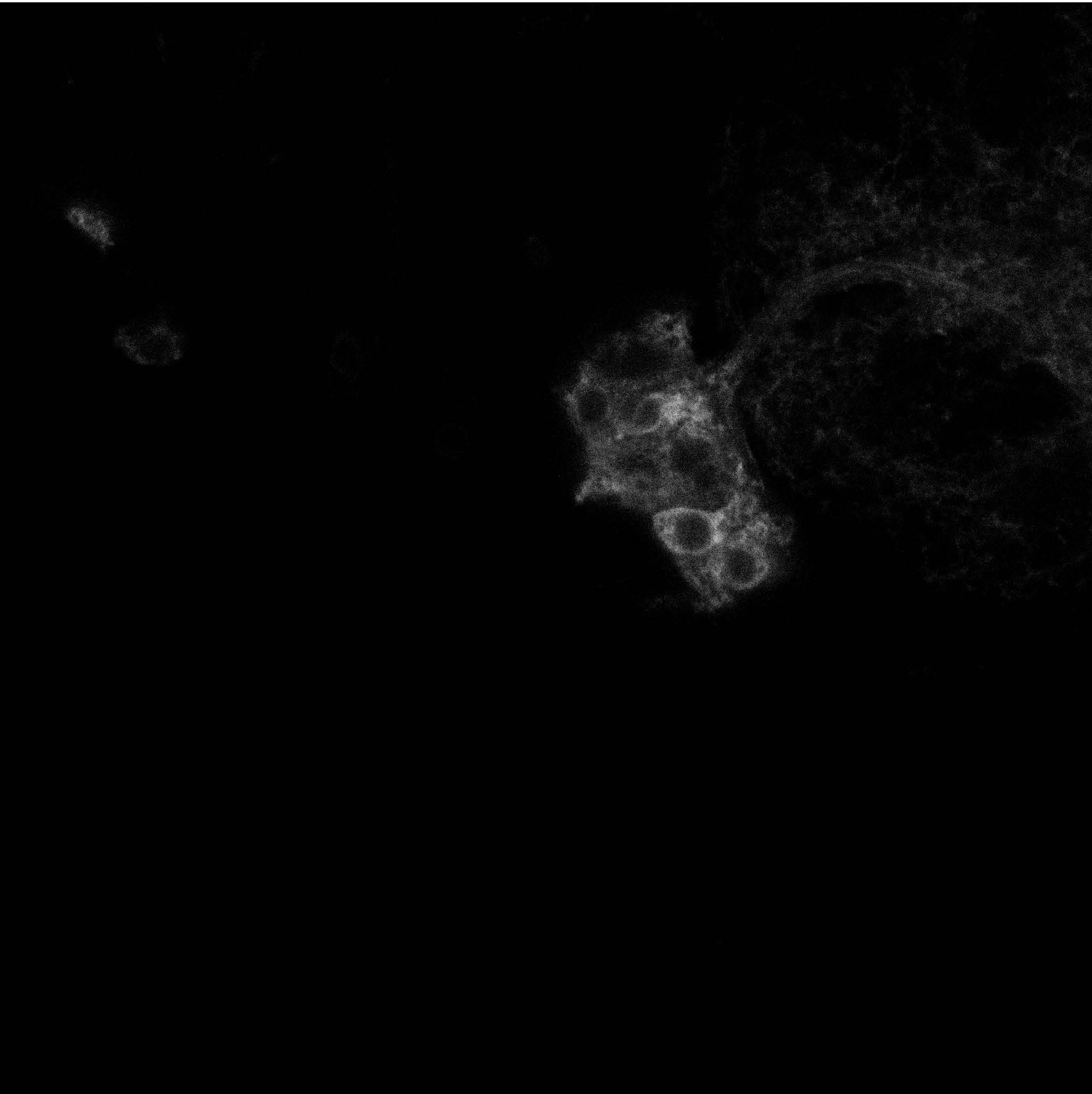

S7C_5B.tif

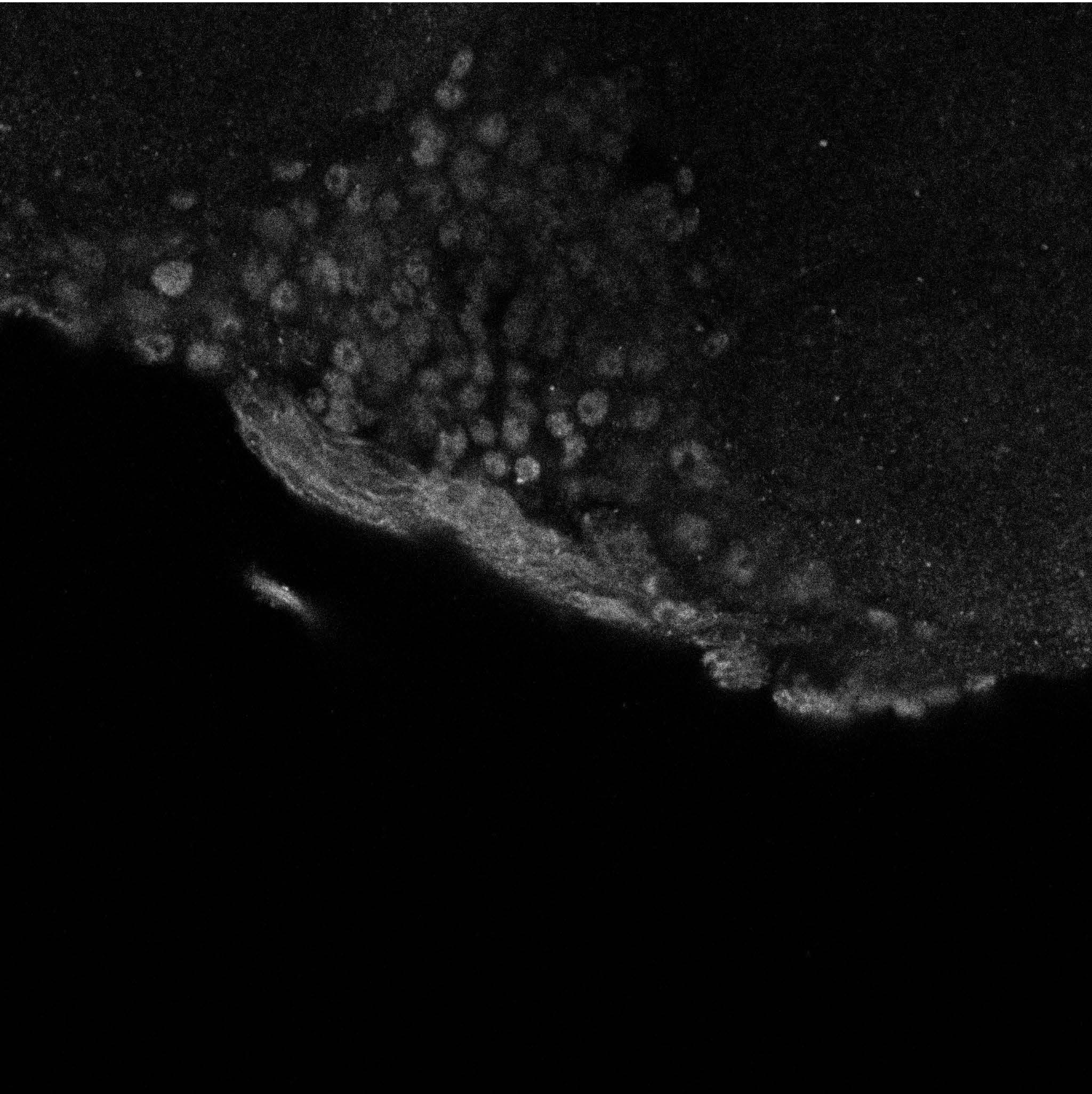

S7C_6A.tif

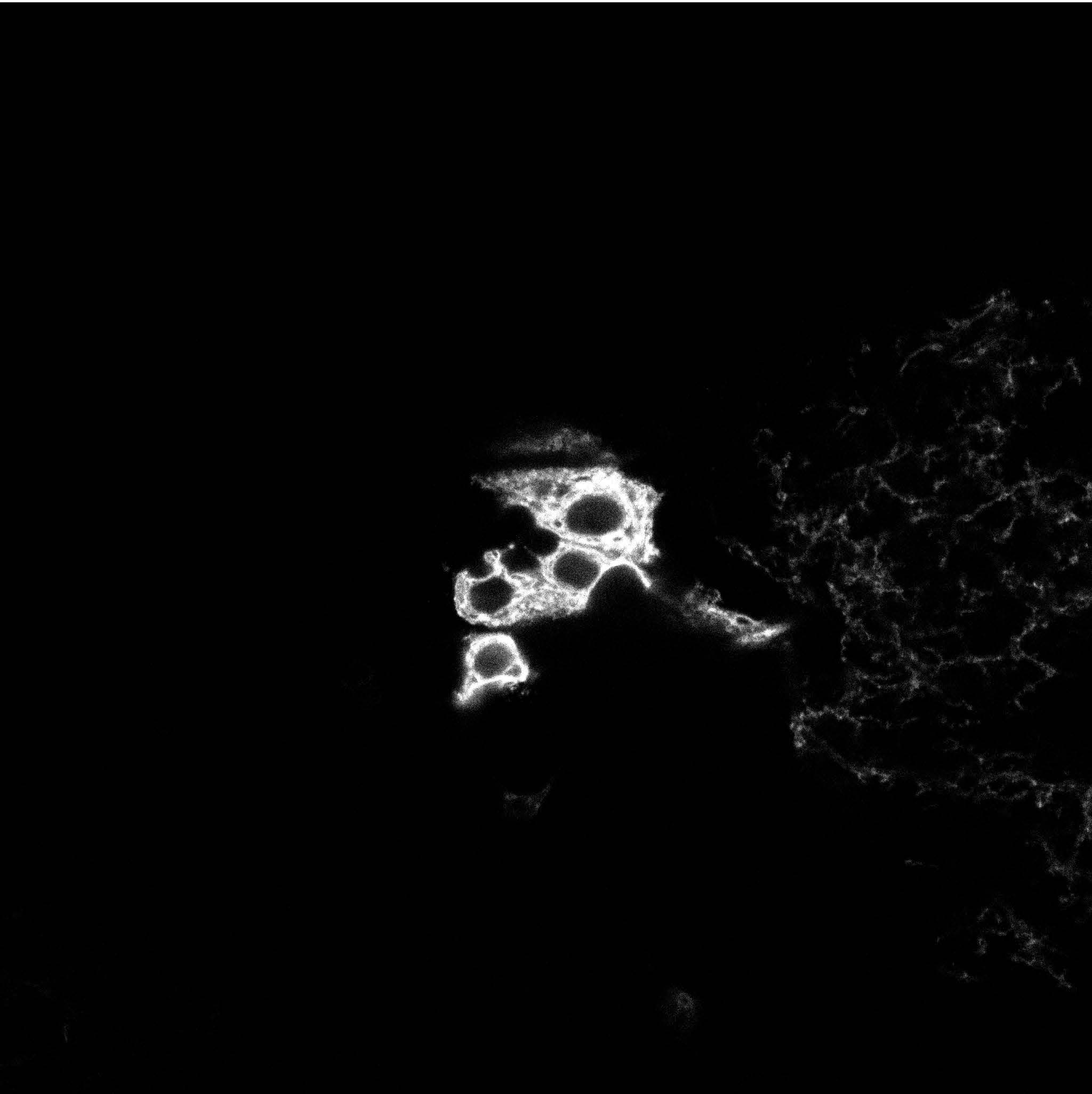

S7C_6B.tif

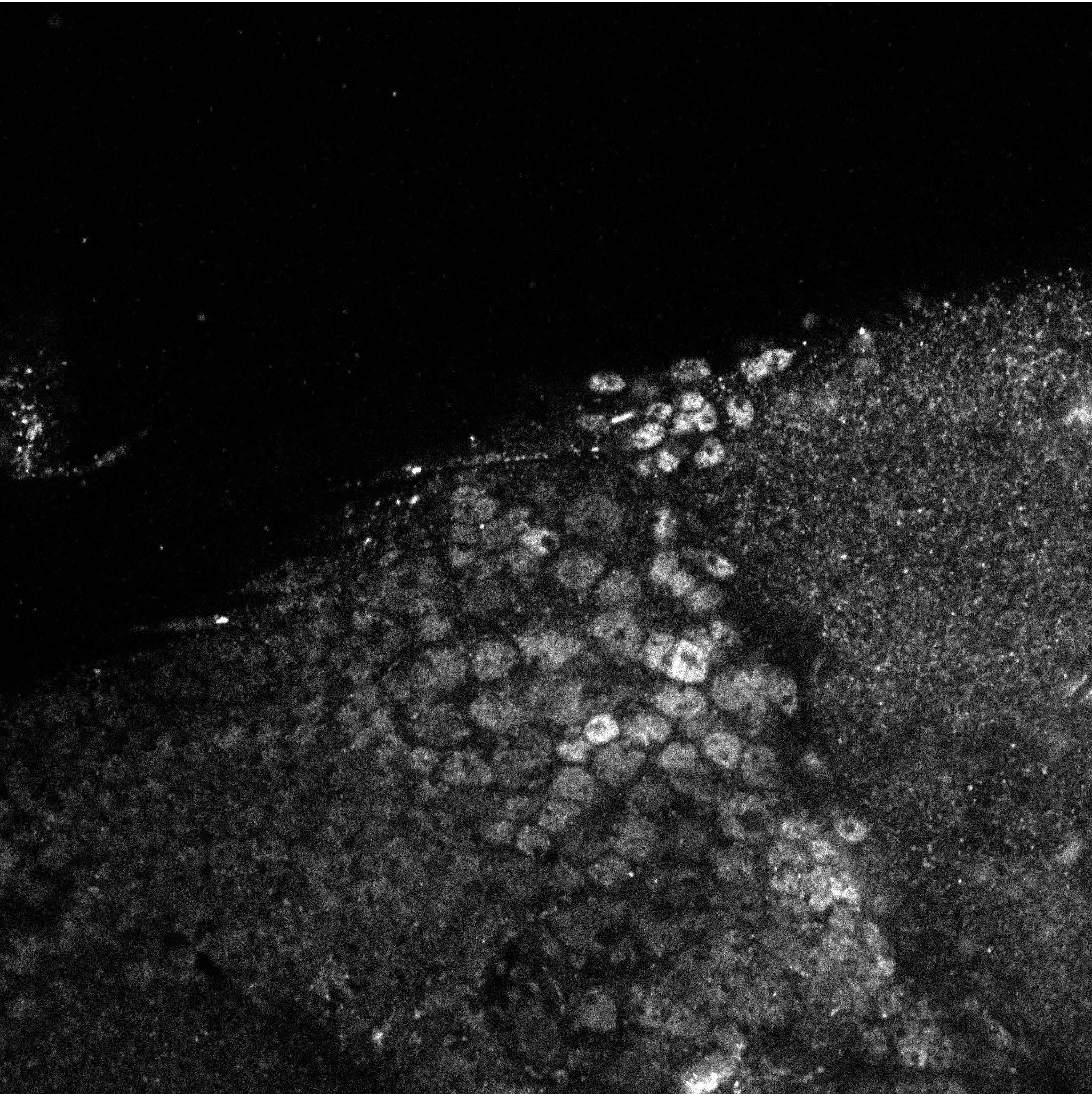

S7C_7A.tif

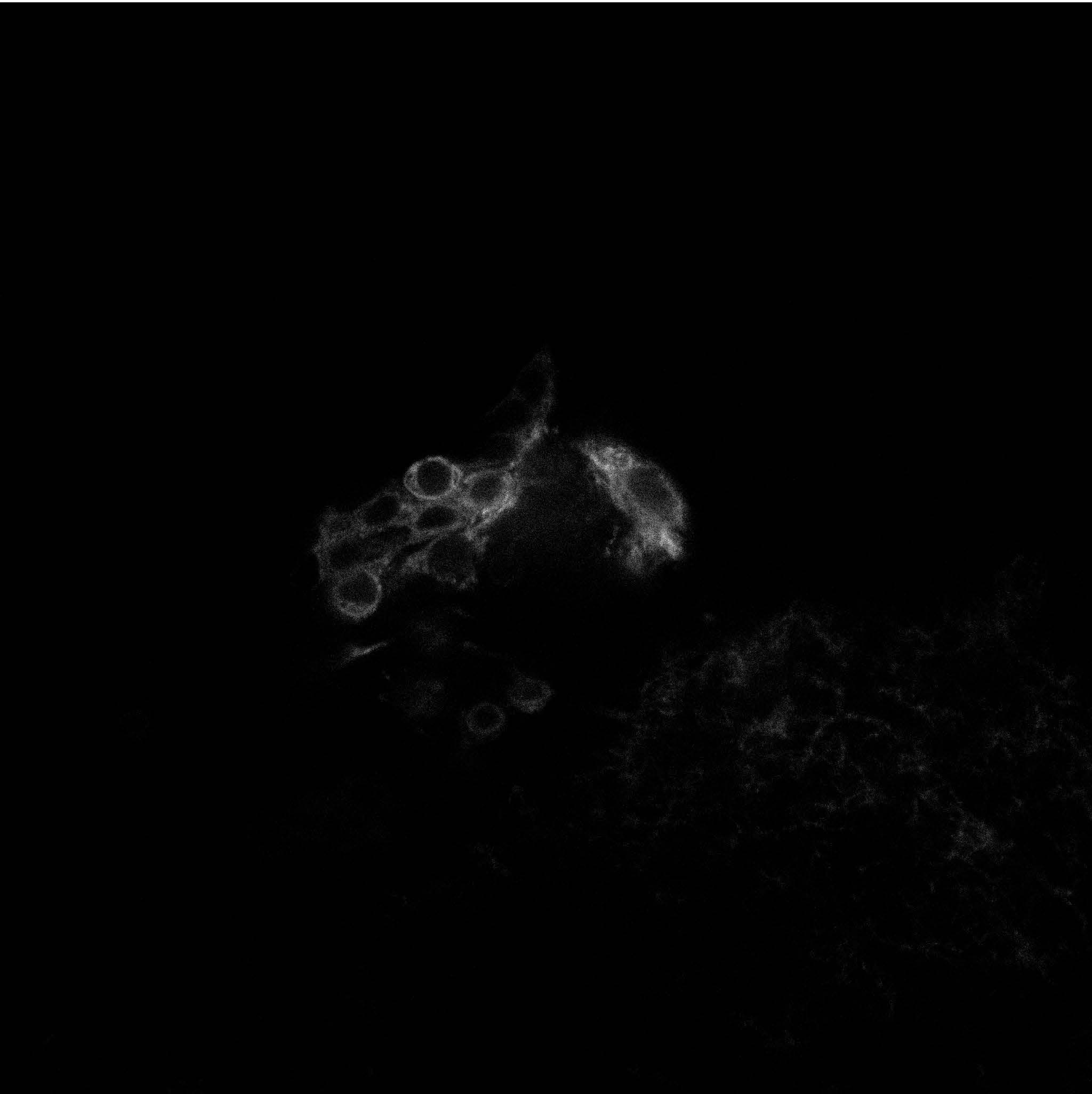

S7C_7B.tif

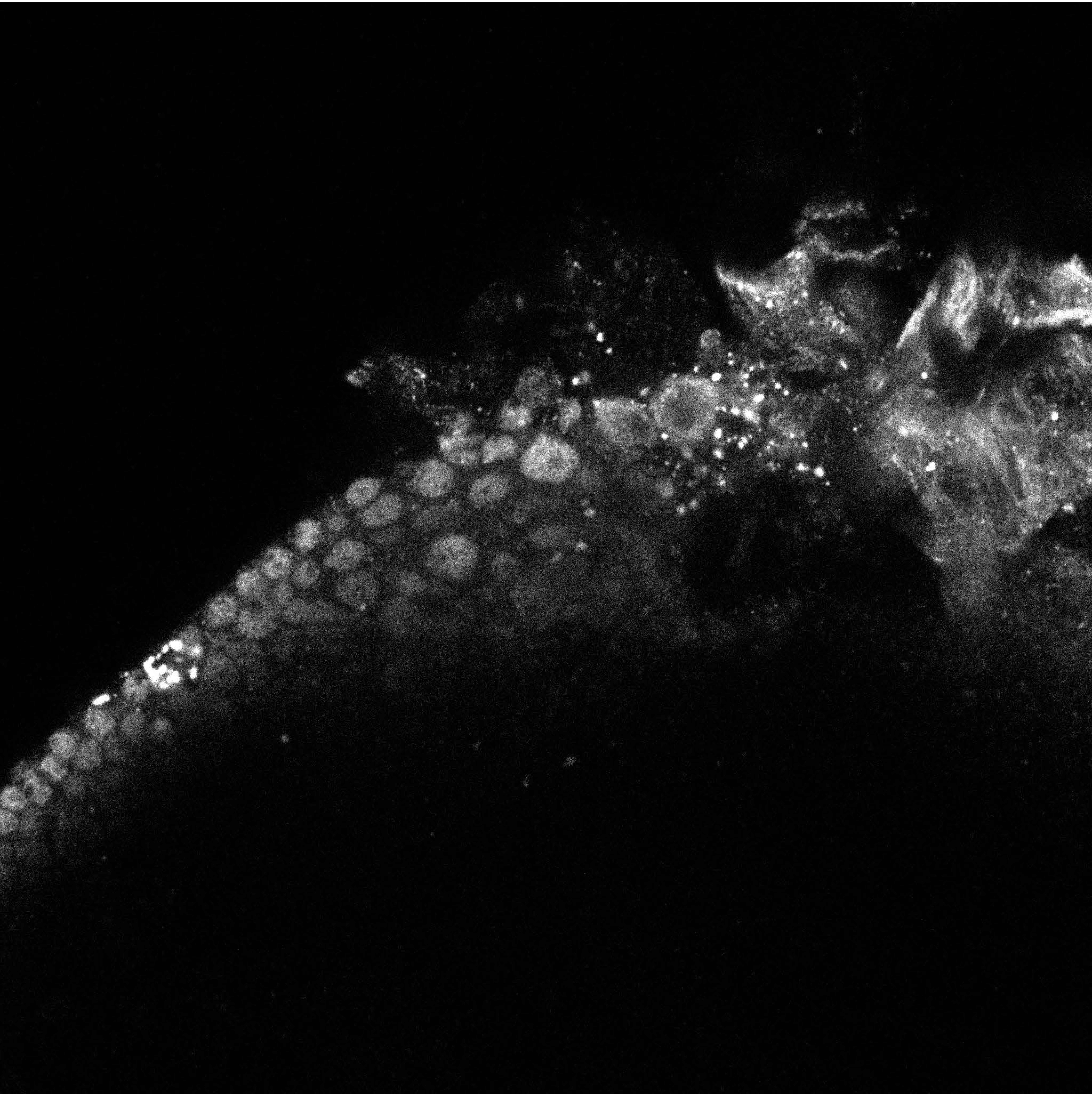

S7C_8A.tif

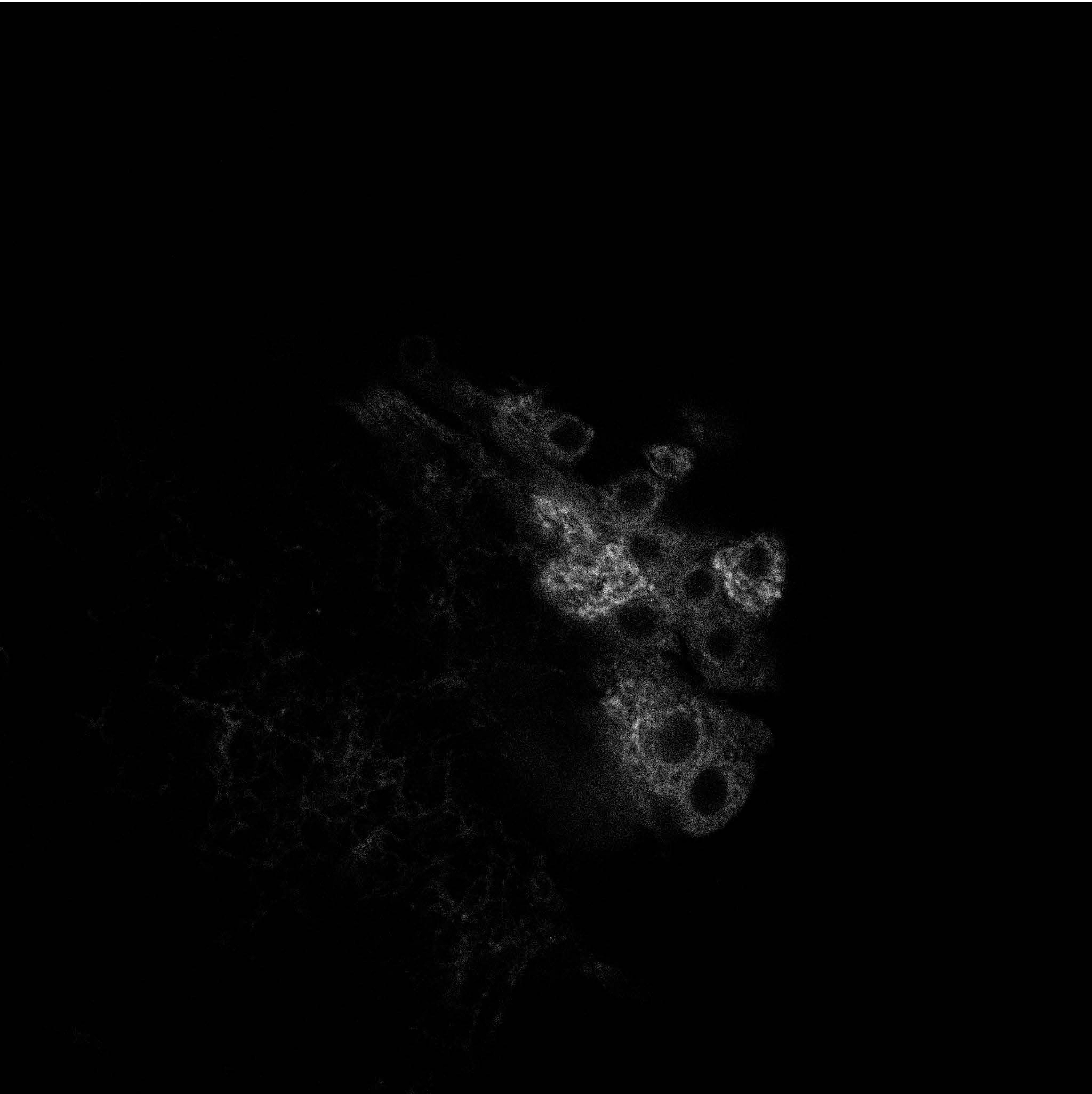

S7C_8B.tif

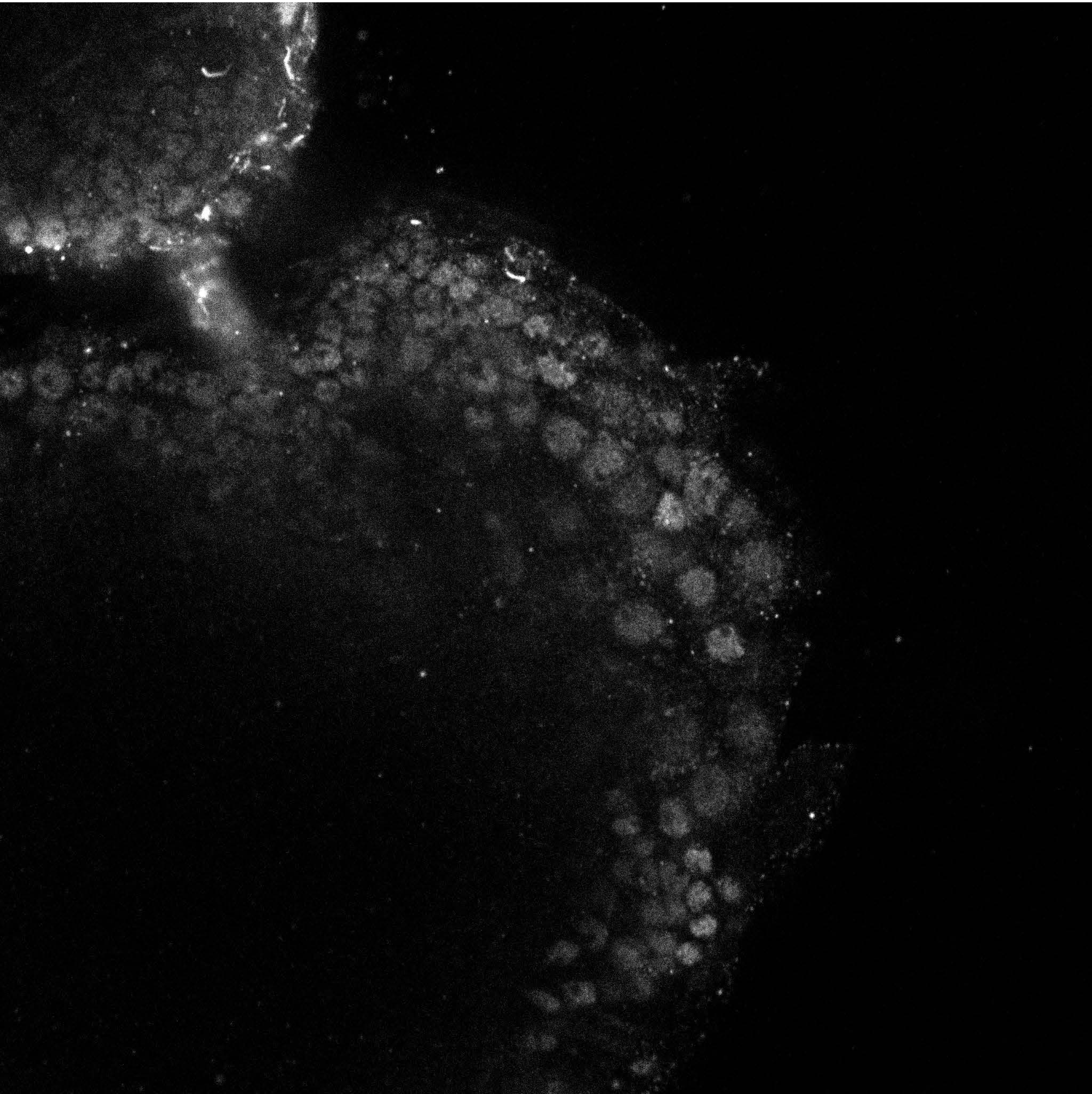

Supplement: Supplementary file 3 [file mmc3.pdf]
